# Transcriptome Analysis of *Barbarea vulgaris* Infested with Diamondback Moth (*Plutella xylostella*) Larvae

**DOI:** 10.1371/journal.pone.0064481

**Published:** 2013-05-16

**Authors:** Xiaochun Wei, Xiaohui Zhang, Di Shen, Haiping Wang, Qingjun Wu, Peng Lu, Yang Qiu, Jiangping Song, Youjun Zhang, Xixiang Li

**Affiliations:** Institute of Vegetables and Flowers, China Academy of Agricultural Sciences, Beijing, China; University of Georgia, United States of America

## Abstract

**Background:**

The diamondback moth (DBM, *Plutella xylostella*) is a crucifer-specific pest that causes significant crop losses worldwide. *Barbarea vulgaris* (Brassicaceae) can resist DBM and other herbivorous insects by producing feeding-deterrent triterpenoid saponins. Plant breeders have long aimed to transfer this insect resistance to other crops. However, a lack of knowledge on the biosynthetic pathways and regulatory networks of these insecticidal saponins has hindered their practical application. A pyrosequencing-based transcriptome analysis of *B. vulgaris* during DBM larval feeding was performed to identify genes and gene networks responsible for saponin biosynthesis and its regulation at the genome level.

**Principal Findings:**

Approximately 1.22, 1.19, 1.16, 1.23, 1.16, 1.20, and 2.39 giga base pairs of clean nucleotides were generated from *B. vulgaris* transcriptomes sampled 1, 4, 8, 12, 24, and 48 h after onset of *P. xylostella* feeding and from non-inoculated controls, respectively. *De novo* assembly using all data of the seven transcriptomes generated 39,531 unigenes. A total of 37,780 (95.57%) unigenes were annotated, 14,399 of which were assigned to one or more gene ontology terms and 19,620 of which were assigned to 126 known pathways. Expression profiles revealed 2,016–4,685 up-regulated and 557–5188 down-regulated transcripts. Secondary metabolic pathways, such as those of terpenoids, glucosinolates, and phenylpropanoids, and its related regulators were elevated. Candidate genes for the triterpene saponin pathway were found in the transcriptome. Orthological analysis of the transcriptome with four other crucifer transcriptomes identified 592 *B. vulgaris*-specific gene families with a *P*-value cutoff of 1e^−5^.

**Conclusion:**

This study presents the first comprehensive transcriptome analysis of *B. vulgaris* subjected to a series of DBM feedings. The biosynthetic and regulatory pathways of triterpenoid saponins and other DBM deterrent metabolites in this plant were classified. The results of this study will provide useful data for future investigations on pest-resistance phytochemistry and plant breeding.

## Introduction

The diamondback moth (DBM), *Plutella xylostella* (Lepidoptera), is a well-known destructive insect pest of crucifer crops worldwide. DBM reproduces vigorously and can produce up to 20 generations per year in tropical regions [1], indicating rapid evolutionary adaptability. Not only have its populations evolved significant resistance to almost every synthetic insecticide applied to it, DBM was also the first species to develop resistance to *Bacillus thuringiensis* (*Bt*) insecticidal proteins in the field [2]. Its ability to cause tremendous damage and to rapidly evolve insecticide resistance pose significant challenges to the crucifer industry.

Brassicaceae (Cruciferae) comprises a diverse group of 350 genera and over 3,500 species, including many economically important crops, such as oilseeds, radish, cabbages, Chinese cabbage, and many other vegetables. To the best of our knowledge, *Barbarea vulgaris* is the only crucifer species resistant to DBM. *Barbarea vulgaris* can be divided into two types: the pubescent (P) type has more trichomes on the leaf and is susceptible to DBM, while the glabrous (G) type has fewer trichomes and is resistant to DBM and many other insect pests [3]. G-type *B. vulgaris* is the only crucifer known to synthesize saponins. The five saponins identified in *B. vulgaris* include 3-*O*-cellobiosyl-cochalic acid, 3-*O*-cellobiosyl-oleanoic acid, 3-*O*-cellobiosyl-gypsogenin, 3-*O*-cellobiosyl-hederagenin and 3-*O*-cellobiosyl-4-epihederagenin; some of these saponins are believed to be the main regents responsible for the insect resistance of *B. vulgaris* [3], [4], [5].

Saponins are a class of triterpenoid glycosides found in a variety of plants [6]. They are composed of a triterpenoid-derived aglycon (sapogenin) and a covalently-linked sugar chain. Various biological functions have been identified among different saponins, which most commonly serve as plant defense compounds against pathogens and herbivores [7] because of their significant variability in molecular structure. Many saponins have important medicinal value, including anti-tumorigenic and immunomodulatory effects [8], [9], [10], [11]. Progress toward understanding the complete biosynthetic pathway of saponins has been hindered because of the diverse molecular structure and complexity of catalytic enzymes, particularly those from the cytochrome P450 (P450) and glycosyltransferase (GT) superfamilies.

Glucosinolates, a class of crucifer-specific compounds containing sulfur and nitrogen derived from glucose and amino acids, can be degraded to components that are toxic to insects. However, DBM produces glucosinolate sulfatase, which desulfates glucosinolates and prevents their degradation [12]. The crucifer and DBM system is an excellent model of co-evolution between plants and insects and has been investigated in *Arabidopsis* [13]. However, mismatches in phenology have limited the establishment of a resistance/counter-adaptation arms race between *Arabidopsis thaliana* and DBM. *Barbarea vulgaris* is a perennial crop that has been exposed to DBM long enough to have developed resistance [14]. Genomic research can provide data on the defense strategies of *B. vulgaris* against insect herbivory.

In this study, transcriptomes of *B. vulgaris* under DBM herbivory were generated by Illumina/Solexa pyrosequencing, after which the pathways affected by DBM were transcriptionally profiled. The genes involved in triterpenoid saponin synthesis were detected and analyzed. *Barbarea vulgaris*-specific transcripts were compared to four sequenced genomes from Cruciferae.

## Results

### Transcriptome generation and assembly

**Figure 1:**
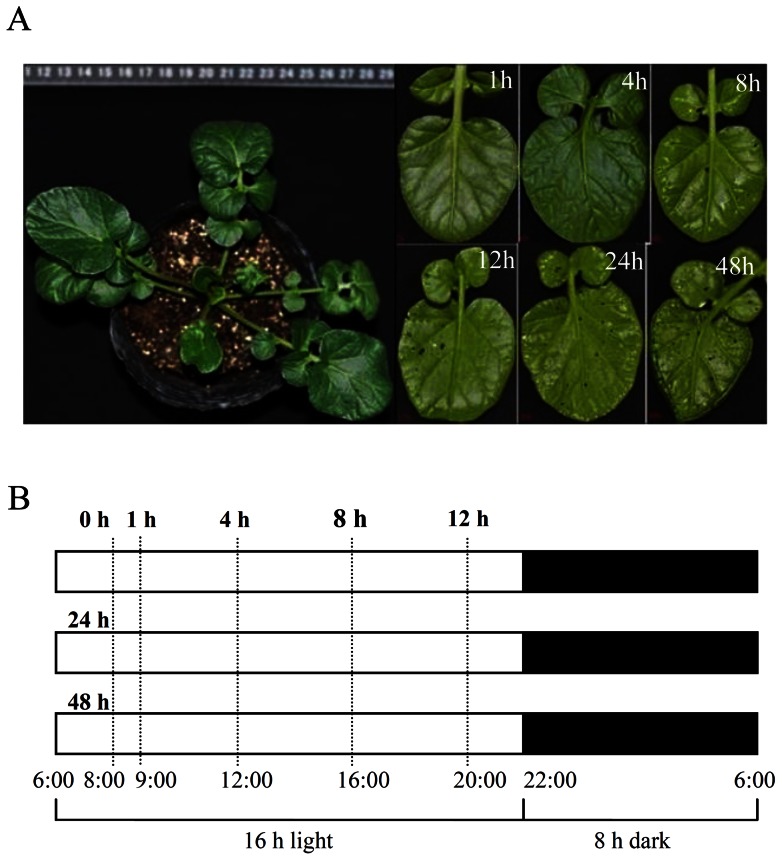
G-type *Barbarea vulgaris* and samples used for transcriptome sequencing. **A.** Plant status. A whole plant and leaf samples are shown before the experiment (left) and 1, 4, 8, 12, 24, and 48 h after being infested by *Plutella xylostella* (right), respectively. **B.** Sketch showing sampling times.

Two-month old *B. vulgaris* seedlings were planted in an artificial climate chamber to limit environmental effects then inoculated with second-instar larvae of *P. xylostella*. Insect-damaged leaves from five individual plants were sampled for RNA extraction 1, 4, 8, 12, 24, and 48 h post inoculation (hpi). Non-inoculated plant leaves were used as the control. Herbivorized leaves and their sampling times are depicted in Figure 1. Using Illumina/Solexa pair-end sequencing, a total of 106,093,082 clean reads containing more than 9.5 giga base pairs of clean nucleotides were obtained. Table 1 and Figure S1 summarize the 33,271–39,270 unigenes assembled in the treatments. All of the unigenes from the seven samples were assembled into a single set of 39,531 non-redundant unigenes. Unigene length increased significantly to a mean length of 815 nt from 509–522 nt in the separate samples (Table 1, Figure S1). Over 85% of the unigenes were clustered, which means they were more reliable than the singletons in separate samples (Table 1). This set of unigenes was used as a reference transcriptome for further analysis.

**Table 1:** Sequence and assembly summary of *Barbarea vulgaris* transcriptomes.

Sample	Total raw reads	Clean reads	Clean nucleotides	Unigene	Mean length (nt)	Clusters	Singletons
NF^a^	28,064,218	26,590,648	2,393,158,320	38,263	522	429	37,834
1 hF^b^	13,515,456	13,515,456	1,216,391,040	37,930	542	325	37,605
4 hF	13,172,054	13,172,054	1,185,484,860	38,251	531	417	37,834
8 hF	12,912,422	12,912,422	1,162,117,980	38,047	540	336	37,711
12 hF	13,683,250	13,683,250	1,231,492,500	39,270	538	356	38,914
24 hF	12,836,672	12,836,672	1,155,300,480	33,271	509	252	33,019
48 hF	13,382,580	13,382,580	1,204,432,200	33,947	524	279	33,668
Total^c^	107,566,652	106,093,082	9,548,377,380	39,531	815	34,006	5,525

a Non-feeding control.

b hF, hours of of *Plutella xylostella* feeding.

c Combined data from all seven treatments.

### Functional annotation and pathway analysis

**Figure 2:**
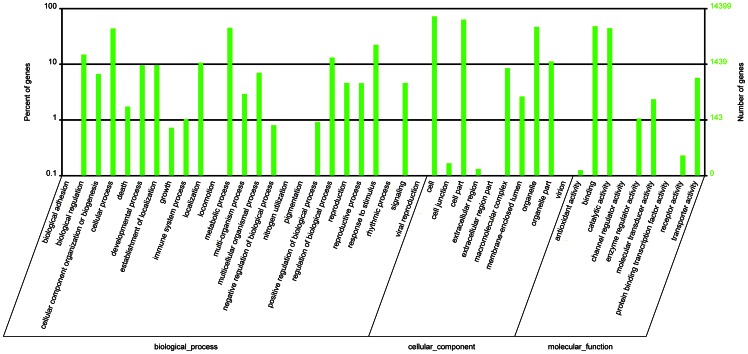
Gene Ontology distribution of functional categories of genes in the transcriptome of *Barbarea vulgaris*.

We screened the unigene sequences against the NCBI non-redundant (Nr), SwissProt, Clusters of Orthologous Groups of proteins (COGs), and Kyoto Encyclopedia of Genes and Genomes (KEGG) pathway protein databases using BLASTX (*e*-value <0.00001). Protein function was predicted from the annotations of the most similar proteins in those databases. A total of 36,133 (95.6%) and 22,588 (57.1%) of the 39,531 unigenes were annotated based on similarity to the Nr and SwissProt databases, respectively (Tables S1 and S2). Gene functions were further classified using Gene Ontology (GO) analysis. As shown in Figure 2, 14,399 unigenes were assigned to one or more GO terms. A total of 44 GO terms were attributed to one of the three GO ontologies: biological process (25 GO terms), cellular component (10 GO terms), or molecular function (nine GO terms). The largest GO terms found in the “biological process” ontology were “cellular process” and “metabolic process,” comprising 43.7% and 45.0% of the GO-termed unigenes, respectively. In the “cellular component” and “molecular function” ontologies, the top terms were “cell” (72.1%) and “binding” (47.9%).

**Figure 3:**
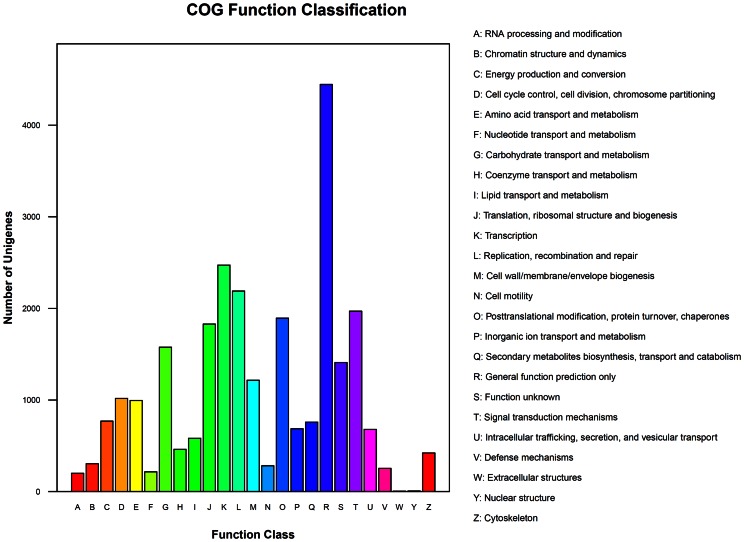
COG function classification of the *Barbarea vulgaris* transcriptome.

COGs were delineated by comparing the deduced protein sequences of the transcriptome. Figure 3 shows that 13,118 unigenes were assigned to one or more COG functional classes. The most abundant class was “general function prediction only,” including 4,444 (33.9% of annotated COGs) unigenes, followed by the classes “transcription” (2,472; 18.8%) and “replication, recombination and repair” (2,189; 16.7%). KEGG metabolic pathway analysis revealed that 19,620 (49.6%) unigenes could be assigned to 126 pathways (Table S3). Secondary metabolic pathways, such as those for saponin, terpenoid, and glucosinolate biosynthesis, which are putatively involved in resistance to *P. xylostella*, were further analyzed (see below).

### Overall changes in the *B. vulgaris* transcriptome in response to *P. xylostella*

**Figure 4:**
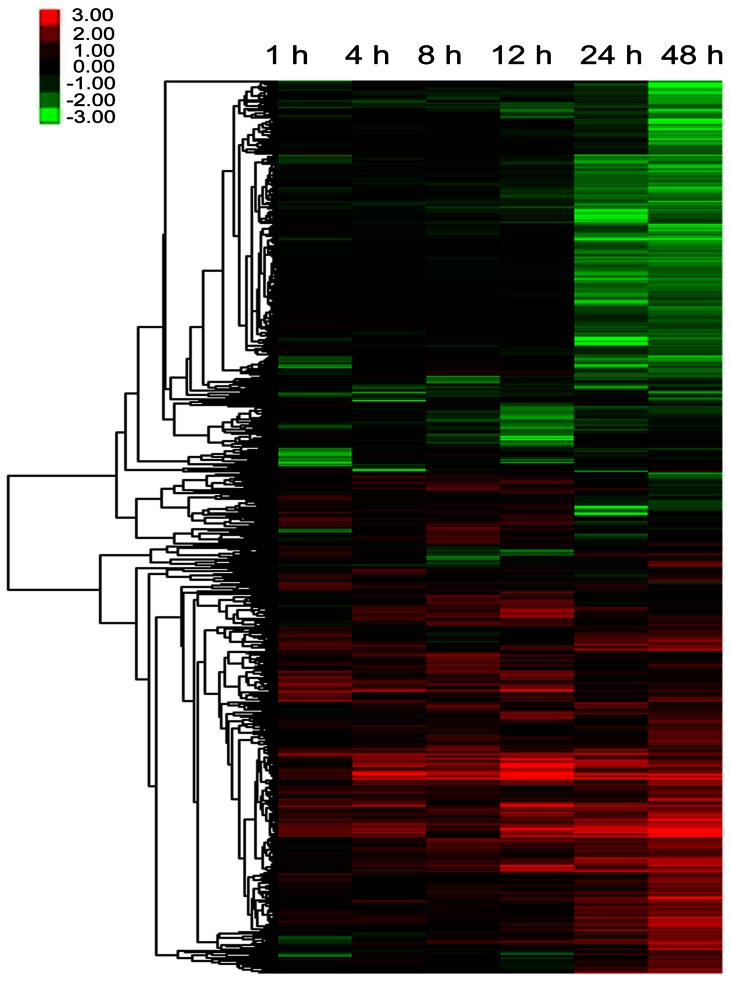
Heatmap of overall changes in the *Barbarea vulgaris* transcriptome in response to *Plutella xylostella.* The color scales represent the log_2_-transformed reads per kilobases per million reads ratios of plants 1, 4, 8, 12, 24, and 48 h after diamondback moth herbivory compared with the uneaten. The inset (upper left) shows the color bar. The trees on the left were generated using Cluster 3.0.

The reads per kilo bases per million reads (RPKM) of each unigene were used for expression level analysis. The unigenes were considered to be differentially expressed when the RPKM between treatments and the control displayed a more than twofold change with a false discovery rate (FDR) of less than 10^−3^. Table 2 and Figure S2 show that the number of differentially expressed transcripts reached 3,356 (2,102 up- and 1,254 down-regulated) by 1 hpi, possibly indicating early responsive genes, but decreased to 2,573 (2016 up- and 557 down-regulated) by 4 hpi, demonstrating decreased expression of some of the early responsive genes. Differentially expressed transcripts then increased gradually to 9,855 (4,685 up- and 5,170 down-regulated) after 48 h of DBM herbivory, representing the activation of secondary responsive genes. The majority (∼60%) of differentially expressed transcripts displayed the same directional change (down or up) throughout the time course analyzed, a fraction of transcripts reversed their expression profiles over time, and a small portion of the transcripts undulated their expression patterns (Figure 4). Interestingly, the number of down-regulated genes increased dramatically from 1,615 at 12 h to 5,188 at 24 h then remained constant until 48 h hpi (Table 2). However, most of the genes that were down-regulated later were also trending downward in expression at 12 h hpi and before (Figure 4), but they did not reach our threshold levels and thus were not listed in Table 2.

**Table 2:** Summary of differentially expressed genes in *Barbarea vulgaris* herbivorized by *Plutella xylostella.*

	1 h	4 h	8 h	12 h	24 h	48 h
Up-regulated	2102	2016	2781	3693	3753	4685
Down-regulated	1254	557	975	1615	5188	5170
Total	3356	2573	3756	5308	8871	9855

**Figure 5:**
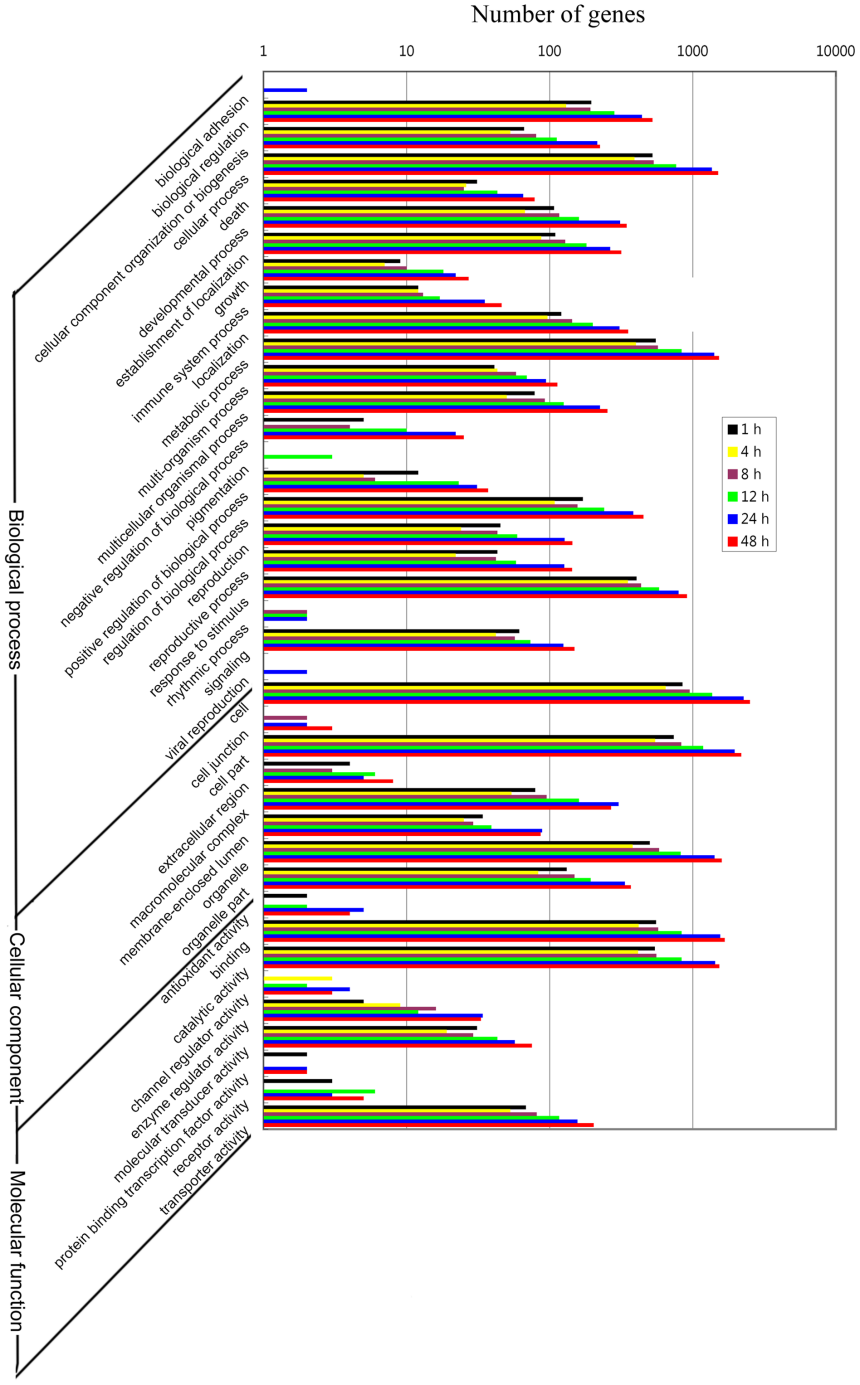
Distribution of differentially expressed genes in *Barbarea vulgaris* based on Gene Ontology functional categories. The differentially expressed genes are those displaying a more than twofold change with a false discovery rate less than 10^−3^ in at least one time point.

GO annotation revealed that these differentially expressed transcripts mainly fell into GO terms of “biological regulation,” “cellular process,” “developmental process,” “immune system process,” “metabolic process,” “regulation of biological process,” “response to stimulus,” and “signaling” from the “biological process” subgroup, as well as “binding,” “catalytic activity,” and “transporter activity” from the “molecular function” subgroup (Figure 5). These genes are probably induced for resistance and anti-insect metabolite synthesis/regulation/transportation and also possibly for wound healing. The down-regulated genes appeared after 24 h hpi mainly comprised protein kinases and genes involved in ATP-dependent metabolism and transportation, indicating that after prolonged insect herbivory, energy-consuming cell activities were suppressed and the replenishment of intracellular contents slowed to conserve material and energy for damage repair and anti-insect defenses.

### *Plutella xylostella-*induced transcription factor genes

Transcription factors serve as important regulators of biotic and abiotic stress responses by turning on/off immune system and secondary metabolism pathways. To gain insights into the possible regulatory processes elicited by DBM feeding, we analyzed the expression profiles of genes annotated as “regulation of gene expression.” A total of 403 transcripts were differentially expressed in at least one time point. One hundred eighteen were differentially expressed at more than three time points, including 10 WRKY, 15 zinc finger (ZF), 1 MYB, 12 NAC, 6 helix–loop–helix (bHLH), 18 ethylene response factors (ERFs), 1 BEL, 1 elongated hypocotyl 5 (HY5), 1 TCP, and 1 squamosa promoter-binding-like (SPL) transcription factor, as well as 36 undefined DNA-binding proteins.

**Figure 6:**
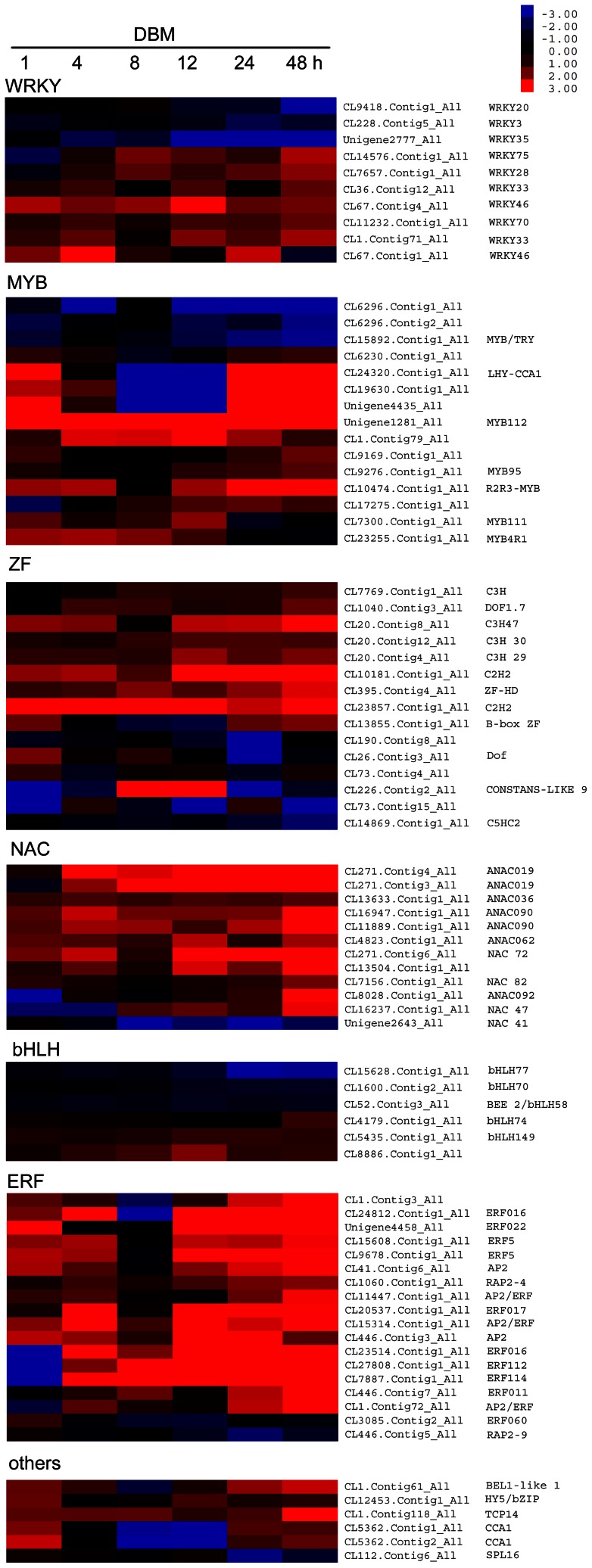
Heatmap of differentially expressed transcription factors in *Barbarea vulgaris* herbivorized by *Plutella xylostella.* The transcription factor-encoding genes were retrieved from the annotated Gene Ontology. The heatmap shows genes that are differentially expressed in at least three time points. The color bar is shown at the upper right. Gene IDs and names are on the right (see Table S1 for detailed gene information).

Figure 6 summarizes the expression responses of some important transcription factors. WRKY transcription factors in plants are one of the largest families of zinc finger transcription factors and modulate development as well as responses to abiotic stresses, wounding, pathogens, and herbivore attack [15], [16]. In the present study, four and three WRKYs were consistently up- and down-regulated, respectively, during DBM herbivory. Other zinc finger-containing transcription factors, four C3H, two C2H2, two Dof, a B-box, a constant, and several other proteins were also differentially expressed. MYB proteins are another large family of regulators that control development, metabolism, and responses to biotic and abiotic stresses [17]. R2R3-MYBs regulate the phenylpropanoid biosynthetic pathway and enhance defenses against insect herbivores [18], [19], [20]. Of the 15 differentially expressed MYB genes, six were up-regulated and three were down-regulated; another six displayed mixed expression. NAC transcription factors have been shown to regulate plant development, phytohormone signaling, and abiotic stress responses [21], [22], [23], [24]. In this study, 12 NAC genes were differentially expressed, of which eight were up-regulated and one was constantly down-regulated. Basic bHLH transcription factors regulate plant cell and tissue development, as well as phytohormone signaling [25], [26]. Three of the six differentially expressed genes in this study were consistently up- or down-regulated. ERFs integrate into the jasmonic acid (JA), salicylic acid (SA), and ethylene signal transduction pathways and mediate disease, drought, and cold resistance [27], [28], [29]. The majority of the DBM-affected ERF genes were induced. Other differentially expressed transcription factors were involved in circadian rhythms (e.g., HY5 and CCA1) and developmental process (e.g., BEL1, TCP14, and SPL16) [30], [31], [32], [33].

### *Plutella xylostella-*induced phytohormone-related genes

**Figure 7:**
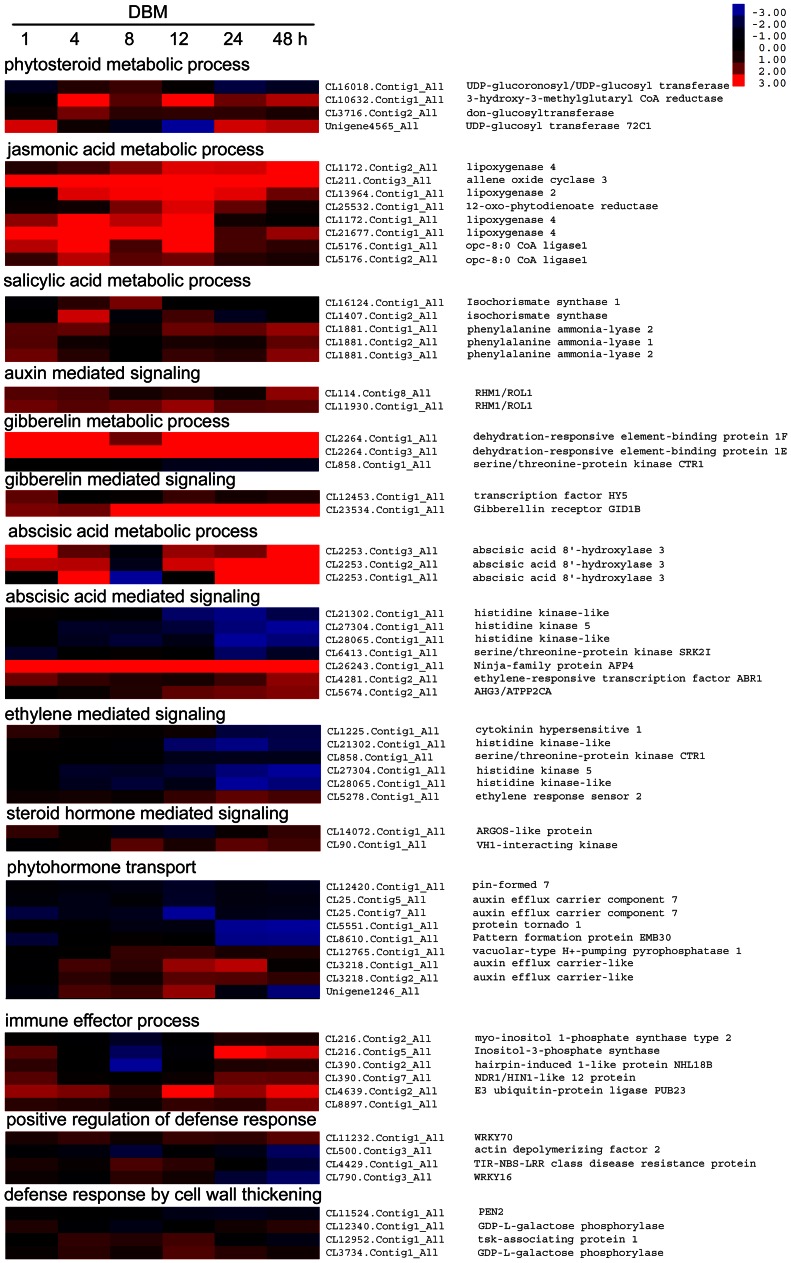
Heatmap of differentially expressed phytohormone and immune-related genes in *Barbarea vulgaris* herbivorized by *Plutella xylostella*. Genes involved in metabolism, signal transduction, and responses to phytohormone signaling were identified based on their annotated Gene Ontology categories. Genes differentially expressed in at least three time points were used to generate the heatmap. The color bar is at the upper right. Gene IDs and names are on the right (see Table S1 for detailed gene information).

Phytohormones mediate the perception of insect signals and elicitation of defenses during insect herbivory. We compared the transcript profiles associated with defense signals and phytohormones (Figure 7). Several signaling pathways, including those of JA, SA, and ethylene, are believed to orchestrate the induction of insect defenses [34], [35]. These pathways were overrepresented in DBM-affected transcriptomes. The genes of JA and SA pathways were up-regulated whereas those of the ethylene-mediated signaling pathway were down-regulated. This phenomenon was consistent with the finding that JA and SA induce plant resistance to herbivory whereas ethylene signaling reduces it [36].

Auxin- and gibberellin- (GA) mediated signaling pathways were up-regulated but not over-represented (Figure 7). Two transcripts homologous to RHM1/ROL1, which is involved in the auxin-mediated signaling pathway, were significantly up-regulated. RHM1/ROL1 is a rhamnose synthase protein that converts UDP-D-glucose to UDP-L-rhamnose; the *rol1* mutant shows a modified flavonol glycosylation profile [37], [38]. The asymmetric accumulation of flavonols further affects auxin transport and cell wall construction [37], [39]. Our transcriptome data indicated that the flavonol syntheses–auxin transport–cell wall formation cascade was also involved in the herbivory response of *B. vulgaris*. In the GA synthesis and signaling pathway, a GA receptor gene *GID1B*, which has maximum binding ability and affinity to GA4 [40], [41], [42], and two dehydration-responsive element-binding proteins (DREB1E and DREB1F), which respond to dehydration [43], were significantly up-regulated. This result was due to the fact that herbivory of leaves can enhance water loss.

Three transcripts of *abscisic acid 8′-hydroxylase 3*, which is involved in the oxidative degradation of abscisic acid (ABA) [44], were up-regulated at all time points except 8 hpi (Figure 7). In the ABA signaling pathway, all of the up-regulated genes, including *Ninja-family protein AFP4, Ethylene-responsive transcription factor ABR1*, and *AHG3/ATP2CA*, were negative regulators [45], [46], [47]. These results indicated that ABA metabolism and the ABA-mediated signaling pathway were depressed by DBM herbivory in *B. vulgaris*.

### *Plutella xylostella-*induced secondary metabolic pathways

**Figure 8:**
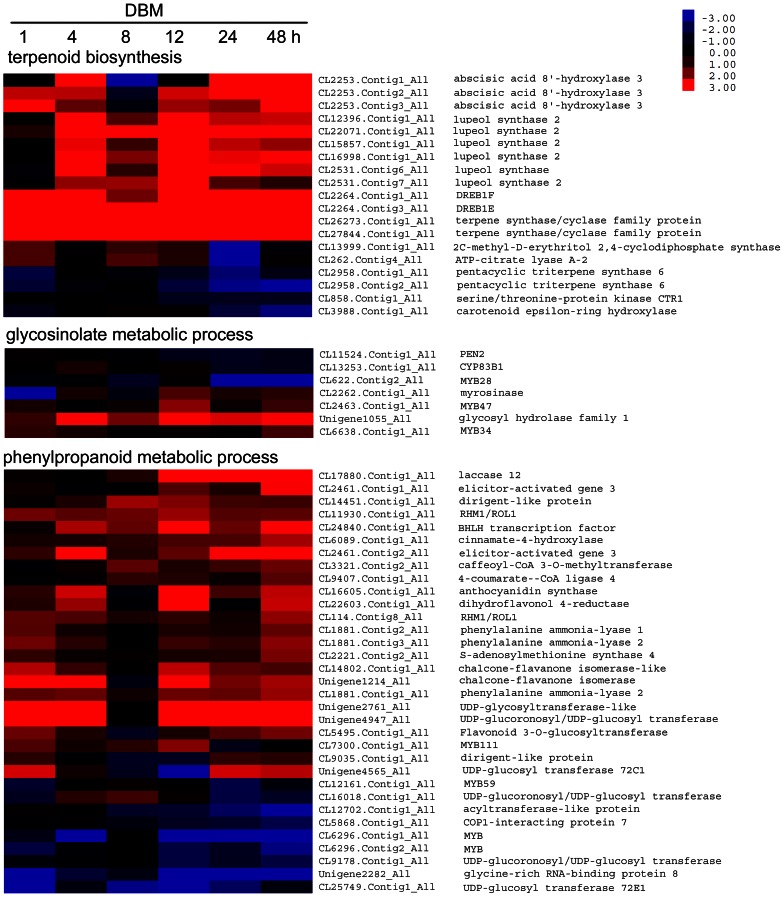
Heatmap of differentially expressed secondary metabolism pathway genes in *Barbarea vulgaris* herbivorized by *Plutella xylostella*. Genes involved in the secondary metabolic pathways of terpenoids, glucosinolates, and phenylpropanoids were isolated based on their Gene Ontology annotations. Genes differentially expressed in at least three time points were used to generate the heatmap. The color bar is at the upper right. Gene IDs and names are on the right (see Table S1 for detailed gene information).

Production of secondary metabolites is one of the most common plant responses to insect attacks. The genes of the GO term “secondary metabolic process” were extracted from seven transcriptomes. A total of 148 transcripts were differentially expressed in at least one time point. Pathways involved in terpenoid, glucosinolate, and phenylpropanoid syntheses, which are believed to be affected by herbivory, were further analyzed. In the terpenoid metabolic pathway, 34 transcripts were differentially expressed in at least one time point. Nineteen were differentially expressed in more than three time points and were used in further studies. As shown in Figure 8, three homologs of *abscisic acid 8′-hydroxylase 3* (CL2253.Contig1, 2, and 3), which belonging to the cytochrome P450 monooxygenase (CYP) 707 family and involved in the oxidative degradation of ABA and dehydration and rehydration responses [48], [49], were up-regulated at all time points except 8 hpi. Six *oxidosqualene cyclase* (OSC) genes (i.e., lupeol synthase, CL2531.Contig6; lupeol synthase 2, CL12396.Contig1, CL22071.Contig1, CL15857.Contig1, CL16998.Contig1, and CL2531.Contig7), which are key enzymes in the cyclization of 2,3-oxidosqualene in triterpenoid biosynthesis, were induced 4 h after herbivory [50], [51]. Two other *terpene synthase/cyclase* family protein (CL26273.Contig1 and CL27884.Contig1) and two *abiotic stress-responsive transcription factor* (*DREB1E*, CL2264.Contig3 and *DREB1F*, CL2264.Contig1) genes were strongly up-regulated at all time points throughout the experiment [52]. In contrast, two *pentacyclic triterpene synthase 6* genes (CL2958.Contig1 and 2) and a *carotenoid epsilon-ring hydroxylase* (CL3988.Contig1) gene, which catalyze the formation of lutein from alpha-carotene [53], were down-regulated. These results indicated that triterpenoids, not tetraterpenes, responded to insect herbivory in *B. vulgaris*.

Glucosinolates and their autolysis products are oviposition stimulants for DBM [54]. During DBM feeding, a *glycosyl hydrolase family 1* (Unigene1055) gene and a *myrosinase* (CL2262.Contig1) gene were up- or partially up-regulated; these genes are involved in the catalysis of glucosinolates to toxic compounds, such as nitriles, isothiocyanates, epithionitriles, and thiocyanates [55]. An atypical *myrosinase* gene (PEN2, CL11524.Contig1), which confers antifungal defense [56], was down-regulated. Also down-regulated was a *cytochrome P450 monooxygenase* gene (CYP83B1, CL13253.Contig1), which catalyzes indole glucosinolate biosynthesis [57].

In the phenylpropanoid metabolic process pathway, 33 genes were differentially expressed at more than three time points (Figure 8). The significantly up-regulated genes, including three *Phenylalanine ammonia-lyase* (*PAL*) homolog genes (PAL 1, CL1881.Contig2; PAL 2, CL1881.Contig1 and 3) catalyzed the deamination of L-phenylalanine to form cinnamic acid, which is the first step of the phenylpropanoid pathway and a key regulation point between primary and secondary metabolism [58]. A *cinnamate-4-hydroxylase* (CL6089.Contig1) gene and a *4-coumarate-CoA ligase 4* (CL9407.Contig1) gene were also up-regulated; these encode enzymes that catalyze the second (cinnamic acid to *p*-coumaric acid) and third (*p*-coumaric acid to 4-coumaroyl-CoA) steps of the phenylpropanoid pathway [59], [60], [61]. These three steps are often referred to as the “general phenylpropanoid pathway” because their products are shared and further catalyzed, leading to flavonoids, anthocyanins, monolignols, sinapate esters, and lignans.

Caffeoyl-CoA 3-*O*-methyltransferase (CL3321.Contig2) catalyzes key steps in the biosyntheses of monolignols and sinapates, which serve as building blocks of plant lignin [62]. Chalcone-flavanone isomerase (CL14802.Contig1 and Unigene1214) is a key enzyme in flavonoid and anthocyanin biosyntheses [63], [64]. Dihydroflavonol 4-reductase (DFR; CL22603.Contig1) is a rate-limiting enzyme in anthocyanin and condensed tannin (proanthocyanidin) biosyntheses that catalyzes the reduction of dihydroflavonols to leucoanthocyanins [65], [66]. *DFR* is induced by simulated herbivory in *Populus tremuloides* [66]. Anthocyanidin synthase (CL16605.Contig1) catalyzes the conversion of colorless leucoanthocyanidins to colored anthocyanidins [67]. UDP-glucose: Flavonoid 3-*O*-glucosyltransferase (CL5495.Contig1) catalyzes the transfer of the glucosyl moiety from UDP-glucose to the 3-hydroxyl group of anthocyanidins, producing the first stable anthocyanins [68]. All of these genes were up-regulated in *B. vulgaris*, indicating the overall induction of the phenylpropanoid metabolic pathway by DBM herbivory.

### Expression profiles of triterpene saponin biosynthesis pathway

**Figure 9:**
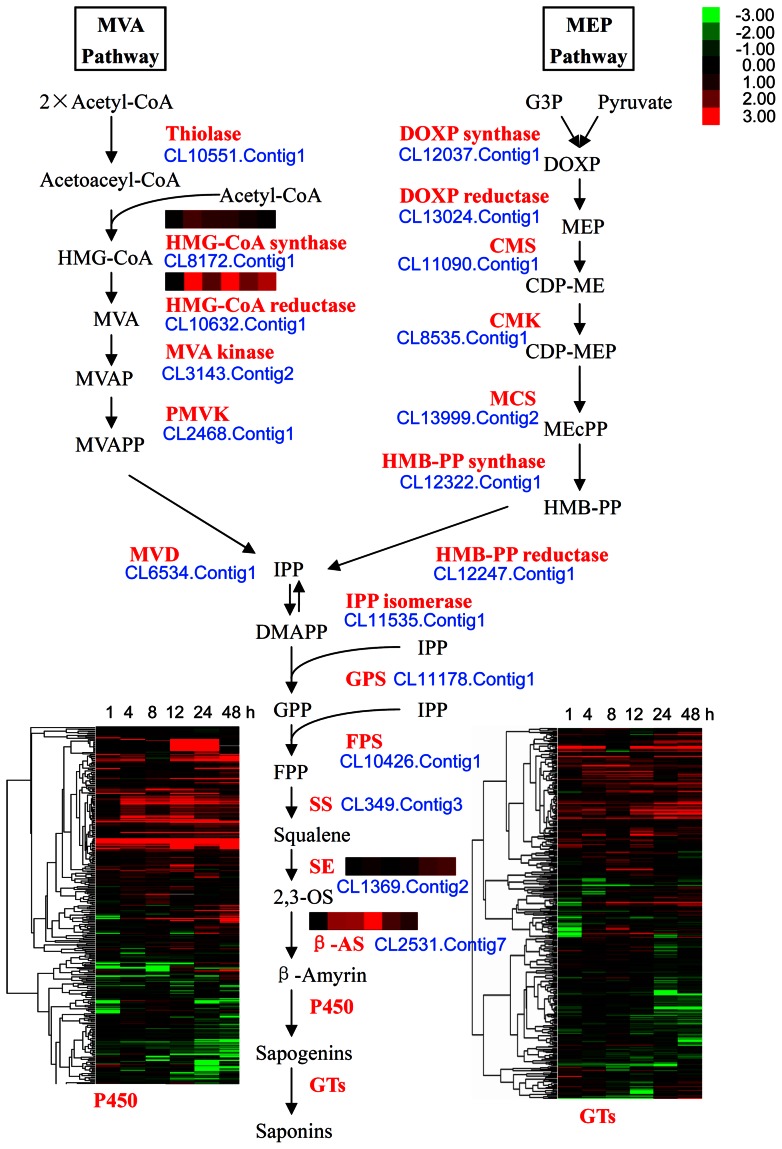
Differentially expressed genes potentially involved in the triterpene saponin synthetic pathway in *Barbarea vulgaris*. Heatmaps represent relative expression levels of different genes/families determined by RNA-seq. The color bar is at the top right. Data represent log_2_ scale ratios of reads per kilo bases per million reads of plants treated 1, 4, 8, 12, 24, and 48 h after DBM feeding compared with non-feeding controls. The cytochrome P450 (P450) and glycosyltransferase (GT) families were analyzed using Cluster 3.0. HMG-CoA, 3-hydroxy-3-methylglutaryl CoA; MVA, mevalonic acid; MVAP, mevalonic acid 5-phosphate; PMVK, phosphomevalonate kinase; MVD, MVA diphosphate decarboxylase; G3P, glyceraldehyde 3-phosphate; DOXP, deoxy-D-xylulose 5-phosphate; MEP, 2-C-methyl-D-erythritol 4-phosphate; CMS, 4-diphosphocytidyl-2-C-methyl-D-erythritol synthase; CDP-ME, 4-diphosphocytidyl-2-C-methylerythritol; CMK, 4-diphosphocytidyl-2-C-methyl-D-erythritol kinase; CDP-MEP, 4-diphosphocytidyl-2-C-methyl-D-erythritol 2-phosphate; MCS, 2-C-methyl-D-erythritol 2,4-cyclodiphosphate synthase; MEcPP, 2-C-methyl-D-erythritol 2,4-cyclodiphosphate; HMB-PP, hydroxy-2-methyl-2-diphosphate; IPP, isopentenyl diphosphate; DMAPP, dimethylallyl diphosphate; GPS, geranylgeranyl pyrophosphate synthase; FPS, farnesyl pyrophosphate synthase; GPP, geranyl diphosphate; FPP, farnesyl diphosphate; SS, squalene synthase; SE, squalene epoxidase; 2,3-OSCs, 2,3-oxidosqualene cyclases; β-AS, β-amyrin synthase.

Triterpene saponins have been identified as the major insect antifeedant produced by *B. vulgaris* [3]. The genes of all of the enzymes catalyzing the triterpene saponin pathway were represented in the transcriptomes (Table S4, Figure 9). Two pathways for isoprenoid (precursors of saponins and other metabolites) biosynthesis in plants have been identified: the cytosol mevalonic acid (MVA) and plastidial methylerythritol phosphate (MEP) pathways [69], [70]. In the MVA pathway, genes encoding HMG-CoA synthase and HMG-CoA reductase were up-regulated 4 hpi (Table S4, Figure 9). The overall output of the MVA pathway might be improved by herbivory because HMG-CoA reductase is a rate-controlling enzyme [71]. The MEP pathway was not significantly affected by DBM feeding.

Squalene epoxidase (SE) catalyzes the first oxygenation step in triterpenoid saponin biosynthesis and is one of the rate-limiting enzymes in this pathway [72]. β-Amyrin synthase (β-AS) is a key enzyme that catalyzes the cyclization of epoxysqualene into a common aglycon, the first committed step in the biosynthesis of triterpenoid saponins [73], [74]. Both SE and β-AS were significantly up-regulated by DBM herbivory (Figure 9), consistent with the finding that triterpenoid saponins respond to and defend against DBM herbivory [3].

The aglycons were subsequently modified by P450s to elaborate sapogenin diversity and then processed by GTs to introduce saccharide side chains to the sapogenin backbone and confer biological activity [6]. P450s and GTs, which are members of two of the largest and most diverse gene families in plants, are involved in numerous metabolic processes and function by catabolization and glycosylation of a vast array of metabolites and xenobiotics [75]. A total of 334 *P450* and 423 *GT* gene transcripts were found in the *B. vulgaris* transcriptome (Table S4). Among these transcripts, 128 (38.3%) *P450* and 120 (28.4%) *GT* genes were up-regulated, 74 (22.2%) *P450* and 120 (28.4%) *GT* genes were down-regulated, and 47 (14.1%) *P450* and 64 (15.1%) *GT* genes had mixed expression (Table S4, Figure 9). About 63 *P450* and 64 *GT* transcripts were up-regulated at more than three time points; these transcripts probably contain the genes necessary to biosynthesize the *B. vulgaris*-specific insect resistance saponins.

**Figure 10:**
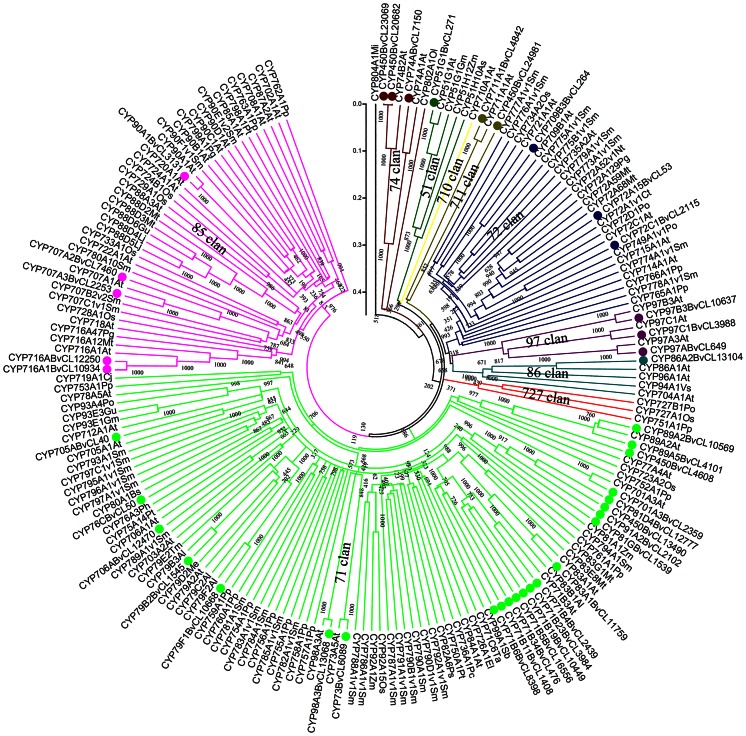
Neighbor-joining bootstrap tree of CYP450 proteins in plants. Amino acid sequences of 148 plant cytochrome P450s (CYPs), including 105 sequences representative of the 10 CYP families in land plant and 41 P450s from the *B. vulgaris* transcriptome (marked with dots), were used to construct the phylogenetic tree. Multiple alignments were performed with Clustal W, and the tree was generated with MEGA 5. The 10 CYP clades are labeled. Bootstrap values are shown at the nodes. Al, *Arabidopsis lyrata*; As, *Avena strigosa*; At, *Arabidopsis thaliana*; Bs, *Berberis stolinifera*; Cj, *Coptis japonica*; Ct, *Catharanthus roseus*; El, *Euphorbia lagascae*; Gu, *Glycyrrhiza uralensis*; Lj, *Lotus japonicas*; Me, *Manihot esculenta*; Mi, *Micromonas pusilla*; Mt, *Medicago truncatula*; Nt, *Nicotiana tabacum*; Os, *Oryza sativa*; Ph, *Petunia hybrida*; Po, *Populus trichocarpa*; Pp, *Physcomitrella patens*; Pt, *Pinus taeda*; Py, *Pyrus communis*; Sb, *Sorghum bicolor*; Sm, *Selaginella moellendorffii*; Ta, *Triticum aestivum*; Tm, *Triglochin maritime*; Vs, *Vicia sativa*; Zm, *Zea mays*.

Transcripts harboring the full-length coding sequence of *P450*s were isolated and compared with representative members of this superfamily from land plants. The phylogenic tree obtained showed that 41 deduced P450 proteins from *B. vulgaris* were distributed in eight of the 10 major clades of this superfamily (Figure 10), about half of which genes were enriched in the CYP71 clade, consistent with findings in other plants [75]. The presence of *B. vulgaris* members in the two other CYP clades cannot be excluded because a large number of *P450* transcripts were incomplete and not included in the phylogenetic analysis.

**Figure 11:**
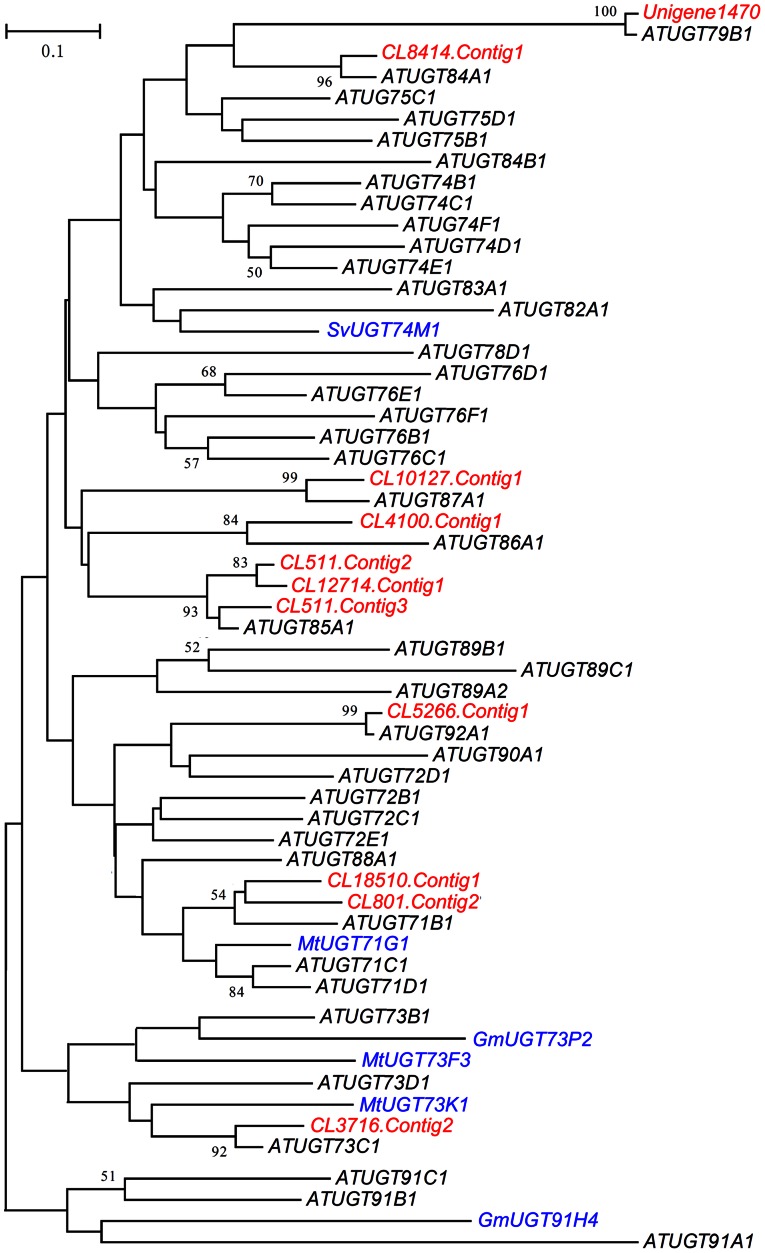
Neighbor-joining bootstrap tree of UGT proteins. The PROSITE consensus sequence of the 11 significantly up-regulated *Barbarea vulgaris UGTs* transcripts (red), as well as 41 *UGTs* representative of the *Arabidopsis* genome (black) and six reported to be involved in saponin biosynthesis (blue), were used to construct the phylogenetic tree. Multiple alignments were performed with Clustal W, and the tree was generated with MEGA 5. Bootstrap values above 50% are shown at the nodes.

To date, only three P450s have been reported to function in the biosynthesis of triterpenoid saponins. These P450s are CYP93E1 in soybean (*Glycine max*) [76], CYP51H10 in oat (*Avena strigosa*) [77], and CYP88D6 in licorice (*Glycyrrhiza uralensis*) [78], which show no regularity in molecular family distribution. *P450* genes that were up-regulated at more than three time points were distributed among the CYP clades (Figure 10). Family 1 *GTs* containing a PROSITE consensus sequence at the C-terminus were defined as UDP-glycosyltransferases (UGTs), which are the major *GT* subfamily and responsible for the glycosylation of saponins [79], [80]. Of the 64 significantly up-regulated *GT* transcripts, 11 containing the complete PROSITE consensus sequence were identified and compared with *UGTs* representative of the *Arabidopsis* genome and six ones reported to be involved in saponin biosynthesis in other plants. The phylogenic tree showed that DBM-induced *UGTs* were from the *UGT71*, *73*, *79*, *84*, *85*, *86*, *87*, and *92* families (Figure 11). The six saponin-related UTGs belonged to the *UGT71*, *73*, *74*, and *91* families; three were in the *UGT73* family [81], [82], [83], [84]. The large number and wide diversity of members of the P450 and UGT multigene families make identification of individual genes responsible for the biosynthesis of triterpenoid saponins by simple homology analysis difficult. However, the combination of expression profiles and the phylogenetic tree provided useful data.

### *Barbarea vulgaris* specific genes by comparison with other crucifer transcriptomes

**Figure 12:**
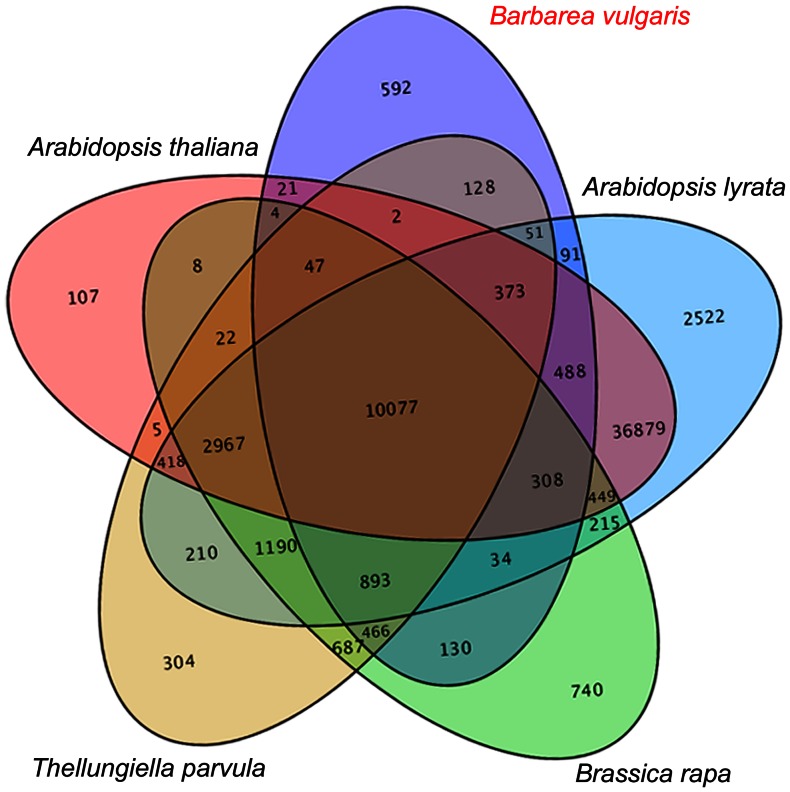
Venn diagram showing unique and shared gene families in five crucifer species.

By comparing the *B. vulgaris* transcriptome with those of four other crucifers (*A. thaliana*, *Arabidopsis lyrata*, *Thellungiella parvula*, and *Brassica rapa*), a total of 592 *B. vulgaris*-specific gene families were isolated with a *p*-value cutoff of 1e^−5^ (Table 3, Figure 12). The 592 families contained 1434 transcripts (Table S5). Based on expression level data, 537 transcripts were differentially expressed in at least one time point, and 405 were annotated to the Nr database (Table S5). Most of these genes (383 of 405) were distributed to 22 terms according to gene function (Table 4). The largest fractions of DBM-affected *B. vulgaris*-specific genes were those involved in “secondary metabolism” and “disease resistance”. In the “secondary metabolism” term, two *AtLUP2* homologous genes (CL6199.Contig1 and 2) that encode OSCs, several *Cytochrome P450* (CL24981.Contig1, CL18450.Contig1, and CL26367.Contig1) genes, and a *glycosyltransferase* (CL15906.Contig1) gene were up-regulated (Table S6) [6], [85]. *Barbarea vulgaris* specificity and the DBM-responsive expression indicated that these genes could be candidates for the synthesis of DBM-resistant saponins. Other *B. vulgaris*-specific secondary metabolism genes were involved in the glucosinolate and phenylpropanoid pathways (Table S6), which are also believed to play important roles in insect resistance.

**Table 3:** Comparison of transcriptome data in five crucifer species.

Species	No. of genes	No. of genes in families	No. of unclustered genes	No. of families	No. of unique families	Average genes per family
*Barbarea vulgaris*	36,205	19,052	17,153	13,705	592	1.39
*Arabidopsis lyrata*	126,123	106,833	19,290	57,165	2,522	1.87
*Brassica rapa*	41,173	30,815	10,358	18,237	740	1.69
*Thellungiella parvula*	27,132	23,837	3,295	17,840	304	1.34
*Arabidopsis thaliana*	78,096	71,805	6,291	52,175	107	1.38

**Table 4:** Functional distribution of *Barbarea vulgaris*-specific genes responded to diamondback moth herbivory.

Gene function	Number
DNA/RNA structure and RNA splicing	21
Transcription factor	30
Other transcription-related proteins	9
Nucleic acid metabolism	3
Translation	25
Protein–protein interactions	13
Protein metabolism	25
Protease	23
Alpha-zein	13
Crambin precursor	1
Disease resistance protein	35
Kinase	23
Photosynthetic proteins	6
Energy currency syntheses protein	9
Carbohydrate metabolism	6
Lipid metabolism	7
Secondary metabolism	46
Mineral and other metabolisms	9
Cell wall proteins	8
Signaling	14
Transport proteins	31
Retroelement	26
Protein of unknown function	22
Total	405

Nucleotide binding site–leucine-rich repeat (NBS–LRR) class disease resistance proteins belong to a classic R gene superfamily that confers innate resistance to multiple invading organisms, including insects [86], [87]. The two confirmed NBS–LRR resistance genes are *Mi-1.2* from tomato and *Vat* from melon; both of these genes confer resistance to sucking pests [88], [89], [90], [91]. To the best of our knowledge, NBS–LRR protein-induced chewing insect resistance has yet to be reported. A large collection of disease-resistance protein genes have been found specifically in *B. vulgaris*, 70% of which were up-regulated by DBM herbivory (Table S6). These results indicated that NBS-LRR proteins may also play a role in herbivore resistance.

Another large collection of *B. vulgaris*-specific genes encoded protease and ubiquitin proteins (Table S6). The protease and ubiquitin–proteasome systems belonged to two highly selective and tightly regulated proteolytic pathways responsible for protein degradation and regulate a striking variety of biological processes, including local and systemic defense responses [92], [93]. The presence of these genes in our analyses indicated that proteolytic pathways could also function in insect responses in plant.

Retroelements are important creative forces in gene duplication and genome evolution [94], [95]. In the present study, we found the expression of a large number of *B. vulgaris*-specific retroelement genes, most of which were down-regulated by DBM inoculation (Table S6); these findings indicated that retrotransposition activity was suppressed when the plants were faced with insect herbivory. A total of 13 transcripts homologous to the *alpha-zein* precursor were specific to *B. vulgaris* and up-regulated by DBM inoculation (Table S6). Alpha-zein is the major storage protein in maize [96]; its role in insect resistance remains unclear.

### Reverse transcription–PCR cloning and real-time RT–PCR confirmation of the sequence data

Reverse transcription (RT)–PCR cloning and real-time RT–PCR were performed to confirm the quality of our assembly and expression profiles. We designed primers specific to a *β-AS* and a *UGT* gene and cloned the fragments by RT–PCR. The PCR products were then sequenced using an ABI 3730. Alignment of the cDNA clones and the Illumina sequencing products showed that the 2,537 bp of *β-AS* were perfectly matched, whereas the 1,386 bp of *UGT74* fragment contained only 2 bp mismatches (Text S1). These results indicated the high quality of the transcriptome assembly.

**Figure 13:**
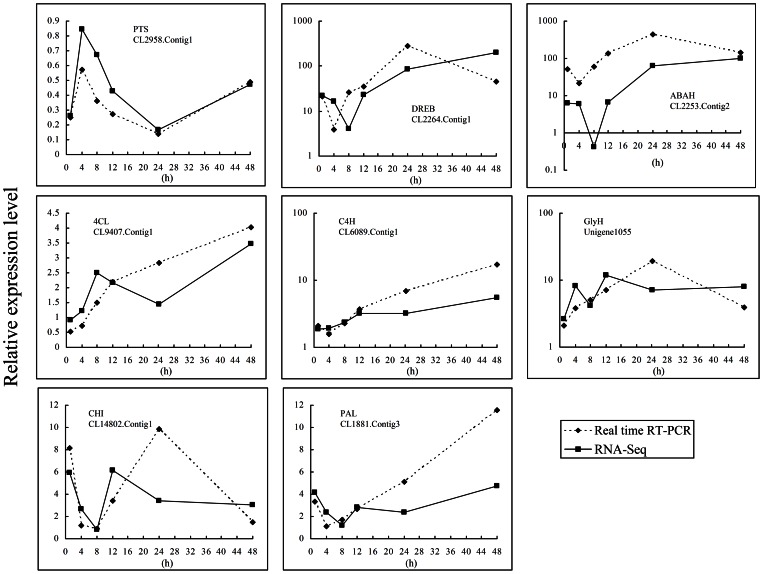
Comparison of real-time RT–PCR and RNA sequencing expression data. Eight secondary metabolic genes with different expression patterns were analyzed for RNA abundance using real-time RT–PCR. The relative expression levels in RNA sequencing (solid lines) were determined as the ratio of the reads per kilo bases per million reads of samples of *Barbarea vulgaris* 1, 4, 8, 12, 24, and 48 h after continuous herbivory by *Plutella xylostella* relative to the non-herbivorized control. Real-time RT–PCR results (dotted lines) were calculated with 2^−ΔΔCT^ using similarly-treated samples. Gene IDs and names are labeled on each panels (see Table S1 for detailed gene information).

Several secondary metabolism-related genes with different expression patterns were chosen for real-time RT–PCR analysis. As shown in Figure 13, the expression patterns obtained between the RNA sequencing and real-time RT–PCR methods were highly similar, indicating the accuracy of our transcriptome profile results.

## Discussion

*Barbarea vulgaris* as a non-model plant has increasingly been the target of plant–insect interaction studies; however, published genetic and sequence resources on this plant remain limited. The development of next-generation sequencing technology has made possible the production of genome and transcriptome sequence data of niche plants such as *B. vulgaris*. In this study, we generated an integrated transcriptome of *B. vulgaris* seedlings under different degrees of DBM herbivory. Among the 39,531 assembled unigenes, multiple transcripts could come from a single gene due to alternate splicing, and some transcripts could be partial fragments of a single gene that were not connected in contigs. The long stretches (average, 815 nt) and high quality (perfect alignment with cDNA sequences) of the assembled unigenes suggested that these transcriptome data will be valuable in facilitating future gene discoveries.

We used *B. vulgaris* seedlings subjected to different durations of DBM herbivory to determine expression profiles. We could not exclude the presence of genes responsible for photoperiod and biological rhythms among those that were differentially expressed among treatments. However, genes that were up- or down-regulated in the same direction at more than three time points could be truly affected by DBM.

A number of studies on the triterpene saponin biosynthetic pathway have been performed in *B. vulgaris* and other plants [6], [81], [97]. Overexpression of a *β-AS* gene has been reported to improve saponin accumulation in plants [73]. In the present study, one *β-AS* gene was isolated and found to be induced by DBM. The triterpenoid pathway is essential for plant development, and the enzymes upstream of OSCs are common in plants, including crucifers. Thus, the absence of saponins in most crucifer crops is due to the lack of enzymes catalyzing the modification and saccharide-chain elongation of sapogenin, specifically P450s and UGTs. Obtaining the P450 and UGT genes responsible for saponin biosynthesis is the first step in introducing insect resistance to cultivated crucifer plants. More comprehensive experiments are necessary because of the many members and wide diversity within these multigene families. Our transcripts and profiles will help to solve this problem.

Apart from saponins, many other secondary metabolites, such as terpenoids, glucosinolates, and phenylpropanoids, also confer resistance to herbivory or serve as communication signals between plants and insects. Terpenoids can kill pests directly, manipulate pest development, repel pests, and attract enemies of pests [98], [99]. We found that some genes in the terpenoid metabolic pathway were induced by DBM; the role of terpenoids in the plant–DBM community is an interesting subject worthy of further research. The phenylpropanoid pathway was significantly up-regulated by DBM in this study. Lignans and other phenylpropanoids have been shown to serve as insect feeding-deterrents and as molting modifiers or antihormonal substances [100]. While these metabolites may be induced as a form of basal resistance, the exact ecological roles of these molecules remain unknown. Glucosinolates are a class of crucifer-specific anti-insect compounds. DBM has evolved the capacity to detoxify these compounds and use them as attractive signals for oviposition [54]. After DBM herbivory, genes responsible for the catalysis of glucosinolates to toxic compounds were up-regulated (Figure 8), indicating that some evolutionary legacy of the anti-insect mechanism remains functional, although it may benefit the caterpillars.

Transcription factors are highly-efficient regulators of secondary metabolism. For example, MYB transcription factors control multiple steps in the phenylpropanoid pathway [101], [102], [103]. However, the transcription factor(s) controlling the saponin biosynthetic pathway has yet to be identified. In this study, 33 transcription factors were up-regulated consistently, and some displayed expression patterns similar to the *β-AS* gene (Figure 6). Whether or not these transcription factors include a saponin-regulating transcription factor needs more research.

JA, SA, and ethylene are defense signals that are broadly associated with various biotic and abiotic stresses [34], [35]. In the present study, these pathways were also overrepresented in the DBM-affected transcriptomes, with the JA and SA pathways being up-regulated and the ethylene pathway being down-regulated (Figure 7). These results indicated that JA and SA, but not ethylene, were more likely to elevate the basal resistance of plants to herbivory.

Species-specific genes were isolated from five crucifer plants (Figure 12) via the ortholog comparison. The *B. vulgaris*-specific set of genes identified *β-AS* as a saponin synthase and narrowed the candidates of P450s and UGTs. *Barbarea vulgaris*-specific genes encoding proteases, kinases, transporters, and transcription and translation-related proteins comprised a systemic network that induced the specialized secondary metabolism of the plant.

In conclusion, a comprehensive transcriptome analysis of *B. vulgaris* subjected to a series of DBM feedings was presented in this study for the first time to our knowledge. The long stretch unigenes with high accuracy in this transcriptome data set will be valuable in facilitating future gene discoveries. The major DBM-induced changes, as well as genes involved in secondary metabolism, expressional regulation, and signaling, were profiled, convenient the revealing of the interaction between resistant plant and pest. The biosynthetic pathway of triterpenoid saponins in this plant was classified and the candidates of the key enzymes were highlighted. These findings will provide useful information for transferring the herbivorous insect resistance property to other crops by biotechnology and molecular plant breeding.

## Materials and Methods

### Plant and insect materials

G-type *B. vulgaris* ssp. *arcuata* plants were grown in an artificial climate room. Natural daylight was supplemented with sodium lamps to maintain a minimum photosynthetically active radiation (PAR) of 225 μmol·m^−2^·s^−1^ with a photoperiod of 16∶8 h L:D. The room temperature was 25°C (light) and 20°C (dark), with a relative humidity of 60%. Seeds were surface-sterilized in 1% NaClO and germinated on glass Petri dishes. Seedlings were transplanted into a mixture of peat soil (peat: moss: perlite: vermiculite soil  = 3∶2∶1∶1) 3–4 d after germination. The seedlings were then transplanted individually into plastic pots (10 cm wide ×10 cm tall) after 2 wk, watered once every 2 d, and fertilized regularly with half-strength Hoagland nutrient solution. The plants were 6–7 wk old and still in the vegetative state when used for bioassays.

DBM (*P. xylostella*) larvae were maintained on cabbage (*Brassica oleracea*) plants in a climate-controlled room at 25°C with a 12∶12 h photoperiod and 50–60% relative humidity. For insect treatments, four DBM larvae (second or third instars) were placed on each leaf of the seedlings until harvest. The control group comprised similar plants maintained under the same conditions but without exposure to DBM larvae. Leaves from DBM-exposed and control plants were harvested 0, 1, 4, 8, 12, 24, and 48 h after the onset of herbivory. For each treatment group and time point, all of the leaves were harvested from five plants and flash-frozen in liquid nitrogen.

### RNA isolation and sequencing

Total RNA from each sample was isolated with TRIZOL (Invitrogen, Carlsbad, CA, USA) according to the manufacturer's instructions. The mRNA was purified from 10 μg of total RNA using oligo (dT) magnetic beads. After purification, the mRNA was fragmented into small pieces using divalent cations under elevated temperatures. The RNA fragments were used for first-strand cDNA synthesis with random primers. Second-strand cDNA synthesis was performed using DNA polymerase I and RNaseH (Invitrogen). These cDNA fragments went subjected to an end repair process and then ligated to adapters. The products were purified, enriched with PCR, and then used as templates for sequencing.

### Assembly

The sequencing and assembly were performed by the Beijing Genomics Institute (Shenzhen, China) using the Illumina HiSeq™ 2000 platform (Hayward, CA, USA). Raw reads were first subjected to purification by removal of adaptor reads, low-quality reads, and reads containing more than 5% unknown nucleotides. Transcriptome *de novo* assembly was performed using a short-read assembling program (Trinity) [104] to create contigs. The paired-end reads were then mapped back to the contigs. Sequences without Ns that could not be extended at either end were defined as unigenes. The TGI Clustering Tool [105] was used to assemble the unigenes from different samples to form a single non-redundant set. The following parameters were used to ensure assembly quality: a minimum of 95% identity, a minimum of 35 overlapping bases, a minimum of 35 scores, and a maximum of 30 unmatched overhanging bases at sequence ends. After clustering, the unigenes were divided into clusters (prefix CL) and singletons (prefix Unigene).

### Functional annotations

Unigenes were functionally annotated using protein sequence similarity and KEGG pathway, COG, and GO databases. We searched all unigene sequences against several protein databases (Nr, SwissProt, KEGG, and COG) using BLASTX (*e*-value <0.00001). Protein function was predicted based on the most similar proteins in those databases. Coding region sequences (CDS) of the unigenes were determined using the proteins with the highest ranks in the BLAST results, after which CDS were translated into amino sequences using the eukaryotic nuclear genetic code. Both nucleotide (5′–3′) and amino acid sequences of the unigene coding region were acquired. Unigenes that could not be aligned to any database were scanned by ESTScan [106] to identify the nucleotide sequence direction and amino sequence of the predicted CDS.

### Expression levels

Unigene expression levels were calculated using the RPKM method [107], the formula for which is RPKM(A)  = (1,000,000×C)/(N×L×1000), where RPKM(A) is the expression of gene A, C is the number of reads that uniquely align to gene A, N is the total number of reads that uniquely align to all genes, and L is the number of bases in gene A. Statistical comparison was performed as previously described [108]. FDR was used to determine the *P*-value threshold in multiple tests and analyses. Unigenes were considered differentially expressed when the RPKM between treatments and the control displayed a more than twofold change with an FDR less than 10^−3^.

### Orthological analysis

We downloaded transcriptome data for four crucifers: *Arabidopsis lyrata* (http://www.phytozome.net/alyrata.php), *Brassica rapa* (http://brassicadb.org/brad/downloadOverview.php), *Thellungiella parvula* (http://thellungiella.org/data/), and *Arabidopsis thaliana* (ftp://ftp.arabidopsis.org/home/tair/Sequences/). The CDS from these transcriptomes were translated to amino acid sequences and then subjected to BLASTP analysis with a threshold value of *P*<10^−5^. Ortholog gene families of the five species were analyzed using OrthoMCL V1.4 [109] with a *P*-value cutoff of 1e^−5^.

### Real-time RT–PCR validation and expression analysis

Total RNA samples were prepared from leaf tissue as outlined above. Experiments were performed on three independent biological and technical replicates. First-strand cDNA was synthesized from 650 ng of DNase-treated (Promega, Fitchburg, WI, USA) total RNA using ImProm-II TM Reverse Transcriptase (Promega) and diluted 20-fold for use as template. Primers for selected genes were designed using Primer Express 3.0 (Table S7). Experiments were performed using Power SYBR Green PCR Master Mix (Applied Biosystems, Foster City, CA, USA) in a StepOne™ Real-Time PCR System (Applied Biosystems). The reaction volume was 20 μL, including 10 μL of Power SYBR Green PCR master mix, 0.9 μL of 10 mM primer, 2.0 μL of the cDNA sample, and 6.20 μL of dH_2_O. The thermal cycling profile was: 95°C for 10 min; 40 cycles of 95°C for 15 s, 59°C for 15 s, 72°C for 15 s; then 95°C for 15 s, 60°C for 1 min, ramping to 95°C. Data were analyzed using StepOne™ Software v. 2.0 (Applied Biosystems). *Tubulin* expression was used as an internal control to normalize all of the data. The fold change in mRNA expression was estimated in terms of threshold cycles by the 2^−ΔΔCT^ method [110].

### Data deposition

The Illumina reads of *Barbarea vulgaris* transcriptomes have been deposited at ftp://shanjie:shanjie123@brassicadb.org.

## Supporting Information

Figure S1 **Length distribution of assembled unigenes.** 0 h, the non-inoculated control; 1 h, 4 h, 8 h,12 h, 24 h and 48 h indicate the plant tissues of 1, 4, 8, 12, 24 and 48 hour DBM feeding; All-unigene, the unigenes assembled use the mix of the 7 sequence library.
(TIF)

Figure S2 **Heat map of relative expression level between plants inoculated with DBM and non-inoculated control**. 0 h, the non-inoculated control; 1 h, 4 h, 8 h, 12 h, 24 h and 48 h indicate the plant tissues of 1, 4, 8, 12, 24 and 48 hour DBM feeding.
(TIF)

Table S1 **The statistics and annotations of unigenes.**
(RAR)

Table S2 **Statistics of annotated unigenes.**
(XLS)

Table S3 **The KEGG pathways.**
(PDF)

Table S4 **Candidate genes of saponin pathway in** ***B. Vulgaris.***
(XLS)

Table S5 **Genes specificly expressed in** ***B.Vulgaris.***
(XLS)

Table S6 **Annotation of** ***B.Vulgaris*** **specific genes.**
(XLS)

Table S7 **Primers used in this study.**
(XLS)

Text S1 **Alignment of cDNA cloning and Illumina sequencing of β-AS gene.**
(PDF)
