# Correction: Transcriptome Analysis of *Barbarea vulgaris* Infested with Diamondback Moth (*Plutella xylostella*) Larvae

**DOI:** 10.1371/annotation/83c5e6a5-2633-46d8-b138-2ac1f0f2706a

**Published:** 2013-09-10

**Authors:** Xiaochun Wei, Xiaohui Zhang, Di Shen, Haiping Wang, Qingjun Wu, Peng Lu, Yang Qiu, Jiangping Song, Youjun Zhang, Xixiang Li

The institutional affiliation for all authors is incorrect. The correct affiliation is: Institute of Vegetables and Flowers, Chinese Academy of Agricultural Sciences, Beijing, China. 

