# Supplementary figures and images for: Transcriptome Analysis of Barbarea vulgaris Infested with Diamondback Moth (Plutella xylostella) Larvae
Source: PLoS One. 2013 May 16;8(5):e64481. doi: 10.1371/journal.pone.0064481 (PMC3655962; doi:10.1371/journal.pone.0064481)

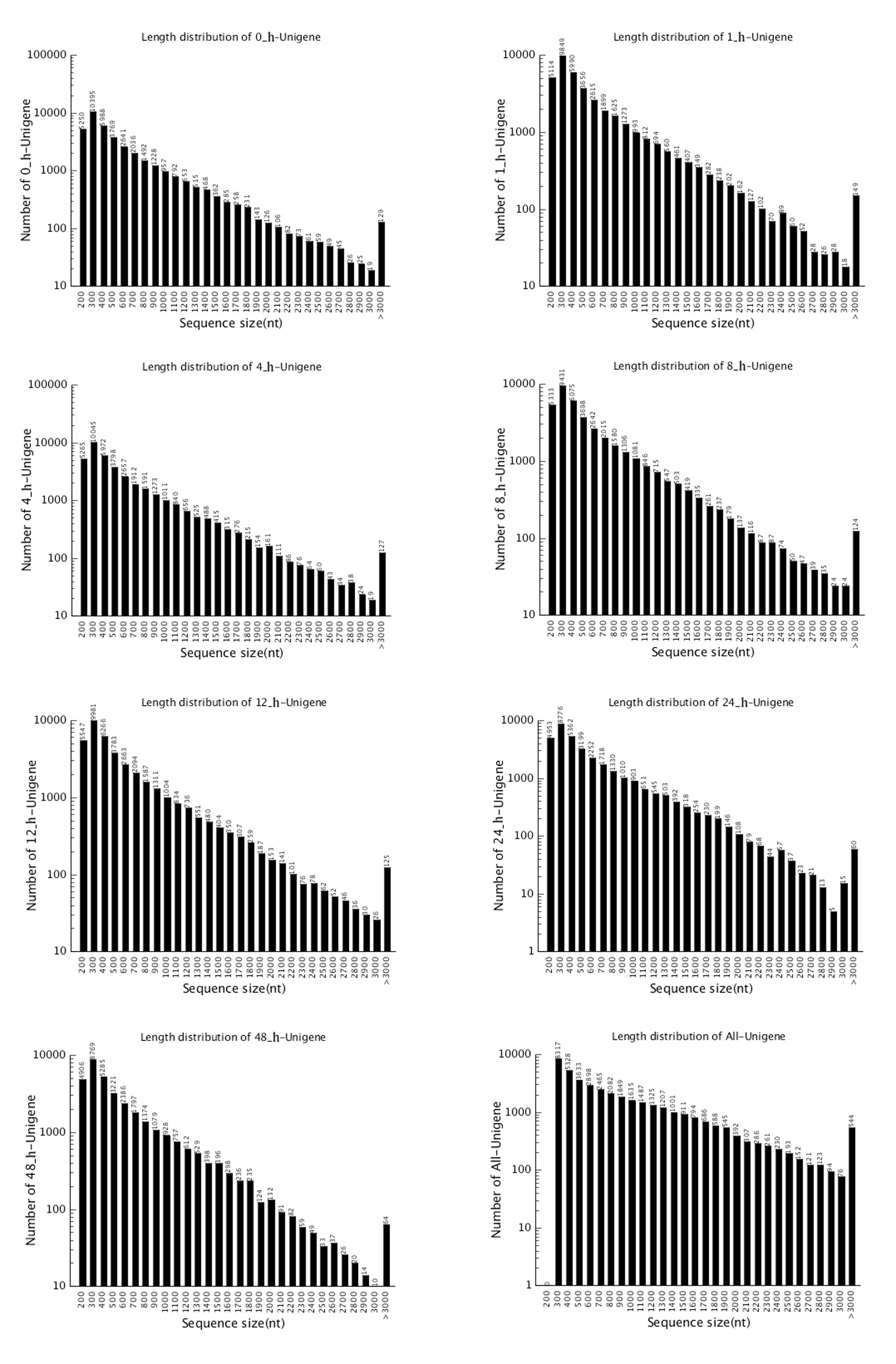

Supplement: Figure S1 — Length distribution of assembled unigenes. 0 h, the non-inoculated control; 1 h, 4 h, 8 h,12 h, 24 h and 48 h indicate the plant tissues of 1, 4, 8, 12, 24 and 48 hour DBM feeding; All-unigene, the unigenes assembled use the mix of the 7 sequence library. (TIF) [file pone.0064481.s001.tif]

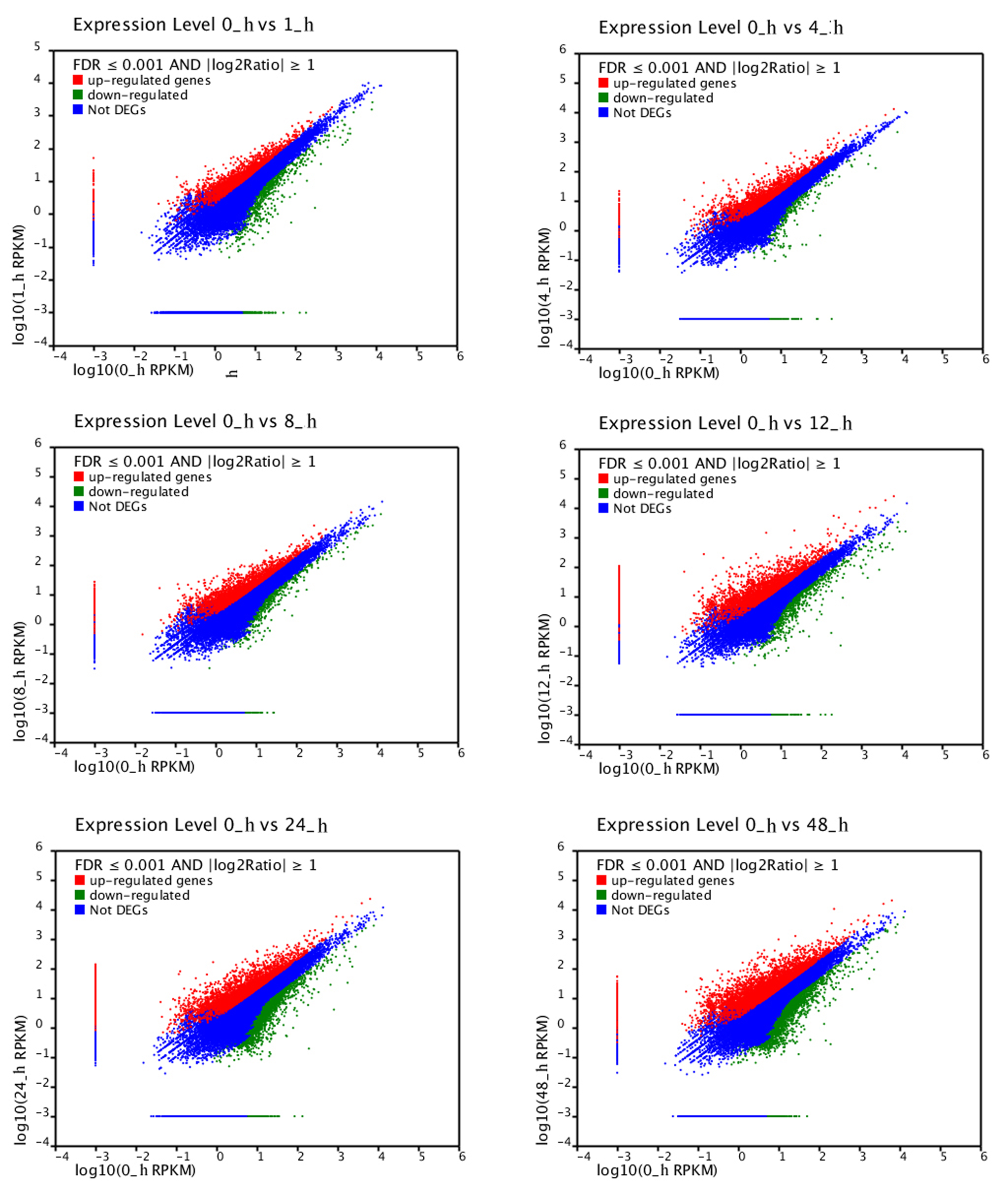

Supplement: Figure S2 — Heat map of relative expression level between plants inoculated with DBM and non-inoculated control. 0 h, the non-inoculated control; 1 h, 4 h, 8 h, 12 h, 24 h and 48 h indicate the plant tissues of 1, 4, 8, 12, 24 and 48 hour DBM feeding. (TIF) [file pone.0064481.s002.tif]
