# Supplementary material for: Transcriptome Analysis of Barbarea vulgaris Infested with Diamondback Moth (Plutella xylostella) Larvae
Source: PLoS One. 2013 May 16;8(5):e64481. doi: 10.1371/journal.pone.0064481 (PMC3655962; doi:10.1371/journal.pone.0064481)
Supplement: Table S3 — The KEGG pathways. (PDF) [file pone.0064481.s005.pdf]

1. All-Unigene.fa

#	Pathway	Count (19620)	Pathway ID
1	Metabolic pathways	4099	ko01100
2	Biosynthesis of secondary metabolites	1916	ko01110
3	Plant-pathogen interaction	1390	ko04626
4	Plant hormone signal transduction	1295	ko04075
5	RNA transport	824	ko03013
6	Spliceosome	817	ko03040
7	Endocytosis	581	ko04144
8	Protein processing in endoplasmic reticulum	560	ko04141
9	Ribosome	544	ko03010
10	Glycerophospholipid metabolism	501	ko00564
11	Ribosome biogenesis in eukaryotes	479	ko03008
12	Starch and sucrose metabolism	423	ko00500
13	Purine metabolism	421	ko00230
14	mRNA surveillance pathway	394	ko03015
15	Ubiquitin mediated proteolysis	379	ko04120
16	Ether lipid metabolism	365	ko00565
17	RNA degradation	360	ko03018
18	Phenylpropanoid biosynthesis	319	ko00940
19	Pyrimidine metabolism	297	ko00240
20	Oxidative phosphorylation	278	ko00190
21	Amino sugar and nucleotide sugar metabolism	259	ko00520
22	Phagosome	252	ko04145
23	Circadian rhythm - plant	235	ko04712
24	Stilbenoid, diarylheptanoid and gingerol biosynthesis	227	ko00945
25	Glycolysis / Gluconeogenesis	224	ko00010
26	Nucleotide excision repair	207	ko03420
27	Limonene and pinene degradation	203	ko00903
28	Peroxisome	192	ko04146
29	Flavonoid biosynthesis	189	ko00941
30	Basal transcription factors	185	ko03022
31	Cysteine and methionine metabolism	180	ko00270
32	Phosphatidylinositol signaling system	175	ko04070
33	Pyruvate metabolism	162	ko00620
34	Homologous recombination	160	ko03440
35	Glutathione metabolism	159	ko00480
36	Inositol phosphate metabolism	153	ko00562
37	Cyanoamino acid metabolism	153	ko00460
38	Aminoacyl-tRNA biosynthesis	151	ko00970
39	ABC transporters	148	ko02010
40	Pentose and glucuronate interconversions	142	ko00040
41	Arginine and proline metabolism	140	ko00330
42	Carotenoid biosynthesis	136	ko00906
43	RNA polymerase	135	ko03020
44	DNA replication	132	ko03030
45	Zeatin biosynthesis	127	ko00908

	Tryptophan metabolism	125	ko00380
47	Regulation of autophagy	124	ko04140
48	Porphyrin and chlorophyll metabolism	123	ko00860
49	Proteasome	122	ko03050
50	Carbon fixation in photosynthetic organisms	122	ko00710
51	Galactose metabolism	120	ko00052
52	Base excision repair	120	ko03410
53	Citrate cycle (TCA cycle)	119	ko00020
54	alpha-Linolenic acid metabolism	118	ko00592
55	Phenylalanine metabolism	118	ko00360
56	Glycerolipid metabolism	116	ko00561
57	Glycine, serine and threonine metabolism	113	ko00260
58	Pentose phosphate pathway	111	ko00030
59	Ascorbate and aldarate metabolism	107	ko00053
60	Nitrogen metabolism	104	ko00910
61	N-Glycan biosynthesis	102	ko00510
62	Photosynthesis	99	ko00195
63	Alanine, aspartate and glutamate metabolism	99	ko00250
64	Protein export	98	ko03060
65	Fatty acid metabolism	98	ko00071
66	Natural killer cell mediated cytotoxicity	95	ko04650
67	Fructose and mannose metabolism	95	ko00051
68	Mismatch repair	92	ko03430
69	Biosynthesis of unsaturated fatty acids	89	ko01040
70	Phenylalanine, tyrosine and tryptophan biosynthesis	86	ko00400
71	SNARE interactions in vesicular transport	85	ko04130
72	Valine, leucine and isoleucine degradation	83	ko00280
73	Sphingolipid metabolism	82	ko00600
74	Steroid biosynthesis	79	ko00100
75	Tyrosine metabolism	79	ko00350
76	Ubiquinone and other terpenoid-quinone biosynthesis	78	ko00130
77	Propanoate metabolism	77	ko00640
78	Terpenoid backbone biosynthesis	76	ko00900
79	Valine, leucine and isoleucine biosynthesis	72	ko00290
80	beta-Alanine metabolism	72	ko00410
81	Glyoxylate and dicarboxylate metabolism	69	ko00630
82	Lysine degradation	69	ko00310
83	Sulfur metabolism	69	ko00920
84	Glycosylphosphatidylinositol(GPI)-anchor biosynthesis	68	ko00563
85	Tropane, piperidine and pyridine alkaloid biosynthesis	60	ko00960
86	Fatty acid biosynthesis	56	ko00061
87	Butanoate metabolism	56	ko00650
88	Flavone and flavonol biosynthesis	55	ko00944
89	Other glycan degradation	52	ko00511
90	Glucosinolate biosynthesis	47	ko00966
91	Glycosaminoglycan degradation	46	ko00531
92	Lysine biosynthesis	46	ko00300

	Histidine metabolism	45	ko00340
94	Pantothenate and CoA biosynthesis	44	ko00770
95	Riboflavin metabolism	44	ko00740
96	Non-homologous end-joining	41	ko03450
97	Benzoxazinoid biosynthesis	37	ko00402
98	Indole alkaloid biosynthesis	37	ko00901
99	Circadian rhythm - mammal	35	ko04710
100	Isoquinoline alkaloid biosynthesis	35	ko00950
101	Photosynthesis - antenna proteins	34	ko00196
102	One carbon pool by folate	34	ko00670
103	Glycosphingolipid biosynthesis - ganglio series	33	ko00604
104	Folate biosynthesis	33	ko00790
105	Diterpenoid biosynthesis	33	ko00904
106	Nicotinate and nicotinamide metabolism	31	ko00760
107	Selenocompound metabolism	30	ko00450
108	Vitamin B6 metabolism	30	ko00750
109	Glycosphingolipid biosynthesis - globo series	25	ko00603
110	Linoleic acid metabolism	24	ko00591
111	Other types of O-glycan biosynthesis	21	ko00514
112	Brassinosteroid biosynthesis	19	ko00905
113	Arachidonic acid metabolism	18	ko00590
114	Sulfur relay system	18	ko04122
115	Fatty acid elongation	17	ko00062
116	Taurine and hypotaurine metabolism	16	ko00430
117	Thiamine metabolism	13	ko00730
118	C5-Branched dibasic acid metabolism	13	ko00660
119	Caffeine metabolism	13	ko00232
120	Monoterpenoid biosynthesis	10	ko00902
121	Anthocyanin biosynthesis	9	ko00942
122	Synthesis and degradation of ketone bodies	8	ko00072
123	Biotin metabolism	7	ko00780
124	Lipoic acid metabolism	5	ko00785
125	Glycosphingolipid biosynthesis - lacto and neolacto series	1	ko00601
126	Sesquiterpenoid biosynthesis	1	ko00909

#	Pathway	Differentially expressed genes
		CL1.Contig101_All, CL1.Contig102_All, CL1.Contig110_All, CL1.Contig120_All, CL1.Contig123_All, CL1.Contig128_All, CL1.Contig12_All, CL1.Contig130_All, CL1.Contig23_All, CL1.Contig39_All, CL1.Contig45_All, CL1.Contig46_All, CL1.Contig57_All, CL1.Contig59_All, CL1.Contig63_All, CL1.Contig81_All, CL1.Contig91_All, CL1.Contig94_All, CL10.Contig14_All, CL10.Contig30_All, CL10004.Contig1_All, CL10008.Contig1_All, CL10020.Contig1_All, CL10026.Contig1_All, CL10038.Contig1_All, CL10048.Contig1_All, CL10055.Contig1_All, CL10057.Contig1_All, CL10058.Contig1_All,

1			CL10068.Contig1_All, CL10072.Contig2_All, CL10073.Contig1_All, CL1009.Contig1_All, CL10104.Contig1_All, CL10108.Contig1_All, CL10109.Contig1_All, CL10115.Contig1_All, CL10128.Contig1_All, CL10132.Contig1_All, CL10134.Contig1_All, CL10136.Contig1_All, CL10145.Contig1_All, CL10146.Contig1_All, CL10151.Contig1_All, CL10155.Contig1_All, CL10161.Contig1_All, CL10162.Contig1_All, CL10163.Contig1_All, CL10165.Contig1_All, CL10171.Contig1_All, CL10183.Contig1_All, CL10189.Contig1_All, CL10198.Contig2_All, CL102.Contig10_All, CL102.Contig1_All, CL102.Contig2_All, CL102.Contig3_All, CL102.Contig4_All, CL102.Contig5_All, CL102.Contig6_All, CL102.Contig7_All, CL102.Contig8_All, CL102.Contig9_All, CL10200.Contig1_All, CL10202.Contig1_All, CL10211.Contig1_All, CL10214.Contig1_All, CL10215.Contig1_All, CL10216.Contig1_All, CL10217.Contig1_All, CL10224.Contig1_All, CL10230.Contig1_All, CL10235.Contig1_All, CL10238.Contig1_All, CL10240.Contig1_All, CL10242.Contig1_All, CL10251.Contig1_All, CL10266.Contig1_All, CL10278.Contig1_All, CL10280.Contig1_All, CL1029.Contig2_All, CL10292.Contig1_All, CL10305.Contig1_All, CL10306.Contig1_All, CL10318.Contig1_All, CL10319.Contig1_All, CL10321.Contig2_All, CL10322.Contig1_All, CL10347.Contig1_All, CL10353.Contig1_All, CL10355.Contig1_All, CL10360.Contig1_All, CL10382.Contig1_All, CL10384.Contig1_All, CL10394.Contig1_All, CL10397.Contig1_All, CL10403.Contig1_All, CL10406.Contig1_All, CL10408.Contig1_All, CL10414.Contig1_All, CL10416.Contig1_All, CL10416.Contig2_All, CL10416.Contig3_All, CL10417.Contig1_All, CL1042.Contig1_All, CL10426.Contig1_All, CL10433.Contig1_All, CL10444.Contig1_All, CL10445.Contig1_All, CL10449.Contig1_All, CL10452.Contig1_All, CL10458.Contig1_All, CL10460.Contig1_All, CL10469.Contig1_All, CL10472.Contig1_All, CL10477.Contig1_All, CL1048.Contig1_All, CL10481.Contig1_All, CL10483.Contig1_All, CL10490.Contig1_All, CL10495.Contig1_All, CL10496.Contig1_All, CL10497.Contig1_All, CL10504.Contig1_All, CL10505.Contig1_All, CL10507.Contig1_All, CL1051.Contig1_All, CL1051.Contig2_All, CL10515.Contig1_All, CL10526.Contig1_All, CL10532.Contig1_All, CL10534.Contig1_All, CL10535.Contig1_All, CL1054.Contig1_All, CL1054.Contig2_All, CL1054.Contig3_All, CL10541.Contig1_All, CL10551.Contig1_All, CL10556.Contig1_All, CL10557.Contig1_All,

	Metabolic pathways (no map	CL10565.Contig1_All, CL10565.Contig2_All, CL10568.Contig1_All, CL10569.Contig1_All, CL10577.Contig1_All, CL10582.Contig1_All, CL10583.Contig1_All, CL10585.Contig1_All, CL10587.Contig1_All, CL1059.Contig1_All, CL1059.Contig2_All, CL10592.Contig1_All, CL106.Contig1_All, CL106.Contig2_All, CL106.Contig3_All, CL106.Contig4_All, CL106.Contig5_All, CL106.Contig6_All, CL10600.Contig1_All, CL10621.Contig1_All, CL10623.Contig1_All, CL10624.Contig1_All, CL10629.Contig1_All, CL10630.Contig1_All, CL10632.Contig1_All, CL10637.Contig1_All, CL10638.Contig1_All, CL10651.Contig1_All, CL10652.Contig1_All, CL10654.Contig1_All, CL10655.Contig1_All, CL10667.Contig1_All, CL10669.Contig1_All, CL10671.Contig1_All, CL10672.Contig1_All, CL10675.Contig1_All, CL10678.Contig1_All, CL10685.Contig1_All, CL10686.Contig1_All, CL10687.Contig1_All, CL1069.Contig1_All, CL10690.Contig1_All, CL10691.Contig1_All, CL10694.Contig1_All, CL10716.Contig1_All, CL10727.Contig1_All, CL10728.Contig1_All, CL10729.Contig1_All, CL10736.Contig1_All, CL1074.Contig1_All, CL1074.Contig2_All, CL10740.Contig1_All, CL10743.Contig1_All, CL10746.Contig1_All, CL10749.Contig1_All, CL10753.Contig1_All, CL1076.Contig1_All, CL1076.Contig2_All, CL1076.Contig3_All, CL1076.Contig4_All, CL1077.Contig1_All, CL10780.Contig1_All, CL10788.Contig1_All, CL108.Contig2_All, CL10814.Contig1_All, CL10816.Contig1_All, CL10825.Contig1_All, CL10829.Contig1_All, CL10834.Contig1_All, CL1084.Contig1_All, CL1084.Contig2_All, CL1084.Contig3_All, CL1084.Contig4_All, CL1084.Contig5_All, CL10840.Contig1_All, CL10841.Contig1_All, CL10846.Contig1_All, CL10846.Contig2_All, CL1086.Contig1_All, CL1086.Contig2_All, CL10864.Contig1_All, CL10865.Contig1_All, CL10870.Contig1_All, CL10871.Contig1_All, CL10875.Contig1_All, CL10877.Contig1_All, CL10881.Contig1_All, CL10883.Contig1_All, CL10886.Contig1_All, CL1089.Contig1_All, CL10901.Contig1_All, CL10902.Contig1_All, CL10924.Contig1_All, CL10932.Contig1_All, CL10943.Contig1_All, CL10964.Contig1_All, CL10971.Contig1_All, CL10992.Contig1_All, CL10994.Contig1_All, CL11.Contig17_All, CL11013.Contig1_All, CL11017.Contig1_All, CL11021.Contig1_All, CL11022.Contig1_All, CL11023.Contig1_All, CL11032.Contig1_All, CL11033.Contig1_All, CL11036.Contig1_All, CL11041.Contig1_All, CL11047.Contig1_All,

	in kegg database)	CL11064.Contig1_All, CL11067.Contig1_All, CL11070.Contig1_All, CL11072.Contig1_All, CL11080.Contig1_All, CL11090.Contig1_All, CL11091.Contig1_All, CL11092.Contig1_All, CL11093.Contig1_All, CL11094.Contig1_All, CL11099.Contig1_All, CL1111.Contig1_All, CL1111.Contig2_All, CL11110.Contig1_All, CL11111.Contig1_All, CL11116.Contig1_All, CL11117.Contig1_All, CL11129.Contig1_All, CL11130.Contig1_All, CL11135.Contig1_All, CL11137.Contig1_All, CL11144.Contig1_All, CL11145.Contig1_All, CL1115.Contig1_All, CL1115.Contig2_All, CL11160.Contig1_All, CL11163.Contig1_All, CL11178.Contig1_All, CL11203.Contig1_All, CL11212.Contig1_All, CL11213.Contig1_All, CL11224.Contig1_All, CL11229.Contig1_All, CL11236.Contig1_All, CL11247.Contig1_All, CL11256.Contig1_All, CL11261.Contig1_All, CL11262.Contig1_All, CL11271.Contig1_All, CL11277.Contig1_All, CL11279.Contig1_All, CL11284.Contig1_All, CL11286.Contig1_All, CL11287.Contig1_All, CL11296.Contig1_All, CL11320.Contig1_All, CL11324.Contig1_All, CL11330.Contig1_All, CL11342.Contig1_All, CL11348.Contig1_All, CL11370.Contig1_All, CL11391.Contig1_All, CL11392.Contig1_All, CL11396.Contig1_All, CL11401.Contig1_All, CL11408.Contig1_All, CL11416.Contig1_All, CL11423.Contig1_All, CL11433.Contig1_All, CL11461.Contig1_All, CL11464.Contig1_All, CL11465.Contig1_All, CL11468.Contig1_All, CL11474.Contig1_All, CL11477.Contig1_All, CL11480.Contig1_All, CL11484.Contig1_All, CL11490.Contig1_All, CL1150.Contig1_All, CL11513.Contig1_All, CL11520.Contig1_All, CL11523.Contig1_All, CL11525.Contig1_All, CL11527.Contig1_All, CL11535.Contig1_All, CL11542.Contig1_All, CL11555.Contig1_All, CL11562.Contig1_All, CL11570.Contig1_All, CL11575.Contig1_All, CL11580.Contig1_All, CL11594.Contig1_All, CL11595.Contig1_All, CL11606.Contig1_All, CL11613.Contig1_All, CL11616.Contig1_All, CL11617.Contig1_All, CL1162.Contig1_All, CL11625.Contig1_All, CL11629.Contig1_All, CL11632.Contig1_All, CL11635.Contig1_All, CL11649.Contig1_All, CL1165.Contig1_All, CL1165.Contig2_All, CL1165.Contig3_All, CL1165.Contig4_All, CL11653.Contig1_All, CL11655.Contig1_All, CL1166.Contig1_All, CL1166.Contig2_All, CL1166.Contig3_All, CL11669.Contig1_All, CL11672.Contig1_All, CL11695.Contig1_All, CL11699.Contig1_All, CL11710.Contig1_All, CL11719.Contig1_All,

		CL1172.Contig1_All, CL1172.Contig2_All, CL11729.Contig1_All, CL1173.Contig1_All, CL11734.Contig1_All, CL11738.Contig1_All, CL11741.Contig1_All, CL1175.Contig1_All, CL11754.Contig1_All, CL11759.Contig1_All, CL11762.Contig1_All, CL1177.Contig1_All, CL11779.Contig1_All, CL118.Contig10_All, CL118.Contig12_All, CL118.Contig13_All, CL118.Contig14_All, CL118.Contig4_All, CL118.Contig5_All, CL1180.Contig1_All, CL1180.Contig2_All, CL1180.Contig3_All, CL11811.Contig1_All, CL11812.Contig1_All, CL11816.Contig1_All, CL11819.Contig1_All, CL11827.Contig1_All, CL11828.Contig1_All, CL11835.Contig1_All, CL11840.Contig1_All, CL11843.Contig1_All, CL11845.Contig1_All, CL11866.Contig1_All, CL11877.Contig1_All, CL11878.Contig1_All, CL11881.Contig1_All, CL11883.Contig1_All, CL11909.Contig1_All, CL11912.Contig1_All, CL11926.Contig1_All, CL11940.Contig1_All, CL11944.Contig1_All, CL11952.Contig1_All, CL11954.Contig1_All, CL1196.Contig4_All, CL11964.Contig1_All, CL11966.Contig1_All, CL1197.Contig1_All, CL1197.Contig2_All, CL11970.Contig1_All, CL11971.Contig1_All, CL11973.Contig1_All, CL11980.Contig1_All, CL11985.Contig1_All, CL11993.Contig1_All, CL11994.Contig1_All, CL12028.Contig1_All, CL12029.Contig1_All, CL12037.Contig1_All, CL12039.Contig1_All, CL12039.Contig2_All, CL12055.Contig1_All, CL12059.Contig1_All, CL12067.Contig1_All, CL1207.Contig1_All, CL1207.Contig2_All, CL1207.Contig3_All, CL1207.Contig4_All, CL12074.Contig1_All, CL12075.Contig1_All, CL12077.Contig1_All, CL12091.Contig1_All, CL12095.Contig1_All, CL12097.Contig1_All, CL12102.Contig1_All, CL12104.Contig1_All, CL12107.Contig1_All, CL12112.Contig1_All, CL12124.Contig1_All, CL12126.Contig1_All, CL12133.Contig1_All, CL12145.Contig1_All, CL12156.Contig1_All, CL12159.Contig1_All, CL12174.Contig1_All, CL12183.Contig1_All, CL12184.Contig1_All, CL12185.Contig1_All, CL12194.Contig1_All, CL12202.Contig1_All, CL12207.Contig1_All, CL12208.Contig1_All, CL12210.Contig1_All, CL12211.Contig1_All, CL12222.Contig1_All, CL12226.Contig1_All, CL12232.Contig1_All, CL12238.Contig1_All, CL12247.Contig1_All, CL12260.Contig1_All, CL1227.Contig1_All, CL12270.Contig1_All, CL12277.Contig1_All, CL1228.Contig1_All, CL12281.Contig1_All, CL12283.Contig1_All, CL12293.Contig1_All, CL12294.Contig1_All, CL12297.Contig1_All,

			CL12301.Contig1_All, CL12307.Contig1_All, CL1231.Contig3_All, CL12316.Contig1_All, CL12317.Contig1_All, CL12322.Contig1_All, CL12334.Contig1_All, CL1234.Contig1_All, CL1234.Contig2_All, CL12340.Contig1_All, CL12342.Contig1_All, CL12346.Contig1_All, CL12354.Contig1_All, CL12364.Contig1_All, CL12367.Contig1_All, CL12369.Contig1_All, CL12373.Contig1_All, CL12376.Contig1_All, CL12385.Contig1_All, CL12389.Contig1_All, CL12396.Contig1_All, CL12410.Contig1_All, CL12413.Contig1_All, CL12418.Contig1_All, CL12419.Contig1_All, CL12424.Contig1_All, CL12429.Contig1_All, CL12430.Contig1_All, CL12447.Contig1_All, CL1246.Contig1_All, CL12463.Contig1_All, CL12466.Contig1_All, CL12470.Contig1_All, CL12487.Contig1_All, CL12496.Contig1_All, CL12497.Contig1_All, CL12498.Contig1_All, CL12514.Contig1_All, CL12520.Contig1_All, CL12523.Contig1_All, CL12526.Contig1_All, CL12530.Contig1_All, CL12532.Contig1_All, CL12555.Contig1_All, CL12556.Contig1_All, CL1256.Contig1_All, CL1256.Contig2_All, CL12575.Contig1_All, CL12597.Contig1_All, CL12607.Contig1_All, CL12611.Contig1_All, CL12612.Contig1_All, CL12629.Contig1_All, CL12640.Contig1_All, CL12644.Contig1_All, CL12645.Contig1_All, CL12667.Contig1_All, CL12668.Contig1_All, CL12674.Contig1_All, CL12687.Contig1_All, CL1269.Contig1_All, CL1269.Contig3_All, CL1269.Contig4_All, CL1269.Contig5_All, CL12692.Contig1_All, CL12698.Contig1_All, CL12705.Contig1_All, CL12715.Contig1_All, CL1272.Contig1_All, CL1272.Contig2_All, CL1272.Contig3_All, CL12720.Contig1_All, CL12722.Contig1_All, CL12743.Contig1_All, CL12748.Contig1_All, CL12751.Contig1_All, CL12753.Contig1_All, CL12762.Contig1_All, CL1277.Contig2_All, CL12777.Contig1_All, CL12780.Contig1_All, CL12810.Contig1_All, CL12816.Contig1_All, CL12818.Contig1_All, CL1282.Contig1_All, CL12820.Contig1_All, CL12822.Contig1_All, CL12848.Contig1_All, CL12850.Contig1_All, CL12852.Contig1_All, CL12857.Contig1_All, CL12865.Contig1_All, CL12868.Contig1_All, CL12869.Contig1_All, CL12875.Contig1_All, CL1288.Contig1_All, CL1288.Contig2_All, CL1288.Contig3_All, CL1288.Contig4_All, CL12888.Contig1_All, CL12898.Contig1_All, CL12906.Contig1_All, CL12909.Contig1_All, CL12911.Contig1_All, CL12912.Contig1_All, CL12917.Contig1_All, CL12925.Contig1_All, CL12927.Contig1_All,

		CL12927.Contig2_All, CL12928.Contig1_All, CL12937.Contig1_All, CL1294.Contig1_All, CL1294.Contig2_All, CL1294.Contig3_All, CL1294.Contig4_All, CL12942.Contig1_All, CL12944.Contig1_All, CL12945.Contig1_All, CL12945.Contig2_All, CL12951.Contig1_All, CL12957.Contig1_All, CL12981.Contig1_All, CL12985.Contig1_All, CL12993.Contig1_All, CL12996.Contig1_All, CL12996.Contig2_All, CL13001.Contig1_All, CL13016.Contig1_All, CL13018.Contig1_All, CL13019.Contig1_All, CL13023.Contig1_All, CL13024.Contig1_All, CL13025.Contig1_All, CL13034.Contig1_All, CL1304.Contig1_All, CL1304.Contig2_All, CL1304.Contig3_All, CL13046.Contig1_All, CL13060.Contig1_All, CL13068.Contig1_All, CL13071.Contig1_All, CL13073.Contig1_All, CL13080.Contig1_All, CL13083.Contig1_All, CL13084.Contig1_All, CL13092.Contig1_All, CL131.Contig1_All, CL131.Contig2_All, CL131.Contig3_All, CL131.Contig4_All, CL131.Contig5_All, CL131.Contig6_All, CL131.Contig7_All, CL13104.Contig1_All, CL1312.Contig1_All, CL13139.Contig1_All, CL13152.Contig1_All, CL13162.Contig1_All, CL13169.Contig1_All, CL13172.Contig1_All, CL13175.Contig1_All, CL13176.Contig1_All, CL13179.Contig1_All, CL13220.Contig1_All, CL13222.Contig1_All, CL13232.Contig1_All, CL13244.Contig1_All, CL13246.Contig1_All, CL13253.Contig1_All, CL13256.Contig1_All, CL1326.Contig1_All, CL1326.Contig2_All, CL13269.Contig1_All, CL13270.Contig1_All, CL13272.Contig1_All, CL13275.Contig1_All, CL13277.Contig1_All, CL1328.Contig1_All, CL13286.Contig1_All, CL13291.Contig1_All, CL13297.Contig2_All, CL13321.Contig1_All, CL13326.Contig1_All, CL13338.Contig1_All, CL13341.Contig1_All, CL13347.Contig1_All, CL13356.Contig1_All, CL13357.Contig1_All, CL13381.Contig1_All, CL13384.Contig1_All, CL13384.Contig2_All, CL13385.Contig1_All, CL1339.Contig1_All, CL1339.Contig2_All, CL1339.Contig3_All, CL1339.Contig4_All, CL13408.Contig1_All, CL1342.Contig1_All, CL1342.Contig2_All, CL1342.Contig3_All, CL1342.Contig4_All, CL13436.Contig1_All, CL13440.Contig1_All, CL13444.Contig1_All, CL13445.Contig1_All, CL13451.Contig1_All, CL1346.Contig1_All, CL1346.Contig2_All, CL1346.Contig3_All, CL1346.Contig4_All, CL13475.Contig1_All, CL13486.Contig1_All, CL13489.Contig1_All, CL13490.Contig1_All, CL13492.Contig1_All, CL13506.Contig1_All, CL13520.Contig1_All, CL13523.Contig1_All,

		CL13524.Contig1_All, CL13525.Contig1_All, CL13527.Contig1_All, CL13531.Contig1_All, CL13535.Contig1_All, CL13538.Contig1_All, CL13540.Contig1_All, CL13548.Contig1_All, CL13549.Contig1_All, CL13560.Contig1_All, CL13569.Contig1_All, CL13571.Contig1_All, CL13574.Contig1_All, CL13575.Contig1_All, CL13586.Contig1_All, CL13590.Contig1_All, CL13593.Contig1_All, CL13594.Contig1_All, CL13600.Contig1_All, CL13604.Contig1_All, CL13630.Contig1_All, CL13634.Contig1_All, CL13635.Contig1_All, CL13661.Contig1_All, CL13666.Contig1_All, CL1369.Contig1_All, CL1369.Contig2_All, CL1369.Contig3_All, CL13712.Contig1_All, CL13714.Contig1_All, CL13717.Contig1_All, CL13717.Contig2_All, CL13721.Contig1_All, CL13729.Contig1_All, CL13756.Contig1_All, CL13770.Contig1_All, CL13772.Contig1_All, CL13776.Contig1_All, CL13779.Contig1_All, CL1378.Contig1_All, CL1378.Contig2_All, CL13789.Contig1_All, CL13814.Contig1_All, CL13817.Contig1_All, CL13818.Contig1_All, CL13819.Contig1_All, CL13835.Contig1_All, CL1384.Contig1_All, CL13859.Contig1_All, CL13877.Contig1_All, CL13884.Contig1_All, CL13889.Contig1_All, CL13895.Contig1_All, CL1390.Contig1_All, CL13905.Contig1_All, CL13923.Contig1_All, CL13925.Contig1_All, CL13926.Contig1_All, CL13928.Contig1_All, CL13936.Contig1_All, CL13956.Contig1_All, CL13964.Contig1_All, CL13975.Contig1_All, CL13975.Contig2_All, CL13980.Contig1_All, CL13995.Contig1_All, CL13999.Contig1_All, CL13999.Contig2_All, CL14003.Contig1_All, CL14008.Contig1_All, CL14024.Contig1_All, CL14027.Contig1_All, CL14038.Contig1_All, CL14045.Contig1_All, CL14053.Contig1_All, CL1406.Contig2_All, CL1406.Contig3_All, CL14060.Contig1_All, CL1407.Contig1_All, CL1407.Contig2_All, CL1407.Contig3_All, CL14074.Contig1_All, CL1408.Contig1_All, CL1408.Contig2_All, CL1408.Contig3_All, CL1408.Contig4_All, CL14083.Contig1_All, CL14098.Contig1_All, CL14112.Contig1_All, CL14117.Contig1_All, CL14119.Contig1_All, CL1412.Contig1_All, CL1412.Contig2_All, CL14122.Contig1_All, CL14122.Contig2_All, CL14136.Contig1_All, CL14140.Contig1_All, CL14141.Contig1_All, CL14164.Contig1_All, CL14169.Contig1_All, CL14182.Contig1_All, CL14213.Contig1_All, CL14225.Contig1_All, CL14237.Contig1_All, CL1424.Contig1_All, CL14252.Contig1_All, CL1426.Contig1_All, CL14261.Contig1_All,

			CL14267.Contig1_All, CL14269.Contig1_All, CL1427.Contig1_All, CL1427.Contig3_All, CL1427.Contig4_All, CL1427.Contig5_All, CL14283.Contig1_All, CL14293.Contig1_All, CL14294.Contig1_All, CL143.Contig1_All, CL143.Contig4_All, CL14304.Contig1_All, CL14310.Contig1_All, CL14311.Contig1_All, CL14313.Contig1_All, CL14314.Contig1_All, CL14326.Contig1_All, CL14352.Contig1_All, CL14365.Contig1_All, CL14367.Contig1_All, CL14375.Contig1_All, CL14379.Contig1_All, CL14385.Contig1_All, CL14388.Contig1_All, CL14392.Contig1_All, CL14394.Contig1_All, CL14400.Contig1_All, CL14404.Contig1_All, CL14416.Contig1_All, CL14422.Contig1_All, CL14423.Contig1_All, CL14439.Contig1_All, CL1445.Contig3_All, CL1445.Contig6_All, CL14468.Contig1_All, CL14470.Contig1_All, CL14476.Contig1_All, CL14476.Contig2_All, CL14487.Contig1_All, CL14498.Contig1_All, CL14503.Contig1_All, CL14505.Contig1_All, CL14510.Contig1_All, CL14510.Contig2_All, CL14543.Contig1_All, CL1455.Contig1_All, CL1455.Contig2_All, CL1455.Contig3_All, CL14555.Contig1_All, CL14558.Contig1_All, CL14560.Contig1_All, CL14573.Contig1_All, CL14574.Contig1_All, CL14588.Contig1_All, CL14590.Contig1_All, CL14592.Contig1_All, CL1460.Contig2_All, CL14605.Contig1_All, CL14659.Contig1_All, CL14669.Contig1_All, CL14671.Contig1_All, CL14671.Contig2_All, CL14672.Contig1_All, CL14677.Contig1_All, CL1469.Contig3_All, CL147.Contig1_All, CL14726.Contig1_All, CL14728.Contig1_All, CL14732.Contig1_All, CL14738.Contig1_All, CL14751.Contig1_All, CL14752.Contig1_All, CL14753.Contig1_All, CL14758.Contig1_All, CL14764.Contig1_All, CL1477.Contig1_All, CL1477.Contig2_All, CL1477.Contig3_All, CL14772.Contig1_All, CL14774.Contig1_All, CL14782.Contig1_All, CL1479.Contig1_All, CL1479.Contig2_All, CL1479.Contig3_All, CL1479.Contig4_All, CL1479.Contig5_All, CL14795.Contig1_All, CL14802.Contig1_All, CL14818.Contig1_All, CL14824.Contig1_All, CL14842.Contig1_All, CL14853.Contig1_All, CL14855.Contig1_All, CL14866.Contig1_All, CL14874.Contig1_All, CL14877.Contig1_All, CL14883.Contig1_All, CL14892.Contig1_All, CL14897.Contig1_All, CL14922.Contig1_All, CL14927.Contig1_All, CL14931.Contig1_All, CL14933.Contig1_All, CL14937.Contig1_All, CL1494.Contig1_All, CL1494.Contig2_All, CL1494.Contig3_All, CL1494.Contig4_All, CL14954.Contig1_All,

		CL1496.Contig1_All, CL14966.Contig1_All, CL14981.Contig1_All, CL14988.Contig1_All, CL150.Contig7_All, CL15015.Contig1_All, CL1502.Contig1_All, CL15029.Contig1_All, CL15035.Contig1_All, CL15039.Contig1_All, CL15079.Contig1_All, CL151.Contig2_All, CL151.Contig4_All, CL15100.Contig1_All, CL15111.Contig1_All, CL15119.Contig1_All, CL15125.Contig1_All, CL15140.Contig1_All, CL15143.Contig1_All, CL15147.Contig1_All, CL15160.Contig1_All, CL15161.Contig1_All, CL15163.Contig1_All, CL15169.Contig1_All, CL15175.Contig1_All, CL15189.Contig1_All, CL15189.Contig2_All, CL15191.Contig1_All, CL15207.Contig1_All, CL15213.Contig1_All, CL1523.Contig1_All, CL15237.Contig1_All, CL15251.Contig1_All, CL15253.Contig1_All, CL1526.Contig1_All, CL1526.Contig2_All, CL15260.Contig1_All, CL1527.Contig1_All, CL1527.Contig2_All, CL1527.Contig3_All, CL15275.Contig1_All, CL15284.Contig1_All, CL1529.Contig1_All, CL1529.Contig2_All, CL1529.Contig3_All, CL1529.Contig4_All, CL1529.Contig5_All, CL15300.Contig1_All, CL15301.Contig1_All, CL15303.Contig1_All, CL1531.Contig1_All, CL15319.Contig1_All, CL15323.Contig1_All, CL15330.Contig1_All, CL15335.Contig1_All, CL15336.Contig1_All, CL15340.Contig1_All, CL15341.Contig1_All, CL15356.Contig1_All, CL15361.Contig1_All, CL15367.Contig1_All, CL15368.Contig1_All, CL15378.Contig1_All, CL15382.Contig1_All, CL15387.Contig1_All, CL1539.Contig1_All, CL1539.Contig2_All, CL15392.Contig1_All, CL15401.Contig1_All, CL15403.Contig1_All, CL15424.Contig1_All, CL15428.Contig1_All, CL15430.Contig1_All, CL15436.Contig1_All, CL1544.Contig1_All, CL15446.Contig1_All, CL15447.Contig1_All, CL15451.Contig1_All, CL15475.Contig1_All, CL1548.Contig2_All, CL15504.Contig1_All, CL15517.Contig1_All, CL15518.Contig1_All, CL1552.Contig1_All, CL15530.Contig1_All, CL15538.Contig1_All, CL15546.Contig1_All, CL15566.Contig1_All, CL1558.Contig1_All, CL1558.Contig2_All, CL1559.Contig1_All, CL1559.Contig2_All, CL15591.Contig1_All, CL156.Contig1_All, CL156.Contig2_All, CL156.Contig3_All, CL156.Contig4_All, CL156.Contig5_All, CL156.Contig6_All, CL156.Contig7_All, CL156.Contig8_All, CL156.Contig9_All, CL15602.Contig1_All, CL1562.Contig1_All, CL1562.Contig2_All, CL1562.Contig3_All, CL1562.Contig4_All, CL15626.Contig1_All, CL15629.Contig1_All, CL15635.Contig1_All, CL15643.Contig1_All, CL15652.Contig1_All,

		CL15672.Contig1_All, CL15676.Contig1_All, CL1568.Contig1_All, CL1568.Contig2_All, CL1568.Contig3_All, CL1568.Contig4_All, CL15686.Contig1_All, CL15697.Contig1_All, CL15700.Contig1_All, CL15700.Contig2_All, CL15733.Contig1_All, CL15754.Contig1_All, CL15778.Contig1_All, CL1578.Contig1_All, CL1578.Contig2_All, CL1578.Contig3_All, CL1578.Contig4_All, CL15790.Contig1_All, CL15799.Contig1_All, CL15809.Contig1_All, CL15813.Contig1_All, CL15822.Contig1_All, CL15849.Contig1_All, CL15857.Contig1_All, CL15867.Contig1_All, CL1587.Contig1_All, CL15878.Contig1_All, CL15902.Contig1_All, CL1591.Contig1_All, CL1591.Contig2_All, CL1591.Contig3_All, CL1591.Contig4_All, CL1591.Contig5_All, CL15947.Contig1_All, CL15961.Contig1_All, CL1599.Contig1_All, CL16000.Contig1_All, CL1603.Contig1_All, CL1603.Contig2_All, CL16037.Contig1_All, CL16038.Contig1_All, CL16043.Contig1_All, CL16051.Contig1_All, CL16054.Contig1_All, CL16056.Contig1_All, CL16059.Contig1_All, CL16061.Contig1_All, CL16080.Contig1_All, CL1609.Contig1_All, CL1609.Contig4_All, CL16094.Contig1_All, CL16109.Contig1_All, CL16124.Contig1_All, CL16128.Contig1_All, CL1615.Contig1_All, CL16157.Contig1_All, CL16172.Contig1_All, CL16173.Contig1_All, CL1618.Contig1_All, CL1618.Contig2_All, CL16186.Contig1_All, CL16187.Contig1_All, CL16203.Contig1_All, CL16204.Contig1_All, CL16214.Contig1_All, CL16225.Contig1_All, CL16225.Contig2_All, CL16225.Contig3_All, CL16239.Contig1_All, CL16268.Contig1_All, CL16275.Contig1_All, CL16277.Contig1_All, CL16277.Contig2_All, CL16291.Contig1_All, CL16293.Contig1_All, CL16302.Contig1_All, CL16319.Contig1_All, CL16330.Contig1_All, CL16345.Contig1_All, CL16358.Contig1_All, CL16372.Contig1_All, CL16385.Contig1_All, CL16388.Contig1_All, CL1642.Contig1_All, CL16428.Contig1_All, CL16430.Contig1_All, CL16449.Contig1_All, CL16457.Contig1_All, CL16462.Contig1_All, CL16464.Contig1_All, CL16473.Contig1_All, CL16492.Contig1_All, CL16502.Contig1_All, CL16515.Contig1_All, CL16534.Contig1_All, CL16540.Contig1_All, CL16543.Contig1_All, CL16555.Contig1_All, CL16556.Contig1_All, CL16561.Contig1_All, CL16566.Contig1_All, CL16586.Contig1_All, CL16589.Contig1_All, CL16603.Contig1_All, CL16605.Contig1_All, CL16638.Contig1_All, CL16640.Contig1_All, CL16643.Contig1_All,

			CL16661.Contig1_All, CL1667.Contig1_All, CL16675.Contig1_All, CL16676.Contig1_All, CL16706.Contig1_All, CL1671.Contig1_All, CL16719.Contig1_All, CL1672.Contig1_All, CL16723.Contig1_All, CL16728.Contig1_All, CL16728.Contig2_All, CL1675.Contig1_All, CL16751.Contig1_All, CL16771.Contig1_All, CL16785.Contig1_All, CL16787.Contig1_All, CL1679.Contig1_All, CL16790.Contig1_All, CL16795.Contig1_All, CL16802.Contig1_All, CL16817.Contig1_All, CL16824.Contig1_All, CL16833.Contig1_All, CL16840.Contig1_All, CL16853.Contig1_All, CL16871.Contig1_All, CL1689.Contig1_All, CL16918.Contig1_All, CL16932.Contig1_All, CL16982.Contig1_All, CL16998.Contig1_All, CL17057.Contig1_All, CL17059.Contig1_All, CL17072.Contig1_All, CL17086.Contig1_All, CL17086.Contig2_All, CL171.Contig6_All, CL17109.Contig1_All, CL1711.Contig2_All, CL17111.Contig1_All, CL17124.Contig1_All, CL17148.Contig1_All, CL17158.Contig1_All, CL17189.Contig1_All, CL17196.Contig1_All, CL17200.Contig1_All, CL17229.Contig1_All, CL17233.Contig1_All, CL17263.Contig1_All, CL17274.Contig1_All, CL17278.Contig1_All, CL17278.Contig2_All, CL1728.Contig1_All, CL1728.Contig2_All, CL17291.Contig1_All, CL17291.Contig2_All, CL17309.Contig1_All, CL17313.Contig1_All, CL17326.Contig1_All, CL17355.Contig1_All, CL17356.Contig1_All, CL17370.Contig1_All, CL17379.Contig1_All, CL1738.Contig1_All, CL17382.Contig1_All, CL17393.Contig1_All, CL17401.Contig1_All, CL17409.Contig1_All, CL17410.Contig1_All, CL17419.Contig1_All, CL17426.Contig1_All, CL17441.Contig1_All, CL17459.Contig1_All, CL17459.Contig2_All, CL17466.Contig1_All, CL17482.Contig1_All, CL17486.Contig1_All, CL1750.Contig1_All, CL17539.Contig1_All, CL17547.Contig1_All, CL17548.Contig1_All, CL17574.Contig1_All, CL17581.Contig1_All, CL17597.Contig1_All, CL17597.Contig2_All, CL17630.Contig1_All, CL17637.Contig1_All, CL17648.Contig1_All, CL1766.Contig1_All, CL1766.Contig2_All, CL1766.Contig3_All, CL17684.Contig1_All, CL17689.Contig1_All, CL17693.Contig1_All, CL17699.Contig1_All, CL17705.Contig1_All, CL17732.Contig1_All, CL17743.Contig1_All, CL1776.Contig1_All, CL1776.Contig2_All, CL1776.Contig3_All, CL1776.Contig4_All, CL1776.Contig5_All, CL1776.Contig6_All, CL17764.Contig1_All, CL17778.Contig1_All, CL1779.Contig3_All, CL17793.Contig1_All,

		CL178.Contig1_All, CL178.Contig2_All, CL178.Contig3_All, CL178.Contig4_All, CL17852.Contig1_All, CL17858.Contig1_All, CL17880.Contig1_All, CL1791.Contig1_All, CL17914.Contig1_All, CL17921.Contig1_All, CL17938.Contig1_All, CL17949.Contig1_All, CL17962.Contig1_All, CL180.Contig2_All, CL180.Contig4_All, CL180.Contig7_All, CL18054.Contig1_All, CL18061.Contig1_All, CL181.Contig1_All, CL181.Contig2_All, CL181.Contig3_All, CL181.Contig5_All, CL18104.Contig1_All, CL18109.Contig1_All, CL18111.Contig1_All, CL18117.Contig1_All, CL18144.Contig1_All, CL18159.Contig1_All, CL18194.Contig1_All, CL18202.Contig1_All, CL18211.Contig1_All, CL18212.Contig1_All, CL18214.Contig1_All, CL18215.Contig1_All, CL18226.Contig1_All, CL18233.Contig1_All, CL18244.Contig1_All, CL18250.Contig1_All, CL18254.Contig1_All, CL18261.Contig1_All, CL18263.Contig1_All, CL18268.Contig1_All, CL18289.Contig1_All, CL1830.Contig1_All, CL18317.Contig1_All, CL18320.Contig1_All, CL18325.Contig1_All, CL18330.Contig1_All, CL1834.Contig3_All, CL18342.Contig1_All, CL18351.Contig1_All, CL18355.Contig1_All, CL18360.Contig1_All, CL18369.Contig1_All, CL18381.Contig1_All, CL18418.Contig1_All, CL18429.Contig1_All, CL18440.Contig1_All, CL18441.Contig1_All, CL18450.Contig1_All, CL18453.Contig1_All, CL1847.Contig1_All, CL18472.Contig1_All, CL18474.Contig1_All, CL18491.Contig1_All, CL18492.Contig1_All, CL18529.Contig1_All, CL18532.Contig1_All, CL18538.Contig1_All, CL18544.Contig1_All, CL1855.Contig3_All, CL1858.Contig1_All, CL18609.Contig1_All, CL18626.Contig1_All, CL1863.Contig2_All, CL1863.Contig3_All, CL1865.Contig1_All, CL18669.Contig1_All, CL1868.Contig1_All, CL1871.Contig1_All, CL18727.Contig1_All, CL18732.Contig1_All, CL1874.Contig1_All, CL1874.Contig2_All, CL1874.Contig3_All, CL1874.Contig4_All, CL18751.Contig1_All, CL18780.Contig1_All, CL18785.Contig1_All, CL18789.Contig1_All, CL18792.Contig1_All, CL188.Contig1_All, CL188.Contig2_All, CL188.Contig3_All, CL18800.Contig1_All, CL1881.Contig1_All, CL1881.Contig2_All, CL1881.Contig3_All, CL18816.Contig1_All, CL1882.Contig1_All, CL1882.Contig2_All, CL1882.Contig3_All, CL1882.Contig4_All, CL1882.Contig5_All, CL1882.Contig6_All, CL18822.Contig1_All, CL18836.Contig1_All, CL18849.Contig1_All, CL18868.Contig1_All, CL18904.Contig1_All, CL18907.Contig1_All, CL1893.Contig1_All,

			CL18933.Contig1_All, CL18979.Contig1_All, CL18986.Contig1_All, CL18999.Contig1_All, CL19.Contig21_All, CL19021.Contig1_All, CL19037.Contig1_All, CL19059.Contig1_All, CL19062.Contig1_All, CL19075.Contig1_All, CL19110.Contig1_All, CL19121.Contig1_All, CL19148.Contig1_All, CL1916.Contig1_All, CL1917.Contig1_All, CL19178.Contig1_All, CL1919.Contig1_All, CL19193.Contig1_All, CL19200.Contig1_All, CL19257.Contig1_All, CL19263.Contig1_All, CL19267.Contig1_All, CL19281.Contig1_All, CL19286.Contig1_All, CL19337.Contig1_All, CL19341.Contig1_All, CL19368.Contig1_All, CL19398.Contig1_All, CL19408.Contig1_All, CL1942.Contig1_All, CL19423.Contig1_All, CL19434.Contig1_All, CL19445.Contig1_All, CL19512.Contig1_All, CL19523.Contig1_All, CL19553.Contig1_All, CL19564.Contig1_All, CL19567.Contig1_All, CL19570.Contig1_All, CL19577.Contig1_All, CL1958.Contig2_All, CL19602.Contig1_All, CL19635.Contig1_All, CL19642.Contig1_All, CL19644.Contig1_All, CL19651.Contig1_All, CL19659.Contig1_All, CL19667.Contig1_All, CL1967.Contig1_All, CL1967.Contig2_All, CL1967.Contig3_All, CL19670.Contig1_All, CL1968.Contig1_All, CL19694.Contig1_All, CL197.Contig1_All, CL197.Contig2_All, CL19701.Contig1_All, CL19710.Contig1_All, CL19717.Contig1_All, CL1976.Contig1_All, CL1977.Contig1_All, CL19779.Contig1_All, CL1978.Contig1_All, CL19784.Contig1_All, CL198.Contig1_All, CL198.Contig2_All, CL198.Contig3_All, CL198.Contig4_All, CL198.Contig5_All, CL198.Contig6_All, CL198.Contig7_All, CL198.Contig8_All, CL198.Contig9_All, CL19812.Contig1_All, CL1983.Contig1_All, CL19831.Contig1_All, CL19848.Contig1_All, CL19855.Contig1_All, CL19884.Contig1_All, CL1989.Contig2_All, CL19904.Contig1_All, CL19934.Contig1_All, CL1995.Contig1_All, CL1995.Contig2_All, CL1995.Contig3_All, CL1997.Contig1_All, CL1997.Contig2_All, CL1997.Contig3_All, CL1999.Contig1_All, CL1999.Contig2_All, CL2.Contig1_All, CL2.Contig22_All, CL2.Contig25_All, CL2.Contig35_All, CL20016.Contig1_All, CL20024.Contig1_All, CL20025.Contig1_All, CL2003.Contig1_All, CL2003.Contig2_All, CL2003.Contig3_All, CL2003.Contig4_All, CL2005.Contig1_All, CL20052.Contig1_All, CL2008.Contig1_All, CL20081.Contig1_All, CL20094.Contig1_All, CL201.Contig5_All, CL20125.Contig1_All, CL2015.Contig1_All, CL20175.Contig1_All, CL20187.Contig1_All, CL2026.Contig1_All, CL20286.Contig1_All, CL2030.Contig1_All,

CL20321.Contig1_All, CL2033.Contig1_All,
CL20332.Contig1_All, CL20335.Contig1_All,
CL20345.Contig1_All, CL20358.Contig1_All,
CL20376.Contig1_All, CL20397.Contig1_All,
CL20405.Contig1_All, CL2041.Contig1_All,
CL2041.Contig2_All, CL2043.Contig1_All,
CL20454.Contig1_All, CL2046.Contig1_All,
CL20473.Contig1_All, CL20484.Contig1_All,
CL2049.Contig2_All, CL2049.Contig4_All,
CL20496.Contig1_All, CL20504.Contig1_All,
CL20518.Contig1_All, CL2053.Contig1_All,
CL2053.Contig2_All, CL20564.Contig1_All,
CL20574.Contig1_All, CL20578.Contig1_All,
CL20595.Contig1_All, CL20606.Contig1_All,
CL20608.Contig1_All, CL2062.Contig1_All,
CL20624.Contig1_All, CL20630.Contig1_All,
CL20640.Contig1_All, CL20664.Contig1_All,
CL20666.Contig1_All, CL20667.Contig1_All,
CL20673.Contig1_All, CL20680.Contig1_All,
CL20703.Contig1_All, CL20717.Contig1_All,
CL20752.Contig1_All, CL20766.Contig1_All,
CL20773.Contig1_All, CL20774.Contig1_All,
CL20788.Contig1_All, CL2079.Contig1_All,
CL20798.Contig1_All, CL20816.Contig1_All,
CL20826.Contig1_All, CL20832.Contig1_All,
CL20859.Contig1_All, CL20870.Contig1_All,
CL20874.Contig1_All, CL20875.Contig1_All,
CL20879.Contig1_All, CL20893.Contig1_All,
CL20897.Contig1_All, CL209.Contig4_All,
CL20900.Contig1_All, CL20934.Contig1_All,
CL20943.Contig1_All, CL21003.Contig1_All,
CL21011.Contig1_All, CL21015.Contig1_All,
CL2102.Contig1_All, CL21049.Contig1_All,
CL21051.Contig1_All, CL21053.Contig1_All,
CL21079.Contig1_All, CL2108.Contig1_All,
CL2108.Contig2_All, CL21082.Contig1_All,
CL21086.Contig1_All, CL21089.Contig1_All,
CL21093.Contig1_All, CL211.Contig10_All,
CL211.Contig11_All, CL211.Contig1_All, CL211.Contig2_All,
CL211.Contig3_All, CL211.Contig4_All, CL211.Contig5_All,
CL211.Contig6_All, CL211.Contig7_All, CL211.Contig8_All,
CL211.Contig9_All, CL21109.Contig1_All,
CL21134.Contig1_All, CL21148.Contig1_All,
CL2115.Contig1_All, CL21155.Contig1_All,
CL2116.Contig1_All, CL21173.Contig1_All,
CL21178.Contig1_All, CL2118.Contig2_All,
CL21201.Contig1_All, CL21227.Contig1_All,
CL21241.Contig1_All, CL21259.Contig1_All,
CL21274.Contig1_All, CL21275.Contig1_All,
CL21286.Contig1_All, CL2129.Contig2_All,
CL21298.Contig1_All, CL21299.Contig1_All,
CL213.Contig1_All, CL2130.Contig1_All,
CL21307.Contig1_All, CL21309.Contig1_All,
CL21321.Contig1_All, CL21323.Contig1_All,

			CL21335.Contig1_All, CL21352.Contig1_All, CL21356.Contig1_All, CL21365.Contig1_All, CL21372.Contig1_All, CL2138.Contig1_All, CL21393.Contig1_All, CL21399.Contig1_All, CL2143.Contig1_All, CL21451.Contig1_All, CL21453.Contig1_All, CL21473.Contig1_All, CL21476.Contig1_All, CL21511.Contig1_All, CL21539.Contig1_All, CL21562.Contig1_All, CL21565.Contig1_All, CL21568.Contig1_All, CL21576.Contig1_All, CL216.Contig1_All, CL216.Contig2_All, CL216.Contig3_All, CL216.Contig4_All, CL216.Contig5_All, CL2160.Contig1_All, CL21613.Contig1_All, CL21623.Contig1_All, CL2164.Contig1_All, CL21671.Contig1_All, CL21674.Contig1_All, CL21677.Contig1_All, CL21692.Contig1_All, CL21695.Contig1_All, CL21700.Contig1_All, CL21718.Contig1_All, CL21729.Contig1_All, CL21740.Contig1_All, CL21741.Contig1_All, CL21764.Contig1_All, CL21780.Contig1_All, CL2179.Contig1_All, CL2180.Contig1_All, CL2181.Contig1_All, CL2181.Contig2_All, CL2181.Contig3_All, CL21819.Contig1_All, CL21826.Contig1_All, CL2185.Contig1_All, CL2185.Contig2_All, CL21851.Contig1_All, CL21853.Contig1_All, CL21862.Contig1_All, CL21897.Contig1_All, CL21909.Contig1_All, CL2192.Contig1_All, CL2192.Contig3_All, CL21921.Contig1_All, CL21929.Contig1_All, CL21945.Contig1_All, CL21955.Contig1_All, CL21987.Contig1_All, CL22002.Contig1_All, CL22010.Contig1_All, CL22012.Contig1_All, CL22027.Contig1_All, CL2203.Contig1_All, CL22044.Contig1_All, CL2205.Contig1_All, CL22054.Contig1_All, CL22065.Contig1_All, CL2207.Contig1_All, CL22071.Contig1_All, CL22077.Contig1_All, CL22085.Contig1_All, CL22102.Contig1_All, CL2212.Contig1_All, CL2212.Contig2_All, CL22129.Contig1_All, CL22136.Contig1_All, CL2214.Contig1_All, CL22152.Contig1_All, CL22167.Contig1_All, CL22206.Contig1_All, CL2221.Contig1_All, CL2221.Contig2_All, CL22227.Contig1_All, CL2225.Contig1_All, CL2225.Contig2_All, CL22250.Contig1_All, CL2226.Contig1_All, CL2227.Contig1_All, CL22276.Contig1_All, CL22290.Contig1_All, CL22305.Contig1_All, CL22314.Contig1_All, CL22325.Contig1_All, CL22335.Contig1_All, CL22352.Contig1_All, CL22366.Contig1_All, CL22377.Contig1_All, CL22380.Contig1_All, CL22382.Contig1_All, CL22411.Contig1_All, CL22417.Contig1_All, CL2242.Contig1_All, CL22454.Contig1_All, CL22461.Contig1_All, CL22464.Contig1_All, CL22484.Contig1_All, CL22514.Contig1_All,

CL22539.Contig1_All, CL22545.Contig1_All,
CL22547.Contig1_All, CL22556.Contig1_All,
CL22573.Contig1_All, CL22599.Contig1_All,
CL22601.Contig1_All, CL22607.Contig1_All,
CL22639.Contig1_All, CL22641.Contig1_All,
CL22652.Contig1_All, CL22666.Contig1_All,
CL22682.Contig1_All, CL22703.Contig1_All,
CL22739.Contig1_All, CL22751.Contig1_All,
CL22771.Contig1_All, CL22776.Contig1_All,
CL22788.Contig1_All, CL22822.Contig1_All,
CL22823.Contig1_All, CL22879.Contig1_All,
CL22885.Contig1_All, CL22895.Contig1_All,
CL22913.Contig1_All, CL22966.Contig1_All,
CL22974.Contig1_All, CL22977.Contig1_All,
CL22990.Contig1_All, CL23.Contig10_All, CL23.Contig14_All,
CL23.Contig1_All, CL23.Contig3_All, CL23002.Contig1_All,
CL23006.Contig1_All, CL2301.Contig1_All,
CL23024.Contig1_All, CL23027.Contig1_All,
CL23042.Contig1_All, CL23047.Contig1_All,
CL23055.Contig1_All, CL23087.Contig1_All,
CL23117.Contig1_All, CL23127.Contig1_All,
CL23133.Contig1_All, CL23155.Contig1_All,
CL23161.Contig1_All, CL23167.Contig1_All,
CL2320.Contig1_All, CL2320.Contig2_All,
CL23202.Contig1_All, CL23220.Contig1_All,
CL23227.Contig1_All, CL2323.Contig1_All,
CL2323.Contig2_All, CL23231.Contig1_All,
CL23240.Contig1_All, CL23243.Contig1_All,
CL23271.Contig1_All, CL23272.Contig1_All,
CL23275.Contig1_All, CL23285.Contig1_All,
CL23294.Contig1_All, CL233.Contig2_All, CL233.Contig3_All,
CL233.Contig5_All, CL23312.Contig1_All,
CL23314.Contig1_All, CL23320.Contig1_All,
CL23328.Contig1_All, CL23330.Contig1_All,
CL23347.Contig1_All, CL23358.Contig1_All,
CL23363.Contig1_All, CL23369.Contig1_All,
CL23371.Contig1_All, CL23375.Contig1_All,
CL23377.Contig1_All, CL23391.Contig1_All,
CL234.Contig5_All, CL234.Contig6_All, CL2341.Contig1_All,
CL23423.Contig1_All, CL23435.Contig1_All,
CL23445.Contig1_All, CL23456.Contig1_All,
CL23475.Contig1_All, CL23524.Contig1_All,
CL23525.Contig1_All, CL23526.Contig1_All,
CL2353.Contig1_All, CL2353.Contig4_All,
CL23544.Contig1_All, CL2355.Contig1_All,
CL23560.Contig1_All, CL23570.Contig1_All,
CL23582.Contig1_All, CL23585.Contig1_All,
CL23588.Contig1_All, CL2359.Contig1_All,
CL23611.Contig1_All, CL23614.Contig1_All,
CL23615.Contig1_All, CL23629.Contig1_All,
CL23651.Contig1_All, CL23669.Contig1_All,
CL2367.Contig1_All, CL2367.Contig2_All,
CL2367.Contig3_All, CL23687.Contig1_All,
CL23703.Contig1_All, CL23718.Contig1_All,

		CL23735.Contig1_All, CL23767.Contig1_All, CL23768.Contig1_All, CL23777.Contig1_All, CL23781.Contig1_All, CL23791.Contig1_All, CL23799.Contig1_All, CL2381.Contig1_All, CL23818.Contig1_All, CL23834.Contig1_All, CL23845.Contig1_All, CL2387.Contig1_All, CL23898.Contig1_All, CL23909.Contig1_All, CL23911.Contig1_All, CL23940.Contig1_All, CL23945.Contig1_All, CL23965.Contig1_All, CL2398.Contig1_All, CL24005.Contig1_All, CL24009.Contig1_All, CL24022.Contig1_All, CL24023.Contig1_All, CL24032.Contig1_All, CL24033.Contig1_All, CL24036.Contig1_All, CL24045.Contig1_All, CL24063.Contig1_All, CL24066.Contig1_All, CL24070.Contig1_All, CL24071.Contig1_All, CL24077.Contig1_All, CL24081.Contig1_All, CL24088.Contig1_All, CL24100.Contig1_All, CL24115.Contig1_All, CL2413.Contig1_All, CL2414.Contig1_All, CL24148.Contig1_All, CL24211.Contig1_All, CL24213.Contig1_All, CL24251.Contig1_All, CL24281.Contig1_All, CL24287.Contig1_All, CL2429.Contig1_All, CL24319.Contig1_All, CL24371.Contig1_All, CL24379.Contig1_All, CL2439.Contig1_All, CL2439.Contig2_All, CL2445.Contig1_All, CL24467.Contig1_All, CL24469.Contig1_All, CL24479.Contig1_All, CL24508.Contig1_All, CL24517.Contig1_All, CL24524.Contig1_All, CL24532.Contig1_All, CL24536.Contig1_All, CL2455.Contig1_All, CL24569.Contig1_All, CL24579.Contig1_All, CL24599.Contig1_All, CL2460.Contig1_All, CL2460.Contig2_All, CL24604.Contig1_All, CL24605.Contig1_All, CL2461.Contig1_All, CL2461.Contig2_All, CL2461.Contig3_All, CL2461.Contig4_All, CL24615.Contig1_All, CL24617.Contig1_All, CL24622.Contig1_All, CL24635.Contig1_All, CL24640.Contig1_All, CL24664.Contig1_All, CL2468.Contig1_All, CL24683.Contig1_All, CL24705.Contig1_All, CL24709.Contig1_All, CL24714.Contig1_All, CL2475.Contig1_All, CL24764.Contig1_All, CL24786.Contig1_All, CL24825.Contig1_All, CL2483.Contig1_All, CL24831.Contig1_All, CL24839.Contig1_All, CL24878.Contig1_All, CL24890.Contig1_All, CL24900.Contig1_All, CL24913.Contig1_All, CL2498.Contig1_All, CL24981.Contig1_All, CL25.Contig11_All, CL25.Contig12_All, CL25.Contig14_All, CL25.Contig15_All, CL25.Contig2_All, CL25.Contig9_All, CL250.Contig1_All, CL250.Contig2_All, CL250.Contig3_All, CL250.Contig4_All, CL250.Contig5_All, CL250.Contig6_All, CL25005.Contig1_All, CL25009.Contig1_All, CL25040.Contig1_All, CL25044.Contig1_All, CL2505.Contig1_All,

		CL2505.Contig2_All, CL25051.Contig1_All, CL25053.Contig1_All, CL25107.Contig1_All, CL25126.Contig1_All, CL25142.Contig1_All, CL25143.Contig1_All, CL2515.Contig1_All, CL25160.Contig1_All, CL25192.Contig1_All, CL25210.Contig1_All, CL25220.Contig1_All, CL25269.Contig1_All, CL25277.Contig1_All, CL25282.Contig1_All, CL2531.Contig1_All, CL2531.Contig4_All, CL2531.Contig5_All, CL2531.Contig6_All, CL2531.Contig7_All, CL25324.Contig1_All, CL25340.Contig1_All, CL25352.Contig1_All, CL25363.Contig1_All, CL25376.Contig1_All, CL25387.Contig1_All, CL25407.Contig1_All, CL25414.Contig1_All, CL25435.Contig1_All, CL25441.Contig1_All, CL25456.Contig1_All, CL2546.Contig1_All, CL25467.Contig1_All, CL25472.Contig1_All, CL25479.Contig1_All, CL2549.Contig1_All, CL25504.Contig1_All, CL25514.Contig1_All, CL25515.Contig1_All, CL25532.Contig1_All, CL2554.Contig1_All, CL25555.Contig1_All, CL25556.Contig1_All, CL25567.Contig1_All, CL25603.Contig1_All, CL25606.Contig1_All, CL25612.Contig1_All, CL25641.Contig1_All, CL25658.Contig1_All, CL25667.Contig1_All, CL25675.Contig1_All, CL2568.Contig1_All, CL2568.Contig2_All, CL2568.Contig3_All, CL2568.Contig4_All, CL25688.Contig1_All, CL25689.Contig1_All, CL25705.Contig1_All, CL2571.Contig1_All, CL25710.Contig1_All, CL25761.Contig1_All, CL2579.Contig1_All, CL25791.Contig1_All, CL25792.Contig1_All, CL25793.Contig1_All, CL2582.Contig1_All, CL25833.Contig1_All, CL25835.Contig1_All, CL25836.Contig1_All, CL25841.Contig1_All, CL25842.Contig1_All, CL25845.Contig1_All, CL25852.Contig1_All, CL25863.Contig1_All, CL25898.Contig1_All, CL2591.Contig1_All, CL25939.Contig1_All, CL2594.Contig1_All, CL25955.Contig1_All, CL25963.Contig1_All, CL25987.Contig1_All, CL25992.Contig1_All, CL25996.Contig1_All, CL26011.Contig1_All, CL26021.Contig1_All, CL26026.Contig1_All, CL26066.Contig1_All, CL26096.Contig1_All, CL26121.Contig1_All, CL26145.Contig1_All, CL26163.Contig1_All, CL2618.Contig1_All, CL2618.Contig2_All, CL262.Contig1_All, CL262.Contig2_All, CL262.Contig3_All, CL262.Contig4_All, CL262.Contig5_All, CL262.Contig6_All, CL262.Contig7_All, CL2621.Contig1_All, CL2621.Contig2_All, CL26257.Contig1_All, CL26273.Contig1_All, CL26299.Contig1_All, CL2630.Contig1_All, CL2630.Contig2_All, CL26303.Contig1_All, CL26309.Contig1_All, CL26366.Contig1_All, CL264.Contig1_All, CL264.Contig2_All,

		CL264.Contig3_All, CL264.Contig4_All, CL264.Contig5_All, CL264.Contig6_All, CL264.Contig7_All, CL264.Contig8_All, CL26426.Contig1_All, CL2643.Contig1_All, CL2643.Contig2_All, CL2643.Contig3_All, CL26460.Contig1_All, CL26467.Contig1_All, CL2647.Contig1_All, CL26483.Contig1_All, CL26484.Contig1_All, CL26496.Contig1_All, CL26501.Contig1_All, CL26518.Contig1_All, CL26522.Contig1_All, CL26534.Contig1_All, CL2655.Contig1_All, CL26560.Contig1_All, CL26575.Contig1_All, CL26584.Contig1_All, CL26593.Contig1_All, CL26598.Contig1_All, CL266.Contig1_All, CL266.Contig2_All, CL266.Contig3_All, CL266.Contig4_All, CL266.Contig5_All, CL266.Contig6_All, CL26610.Contig1_All, CL26623.Contig1_All, CL26639.Contig1_All, CL26645.Contig1_All, CL26673.Contig1_All, CL26676.Contig1_All, CL2668.Contig1_All, CL26687.Contig1_All, CL2670.Contig1_All, CL26700.Contig1_All, CL26701.Contig1_All, CL26736.Contig1_All, CL2674.Contig1_All, CL26742.Contig1_All, CL26750.Contig1_All, CL26751.Contig1_All, CL26756.Contig1_All, CL26769.Contig1_All, CL26770.Contig1_All, CL2679.Contig1_All, CL26793.Contig1_All, CL26806.Contig1_All, CL2682.Contig1_All, CL26827.Contig1_All, CL2686.Contig1_All, CL26876.Contig1_All, CL26882.Contig1_All, CL26888.Contig1_All, CL26896.Contig1_All, CL26904.Contig1_All, CL26929.Contig1_All, CL26933.Contig1_All, CL26948.Contig1_All, CL26953.Contig1_All, CL26958.Contig1_All, CL26967.Contig1_All, CL26978.Contig1_All, CL27010.Contig1_All, CL27025.Contig1_All, CL27060.Contig1_All, CL27064.Contig1_All, CL27068.Contig1_All, CL27069.Contig1_All, CL27071.Contig1_All, CL27097.Contig1_All, CL271.Contig1_All, CL271.Contig7_All, CL271.Contig8_All, CL27120.Contig1_All, CL27126.Contig1_All, CL2717.Contig1_All, CL27180.Contig1_All, CL27221.Contig1_All, CL2724.Contig1_All, CL27255.Contig1_All, CL27261.Contig1_All, CL27264.Contig1_All, CL27267.Contig1_All, CL27281.Contig1_All, CL27289.Contig1_All, CL2729.Contig1_All, CL27290.Contig1_All, CL273.Contig6_All, CL27307.Contig1_All, CL27308.Contig1_All, CL27321.Contig1_All, CL27352.Contig1_All, CL27370.Contig1_All, CL274.Contig2_All, CL27416.Contig1_All, CL27432.Contig1_All, CL27439.Contig1_All, CL2745.Contig1_All, CL2745.Contig2_All, CL2746.Contig1_All, CL27470.Contig1_All, CL27482.Contig1_All, CL27486.Contig1_All, CL275.Contig2_All, CL27506.Contig1_All, CL27552.Contig1_All,

		CL27592.Contig1_All, CL27603.Contig1_All, CL2761.Contig1_All, CL2762.Contig1_All, CL27620.Contig1_All, CL27648.Contig1_All, CL27661.Contig1_All, CL27703.Contig1_All, CL27706.Contig1_All, CL27710.Contig1_All, CL27720.Contig1_All, CL27744.Contig1_All, CL2775.Contig1_All, CL27778.Contig1_All, CL27788.Contig1_All, CL27796.Contig1_All, CL27798.Contig1_All, CL278.Contig1_All, CL278.Contig2_All, CL278.Contig6_All, CL278.Contig7_All, CL27806.Contig1_All, CL27816.Contig1_All, CL2783.Contig1_All, CL27833.Contig1_All, CL27834.Contig1_All, CL27867.Contig1_All, CL27887.Contig1_All, CL27895.Contig1_All, CL27901.Contig1_All, CL27904.Contig1_All, CL27907.Contig1_All, CL27926.Contig1_All, CL2794.Contig1_All, CL2796.Contig1_All, CL2796.Contig2_All, CL2796.Contig3_All, CL2796.Contig4_All, CL27973.Contig1_All, CL27998.Contig1_All, CL28009.Contig1_All, CL28088.Contig1_All, CL281.Contig1_All, CL281.Contig2_All, CL281.Contig3_All, CL281.Contig4_All, CL281.Contig5_All, CL281.Contig6_All, CL281.Contig7_All, CL28135.Contig1_All, CL2831.Contig1_All, CL2831.Contig2_All, CL2839.Contig1_All, CL285.Contig2_All, CL2854.Contig1_All, CL2858.Contig1_All, CL2858.Contig2_All, CL2860.Contig3_All, CL2874.Contig1_All, CL2874.Contig2_All, CL2876.Contig1_All, CL288.Contig1_All, CL288.Contig2_All, CL288.Contig3_All, CL288.Contig6_All, CL288.Contig7_All, CL288.Contig8_All, CL2887.Contig1_All, CL2887.Contig2_All, CL2902.Contig1_All, CL2903.Contig1_All, CL2929.Contig1_All, CL2932.Contig1_All, CL2942.Contig2_All, CL2942.Contig3_All, CL2950.Contig1_All, CL2958.Contig1_All, CL2958.Contig2_All, CL2961.Contig4_All, CL2980.Contig1_All, CL2993.Contig1_All, CL2996.Contig1_All, CL3003.Contig1_All, CL3011.Contig1_All, CL3014.Contig1_All, CL3022.Contig1_All, CL3027.Contig1_All, CL3031.Contig1_All, CL3034.Contig1_All, CL3039.Contig1_All, CL3041.Contig1_All, CL3049.Contig1_All, CL3057.Contig1_All, CL3061.Contig1_All, CL3062.Contig2_All, CL3064.Contig1_All, CL3070.Contig1_All, CL3070.Contig2_All, CL3070.Contig3_All, CL3083.Contig1_All, CL3103.Contig1_All, CL311.Contig1_All, CL3116.Contig1_All, CL3118.Contig1_All, CL3134.Contig1_All, CL3143.Contig1_All, CL3143.Contig2_All, CL315.Contig1_All, CL315.Contig2_All, CL315.Contig3_All, CL315.Contig4_All, CL315.Contig5_All, CL315.Contig6_All, CL315.Contig7_All, CL315.Contig8_All, CL3152.Contig1_All, CL3170.Contig1_All, CL3176.Contig1_All, CL318.Contig2_All, CL318.Contig4_All, CL3182.Contig1_All, CL3182.Contig2_All,

			CL3186.Contig1_All, CL32.Contig12_All, CL3200.Contig1_All, CL3201.Contig1_All, CL3226.Contig1_All, CL323.Contig1_All, CL323.Contig2_All, CL323.Contig3_All, CL323.Contig4_All, CL3230.Contig1_All, CL3243.Contig1_All, CL3243.Contig2_All, CL3246.Contig1_All, CL3246.Contig2_All, CL3246.Contig3_All, CL3246.Contig4_All, CL3260.Contig1_All, CL3264.Contig1_All, CL3264.Contig2_All, CL3270.Contig1_All, CL3270.Contig2_All, CL3270.Contig3_All, CL3270.Contig4_All, CL3285.Contig1_All, CL3285.Contig2_All, CL3308.Contig2_All, CL331.Contig1_All, CL331.Contig2_All, CL331.Contig3_All, CL331.Contig4_All, CL3314.Contig1_All, CL3314.Contig2_All, CL3321.Contig1_All, CL3321.Contig2_All, CL3321.Contig3_All, CL3328.Contig1_All, CL334.Contig1_All, CL334.Contig2_All, CL334.Contig3_All, CL3342.Contig1_All, CL3346.Contig1_All, CL3346.Contig2_All, CL3351.Contig1_All, CL3356.Contig1_All, CL3367.Contig1_All, CL3367.Contig2_All, CL3376.Contig1_All, CL3398.Contig1_All, CL3401.Contig1_All, CL3408.Contig1_All, CL3408.Contig2_All, CL3408.Contig3_All, CL3427.Contig1_All, CL3433.Contig1_All, CL3433.Contig2_All, CL3434.Contig1_All, CL3437.Contig1_All, CL3444.Contig1_All, CL3445.Contig1_All, CL3445.Contig2_All, CL3445.Contig3_All, CL3448.Contig1_All, CL3448.Contig2_All, CL345.Contig5_All, CL3452.Contig1_All, CL3452.Contig2_All, CL3456.Contig1_All, CL3457.Contig1_All, CL3466.Contig1_All, CL3466.Contig2_All, CL3473.Contig1_All, CL3481.Contig1_All, CL3481.Contig2_All, CL3481.Contig3_All, CL349.Contig1_All, CL349.Contig2_All, CL349.Contig3_All, CL349.Contig5_All, CL3494.Contig1_All, CL35.Contig11_All, CL35.Contig16_All, CL35.Contig1_All, CL35.Contig22_All, CL35.Contig4_All, CL35.Contig6_All, CL35.Contig8_All, CL3505.Contig1_All, CL3508.Contig1_All, CL3509.Contig1_All, CL351.Contig1_All, CL351.Contig5_All, CL3528.Contig1_All, CL353.Contig1_All, CL353.Contig2_All, CL353.Contig3_All, CL353.Contig4_All, CL353.Contig5_All, CL3530.Contig1_All, CL3532.Contig1_All, CL3532.Contig2_All, CL3532.Contig3_All, CL3538.Contig1_All, CL3545.Contig1_All, CL3549.Contig1_All, CL3564.Contig1_All, CL3572.Contig1_All, CL3572.Contig2_All, CL3576.Contig1_All, CL3576.Contig2_All, CL3581.Contig1_All, CL3581.Contig2_All, CL3581.Contig3_All, CL3581.Contig4_All, CL3587.Contig1_All, CL3590.Contig1_All, CL36.Contig13_All, CL36.Contig15_All, CL36.Contig16_All, CL36.Contig17_All, CL36.Contig3_All, CL36.Contig9_All, CL3610.Contig1_All, CL3618.Contig1_All, CL3622.Contig1_All, CL3629.Contig1_All, CL3646.Contig1_All, CL3646.Contig2_All, CL3654.Contig1_All,

		CL3665.Contig1_All, CL3673.Contig1_All, CL3695.Contig1_All, CL370.Contig2_All, CL370.Contig3_All, CL370.Contig8_All, CL3723.Contig1_All, CL3723.Contig2_All, CL3725.Contig1_All, CL3734.Contig1_All, CL374.Contig5_All, CL3740.Contig1_All, CL3740.Contig2_All, CL3744.Contig1_All, CL3750.Contig1_All, CL3770.Contig1_All, CL3773.Contig1_All, CL3777.Contig1_All, CL3788.Contig1_All, CL3799.Contig1_All, CL3799.Contig2_All, CL38.Contig10_All, CL38.Contig13_All, CL38.Contig8_All, CL3803.Contig1_All, CL3804.Contig1_All, CL3804.Contig2_All, CL3805.Contig1_All, CL3826.Contig1_All, CL3831.Contig1_All, CL3838.Contig1_All, CL3838.Contig2_All, CL384.Contig3_All, CL384.Contig8_All, CL3840.Contig1_All, CL3841.Contig1_All, CL3846.Contig1_All, CL385.Contig1_All, CL385.Contig2_All, CL385.Contig3_All, CL3866.Contig1_All, CL3879.Contig1_All, CL388.Contig1_All, CL388.Contig2_All, CL388.Contig3_All, CL388.Contig4_All, CL388.Contig5_All, CL3882.Contig1_All, CL3884.Contig1_All, CL39.Contig10_All, CL39.Contig12_All, CL39.Contig14_All, CL39.Contig2_All, CL39.Contig3_All, CL39.Contig4_All, CL39.Contig5_All, CL39.Contig6_All, CL39.Contig8_All, CL39.Contig9_All, CL3905.Contig1_All, CL3914.Contig1_All, CL3915.Contig1_All, CL3916.Contig1_All, CL3916.Contig2_All, CL3932.Contig1_All, CL3932.Contig2_All, CL3938.Contig1_All, CL3938.Contig2_All, CL3938.Contig3_All, CL3942.Contig1_All, CL3943.Contig1_All, CL395.Contig2_All, CL395.Contig3_All, CL395.Contig5_All, CL395.Contig6_All, CL3952.Contig1_All, CL3955.Contig1_All, CL3957.Contig1_All, CL3957.Contig2_All, CL3983.Contig1_All, CL3983.Contig2_All, CL3984.Contig1_All, CL3988.Contig1_All, CL399.Contig1_All, CL399.Contig2_All, CL399.Contig3_All, CL399.Contig4_All, CL399.Contig5_All, CL399.Contig6_All, CL3991.Contig1_All, CL3991.Contig2_All, CL3992.Contig1_All, CL3996.Contig1_All, CL3999.Contig1_All, CL4.Contig17_All, CL4.Contig25_All, CL4.Contig32_All, CL4.Contig34_All, CL4.Contig35_All, CL4.Contig4_All, CL4.Contig5_All, CL40.Contig10_All, CL40.Contig11_All, CL40.Contig1_All, CL40.Contig2_All, CL40.Contig3_All, CL40.Contig4_All, CL40.Contig5_All, CL40.Contig6_All, CL40.Contig7_All, CL40.Contig8_All, CL40.Contig9_All, CL4024.Contig1_All, CL403.Contig1_All, CL4030.Contig1_All, CL4031.Contig1_All, CL4042.Contig1_All, CL4048.Contig1_All, CL4055.Contig1_All, CL4062.Contig1_All, CL4062.Contig2_All, CL4077.Contig1_All, CL4094.Contig1_All, CL4095.Contig1_All, CL4098.Contig1_All, CL4098.Contig2_All, CL4099.Contig1_All, CL4101.Contig1_All, CL4101.Contig2_All, CL4125.Contig1_All, CL4129.Contig1_All, CL4140.Contig1_All, CL4141.Contig1_All, CL4161.Contig1_All,

			CL4168.Contig1_All, CL4170.Contig1_All, CL4171.Contig1_All, CL4171.Contig2_All, CL4174.Contig1_All, CL4177.Contig1_All, CL4182.Contig1_All, CL4182.Contig2_All, CL4183.Contig1_All, CL4190.Contig1_All, CL4190.Contig2_All, CL4199.Contig1_All, CL4205.Contig1_All, CL4224.Contig1_All, CL4229.Contig1_All, CL4231.Contig1_All, CL4231.Contig2_All, CL4268.Contig1_All, CL4275.Contig1_All, CL4295.Contig1_All, CL4295.Contig2_All, CL4299.Contig1_All, CL4299.Contig2_All, CL4305.Contig1_All, CL4306.Contig1_All, CL4306.Contig2_All, CL4312.Contig1_All, CL4312.Contig2_All, CL4316.Contig1_All, CL4331.Contig1_All, CL4342.Contig1_All, CL4347.Contig1_All, CL4347.Contig2_All, CL4347.Contig3_All, CL4347.Contig4_All, CL4353.Contig1_All, CL4359.Contig1_All, CL4362.Contig1_All, CL4363.Contig1_All, CL4363.Contig3_All, CL437.Contig1_All, CL437.Contig2_All, CL437.Contig3_All, CL437.Contig4_All, CL437.Contig5_All, CL437.Contig6_All, CL4391.Contig1_All, CL4392.Contig1_All, CL4403.Contig1_All, CL4405.Contig1_All, CL4410.Contig1_All, CL4415.Contig1_All, CL4430.Contig1_All, CL4432.Contig1_All, CL444.Contig3_All, CL4445.Contig1_All, CL4448.Contig1_All, CL4450.Contig1_All, CL4453.Contig1_All, CL4455.Contig1_All, CL4462.Contig1_All, CL4468.Contig1_All, CL447.Contig1_All, CL447.Contig2_All, CL447.Contig3_All, CL447.Contig4_All, CL447.Contig5_All, CL447.Contig6_All, CL4471.Contig1_All, CL4477.Contig1_All, CL4477.Contig2_All, CL4484.Contig1_All, CL4484.Contig2_All, CL4494.Contig1_All, CL4507.Contig1_All, CL4509.Contig1_All, CL4511.Contig1_All, CL452.Contig1_All, CL452.Contig2_All, CL452.Contig3_All, CL452.Contig4_All, CL452.Contig5_All, CL4520.Contig1_All, CL4525.Contig1_All, CL4529.Contig1_All, CL4534.Contig1_All, CL4535.Contig1_All, CL4536.Contig1_All, CL4537.Contig1_All, CL4543.Contig1_All, CL4549.Contig1_All, CL456.Contig1_All, CL456.Contig2_All, CL456.Contig3_All, CL456.Contig4_All, CL456.Contig5_All, CL456.Contig6_All, CL4568.Contig1_All, CL4571.Contig1_All, CL4571.Contig2_All, CL4574.Contig1_All, CL4574.Contig2_All, CL4576.Contig1_All, CL4578.Contig1_All, CL4591.Contig2_All, CL4591.Contig3_All, CL4593.Contig1_All, CL4608.Contig1_All, CL4615.Contig1_All, CL4616.Contig1_All, CL4628.Contig1_All, CL4628.Contig2_All, CL4629.Contig3_All, CL4630.Contig1_All, CL4632.Contig1_All, CL4632.Contig2_All, CL4636.Contig1_All, CL4647.Contig1_All, CL4647.Contig3_All, CL4666.Contig1_All, CL4675.Contig1_All,

			CL4678.Contig1_All, CL468.Contig1_All, CL468.Contig2_All, CL468.Contig3_All, CL4682.Contig1_All, CL4685.Contig1_All, CL4716.Contig1_All, CL4716.Contig2_All, CL4723.Contig2_All, CL4724.Contig1_All, CL4743.Contig1_All, CL4751.Contig1_All, CL476.Contig1_All, CL476.Contig2_All, CL476.Contig3_All, CL476.Contig4_All, CL476.Contig5_All, CL4775.Contig1_All, CL479.Contig2_All, CL479.Contig3_All, CL479.Contig5_All, CL4793.Contig1_All, CL4806.Contig1_All, CL4811.Contig1_All, CL4821.Contig1_All, CL4836.Contig1_All, CL4837.Contig1_All, CL4842.Contig1_All, CL4845.Contig1_All, CL4845.Contig2_All, CL4846.Contig1_All, CL4849.Contig1_All, CL4850.Contig1_All, CL4850.Contig2_All, CL4850.Contig3_All, CL4858.Contig1_All, CL486.Contig1_All, CL486.Contig2_All, CL486.Contig3_All, CL486.Contig4_All, CL4869.Contig1_All, CL4873.Contig1_All, CL4874.Contig1_All, CL4892.Contig1_All, CL4892.Contig2_All, CL4903.Contig1_All, CL4917.Contig1_All, CL4932.Contig1_All, CL4944.Contig1_All, CL4946.Contig2_All, CL4946.Contig3_All, CL4951.Contig1_All, CL4953.Contig1_All, CL4953.Contig2_All, CL4954.Contig1_All, CL4954.Contig2_All, CL4961.Contig1_All, CL4968.Contig1_All, CL4972.Contig1_All, CL4974.Contig1_All, CL4975.Contig1_All, CL498.Contig1_All, CL4980.Contig1_All, CL4987.Contig1_All, CL50.Contig10_All, CL50.Contig11_All, CL50.Contig12_All, CL50.Contig13_All, CL50.Contig14_All, CL50.Contig15_All, CL50.Contig16_All, CL50.Contig1_All, CL50.Contig2_All, CL50.Contig3_All, CL50.Contig4_All, CL50.Contig5_All, CL50.Contig6_All, CL50.Contig7_All, CL50.Contig8_All, CL50.Contig9_All, CL5017.Contig1_All, CL5020.Contig1_All, CL5020.Contig2_All, CL5024.Contig1_All, CL5036.Contig1_All, CL504.Contig1_All, CL504.Contig2_All, CL504.Contig3_All, CL504.Contig4_All, CL504.Contig5_All, CL5042.Contig1_All, CL505.Contig1_All, CL505.Contig2_All, CL505.Contig3_All, CL505.Contig4_All, CL5066.Contig1_All, CL5068.Contig1_All, CL5072.Contig1_All, CL5081.Contig1_All, CL5098.Contig1_All, CL51.Contig10_All, CL51.Contig11_All, CL51.Contig12_All, CL51.Contig13_All, CL51.Contig14_All, CL51.Contig15_All, CL51.Contig17_All, CL51.Contig1_All, CL51.Contig2_All, CL51.Contig3_All, CL51.Contig4_All, CL51.Contig5_All, CL51.Contig6_All, CL51.Contig8_All, CL51.Contig9_All, CL5100.Contig1_All, CL5138.Contig1_All, CL5150.Contig1_All, CL5173.Contig1_All, CL5175.Contig1_All, CL5176.Contig1_All, CL5176.Contig2_All, CL5179.Contig1_All, CL5179.Contig2_All, CL518.Contig1_All, CL518.Contig2_All, CL518.Contig3_All, CL518.Contig4_All, CL518.Contig5_All, CL5182.Contig1_All, CL5183.Contig1_All, CL5183.Contig2_All, CL5183.Contig3_All, CL52.Contig7_All, CL522.Contig1_All, CL5220.Contig1_All, CL5224.Contig1_All,

		CL5225.Contig1_All, CL5230.Contig1_All, CL5231.Contig1_All, CL5236.Contig1_All, CL5237.Contig1_All, CL5243.Contig1_All, CL5251.Contig1_All, CL5253.Contig1_All, CL5261.Contig1_All, CL5261.Contig2_All, CL5264.Contig1_All, CL5264.Contig2_All, CL5271.Contig1_All, CL5276.Contig1_All, CL5282.Contig1_All, CL5282.Contig2_All, CL5286.Contig1_All, CL5288.Contig1_All, CL5288.Contig2_All, CL5288.Contig3_All, CL53.Contig10_All, CL53.Contig11_All, CL53.Contig1_All, CL53.Contig3_All, CL53.Contig6_All, CL53.Contig7_All, CL53.Contig8_All, CL53.Contig9_All, CL530.Contig1_All, CL530.Contig2_All, CL530.Contig3_All, CL5300.Contig1_All, CL5309.Contig1_All, CL5312.Contig1_All, CL5317.Contig1_All, CL5321.Contig1_All, CL5325.Contig1_All, CL5333.Contig1_All, CL5337.Contig1_All, CL535.Contig1_All, CL5359.Contig1_All, CL5363.Contig1_All, CL537.Contig2_All, CL537.Contig5_All, CL5385.Contig1_All, CL5385.Contig2_All, CL5393.Contig1_All, CL5395.Contig1_All, CL54.Contig17_All, CL54.Contig18_All, CL54.Contig19_All, CL54.Contig3_All, CL5400.Contig1_All, CL5401.Contig1_All, CL5402.Contig1_All, CL5408.Contig1_All, CL5410.Contig1_All, CL5425.Contig1_All, CL5431.Contig1_All, CL5436.Contig1_All, CL5440.Contig1_All, CL5440.Contig2_All, CL5443.Contig1_All, CL5445.Contig1_All, CL5475.Contig1_All, CL5477.Contig1_All, CL5478.Contig1_All, CL5494.Contig1_All, CL5495.Contig1_All, CL55.Contig2_All, CL55.Contig4_All, CL5500.Contig1_All, CL5500.Contig2_All, CL5516.Contig1_All, CL5523.Contig1_All, CL5526.Contig1_All, CL5538.Contig1_All, CL5546.Contig1_All, CL5549.Contig1_All, CL5549.Contig2_All, CL5560.Contig1_All, CL5569.Contig1_All, CL557.Contig1_All, CL557.Contig2_All, CL557.Contig3_All, CL557.Contig4_All, CL5583.Contig1_All, CL5585.Contig1_All, CL5596.Contig1_All, CL56.Contig10_All, CL56.Contig12_All, CL56.Contig9_All, CL560.Contig1_All, CL5607.Contig2_All, CL5615.Contig1_All, CL5618.Contig1_All, CL5618.Contig2_All, CL5621.Contig1_All, CL5633.Contig1_All, CL5633.Contig2_All, CL5637.Contig1_All, CL5637.Contig2_All, CL5639.Contig1_All, CL5649.Contig1_All, CL5661.Contig1_All, CL5662.Contig1_All, CL5664.Contig1_All, CL5678.Contig1_All, CL5680.Contig1_All, CL5684.Contig1_All, CL5685.Contig1_All, CL569.Contig2_All, CL569.Contig5_All, CL569.Contig6_All, CL5690.Contig1_All, CL5691.Contig1_All, CL5691.Contig2_All, CL5703.Contig1_All, CL5709.Contig1_All, CL5737.Contig1_All, CL5745.Contig1_All, CL5755.Contig1_All, CL5769.Contig1_All, CL5787.Contig1_All, CL5793.Contig1_All,

			CL5819.Contig1_All, CL582.Contig1_All, CL5829.Contig1_All, CL5837.Contig1_All, CL5843.Contig1_All, CL5856.Contig1_All, CL5864.Contig1_All, CL5864.Contig2_All, CL587.Contig1_All, CL587.Contig3_All, CL587.Contig4_All, CL587.Contig5_All, CL587.Contig6_All, CL5870.Contig1_All, CL5884.Contig1_All, CL589.Contig1_All, CL589.Contig2_All, CL589.Contig3_All, CL5906.Contig1_All, CL5922.Contig1_All, CL5925.Contig1_All, CL593.Contig1_All, CL593.Contig2_All, CL593.Contig3_All, CL593.Contig4_All, CL593.Contig5_All, CL5935.Contig1_All, CL5936.Contig1_All, CL5936.Contig2_All, CL5937.Contig1_All, CL5938.Contig1_All, CL595.Contig2_All, CL5950.Contig1_All, CL5952.Contig1_All, CL5957.Contig1_All, CL5968.Contig1_All, CL5985.Contig1_All, CL5985.Contig2_All, CL6.Contig1_All, CL6.Contig25_All, CL6002.Contig1_All, CL6002.Contig2_All, CL6009.Contig1_All, CL6009.Contig2_All, CL6013.Contig1_All, CL6017.Contig1_All, CL6025.Contig1_All, CL6039.Contig1_All, CL6039.Contig2_All, CL6042.Contig1_All, CL6051.Contig1_All, CL6058.Contig1_All, CL6058.Contig2_All, CL606.Contig1_All, CL6064.Contig1_All, CL6079.Contig1_All, CL6079.Contig2_All, CL6083.Contig1_All, CL6085.Contig1_All, CL6089.Contig1_All, CL609.Contig1_All, CL609.Contig2_All, CL609.Contig3_All, CL609.Contig4_All, CL6091.Contig1_All, CL6096.Contig1_All, CL61.Contig10_All, CL61.Contig1_All, CL61.Contig2_All, CL61.Contig3_All, CL61.Contig4_All, CL61.Contig5_All, CL61.Contig6_All, CL61.Contig7_All, CL61.Contig8_All, CL61.Contig9_All, CL6101.Contig1_All, CL612.Contig1_All, CL6124.Contig1_All, CL613.Contig1_All, CL613.Contig2_All, CL613.Contig3_All, CL613.Contig4_All, CL6141.Contig1_All, CL6141.Contig2_All, CL6145.Contig1_All, CL6146.Contig1_All, CL6153.Contig1_All, CL6153.Contig2_All, CL6155.Contig1_All, CL6172.Contig1_All, CL6199.Contig1_All, CL6199.Contig2_All, CL621.Contig1_All, CL621.Contig2_All, CL6212.Contig1_All, CL6214.Contig1_All, CL6224.Contig1_All, CL6233.Contig1_All, CL6242.Contig1_All, CL625.Contig1_All, CL6259.Contig1_All, CL626.Contig1_All, CL6263.Contig1_All, CL6263.Contig2_All, CL6267.Contig1_All, CL627.Contig1_All, CL6287.Contig1_All, CL6290.Contig1_All, CL6299.Contig1_All, CL63.Contig3_All, CL63.Contig5_All, CL63.Contig7_All, CL6305.Contig1_All, CL6319.Contig1_All, CL6319.Contig2_All, CL6330.Contig1_All, CL6371.Contig1_All, CL6377.Contig1_All, CL6388.Contig1_All, CL6395.Contig1_All, CL6396.Contig1_All, CL6401.Contig1_All, CL6402.Contig1_All, CL6407.Contig1_All, CL641.Contig1_All, CL641.Contig2_All, CL641.Contig3_All, CL6410.Contig2_All, CL644.Contig1_All, CL644.Contig2_All, CL644.Contig3_All, CL644.Contig4_All, CL644.Contig5_All, CL644.Contig6_All, CL644.Contig7_All, CL644.Contig8_All, CL6441.Contig1_All, CL6442.Contig1_All,

CL6454.Contig1_All, CL6454.Contig2_All,
CL6475.Contig1_All, CL6479.Contig1_All,
CL6481.Contig1_All, CL6481.Contig2_All,
CL6485.Contig1_All, CL649.Contig1_All, CL649.Contig2_All,
CL6493.Contig1_All, CL6501.Contig1_All,
CL6506.Contig1_All, CL6507.Contig1_All,
CL6509.Contig1_All, CL651.Contig1_All, CL6511.Contig1_All,
CL6515.Contig1_All, CL6521.Contig1_All,
CL6522.Contig1_All, CL6534.Contig1_All,
CL6538.Contig1_All, CL6557.Contig1_All,
CL6557.Contig2_All, CL6559.Contig1_All, CL656.Contig1_All,
CL656.Contig2_All, CL656.Contig3_All, CL656.Contig4_All,
CL6565.Contig1_All, CL657.Contig1_All, CL657.Contig3_All,
CL657.Contig4_All, CL657.Contig5_All, CL657.Contig6_All,
CL6579.Contig1_All, CL6581.Contig1_All,
CL6587.Contig1_All, CL6596.Contig1_All, CL66.Contig4_All,
CL6607.Contig1_All, CL6612.Contig1_All,
CL6612.Contig2_All, CL6613.Contig1_All,
CL6613.Contig2_All, CL6617.Contig1_All,
CL6617.Contig2_All, CL6626.Contig1_All,
CL6637.Contig1_All, CL6640.Contig1_All,
CL6652.Contig1_All, CL6662.Contig1_All,
CL6696.Contig1_All, CL6700.Contig1_All,
CL6705.Contig1_All, CL6709.Contig1_All,
CL6715.Contig1_All, CL6722.Contig1_All, CL674.Contig1_All,
CL674.Contig2_All, CL674.Contig3_All, CL674.Contig4_All,
CL674.Contig5_All, CL6744.Contig1_All, CL6752.Contig1_All,
CL6753.Contig1_All, CL6753.Contig2_All,
CL6763.Contig1_All, CL6772.Contig1_All,
CL6791.Contig1_All, CL6792.Contig1_All,
CL6792.Contig2_All, CL68.Contig1_All, CL68.Contig3_All,
CL68.Contig4_All, CL68.Contig6_All, CL68.Contig8_All,
CL6805.Contig1_All, CL6817.Contig1_All,
CL6840.Contig1_All, CL6842.Contig1_All,
CL6861.Contig1_All, CL6865.Contig1_All,
CL6870.Contig1_All, CL6871.Contig1_All,
CL6879.Contig1_All, CL6879.Contig2_All,
CL6889.Contig1_All, CL69.Contig1_All, CL69.Contig2_All,
CL69.Contig3_All, CL69.Contig4_All, CL69.Contig5_All,
CL69.Contig6_All, CL69.Contig7_All, CL69.Contig8_All,
CL6913.Contig1_All, CL6914.Contig1_All,
CL6917.Contig1_All, CL6928.Contig1_All,
CL6929.Contig1_All, CL697.Contig1_All, CL6970.Contig1_All,
CL6973.Contig1_All, CL6976.Contig1_All,
CL6981.Contig1_All, CL6984.Contig1_All, CL699.Contig1_All,
CL7.Contig25_All, CL7.Contig28_All, CL70.Contig8_All,
CL7007.Contig1_All, CL7030.Contig1_All,
CL7032.Contig1_All, CL7040.Contig1_All,
CL7049.Contig1_All, CL7052.Contig1_All,
CL7057.Contig1_All, CL7057.Contig2_All,
CL7063.Contig1_All, CL7063.Contig2_All,
CL7069.Contig1_All, CL7072.Contig1_All,
CL7085.Contig1_All, CL7090.Contig1_All,
CL7095.Contig1_All, CL71.Contig2_All, CL71.Contig3_All,

			CL71.Contig7_All, CL710.Contig1_All, CL710.Contig2_All, CL710.Contig3_All, CL7101.Contig1_All, CL7104.Contig1_All, CL7112.Contig1_All, CL7125.Contig1_All, CL7130.Contig1_All, CL7142.Contig1_All, CL7150.Contig1_All, CL7155.Contig1_All, CL7158.Contig1_All, CL7158.Contig2_All, CL7167.Contig1_All, CL717.Contig1_All, CL717.Contig2_All, CL717.Contig3_All, CL717.Contig4_All, CL7174.Contig1_All, CL7181.Contig1_All, CL7184.Contig2_All, CL7184.Contig3_All, CL7192.Contig1_All, CL7192.Contig2_All, CL7195.Contig2_All, CL7202.Contig1_All, CL7230.Contig1_All, CL7240.Contig1_All, CL7240.Contig2_All, CL7244.Contig1_All, CL7244.Contig2_All, CL7254.Contig1_All, CL7255.Contig1_All, CL7257.Contig1_All, CL7260.Contig1_All, CL7261.Contig1_All, CL7261.Contig2_All, CL7271.Contig1_All, CL7276.Contig1_All, CL7276.Contig2_All, CL7279.Contig1_All, CL728.Contig1_All, CL728.Contig2_All, CL728.Contig3_All, CL7280.Contig1_All, CL7289.Contig1_All, CL729.Contig1_All, CL729.Contig2_All, CL729.Contig3_All, CL729.Contig4_All, CL7296.Contig1_All, CL7303.Contig1_All, CL7303.Contig2_All, CL7314.Contig1_All, CL7315.Contig1_All, CL7326.Contig1_All, CL7335.Contig1_All, CL7354.Contig1_All, CL7364.Contig1_All, CL7375.Contig1_All, CL7379.Contig1_All, CL7379.Contig2_All, CL7381.Contig1_All, CL7382.Contig1_All, CL7387.Contig1_All, CL7388.Contig1_All, CL7401.Contig1_All, CL7409.Contig1_All, CL7412.Contig2_All, CL7438.Contig1_All, CL7448.Contig1_All, CL7449.Contig1_All, CL7453.Contig1_All, CL7463.Contig1_All, CL7476.Contig1_All, CL7478.Contig1_All, CL7518.Contig1_All, CL752.Contig1_All, CL752.Contig2_All, CL752.Contig3_All, CL752.Contig4_All, CL752.Contig5_All, CL7521.Contig1_All, CL7529.Contig1_All, CL7537.Contig1_All, CL7537.Contig2_All, CL7537.Contig3_All, CL7545.Contig1_All, CL7545.Contig2_All, CL7555.Contig1_All, CL7557.Contig1_All, CL7561.Contig1_All, CL7562.Contig1_All, CL7566.Contig1_All, CL757.Contig1_All, CL757.Contig2_All, CL757.Contig3_All, CL757.Contig4_All, CL7574.Contig1_All, CL7580.Contig1_All, CL7581.Contig1_All, CL7584.Contig1_All, CL759.Contig1_All, CL759.Contig2_All, CL759.Contig3_All, CL759.Contig4_All, CL759.Contig5_All, CL759.Contig6_All, CL7593.Contig1_All, CL7597.Contig2_All, CL761.Contig1_All, CL761.Contig2_All, CL761.Contig3_All, CL761.Contig4_All, CL7615.Contig2_All, CL7617.Contig1_All, CL7621.Contig1_All, CL7627.Contig1_All, CL7655.Contig1_All, CL7655.Contig2_All, CL7664.Contig1_All, CL7670.Contig1_All, CL7678.Contig1_All, CL768.Contig1_All, CL768.Contig2_All, CL768.Contig3_All, CL768.Contig4_All,

CL768.Contig5_All, CL768.Contig6_All, CL7680.Contig1_All, CL77.Contig10_All, CL77.Contig1_All, CL77.Contig2_All, CL77.Contig5_All, CL77.Contig7_All, CL77.Contig8_All, CL77.Contig9_All, CL7700.Contig1_All, CL771.Contig1_All, CL7724.Contig1_All, CL7732.Contig1_All, CL7733.Contig1_All, CL774.Contig1_All, CL774.Contig2_All, CL774.Contig3_All, CL774.Contig4_All, CL7740.Contig1_All, CL7747.Contig1_All, CL7772.Contig1_All, CL7786.Contig1_All, CL7796.Contig1_All, CL7799.Contig1_All, CL7800.Contig1_All, CL7816.Contig1_All, CL7823.Contig1_All, CL7829.Contig1_All, CL7833.Contig1_All, CL7874.Contig1_All, CL7879.Contig1_All, CL7904.Contig1_All, CL7909.Contig1_All, CL7909.Contig2_All, CL7917.Contig1_All, CL7923.Contig1_All, CL7923.Contig2_All, CL793.Contig1_All, CL7941.Contig1_All, CL7959.Contig1_All, CL7961.Contig1_All, CL7965.Contig1_All, CL7974.Contig1_All, CL7978.Contig1_All, CL7983.Contig1_All, CL7994.Contig1_All, CL7997.Contig1_All, CL8001.Contig1_All, CL8003.Contig1_All, CL8008.Contig1_All, CL8014.Contig1_All, CL8030.Contig1_All, CL805.Contig1_All, CL805.Contig2_All, CL805.Contig3_All, CL805.Contig4_All, CL805.Contig5_All, CL8052.Contig1_All, CL8059.Contig1_All, CL8094.Contig1_All, CL8104.Contig1_All, CL812.Contig1_All, CL8132.Contig1_All, CL8132.Contig2_All, CL8138.Contig1_All, CL8159.Contig1_All, CL8161.Contig1_All, CL8165.Contig1_All, CL8172.Contig1_All, CL8197.Contig1_All, CL820.Contig1_All, CL820.Contig2_All, CL8200.Contig1_All, CL8213.Contig1_All, CL8217.Contig1_All, CL8219.Contig2_All, CL8222.Contig1_All, CL8223.Contig1_All, CL8225.Contig1_All, CL8236.Contig1_All, CL8239.Contig1_All, CL8261.Contig1_All, CL8262.Contig1_All, CL8277.Contig1_All, CL8288.Contig1_All, CL829.Contig1_All, CL829.Contig2_All, CL829.Contig3_All, CL8297.Contig1_All, CL8299.Contig1_All, CL8300.Contig1_All, CL8307.Contig1_All, CL8320.Contig1_All, CL8324.Contig1_All, CL8337.Contig1_All, CL8338.Contig1_All, CL8338.Contig2_All, CL8339.Contig1_All, CL8346.Contig1_All, CL8358.Contig1_All, CL8366.Contig1_All, CL837.Contig1_All, CL837.Contig2_All, CL837.Contig3_All, CL837.Contig4_All, CL8375.Contig1_All, CL8381.Contig1_All, CL8384.Contig1_All, CL8386.Contig1_All, CL8390.Contig1_All, CL8393.Contig1_All, CL8398.Contig1_All, CL84.Contig12_All, CL84.Contig14_All, CL84.Contig1_All, CL84.Contig2_All, CL84.Contig7_All, CL84.Contig9_All, CL8407.Contig1_All, CL8423.Contig1_All, CL8437.Contig1_All, CL8439.Contig1_All, CL8449.Contig1_All, CL8452.Contig1_All, CL8468.Contig1_All, CL8468.Contig2_All, CL8487.Contig1_All, CL849.Contig1_All,

			CL849.Contig2_All, CL8491.Contig1_All, CL8491.Contig2_All, CL8494.Contig1_All, CL8496.Contig1_All, CL8499.Contig1_All, CL8505.Contig1_All, CL851.Contig4_All, CL8511.Contig1_All, CL8528.Contig1_All, CL8535.Contig1_All, CL8545.Contig1_All, CL8546.Contig1_All, CL8559.Contig1_All, CL856.Contig1_All, CL856.Contig2_All, CL8562.Contig1_All, CL8564.Contig1_All, CL8587.Contig1_All, CL8588.Contig1_All, CL8600.Contig1_All, CL8608.Contig1_All, CL8608.Contig2_All, CL8638.Contig1_All, CL8639.Contig1_All, CL8639.Contig2_All, CL864.Contig1_All, CL864.Contig3_All, CL8643.Contig1_All, CL8643.Contig2_All, CL8644.Contig1_All, CL8646.Contig1_All, CL8651.Contig1_All, CL8662.Contig1_All, CL8663.Contig1_All, CL8668.Contig1_All, CL8687.Contig1_All, CL8690.Contig1_All, CL8691.Contig1_All, CL8695.Contig1_All, CL8697.Contig1_All, CL8700.Contig1_All, CL8707.Contig1_All, CL8718.Contig1_All, CL8720.Contig1_All, CL8723.Contig1_All, CL8738.Contig1_All, CL8739.Contig1_All, CL8747.Contig1_All, CL8748.Contig1_All, CL8749.Contig1_All, CL8758.Contig1_All, CL876.Contig3_All, CL8762.Contig1_All, CL878.Contig1_All, CL8781.Contig1_All, CL8781.Contig2_All, CL8803.Contig1_All, CL8804.Contig1_All, CL881.Contig1_All, CL881.Contig2_All, CL881.Contig3_All, CL881.Contig4_All, CL8814.Contig1_All, CL8819.Contig1_All, CL8823.Contig1_All, CL8830.Contig1_All, CL8831.Contig1_All, CL8838.Contig1_All, CL8845.Contig1_All, CL8846.Contig1_All, CL8846.Contig2_All, CL8859.Contig1_All, CL8869.Contig1_All, CL8872.Contig1_All, CL8878.Contig1_All, CL8894.Contig1_All, CL8896.Contig1_All, CL8915.Contig1_All, CL8918.Contig1_All, CL8932.Contig1_All, CL8935.Contig1_All, CL894.Contig4_All, CL8943.Contig1_All, CL8945.Contig1_All, CL8949.Contig1_All, CL8959.Contig1_All, CL8964.Contig1_All, CL8972.Contig1_All, CL898.Contig1_All, CL898.Contig2_All, CL8988.Contig1_All, CL9002.Contig1_All, CL9026.Contig1_All, CL9054.Contig1_All, CL9055.Contig1_All, CL9057.Contig1_All, CL908.Contig1_All, CL908.Contig2_All, CL908.Contig3_All, CL908.Contig4_All, CL9085.Contig1_All, CL9093.Contig1_All, CL9095.Contig1_All, CL9104.Contig1_All, CL9104.Contig2_All, CL9106.Contig1_All, CL9109.Contig1_All, CL9122.Contig1_All, CL9129.Contig1_All, CL9150.Contig1_All, CL9151.Contig1_All, CL9164.Contig1_All, CL9165.Contig1_All, CL917.Contig1_All, CL917.Contig2_All, CL917.Contig3_All, CL9172.Contig1_All, CL9174.Contig1_All, CL9184.Contig1_All, CL9190.Contig1_All, CL9192.Contig1_All, CL9195.Contig1_All, CL9200.Contig1_All, CL9202.Contig1_All,

		CL9204.Contig1_All, CL921.Contig1_All, CL921.Contig2_All, CL921.Contig3_All, CL921.Contig4_All, CL921.Contig5_All, CL921.Contig6_All, CL921.Contig7_All, CL921.Contig8_All, CL922.Contig3_All, CL9220.Contig1_All, CL9226.Contig1_All, CL9236.Contig1_All, CL9243.Contig1_All, CL9266.Contig1_All, CL927.Contig1_All, CL927.Contig2_All, CL927.Contig3_All, CL927.Contig4_All, CL927.Contig5_All, CL927.Contig6_All, CL927.Contig7_All, CL927.Contig8_All, CL9270.Contig1_All, CL9275.Contig1_All, CL928.Contig1_All, CL928.Contig2_All, CL928.Contig3_All, CL9291.Contig1_All, CL9291.Contig2_All, CL9302.Contig1_All, CL9303.Contig1_All, CL932.Contig1_All, CL932.Contig2_All, CL932.Contig3_All, CL932.Contig4_All, CL9339.Contig1_All, CL9383.Contig1_All, CL9383.Contig2_All, CL9385.Contig1_All, CL94.Contig2_All, CL94.Contig3_All, CL94.Contig5_All, CL94.Contig6_All, CL94.Contig8_All, CL94.Contig9_All, CL9407.Contig1_All, CL9408.Contig1_All, CL9429.Contig1_All, CL9435.Contig1_All, CL9450.Contig1_All, CL946.Contig1_All, CL946.Contig2_All, CL946.Contig3_All, CL946.Contig4_All, CL9508.Contig1_All, CL9517.Contig1_All, CL9550.Contig1_All, CL9568.Contig1_All, CL9570.Contig1_All, CL9578.Contig1_All, CL9598.Contig1_All, CL9603.Contig1_All, CL9611.Contig1_All, CL9652.Contig1_All, CL9657.Contig1_All, CL9673.Contig1_All, CL9677.Contig1_All, CL968.Contig1_All, CL9682.Contig1_All, CL9688.Contig1_All, CL9723.Contig1_All, CL9724.Contig1_All, CL973.Contig2_All, CL9739.Contig1_All, CL9741.Contig1_All, CL9745.Contig1_All, CL9749.Contig1_All, CL9777.Contig1_All, CL9790.Contig1_All, CL9797.Contig1_All, CL9799.Contig1_All, CL9805.Contig1_All, CL9810.Contig1_All, CL9842.Contig1_All, CL9846.Contig1_All, CL9848.Contig1_All, CL986.Contig1_All, CL986.Contig2_All, CL986.Contig3_All, CL986.Contig4_All, CL986.Contig5_All, CL9882.Contig1_All, CL9882.Contig2_All, CL9921.Contig1_All, CL9935.Contig1_All, CL9938.Contig1_All, CL9947.Contig1_All, CL9950.Contig1_All, CL996.Contig1_All, CL996.Contig2_All, CL996.Contig3_All, CL996.Contig4_All, CL996.Contig5_All, CL9960.Contig1_All, CL998.Contig2_All, CL998.Contig3_All, CL998.Contig4_All, CL9981.Contig1_All, CL9984.Contig1_All, CL9987.Contig1_All, Unigene1017_All, Unigene1020_All, Unigene102_All, Unigene1037_All, Unigene1043_All, Unigene1045_All, Unigene1053_All, Unigene1062_All, Unigene1064_All, Unigene1083_All, Unigene1104_All, Unigene1108_All, Unigene1109_All, Unigene1120_All, Unigene1135_All, Unigene1142_All, Unigene1150_All, Unigene1153_All, Unigene1155_All, Unigene1207_All, Unigene1222_All, Unigene1230_All, Unigene1235_All, Unigene1242_All, Unigene1245_All, Unigene1262_All, Unigene1264_All, Unigene1266_All, Unigene1269_All, Unigene1274_All, Unigene127_All, Unigene1285_All,

		Unigene1294_All, Unigene1329_All, Unigene1335_All, Unigene1386_All, Unigene1401_All, Unigene1416_All, Unigene1419_All, Unigene1421_All, Unigene1434_All, Unigene1441_All, Unigene144_All, Unigene1453_All, Unigene145_All, Unigene1470_All, Unigene1482_All, Unigene1483_All, Unigene1539_All, Unigene1564_All, Unigene1586_All, Unigene1597_All, Unigene1599_All, Unigene1600_All, Unigene1603_All, Unigene1634_All, Unigene1640_All, Unigene1655_All, Unigene1683_All, Unigene1686_All, Unigene1694_All, Unigene1697_All, Unigene1707_All, Unigene1712_All, Unigene1734_All, Unigene1738_All, Unigene173_All, Unigene1762_All, Unigene1773_All, Unigene1774_All, Unigene177_All, Unigene1781_All, Unigene1782_All, Unigene1783_All, Unigene1792_All, Unigene1819_All, Unigene185_All, Unigene1878_All, Unigene1889_All, Unigene1907_All, Unigene1914_All, Unigene1931_All, Unigene1935_All, Unigene193_All, Unigene1957_All, Unigene1978_All, Unigene198_All, Unigene1990_All, Unigene2001_All, Unigene205_All, Unigene2094_All, Unigene2102_All, Unigene2126_All, Unigene2129_All, Unigene2133_All, Unigene2140_All, Unigene2148_All, Unigene2167_All, Unigene2178_All, Unigene2195_All, Unigene2207_All, Unigene2218_All, Unigene2250_All, Unigene2253_All, Unigene2263_All, Unigene2282_All, Unigene2287_All, Unigene2290_All, Unigene2291_All, Unigene2292_All, Unigene229_All, Unigene2302_All, Unigene2308_All, Unigene2338_All, Unigene2340_All, Unigene2341_All, Unigene234_All, Unigene239_All, Unigene2408_All, Unigene2449_All, Unigene2466_All, Unigene2472_All, Unigene2486_All, Unigene2509_All, Unigene2510_All, Unigene252_All, Unigene2530_All, Unigene2543_All, Unigene2549_All, Unigene2550_All, Unigene2552_All, Unigene255_All, Unigene2560_All, Unigene2574_All, Unigene259_All, Unigene2600_All, Unigene2602_All, Unigene2613_All, Unigene2628_All, Unigene2636_All, Unigene2637_All, Unigene2646_All, Unigene2660_All, Unigene2681_All, Unigene2683_All, Unigene2687_All, Unigene2688_All, Unigene2706_All, Unigene2709_All, Unigene2714_All, Unigene2717_All, Unigene2719_All, Unigene2741_All, Unigene278_All, Unigene2797_All, Unigene2826_All, Unigene2829_All, Unigene2836_All, Unigene2839_All, Unigene2869_All, Unigene2872_All, Unigene2873_All, Unigene2876_All, Unigene2880_All, Unigene2881_All, Unigene2884_All, Unigene2888_All, Unigene2921_All, Unigene2961_All, Unigene2962_All, Unigene2963_All, Unigene2964_All, Unigene2966_All, Unigene2970_All, Unigene2974_All, Unigene2994_All, Unigene3023_All, Unigene3029_All, Unigene3041_All, Unigene3055_All, Unigene3064_All, Unigene3071_All, Unigene3075_All, Unigene3077_All, Unigene3079_All, Unigene3094_All, Unigene3101_All, Unigene3102_All, Unigene3120_All, Unigene3138_All, Unigene3151_All, Unigene3156_All, Unigene3163_All, Unigene3179_All,

		Unigene3185_All, Unigene3202_All, Unigene3203_All, Unigene3236_All, Unigene324_All, Unigene325_All, Unigene3275_All, Unigene327_All, Unigene3293_All, Unigene3313_All, Unigene3346_All, Unigene335_All, Unigene3368_All, Unigene3378_All, Unigene337_All, Unigene3397_All, Unigene3423_All, Unigene3426_All, Unigene3427_All, Unigene3432_All, Unigene343_All, Unigene3443_All, Unigene3447_All, Unigene3468_All, Unigene346_All, Unigene3504_All, Unigene350_All, Unigene3522_All, Unigene3548_All, Unigene3563_All, Unigene3566_All, Unigene3611_All, Unigene3637_All, Unigene3638_All, Unigene363_All, Unigene3654_All, Unigene3657_All, Unigene3666_All, Unigene3669_All, Unigene366_All, Unigene3670_All, Unigene3680_All, Unigene3707_All, Unigene3708_All, Unigene3710_All, Unigene3711_All, Unigene3719_All, Unigene3744_All, Unigene3769_All, Unigene3772_All, Unigene3774_All, Unigene3789_All, Unigene3792_All, Unigene3793_All, Unigene380_All, Unigene3870_All, Unigene3918_All, Unigene3943_All, Unigene3947_All, Unigene3960_All, Unigene3980_All, Unigene3983_All, Unigene3997_All, Unigene4000_All, Unigene4007_All, Unigene400_All, Unigene4011_All, Unigene4017_All, Unigene4038_All, Unigene4051_All, Unigene4058_All, Unigene4065_All, Unigene4096_All, Unigene4109_All, Unigene4114_All, Unigene4118_All, Unigene4152_All, Unigene4153_All, Unigene4157_All, Unigene4164_All, Unigene4170_All, Unigene4178_All, Unigene4184_All, Unigene4211_All, Unigene4219_All, Unigene4277_All, Unigene4281_All, Unigene4293_All, Unigene4302_All, Unigene4316_All, Unigene4340_All, Unigene4342_All, Unigene4348_All, Unigene4352_All, Unigene4354_All, Unigene4370_All, Unigene4371_All, Unigene4372_All, Unigene4397_All, Unigene4401_All, Unigene4403_All, Unigene4406_All, Unigene4409_All, Unigene4417_All, Unigene441_All, Unigene4420_All, Unigene4438_All, Unigene4439_All, Unigene4444_All, Unigene4464_All, Unigene4469_All, Unigene4497_All, Unigene4499_All, Unigene4502_All, Unigene4503_All, Unigene4507_All, Unigene4510_All, Unigene4523_All, Unigene4527_All, Unigene4528_All, Unigene4538_All, Unigene4539_All, Unigene4548_All, Unigene4558_All, Unigene4613_All, Unigene4628_All, Unigene462_All, Unigene4633_All, Unigene4640_All, Unigene4646_All, Unigene4669_All, Unigene4676_All, Unigene4679_All, Unigene4680_All, Unigene4685_All, Unigene4687_All, Unigene4688_All, Unigene4727_All, Unigene4732_All, Unigene4741_All, Unigene4745_All, Unigene4755_All, Unigene4773_All, Unigene4775_All, Unigene4790_All, Unigene4805_All, Unigene4810_All, Unigene4811_All, Unigene4818_All, Unigene4843_All, Unigene484_All, Unigene4862_All, Unigene4864_All, Unigene4889_All, Unigene4899_All, Unigene4908_All, Unigene4943_All, Unigene4953_All, Unigene4968_All, Unigene4973_All, Unigene4983_All, Unigene4987_All,

		Unigene4999_All, Unigene5010_All, Unigene5015_All, Unigene5048_All, Unigene5049_All, Unigene5089_All, Unigene5120_All, Unigene5126_All, Unigene5134_All, Unigene5143_All, Unigene515_All, Unigene5184_All, Unigene5188_All, Unigene518_All, Unigene5207_All, Unigene5214_All, Unigene5224_All, Unigene5236_All, Unigene5238_All, Unigene523_All, Unigene5242_All, Unigene5261_All, Unigene5262_All, Unigene5264_All, Unigene5279_All, Unigene5280_All, Unigene5292_All, Unigene5302_All, Unigene5316_All, Unigene5322_All, Unigene5329_All, Unigene5332_All, Unigene5339_All, Unigene5357_All, Unigene5362_All, Unigene5364_All, Unigene5373_All, Unigene5389_All, Unigene5410_All, Unigene5413_All, Unigene5436_All, Unigene5437_All, Unigene5438_All, Unigene544_All, Unigene5473_All, Unigene5474_All, Unigene5496_All, Unigene5506_All, Unigene5509_All, Unigene5511_All, Unigene5518_All, Unigene5522_All, Unigene5525_All, Unigene560_All, Unigene576_All, Unigene579_All, Unigene606_All, Unigene607_All, Unigene622_All, Unigene644_All, Unigene652_All, Unigene657_All, Unigene664_All, Unigene666_All, Unigene683_All, Unigene705_All, Unigene708_All, Unigene71_All, Unigene734_All, Unigene735_All, Unigene760_All, Unigene761_All, Unigene790_All, Unigene800_All, Unigene828_All, Unigene831_All, Unigene832_All, Unigene865_All, Unigene874_All, Unigene87_All, Unigene882_All, Unigene895_All, Unigene915_All, Unigene920_All, Unigene930_All, Unigene932_All, Unigene938_All, Unigene939_All, Unigene961_All, Unigene975_All, Unigene990_All
2		CL10.Contig14_All, CL10.Contig30_All, CL10004.Contig1_All, CL10008.Contig1_All, CL10026.Contig1_All, CL10035.Contig1_All, CL10038.Contig1_All, CL10048.Contig1_All, CL10055.Contig1_All, CL10068.Contig1_All, CL10072.Contig2_All, CL10073.Contig1_All, CL10104.Contig1_All, CL10108.Contig1_All, CL10109.Contig1_All, CL10128.Contig1_All, CL10136.Contig1_All, CL10146.Contig1_All, CL10151.Contig1_All, CL10162.Contig1_All, CL10171.Contig1_All, CL10183.Contig1_All, CL102.Contig10_All, CL102.Contig1_All, CL102.Contig2_All, CL102.Contig3_All, CL102.Contig4_All, CL102.Contig5_All, CL102.Contig6_All, CL102.Contig7_All, CL102.Contig8_All, CL102.Contig9_All, CL10202.Contig1_All, CL10214.Contig1_All, CL10215.Contig1_All, CL10216.Contig1_All, CL10217.Contig1_All, CL10224.Contig1_All, CL10230.Contig1_All, CL10238.Contig1_All, CL10240.Contig1_All, CL10242.Contig1_All, CL10266.Contig1_All, CL10278.Contig1_All, CL1029.Contig2_All, CL10318.Contig1_All, CL10334.Contig1_All, CL10334.Contig2_All, CL10353.Contig1_All, CL10366.Contig1_All,

	Biosynthesis of secondary	CL10384.Contig1_All, CL10397.Contig1_All, CL10426.Contig1_All, CL10433.Contig1_All, CL10449.Contig1_All, CL10453.Contig1_All, CL10453.Contig2_All, CL10458.Contig1_All, CL10469.Contig1_All, CL10472.Contig1_All, CL10477.Contig1_All, CL1048.Contig1_All, CL1051.Contig1_All, CL1051.Contig2_All, CL10551.Contig1_All, CL10556.Contig1_All, CL10568.Contig1_All, CL10569.Contig1_All, CL10571.Contig1_All, CL10582.Contig1_All, CL10585.Contig1_All, CL10592.Contig1_All, CL10594.Contig1_All, CL10600.Contig1_All, CL10623.Contig1_All, CL10632.Contig1_All, CL10637.Contig1_All, CL10650.Contig1_All, CL10655.Contig1_All, CL10668.Contig1_All, CL10671.Contig1_All, CL10672.Contig1_All, CL10687.Contig1_All, CL10727.Contig1_All, CL10740.Contig1_All, CL10753.Contig1_All, CL1077.Contig1_All, CL10775.Contig1_All, CL10788.Contig1_All, CL10814.Contig1_All, CL10825.Contig1_All, CL10834.Contig1_All, CL10840.Contig1_All, CL10841.Contig1_All, CL10846.Contig1_All, CL10846.Contig2_All, CL10856.Contig1_All, CL1086.Contig1_All, CL1086.Contig2_All, CL10870.Contig1_All, CL10877.Contig1_All, CL10883.Contig1_All, CL10924.Contig1_All, CL10951.Contig1_All, CL10992.Contig1_All, CL10994.Contig1_All, CL11.Contig17_All, CL11013.Contig1_All, CL11017.Contig1_All, CL11021.Contig1_All, CL11032.Contig1_All, CL11047.Contig1_All, CL11064.Contig1_All, CL11070.Contig1_All, CL11080.Contig1_All, CL11090.Contig1_All, CL11093.Contig1_All, CL11111.Contig1_All, CL11116.Contig1_All, CL11117.Contig1_All, CL11129.Contig1_All, CL11130.Contig1_All, CL11132.Contig1_All, CL11135.Contig1_All, CL11140.Contig1_All, CL11163.Contig1_All, CL11178.Contig1_All, CL11203.Contig1_All, CL11224.Contig1_All, CL11229.Contig1_All, CL11247.Contig1_All, CL11256.Contig1_All, CL11271.Contig1_All, CL11277.Contig1_All, CL11286.Contig1_All, CL11320.Contig1_All, CL11330.Contig1_All, CL11348.Contig1_All, CL11351.Contig1_All, CL11370.Contig1_All, CL11396.Contig1_All, CL11401.Contig1_All, CL11461.Contig1_All, CL11513.Contig1_All, CL11524.Contig1_All, CL11527.Contig1_All, CL11535.Contig1_All, CL11542.Contig1_All, CL11562.Contig1_All, CL11570.Contig1_All, CL11595.Contig1_All, CL1162.Contig1_All, CL11625.Contig1_All, CL11632.Contig1_All, CL11635.Contig1_All, CL1165.Contig1_All, CL1165.Contig2_All, CL1165.Contig3_All,

	metabolites (no map in kegg	CL1165.Contig4_All, CL11655.Contig1_All, CL1166.Contig1_All, CL1166.Contig2_All, CL1166.Contig3_All, CL11669.Contig1_All, CL11710.Contig1_All, CL11718.Contig1_All, CL11738.Contig1_All, CL11754.Contig1_All, CL11759.Contig1_All, CL11762.Contig1_All, CL11779.Contig1_All, CL11785.Contig1_All, CL11811.Contig1_All, CL11816.Contig1_All, CL11819.Contig1_All, CL11827.Contig1_All, CL11835.Contig1_All, CL11840.Contig1_All, CL11843.Contig1_All, CL11866.Contig1_All, CL11877.Contig1_All, CL11878.Contig1_All, CL11900.Contig1_All, CL11926.Contig1_All, CL11940.Contig1_All, CL11944.Contig1_All, CL11966.Contig1_All, CL1197.Contig1_All, CL1197.Contig2_All, CL11985.Contig1_All, CL11994.Contig1_All, CL12037.Contig1_All, CL12059.Contig1_All, CL12067.Contig1_All, CL12074.Contig1_All, CL12133.Contig1_All, CL12156.Contig1_All, CL12183.Contig1_All, CL12184.Contig1_All, CL12185.Contig1_All, CL12194.Contig1_All, CL12202.Contig1_All, CL12207.Contig1_All, CL12218.Contig1_All, CL12232.Contig1_All, CL12238.Contig1_All, CL12239.Contig1_All, CL12247.Contig1_All, CL12250.Contig1_All, CL12261.Contig1_All, CL1227.Contig1_All, CL12277.Contig1_All, CL1228.Contig1_All, CL12281.Contig1_All, CL12294.Contig1_All, CL12297.Contig1_All, CL12301.Contig1_All, CL12307.Contig1_All, CL12316.Contig1_All, CL12322.Contig1_All, CL12334.Contig1_All, CL1234.Contig1_All, CL1234.Contig2_All, CL12340.Contig1_All, CL12345.Contig1_All, CL12346.Contig1_All, CL12364.Contig1_All, CL12370.Contig1_All, CL12373.Contig1_All, CL12376.Contig1_All, CL12389.Contig1_All, CL12396.Contig1_All, CL12410.Contig1_All, CL12429.Contig1_All, CL12443.Contig1_All, CL12447.Contig1_All, CL1246.Contig1_All, CL12466.Contig1_All, CL12470.Contig1_All, CL12487.Contig1_All, CL12496.Contig1_All, CL12498.Contig1_All, CL12514.Contig1_All, CL12523.Contig1_All, CL12532.Contig1_All, CL12550.Contig1_All, CL1256.Contig1_All, CL1256.Contig2_All, CL12575.Contig1_All, CL12611.Contig1_All, CL12612.Contig1_All, CL12640.Contig1_All, CL12653.Contig1_All, CL1266.Contig1_All, CL12667.Contig1_All, CL12674.Contig1_All, CL1269.Contig1_All, CL1269.Contig3_All, CL1269.Contig4_All, CL1269.Contig5_All, CL12692.Contig1_All, CL12698.Contig1_All, CL12702.Contig1_All, CL12705.Contig1_All, CL12714.Contig1_All, CL12720.Contig1_All,

	database)	CL12722.Contig1_All, CL12743.Contig1_All, CL12751.Contig1_All, CL12753.Contig1_All, CL12762.Contig1_All, CL1277.Contig2_All, CL12777.Contig1_All, CL12780.Contig1_All, CL12810.Contig1_All, CL12818.Contig1_All, CL12820.Contig1_All, CL12842.Contig1_All, CL12865.Contig1_All, CL12868.Contig1_All, CL12875.Contig1_All, CL12888.Contig1_All, CL12898.Contig1_All, CL12906.Contig1_All, CL12909.Contig1_All, CL12911.Contig1_All, CL12912.Contig1_All, CL12917.Contig1_All, CL12925.Contig1_All, CL12928.Contig1_All, CL12942.Contig1_All, CL12949.Contig1_All, CL12957.Contig1_All, CL12981.Contig1_All, CL12985.Contig1_All, CL12993.Contig1_All, CL13001.Contig1_All, CL13016.Contig1_All, CL13018.Contig1_All, CL13019.Contig1_All, CL13023.Contig1_All, CL13024.Contig1_All, CL13054.Contig1_All, CL13068.Contig1_All, CL13071.Contig1_All, CL13083.Contig1_All, CL13104.Contig1_All, CL13139.Contig1_All, CL13152.Contig1_All, CL13172.Contig1_All, CL13175.Contig1_All, CL13179.Contig1_All, CL13244.Contig1_All, CL13246.Contig1_All, CL13253.Contig1_All, CL13256.Contig1_All, CL13269.Contig1_All, CL13277.Contig1_All, CL13286.Contig1_All, CL13321.Contig1_All, CL13326.Contig1_All, CL13338.Contig1_All, CL13347.Contig1_All, CL13385.Contig1_All, CL1339.Contig1_All, CL1339.Contig2_All, CL1339.Contig3_All, CL1339.Contig4_All, CL13408.Contig1_All, CL13440.Contig1_All, CL13444.Contig1_All, CL13445.Contig1_All, CL13451.Contig1_All, CL1346.Contig1_All, CL1346.Contig2_All, CL1346.Contig3_All, CL1346.Contig4_All, CL13475.Contig1_All, CL13486.Contig1_All, CL13490.Contig1_All, CL13492.Contig1_All, CL13520.Contig1_All, CL13524.Contig1_All, CL13530.Contig1_All, CL13535.Contig1_All, CL13538.Contig1_All, CL13540.Contig1_All, CL13548.Contig1_All, CL13549.Contig1_All, CL13569.Contig1_All, CL13574.Contig1_All, CL13575.Contig1_All, CL13586.Contig1_All, CL13593.Contig1_All, CL13594.Contig1_All, CL13600.Contig1_All, CL13634.Contig1_All, CL13635.Contig1_All, CL13661.Contig1_All, CL1369.Contig1_All, CL1369.Contig2_All, CL1369.Contig3_All, CL13721.Contig1_All, CL13770.Contig1_All, CL13835.Contig1_All, CL1384.Contig1_All, CL139.Contig6_All, CL13923.Contig1_All, CL13926.Contig1_All, CL13936.Contig1_All, CL13956.Contig1_All, CL13980.Contig1_All, CL13999.Contig1_All, CL13999.Contig2_All,

			CL14038.Contig1_All, CL1407.Contig1_All, CL1407.Contig2_All, CL1407.Contig3_All, CL1408.Contig1_All, CL1408.Contig2_All, CL1408.Contig3_All, CL1408.Contig4_All, CL1409.Contig1_All, CL1409.Contig2_All, CL1409.Contig3_All, CL1409.Contig4_All, CL1409.Contig5_All, CL1412.Contig1_All, CL1412.Contig2_All, CL1418.Contig1_All, CL14182.Contig1_All, CL14213.Contig1_All, CL14294.Contig1_All, CL143.Contig1_All, CL143.Contig4_All, CL14311.Contig1_All, CL14439.Contig1_All, CL14476.Contig1_All, CL14476.Contig2_All, CL14522.Contig1_All, CL14588.Contig1_All, CL1460.Contig2_All, CL14609.Contig1_All, CL14669.Contig1_All, CL1469.Contig3_All, CL14695.Contig1_All, CL14726.Contig1_All, CL14758.Contig1_All, CL14782.Contig1_All, CL14802.Contig1_All, CL14824.Contig1_All, CL14842.Contig1_All, CL14853.Contig1_All, CL14933.Contig1_All, CL14941.Contig1_All, CL14988.Contig1_All, CL150.Contig7_All, CL1502.Contig1_All, CL15125.Contig1_All, CL15140.Contig1_All, CL15166.Contig1_All, CL15175.Contig1_All, CL1523.Contig1_All, CL15253.Contig1_All, CL15275.Contig1_All, CL1529.Contig1_All, CL1529.Contig2_All, CL1529.Contig3_All, CL1529.Contig4_All, CL1529.Contig5_All, CL15303.Contig1_All, CL15336.Contig1_All, CL15367.Contig1_All, CL1539.Contig1_All, CL1539.Contig2_All, CL1544.Contig1_All, CL15447.Contig1_All, CL1545.Contig1_All, CL1545.Contig2_All, CL15538.Contig1_All, CL1559.Contig1_All, CL1559.Contig2_All, CL156.Contig1_All, CL156.Contig2_All, CL156.Contig3_All, CL156.Contig4_All, CL156.Contig5_All, CL156.Contig6_All, CL156.Contig7_All, CL156.Contig8_All, CL156.Contig9_All, CL15602.Contig1_All, CL15606.Contig1_All, CL1562.Contig1_All, CL1562.Contig2_All, CL1562.Contig3_All, CL1562.Contig4_All, CL15623.Contig1_All, CL15629.Contig1_All, CL15652.Contig1_All, CL15670.Contig1_All, CL1568.Contig1_All, CL1568.Contig2_All, CL1568.Contig3_All, CL1568.Contig4_All, CL15730.Contig1_All, CL15730.Contig2_All, CL15733.Contig1_All, CL15754.Contig1_All, CL15778.Contig1_All, CL15799.Contig1_All, CL15813.Contig1_All, CL15857.Contig1_All, CL15867.Contig1_All, CL1587.Contig1_All, CL15902.Contig1_All, CL1591.Contig1_All, CL1591.Contig2_All, CL1591.Contig3_All, CL1591.Contig4_All, CL1591.Contig5_All, CL1599.Contig1_All, CL16000.Contig1_All, CL16038.Contig1_All, CL16054.Contig1_All, CL16056.Contig1_All,

		CL16109.Contig1_All, CL16124.Contig1_All, CL16130.Contig1_All, CL16172.Contig1_All, CL16173.Contig1_All, CL16186.Contig1_All, CL16203.Contig1_All, CL16214.Contig1_All, CL16239.Contig1_All, CL16268.Contig1_All, CL16274.Contig1_All, CL16277.Contig1_All, CL16277.Contig2_All, CL16291.Contig1_All, CL16298.Contig1_All, CL16347.Contig1_All, CL16358.Contig1_All, CL16372.Contig1_All, CL16414.Contig1_All, CL1642.Contig1_All, CL16430.Contig1_All, CL1645.Contig1_All, CL16457.Contig1_All, CL16464.Contig1_All, CL16473.Contig1_All, CL16502.Contig1_All, CL16534.Contig1_All, CL16543.Contig1_All, CL16555.Contig1_All, CL16556.Contig1_All, CL16561.Contig1_All, CL16566.Contig1_All, CL16603.Contig1_All, CL16605.Contig1_All, CL16640.Contig1_All, CL16643.Contig1_All, CL16661.Contig1_All, CL16675.Contig1_All, CL16676.Contig1_All, CL16719.Contig1_All, CL1672.Contig1_All, CL16751.Contig1_All, CL16785.Contig1_All, CL16810.Contig1_All, CL16833.Contig1_All, CL16840.Contig1_All, CL16871.Contig1_All, CL16913.Contig1_All, CL16918.Contig1_All, CL16932.Contig1_All, CL16998.Contig1_All, CL17004.Contig1_All, CL17059.Contig1_All, CL17086.Contig1_All, CL17086.Contig2_All, CL17189.Contig1_All, CL17233.Contig1_All, CL1728.Contig1_All, CL1728.Contig2_All, CL1729.Contig1_All, CL1729.Contig2_All, CL1729.Contig3_All, CL1729.Contig4_All, CL17291.Contig1_All, CL17291.Contig2_All, CL17313.Contig1_All, CL17356.Contig1_All, CL17379.Contig1_All, CL17409.Contig1_All, CL17419.Contig1_All, CL17441.Contig1_All, CL17459.Contig1_All, CL17459.Contig2_All, CL17466.Contig1_All, CL17486.Contig1_All, CL1749.Contig1_All, CL17498.Contig1_All, CL17539.Contig1_All, CL17542.Contig1_All, CL17547.Contig1_All, CL17597.Contig1_All, CL17597.Contig2_All, CL17637.Contig1_All, CL17667.Contig1_All, CL17684.Contig1_All, CL17689.Contig1_All, CL17693.Contig1_All, CL17699.Contig1_All, CL17719.Contig1_All, CL17743.Contig1_All, CL17764.Contig1_All, CL17793.Contig1_All, CL17858.Contig1_All, CL17914.Contig1_All, CL17921.Contig1_All, CL17962.Contig1_All, CL17967.Contig1_All, CL17992.Contig1_All, CL180.Contig2_All, CL180.Contig4_All, CL180.Contig7_All, CL18054.Contig1_All, CL18061.Contig1_All, CL18117.Contig1_All, CL18144.Contig1_All, CL18183.Contig1_All, CL18226.Contig1_All, CL18244.Contig1_All, CL18250.Contig1_All,

CL18268.Contig1_All, CL18320.Contig1_All,
CL18330.Contig1_All, CL18342.Contig1_All,
CL18360.Contig1_All, CL18381.Contig1_All,
CL18418.Contig1_All, CL18450.Contig1_All,
CL1847.Contig1_All, CL18472.Contig1_All,
CL18491.Contig1_All, CL18532.Contig1_All,
CL18544.Contig1_All, CL1855.Contig3_All,
CL1865.Contig1_All, CL18669.Contig1_All,
CL1874.Contig1_All, CL1874.Contig2_All,
CL1874.Contig3_All, CL1874.Contig4_All,
CL1881.Contig1_All, CL1881.Contig2_All,
CL1881.Contig3_All, CL18822.Contig1_All,
CL18836.Contig1_All, CL18841.Contig1_All,
CL18869.Contig1_All, CL18986.Contig1_All,
CL18999.Contig1_All, CL19021.Contig1_All,
CL19037.Contig1_All, CL19059.Contig1_All,
CL19070.Contig1_All, CL19111.Contig1_All,
CL19121.Contig1_All, CL1916.Contig1_All,
CL1919.Contig1_All, CL19200.Contig1_All,
CL19263.Contig1_All, CL19281.Contig1_All,
CL19283.Contig1_All, CL19286.Contig1_All,
CL19381.Contig1_All, CL19398.Contig1_All,
CL19553.Contig1_All, CL19564.Contig1_All,
CL19577.Contig1_All, CL19616.Contig1_All,
CL19619.Contig1_All, CL1968.Contig1_All,
CL19754.Contig1_All, CL19779.Contig1_All,
CL19792.Contig1_All, CL198.Contig1_All, CL198.Contig2_All,
CL198.Contig3_All, CL198.Contig4_All, CL198.Contig5_All,
CL198.Contig6_All, CL198.Contig7_All, CL198.Contig8_All,
CL198.Contig9_All, CL1989.Contig2_All,
CL19904.Contig1_All, CL19934.Contig1_All,
CL1997.Contig1_All, CL1997.Contig2_All,
CL1997.Contig3_All, CL2.Contig1_All, CL2.Contig22_All,
CL2.Contig25_All, CL2.Contig35_All, CL20001.Contig1_All,
CL20016.Contig1_All, CL20025.Contig1_All,
CL2003.Contig1_All, CL2003.Contig2_All,
CL2003.Contig3_All, CL2003.Contig4_All,
CL2005.Contig1_All, CL20052.Contig1_All,
CL20081.Contig1_All, CL20094.Contig1_All,
CL201.Contig5_All, CL20125.Contig1_All,
CL20154.Contig1_All, CL20175.Contig1_All,
CL20187.Contig1_All, CL20240.Contig1_All,
CL2030.Contig1_All, CL20321.Contig1_All,
CL20332.Contig1_All, CL20335.Contig1_All,
CL20345.Contig1_All, CL2041.Contig1_All,
CL2041.Contig2_All, CL2043.Contig1_All,
CL20473.Contig1_All, CL20484.Contig1_All,
CL2049.Contig2_All, CL2049.Contig4_All,
CL20518.Contig1_All, CL2053.Contig1_All,
CL2053.Contig2_All, CL20571.Contig1_All,
CL20574.Contig1_All, CL20630.Contig1_All,
CL20673.Contig1_All, CL20717.Contig1_All,
CL20773.Contig1_All, CL20774.Contig1_All,
CL20788.Contig1_All, CL20798.Contig1_All,

		CL20832.Contig1_All, CL20859.Contig1_All, CL20874.Contig1_All, CL20875.Contig1_All, CL20943.Contig1_All, CL20987.Contig1_All, CL2102.Contig1_All, CL21022.Contig1_All, CL21049.Contig1_All, CL21051.Contig1_All, CL21053.Contig1_All, CL21082.Contig1_All, CL21086.Contig1_All, CL21133.Contig1_All, CL2115.Contig1_All, CL2118.Contig2_All, CL21201.Contig1_All, CL21241.Contig1_All, CL21272.Contig1_All, CL21286.Contig1_All, CL21299.Contig1_All, CL213.Contig1_All, CL2130.Contig1_All, CL21307.Contig1_All, CL21372.Contig1_All, CL2143.Contig1_All, CL21454.Contig1_All, CL21511.Contig1_All, CL21539.Contig1_All, CL21565.Contig1_All, CL216.Contig1_All, CL216.Contig2_All, CL216.Contig3_All, CL216.Contig4_All, CL216.Contig5_All, CL21613.Contig1_All, CL21671.Contig1_All, CL21674.Contig1_All, CL21692.Contig1_All, CL21700.Contig1_All, CL21740.Contig1_All, CL21741.Contig1_All, CL21780.Contig1_All, CL21826.Contig1_All, CL2185.Contig1_All, CL2185.Contig2_All, CL21853.Contig1_All, CL21901.Contig1_All, CL21909.Contig1_All, CL21921.Contig1_All, CL21929.Contig1_All, CL21945.Contig1_All, CL21987.Contig1_All, CL22002.Contig1_All, CL2205.Contig1_All, CL22071.Contig1_All, CL22077.Contig1_All, CL22098.Contig1_All, CL22102.Contig1_All, CL2212.Contig1_All, CL2212.Contig2_All, CL22167.Contig1_All, CL2221.Contig1_All, CL2221.Contig2_All, CL22250.Contig1_All, CL2226.Contig1_All, CL2227.Contig1_All, CL22294.Contig1_All, CL22305.Contig1_All, CL22352.Contig1_All, CL22366.Contig1_All, CL22411.Contig1_All, CL2245.Contig1_All, CL2245.Contig2_All, CL22454.Contig1_All, CL22464.Contig1_All, CL22484.Contig1_All, CL22514.Contig1_All, CL22545.Contig1_All, CL22561.Contig1_All, CL22573.Contig1_All, CL22599.Contig1_All, CL22601.Contig1_All, CL2262.Contig1_All, CL2262.Contig2_All, CL2262.Contig3_All, CL2262.Contig4_All, CL22641.Contig1_All, CL22652.Contig1_All, CL22703.Contig1_All, CL22751.Contig1_All, CL22771.Contig1_All, CL22776.Contig1_All, CL22879.Contig1_All, CL22885.Contig1_All, CL22926.Contig1_All, CL22966.Contig1_All, CL23002.Contig1_All, CL23027.Contig1_All, CL23042.Contig1_All, CL23047.Contig1_All, CL23059.Contig1_All, CL2306.Contig1_All, CL23117.Contig1_All, CL23127.Contig1_All, CL23161.Contig1_All, CL2323.Contig1_All, CL2323.Contig2_All, CL23231.Contig1_All, CL23275.Contig1_All,

			CL23294.Contig1_All, CL23297.Contig1_All, CL233.Contig2_All, CL233.Contig3_All, CL233.Contig5_All, CL23320.Contig1_All, CL23330.Contig1_All, CL23347.Contig1_All, CL23369.Contig1_All, CL23375.Contig1_All, CL23391.Contig1_All, CL234.Contig5_All, CL234.Contig6_All, CL23445.Contig1_All, CL23475.Contig1_All, CL23525.Contig1_All, CL23526.Contig1_All, CL23582.Contig1_All, CL2359.Contig1_All, CL23611.Contig1_All, CL23615.Contig1_All, CL23687.Contig1_All, CL23799.Contig1_All, CL2385.Contig1_All, CL23911.Contig1_All, CL23940.Contig1_All, CL23945.Contig1_All, CL2398.Contig1_All, CL23997.Contig1_All, CL24022.Contig1_All, CL24032.Contig1_All, CL24033.Contig1_All, CL24036.Contig1_All, CL24040.Contig1_All, CL24077.Contig1_All, CL24081.Contig1_All, CL24088.Contig1_All, CL24115.Contig1_All, CL2414.Contig1_All, CL24211.Contig1_All, CL24249.Contig1_All, CL24287.Contig1_All, CL24379.Contig1_All, CL2439.Contig1_All, CL2439.Contig2_All, CL24469.Contig1_All, CL24508.Contig1_All, CL2455.Contig1_All, CL24579.Contig1_All, CL24604.Contig1_All, CL24605.Contig1_All, CL2461.Contig1_All, CL2461.Contig2_All, CL2461.Contig3_All, CL2461.Contig4_All, CL24617.Contig1_All, CL2468.Contig1_All, CL24683.Contig1_All, CL24764.Contig1_All, CL2483.Contig1_All, CL2485.Contig1_All, CL2490.Contig1_All, CL24900.Contig1_All, CL2498.Contig1_All, CL24981.Contig1_All, CL250.Contig1_All, CL250.Contig2_All, CL250.Contig3_All, CL250.Contig4_All, CL250.Contig5_All, CL250.Contig6_All, CL25097.Contig1_All, CL25126.Contig1_All, CL25142.Contig1_All, CL2516.Contig1_All, CL25160.Contig1_All, CL25208.Contig1_All, CL25210.Contig1_All, CL25282.Contig1_All, CL2531.Contig1_All, CL2531.Contig4_All, CL2531.Contig5_All, CL2531.Contig6_All, CL2531.Contig7_All, CL2536.Contig1_All, CL2536.Contig2_All, CL25407.Contig1_All, CL25414.Contig1_All, CL25456.Contig1_All, CL2546.Contig1_All, CL25490.Contig1_All, CL25514.Contig1_All, CL2554.Contig1_All, CL25555.Contig1_All, CL25556.Contig1_All, CL25567.Contig1_All, CL25606.Contig1_All, CL25612.Contig1_All, CL25641.Contig1_All, CL25658.Contig1_All, CL25675.Contig1_All, CL25689.Contig1_All, CL257.Contig1_All, CL257.Contig2_All, CL25792.Contig1_All, CL25793.Contig1_All, CL25841.Contig1_All, CL25842.Contig1_All, CL25890.Contig1_All, CL2594.Contig1_All, CL25992.Contig1_All, CL25996.Contig1_All, CL26021.Contig1_All,

			CL26066.Contig1_All, CL26096.Contig1_All, CL26121.Contig1_All, CL262.Contig1_All, CL262.Contig2_All, CL262.Contig3_All, CL262.Contig4_All, CL262.Contig5_All, CL262.Contig6_All, CL262.Contig7_All, CL26226.Contig1_All, CL26257.Contig1_All, CL26273.Contig1_All, CL26274.Contig1_All, CL26299.Contig1_All, CL264.Contig1_All, CL264.Contig2_All, CL264.Contig3_All, CL264.Contig4_All, CL264.Contig5_All, CL264.Contig6_All, CL264.Contig7_All, CL2640.Contig1_All, CL2643.Contig1_All, CL2643.Contig2_All, CL2643.Contig3_All, CL26484.Contig1_All, CL26496.Contig1_All, CL26522.Contig1_All, CL2655.Contig1_All, CL26560.Contig1_All, CL26575.Contig1_All, CL266.Contig1_All, CL266.Contig2_All, CL266.Contig3_All, CL266.Contig4_All, CL266.Contig5_All, CL266.Contig6_All, CL26676.Contig1_All, CL26700.Contig1_All, CL26701.Contig1_All, CL26736.Contig1_All, CL2674.Contig1_All, CL26742.Contig1_All, CL26882.Contig1_All, CL26896.Contig1_All, CL26929.Contig1_All, CL26958.Contig1_All, CL26967.Contig1_All, CL27025.Contig1_All, CL27033.Contig1_All, CL27064.Contig1_All, CL27097.Contig1_All, CL271.Contig1_All, CL271.Contig7_All, CL271.Contig8_All, CL27120.Contig1_All, CL27126.Contig1_All, CL27255.Contig1_All, CL27261.Contig1_All, CL27264.Contig1_All, CL27289.Contig1_All, CL2729.Contig1_All, CL273.Contig6_All, CL27307.Contig1_All, CL27308.Contig1_All, CL27352.Contig1_All, CL27370.Contig1_All, CL27416.Contig1_All, CL27439.Contig1_All, CL2745.Contig1_All, CL2745.Contig2_All, CL275.Contig1_All, CL275.Contig2_All, CL2761.Contig1_All, CL2762.Contig1_All, CL27706.Contig1_All, CL27714.Contig1_All, CL278.Contig1_All, CL278.Contig2_All, CL278.Contig6_All, CL278.Contig7_All, CL27806.Contig1_All, CL27816.Contig1_All, CL27887.Contig1_All, CL27926.Contig1_All, CL2794.Contig1_All, CL27973.Contig1_All, CL281.Contig1_All, CL281.Contig2_All, CL281.Contig3_All, CL281.Contig4_All, CL281.Contig5_All, CL281.Contig6_All, CL281.Contig7_All, CL28135.Contig1_All, CL2874.Contig1_All, CL2874.Contig2_All, CL2876.Contig1_All, CL2879.Contig1_All, CL2880.Contig1_All, CL2902.Contig1_All, CL2929.Contig1_All, CL2942.Contig2_All, CL2942.Contig3_All, CL2950.Contig1_All, CL2958.Contig1_All, CL2958.Contig2_All, CL2980.Contig1_All, CL2991.Contig1_All, CL2991.Contig2_All, CL2991.Contig3_All, CL2995.Contig1_All, CL2995.Contig2_All, CL3041.Contig1_All, CL3049.Contig1_All, CL3057.Contig1_All, CL3062.Contig2_All, CL3076.Contig1_All, CL3083.Contig1_All, CL311.Contig1_All, CL3131.Contig2_All, CL3143.Contig1_All,

			CL3143.Contig2_All, CL3152.Contig1_All, CL3170.Contig1_All, CL3176.Contig1_All, CL3191.Contig1_All, CL32.Contig12_All, CL3225.Contig1_All, CL3225.Contig3_All, CL3226.Contig1_All, CL323.Contig1_All, CL323.Contig2_All, CL323.Contig3_All, CL323.Contig4_All, CL3230.Contig1_All, CL3246.Contig1_All, CL3246.Contig2_All, CL3246.Contig3_All, CL3246.Contig4_All, CL3307.Contig1_All, CL3310.Contig1_All, CL3314.Contig1_All, CL3314.Contig2_All, CL3321.Contig1_All, CL3321.Contig2_All, CL3321.Contig3_All, CL3342.Contig1_All, CL3351.Contig1_All, CL3408.Contig1_All, CL3408.Contig2_All, CL3408.Contig3_All, CL3413.Contig1_All, CL3413.Contig2_All, CL3444.Contig1_All, CL3445.Contig1_All, CL3445.Contig2_All, CL3445.Contig3_All, CL3448.Contig1_All, CL3448.Contig2_All, CL3455.Contig1_All, CL3457.Contig1_All, CL3473.Contig1_All, CL3476.Contig1_All, CL3476.Contig2_All, CL3481.Contig1_All, CL3481.Contig2_All, CL3481.Contig3_All, CL349.Contig1_All, CL349.Contig2_All, CL349.Contig3_All, CL349.Contig5_All, CL3494.Contig1_All, CL3505.Contig1_All, CL3528.Contig1_All, CL353.Contig1_All, CL353.Contig2_All, CL353.Contig3_All, CL353.Contig4_All, CL353.Contig5_All, CL3532.Contig1_All, CL3532.Contig2_All, CL3532.Contig3_All, CL3545.Contig1_All, CL3581.Contig1_All, CL3581.Contig2_All, CL3581.Contig3_All, CL3581.Contig4_All, CL3587.Contig1_All, CL3590.Contig1_All, CL3610.Contig1_All, CL3618.Contig1_All, CL3629.Contig1_All, CL3673.Contig1_All, CL3734.Contig1_All, CL3740.Contig1_All, CL3740.Contig2_All, CL3777.Contig1_All, CL3788.Contig1_All, CL38.Contig10_All, CL38.Contig13_All, CL38.Contig8_All, CL3803.Contig1_All, CL3804.Contig1_All, CL3804.Contig2_All, CL3805.Contig1_All, CL3831.Contig1_All, CL3838.Contig1_All, CL3838.Contig2_All, CL3841.Contig1_All, CL3846.Contig1_All, CL3914.Contig1_All, CL3915.Contig1_All, CL3916.Contig1_All, CL3916.Contig2_All, CL3942.Contig1_All, CL3952.Contig1_All, CL3955.Contig1_All, CL3984.Contig1_All, CL3988.Contig1_All, CL3991.Contig1_All, CL3991.Contig2_All, CL3992.Contig1_All, CL4.Contig17_All, CL4.Contig25_All, CL4.Contig5_All, CL40.Contig10_All, CL40.Contig11_All, CL40.Contig1_All, CL40.Contig2_All, CL40.Contig3_All, CL40.Contig4_All, CL40.Contig5_All, CL40.Contig6_All, CL40.Contig7_All, CL40.Contig8_All, CL40.Contig9_All, CL4024.Contig1_All, CL4030.Contig1_All, CL4055.Contig1_All, CL4095.Contig1_All, CL4101.Contig1_All, CL4101.Contig2_All, CL412.Contig1_All, CL412.Contig2_All, CL412.Contig3_All, CL4120.Contig1_All,

		CL4121.Contig1_All, CL4129.Contig1_All, CL4140.Contig1_All, CL4141.Contig1_All, CL4161.Contig1_All, CL4168.Contig1_All, CL4174.Contig1_All, CL4177.Contig1_All, CL4183.Contig1_All, CL4224.Contig1_All, CL4280.Contig1_All, CL4299.Contig1_All, CL4299.Contig2_All, CL4306.Contig1_All, CL4306.Contig2_All, CL4312.Contig1_All, CL4312.Contig2_All, CL4316.Contig1_All, CL4331.Contig1_All, CL437.Contig1_All, CL437.Contig2_All, CL437.Contig3_All, CL437.Contig4_All, CL437.Contig5_All, CL437.Contig6_All, CL4392.Contig1_All, CL4405.Contig1_All, CL4415.Contig1_All, CL4430.Contig1_All, CL444.Contig3_All, CL4445.Contig1_All, CL4462.Contig1_All, CL447.Contig1_All, CL447.Contig2_All, CL447.Contig3_All, CL447.Contig4_All, CL447.Contig5_All, CL447.Contig6_All, CL4471.Contig1_All, CL4477.Contig1_All, CL4477.Contig2_All, CL452.Contig1_All, CL452.Contig2_All, CL452.Contig3_All, CL452.Contig4_All, CL452.Contig5_All, CL4529.Contig1_All, CL4543.Contig1_All, CL4549.Contig1_All, CL4568.Contig1_All, CL4591.Contig2_All, CL4591.Contig3_All, CL4608.Contig1_All, CL4629.Contig3_All, CL4632.Contig1_All, CL4632.Contig2_All, CL4647.Contig1_All, CL4647.Contig3_All, CL4666.Contig1_All, CL4675.Contig1_All, CL4682.Contig1_All, CL4716.Contig1_All, CL4716.Contig2_All, CL4731.Contig1_All, CL4743.Contig1_All, CL4751.Contig1_All, CL476.Contig1_All, CL476.Contig2_All, CL476.Contig3_All, CL476.Contig4_All, CL476.Contig5_All, CL4775.Contig1_All, CL4811.Contig1_All, CL4821.Contig1_All, CL4836.Contig1_All, CL4837.Contig1_All, CL4842.Contig1_All, CL4846.Contig1_All, CL4850.Contig1_All, CL4850.Contig2_All, CL4850.Contig3_All, CL4865.Contig1_All, CL4865.Contig2_All, CL4869.Contig1_All, CL4917.Contig1_All, CL4932.Contig1_All, CL4954.Contig1_All, CL4954.Contig2_All, CL4961.Contig1_All, CL4963.Contig1_All, CL4974.Contig1_All, CL4980.Contig1_All, CL50.Contig10_All, CL50.Contig11_All, CL50.Contig12_All, CL50.Contig13_All, CL50.Contig14_All, CL50.Contig15_All, CL50.Contig16_All, CL50.Contig1_All, CL50.Contig2_All, CL50.Contig3_All, CL50.Contig4_All, CL50.Contig5_All, CL50.Contig6_All, CL50.Contig7_All, CL50.Contig8_All, CL50.Contig9_All, CL5017.Contig1_All, CL5020.Contig1_All, CL5020.Contig2_All, CL504.Contig1_All, CL504.Contig2_All, CL504.Contig3_All, CL504.Contig4_All, CL504.Contig5_All, CL5066.Contig1_All, CL5081.Contig1_All, CL5092.Contig1_All, CL5100.Contig1_All, CL511.Contig1_All, CL511.Contig2_All, CL511.Contig3_All, CL511.Contig4_All, CL511.Contig5_All, CL5138.Contig1_All, CL5179.Contig1_All, CL5179.Contig2_All, CL5183.Contig1_All, CL5183.Contig2_All, CL5183.Contig3_All, CL5201.Contig1_All, CL5215.Contig1_All,

			CL5224.Contig1_All, CL5225.Contig1_All, CL5230.Contig1_All, CL5231.Contig1_All, CL5237.Contig1_All, CL5264.Contig1_All, CL5264.Contig2_All, CL53.Contig10_All, CL53.Contig11_All, CL53.Contig1_All, CL53.Contig3_All, CL53.Contig6_All, CL53.Contig7_All, CL53.Contig8_All, CL53.Contig9_All, CL530.Contig1_All, CL530.Contig2_All, CL530.Contig3_All, CL5300.Contig1_All, CL5309.Contig1_All, CL5325.Contig1_All, CL5333.Contig1_All, CL5337.Contig1_All, CL5395.Contig1_All, CL5400.Contig1_All, CL5402.Contig1_All, CL5408.Contig1_All, CL5410.Contig1_All, CL5431.Contig1_All, CL5443.Contig1_All, CL5445.Contig1_All, CL5463.Contig1_All, CL5477.Contig1_All, CL5478.Contig1_All, CL5495.Contig1_All, CL5504.Contig1_All, CL5516.Contig1_All, CL5523.Contig1_All, CL5526.Contig1_All, CL5538.Contig1_All, CL5549.Contig1_All, CL5549.Contig2_All, CL5583.Contig1_All, CL5585.Contig1_All, CL56.Contig10_All, CL56.Contig12_All, CL56.Contig9_All, CL5637.Contig1_All, CL5637.Contig2_All, CL5639.Contig1_All, CL5678.Contig1_All, CL5691.Contig1_All, CL5691.Contig2_All, CL5737.Contig1_All, CL5755.Contig1_All, CL5787.Contig1_All, CL5793.Contig1_All, CL5829.Contig1_All, CL5843.Contig1_All, CL5864.Contig1_All, CL5864.Contig2_All, CL5877.Contig1_All, CL5884.Contig1_All, CL5906.Contig1_All, CL5930.Contig1_All, CL5936.Contig1_All, CL5936.Contig2_All, CL5942.Contig1_All, CL5968.Contig1_All, CL5984.Contig1_All, CL6013.Contig1_All, CL6025.Contig1_All, CL6042.Contig1_All, CL6051.Contig1_All, CL6064.Contig1_All, CL6089.Contig1_All, CL609.Contig1_All, CL609.Contig2_All, CL609.Contig3_All, CL609.Contig4_All, CL61.Contig10_All, CL61.Contig1_All, CL61.Contig2_All, CL61.Contig3_All, CL61.Contig4_All, CL61.Contig5_All, CL61.Contig6_All, CL61.Contig7_All, CL61.Contig8_All, CL61.Contig9_All, CL6124.Contig1_All, CL6141.Contig1_All, CL6141.Contig2_All, CL6146.Contig1_All, CL6172.Contig1_All, CL619.Contig1_All, CL6199.Contig1_All, CL6199.Contig2_All, CL6214.Contig1_All, CL6233.Contig1_All, CL6287.Contig1_All, CL63.Contig3_All, CL63.Contig5_All, CL63.Contig7_All, CL6371.Contig1_All, CL6395.Contig1_All, CL6396.Contig1_All, CL6401.Contig1_All, CL6402.Contig1_All, CL641.Contig1_All, CL641.Contig2_All, CL641.Contig3_All, CL6410.Contig2_All, CL6466.Contig2_All, CL6475.Contig1_All, CL6479.Contig1_All, CL649.Contig1_All, CL649.Contig2_All, CL6501.Contig1_All, CL6511.Contig1_All, CL6515.Contig1_All, CL6534.Contig1_All, CL6559.Contig1_All, CL656.Contig1_All, CL656.Contig2_All, CL656.Contig3_All, CL656.Contig4_All, CL6565.Contig1_All,

			CL6587.Contig1_All, CL66.Contig4_All, CL6613.Contig1_All, CL6613.Contig2_All, CL6626.Contig1_All, CL6637.Contig1_All, CL6652.Contig1_All, CL6700.Contig1_All, CL6709.Contig1_All, CL6722.Contig1_All, CL6744.Contig1_All, CL6792.Contig1_All, CL6792.Contig2_All, CL6799.Contig1_All, CL6799.Contig2_All, CL68.Contig1_All, CL68.Contig3_All, CL68.Contig4_All, CL68.Contig6_All, CL68.Contig8_All, CL6805.Contig1_All, CL6840.Contig1_All, CL6861.Contig1_All, CL6867.Contig1_All, CL6870.Contig1_All, CL6877.Contig1_All, CL6877.Contig2_All, CL69.Contig1_All, CL69.Contig2_All, CL69.Contig3_All, CL69.Contig4_All, CL69.Contig5_All, CL69.Contig6_All, CL69.Contig7_All, CL69.Contig8_All, CL6914.Contig1_All, CL6929.Contig1_All, CL6946.Contig1_All, CL697.Contig1_All, CL6981.Contig1_All, CL700.Contig1_All, CL700.Contig2_All, CL700.Contig3_All, CL700.Contig4_All, CL700.Contig5_All, CL702.Contig1_All, CL702.Contig2_All, CL702.Contig3_All, CL702.Contig4_All, CL7032.Contig1_All, CL7049.Contig1_All, CL7052.Contig1_All, CL7063.Contig1_All, CL7063.Contig2_All, CL7069.Contig1_All, CL7072.Contig1_All, CL7095.Contig1_All, CL71.Contig2_All, CL71.Contig3_All, CL7125.Contig1_All, CL7130.Contig1_All, CL7174.Contig1_All, CL7192.Contig1_All, CL7192.Contig2_All, CL7195.Contig2_All, CL7202.Contig1_All, CL7213.Contig1_All, CL7213.Contig2_All, CL7230.Contig1_All, CL7244.Contig1_All, CL7244.Contig2_All, CL7257.Contig1_All, CL7260.Contig1_All, CL7261.Contig1_All, CL7261.Contig2_All, CL7271.Contig1_All, CL7280.Contig1_All, CL7289.Contig1_All, CL729.Contig1_All, CL729.Contig2_All, CL729.Contig3_All, CL729.Contig4_All, CL7314.Contig1_All, CL7326.Contig1_All, CL7335.Contig1_All, CL7354.Contig1_All, CL7364.Contig1_All, CL7381.Contig1_All, CL7409.Contig1_All, CL7438.Contig1_All, CL7442.Contig1_All, CL7478.Contig1_All, CL752.Contig1_All, CL752.Contig2_All, CL752.Contig3_All, CL752.Contig4_All, CL752.Contig5_All, CL7521.Contig1_All, CL7545.Contig1_All, CL7545.Contig2_All, CL757.Contig1_All, CL757.Contig2_All, CL757.Contig3_All, CL757.Contig4_All, CL7584.Contig1_All, CL759.Contig3_All, CL759.Contig4_All, CL759.Contig5_All, CL759.Contig6_All, CL7593.Contig1_All, CL7621.Contig1_All, CL7655.Contig1_All, CL7655.Contig2_All, CL766.Contig1_All, CL766.Contig2_All, CL766.Contig3_All, CL766.Contig4_All, CL766.Contig5_All, CL7700.Contig1_All, CL7707.Contig1_All, CL7732.Contig1_All, CL7733.Contig1_All, CL7747.Contig1_All, CL7758.Contig1_All, CL7772.Contig1_All, CL7786.Contig1_All, CL7799.Contig1_All, CL7816.Contig1_All, CL7829.Contig1_All, CL7833.Contig1_All, CL7840.Contig1_All, CL7879.Contig1_All,

			CL7902.Contig1_All, CL7904.Contig1_All, CL793.Contig1_All, CL7974.Contig1_All, CL8054.Contig1_All, CL8059.Contig1_All, CL8138.Contig1_All, CL8172.Contig1_All, CL8197.Contig1_All, CL8200.Contig1_All, CL8215.Contig1_All, CL8217.Contig1_All, CL8222.Contig1_All, CL8223.Contig1_All, CL8261.Contig1_All, CL8288.Contig1_All, CL8297.Contig1_All, CL8300.Contig1_All, CL8307.Contig1_All, CL832.Contig1_All, CL832.Contig2_All, CL8324.Contig1_All, CL8366.Contig1_All, CL8381.Contig1_All, CL8384.Contig1_All, CL8386.Contig1_All, CL8390.Contig1_All, CL8393.Contig1_All, CL8398.Contig1_All, CL8407.Contig1_All, CL8423.Contig1_All, CL8485.Contig1_All, CL849.Contig1_All, CL849.Contig2_All, CL8496.Contig1_All, CL8505.Contig1_All, CL851.Contig4_All, CL8535.Contig1_All, CL8545.Contig1_All, CL8546.Contig1_All, CL8555.Contig1_All, CL8559.Contig1_All, CL856.Contig2_All, CL8624.Contig1_All, CL8630.Contig1_All, CL8638.Contig1_All, CL8651.Contig1_All, CL8687.Contig1_All, CL8690.Contig1_All, CL8697.Contig1_All, CL8700.Contig1_All, CL8707.Contig1_All, CL8718.Contig1_All, CL8720.Contig1_All, CL8723.Contig1_All, CL8738.Contig1_All, CL8747.Contig1_All, CL8749.Contig1_All, CL8762.Contig1_All, CL8773.Contig1_All, CL8804.Contig1_All, CL881.Contig1_All, CL881.Contig2_All, CL881.Contig3_All, CL881.Contig4_All, CL8823.Contig1_All, CL8873.Contig1_All, CL8878.Contig1_All, CL8905.Contig1_All, CL8915.Contig1_All, CL8943.Contig1_All, CL8945.Contig1_All, CL8959.Contig1_All, CL8962.Contig1_All, CL8979.Contig1_All, CL898.Contig1_All, CL898.Contig2_All, CL9002.Contig1_All, CL9055.Contig1_All, CL9057.Contig1_All, CL908.Contig1_All, CL908.Contig2_All, CL908.Contig3_All, CL908.Contig4_All, CL9106.Contig1_All, CL9150.Contig1_All, CL9174.Contig1_All, CL9184.Contig1_All, CL9195.Contig1_All, CL921.Contig1_All, CL921.Contig2_All, CL921.Contig3_All, CL921.Contig4_All, CL921.Contig5_All, CL921.Contig6_All, CL921.Contig7_All, CL921.Contig8_All, CL9220.Contig1_All, CL9226.Contig1_All, CL927.Contig1_All, CL927.Contig2_All, CL927.Contig3_All, CL927.Contig4_All, CL927.Contig5_All, CL927.Contig6_All, CL927.Contig7_All, CL927.Contig8_All, CL9275.Contig1_All, CL928.Contig1_All, CL928.Contig2_All, CL9291.Contig1_All, CL9291.Contig2_All, CL9302.Contig1_All, CL932.Contig1_All, CL932.Contig2_All, CL932.Contig3_All, CL932.Contig4_All, CL9383.Contig1_All, CL9383.Contig2_All, CL9407.Contig1_All, CL9429.Contig1_All, CL9435.Contig1_All, CL9450.Contig1_All, CL9517.Contig1_All, CL9539.Contig1_All, CL9550.Contig1_All, CL9652.Contig1_All, CL9677.Contig1_All, CL9688.Contig1_All,

			CL9723.Contig1_All, CL9749.Contig1_All, CL9821.Contig1_All, CL986.Contig1_All, CL986.Contig2_All, CL986.Contig3_All, CL986.Contig4_All, CL986.Contig5_All, CL9868.Contig1_All, CL9950.Contig1_All, CL9984.Contig1_All, Unigene1017_All, Unigene1045_All, Unigene1055_All, Unigene1062_All, Unigene1064_All, Unigene1109_All, Unigene1120_All, Unigene1142_All, Unigene1232_All, Unigene1267_All, Unigene1269_All, Unigene1285_All, Unigene1348_All, Unigene1401_All, Unigene1419_All, Unigene1434_All, Unigene1470_All, Unigene1482_All, Unigene1484_All, Unigene1543_All, Unigene1564_All, Unigene1586_All, Unigene1599_All, Unigene1603_All, Unigene1634_All, Unigene1655_All, Unigene1694_All, Unigene1707_All, Unigene1760_All, Unigene1773_All, Unigene1774_All, Unigene1781_All, Unigene1788_All, Unigene1796_All, Unigene1878_All, Unigene1909_All, Unigene1914_All, Unigene198_All, Unigene205_All, Unigene2109_All, Unigene2126_All, Unigene2129_All, Unigene2140_All, Unigene2148_All, Unigene2167_All, Unigene2178_All, Unigene2263_All, Unigene2291_All, Unigene2302_All, Unigene2308_All, Unigene2334_All, Unigene2335_All, Unigene2338_All, Unigene2360_All, Unigene2429_All, Unigene2449_All, Unigene2466_All, Unigene2472_All, Unigene2511_All, Unigene2530_All, Unigene2543_All, Unigene2549_All, Unigene2574_All, Unigene2590_All, Unigene259_All, Unigene2637_All, Unigene2646_All, Unigene2697_All, Unigene2717_All, Unigene2836_All, Unigene2839_All, Unigene2865_All, Unigene2869_All, Unigene2872_All, Unigene2873_All, Unigene2888_All, Unigene2961_All, Unigene2962_All, Unigene2963_All, Unigene2964_All, Unigene2970_All, Unigene3055_All, Unigene3071_All, Unigene3163_All, Unigene316_All, Unigene3203_All, Unigene325_All, Unigene327_All, Unigene3368_All, Unigene3378_All, Unigene3397_All, Unigene3447_All, Unigene346_All, Unigene3503_All, Unigene3504_All, Unigene3611_All, Unigene3637_All, Unigene363_All, Unigene3648_All, Unigene3654_All, Unigene3670_All, Unigene3707_All, Unigene3708_All, Unigene3719_All, Unigene3744_All, Unigene3764_All, Unigene3769_All, Unigene3792_All, Unigene3793_All, Unigene3870_All, Unigene3918_All, Unigene3983_All, Unigene4058_All, Unigene4065_All, Unigene4094_All, Unigene4096_All, Unigene4109_All, Unigene4118_All, Unigene4153_All, Unigene4157_All, Unigene4164_All, Unigene4178_All, Unigene4180_All, Unigene4184_All, Unigene4219_All, Unigene4275_All, Unigene4277_All, Unigene4292_All, Unigene4302_All, Unigene4340_All, Unigene4348_All, Unigene4370_All, Unigene4417_All, Unigene4420_All, Unigene4438_All, Unigene4502_All, Unigene4507_All, Unigene4527_All, Unigene4538_All, Unigene4539_All, Unigene4613_All, Unigene4627_All, Unigene462_All, Unigene4640_All, Unigene4646_All, Unigene4671_All, Unigene4676_All, Unigene4688_All, Unigene4727_All,

			Unigene4732_All, Unigene4773_All, Unigene4811_All, Unigene4818_All, Unigene4876_All, Unigene4943_All, Unigene4968_All, Unigene4973_All, Unigene4983_All, Unigene4987_All, Unigene4999_All, Unigene5015_All, Unigene5048_All, Unigene5117_All, Unigene5120_All, Unigene5134_All, Unigene515_All, Unigene5184_All, Unigene5207_All, Unigene5236_All, Unigene5238_All, Unigene5242_All, Unigene5261_All, Unigene5262_All, Unigene5264_All, Unigene5292_All, Unigene5316_All, Unigene5329_All, Unigene5340_All, Unigene5364_All, Unigene5438_All, Unigene5473_All, Unigene5496_All, Unigene5509_All, Unigene5518_All, Unigene5522_All, Unigene5525_All, Unigene619_All, Unigene644_All, Unigene652_All, Unigene657_All, Unigene666_All, Unigene708_All, Unigene71_All, Unigene733_All, Unigene735_All, Unigene800_All, Unigene828_All, Unigene865_All, Unigene895_All, Unigene920_All, Unigene938_All, Unigene961_All, Unigene979_All, Unigene990_All
3			CL1.Contig109_All, CL1.Contig116_All, CL1.Contig125_All, CL1.Contig32_All, CL1.Contig50_All, CL1.Contig71_All, CL1.Contig75_All, CL1.Contig84_All, CL100.Contig2_All, CL100.Contig4_All, CL100.Contig5_All, CL100.Contig7_All, CL10005.Contig1_All, CL1001.Contig1_All, CL10071.Contig1_All, CL10178.Contig1_All, CL10184.Contig1_All, CL10234.Contig1_All, CL1025.Contig1_All, CL10257.Contig1_All, CL1028.Contig1_All, CL1028.Contig2_All, CL1028.Contig4_All, CL103.Contig2_All, CL103.Contig3_All, CL103.Contig7_All, CL10328.Contig1_All, CL10329.Contig1_All, CL10335.Contig1_All, CL10373.Contig1_All, CL10386.Contig1_All, CL10400.Contig1_All, CL10428.Contig1_All, CL10447.Contig1_All, CL10459.Contig1_All, CL10470.Contig1_All, CL10506.Contig1_All, CL1052.Contig1_All, CL1052.Contig2_All, CL1052.Contig3_All, CL10563.Contig1_All, CL10612.Contig1_All, CL10653.Contig1_All, CL1066.Contig3_All, CL10683.Contig1_All, CL10726.Contig1_All, CL10806.Contig1_All, CL10822.Contig1_All, CL10873.Contig1_All, CL10926.Contig1_All, CL10930.Contig2_All, CL10931.Contig1_All, CL10933.Contig1_All, CL10941.Contig1_All, CL10945.Contig1_All, CL10989.Contig1_All, CL11049.Contig1_All, CL11054.Contig1_All, CL11204.Contig2_All, CL113.Contig2_All, CL113.Contig5_All, CL113.Contig6_All, CL113.Contig9_All, CL11313.Contig1_All, CL11395.Contig1_All, CL1146.Contig1_All, CL1146.Contig2_All, CL1146.Contig3_All, CL1146.Contig4_All, CL1153.Contig4_All, CL1159.Contig2_All, CL1159.Contig3_All, CL1163.Contig1_All, CL1163.Contig3_All, CL11716.Contig1_All, CL1179.Contig1_All,

	[Plant-pathogen interaction]()	CL11793.Contig1_All, CL11814.Contig1_All, CL1182.Contig3_All, CL11849.Contig1_All, CL11856.Contig1_All, CL1186.Contig1_All, CL12.Contig10_All, CL12.Contig11_All, CL12.Contig12_All, CL12.Contig13_All, CL12.Contig14_All, CL12.Contig15_All, CL12.Contig16_All, CL12.Contig1_All, CL12.Contig3_All, CL12.Contig4_All, CL12.Contig5_All, CL12.Contig6_All, CL12.Contig7_All, CL12.Contig8_All, CL12.Contig9_All, CL12046.Contig1_All, CL12082.Contig1_All, CL12087.Contig1_All, CL12087.Contig2_All, CL12090.Contig1_All, CL121.Contig2_All, CL121.Contig3_All, CL121.Contig4_All, CL121.Contig5_All, CL12125.Contig1_All, CL1218.Contig1_All, CL1218.Contig2_All, CL12259.Contig1_All, CL12297.Contig1_All, CL12310.Contig1_All, CL12326.Contig1_All, CL1238.Contig2_All, CL12422.Contig1_All, CL12455.Contig1_All, CL12483.Contig1_All, CL125.Contig2_All, CL125.Contig3_All, CL125.Contig4_All, CL125.Contig5_All, CL125.Contig6_All, CL125.Contig7_All, CL12787.Contig1_All, CL12817.Contig1_All, CL12832.Contig1_All, CL12834.Contig1_All, CL12872.Contig1_All, CL12883.Contig1_All, CL12889.Contig1_All, CL12890.Contig2_All, CL12947.Contig1_All, CL1297.Contig1_All, CL13109.Contig1_All, CL13156.Contig1_All, CL13160.Contig1_All, CL13212.Contig1_All, CL13266.Contig1_All, CL1336.Contig1_All, CL13380.Contig1_All, CL13392.Contig1_All, CL13423.Contig1_All, CL13478.Contig1_All, CL13598.Contig1_All, CL136.Contig1_All, CL136.Contig2_All, CL136.Contig3_All, CL136.Contig4_All, CL13658.Contig1_All, CL13813.Contig1_All, CL1389.Contig1_All, CL1389.Contig2_All, CL13917.Contig1_All, CL13937.Contig1_All, CL13938.Contig1_All, CL13947.Contig1_All, CL13959.Contig1_All, CL1398.Contig1_All, CL14.Contig10_All, CL14.Contig11_All, CL14.Contig12_All, CL14.Contig13_All, CL14.Contig14_All, CL14.Contig15_All, CL14.Contig16_All, CL14.Contig17_All, CL14.Contig1_All, CL14.Contig2_All, CL14.Contig4_All, CL14.Contig5_All, CL14.Contig6_All, CL14.Contig7_All, CL14.Contig8_All, CL14.Contig9_All, CL140.Contig1_All, CL140.Contig2_All, CL140.Contig6_All, CL140.Contig9_All, CL14018.Contig1_All, CL14054.Contig1_All, CL14114.Contig1_All, CL14145.Contig1_All, CL14149.Contig1_All, CL14163.Contig1_All, CL142.Contig2_All, CL142.Contig4_All, CL142.Contig5_All, CL142.Contig6_All, CL142.Contig7_All, CL1425.Contig1_All, CL14298.Contig1_All, CL14329.Contig1_All, CL14338.Contig1_All, CL14383.Contig1_All, CL14471.Contig1_All, CL14527.Contig1_All, CL14529.Contig1_All, CL14576.Contig1_All, CL14602.Contig1_All, CL14636.Contig1_All, CL14670.Contig1_All, CL14697.Contig1_All, CL1470.Contig1_All, CL14763.Contig1_All,

		CL14777.Contig1_All, CL14814.Contig1_All, CL14825.Contig1_All, CL14832.Contig1_All, CL14835.Contig1_All, CL14865.Contig1_All, CL1487.Contig1_All, CL14878.Contig1_All, CL14939.Contig1_All, CL14951.Contig1_All, CL14979.Contig1_All, CL1498.Contig2_All, CL15056.Contig1_All, CL15068.Contig1_All, CL15132.Contig1_All, CL15159.Contig1_All, CL15185.Contig1_All, CL15232.Contig1_All, CL15235.Contig1_All, CL15236.Contig1_All, CL15393.Contig1_All, CL15439.Contig1_All, CL15463.Contig1_All, CL1556.Contig1_All, CL15609.Contig1_All, CL15613.Contig1_All, CL15637.Contig1_All, CL15648.Contig1_All, CL1566.Contig1_All, CL15662.Contig1_All, CL1573.Contig1_All, CL15737.Contig1_All, CL15756.Contig1_All, CL15768.Contig1_All, CL15769.Contig1_All, CL15776.Contig1_All, CL15817.Contig1_All, CL15841.Contig1_All, CL15845.Contig1_All, CL15847.Contig1_All, CL15925.Contig1_All, CL15964.Contig1_All, CL15969.Contig1_All, CL15977.Contig1_All, CL1599.Contig1_All, CL15992.Contig1_All, CL1600.Contig2_All, CL16027.Contig1_All, CL1604.Contig1_All, CL16058.Contig1_All, CL16068.Contig1_All, CL16085.Contig1_All, CL16102.Contig1_All, CL16108.Contig1_All, CL1612.Contig1_All, CL16136.Contig1_All, CL16174.Contig1_All, CL16182.Contig1_All, CL16209.Contig1_All, CL16220.Contig1_All, CL16267.Contig1_All, CL16318.Contig1_All, CL1634.Contig1_All, CL1634.Contig2_All, CL1634.Contig3_All, CL1634.Contig4_All, CL16343.Contig1_All, CL16379.Contig1_All, CL1639.Contig1_All, CL164.Contig1_All, CL164.Contig2_All, CL164.Contig3_All, CL164.Contig4_All, CL164.Contig5_All, CL16416.Contig1_All, CL1646.Contig1_All, CL16476.Contig1_All, CL16496.Contig1_All, CL16497.Contig1_All, CL16529.Contig1_All, CL16558.Contig1_All, CL16560.Contig1_All, CL16581.Contig1_All, CL16588.Contig1_All, CL16624.Contig1_All, CL16679.Contig1_All, CL1668.Contig1_All, CL16680.Contig1_All, CL1669.Contig1_All, CL1669.Contig2_All, CL16691.Contig1_All, CL16695.Contig1_All, CL16708.Contig1_All, CL16724.Contig1_All, CL16757.Contig1_All, CL16784.Contig1_All, CL16804.Contig1_All, CL16809.Contig1_All, CL16839.Contig1_All, CL16869.Contig1_All, CL16916.Contig1_All, CL16954.Contig1_All, CL1696.Contig2_All, CL16981.Contig1_All, CL17010.Contig1_All, CL17026.Contig1_All, CL17142.Contig1_All, CL17146.Contig1_All, CL17165.Contig1_All, CL17165.Contig2_All,

			CL17177.Contig1_All, CL17185.Contig1_All, CL1725.Contig1_All, CL17253.Contig1_All, CL17279.Contig1_All, CL17294.Contig1_All, CL1731.Contig1_All, CL17311.Contig1_All, CL17335.Contig1_All, CL17338.Contig1_All, CL17372.Contig2_All, CL174.Contig1_All, CL174.Contig2_All, CL174.Contig3_All, CL174.Contig4_All, CL17416.Contig1_All, CL17440.Contig1_All, CL17442.Contig1_All, CL17463.Contig1_All, CL17500.Contig1_All, CL17505.Contig1_All, CL17720.Contig1_All, CL17762.Contig1_All, CL17770.Contig1_All, CL1778.Contig1_All, CL17825.Contig1_All, CL17874.Contig1_All, CL17892.Contig1_All, CL17924.Contig1_All, CL17943.Contig1_All, CL17948.Contig1_All, CL17953.Contig1_All, CL17959.Contig1_All, CL17985.Contig1_All, CL18006.Contig1_All, CL18140.Contig1_All, CL18169.Contig1_All, CL18374.Contig1_All, CL18376.Contig1_All, CL18395.Contig1_All, CL184.Contig1_All, CL184.Contig2_All, CL184.Contig3_All, CL184.Contig4_All, CL184.Contig5_All, CL18414.Contig1_All, CL1843.Contig2_All, CL18448.Contig1_All, CL1848.Contig1_All, CL1848.Contig2_All, CL1848.Contig3_All, CL1848.Contig4_All, CL18513.Contig1_All, CL18539.Contig1_All, CL18578.Contig1_All, CL18591.Contig1_All, CL18647.Contig1_All, CL18704.Contig1_All, CL18758.Contig1_All, CL18769.Contig1_All, CL1877.Contig1_All, CL18775.Contig1_All, CL18799.Contig1_All, CL18851.Contig1_All, CL18885.Contig1_All, CL18890.Contig1_All, CL18922.Contig1_All, CL18928.Contig1_All, CL1895.Contig2_All, CL1895.Contig3_All, CL18992.Contig1_All, CL19039.Contig1_All, CL19137.Contig1_All, CL19154.Contig1_All, CL19158.Contig1_All, CL19165.Contig1_All, CL19222.Contig1_All, CL19229.Contig1_All, CL19292.Contig1_All, CL19317.Contig1_All, CL19332.Contig1_All, CL19426.Contig1_All, CL19431.Contig1_All, CL19462.Contig1_All, CL19464.Contig1_All, CL19488.Contig1_All, CL19506.Contig1_All, CL19612.Contig1_All, CL19612.Contig2_All, CL19618.Contig1_All, CL1962.Contig1_All, CL19648.Contig1_All, CL19699.Contig1_All, CL19751.Contig1_All, CL19840.Contig1_All, CL19852.Contig1_All, CL19865.Contig1_All, CL199.Contig3_All, CL199.Contig5_All, CL199.Contig6_All, CL199.Contig8_All, CL1990.Contig1_All, CL19945.Contig1_All, CL19970.Contig1_All, CL1998.Contig1_All, CL19980.Contig1_All, CL2.Contig10_All, CL2.Contig19_All, CL2.Contig24_All, CL2.Contig26_All, CL2.Contig27_All, CL2.Contig2_All, CL2.Contig34_All, CL2.Contig3_All, CL2.Contig41_All, CL2.Contig43_All, CL20033.Contig1_All, CL20046.Contig1_All,

			CL20061.Contig1_All, CL20063.Contig1_All, CL20084.Contig1_All, CL20095.Contig1_All, CL20139.Contig1_All, CL20172.Contig1_All, CL20235.Contig1_All, CL20266.Contig1_All, CL20278.Contig1_All, CL20293.Contig1_All, CL20377.Contig1_All, CL20384.Contig1_All, CL20394.Contig1_All, CL20417.Contig1_All, CL20471.Contig1_All, CL20491.Contig1_All, CL20514.Contig1_All, CL20546.Contig1_All, CL206.Contig10_All, CL206.Contig1_All, CL206.Contig2_All, CL206.Contig5_All, CL206.Contig8_All, CL206.Contig9_All, CL20605.Contig1_All, CL20619.Contig1_All, CL20652.Contig1_All, CL20656.Contig1_All, CL20669.Contig1_All, CL20690.Contig1_All, CL20706.Contig1_All, CL20732.Contig1_All, CL20739.Contig1_All, CL20780.Contig1_All, CL20793.Contig1_All, CL2084.Contig1_All, CL20841.Contig1_All, CL20849.Contig1_All, CL20854.Contig1_All, CL20905.Contig1_All, CL20911.Contig1_All, CL20918.Contig1_All, CL20920.Contig1_All, CL20929.Contig1_All, CL20939.Contig1_All, CL20978.Contig1_All, CL21025.Contig1_All, CL21110.Contig1_All, CL21124.Contig1_All, CL21171.Contig1_All, CL212.Contig1_All, CL212.Contig2_All, CL212.Contig3_All, CL212.Contig4_All, CL21254.Contig1_All, CL21263.Contig1_All, CL2128.Contig1_All, CL21316.Contig1_All, CL21330.Contig1_All, CL21362.Contig1_All, CL21412.Contig1_All, CL21526.Contig1_All, CL21543.Contig1_All, CL21575.Contig1_All, CL21587.Contig1_All, CL21587.Contig2_All, CL21673.Contig1_All, CL21706.Contig1_All, CL21767.Contig1_All, CL21772.Contig1_All, CL21786.Contig1_All, CL21795.Contig1_All, CL21803.Contig1_All, CL21805.Contig1_All, CL21847.Contig1_All, CL21866.Contig1_All, CL21974.Contig1_All, CL21980.Contig1_All, CL220.Contig10_All, CL220.Contig1_All, CL220.Contig3_All, CL220.Contig4_All, CL220.Contig5_All, CL220.Contig6_All, CL220.Contig7_All, CL220.Contig8_All, CL220.Contig9_All, CL22018.Contig1_All, CL2206.Contig1_All, CL22090.Contig1_All, CL22097.Contig1_All, CL22151.Contig1_All, CL22197.Contig1_All, CL22207.Contig1_All, CL22208.Contig1_All, CL22248.Contig1_All, CL22283.Contig1_All, CL22347.Contig1_All, CL22521.Contig1_All, CL2256.Contig1_All, CL22566.Contig1_All, CL22574.Contig1_All, CL22579.Contig1_All, CL2258.Contig3_All, CL2261.Contig1_All, CL2261.Contig2_All, CL22689.Contig1_All, CL22701.Contig1_All, CL2272.Contig1_All, CL22750.Contig1_All, CL2276.Contig1_All, CL228.Contig1_All, CL228.Contig2_All, CL228.Contig3_All, CL228.Contig4_All, CL228.Contig5_All,

			CL22831.Contig1_All, CL22871.Contig1_All, CL23019.Contig1_All, CL23023.Contig1_All, CL23028.Contig1_All, CL2307.Contig1_All, CL23097.Contig1_All, CL23125.Contig1_All, CL23247.Contig1_All, CL23264.Contig1_All, CL23293.Contig1_All, CL23295.Contig1_All, CL23315.Contig1_All, CL23322.Contig1_All, CL2339.Contig1_All, CL2340.Contig1_All, CL2340.Contig2_All, CL23417.Contig1_All, CL23442.Contig1_All, CL23454.Contig1_All, CL23474.Contig1_All, CL235.Contig3_All, CL236.Contig1_All, CL236.Contig2_All, CL23605.Contig1_All, CL23666.Contig1_All, CL23793.Contig1_All, CL23807.Contig1_All, CL23830.Contig1_All, CL23868.Contig1_All, CL23883.Contig1_All, CL2389.Contig2_All, CL23892.Contig1_All, CL23926.Contig1_All, CL24076.Contig1_All, CL24077.Contig1_All, CL24085.Contig1_All, CL24099.Contig1_All, CL24103.Contig1_All, CL2415.Contig1_All, CL24152.Contig1_All, CL24167.Contig1_All, CL24201.Contig1_All, CL2421.Contig1_All, CL2421.Contig2_All, CL24223.Contig1_All, CL24236.Contig1_All, CL24290.Contig1_All, CL2430.Contig1_All, CL2435.Contig1_All, CL24500.Contig1_All, CL24504.Contig1_All, CL24505.Contig1_All, CL24507.Contig1_All, CL2456.Contig1_All, CL24593.Contig1_All, CL24607.Contig1_All, CL2482.Contig1_All, CL2482.Contig2_All, CL24840.Contig1_All, CL24962.Contig1_All, CL2506.Contig1_All, CL25082.Contig1_All, CL25168.Contig1_All, CL25235.Contig1_All, CL25259.Contig1_All, CL25268.Contig1_All, CL253.Contig1_All, CL253.Contig2_All, CL2530.Contig1_All, CL2533.Contig1_All, CL254.Contig10_All, CL254.Contig11_All, CL254.Contig12_All, CL254.Contig13_All, CL254.Contig14_All, CL254.Contig15_All, CL254.Contig16_All, CL254.Contig17_All, CL254.Contig18_All, CL254.Contig19_All, CL254.Contig20_All, CL254.Contig3_All, CL254.Contig4_All, CL254.Contig6_All, CL254.Contig8_All, CL254.Contig9_All, CL25405.Contig1_All, CL25459.Contig1_All, CL25465.Contig1_All, CL25510.Contig1_All, CL25561.Contig1_All, CL25581.Contig1_All, CL25592.Contig1_All, CL256.Contig1_All, CL256.Contig2_All, CL256.Contig3_All, CL256.Contig4_All, CL256.Contig5_All, CL256.Contig6_All, CL256.Contig8_All, CL256.Contig9_All, CL25613.Contig1_All, CL25651.Contig1_All, CL25678.Contig1_All, CL25698.Contig1_All, CL25746.Contig1_All, CL25767.Contig1_All, CL25774.Contig1_All, CL25810.Contig1_All, CL25879.Contig1_All, CL25883.Contig1_All, CL2593.Contig1_All, CL25950.Contig1_All, CL25973.Contig1_All,

			CL25981.Contig1_All, CL26049.Contig1_All, CL26078.Contig1_All, CL26087.Contig1_All, CL26089.Contig1_All, CL26095.Contig1_All, CL26174.Contig1_All, CL26181.Contig1_All, CL26207.Contig1_All, CL26220.Contig1_All, CL26278.Contig1_All, CL26338.Contig1_All, CL26358.Contig1_All, CL26363.Contig1_All, CL26381.Contig1_All, CL26436.Contig1_All, CL26448.Contig1_All, CL2651.Contig1_All, CL2651.Contig2_All, CL2652.Contig1_All, CL2652.Contig2_All, CL26523.Contig1_All, CL26533.Contig1_All, CL26579.Contig1_All, CL26604.Contig1_All, CL26657.Contig1_All, CL26739.Contig1_All, CL26744.Contig1_All, CL2678.Contig1_All, CL2681.Contig1_All, CL26874.Contig1_All, CL26879.Contig1_All, CL2690.Contig1_All, CL2690.Contig2_All, CL26909.Contig1_All, CL26928.Contig1_All, CL2693.Contig1_All, CL26954.Contig1_All, CL2696.Contig1_All, CL2696.Contig2_All, CL26974.Contig1_All, CL26990.Contig1_All, CL27.Contig11_All, CL27148.Contig1_All, CL27151.Contig1_All, CL27183.Contig1_All, CL27217.Contig1_All, CL27227.Contig1_All, CL27238.Contig1_All, CL27258.Contig1_All, CL27276.Contig1_All, CL27326.Contig1_All, CL27353.Contig1_All, CL27383.Contig1_All, CL27386.Contig1_All, CL2743.Contig1_All, CL27435.Contig1_All, CL27513.Contig1_All, CL27520.Contig1_All, CL27534.Contig1_All, CL27574.Contig1_All, CL27662.Contig1_All, CL277.Contig10_All, CL277.Contig11_All, CL277.Contig12_All, CL277.Contig1_All, CL277.Contig2_All, CL277.Contig3_All, CL277.Contig4_All, CL277.Contig5_All, CL277.Contig6_All, CL277.Contig7_All, CL277.Contig8_All, CL277.Contig9_All, CL27735.Contig1_All, CL27813.Contig1_All, CL27814.Contig1_All, CL27827.Contig1_All, CL2784.Contig1_All, CL27846.Contig1_All, CL27975.Contig1_All, CL2801.Contig1_All, CL28044.Contig1_All, CL28069.Contig1_All, CL28116.Contig1_All, CL2835.Contig1_All, CL2835.Contig2_All, CL2841.Contig1_All, CL2853.Contig1_All, CL2885.Contig1_All, CL2889.Contig1_All, CL2898.Contig1_All, CL290.Contig5_All, CL290.Contig6_All, CL290.Contig7_All, CL290.Contig8_All, CL2908.Contig2_All, CL2910.Contig1_All, CL2955.Contig1_All, CL2963.Contig1_All, CL2963.Contig2_All, CL2963.Contig3_All, CL30.Contig1_All, CL30.Contig2_All, CL30.Contig3_All, CL30.Contig5_All, CL30.Contig6_All, CL30.Contig7_All, CL30.Contig8_All, CL3000.Contig1_All, CL3010.Contig2_All, CL3032.Contig1_All, CL3032.Contig2_All, CL307.Contig1_All, CL307.Contig2_All, CL307.Contig4_All, CL3097.Contig1_All, CL3107.Contig1_All,

			CL3122.Contig1_All, CL3145.Contig1_All, CL3154.Contig1_All, CL3155.Contig1_All, CL3165.Contig1_All, CL3165.Contig2_All, CL3165.Contig3_All, CL317.Contig1_All, CL317.Contig3_All, CL317.Contig4_All, CL317.Contig5_All, CL317.Contig6_All, CL317.Contig9_All, CL3173.Contig1_All, CL319.Contig1_All, CL319.Contig2_All, CL319.Contig3_All, CL319.Contig4_All, CL319.Contig5_All, CL319.Contig6_All, CL3195.Contig1_All, CL32.Contig15_All, CL3220.Contig1_All, CL3220.Contig2_All, CL3245.Contig1_All, CL3254.Contig1_All, CL3255.Contig2_All, CL3282.Contig1_All, CL3282.Contig2_All, CL3293.Contig1_All, CL3293.Contig2_All, CL33.Contig10_All, CL33.Contig11_All, CL33.Contig13_All, CL33.Contig14_All, CL33.Contig1_All, CL33.Contig2_All, CL33.Contig3_All, CL33.Contig4_All, CL33.Contig5_All, CL33.Contig6_All, CL33.Contig7_All, CL33.Contig9_All, CL3371.Contig1_All, CL3377.Contig1_All, CL3377.Contig2_All, CL3381.Contig1_All, CL3384.Contig1_All, CL34.Contig10_All, CL34.Contig11_All, CL34.Contig13_All, CL34.Contig14_All, CL34.Contig15_All, CL34.Contig16_All, CL34.Contig17_All, CL34.Contig18_All, CL34.Contig1_All, CL34.Contig3_All, CL34.Contig4_All, CL34.Contig5_All, CL34.Contig6_All, CL34.Contig7_All, CL34.Contig9_All, CL3463.Contig1_All, CL3470.Contig1_All, CL3487.Contig1_All, CL3497.Contig1_All, CL35.Contig12_All, CL35.Contig13_All, CL35.Contig15_All, CL35.Contig17_All, CL35.Contig2_All, CL35.Contig3_All, CL35.Contig5_All, CL35.Contig7_All, CL35.Contig9_All, CL3518.Contig1_All, CL3518.Contig2_All, CL3518.Contig3_All, CL3527.Contig1_All, CL3549.Contig1_All, CL3558.Contig1_All, CL3558.Contig2_All, CL357.Contig1_All, CL357.Contig7_All, CL3573.Contig1_All, CL3589.Contig1_All, CL3589.Contig2_All, CL3597.Contig1_All, CL36.Contig12_All, CL3613.Contig1_All, CL3691.Contig1_All, CL3691.Contig2_All, CL3692.Contig1_All, CL3692.Contig2_All, CL372.Contig1_All, CL372.Contig2_All, CL372.Contig3_All, CL372.Contig4_All, CL3731.Contig3_All, CL3748.Contig1_All, CL3756.Contig1_All, CL376.Contig2_All, CL3791.Contig1_All, CL380.Contig1_All, CL380.Contig2_All, CL380.Contig3_All, CL380.Contig4_All, CL380.Contig5_All, CL3823.Contig1_All, CL3831.Contig1_All, CL3832.Contig2_All, CL3861.Contig1_All, CL388.Contig6_All, CL389.Contig1_All, CL389.Contig2_All, CL389.Contig3_All, CL389.Contig4_All, CL389.Contig5_All, CL389.Contig6_All, CL389.Contig7_All, CL3892.Contig1_All, CL391.Contig1_All, CL391.Contig2_All, CL391.Contig3_All, CL3910.Contig1_All, CL3922.Contig1_All, CL3936.Contig1_All, CL3945.Contig1_All, CL3949.Contig1_All, CL397.Contig1_All, CL397.Contig2_All, CL397.Contig3_All, CL397.Contig4_All, CL3981.Contig1_All, CL3982.Contig1_All, CL3982.Contig2_All, CL4.Contig10_All, CL4.Contig11_All, CL4.Contig13_All, CL4.Contig21_All, CL4.Contig24_All, CL4004.Contig1_All, CL4013.Contig1_All, CL4042.Contig1_All, CL4043.Contig1_All,

			CL4047.Contig2_All, CL41.Contig10_All, CL415.Contig1_All, CL415.Contig2_All, CL415.Contig3_All, CL415.Contig4_All, CL415.Contig5_All, CL415.Contig6_All, CL4178.Contig1_All, CL4181.Contig1_All, CL4181.Contig2_All, CL42.Contig9_All, CL4286.Contig1_All, CL431.Contig2_All, CL432.Contig3_All, CL433.Contig1_All, CL433.Contig2_All, CL433.Contig3_All, CL433.Contig4_All, CL433.Contig5_All, CL4377.Contig1_All, CL4397.Contig1_All, CL4429.Contig2_All, CL4456.Contig1_All, CL4467.Contig1_All, CL4472.Contig1_All, CL4508.Contig1_All, CL4517.Contig1_All, CL4531.Contig1_All, CL4556.Contig1_All, CL4592.Contig1_All, CL4631.Contig1_All, CL4631.Contig2_All, CL4645.Contig1_All, CL4657.Contig1_All, CL4664.Contig1_All, CL4674.Contig1_All, CL4690.Contig1_All, CL4690.Contig2_All, CL4690.Contig3_All, CL4729.Contig1_All, CL4746.Contig1_All, CL4791.Contig1_All, CL480.Contig1_All, CL480.Contig4_All, CL4805.Contig1_All, CL4835.Contig1_All, CL4890.Contig1_All, CL49.Contig10_All, CL49.Contig11_All, CL49.Contig1_All, CL49.Contig2_All, CL49.Contig3_All, CL49.Contig4_All, CL49.Contig5_All, CL49.Contig6_All, CL49.Contig7_All, CL49.Contig8_All, CL49.Contig9_All, CL4907.Contig1_All, CL4976.Contig1_All, CL5.Contig16_All, CL5.Contig18_All, CL5.Contig22_All, CL5.Contig23_All, CL5.Contig28_All, CL5.Contig33_All, CL5.Contig34_All, CL5.Contig6_All, CL5051.Contig1_All, CL5058.Contig1_All, CL5067.Contig1_All, CL5071.Contig1_All, CL5088.Contig1_All, CL5088.Contig2_All, CL5118.Contig1_All, CL5142.Contig2_All, CL5172.Contig1_All, CL5177.Contig1_All, CL5202.Contig1_All, CL528.Contig1_All, CL529.Contig1_All, CL529.Contig2_All, CL529.Contig3_All, CL529.Contig4_All, CL5295.Contig1_All, CL5319.Contig1_All, CL5332.Contig1_All, CL5375.Contig1_All, CL5378.Contig1_All, CL5417.Contig1_All, CL5450.Contig1_All, CL546.Contig3_All, CL546.Contig4_All, CL5462.Contig1_All, CL5464.Contig1_All, CL547.Contig1_All, CL547.Contig2_All, CL55.Contig5_All, CL55.Contig6_All, CL55.Contig7_All, CL55.Contig8_All, CL5519.Contig1_All, CL5529.Contig1_All, CL5534.Contig1_All, CL5550.Contig1_All, CL5777.Contig1_All, CL5795.Contig1_All, CL585.Contig2_All, CL5860.Contig1_All, CL5909.Contig1_All, CL5923.Contig1_All, CL5967.Contig1_All, CL5969.Contig1_All, CL5969.Contig2_All, CL60.Contig9_All, CL601.Contig3_All, CL6024.Contig1_All, CL6029.Contig1_All, CL6029.Contig2_All, CL6055.Contig1_All, CL6055.Contig2_All, CL6087.Contig1_All, CL6087.Contig2_All, CL6126.Contig1_All, CL614.Contig1_All, CL614.Contig2_All, CL6188.Contig1_All, CL6228.Contig2_All, CL6228.Contig4_All, CL623.Contig3_All, CL6253.Contig1_All, CL6266.Contig1_All, CL6281.Contig1_All, CL6297.Contig1_All, CL6312.Contig1_All,

		CL6325.Contig1_All, CL6343.Contig1_All, CL6387.Contig1_All, CL639.Contig3_All, CL6399.Contig1_All, CL6408.Contig1_All, CL6435.Contig1_All, CL645.Contig1_All, CL6457.Contig1_All, CL646.Contig1_All, CL646.Contig2_All, CL6477.Contig1_All, CL648.Contig1_All, CL648.Contig2_All, CL648.Contig3_All, CL648.Contig4_All, CL6523.Contig1_All, CL6527.Contig1_All, CL6556.Contig1_All, CL6591.Contig1_All, CL6604.Contig1_All, CL6660.Contig1_All, CL6672.Contig1_All, CL67.Contig1_All, CL67.Contig2_All, CL67.Contig3_All, CL67.Contig4_All, CL67.Contig7_All, CL6725.Contig1_All, CL6756.Contig1_All, CL6766.Contig1_All, CL678.Contig1_All, CL678.Contig2_All, CL678.Contig3_All, CL678.Contig4_All, CL678.Contig5_All, CL6780.Contig1_All, CL6806.Contig1_All, CL684.Contig2_All, CL684.Contig3_All, CL684.Contig4_All, CL690.Contig1_All, CL690.Contig2_All, CL690.Contig3_All, CL692.Contig1_All, CL692.Contig2_All, CL6933.Contig1_All, CL6952.Contig1_All, CL6967.Contig1_All, CL6978.Contig1_All, CL7.Contig10_All, CL7.Contig26_All, CL7.Contig3_All, CL7.Contig9_All, CL7114.Contig1_All, CL7123.Contig1_All, CL7133.Contig1_All, CL7183.Contig1_All, CL7207.Contig1_All, CL7250.Contig1_All, CL7272.Contig1_All, CL7274.Contig1_All, CL7349.Contig1_All, CL7351.Contig1_All, CL736.Contig2_All, CL7393.Contig1_All, CL74.Contig10_All, CL74.Contig2_All, CL7499.Contig1_All, CL7541.Contig1_All, CL7542.Contig1_All, CL7546.Contig1_All, CL7623.Contig1_All, CL7657.Contig1_All, CL7691.Contig1_All, CL7709.Contig1_All, CL7775.Contig1_All, CL7779.Contig1_All, CL7801.Contig1_All, CL782.Contig2_All, CL7822.Contig1_All, CL7827.Contig1_All, CL7850.Contig1_All, CL7852.Contig1_All, CL7863.Contig1_All, CL7892.Contig1_All, CL7898.Contig1_All, CL790.Contig2_All, CL7969.Contig1_All, CL8.Contig30_All, CL803.Contig1_All, CL803.Contig2_All, CL803.Contig3_All, CL803.Contig4_All, CL803.Contig5_All, CL8074.Contig1_All, CL8083.Contig1_All, CL809.Contig1_All, CL809.Contig2_All, CL809.Contig3_All, CL8090.Contig1_All, CL8139.Contig1_All, CL815.Contig4_All, CL815.Contig5_All, CL8150.Contig1_All, CL8152.Contig1_All, CL819.Contig3_All, CL8208.Contig1_All, CL8209.Contig1_All, CL8217.Contig1_All, CL8248.Contig1_All, CL8319.Contig1_All, CL8387.Contig1_All, CL8402.Contig1_All, CL8411.Contig1_All, CL8498.Contig1_All, CL8498.Contig2_All, CL8506.Contig1_All, CL854.Contig1_All, CL854.Contig2_All, CL854.Contig3_All, CL8548.Contig1_All, CL8560.Contig1_All, CL8570.Contig1_All, CL8589.Contig1_All, CL8589.Contig2_All, CL8594.Contig1_All, CL8623.Contig1_All, CL8648.Contig1_All, CL8652.Contig1_All, CL8682.Contig1_All, CL87.Contig4_All, CL87.Contig5_All, CL8726.Contig1_All, CL877.Contig2_All, CL877.Contig3_All, CL8782.Contig1_All, CL8783.Contig1_All, CL8801.Contig1_All, CL881.Contig5_All, CL881.Contig6_All,

			CL8820.Contig1_All, CL8822.Contig1_All, CL885.Contig2_All, CL885.Contig3_All, CL885.Contig4_All, CL8886.Contig1_All, CL8895.Contig1_All, CL8895.Contig2_All, CL8920.Contig1_All, CL9.Contig18_All, CL9.Contig26_All, CL9.Contig27_All, CL9.Contig30_All, CL9.Contig31_All, CL9000.Contig1_All, CL9014.Contig1_All, CL9052.Contig1_All, CL906.Contig2_All, CL9071.Contig1_All, CL91.Contig4_All, CL91.Contig6_All, CL912.Contig3_All, CL912.Contig4_All, CL92.Contig1_All, CL92.Contig2_All, CL92.Contig3_All, CL92.Contig4_All, CL92.Contig5_All, CL92.Contig6_All, CL92.Contig7_All, CL9206.Contig1_All, CL9235.Contig1_All, CL9240.Contig1_All, CL9331.Contig1_All, CL9331.Contig2_All, CL9350.Contig1_All, CL9397.Contig1_All, CL9418.Contig1_All, CL9432.Contig1_All, CL9439.Contig1_All, CL9453.Contig1_All, CL9476.Contig1_All, CL9532.Contig1_All, CL9561.Contig1_All, CL9596.Contig1_All, CL9622.Contig1_All, CL9649.Contig1_All, CL9686.Contig1_All, CL97.Contig4_All, CL97.Contig5_All, CL97.Contig8_All, CL970.Contig1_All, CL970.Contig2_All, CL9706.Contig1_All, CL9710.Contig1_All, CL9718.Contig1_All, CL9780.Contig1_All, CL9836.Contig1_All, CL9856.Contig1_All, CL9864.Contig1_All, CL991.Contig1_All, CL991.Contig2_All, CL991.Contig3_All, CL9937.Contig1_All, Unigene1009_All, Unigene1093_All, Unigene1193_All, Unigene1302_All, Unigene1304_All, Unigene1333_All, Unigene1344_All, Unigene1366_All, Unigene1545_All, Unigene1571_All, Unigene1668_All, Unigene1701_All, Unigene1714_All, Unigene1749_All, Unigene1790_All, Unigene1846_All, Unigene1854_All, Unigene1900_All, Unigene1902_All, Unigene1959_All, Unigene1989_All, Unigene2027_All, Unigene2041_All, Unigene2057_All, Unigene2132_All, Unigene2134_All, Unigene2216_All, Unigene2314_All, Unigene2446_All, Unigene2482_All, Unigene2495_All, Unigene2523_All, Unigene257_All, Unigene2642_All, Unigene2663_All, Unigene2765_All, Unigene2777_All, Unigene2810_All, Unigene2892_All, Unigene2938_All, Unigene2987_All, Unigene3006_All, Unigene3054_All, Unigene3080_All, Unigene3145_All, Unigene3169_All, Unigene3226_All, Unigene3241_All, Unigene3296_All, Unigene3316_All, Unigene3317_All, Unigene3343_All, Unigene3383_All, Unigene3401_All, Unigene3408_All, Unigene3428_All, Unigene3466_All, Unigene3480_All, Unigene3485_All, Unigene3550_All, Unigene3582_All, Unigene3622_All, Unigene3643_All, Unigene3690_All, Unigene3784_All, Unigene3872_All, Unigene3884_All, Unigene3885_All, Unigene388_All, Unigene3935_All, Unigene3958_All, Unigene3985_All, Unigene3986_All, Unigene3987_All, Unigene4019_All, Unigene4024_All, Unigene406_All, Unigene4168_All, Unigene422_All, Unigene4231_All, Unigene4239_All, Unigene4270_All, Unigene4327_All, Unigene4328_All, Unigene4375_All,

			Unigene4470_All, Unigene4490_All, Unigene4499_All, Unigene4595_All, Unigene4599_All, Unigene4622_All, Unigene4648_All, Unigene4670_All, Unigene4774_All, Unigene4787_All, Unigene4852_All, Unigene4957_All, Unigene500_All, Unigene5062_All, Unigene5087_All, Unigene5172_All, Unigene5211_All, Unigene5215_All, Unigene5225_All, Unigene522_All, Unigene5270_All, Unigene5335_All, Unigene5379_All, Unigene5451_All, Unigene546_All, Unigene5478_All, Unigene5504_All, Unigene5505_All, Unigene5521_All, Unigene592_All, Unigene636_All, Unigene699_All, Unigene69_All, Unigene806_All, Unigene866_All, Unigene935_All, Unigene941_All
4			CL1.Contig100_All, CL1.Contig126_All, CL1.Contig137_All, CL1.Contig24_All, CL1.Contig66_All, CL1.Contig6_All, CL1.Contig76_All, CL1.Contig7_All, CL1.Contig82_All, CL100.Contig2_All, CL100.Contig3_All, CL100.Contig4_All, CL100.Contig5_All, CL100.Contig6_All, CL100.Contig7_All, CL100.Contig8_All, CL1001.Contig1_All, CL10085.Contig1_All, CL10178.Contig1_All, CL10193.Contig1_All, CL10234.Contig1_All, CL1024.Contig1_All, CL1024.Contig2_All, CL1024.Contig3_All, CL1024.Contig4_All, CL1024.Contig5_All, CL103.Contig6_All, CL10300.Contig1_All, CL10317.Contig1_All, CL10328.Contig1_All, CL1034.Contig1_All, CL10380.Contig1_All, CL10463.Contig1_All, CL10506.Contig1_All, CL10554.Contig1_All, CL1063.Contig1_All, CL1063.Contig2_All, CL1063.Contig3_All, CL1063.Contig4_All, CL1063.Contig5_All, CL10659.Contig1_All, CL1066.Contig3_All, CL10674.Contig1_All, CL1072.Contig1_All, CL1072.Contig2_All, CL1072.Contig3_All, CL1072.Contig4_All, CL10726.Contig1_All, CL1077.Contig2_All, CL108.Contig1_All, CL108.Contig3_All, CL108.Contig4_All, CL10806.Contig1_All, CL10832.Contig1_All, CL10926.Contig1_All, CL10930.Contig2_All, CL10945.Contig1_All, CL10956.Contig1_All, CL10980.Contig1_All, CL10989.Contig1_All, CL11089.Contig1_All, CL11096.Contig1_All, CL11128.Contig1_All, CL11139.Contig1_All, CL11180.Contig1_All, CL1119.Contig1_All, CL11233.Contig1_All, CL113.Contig8_All, CL11323.Contig1_All, CL11349.Contig1_All, CL11352.Contig1_All, CL11395.Contig1_All, CL11426.Contig1_All, CL11475.Contig1_All, CL11482.Contig1_All, CL1151.Contig1_All, CL1151.Contig2_All, CL1151.Contig3_All, CL1153.Contig1_All, CL1153.Contig2_All, CL11578.Contig1_All, CL11586.Contig1_All, CL1163.Contig1_All, CL1163.Contig3_All, CL11720.Contig1_All, CL11733.Contig1_All, CL1179.Contig1_All, CL1182.Contig3_All,

	Plant hormone signal	CL11856.Contig1_All, CL1186.Contig1_All, CL11860.Contig1_All, CL1189.Contig1_All, CL1189.Contig2_All, CL1189.Contig3_All, CL11890.Contig1_All, CL1202.Contig1_All, CL1202.Contig2_All, CL1202.Contig3_All, CL12033.Contig1_All, CL12046.Contig1_All, CL12092.Contig1_All, CL12125.Contig1_All, CL12259.Contig1_All, CL1229.Contig3_All, CL12320.Contig1_All, CL12326.Contig1_All, CL12393.Contig1_All, CL12404.Contig1_All, CL12417.Contig1_All, CL12438.Contig1_All, CL12441.Contig1_All, CL12453.Contig1_All, CL12476.Contig1_All, CL12484.Contig1_All, CL1253.Contig1_All, CL12566.Contig1_All, CL12589.Contig1_All, CL12598.Contig1_All, CL12637.Contig1_All, CL12652.Contig1_All, CL12658.Contig1_All, CL127.Contig1_All, CL127.Contig2_All, CL127.Contig3_All, CL127.Contig4_All, CL127.Contig5_All, CL12872.Contig1_All, CL12890.Contig2_All, CL12947.Contig1_All, CL12970.Contig1_All, CL12975.Contig1_All, CL130.Contig10_All, CL130.Contig1_All, CL130.Contig2_All, CL130.Contig3_All, CL130.Contig4_All, CL130.Contig5_All, CL130.Contig6_All, CL130.Contig7_All, CL130.Contig8_All, CL130.Contig9_All, CL13045.Contig1_All, CL13052.Contig1_All, CL13108.Contig1_All, CL13109.Contig1_All, CL13148.Contig1_All, CL13160.Contig1_All, CL13185.Contig1_All, CL13196.Contig1_All, CL13212.Contig1_All, CL13224.Contig1_All, CL13231.Contig1_All, CL13233.Contig1_All, CL1325.Contig1_All, CL13315.Contig1_All, CL13371.Contig1_All, CL13380.Contig1_All, CL13478.Contig1_All, CL13479.Contig1_All, CL13497.Contig1_All, CL1353.Contig1_All, CL13543.Contig1_All, CL13544.Contig1_All, CL13597.Contig1_All, CL136.Contig1_All, CL136.Contig2_All, CL136.Contig3_All, CL136.Contig4_All, CL13658.Contig1_All, CL13707.Contig1_All, CL13778.Contig1_All, CL13821.Contig1_All, CL13897.Contig1_All, CL13939.Contig1_All, CL1395.Contig1_All, CL1395.Contig2_All, CL1395.Contig3_All, CL1395.Contig4_All, CL1398.Contig1_All, CL14018.Contig1_All, CL14049.Contig1_All, CL1413.Contig1_All, CL142.Contig1_All, CL142.Contig5_All, CL142.Contig6_All, CL142.Contig7_All, CL14213.Contig1_All, CL1423.Contig1_All, CL1423.Contig2_All, CL1423.Contig3_All, CL1423.Contig4_All, CL143.Contig2_All, CL143.Contig3_All, CL143.Contig5_All, CL1432.Contig2_All, CL14373.Contig1_All, CL14409.Contig1_All, CL14409.Contig2_All, CL14415.Contig1_All, CL1453.Contig1_All, CL14602.Contig1_All, CL14636.Contig1_All, CL1467.Contig1_All, CL1467.Contig2_All, CL14721.Contig1_All, CL14739.Contig1_All, CL1474.Contig1_All,

	transduction	CL1480.Contig1_All, CL1483.Contig1_All, CL14850.Contig1_All, CL14929.Contig1_All, CL14970.Contig1_All, CL14989.Contig1_All, CL15007.Contig1_All, CL1505.Contig1_All, CL1505.Contig2_All, CL15056.Contig1_All, CL15068.Contig1_All, CL15134.Contig1_All, CL15159.Contig1_All, CL15232.Contig1_All, CL1524.Contig1_All, CL1528.Contig1_All, CL15314.Contig1_All, CL15342.Contig1_All, CL15374.Contig1_All, CL15393.Contig1_All, CL1542.Contig1_All, CL1542.Contig2_All, CL15429.Contig1_All, CL1549.Contig1_All, CL1549.Contig2_All, CL1549.Contig3_All, CL1549.Contig4_All, CL1549.Contig5_All, CL1553.Contig1_All, CL1553.Contig3_All, CL15583.Contig1_All, CL15583.Contig2_All, CL15613.Contig1_All, CL15706.Contig1_All, CL15732.Contig1_All, CL15737.Contig1_All, CL15753.Contig1_All, CL15781.Contig1_All, CL15781.Contig2_All, CL1581.Contig1_All, CL15831.Contig1_All, CL15925.Contig1_All, CL15964.Contig1_All, CL15978.Contig1_All, CL1600.Contig2_All, CL16011.Contig1_All, CL1602.Contig1_All, CL16027.Contig1_All, CL16035.Contig1_All, CL16136.Contig1_All, CL16139.Contig1_All, CL16144.Contig1_All, CL16167.Contig1_All, CL16174.Contig1_All, CL16220.Contig1_All, CL16265.Contig1_All, CL16282.Contig1_All, CL16292.Contig1_All, CL16312.Contig1_All, CL16332.Contig1_All, CL16367.Contig1_All, CL16456.Contig1_All, CL16476.Contig1_All, CL16497.Contig1_All, CL16501.Contig1_All, CL16606.Contig1_All, CL16606.Contig2_All, CL16624.Contig1_All, CL1668.Contig1_All, CL1669.Contig1_All, CL1669.Contig2_All, CL16724.Contig1_All, CL16754.Contig1_All, CL16809.Contig1_All, CL16857.Contig1_All, CL16869.Contig1_All, CL16886.Contig1_All, CL16937.Contig1_All, CL16960.Contig1_All, CL16973.Contig1_All, CL16991.Contig1_All, CL17010.Contig1_All, CL17045.Contig1_All, CL17059.Contig1_All, CL17092.Contig1_All, CL17092.Contig2_All, CL17173.Contig1_All, CL17177.Contig1_All, CL17185.Contig1_All, CL1725.Contig1_All, CL17276.Contig1_All, CL173.Contig10_All, CL173.Contig2_All, CL173.Contig3_All, CL173.Contig4_All, CL173.Contig6_All, CL173.Contig7_All, CL173.Contig8_All, CL17305.Contig1_All, CL1731.Contig1_All, CL17327.Contig1_All, CL17338.Contig1_All, CL17350.Contig1_All, CL17372.Contig1_All, CL17372.Contig2_All, CL174.Contig1_All, CL174.Contig2_All, CL174.Contig3_All, CL174.Contig4_All, CL1740.Contig1_All, CL1740.Contig2_All, CL1740.Contig3_All,

CL1740.Contig4_All, CL1740.Contig5_All,
CL17416.Contig1_All, CL17442.Contig1_All,
CL17463.Contig1_All, CL1747.Contig1_All,
CL17476.Contig1_All, CL17541.Contig1_All,
CL17640.Contig1_All, CL17734.Contig1_All,
CL17770.Contig1_All, CL1778.Contig1_All,
CL17812.Contig1_All, CL1794.Contig1_All,
CL1794.Contig2_All, CL1794.Contig3_All,
CL1794.Contig4_All, CL1794.Contig5_All,
CL17953.Contig1_All, CL17985.Contig1_All,
CL1812.Contig1_All, CL18169.Contig1_All,
CL1822.Contig1_All, CL18300.Contig1_All,
CL1833.Contig1_All, CL1833.Contig2_All,
CL18365.Contig1_All, CL18376.Contig1_All,
CL18395.Contig1_All, CL18458.Contig1_All,
CL18485.Contig1_All, CL18569.Contig1_All,
CL18634.Contig1_All, CL18647.Contig1_All,
CL18704.Contig1_All, CL18710.Contig1_All,
CL18786.Contig1_All, CL18928.Contig1_All,
CL18930.Contig1_All, CL18978.Contig1_All,
CL18992.Contig1_All, CL1910.Contig2_All,
CL19142.Contig1_All, CL19275.Contig1_All,
CL1931.Contig1_All, CL1931.Contig2_All,
CL1931.Contig3_All, CL1931.Contig4_All,
CL19352.Contig1_All, CL19372.Contig1_All,
CL19417.Contig1_All, CL19541.Contig1_All,
CL19569.Contig1_All, CL19603.Contig1_All,
CL19612.Contig1_All, CL19612.Contig2_All,
CL19618.Contig1_All, CL19699.Contig1_All,
CL19865.Contig1_All, CL1989.Contig1_All,
CL1989.Contig3_All, CL199.Contig2_All, CL199.Contig4_All,
CL1990.Contig1_All, CL1994.Contig1_All,
CL19959.Contig1_All, CL19964.Contig1_All,
CL19980.Contig1_All, CL2.Contig23_All, CL2.Contig29_All,
CL2.Contig31_All, CL2.Contig36_All, CL2.Contig37_All,
CL20.Contig13_All, CL20.Contig1_All, CL20.Contig2_All,
CL20.Contig5_All, CL20.Contig6_All, CL20.Contig9_All,
CL20068.Contig1_All, CL20088.Contig1_All,
CL20096.Contig1_All, CL20139.Contig1_All,
CL20186.Contig1_All, CL20226.Contig1_All,
CL20228.Contig1_All, CL20232.Contig1_All,
CL20266.Contig1_All, CL20278.Contig1_All,
CL20310.Contig1_All, CL20377.Contig1_All,
CL20471.Contig1_All, CL20493.Contig1_All,
CL20500.Contig1_All, CL2054.Contig1_All,
CL20563.Contig1_All, CL20576.Contig1_All,
CL20599.Contig1_All, CL20623.Contig1_All,
CL20631.Contig1_All, CL20656.Contig1_All,
CL2066.Contig1_All, CL2068.Contig1_All,
CL2068.Contig2_All, CL2068.Contig3_All,
CL2068.Contig4_All, CL2068.Contig5_All,
CL20706.Contig1_All, CL20736.Contig1_All,
CL20784.Contig1_All, CL20815.Contig1_All,
CL20841.Contig1_All, CL20849.Contig1_All,

			CL2087.Contig1_All, CL20911.Contig1_All, CL20945.Contig1_All, CL20998.Contig1_All, CL21114.Contig1_All, CL21116.Contig1_All, CL2113.Contig1_All, CL2113.Contig2_All, CL2113.Contig3_All, CL21171.Contig1_All, CL21215.Contig1_All, CL21221.Contig1_All, CL21231.Contig1_All, CL21242.Contig1_All, CL21246.Contig1_All, CL21254.Contig1_All, CL21291.Contig1_All, CL21398.Contig1_All, CL21412.Contig1_All, CL21415.Contig1_All, CL21425.Contig1_All, CL21461.Contig1_All, CL21491.Contig1_All, CL2151.Contig1_All, CL2151.Contig2_All, CL21526.Contig1_All, CL21587.Contig1_All, CL21587.Contig2_All, CL21767.Contig1_All, CL21772.Contig1_All, CL21786.Contig1_All, CL2194.Contig1_All, CL2197.Contig1_All, CL2200.Contig1_All, CL22039.Contig1_All, CL22056.Contig1_All, CL2206.Contig1_All, CL22184.Contig1_All, CL22207.Contig1_All, CL22210.Contig1_All, CL22236.Contig1_All, CL22237.Contig1_All, CL22248.Contig1_All, CL22289.Contig1_All, CL2231.Contig1_All, CL22334.Contig1_All, CL22347.Contig1_All, CL22348.Contig1_All, CL22356.Contig1_All, CL22381.Contig1_All, CL22483.Contig1_All, CL22579.Contig1_All, CL22632.Contig1_All, CL22768.Contig1_All, CL22871.Contig1_All, CL22962.Contig1_All, CL22967.Contig1_All, CL23012.Contig1_All, CL23019.Contig1_All, CL2307.Contig1_All, CL2316.Contig1_All, CL2316.Contig2_All, CL23212.Contig1_All, CL2324.Contig1_All, CL23281.Contig1_All, CL23291.Contig1_All, CL23295.Contig1_All, CL2340.Contig1_All, CL2340.Contig2_All, CL23476.Contig1_All, CL23487.Contig1_All, CL235.Contig3_All, CL23516.Contig1_All, CL23523.Contig1_All, CL23534.Contig1_All, CL2362.Contig1_All, CL2362.Contig2_All, CL2362.Contig3_All, CL2362.Contig4_All, CL2366.Contig1_All, CL2369.Contig1_All, CL2377.Contig1_All, CL23793.Contig1_All, CL2383.Contig1_All, CL2383.Contig2_All, CL2383.Contig3_All, CL23852.Contig1_All, CL23868.Contig1_All, CL23883.Contig1_All, CL2392.Contig1_All, CL23926.Contig1_All, CL23975.Contig1_All, CL24008.Contig1_All, CL24072.Contig1_All, CL24103.Contig1_All, CL24152.Contig1_All, CL24167.Contig1_All, CL24201.Contig1_All, CL2421.Contig2_All, CL2422.Contig1_All, CL2422.Contig2_All, CL24223.Contig1_All, CL24236.Contig1_All, CL2435.Contig1_All, CL24422.Contig1_All, CL24436.Contig1_All, CL24504.Contig1_All, CL24505.Contig1_All,

CL24514.Contig1_All, CL24603.Contig1_All,
CL24837.Contig1_All, CL24840.Contig1_All,
CL24868.Contig1_All, CL249.Contig1_All, CL249.Contig2_All,
CL249.Contig3_All, CL249.Contig4_All, CL24980.Contig1_All,
CL25045.Contig1_All, CL25125.Contig1_All,
CL25154.Contig1_All, CL25235.Contig1_All,
CL2532.Contig1_All, CL2532.Contig2_All,
CL2533.Contig1_All, CL25395.Contig1_All,
CL25523.Contig1_All, CL25548.Contig1_All,
CL2559.Contig1_All, CL256.Contig1_All, CL256.Contig2_All,
CL256.Contig3_All, CL256.Contig4_All, CL256.Contig5_All,
CL256.Contig6_All, CL256.Contig8_All, CL256.Contig9_All,
CL25651.Contig1_All, CL25669.Contig1_All,
CL25698.Contig1_All, CL25714.Contig1_All,
CL25738.Contig1_All, CL25741.Contig1_All,
CL25745.Contig1_All, CL25767.Contig1_All,
CL25803.Contig1_All, CL25815.Contig1_All,
CL2583.Contig1_All, CL2583.Contig2_All,
CL25837.Contig1_All, CL25950.Contig1_All,
CL25973.Contig1_All, CL26050.Contig1_All,
CL26078.Contig1_All, CL26079.Contig1_All,
CL26232.Contig1_All, CL26240.Contig1_All,
CL26300.Contig1_All, CL26307.Contig1_All,
CL26335.Contig1_All, CL26338.Contig1_All,
CL2635.Contig1_All, CL2636.Contig1_All,
CL26377.Contig1_All, CL26464.Contig1_All,
CL2651.Contig1_All, CL2651.Contig2_All,
CL26579.Contig1_All, CL26600.Contig1_All,
CL26685.Contig1_All, CL26690.Contig1_All,
CL26783.Contig1_All, CL26789.Contig1_All,
CL26824.Contig1_All, CL26879.Contig1_All,
CL2690.Contig1_All, CL2690.Contig2_All,
CL26909.Contig1_All, CL26924.Contig1_All,
CL26928.Contig1_All, CL27.Contig11_All, CL27.Contig13_All,
CL27.Contig1_All, CL270.Contig1_All, CL27005.Contig1_All,
CL27017.Contig1_All, CL27116.Contig1_All,
CL27148.Contig1_All, CL27183.Contig1_All,
CL2719.Contig1_All, CL27258.Contig1_All,
CL27266.Contig1_All, CL27276.Contig1_All,
CL27304.Contig1_All, CL27383.Contig1_All,
CL27386.Contig1_All, CL27489.Contig1_All,
CL27513.Contig1_All, CL27665.Contig1_All,
CL27716.Contig1_All, CL27806.Contig1_All,
CL27822.Contig1_All, CL27910.Contig1_All,
CL27915.Contig1_All, CL27988.Contig1_All,
CL280.Contig1_All, CL28018.Contig1_All,
CL28044.Contig1_All, CL2816.Contig1_All,
CL2835.Contig1_All, CL2835.Contig2_All, CL291.Contig1_All,
CL291.Contig2_All, CL291.Contig3_All, CL291.Contig4_All,
CL291.Contig5_All, CL291.Contig6_All, CL2910.Contig1_All,
CL2949.Contig2_All, CL295.Contig2_All, CL30.Contig2_All,
CL3002.Contig1_All, CL3037.Contig1_All,
CL3037.Contig2_All, CL3052.Contig1_All,
CL3087.Contig1_All, CL3138.Contig1_All,

			CL3138.Contig2_All, CL317.Contig9_All, CL3183.Contig1_All, CL319.Contig5_All, CL319.Contig6_All, CL3195.Contig1_All, CL3225.Contig2_All, CL3255.Contig2_All, CL3263.Contig1_All, CL3282.Contig1_All, CL3282.Contig2_All, CL33.Contig13_All, CL33.Contig14_All, CL33.Contig1_All, CL33.Contig4_All, CL33.Contig6_All, CL33.Contig9_All, CL3327.Contig1_All, CL3327.Contig2_All, CL3353.Contig2_All, CL337.Contig1_All, CL337.Contig2_All, CL337.Contig3_All, CL3371.Contig2_All, CL3381.Contig1_All, CL3384.Contig1_All, CL3391.Contig1_All, CL34.Contig10_All, CL34.Contig14_All, CL34.Contig17_All, CL34.Contig2_All, CL34.Contig3_All, CL34.Contig4_All, CL34.Contig5_All, CL34.Contig6_All, CL34.Contig9_All, CL3429.Contig1_All, CL3449.Contig1_All, CL345.Contig1_All, CL345.Contig3_All, CL345.Contig6_All, CL3454.Contig1_All, CL3464.Contig1_All, CL3469.Contig1_All, CL3469.Contig2_All, CL3482.Contig1_All, CL351.Contig2_All, CL351.Contig3_All, CL351.Contig4_All, CL351.Contig5_All, CL3518.Contig1_All, CL3518.Contig2_All, CL3518.Contig3_All, CL3533.Contig1_All, CL3548.Contig1_All, CL3558.Contig1_All, CL3558.Contig2_All, CL3562.Contig1_All, CL3563.Contig1_All, CL357.Contig4_All, CL357.Contig5_All, CL3589.Contig1_All, CL3589.Contig2_All, CL360.Contig2_All, CL360.Contig3_All, CL360.Contig4_All, CL360.Contig5_All, CL360.Contig6_All, CL360.Contig8_All, CL3602.Contig1_All, CL3613.Contig1_All, CL3650.Contig1_All, CL3683.Contig1_All, CL3691.Contig1_All, CL3748.Contig1_All, CL38.Contig13_All, CL38.Contig2_All, CL38.Contig4_All, CL38.Contig6_All, CL3835.Contig1_All, CL3835.Contig2_All, CL386.Contig1_All, CL386.Contig2_All, CL386.Contig4_All, CL386.Contig5_All, CL386.Contig6_All, CL3861.Contig1_All, CL389.Contig1_All, CL389.Contig2_All, CL389.Contig3_All, CL389.Contig4_All, CL389.Contig5_All, CL389.Contig6_All, CL389.Contig7_All, CL39.Contig11_All, CL39.Contig13_All, CL39.Contig15_All, CL39.Contig1_All, CL39.Contig7_All, CL3902.Contig1_All, CL3902.Contig2_All, CL3902.Contig3_All, CL391.Contig1_All, CL391.Contig2_All, CL391.Contig3_All, CL393.Contig1_All, CL393.Contig2_All, CL393.Contig3_All, CL393.Contig4_All, CL393.Contig5_All, CL3949.Contig1_All, CL3981.Contig1_All, CL3985.Contig2_All, CL3993.Contig1_All, CL4.Contig10_All, CL4.Contig11_All, CL4.Contig13_All, CL4.Contig21_All, CL4.Contig23_All, CL4.Contig24_All, CL4.Contig29_All, CL400.Contig1_All, CL400.Contig2_All, CL400.Contig3_All, CL400.Contig4_All, CL4047.Contig2_All, CL4085.Contig1_All, CL4087.Contig1_All, CL409.Contig1_All, CL409.Contig2_All, CL409.Contig3_All, CL409.Contig4_All, CL409.Contig5_All, CL409.Contig6_All, CL4096.Contig1_All, CL4097.Contig1_All, CL4111.Contig1_All, CL415.Contig2_All, CL415.Contig4_All, CL415.Contig5_All, CL4178.Contig1_All, CL419.Contig1_All, CL423.Contig1_All, CL423.Contig2_All, CL423.Contig3_All, CL423.Contig4_All, CL4264.Contig1_All, CL427.Contig13_All, CL4274.Contig1_All, CL4286.Contig1_All, CL433.Contig3_All, CL433.Contig5_All, CL4404.Contig1_All, CL4418.Contig1_All,

			CL4426.Contig1_All, CL4440.Contig1_All, CL4447.Contig1_All, CL4447.Contig2_All, CL4449.Contig1_All, CL445.Contig1_All, CL4467.Contig1_All, CL4496.Contig1_All, CL45.Contig10_All, CL45.Contig14_All, CL4508.Contig1_All, CL4517.Contig1_All, CL4532.Contig1_All, CL4556.Contig1_All, CL4604.Contig1_All, CL4631.Contig1_All, CL4631.Contig2_All, CL4650.Contig1_All, CL466.Contig1_All, CL466.Contig2_All, CL466.Contig3_All, CL466.Contig4_All, CL466.Contig5_All, CL4664.Contig1_All, CL4690.Contig1_All, CL4690.Contig2_All, CL4690.Contig3_All, CL4698.Contig1_All, CL4783.Contig1_All, CL4783.Contig2_All, CL4783.Contig3_All, CL480.Contig1_All, CL480.Contig4_All, CL4812.Contig1_All, CL4812.Contig2_All, CL4835.Contig1_All, CL4868.Contig1_All, CL4895.Contig1_All, CL4895.Contig2_All, CL4895.Contig3_All, CL4919.Contig1_All, CL4938.Contig1_All, CL4938.Contig2_All, CL4996.Contig1_All, CL5.Contig11_All, CL5.Contig16_All, CL5.Contig1_All, CL5.Contig23_All, CL5.Contig27_All, CL5.Contig33_All, CL5.Contig34_All, CL5.Contig7_All, CL5.Contig8_All, CL5.Contig9_All, CL5013.Contig1_All, CL5030.Contig1_All, CL5047.Contig1_All, CL5047.Contig2_All, CL5058.Contig1_All, CL5059.Contig1_All, CL5067.Contig1_All, CL5080.Contig1_All, CL5080.Contig2_All, CL5088.Contig1_All, CL5088.Contig2_All, CL5111.Contig1_All, CL5111.Contig4_All, CL5127.Contig1_All, CL5142.Contig2_All, CL5159.Contig3_All, CL5162.Contig1_All, CL5163.Contig1_All, CL5172.Contig1_All, CL5177.Contig1_All, CL52.Contig10_All, CL52.Contig1_All, CL52.Contig7_All, CL52.Contig8_All, CL5208.Contig1_All, CL5231.Contig2_All, CL5270.Contig1_All, CL5273.Contig1_All, CL5278.Contig1_All, CL529.Contig5_All, CL5293.Contig1_All, CL5298.Contig1_All, CL5353.Contig1_All, CL5358.Contig1_All, CL5374.Contig1_All, CL5383.Contig1_All, CL5383.Contig2_All, CL5387.Contig1_All, CL5387.Contig2_All, CL5396.Contig1_All, CL5417.Contig1_All, CL5428.Contig1_All, CL543.Contig1_All, CL5430.Contig1_All, CL5433.Contig1_All, CL5448.Contig1_All, CL546.Contig2_All, CL5471.Contig1_All, CL5496.Contig1_All, CL5515.Contig1_All, CL5519.Contig1_All, CL5529.Contig1_All, CL5552.Contig1_All, CL5579.Contig1_All, CL5579.Contig2_All, CL5582.Contig1_All, CL5626.Contig1_All, CL566.Contig1_All, CL566.Contig2_All, CL566.Contig3_All, CL5674.Contig1_All, CL5674.Contig2_All, CL5701.Contig1_All, CL5727.Contig1_All, CL5777.Contig1_All, CL5809.Contig1_All, CL581.Contig1_All, CL581.Contig2_All, CL581.Contig3_All, CL581.Contig4_All, CL5826.Contig1_All, CL585.Contig2_All, CL5886.Contig1_All, CL5886.Contig2_All, CL5905.Contig1_All,

			CL5909.Contig1_All, CL5923.Contig1_All, CL5967.Contig1_All, CL5991.Contig1_All, CL6020.Contig1_All, CL6038.Contig1_All, CL6055.Contig1_All, CL6055.Contig2_All, CL6084.Contig1_All, CL6086.Contig1_All, CL6103.Contig1_All, CL6119.Contig1_All, CL6125.Contig1_All, CL6125.Contig2_All, CL6126.Contig1_All, CL6127.Contig1_All, CL614.Contig1_All, CL6170.Contig1_All, CL6188.Contig1_All, CL6191.Contig1_All, CL6203.Contig1_All, CL6213.Contig1_All, CL6213.Contig2_All, CL6228.Contig2_All, CL6228.Contig4_All, CL623.Contig1_All, CL623.Contig2_All, CL623.Contig3_All, CL6230.Contig1_All, CL6266.Contig1_All, CL6297.Contig1_All, CL6343.Contig1_All, CL6363.Contig1_All, CL6366.Contig1_All, CL6387.Contig1_All, CL6409.Contig1_All, CL6413.Contig1_All, CL6420.Contig1_All, CL6437.Contig1_All, CL6448.Contig1_All, CL645.Contig1_All, CL6457.Contig1_All, CL646.Contig1_All, CL646.Contig2_All, CL6460.Contig1_All, CL6495.Contig2_All, CL6512.Contig1_All, CL6523.Contig1_All, CL6527.Contig1_All, CL653.Contig1_All, CL6549.Contig1_All, CL6590.Contig2_All, CL6590.Contig4_All, CL66.Contig5_All, CL6632.Contig1_All, CL6660.Contig1_All, CL6725.Contig1_All, CL6747.Contig1_All, CL675.Contig1_All, CL675.Contig3_All, CL675.Contig4_All, CL6756.Contig1_All, CL6780.Contig1_All, CL6793.Contig1_All, CL6811.Contig1_All, CL6841.Contig1_All, CL6888.Contig1_All, CL690.Contig3_All, CL6926.Contig1_All, CL6926.Contig2_All, CL6933.Contig1_All, CL6952.Contig1_All, CL696.Contig1_All, CL696.Contig2_All, CL696.Contig3_All, CL696.Contig4_All, CL696.Contig5_All, CL696.Contig6_All, CL696.Contig7_All, CL696.Contig8_All, CL6974.Contig1_All, CL7015.Contig1_All, CL7109.Contig1_All, CL7147.Contig1_All, CL7148.Contig1_All, CL72.Contig11_All, CL72.Contig12_All, CL72.Contig14_All, CL72.Contig15_All, CL72.Contig2_All, CL72.Contig3_All, CL72.Contig7_All, CL72.Contig9_All, CL7272.Contig1_All, CL7283.Contig1_All, CL730.Contig1_All, CL730.Contig2_All, CL730.Contig3_All, CL730.Contig4_All, CL7302.Contig1_All, CL7343.Contig1_All, CL7345.Contig1_All, CL7393.Contig1_All, CL7413.Contig1_All, CL7421.Contig1_All, CL7451.Contig1_All, CL7470.Contig1_All, CL7483.Contig1_All, CL7483.Contig2_All, CL7486.Contig1_All, CL7496.Contig1_All, CL7535.Contig1_All, CL7542.Contig1_All, CL7546.Contig1_All, CL7692.Contig1_All, CL7720.Contig1_All, CL778.Contig6_All, CL782.Contig1_All, CL7821.Contig1_All, CL7827.Contig1_All, CL7842.Contig1_All, CL7857.Contig1_All, CL7867.Contig1_All, CL7867.Contig2_All, CL788.Contig1_All, CL788.Contig2_All, CL7887.Contig1_All, CL7892.Contig1_All, CL79.Contig2_All, CL79.Contig3_All, CL79.Contig5_All,

			CL79.Contig7_All, CL7928.Contig1_All, CL7952.Contig1_All, CL7969.Contig1_All, CL8021.Contig1_All, CL8036.Contig1_All, CL8051.Contig1_All, CL8074.Contig1_All, CL809.Contig1_All, CL809.Contig2_All, CL809.Contig3_All, CL8146.Contig1_All, CL815.Contig3_All, CL8150.Contig1_All, CL8152.Contig1_All, CL8162.Contig1_All, CL8181.Contig1_All, CL8196.Contig1_All, CL8202.Contig1_All, CL8208.Contig1_All, CL8248.Contig1_All, CL8292.Contig1_All, CL8305.Contig1_All, CL8328.Contig1_All, CL8354.Contig2_All, CL8359.Contig1_All, CL8427.Contig1_All, CL843.Contig1_All, CL843.Contig2_All, CL843.Contig3_All, CL8497.Contig1_All, CL8501.Contig1_All, CL8506.Contig1_All, CL8518.Contig1_All, CL8519.Contig1_All, CL854.Contig1_All, CL8554.Contig1_All, CL858.Contig1_All, CL8594.Contig1_All, CL8631.Contig1_All, CL864.Contig2_All, CL8647.Contig1_All, CL8652.Contig1_All, CL8682.Contig1_All, CL87.Contig1_All, CL870.Contig1_All, CL871.Contig1_All, CL871.Contig2_All, CL8775.Contig1_All, CL8780.Contig1_All, CL8782.Contig1_All, CL8812.Contig1_All, CL8829.Contig1_All, CL8842.Contig1_All, CL8851.Contig1_All, CL8868.Contig1_All, CL8886.Contig1_All, CL89.Contig1_All, CL89.Contig5_All, CL89.Contig6_All, CL8910.Contig1_All, CL8955.Contig1_All, CL8967.Contig1_All, CL8967.Contig2_All, CL90.Contig1_All, CL9000.Contig1_All, CL9043.Contig1_All, CL9056.Contig1_All, CL9074.Contig1_All, CL91.Contig4_All, CL9110.Contig1_All, CL9115.Contig1_All, CL9115.Contig2_All, CL9196.Contig1_All, CL9249.Contig1_All, CL9256.Contig1_All, CL9297.Contig1_All, CL9331.Contig1_All, CL9331.Contig2_All, CL9336.Contig1_All, CL9362.Contig1_All, CL9377.Contig1_All, CL9453.Contig1_All, CL9482.Contig1_All, CL958.Contig1_All, CL958.Contig2_All, CL96.Contig2_All, CL96.Contig5_All, CL9607.Contig1_All, CL9622.Contig1_All, CL9636.Contig1_All, CL970.Contig1_All, CL970.Contig2_All, CL9706.Contig1_All, CL971.Contig1_All, CL9717.Contig1_All, CL9718.Contig1_All, CL9752.Contig1_All, CL9752.Contig2_All, CL9766.Contig1_All, CL977.Contig1_All, CL977.Contig2_All, CL977.Contig3_All, CL977.Contig4_All, CL9774.Contig1_All, CL9774.Contig2_All, CL9785.Contig1_All, CL9815.Contig1_All, CL9825.Contig1_All, CL9845.Contig1_All, CL9856.Contig1_All, CL995.Contig1_All, CL995.Contig2_All, CL995.Contig3_All, CL9968.Contig1_All, CL9983.Contig1_All, CL9997.Contig1_All, Unigene1044_All, Unigene1049_All, Unigene119_All, Unigene1263_All, Unigene1304_All, Unigene1333_All, Unigene1355_All, Unigene136_All, Unigene1382_All, Unigene1388_All, Unigene1410_All, Unigene1545_All, Unigene1726_All, Unigene1739_All, Unigene176_All, Unigene1803_All, Unigene1854_All, Unigene1921_All, Unigene1959_All, Unigene2134_All,

		Unigene2314_All, Unigene2482_All, Unigene2495_All, Unigene2512_All, Unigene2664_All, Unigene2701_All, Unigene2886_All, Unigene2946_All, Unigene2987_All, Unigene2996_All, Unigene3062_All, Unigene3080_All, Unigene3143_All, Unigene322_All, Unigene3296_All, Unigene3343_All, Unigene3369_All, Unigene3383_All, Unigene3472_All, Unigene3573_All, Unigene3581_All, Unigene3582_All, Unigene3690_All, Unigene374_All, Unigene3858_All, Unigene3927_All, Unigene3966_All, Unigene4006_All, Unigene4014_All, Unigene406_All, Unigene4085_All, Unigene4144_All, Unigene4228_All, Unigene4270_All, Unigene4347_All, Unigene4350_All, Unigene4375_All, Unigene4470_All, Unigene4490_All, Unigene4588_All, Unigene4595_All, Unigene4623_All, Unigene4648_All, Unigene4666_All, Unigene4675_All, Unigene4787_All, Unigene4812_All, Unigene481_All, Unigene4836_All, Unigene4844_All, Unigene4850_All, Unigene4885_All, Unigene4895_All, Unigene48_All, Unigene4950_All, Unigene4966_All, Unigene5055_All, Unigene5062_All, Unigene5098_All, Unigene5164_All, Unigene5172_All, Unigene5174_All, Unigene5215_All, Unigene5225_All, Unigene5265_All, Unigene5267_All, Unigene5270_All, Unigene5278_All, Unigene5306_All, Unigene533_All, Unigene5379_All, Unigene546_All, Unigene5478_All, Unigene5521_All, Unigene553_All, Unigene561_All, Unigene578_All, Unigene636_All, Unigene651_All, Unigene671_All, Unigene722_All, Unigene867_All, Unigene918_All, Unigene93_All, Unigene941_All
5		CL1.Contig127_All, CL1.Contig141_All, CL1.Contig92_All, CL10037.Contig1_All, CL10066.Contig1_All, CL10080.Contig1_All, CL10103.Contig1_All, CL10209.Contig1_All, CL10249.Contig1_All, CL10253.Contig1_All, CL10264.Contig1_All, CL10275.Contig1_All, CL10285.Contig1_All, CL10367.Contig1_All, CL10405.Contig1_All, CL10432.Contig1_All, CL10466.Contig1_All, CL10478.Contig1_All, CL105.Contig10_All, CL105.Contig11_All, CL105.Contig4_All, CL105.Contig6_All, CL105.Contig8_All, CL10522.Contig1_All, CL10549.Contig1_All, CL1058.Contig1_All, CL1058.Contig2_All, CL1058.Contig3_All, CL10619.Contig1_All, CL10735.Contig1_All, CL10796.Contig1_All, CL10903.Contig1_All, CL10912.Contig1_All, CL1094.Contig1_All, CL10976.Contig1_All, CL1098.Contig1_All, CL1098.Contig2_All, CL1098.Contig3_All, CL1098.Contig4_All, CL11012.Contig1_All, CL11055.Contig1_All, CL11055.Contig2_All, CL11206.Contig1_All, CL11346.Contig1_All, CL11373.Contig1_All, CL11381.Contig1_All, CL11455.Contig1_All, CL11478.Contig1_All, CL115.Contig2_All, CL11517.Contig1_All, CL11588.Contig1_All, CL11616.Contig1_All,

	RNA transport	CL11618.Contig1_All, CL11644.Contig1_All, CL11694.Contig1_All, CL1170.Contig1_All, CL11708.Contig1_All, CL11721.Contig1_All, CL11780.Contig1_All, CL11830.Contig1_All, CL11860.Contig1_All, CL11885.Contig1_All, CL11980.Contig1_All, CL12.Contig12_All, CL12010.Contig1_All, CL1208.Contig1_All, CL12169.Contig1_All, CL1219.Contig1_All, CL1219.Contig2_All, CL1224.Contig1_All, CL1225.Contig1_All, CL1225.Contig2_All, CL12425.Contig1_All, CL125.Contig3_All, CL125.Contig4_All, CL125.Contig5_All, CL125.Contig6_All, CL125.Contig7_All, CL12595.Contig1_All, CL12650.Contig1_All, CL12679.Contig1_All, CL12736.Contig1_All, CL12786.Contig1_All, CL12825.Contig1_All, CL1290.Contig1_All, CL1290.Contig2_All, CL1295.Contig1_All, CL12960.Contig1_All, CL1299.Contig1_All, CL1299.Contig2_All, CL1305.Contig1_All, CL1308.Contig1_All, CL13204.Contig1_All, CL13248.Contig2_All, CL13296.Contig1_All, CL13361.Contig1_All, CL13369.Contig1_All, CL13425.Contig1_All, CL135.Contig1_All, CL135.Contig2_All, CL135.Contig3_All, CL1357.Contig1_All, CL13570.Contig1_All, CL13611.Contig1_All, CL13624.Contig1_All, CL13673.Contig1_All, CL13697.Contig1_All, CL13768.Contig1_All, CL13795.Contig1_All, CL1382.Contig1_All, CL13854.Contig1_All, CL1387.Contig1_All, CL13885.Contig1_All, CL139.Contig1_All, CL139.Contig4_All, CL139.Contig6_All, CL139.Contig7_All, CL13996.Contig1_All, CL14085.Contig1_All, CL14127.Contig1_All, CL14161.Contig1_All, CL14232.Contig1_All, CL14288.Contig1_All, CL144.Contig4_All, CL144.Contig6_All, CL144.Contig7_All, CL144.Contig8_All, CL14413.Contig1_All, CL14430.Contig1_All, CL1451.Contig2_All, CL14620.Contig1_All, CL14622.Contig1_All, CL1463.Contig1_All, CL1463.Contig2_All, CL1463.Contig3_All, CL1463.Contig4_All, CL1463.Contig5_All, CL14649.Contig1_All, CL14695.Contig1_All, CL1475.Contig2_All, CL14830.Contig1_All, CL14870.Contig1_All, CL14928.Contig1_All, CL14948.Contig1_All, CL150.Contig7_All, CL15033.Contig1_All, CL15076.Contig1_All, CL15083.Contig1_All, CL1512.Contig1_All, CL15150.Contig1_All, CL15346.Contig1_All, CL15371.Contig1_All, CL15499.Contig1_All, CL15559.Contig1_All, CL15744.Contig1_All, CL15775.Contig1_All, CL15824.Contig1_All, CL1584.Contig1_All, CL15895.Contig1_All, CL15981.Contig1_All, CL15987.Contig1_All, CL1600.Contig1_All, CL16039.Contig1_All, CL16062.Contig1_All, CL16131.Contig1_All, CL16181.Contig1_All,

		CL16229.Contig1_All, CL16262.Contig1_All, CL1629.Contig1_All, CL16333.Contig1_All, CL1636.Contig1_All, CL16520.Contig1_All, CL16533.Contig1_All, CL16548.Contig1_All, CL1672.Contig2_All, CL16819.Contig1_All, CL1685.Contig1_All, CL1691.Contig1_All, CL16952.Contig1_All, CL16986.Contig1_All, CL17.Contig39_All, CL17.Contig42_All, CL17088.Contig1_All, CL1712.Contig1_All, CL1712.Contig2_All, CL17142.Contig1_All, CL17175.Contig1_All, CL1720.Contig1_All, CL17286.Contig1_All, CL1732.Contig4_All, CL1733.Contig1_All, CL17380.Contig1_All, CL17422.Contig1_All, CL17426.Contig1_All, CL17430.Contig1_All, CL17457.Contig1_All, CL17461.Contig1_All, CL17514.Contig1_All, CL17527.Contig1_All, CL17534.Contig1_All, CL176.Contig1_All, CL1761.Contig1_All, CL17611.Contig1_All, CL17619.Contig1_All, CL17628.Contig1_All, CL17661.Contig1_All, CL1767.Contig1_All, CL1767.Contig2_All, CL17792.Contig1_All, CL17798.Contig1_All, CL17802.Contig1_All, CL1786.Contig1_All, CL1786.Contig2_All, CL17964.Contig1_All, CL18114.Contig1_All, CL182.Contig6_All, CL18208.Contig1_All, CL18223.Contig1_All, CL1823.Contig1_All, CL1823.Contig2_All, CL18232.Contig1_All, CL18256.Contig1_All, CL18308.Contig1_All, CL18326.Contig1_All, CL18388.Contig1_All, CL18478.Contig1_All, CL18507.Contig1_All, CL18551.Contig1_All, CL18592.Contig1_All, CL18603.Contig1_All, CL18608.Contig1_All, CL18721.Contig1_All, CL18764.Contig1_All, CL18772.Contig1_All, CL18835.Contig1_All, CL1886.Contig1_All, CL19053.Contig1_All, CL19080.Contig1_All, CL19094.Contig1_All, CL19139.Contig1_All, CL1920.Contig1_All, CL1923.Contig1_All, CL19274.Contig1_All, CL19317.Contig1_All, CL19383.Contig1_All, CL194.Contig1_All, CL19427.Contig1_All, CL19464.Contig1_All, CL195.Contig1_All, CL195.Contig2_All, CL195.Contig3_All, CL195.Contig4_All, CL195.Contig5_All, CL1953.Contig1_All, CL196.Contig3_All, CL196.Contig6_All, CL196.Contig8_All, CL1965.Contig1_All, CL19684.Contig1_All, CL1971.Contig1_All, CL1971.Contig2_All, CL19715.Contig1_All, CL19748.Contig1_All, CL19754.Contig1_All, CL19962.Contig1_All, CL2011.Contig1_All, CL20230.Contig1_All, CL20239.Contig1_All, CL2036.Contig1_All, CL20451.Contig1_All, CL20493.Contig1_All, CL20610.Contig1_All, CL20647.Contig1_All, CL2073.Contig1_All, CL20971.Contig1_All, CL21005.Contig1_All, CL2103.Contig2_All, CL2103.Contig3_All,

			CL21075.Contig1_All, CL2109.Contig1_All, CL21194.Contig1_All, CL21208.Contig1_All, CL21317.Contig1_All, CL21367.Contig1_All, CL21418.Contig1_All, CL21441.Contig1_All, CL2145.Contig1_All, CL21454.Contig1_All, CL21546.Contig1_All, CL21752.Contig1_All, CL21797.Contig1_All, CL2186.Contig1_All, CL21895.Contig1_All, CL22127.Contig1_All, CL2215.Contig1_All, CL2232.Contig1_All, CL22422.Contig1_All, CL22459.Contig1_All, CL2249.Contig1_All, CL22526.Contig1_All, CL2257.Contig1_All, CL22625.Contig1_All, CL22687.Contig1_All, CL227.Contig1_All, CL227.Contig5_All, CL22719.Contig1_All, CL22749.Contig1_All, CL2275.Contig1_All, CL2275.Contig2_All, CL229.Contig2_All, CL22907.Contig1_All, CL2291.Contig1_All, CL22939.Contig1_All, CL22991.Contig1_All, CL23090.Contig1_All, CL23092.Contig1_All, CL23139.Contig1_All, CL23142.Contig1_All, CL23162.Contig1_All, CL23175.Contig1_All, CL23183.Contig1_All, CL2330.Contig1_All, CL2330.Contig2_All, CL23322.Contig1_All, CL23434.Contig1_All, CL2353.Contig1_All, CL2353.Contig3_All, CL2353.Contig4_All, CL23586.Contig1_All, CL23602.Contig1_All, CL23706.Contig1_All, CL2376.Contig1_All, CL23790.Contig1_All, CL23869.Contig1_All, CL23892.Contig1_All, CL240.Contig2_All, CL240.Contig5_All, CL24008.Contig1_All, CL24281.Contig1_All, CL243.Contig2_All, CL243.Contig3_All, CL243.Contig4_All, CL243.Contig5_All, CL2440.Contig1_All, CL24401.Contig1_All, CL246.Contig1_All, CL246.Contig2_All, CL246.Contig3_All, CL246.Contig4_All, CL24721.Contig1_All, CL24798.Contig1_All, CL24872.Contig1_All, CL24898.Contig1_All, CL24924.Contig1_All, CL2497.Contig1_All, CL25009.Contig1_All, CL2511.Contig1_All, CL2511.Contig2_All, CL2511.Contig3_All, CL252.Contig1_All, CL252.Contig6_All, CL2521.Contig1_All, CL25220.Contig1_All, CL25224.Contig1_All, CL2539.Contig1_All, CL25396.Contig1_All, CL2570.Contig1_All, CL2570.Contig2_All, CL25789.Contig1_All, CL25876.Contig1_All, CL25883.Contig1_All, CL26.Contig3_All, CL26.Contig6_All, CL2601.Contig1_All, CL26062.Contig1_All, CL261.Contig2_All, CL2615.Contig1_All, CL2616.Contig1_All, CL26186.Contig1_All, CL26235.Contig1_All, CL26262.Contig1_All, CL26408.Contig1_All, CL26468.Contig1_All, CL26487.Contig1_All, CL26539.Contig1_All, CL26578.Contig1_All, CL2658.Contig1_All, CL26587.Contig1_All, CL2661.Contig1_All, CL26651.Contig1_All, CL26684.Contig1_All, CL26710.Contig1_All, CL2673.Contig1_All, CL26758.Contig1_All,

			CL2679.Contig1_All, CL26810.Contig1_All, CL26871.Contig1_All, CL2691.Contig1_All, CL26910.Contig1_All, CL26935.Contig1_All, CL27151.Contig1_All, CL27224.Contig1_All, CL27271.Contig1_All, CL27286.Contig1_All, CL27345.Contig1_All, CL2741.Contig1_All, CL27443.Contig1_All, CL27451.Contig1_All, CL27517.Contig1_All, CL27553.Contig1_All, CL2756.Contig1_All, CL2756.Contig2_All, CL2767.Contig1_All, CL27783.Contig1_All, CL27802.Contig1_All, CL2784.Contig1_All, CL27897.Contig1_All, CL27913.Contig1_All, CL28049.Contig1_All, CL2808.Contig1_All, CL28114.Contig1_All, CL282.Contig1_All, CL282.Contig2_All, CL282.Contig3_All, CL282.Contig5_All, CL282.Contig6_All, CL282.Contig7_All, CL288.Contig3_All, CL288.Contig4_All, CL288.Contig5_All, CL288.Contig8_All, CL2892.Contig1_All, CL290.Contig6_All, CL2918.Contig1_All, CL292.Contig1_All, CL292.Contig2_All, CL292.Contig3_All, CL292.Contig4_All, CL292.Contig5_All, CL2951.Contig1_All, CL2951.Contig2_All, CL2984.Contig2_All, CL3006.Contig1_All, CL3038.Contig1_All, CL3038.Contig2_All, CL306.Contig1_All, CL306.Contig2_All, CL306.Contig3_All, CL3081.Contig1_All, CL3081.Contig2_All, CL3081.Contig3_All, CL3081.Contig4_All, CL3084.Contig1_All, CL309.Contig1_All, CL3105.Contig2_All, CL3119.Contig1_All, CL3168.Contig1_All, CL3171.Contig1_All, CL3188.Contig2_All, CL3207.Contig1_All, CL3277.Contig1_All, CL3332.Contig1_All, CL3372.Contig1_All, CL3389.Contig1_All, CL3417.Contig1_All, CL3419.Contig1_All, CL3440.Contig1_All, CL3461.Contig1_All, CL3467.Contig1_All, CL3472.Contig1_All, CL3480.Contig1_All, CL351.Contig2_All, CL351.Contig7_All, CL3552.Contig1_All, CL3559.Contig1_All, CL3578.Contig1_All, CL3578.Contig2_All, CL3578.Contig3_All, CL3600.Contig1_All, CL3612.Contig1_All, CL364.Contig2_All, CL366.Contig5_All, CL3679.Contig1_All, CL3693.Contig1_All, CL3693.Contig2_All, CL3694.Contig1_All, CL37.Contig10_All, CL37.Contig12_All, CL37.Contig13_All, CL37.Contig14_All, CL37.Contig15_All, CL37.Contig16_All, CL37.Contig17_All, CL37.Contig1_All, CL37.Contig2_All, CL37.Contig3_All, CL37.Contig4_All, CL37.Contig5_All, CL37.Contig6_All, CL37.Contig7_All, CL37.Contig8_All, CL37.Contig9_All, CL379.Contig1_All, CL3792.Contig2_All, CL3797.Contig1_All, CL3823.Contig1_All, CL3856.Contig1_All, CL3917.Contig1_All, CL3929.Contig1_All, CL3930.Contig1_All, CL3979.Contig2_All, CL4.Contig29_All, CL4.Contig9_All, CL4007.Contig1_All, CL414.Contig3_All, CL414.Contig4_All, CL414.Contig7_All, CL4146.Contig1_All, CL4225.Contig1_All, CL4228.Contig1_All, CL4228.Contig2_All, CL4228.Contig3_All, CL4267.Contig2_All, CL4290.Contig1_All,

			CL4297.Contig1_All, CL43.Contig3_All, CL43.Contig9_All, CL4329.Contig1_All, CL4450.Contig1_All, CL4482.Contig1_All, CL449.Contig3_All, CL45.Contig12_All, CL45.Contig13_All, CL45.Contig1_All, CL45.Contig2_All, CL45.Contig3_All, CL4501.Contig2_All, CL4565.Contig2_All, CL458.Contig3_All, CL4581.Contig1_All, CL4587.Contig2_All, CL4602.Contig1_All, CL4618.Contig1_All, CL4663.Contig1_All, CL4718.Contig1_All, CL4722.Contig1_All, CL4748.Contig1_All, CL4796.Contig1_All, CL4810.Contig1_All, CL4831.Contig1_All, CL4882.Contig1_All, CL4889.Contig1_All, CL492.Contig1_All, CL4924.Contig1_All, CL4939.Contig1_All, CL4959.Contig1_All, CL496.Contig2_All, CL5.Contig30_All, CL5014.Contig1_All, CL5027.Contig1_All, CL5040.Contig1_All, CL5061.Contig1_All, CL5076.Contig1_All, CL5114.Contig1_All, CL5118.Contig1_All, CL513.Contig1_All, CL5131.Contig1_All, CL5149.Contig1_All, CL516.Contig3_All, CL5194.Contig1_All, CL5195.Contig1_All, CL5231.Contig1_All, CL5231.Contig2_All, CL5249.Contig1_All, CL525.Contig5_All, CL5313.Contig1_All, CL532.Contig1_All, CL5323.Contig1_All, CL5344.Contig1_All, CL5354.Contig1_All, CL5438.Contig1_All, CL5454.Contig1_All, CL5481.Contig1_All, CL5524.Contig1_All, CL5524.Contig2_All, CL5524.Contig3_All, CL553.Contig1_All, CL5530.Contig1_All, CL5530.Contig2_All, CL5533.Contig1_All, CL554.Contig1_All, CL563.Contig1_All, CL5647.Contig1_All, CL570.Contig1_All, CL5713.Contig2_All, CL5732.Contig1_All, CL574.Contig1_All, CL574.Contig2_All, CL5740.Contig1_All, CL5879.Contig1_All, CL5880.Contig1_All, CL5896.Contig1_All, CL60.Contig7_All, CL6015.Contig1_All, CL6070.Contig1_All, CL6088.Contig1_All, CL6107.Contig2_All, CL6123.Contig1_All, CL6131.Contig1_All, CL6149.Contig1_All, CL6157.Contig1_All, CL6186.Contig1_All, CL628.Contig1_All, CL6386.Contig1_All, CL6393.Contig1_All, CL6476.Contig1_All, CL6484.Contig1_All, CL6498.Contig1_All, CL6518.Contig1_All, CL6520.Contig1_All, CL6542.Contig1_All, CL6575.Contig1_All, CL6619.Contig1_All, CL6687.Contig1_All, CL6697.Contig1_All, CL6723.Contig1_All, CL6723.Contig2_All, CL6726.Contig1_All, CL673.Contig1_All, CL673.Contig2_All, CL673.Contig3_All, CL673.Contig5_All, CL6741.Contig1_All, CL6768.Contig1_All, CL6768.Contig2_All, CL6768.Contig3_All, CL6779.Contig1_All, CL6808.Contig1_All, CL6833.Contig1_All, CL6887.Contig1_All, CL6959.Contig1_All, CL6983.Contig1_All, CL7.Contig23_All, CL7.Contig5_All, CL7004.Contig1_All, CL7004.Contig2_All, CL7033.Contig1_All, CL7037.Contig1_All, CL7050.Contig1_All, CL7067.Contig1_All, CL7153.Contig1_All, CL7164.Contig1_All, CL719.Contig5_All, CL719.Contig6_All, CL719.Contig8_All, CL7200.Contig1_All,

			CL7206.Contig1_All, CL7237.Contig1_All, CL7264.Contig1_All, CL7330.Contig1_All, CL740.Contig3_All, CL7402.Contig1_All, CL7410.Contig1_All, CL7410.Contig2_All, CL7455.Contig1_All, CL7505.Contig1_All, CL7552.Contig1_All, CL7572.Contig1_All, CL7640.Contig1_All, CL7640.Contig2_All, CL7654.Contig1_All, CL7661.Contig1_All, CL7671.Contig1_All, CL7690.Contig1_All, CL7795.Contig1_All, CL78.Contig11_All, CL787.Contig1_All, CL7871.Contig1_All, CL791.Contig3_All, CL791.Contig4_All, CL792.Contig1_All, CL795.Contig1_All, CL795.Contig2_All, CL795.Contig3_All, CL795.Contig4_All, CL7957.Contig1_All, CL7962.Contig1_All, CL7962.Contig2_All, CL7972.Contig1_All, CL7986.Contig1_All, CL80.Contig4_All, CL80.Contig7_All, CL8034.Contig1_All, CL8065.Contig1_All, CL8164.Contig1_All, CL8224.Contig1_All, CL8302.Contig1_All, CL8306.Contig1_All, CL8329.Contig1_All, CL835.Contig1_All, CL835.Contig2_All, CL8387.Contig1_All, CL840.Contig4_All, CL8411.Contig1_All, CL8428.Contig1_All, CL8467.Contig1_All, CL8473.Contig2_All, CL8541.Contig1_All, CL8547.Contig2_All, CL8553.Contig1_All, CL8623.Contig1_All, CL8627.Contig1_All, CL865.Contig1_All, CL865.Contig2_All, CL8676.Contig1_All, CL8726.Contig1_All, CL876.Contig1_All, CL876.Contig4_All, CL8785.Contig1_All, CL8802.Contig1_All, CL883.Contig1_All, CL8844.Contig1_All, CL8895.Contig1_All, CL8895.Contig2_All, CL9005.Contig1_All, CL9227.Contig1_All, CL928.Contig1_All, CL928.Contig3_All, CL93.Contig2_All, CL93.Contig3_All, CL93.Contig4_All, CL93.Contig5_All, CL93.Contig6_All, CL93.Contig7_All, CL9303.Contig1_All, CL9318.Contig1_All, CL9318.Contig2_All, CL937.Contig2_All, CL94.Contig7_All, CL9486.Contig1_All, CL9529.Contig1_All, CL9605.Contig1_All, CL9623.Contig1_All, CL9641.Contig1_All, CL966.Contig1_All, CL966.Contig2_All, CL974.Contig1_All, CL974.Contig2_All, CL974.Contig3_All, CL9834.Contig1_All, CL9883.Contig1_All, CL9895.Contig1_All, Unigene1058_All, Unigene1059_All, Unigene1068_All, Unigene1092_All, Unigene1094_All, Unigene1100_All, Unigene1150_All, Unigene1194_All, Unigene1272_All, Unigene1273_All, Unigene1279_All, Unigene1311_All, Unigene1312_All, Unigene1455_All, Unigene1534_All, Unigene1549_All, Unigene1597_All, Unigene1607_All, Unigene1609_All, Unigene1682_All, Unigene1723_All, Unigene1733_All, Unigene1735_All, Unigene1780_All, Unigene1824_All, Unigene1825_All, Unigene1929_All, Unigene1944_All, Unigene201_All, Unigene202_All, Unigene2092_All, Unigene2105_All, Unigene2183_All, Unigene2370_All, Unigene2656_All, Unigene2658_All, Unigene2716_All, Unigene2749_All, Unigene2968_All, Unigene2979_All, Unigene3036_All, Unigene3073_All, Unigene3270_All, Unigene3353_All, Unigene3362_All, Unigene3411_All, Unigene3480_All,

		Unigene3565_All, Unigene3571_All, Unigene3787_All, Unigene3791_All, Unigene3807_All, Unigene3842_All, Unigene4008_All, Unigene4107_All, Unigene4284_All, Unigene4434_All, Unigene4441_All, Unigene4488_All, Unigene4611_All, Unigene4688_All, Unigene4746_All, Unigene5067_All, Unigene5307_All, Unigene5378_All, Unigene5475_All, Unigene547_All, Unigene668_All, Unigene836_All, Unigene865_All, Unigene873_All, Unigene889_All
6		CL1.Contig101_All, CL1.Contig142_All, CL1.Contig144_All, CL1.Contig56_All, CL1.Contig57_All, CL10023.Contig1_All, CL10028.Contig1_All, CL10033.Contig1_All, CL10039.Contig1_All, CL10052.Contig1_All, CL10061.Contig1_All, CL10069.Contig1_All, CL10080.Contig1_All, CL10095.Contig1_All, CL10103.Contig1_All, CL10243.Contig1_All, CL10249.Contig1_All, CL10267.Contig1_All, CL10307.Contig2_All, CL1031.Contig1_All, CL1031.Contig2_All, CL1031.Contig3_All, CL1031.Contig4_All, CL1031.Contig5_All, CL10381.Contig1_All, CL10423.Contig1_All, CL10429.Contig1_All, CL10479.Contig1_All, CL10500.Contig1_All, CL10545.Contig1_All, CL10648.Contig1_All, CL10649.Contig1_All, CL10664.Contig1_All, CL1068.Contig1_All, CL10692.Contig1_All, CL10714.Contig1_All, CL10757.Contig1_All, CL10771.Contig1_All, CL1080.Contig1_All, CL1080.Contig2_All, CL10815.Contig1_All, CL10858.Contig1_All, CL10862.Contig1_All, CL10876.Contig1_All, CL10917.Contig1_All, CL1098.Contig1_All, CL1098.Contig2_All, CL1098.Contig3_All, CL1098.Contig4_All, CL10984.Contig1_All, CL1105.Contig1_All, CL11055.Contig1_All, CL11055.Contig2_All, CL11079.Contig1_All, CL11102.Contig1_All, CL11126.Contig1_All, CL1114.Contig1_All, CL1114.Contig2_All, CL1114.Contig3_All, CL1114.Contig4_All, CL1114.Contig5_All, CL11240.Contig1_All, CL1128.Contig1_All, CL1128.Contig2_All, CL1130.Contig1_All, CL1130.Contig2_All, CL1130.Contig3_All, CL1130.Contig4_All, CL1130.Contig5_All, CL11384.Contig1_All, CL1148.Contig1_All, CL1148.Contig3_All, CL1148.Contig4_All, CL115.Contig1_All, CL115.Contig2_All, CL115.Contig4_All, CL11534.Contig1_All, CL11610.Contig1_All, CL11638.Contig1_All, CL1164.Contig1_All, CL11642.Contig1_All, CL11658.Contig1_All, CL11674.Contig1_All, CL11693.Contig1_All, CL11708.Contig1_All, CL11757.Contig1_All, CL11781.Contig1_All, CL11853.Contig1_All, CL11943.Contig1_All, CL1195.Contig1_All, CL12012.Contig1_All, CL1208.Contig1_All, CL12149.Contig1_All,

	Spliceosome	CL1219.Contig1_All, CL1219.Contig2_All, CL12256.Contig1_All, CL12358.Contig1_All, CL12403.Contig1_All, CL12407.Contig1_All, CL12450.Contig1_All, CL12595.Contig1_All, CL1263.Contig1_All, CL1263.Contig2_All, CL12696.Contig1_All, CL1271.Contig1_All, CL1271.Contig2_All, CL1271.Contig3_All, CL1273.Contig1_All, CL1273.Contig2_All, CL12786.Contig1_All, CL12799.Contig1_All, CL1289.Contig1_All, CL1289.Contig2_All, CL1289.Contig3_All, CL12976.Contig1_All, CL13041.Contig1_All, CL13048.Contig1_All, CL13064.Contig1_All, CL13111.Contig1_All, CL13115.Contig1_All, CL13117.Contig1_All, CL1317.Contig1_All, CL1317.Contig3_All, CL13186.Contig1_All, CL13187.Contig1_All, CL13216.Contig1_All, CL13219.Contig1_All, CL13247.Contig1_All, CL13255.Contig1_All, CL13281.Contig1_All, CL13285.Contig1_All, CL13294.Contig1_All, CL13340.Contig1_All, CL13351.Contig1_All, CL13355.Contig1_All, CL13369.Contig1_All, CL13402.Contig1_All, CL135.Contig1_All, CL135.Contig2_All, CL13519.Contig1_All, CL13556.Contig1_All, CL13610.Contig1_All, CL13624.Contig1_All, CL13644.Contig1_All, CL13747.Contig1_All, CL13793.Contig1_All, CL1383.Contig1_All, CL13845.Contig1_All, CL13848.Contig1_All, CL13893.Contig1_All, CL139.Contig2_All, CL13904.Contig1_All, CL13933.Contig1_All, CL1394.Contig1_All, CL13968.Contig1_All, CL13976.Contig1_All, CL13984.Contig1_All, CL13992.Contig1_All, CL14000.Contig1_All, CL14000.Contig2_All, CL14053.Contig1_All, CL1406.Contig3_All, CL14061.Contig1_All, CL14103.Contig1_All, CL14129.Contig1_All, CL14178.Contig1_All, CL14275.Contig1_All, CL14310.Contig1_All, CL14317.Contig1_All, CL14322.Contig1_All, CL14378.Contig1_All, CL14419.Contig1_All, CL14479.Contig1_All, CL14525.Contig1_All, CL14577.Contig1_All, CL14668.Contig1_All, CL14673.Contig1_All, CL14735.Contig1_All, CL14739.Contig1_All, CL14830.Contig1_All, CL14870.Contig1_All, CL15002.Contig1_All, CL151.Contig2_All, CL151.Contig4_All, CL151.Contig8_All, CL151.Contig9_All, CL1512.Contig1_All, CL15187.Contig1_All, CL15193.Contig1_All, CL15227.Contig1_All, CL15268.Contig1_All, CL15274.Contig1_All, CL15320.Contig1_All, CL15328.Contig1_All, CL15346.Contig1_All, CL15348.Contig1_All, CL15348.Contig2_All, CL15351.Contig1_All, CL15370.Contig1_All, CL15444.Contig1_All, CL1546.Contig1_All, CL15461.Contig1_All, CL15513.Contig1_All,

CL15553.Contig1_All, CL15559.Contig1_All,
CL15576.Contig1_All, CL15704.Contig1_All,
CL15744.Contig1_All, CL15745.Contig1_All,
CL15775.Contig1_All, CL15804.Contig1_All,
CL15808.Contig1_All, CL15919.Contig1_All,
CL15926.Contig1_All, CL16120.Contig1_All,
CL16229.Contig1_All, CL1630.Contig2_All,
CL16467.Contig1_All, CL16484.Contig1_All,
CL16548.Contig1_All, CL16576.Contig1_All,
CL16621.Contig1_All, CL16697.Contig1_All,
CL1677.Contig1_All, CL1677.Contig4_All,
CL16774.Contig1_All, CL16851.Contig1_All,
CL169.Contig1_All, CL169.Contig2_All, CL169.Contig3_All,
CL169.Contig4_All, CL169.Contig5_All, CL169.Contig6_All,
CL169.Contig7_All, CL169.Contig8_All, CL169.Contig9_All,
CL16913.Contig1_All, CL16952.Contig1_All,
CL16994.Contig1_All, CL16996.Contig1_All,
CL1700.Contig1_All, CL17043.Contig1_All,
CL1708.Contig1_All, CL17088.Contig1_All,
CL17099.Contig1_All, CL17153.Contig1_All,
CL17157.Contig1_All, CL17229.Contig1_All,
CL17240.Contig1_All, CL17245.Contig1_All,
CL17278.Contig1_All, CL17278.Contig2_All,
CL17284.Contig1_All, CL17286.Contig1_All,
CL17430.Contig1_All, CL17454.Contig1_All,
CL17507.Contig1_All, CL17524.Contig1_All,
CL17575.Contig1_All, CL17578.Contig1_All,
CL1767.Contig1_All, CL1767.Contig2_All,
CL17680.Contig1_All, CL1769.Contig1_All,
CL17792.Contig1_All, CL1785.Contig1_All,
CL17910.Contig1_All, CL17941.Contig1_All,
CL1799.Contig2_All, CL17997.Contig1_All,
CL1807.Contig1_All, CL1809.Contig1_All,
CL18102.Contig1_All, CL18135.Contig1_All,
CL18163.Contig1_All, CL18174.Contig1_All,
CL18208.Contig1_All, CL18231.Contig1_All,
CL18287.Contig1_All, CL1846.Contig2_All,
CL18560.Contig1_All, CL18627.Contig1_All,
CL18639.Contig1_All, CL18713.Contig1_All,
CL18733.Contig1_All, CL18735.Contig1_All,
CL18738.Contig1_All, CL18772.Contig1_All,
CL18827.Contig1_All, CL18886.Contig1_All,
CL18911.Contig1_All, CL18917.Contig1_All,
CL18981.Contig1_All, CL19.Contig14_All, CL19.Contig16_All,
CL19.Contig1_All, CL19.Contig2_All, CL19090.Contig1_All,
CL19094.Contig1_All, CL19238.Contig1_All,
CL19337.Contig1_All, CL19428.Contig1_All,
CL19443.Contig1_All, CL1952.Contig1_All,
CL19534.Contig1_All, CL19671.Contig1_All,
CL19684.Contig1_All, CL19740.Contig1_All,
CL19749.Contig1_All, CL19756.Contig1_All,
CL19861.Contig1_All, CL2000.Contig1_All,
CL2011.Contig1_All, CL2011.Contig2_All,
CL2011.Contig3_All, CL20230.Contig1_All,

CL20260.Contig1_All, CL20299.Contig1_All,
CL20300.Contig1_All, CL20543.Contig1_All,
CL2058.Contig1_All, CL20596.Contig1_All,
CL20608.Contig1_All, CL20647.Contig1_All,
CL207.Contig2_All, CL2078.Contig1_All, CL2081.Contig1_All,
CL2081.Contig3_All, CL20814.Contig1_All,
CL2086.Contig1_All, CL20907.Contig1_All,
CL20971.Contig1_All, CL21.Contig2_All,
CL21023.Contig1_All, CL2103.Contig1_All,
CL2103.Contig2_All, CL2103.Contig3_All,
CL2103.Contig4_All, CL21047.Contig1_All,
CL21066.Contig1_All, CL21166.Contig1_All,
CL21317.Contig1_All, CL21326.Contig1_All,
CL2135.Contig1_All, CL21404.Contig1_All,
CL21465.Contig1_All, CL215.Contig1_All, CL215.Contig2_All,
CL215.Contig3_All, CL21522.Contig1_All,
CL21545.Contig1_All, CL21665.Contig1_All,
CL21676.Contig1_All, CL2168.Contig1_All,
CL21683.Contig1_All, CL2178.Contig1_All,
CL2178.Contig2_All, CL21890.Contig1_All,
CL219.Contig4_All, CL2192.Contig1_All, CL2192.Contig2_All,
CL2192.Contig3_All, CL22179.Contig1_All,
CL2219.Contig1_All, CL2220.Contig1_All,
CL22205.Contig1_All, CL22287.Contig1_All,
CL2230.Contig1_All, CL2230.Contig2_All,
CL2243.Contig1_All, CL22481.Contig1_All,
CL2254.Contig1_All, CL22565.Contig1_All,
CL2263.Contig1_All, CL22646.Contig1_All,
CL2267.Contig1_All, CL2269.Contig1_All,
CL22730.Contig1_All, CL2279.Contig1_All,
CL2279.Contig2_All, CL22866.Contig1_All,
CL229.Contig2_All, CL22985.Contig1_All, CL23.Contig2_All,
CL230.Contig1_All, CL23084.Contig1_All,
CL23090.Contig1_All, CL23140.Contig1_All,
CL23170.Contig1_All, CL23385.Contig1_All,
CL23424.Contig1_All, CL235.Contig1_All,
CL23511.Contig1_All, CL2353.Contig1_All,
CL23553.Contig1_All, CL23630.Contig1_All,
CL2364.Contig1_All, CL237.Contig6_All,
CL23755.Contig1_All, CL23856.Contig1_All,
CL23960.Contig1_All, CL24047.Contig1_All,
CL24058.Contig1_All, CL2410.Contig1_All,
CL24148.Contig1_All, CL24162.Contig1_All,
CL2417.Contig1_All, CL24388.Contig1_All,
CL24521.Contig1_All, CL2466.Contig1_All,
CL24765.Contig1_All, CL24775.Contig1_All,
CL24843.Contig1_All, CL24848.Contig1_All,
CL2488.Contig4_All, CL24937.Contig1_All,
CL2511.Contig1_All, CL2511.Contig2_All,
CL2511.Contig3_All, CL25111.Contig1_All,
CL25119.Contig1_All, CL2521.Contig1_All,
CL25220.Contig1_All, CL2526.Contig1_All,
CL25313.Contig1_All, CL25366.Contig1_All,
CL2538.Contig2_All, CL2538.Contig3_All,

CL2539.Contig1_All, CL25436.Contig1_All,
CL2575.Contig1_All, CL2575.Contig2_All,
CL25831.Contig1_All, CL26.Contig4_All, CL26.Contig6_All,
CL2601.Contig1_All, CL2602.Contig1_All,
CL26126.Contig1_All, CL26396.Contig1_All,
CL26423.Contig1_All, CL26430.Contig1_All,
CL26468.Contig1_All, CL2647.Contig1_All,
CL26704.Contig1_All, CL26710.Contig1_All,
CL2691.Contig1_All, CL26934.Contig1_All,
CL2695.Contig1_All, CL2695.Contig2_All,
CL27104.Contig1_All, CL27122.Contig1_All,
CL27178.Contig1_All, CL27179.Contig1_All,
CL27338.Contig1_All, CL27458.Contig1_All,
CL27491.Contig1_All, CL27639.Contig1_All,
CL2765.Contig1_All, CL2765.Contig2_All,
CL2766.Contig1_All, CL2767.Contig1_All,
CL27734.Contig1_All, CL27754.Contig1_All,
CL27773.Contig1_All, CL27810.Contig1_All,
CL27839.Contig1_All, CL27850.Contig1_All,
CL27913.Contig1_All, CL28038.Contig1_All,
CL28064.Contig1_All, CL28071.Contig1_All,
CL2808.Contig1_All, CL28114.Contig1_All,
CL2817.Contig1_All, CL2822.Contig1_All,
CL2822.Contig2_All, CL285.Contig2_All, CL2885.Contig1_All,
CL2947.Contig1_All, CL2983.Contig1_All,
CL2983.Contig2_All, CL3025.Contig1_All,
CL3038.Contig1_All, CL3038.Contig2_All, CL304.Contig2_All,
CL304.Contig3_All, CL304.Contig4_All, CL3084.Contig2_All,
CL3090.Contig1_All, CL3102.Contig1_All,
CL3119.Contig1_All, CL314.Contig2_All, CL3141.Contig1_All,
CL3176.Contig1_All, CL3177.Contig1_All,
CL3186.Contig1_All, CL3186.Contig2_All, CL321.Contig1_All,
CL3210.Contig1_All, CL3221.Contig1_All,
CL3258.Contig1_All, CL3258.Contig2_All,
CL3275.Contig1_All, CL3275.Contig2_All, CL33.Contig12_All,
CL33.Contig13_All, CL334.Contig4_All, CL335.Contig2_All,
CL3373.Contig1_All, CL3373.Contig2_All,
CL3373.Contig3_All, CL3373.Contig4_All, CL339.Contig2_All,
CL3403.Contig1_All, CL3415.Contig1_All,
CL3417.Contig1_All, CL342.Contig1_All, CL3468.Contig1_All,
CL3494.Contig1_All, CL35.Contig20_All, CL3537.Contig1_All,
CL356.Contig5_All, CL3655.Contig1_All, CL3667.Contig1_All,
CL3669.Contig1_All, CL3670.Contig1_All, CL37.Contig17_All,
CL37.Contig5_All, CL3706.Contig1_All, CL3786.Contig1_All,
CL3802.Contig1_All, CL3839.Contig1_All,
CL3886.Contig1_All, CL3946.Contig1_All,
CL3979.Contig2_All, CL4.Contig16_All, CL4.Contig27_All,
CL4.Contig2_All, CL4.Contig6_All, CL4035.Contig1_All,
CL405.Contig1_All, CL405.Contig4_All, CL405.Contig5_All,
CL405.Contig6_All, CL4166.Contig1_All, CL4228.Contig1_All,
CL4228.Contig2_All, CL4228.Contig3_All,
CL4232.Contig1_All, CL4252.Contig1_All,
CL4255.Contig1_All, CL4284.Contig1_All,
CL4296.Contig1_All, CL4328.Contig1_All,

			CL4333.Contig1_All, CL4362.Contig1_All, CL438.Contig2_All, CL4421.Contig1_All, CL4424.Contig1_All, CL4424.Contig2_All, CL4464.Contig1_All, CL4513.Contig1_All, CL4558.Contig1_All, CL4596.Contig2_All, CL4608.Contig1_All, CL4644.Contig1_All, CL4817.Contig1_All, CL4817.Contig2_All, CL4916.Contig1_All, CL4919.Contig1_All, CL492.Contig1_All, CL4926.Contig1_All, CL4950.Contig1_All, CL4950.Contig2_All, CL4993.Contig1_All, CL5.Contig29_All, CL5.Contig32_All, CL5027.Contig1_All, CL5063.Contig1_All, CL5077.Contig1_All, CL5078.Contig1_All, CL5085.Contig1_All, CL5149.Contig1_All, CL516.Contig3_All, CL5180.Contig1_All, CL5194.Contig1_All, CL5231.Contig1_All, CL5313.Contig1_All, CL5314.Contig2_All, CL5354.Contig1_All, CL5402.Contig1_All, CL5454.Contig1_All, CL5499.Contig1_All, CL55.Contig2_All, CL5530.Contig1_All, CL5530.Contig2_All, CL5565.Contig1_All, CL5565.Contig2_All, CL5574.Contig1_All, CL5578.Contig1_All, CL5593.Contig1_All, CL5632.Contig1_All, CL5632.Contig2_All, CL5688.Contig1_All, CL584.Contig1_All, CL5868.Contig1_All, CL589.Contig1_All, CL589.Contig2_All, CL589.Contig3_All, CL589.Contig4_All, CL5897.Contig1_All, CL5915.Contig1_All, CL5944.Contig1_All, CL5974.Contig1_All, CL5996.Contig1_All, CL6001.Contig1_All, CL6009.Contig1_All, CL6009.Contig2_All, CL603.Contig1_All, CL6102.Contig1_All, CL6123.Contig1_All, CL6137.Contig1_All, CL6156.Contig1_All, CL6163.Contig1_All, CL6163.Contig2_All, CL6166.Contig1_All, CL6202.Contig1_All, CL6292.Contig1_All, CL6344.Contig1_All, CL6344.Contig2_All, CL6359.Contig1_All, CL6394.Contig1_All, CL6476.Contig1_All, CL654.Contig3_All, CL654.Contig4_All, CL6586.Contig1_All, CL66.Contig2_All, CL66.Contig4_All, CL66.Contig6_All, CL6683.Contig1_All, CL6701.Contig1_All, CL6837.Contig1_All, CL6897.Contig1_All, CL6897.Contig2_All, CL6935.Contig1_All, CL6953.Contig1_All, CL6959.Contig1_All, CL6983.Contig1_All, CL7022.Contig1_All, CL7067.Contig1_All, CL71.Contig6_All, CL71.Contig8_All, CL7139.Contig1_All, CL716.Contig1_All, CL7161.Contig1_All, CL719.Contig4_All, CL719.Contig5_All, CL719.Contig6_All, CL719.Contig8_All, CL7209.Contig1_All, CL7237.Contig1_All, CL735.Contig3_All, CL7394.Contig1_All, CL7465.Contig1_All, CL7505.Contig1_All, CL7533.Contig1_All, CL7534.Contig1_All, CL762.Contig3_All, CL7636.Contig1_All, CL769.Contig1_All, CL769.Contig2_All, CL7705.Contig1_All, CL7721.Contig1_All, CL7727.Contig1_All, CL7744.Contig1_All, CL777.Contig1_All, CL777.Contig2_All, CL7871.Contig1_All, CL7972.Contig1_All, CL80.Contig3_All, CL80.Contig4_All, CL80.Contig7_All, CL8043.Contig1_All, CL813.Contig1_All, CL8160.Contig1_All,

		CL824.Contig1_All, CL824.Contig2_All, CL8317.Contig1_All, CL8317.Contig2_All, CL8325.Contig1_All, CL8325.Contig2_All, CL835.Contig1_All, CL8391.Contig1_All, CL8421.Contig1_All, CL8428.Contig1_All, CL8473.Contig1_All, CL8473.Contig2_All, CL8526.Contig1_All, CL8626.Contig1_All, CL864.Contig1_All, CL864.Contig3_All, CL870.Contig1_All, CL8740.Contig1_All, CL8743.Contig1_All, CL876.Contig2_All, CL878.Contig1_All, CL8785.Contig1_All, CL8785.Contig2_All, CL8800.Contig1_All, CL8854.Contig1_All, CL888.Contig1_All, CL888.Contig2_All, CL8906.Contig1_All, CL8941.Contig1_All, CL8993.Contig1_All, CL9007.Contig1_All, CL9047.Contig1_All, CL913.Contig2_All, CL913.Contig3_All, CL913.Contig4_All, CL9188.Contig1_All, CL9189.Contig1_All, CL9338.Contig1_All, CL9400.Contig1_All, CL9401.Contig1_All, CL9410.Contig1_All, CL953.Contig1_All, CL953.Contig2_All, CL956.Contig1_All, CL956.Contig2_All, CL9583.Contig1_All, CL9601.Contig1_All, CL9689.Contig1_All, CL9703.Contig1_All, CL9734.Contig1_All, CL9784.Contig1_All, CL9806.Contig1_All, CL9818.Contig1_All, CL9928.Contig1_All, CL9928.Contig2_All, CL9982.Contig1_All, Unigene1014_All, Unigene1053_All, Unigene1058_All, Unigene1153_All, Unigene1240_All, Unigene1271_All, Unigene1296_All, Unigene1299_All, Unigene1399_All, Unigene1425_All, Unigene1426_All, Unigene1476_All, Unigene1519_All, Unigene1727_All, Unigene1740_All, Unigene1742_All, Unigene1747_All, Unigene1794_All, Unigene187_All, Unigene1894_All, Unigene1930_All, Unigene1967_All, Unigene202_All, Unigene2128_All, Unigene2159_All, Unigene2282_All, Unigene2370_All, Unigene2556_All, Unigene2968_All, Unigene2986_All, Unigene2995_All, Unigene3073_All, Unigene3079_All, Unigene3097_All, Unigene3212_All, Unigene3294_All, Unigene3362_All, Unigene3425_All, Unigene3653_All, Unigene3778_All, Unigene3842_All, Unigene3893_All, Unigene3961_All, Unigene4025_All, Unigene4094_All, Unigene4097_All, Unigene4182_All, Unigene4309_All, Unigene436_All, Unigene4373_All, Unigene4374_All, Unigene4492_All, Unigene4503_All, Unigene4694_All, Unigene4739_All, Unigene4797_All, Unigene4798_All, Unigene4856_All, Unigene4870_All, Unigene4936_All, Unigene5067_All, Unigene5099_All, Unigene513_All, Unigene5157_All, Unigene5231_All, Unigene5340_All, Unigene5447_All, Unigene5481_All, Unigene647_All, Unigene668_All, Unigene688_All, Unigene707_All, Unigene718_All, Unigene829_All, Unigene875_All, Unigene937_All
7		CL1.Contig120_All, CL1.Contig123_All, CL1.Contig130_All, CL1.Contig23_All, CL1.Contig45_All, CL1.Contig46_All, CL1.Contig57_All, CL1.Contig59_All, CL1.Contig63_All, CL1.Contig91_All, CL1.Contig94_All, CL10138.Contig1_All, CL10153.Contig1_All, CL10172.Contig1_All, CL10258.Contig1_All, CL10260.Contig1_All,

	Endocytosis	CL10350.Contig1_All, CL10406.Contig1_All, CL10416.Contig1_All, CL10416.Contig2_All, CL10416.Contig3_All, CL10417.Contig1_All, CL10483.Contig1_All, CL10535.Contig1_All, CL10621.Contig1_All, CL10624.Contig1_All, CL10625.Contig1_All, CL10648.Contig1_All, CL10678.Contig1_All, CL10686.Contig1_All, CL1069.Contig1_All, CL10716.Contig1_All, CL1074.Contig1_All, CL1074.Contig2_All, CL10984.Contig1_All, CL10993.Contig1_All, CL11020.Contig1_All, CL11079.Contig1_All, CL11084.Contig1_All, CL11094.Contig1_All, CL11145.Contig1_All, CL11153.Contig1_All, CL11160.Contig1_All, CL11210.Contig1_All, CL11236.Contig1_All, CL11261.Contig1_All, CL11309.Contig1_All, CL11477.Contig1_All, CL11520.Contig1_All, CL11616.Contig1_All, CL11672.Contig1_All, CL1173.Contig1_All, CL11747.Contig1_All, CL1175.Contig1_All, CL11776.Contig1_All, CL11837.Contig1_All, CL11980.Contig1_All, CL11989.Contig1_All, CL1207.Contig1_All, CL1207.Contig2_All, CL1207.Contig3_All, CL1207.Contig4_All, CL12095.Contig1_All, CL12509.Contig1_All, CL12522.Contig1_All, CL12746.Contig1_All, CL12752.Contig1_All, CL12886.Contig1_All, CL13064.Contig1_All, CL1312.Contig1_All, CL13216.Contig1_All, CL1328.Contig1_All, CL13297.Contig2_All, CL13332.Contig1_All, CL13357.Contig1_All, CL13666.Contig1_All, CL13717.Contig1_All, CL13717.Contig2_All, CL13720.Contig1_All, CL13747.Contig1_All, CL1378.Contig1_All, CL1378.Contig2_All, CL13818.Contig1_All, CL13864.Contig1_All, CL13877.Contig1_All, CL13975.Contig1_All, CL13975.Contig2_All, CL14003.Contig1_All, CL1406.Contig2_All, CL1406.Contig3_All, CL14063.Contig1_All, CL14063.Contig2_All, CL14112.Contig1_All, CL14178.Contig1_All, CL14270.Contig1_All, CL14310.Contig1_All, CL14313.Contig1_All, CL14326.Contig1_All, CL14365.Contig1_All, CL14392.Contig1_All, CL14408.Contig1_All, CL14416.Contig1_All, CL14487.Contig1_All, CL14558.Contig1_All, CL14690.Contig1_All, CL14738.Contig1_All, CL14752.Contig1_All, CL14764.Contig1_All, CL14855.Contig1_All, CL14871.Contig1_All, CL14877.Contig1_All, CL14966.Contig1_All, CL14979.Contig1_All, CL151.Contig2_All, CL151.Contig4_All, CL15160.Contig1_All, CL15163.Contig1_All, CL15207.Contig1_All, CL15251.Contig1_All, CL15301.Contig1_All, CL15321.Contig1_All, CL15341.Contig1_All, CL15553.Contig1_All, CL15591.Contig1_All, CL15594.Contig1_All,

			CL15643.Contig1_All, CL157.Contig1_All, CL157.Contig2_All, CL157.Contig4_All, CL157.Contig5_All, CL157.Contig6_All, CL157.Contig7_All, CL157.Contig8_All, CL157.Contig9_All, CL15700.Contig1_All, CL15700.Contig2_All, CL1580.Contig1_All, CL15822.Contig1_All, CL15849.Contig1_All, CL1590.Contig1_All, CL1590.Contig2_All, CL1590.Contig3_All, CL15945.Contig1_All, CL16059.Contig1_All, CL16082.Contig1_All, CL16345.Contig1_All, CL16363.Contig1_All, CL1640.Contig1_All, CL1640.Contig2_All, CL1640.Contig3_All, CL1640.Contig4_All, CL16697.Contig1_All, CL16706.Contig1_All, CL1675.Contig1_All, CL17025.Contig1_All, CL171.Contig6_All, CL1711.Contig2_All, CL17220.Contig1_All, CL17263.Contig1_All, CL17278.Contig1_All, CL17278.Contig2_All, CL17326.Contig1_All, CL17382.Contig1_All, CL17426.Contig1_All, CL17454.Contig1_All, CL18194.Contig1_All, CL18287.Contig1_All, CL1830.Contig1_All, CL18369.Contig1_All, CL18429.Contig1_All, CL1858.Contig1_All, CL1871.Contig1_All, CL18751.Contig1_All, CL18816.Contig1_All, CL18827.Contig1_All, CL1906.Contig1_All, CL1906.Contig2_All, CL1906.Contig3_All, CL1906.Contig4_All, CL19075.Contig1_All, CL19090.Contig1_All, CL19337.Contig1_All, CL19394.Contig1_All, CL19434.Contig1_All, CL19625.Contig1_All, CL19670.Contig1_All, CL19696.Contig1_All, CL19701.Contig1_All, CL19756.Contig1_All, CL19768.Contig1_All, CL19848.Contig1_All, CL1999.Contig1_All, CL1999.Contig2_All, CL20085.Contig1_All, CL20376.Contig1_All, CL20396.Contig1_All, CL20397.Contig1_All, CL20578.Contig1_All, CL20608.Contig1_All, CL20655.Contig1_All, CL20755.Contig1_All, CL20826.Contig1_All, CL209.Contig4_All, CL2091.Contig1_All, CL2100.Contig1_All, CL2100.Contig2_All, CL21089.Contig1_All, CL21093.Contig1_All, CL21148.Contig1_All, CL21166.Contig1_All, CL21206.Contig1_All, CL21227.Contig1_All, CL21321.Contig1_All, CL21335.Contig1_All, CL21393.Contig1_All, CL21451.Contig1_All, CL21562.Contig1_All, CL2160.Contig1_All, CL21731.Contig1_All, CL2181.Contig1_All, CL2181.Contig2_All, CL2181.Contig3_All, CL2192.Contig1_All, CL2192.Contig3_All, CL21955.Contig1_All, CL21971.Contig1_All, CL22085.Contig1_All, CL22136.Contig1_All, CL22152.Contig1_All, CL22314.Contig1_All, CL22362.Contig1_All, CL22377.Contig1_All, CL22448.Contig1_All, CL22461.Contig1_All, CL22977.Contig1_All, CL23.Contig2_All, CL23120.Contig1_All, CL2320.Contig1_All,

			CL2320.Contig2_All, CL23238.Contig1_All, CL23327.Contig1_All, CL23358.Contig1_All, CL23407.Contig1_All, CL2353.Contig1_All, CL2353.Contig4_All, CL2355.Contig1_All, CL23588.Contig1_All, CL2387.Contig1_All, CL23965.Contig1_All, CL24172.Contig1_All, CL24251.Contig1_All, CL24281.Contig1_All, CL24479.Contig1_All, CL2465.Contig1_All, CL24775.Contig1_All, CL24831.Contig1_All, CL24843.Contig1_All, CL24937.Contig1_All, CL25143.Contig1_All, CL2515.Contig1_All, CL25220.Contig1_All, CL25363.Contig1_All, CL25376.Contig1_All, CL25710.Contig1_All, CL2584.Contig1_All, CL2599.Contig1_All, CL26221.Contig1_All, CL26309.Contig1_All, CL26423.Contig1_All, CL26483.Contig1_All, CL26501.Contig1_All, CL26645.Contig1_All, CL26707.Contig1_All, CL26856.Contig1_All, CL26978.Contig1_All, CL27010.Contig1_All, CL27024.Contig1_All, CL27290.Contig1_All, CL27338.Contig1_All, CL27661.Contig1_All, CL27703.Contig1_All, CL27751.Contig1_All, CL28038.Contig1_All, CL2860.Contig3_All, CL288.Contig3_All, CL288.Contig8_All, CL2961.Contig4_All, CL3186.Contig1_All, CL3187.Contig1_All, CL3199.Contig1_All, CL3199.Contig2_All, CL3203.Contig1_All, CL3203.Contig2_All, CL3285.Contig1_All, CL3285.Contig2_All, CL3308.Contig2_All, CL331.Contig2_All, CL331.Contig4_All, CL35.Contig11_All, CL35.Contig16_All, CL35.Contig1_All, CL35.Contig22_All, CL35.Contig4_All, CL35.Contig6_All, CL35.Contig8_All, CL351.Contig5_All, CL3572.Contig1_All, CL3572.Contig2_All, CL3646.Contig1_All, CL3646.Contig2_All, CL3723.Contig1_All, CL3723.Contig2_All, CL374.Contig5_All, CL3840.Contig1_All, CL385.Contig1_All, CL385.Contig2_All, CL385.Contig3_All, CL388.Contig1_All, CL388.Contig2_All, CL388.Contig3_All, CL388.Contig4_All, CL388.Contig5_All, CL3883.Contig1_All, CL3883.Contig2_All, CL3932.Contig1_All, CL3938.Contig1_All, CL3938.Contig2_All, CL3938.Contig3_All, CL3957.Contig1_All, CL3957.Contig2_All, CL3959.Contig1_All, CL3985.Contig2_All, CL4008.Contig1_All, CL4008.Contig2_All, CL4008.Contig3_All, CL4062.Contig1_All, CL4062.Contig2_All, CL4077.Contig1_All, CL4125.Contig1_All, CL4128.Contig1_All, CL4190.Contig1_All, CL4190.Contig2_All, CL4247.Contig1_All, CL425.Contig1_All, CL425.Contig2_All, CL425.Contig3_All, CL425.Contig4_All, CL425.Contig5_All, CL4295.Contig1_All, CL4295.Contig2_All, CL4328.Contig1_All, CL4362.Contig1_All, CL4363.Contig1_All, CL4363.Contig3_All, CL4466.Contig1_All, CL456.Contig1_All, CL456.Contig2_All, CL456.Contig3_All, CL456.Contig4_All, CL456.Contig5_All,

		CL456.Contig6_All, CL4573.Contig1_All, CL4573.Contig2_All, CL4685.Contig1_All, CL4723.Contig2_All, CL4827.Contig1_All, CL4892.Contig1_All, CL4892.Contig2_All, CL4926.Contig1_All, CL4946.Contig2_All, CL4946.Contig3_All, CL5036.Contig1_All, CL5068.Contig1_All, CL5126.Contig1_All, CL518.Contig1_All, CL518.Contig2_All, CL518.Contig3_All, CL518.Contig4_All, CL518.Contig5_All, CL5236.Contig1_All, CL5269.Contig1_All, CL5269.Contig2_All, CL5317.Contig1_All, CL5321.Contig1_All, CL535.Contig1_All, CL5359.Contig1_All, CL537.Contig2_All, CL537.Contig5_All, CL5401.Contig1_All, CL5475.Contig1_All, CL55.Contig2_All, CL55.Contig4_All, CL5561.Contig1_All, CL557.Contig1_All, CL557.Contig2_All, CL557.Contig3_All, CL557.Contig4_All, CL5608.Contig1_All, CL5703.Contig1_All, CL5798.Contig1_All, CL5814.Contig1_All, CL5867.Contig1_All, CL5870.Contig1_All, CL589.Contig1_All, CL589.Contig2_All, CL589.Contig3_All, CL5964.Contig1_All, CL6.Contig1_All, CL6.Contig25_All, CL6002.Contig1_All, CL6002.Contig2_All, CL6009.Contig1_All, CL6009.Contig2_All, CL6023.Contig1_All, CL6023.Contig2_All, CL6023.Contig3_All, CL606.Contig1_All, CL6096.Contig1_All, CL6155.Contig1_All, CL6156.Contig1_All, CL6204.Contig1_All, CL6248.Contig1_All, CL625.Contig1_All, CL6263.Contig1_All, CL6263.Contig2_All, CL6340.Contig1_All, CL6340.Contig2_All, CL6388.Contig1_All, CL6470.Contig1_All, CL6506.Contig1_All, CL6522.Contig1_All, CL6538.Contig1_All, CL658.Contig1_All, CL6581.Contig1_All, CL6617.Contig1_All, CL6617.Contig2_All, CL6649.Contig1_All, CL6649.Contig2_All, CL6696.Contig1_All, CL6913.Contig1_All, CL7.Contig25_All, CL7.Contig28_All, CL70.Contig8_All, CL7030.Contig1_All, CL7034.Contig1_All, CL7034.Contig2_All, CL7181.Contig1_All, CL7240.Contig1_All, CL7240.Contig2_All, CL7276.Contig1_All, CL7276.Contig2_All, CL7468.Contig1_All, CL751.Contig1_All, CL751.Contig2_All, CL751.Contig3_All, CL7597.Contig2_All, CL7615.Contig2_All, CL762.Contig1_All, CL762.Contig2_All, CL762.Contig3_All, CL7715.Contig1_All, CL7941.Contig1_All, CL7959.Contig1_All, CL7961.Contig1_All, CL8159.Contig1_All, CL817.Contig1_All, CL817.Contig2_All, CL820.Contig1_All, CL820.Contig2_All, CL8225.Contig1_All, CL8246.Contig1_All, CL8299.Contig1_All, CL8371.Contig1_All, CL8439.Contig1_All, CL8562.Contig1_All, CL86.Contig1_All, CL86.Contig2_All, CL86.Contig3_All, CL86.Contig4_All, CL86.Contig5_All, CL86.Contig6_All, CL86.Contig7_All, CL8608.Contig1_All, CL8608.Contig2_All, CL8618.Contig1_All, CL8618.Contig2_All, CL864.Contig1_All, CL864.Contig3_All, CL8643.Contig1_All, CL8643.Contig2_All, CL8669.Contig1_All, CL878.Contig1_All, CL8918.Contig1_All, CL913.Contig2_All, CL913.Contig3_All, CL913.Contig4_All,

		CL9190.Contig1_All, CL9268.Contig1_All, CL9384.Contig1_All, CL9408.Contig1_All, CL9410.Contig1_All, CL95.Contig12_All, CL95.Contig16_All, CL95.Contig23_All, CL95.Contig25_All, CL95.Contig7_All, CL95.Contig8_All, CL9508.Contig1_All, CL9568.Contig1_All, CL9580.Contig1_All, CL9724.Contig1_All, CL9928.Contig1_All, CL9928.Contig2_All, CL9941.Contig1_All, Unigene1043_All, Unigene1053_All, Unigene1104_All, Unigene1153_All, Unigene1207_All, Unigene1240_All, Unigene1262_All, Unigene1271_All, Unigene1274_All, Unigene1294_All, Unigene1329_All, Unigene1355_All, Unigene1683_All, Unigene1747_All, Unigene1782_All, Unigene1783_All, Unigene1819_All, Unigene185_All, Unigene1907_All, Unigene1931_All, Unigene1990_All, Unigene2150_All, Unigene2282_All, Unigene2292_All, Unigene2340_All, Unigene24_All, Unigene255_All, Unigene2560_All, Unigene2613_All, Unigene2681_All, Unigene2709_All, Unigene2741_All, Unigene3023_All, Unigene3042_All, Unigene3077_All, Unigene3079_All, Unigene3093_All, Unigene3101_All, Unigene3300_All, Unigene3553_All, Unigene3774_All, Unigene3778_All, Unigene4095_All, Unigene4316_All, Unigene4371_All, Unigene4372_All, Unigene4406_All, Unigene4464_All, Unigene4492_All, Unigene4503_All, Unigene4619_All, Unigene4628_All, Unigene4669_All, Unigene4679_All, Unigene4680_All, Unigene4685_All, Unigene4687_All, Unigene4688_All, Unigene4741_All, Unigene4805_All, Unigene4810_All, Unigene5339_All, Unigene5407_All, Unigene5413_All, Unigene560_All, Unigene607_All, Unigene939_All
8		CL1.Contig114_All, CL1.Contig127_All, CL1.Contig132_All, CL1.Contig138_All, CL1.Contig58_All, CL1.Contig78_All, CL1.Contig86_All, CL1.Contig92_All, CL10070.Contig1_All, CL10090.Contig1_All, CL10091.Contig1_All, CL10101.Contig1_All, CL10111.Contig1_All, CL10119.Contig1_All, CL10134.Contig1_All, CL10177.Contig1_All, CL1019.Contig1_All, CL1019.Contig2_All, CL1019.Contig3_All, CL1019.Contig4_All, CL10196.Contig1_All, CL1023.Contig1_All, CL1023.Contig2_All, CL10259.Contig1_All, CL10276.Contig1_All, CL10390.Contig1_All, CL10403.Contig1_All, CL10422.Contig1_All, CL10434.Contig1_All, CL10451.Contig1_All, CL10452.Contig1_All, CL10530.Contig1_All, CL10598.Contig1_All, CL10648.Contig1_All, CL10715.Contig1_All, CL10802.Contig1_All, CL1088.Contig1_All, CL10907.Contig1_All, CL1091.Contig1_All, CL1093.Contig1_All, CL10984.Contig1_All, CL11012.Contig1_All, CL11079.Contig1_All, CL11113.Contig1_All, CL11179.Contig1_All, CL11183.Contig1_All, CL11185.Contig1_All, CL11297.Contig1_All, CL11307.Contig1_All, CL11318.Contig1_All, CL11340.Contig1_All,

	Protein processing in	CL11389.Contig1_All, CL11459.Contig1_All, CL11486.Contig1_All, CL11487.Contig1_All, CL11545.Contig1_All, CL11548.Contig1_All, CL11594.Contig1_All, CL11601.Contig1_All, CL11638.Contig1_All, CL11640.Contig1_All, CL11815.Contig1_All, CL11970.Contig1_All, CL12065.Contig1_All, CL12082.Contig1_All, CL12090.Contig1_All, CL12147.Contig1_All, CL12163.Contig1_All, CL12312.Contig1_All, CL12328.Contig1_All, CL12419.Contig1_All, CL1251.Contig1_All, CL1251.Contig2_All, CL1251.Contig3_All, CL12713.Contig1_All, CL12827.Contig1_All, CL12901.Contig1_All, CL13061.Contig1_All, CL13064.Contig1_All, CL13114.Contig1_All, CL13173.Contig1_All, CL1321.Contig1_All, CL1321.Contig2_All, CL1321.Contig3_All, CL1321.Contig4_All, CL13216.Contig1_All, CL13306.Contig1_All, CL13389.Contig1_All, CL13406.Contig1_All, CL1351.Contig1_All, CL13514.Contig1_All, CL13606.Contig1_All, CL13747.Contig1_All, CL13756.Contig1_All, CL13841.Contig1_All, CL13911.Contig1_All, CL13959.Contig1_All, CL14009.Contig1_All, CL1402.Contig1_All, CL14035.Contig1_All, CL14071.Contig1_All, CL1411.Contig1_All, CL14161.Contig1_All, CL14178.Contig1_All, CL14256.Contig1_All, CL14284.Contig1_All, CL14415.Contig1_All, CL14524.Contig1_All, CL1463.Contig1_All, CL1463.Contig2_All, CL1463.Contig3_All, CL1463.Contig4_All, CL1463.Contig5_All, CL14840.Contig1_All, CL1485.Contig1_All, CL1485.Contig2_All, CL1485.Contig3_All, CL1490.Contig1_All, CL14927.Contig1_All, CL15033.Contig1_All, CL15034.Contig1_All, CL15037.Contig1_All, CL15053.Contig1_All, CL1518.Contig1_All, CL1518.Contig2_All, CL1518.Contig3_All, CL1518.Contig4_All, CL15197.Contig1_All, CL15235.Contig1_All, CL15277.Contig1_All, CL15365.Contig1_All, CL15482.Contig1_All, CL15496.Contig1_All, CL15549.Contig1_All, CL15553.Contig1_All, CL15569.Contig1_All, CL15692.Contig1_All, CL15743.Contig1_All, CL15797.Contig1_All, CL15820.Contig1_All, CL15838.Contig1_All, CL1607.Contig1_All, CL16093.Contig1_All, CL16099.Contig1_All, CL16099.Contig2_All, CL16112.Contig1_All, CL16117.Contig1_All, CL1612.Contig1_All, CL16223.Contig1_All, CL16223.Contig2_All, CL16343.Contig1_All, CL1637.Contig1_All, CL165.Contig3_All, CL1653.Contig1_All, CL1653.Contig2_All, CL1653.Contig3_All, CL1653.Contig4_All, CL1660.Contig1_All, CL1660.Contig2_All, CL16619.Contig1_All,

	endoplasmic reticulum	CL16697.Contig1_All, CL16784.Contig1_All, CL16796.Contig1_All, CL16939.Contig1_All, CL16965.Contig1_All, CL17.Contig15_All, CL17.Contig16_All, CL17.Contig30_All, CL17.Contig40_All, CL17.Contig41_All, CL17.Contig45_All, CL17223.Contig1_All, CL1726.Contig1_All, CL17443.Contig1_All, CL17454.Contig1_All, CL17704.Contig1_All, CL17767.Contig1_All, CL17884.Contig1_All, CL1811.Contig1_All, CL1811.Contig2_All, CL18281.Contig1_All, CL18287.Contig1_All, CL18410.Contig1_All, CL1842.Contig1_All, CL1851.Contig2_All, CL1851.Contig3_All, CL18827.Contig1_All, CL18903.Contig1_All, CL19090.Contig1_All, CL19129.Contig1_All, CL19219.Contig1_All, CL19222.Contig1_All, CL1939.Contig1_All, CL19400.Contig1_All, CL19416.Contig1_All, CL1960.Contig1_All, CL19648.Contig1_All, CL19656.Contig1_All, CL19675.Contig1_All, CL19825.Contig1_All, CL20004.Contig1_All, CL2029.Contig1_All, CL2037.Contig1_All, CL20488.Contig1_All, CL20587.Contig1_All, CL20589.Contig1_All, CL20625.Contig1_All, CL20863.Contig1_All, CL20905.Contig1_All, CL20910.Contig1_All, CL2095.Contig1_All, CL20960.Contig1_All, CL20995.Contig1_All, CL21126.Contig1_All, CL21166.Contig1_All, CL21179.Contig1_All, CL21189.Contig1_All, CL21284.Contig1_All, CL21298.Contig1_All, CL21368.Contig1_All, CL21423.Contig1_All, CL21530.Contig1_All, CL21894.Contig1_All, CL2192.Contig3_All, CL2203.Contig1_All, CL22648.Contig1_All, CL2271.Contig1_All, CL2271.Contig2_All, CL2271.Contig3_All, CL2282.Contig1_All, CL23.Contig2_All, CL23084.Contig1_All, CL23229.Contig1_All, CL23348.Contig1_All, CL23384.Contig1_All, CL23558.Contig1_All, CL23663.Contig1_All, CL23761.Contig1_All, CL2401.Contig1_All, CL2403.Contig1_All, CL24052.Contig1_All, CL24532.Contig1_All, CL24775.Contig1_All, CL24843.Contig1_All, CL24937.Contig1_All, CL2518.Contig1_All, CL2548.Contig2_All, CL2548.Contig3_All, CL25506.Contig1_All, CL25657.Contig1_All, CL25722.Contig1_All, CL2573.Contig1_All, CL25782.Contig1_All, CL259.Contig2_All, CL259.Contig3_All, CL259.Contig5_All, CL259.Contig6_All, CL2608.Contig1_All, CL2608.Contig2_All, CL2608.Contig3_All, CL2613.Contig1_All, CL26192.Contig1_All, CL26308.Contig1_All, CL26423.Contig1_All, CL26501.Contig1_All, CL26675.Contig1_All, CL26678.Contig1_All, CL27.Contig5_All, CL27.Contig7_All, CL2709.Contig1_All, CL27105.Contig1_All, CL27153.Contig1_All, CL27238.Contig1_All, CL27265.Contig1_All,

			CL27338.Contig1_All, CL2737.Contig1_All, CL274.Contig2_All, CL2763.Contig1_All, CL27777.Contig1_All, CL27911.Contig1_All, CL2795.Contig1_All, CL2795.Contig2_All, CL2795.Contig3_All, CL27953.Contig1_All, CL27960.Contig1_All, CL2797.Contig1_All, CL2797.Contig2_All, CL2797.Contig3_All, CL28038.Contig1_All, CL28040.Contig1_All, CL2818.Contig1_All, CL2818.Contig2_All, CL2818.Contig3_All, CL2836.Contig1_All, CL2848.Contig1_All, CL2848.Contig2_All, CL2900.Contig2_All, CL2900.Contig5_All, CL292.Contig1_All, CL292.Contig2_All, CL292.Contig3_All, CL292.Contig4_All, CL292.Contig5_All, CL2926.Contig1_All, CL2926.Contig2_All, CL2930.Contig1_All, CL3064.Contig1_All, CL3067.Contig1_All, CL313.Contig1_All, CL313.Contig2_All, CL313.Contig3_All, CL3137.Contig1_All, CL3141.Contig1_All, CL3201.Contig1_All, CL3265.Contig1_All, CL3298.Contig1_All, CL3301.Contig1_All, CL3313.Contig1_All, CL3336.Contig1_All, CL3358.Contig1_All, CL3364.Contig1_All, CL3364.Contig2_All, CL3375.Contig1_All, CL3474.Contig1_All, CL3474.Contig3_All, CL3499.Contig1_All, CL3506.Contig1_All, CL3507.Contig1_All, CL3507.Contig2_All, CL356.Contig2_All, CL356.Contig3_All, CL356.Contig4_All, CL3624.Contig1_All, CL3627.Contig1_All, CL3627.Contig2_All, CL3652.Contig1_All, CL3700.Contig1_All, CL3704.Contig1_All, CL3725.Contig1_All, CL3752.Contig1_All, CL3798.Contig1_All, CL3844.Contig1_All, CL3844.Contig2_All, CL3883.Contig2_All, CL3924.Contig1_All, CL3951.Contig1_All, CL3951.Contig2_All, CL4068.Contig1_All, CL407.Contig1_All, CL407.Contig2_All, CL407.Contig3_All, CL4076.Contig1_All, CL417.Contig1_All, CL417.Contig3_All, CL417.Contig4_All, CL417.Contig6_All, CL417.Contig7_All, CL4186.Contig1_All, CL4186.Contig2_All, CL4193.Contig1_All, CL4206.Contig1_All, CL4206.Contig2_All, CL4208.Contig1_All, CL4282.Contig1_All, CL4321.Contig1_All, CL4321.Contig2_All, CL4321.Contig3_All, CL4328.Contig1_All, CL4396.Contig1_All, CL4410.Contig1_All, CL4450.Contig1_All, CL4472.Contig1_All, CL4476.Contig1_All, CL4553.Contig1_All, CL4612.Contig1_All, CL4614.Contig1_All, CL462.Contig1_All, CL462.Contig2_All, CL462.Contig3_All, CL462.Contig4_All, CL462.Contig5_All, CL462.Contig6_All, CL462.Contig7_All, CL463.Contig4_All, CL4639.Contig2_All, CL467.Contig1_All, CL4700.Contig1_All, CL4707.Contig2_All, CL4740.Contig1_All, CL4887.Contig1_All, CL4915.Contig1_All, CL4926.Contig1_All, CL493.Contig1_All, CL493.Contig6_All, CL4986.Contig1_All, CL4986.Contig2_All, CL5043.Contig1_All, CL5043.Contig2_All,

		CL5056.Contig1_All, CL5060.Contig1_All, CL5079.Contig1_All, CL5164.Contig1_All, CL5164.Contig2_All, CL5252.Contig1_All, CL5273.Contig1_All, CL5286.Contig1_All, CL5371.Contig1_All, CL5375.Contig1_All, CL5446.Contig1_All, CL5473.Contig1_All, CL5532.Contig1_All, CL5590.Contig1_All, CL5658.Contig1_All, CL5672.Contig1_All, CL5774.Contig1_All, CL5783.Contig1_All, CL5861.Contig1_All, CL5931.Contig1_All, CL5957.Contig1_All, CL6031.Contig1_All, CL6032.Contig1_All, CL606.Contig2_All, CL6072.Contig1_All, CL6072.Contig2_All, CL6147.Contig1_All, CL6156.Contig1_All, CL616.Contig2_All, CL616.Contig3_All, CL616.Contig4_All, CL6215.Contig1_All, CL6400.Contig1_All, CL644.Contig1_All, CL644.Contig2_All, CL644.Contig3_All, CL644.Contig4_All, CL644.Contig5_All, CL644.Contig6_All, CL644.Contig7_All, CL644.Contig8_All, CL6531.Contig1_All, CL6532.Contig1_All, CL658.Contig1_All, CL658.Contig2_All, CL6631.Contig1_All, CL6658.Contig1_All, CL6750.Contig1_All, CL6784.Contig1_All, CL6852.Contig1_All, CL7104.Contig1_All, CL711.Contig1_All, CL711.Contig2_All, CL7180.Contig1_All, CL7234.Contig1_All, CL7256.Contig1_All, CL7293.Contig1_All, CL7293.Contig3_All, CL7313.Contig1_All, CL7313.Contig2_All, CL7375.Contig1_All, CL7439.Contig1_All, CL745.Contig1_All, CL745.Contig2_All, CL745.Contig3_All, CL745.Contig4_All, CL7471.Contig1_All, CL7504.Contig1_All, CL7511.Contig1_All, CL762.Contig2_All, CL7629.Contig1_All, CL7766.Contig1_All, CL7773.Contig1_All, CL78.Contig12_All, CL7874.Contig1_All, CL7883.Contig1_All, CL7936.Contig1_All, CL7973.Contig1_All, CL8035.Contig1_All, CL8191.Contig1_All, CL8191.Contig3_All, CL8225.Contig1_All, CL8232.Contig1_All, CL8266.Contig1_All, CL8351.Contig1_All, CL8355.Contig1_All, CL8469.Contig1_All, CL8721.Contig1_All, CL8793.Contig1_All, CL8833.Contig1_All, CL8888.Contig1_All, CL900.Contig2_All, CL9088.Contig1_All, CL913.Contig2_All, CL913.Contig3_All, CL913.Contig4_All, CL9181.Contig1_All, CL9298.Contig1_All, CL9406.Contig1_All, CL9410.Contig1_All, CL9424.Contig1_All, CL9424.Contig2_All, CL9475.Contig1_All, CL9515.Contig1_All, CL954.Contig1_All, CL954.Contig2_All, CL963.Contig1_All, CL9679.Contig1_All, CL9709.Contig1_All, CL9722.Contig1_All, CL9731.Contig1_All, CL9816.Contig1_All, CL9828.Contig1_All, CL9937.Contig1_All, CL9939.Contig1_All, CL9951.Contig1_All, CL9951.Contig2_All, Unigene1240_All, Unigene1259_All, Unigene1271_All, Unigene1299_All, Unigene1344_All, Unigene1346_All, Unigene1351_All, Unigene1355_All, Unigene1366_All, Unigene1369_All, Unigene1515_All, Unigene1717_All, Unigene1718_All, Unigene1747_All,

		Unigene2659_All, Unigene2866_All, Unigene3204_All, Unigene3599_All, Unigene3676_All, Unigene3778_All, Unigene3915_All, Unigene4079_All, Unigene4254_All, Unigene4255_All, Unigene4327_All, Unigene4401_All, Unigene4492_All, Unigene4597_All, Unigene4610_All, Unigene4632_All, Unigene4636_All, Unigene4949_All, Unigene5069_All, Unigene5201_All, Unigene5337_All, Unigene5338_All, Unigene5360_All, Unigene5479_All, Unigene5490_All, Unigene5492_All, Unigene5504_All, Unigene811_All, Unigene845_All, Unigene847_All, Unigene949_All
9		CL10050.Contig1_All, CL10097.Contig1_All, CL10110.Contig1_All, CL10120.Contig1_All, CL10143.Contig1_All, CL10222.Contig1_All, CL10231.Contig1_All, CL10297.Contig1_All, CL10348.Contig1_All, CL10552.Contig1_All, CL10761.Contig1_All, CL10787.Contig1_All, CL10801.Contig1_All, CL10990.Contig1_All, CL11001.Contig1_All, CL11014.Contig1_All, CL11056.Contig1_All, CL11058.Contig1_All, CL11141.Contig1_All, CL11151.Contig1_All, CL11194.Contig1_All, CL11194.Contig2_All, CL11199.Contig1_All, CL11238.Contig1_All, CL11240.Contig1_All, CL11253.Contig1_All, CL11281.Contig1_All, CL11283.Contig1_All, CL11322.Contig1_All, CL11335.Contig1_All, CL11412.Contig1_All, CL11419.Contig1_All, CL11439.Contig1_All, CL11446.Contig1_All, CL11497.Contig1_All, CL11515.Contig1_All, CL11543.Contig1_All, CL11554.Contig1_All, CL11571.Contig1_All, CL11603.Contig1_All, CL11678.Contig1_All, CL11799.Contig1_All, CL11834.Contig1_All, CL11910.Contig1_All, CL11920.Contig1_All, CL12057.Contig1_All, CL12062.Contig1_All, CL12100.Contig1_All, CL12139.Contig1_All, CL12164.Contig1_All, CL12171.Contig1_All, CL12192.Contig1_All, CL12215.Contig1_All, CL12287.Contig1_All, CL12363.Contig1_All, CL12365.Contig1_All, CL12390.Contig1_All, CL12391.Contig1_All, CL12433.Contig1_All, CL12449.Contig1_All, CL12449.Contig2_All, CL12510.Contig1_All, CL12543.Contig1_All, CL12549.Contig1_All, CL12554.Contig1_All, CL12618.Contig1_All, CL12684.Contig1_All, CL12701.Contig1_All, CL12710.Contig1_All, CL12718.Contig1_All, CL12747.Contig1_All, CL12836.Contig1_All, CL12867.Contig1_All, CL12874.Contig1_All, CL12874.Contig2_All, CL12999.Contig1_All, CL13035.Contig1_All, CL13043.Contig1_All, CL13067.Contig1_All, CL13090.Contig1_All, CL13132.Contig1_All, CL13173.Contig1_All, CL13221.Contig1_All, CL13267.Contig1_All, CL13318.Contig1_All, CL13375.Contig1_All,

	Ribosome	CL13438.Contig1_All, CL13470.Contig1_All, CL13494.Contig1_All, CL13507.Contig1_All, CL13529.Contig1_All, CL13568.Contig1_All, CL13603.Contig1_All, CL13614.Contig1_All, CL13619.Contig1_All, CL13638.Contig1_All, CL13646.Contig1_All, CL13676.Contig1_All, CL13706.Contig1_All, CL13709.Contig1_All, CL13711.Contig1_All, CL13726.Contig1_All, CL13749.Contig1_All, CL1375.Contig1_All, CL1375.Contig2_All, CL1375.Contig3_All, CL1375.Contig4_All, CL13758.Contig1_All, CL13784.Contig1_All, CL13815.Contig1_All, CL13830.Contig1_All, CL13837.Contig1_All, CL13852.Contig1_All, CL13860.Contig1_All, CL13861.Contig1_All, CL13863.Contig1_All, CL13879.Contig1_All, CL13882.Contig1_All, CL13920.Contig1_All, CL13921.Contig1_All, CL13930.Contig1_All, CL13951.Contig1_All, CL13953.Contig1_All, CL13953.Contig2_All, CL13960.Contig1_All, CL13987.Contig1_All, CL13988.Contig1_All, CL13991.Contig1_All, CL14015.Contig1_All, CL14022.Contig1_All, CL14026.Contig1_All, CL14073.Contig1_All, CL14079.Contig1_All, CL14081.Contig1_All, CL14110.Contig1_All, CL14118.Contig1_All, CL14131.Contig1_All, CL14137.Contig1_All, CL14147.Contig1_All, CL14159.Contig1_All, CL14162.Contig1_All, CL14184.Contig1_All, CL14197.Contig1_All, CL14216.Contig1_All, CL14227.Contig1_All, CL14257.Contig1_All, CL14290.Contig1_All, CL14291.Contig1_All, CL14377.Contig1_All, CL14403.Contig1_All, CL14417.Contig1_All, CL14417.Contig2_All, CL14429.Contig1_All, CL14431.Contig1_All, CL14441.Contig1_All, CL14465.Contig1_All, CL14484.Contig1_All, CL14499.Contig1_All, CL14517.Contig1_All, CL1452.Contig1_All, CL1452.Contig2_All, CL1452.Contig3_All, CL14541.Contig1_All, CL14611.Contig1_All, CL14625.Contig1_All, CL14637.Contig1_All, CL14642.Contig1_All, CL14643.Contig1_All, CL14682.Contig1_All, CL14705.Contig1_All, CL1471.Contig1_All, CL1471.Contig2_All, CL1471.Contig3_All, CL1471.Contig4_All, CL1471.Contig5_All, CL14729.Contig1_All, CL14730.Contig1_All, CL14730.Contig2_All, CL14800.Contig1_All, CL14872.Contig1_All, CL14885.Contig1_All, CL14893.Contig1_All, CL149.Contig10_All, CL149.Contig2_All, CL149.Contig8_All, CL14942.Contig1_All, CL14963.Contig1_All, CL14993.Contig1_All, CL15036.Contig1_All, CL15052.Contig1_All, CL15087.Contig1_All, CL15088.Contig1_All, CL15102.Contig1_All, CL15108.Contig1_All, CL15115.Contig1_All,

		CL1513.Contig1_All, CL1513.Contig2_All, CL1513.Contig3_All, CL1513.Contig4_All, CL1513.Contig5_All, CL15139.Contig1_All, CL15200.Contig1_All, CL15270.Contig1_All, CL15281.Contig1_All, CL15292.Contig1_All, CL15326.Contig1_All, CL15395.Contig1_All, CL15453.Contig1_All, CL15460.Contig1_All, CL15471.Contig1_All, CL15477.Contig1_All, CL15484.Contig1_All, CL15513.Contig1_All, CL15515.Contig1_All, CL15543.Contig1_All, CL15574.Contig1_All, CL15587.Contig1_All, CL15601.Contig1_All, CL15719.Contig1_All, CL15741.Contig1_All, CL15755.Contig1_All, CL15833.Contig1_All, CL15917.Contig1_All, CL16022.Contig1_All, CL16041.Contig1_All, CL16044.Contig1_All, CL16088.Contig1_All, CL1626.Contig1_All, CL1626.Contig2_All, CL1626.Contig3_All, CL1626.Contig4_All, CL16412.Contig1_All, CL16490.Contig1_All, CL16553.Contig1_All, CL16554.Contig1_All, CL16590.Contig1_All, CL16635.Contig1_All, CL16645.Contig1_All, CL16812.Contig1_All, CL16938.Contig1_All, CL16948.Contig1_All, CL16955.Contig1_All, CL17011.Contig1_All, CL1715.Contig1_All, CL1715.Contig2_All, CL1715.Contig3_All, CL1715.Contig4_All, CL17237.Contig1_All, CL17282.Contig1_All, CL17375.Contig1_All, CL17842.Contig1_All, CL17963.Contig1_All, CL18011.Contig1_All, CL18119.Contig1_All, CL18180.Contig1_All, CL1821.Contig1_All, CL1821.Contig2_All, CL1821.Contig3_All, CL1821.Contig4_All, CL18815.Contig1_All, CL19025.Contig1_All, CL19025.Contig2_All, CL19087.Contig1_All, CL19146.Contig1_All, CL19152.Contig1_All, CL19185.Contig1_All, CL19268.Contig1_All, CL19535.Contig1_All, CL19589.Contig1_All, CL1983.Contig1_All, CL19889.Contig1_All, CL201.Contig2_All, CL201.Contig6_All, CL20292.Contig1_All, CL20517.Contig1_All, CL20677.Contig1_All, CL20729.Contig1_All, CL20887.Contig1_All, CL21001.Contig1_All, CL21035.Contig1_All, CL21092.Contig1_All, CL21095.Contig1_All, CL21197.Contig1_All, CL21197.Contig2_All, CL21206.Contig1_All, CL21233.Contig1_All, CL21295.Contig1_All, CL21528.Contig1_All, CL21630.Contig1_All, CL21708.Contig1_All, CL21766.Contig1_All, CL21776.Contig1_All, CL21942.Contig1_All, CL22061.Contig1_All, CL22137.Contig1_All, CL22360.Contig1_All, CL2237.Contig1_All, CL2237.Contig2_All, CL2237.Contig3_All, CL2237.Contig4_All, CL22567.Contig1_All, CL22585.Contig1_All, CL22656.Contig1_All, CL22715.Contig1_All,

		CL22722.Contig1_All, CL22859.Contig1_All, CL22892.Contig1_All, CL2300.Contig1_All, CL23216.Contig1_All, CL23217.Contig1_All, CL23332.Contig1_All, CL23448.Contig1_All, CL23849.Contig1_All, CL23938.Contig1_All, CL24035.Contig1_All, CL24089.Contig1_All, CL24241.Contig1_All, CL24376.Contig1_All, CL24462.Contig1_All, CL24506.Contig1_All, CL24552.Contig1_All, CL24674.Contig1_All, CL24736.Contig1_All, CL24814.Contig1_All, CL24842.Contig1_All, CL24875.Contig1_All, CL24896.Contig1_All, CL24905.Contig1_All, CL24931.Contig1_All, CL24943.Contig1_All, CL24974.Contig1_All, CL25018.Contig1_All, CL25086.Contig1_All, CL25212.Contig1_All, CL25295.Contig1_All, CL25331.Contig1_All, CL25356.Contig1_All, CL25394.Contig1_All, CL25398.Contig1_All, CL25531.Contig1_All, CL25708.Contig1_All, CL25716.Contig1_All, CL2574.Contig1_All, CL2574.Contig2_All, CL2574.Contig3_All, CL25804.Contig1_All, CL25822.Contig1_All, CL25986.Contig1_All, CL25991.Contig1_All, CL26020.Contig1_All, CL26037.Contig1_All, CL26041.Contig1_All, CL26063.Contig1_All, CL26110.Contig1_All, CL26127.Contig1_All, CL26129.Contig1_All, CL26139.Contig1_All, CL26164.Contig1_All, CL26211.Contig1_All, CL26284.Contig1_All, CL26327.Contig1_All, CL26339.Contig1_All, CL2642.Contig1_All, CL2642.Contig2_All, CL2642.Contig3_All, CL26490.Contig1_All, CL26591.Contig1_All, CL26609.Contig1_All, CL26615.Contig1_All, CL26618.Contig1_All, CL26696.Contig1_All, CL26721.Contig1_All, CL26725.Contig1_All, CL26779.Contig1_All, CL26797.Contig1_All, CL26800.Contig1_All, CL26931.Contig1_All, CL2711.Contig1_All, CL27132.Contig1_All, CL27155.Contig1_All, CL27158.Contig1_All, CL27194.Contig1_All, CL27198.Contig1_All, CL27360.Contig1_All, CL27419.Contig1_All, CL27485.Contig1_All, CL27550.Contig1_All, CL27589.Contig1_All, CL27645.Contig1_All, CL27659.Contig1_All, CL27777.Contig1_All, CL278.Contig4_All, CL278.Contig5_All, CL27817.Contig1_All, CL27830.Contig1_All, CL27842.Contig1_All, CL27875.Contig1_All, CL27990.Contig1_All, CL27996.Contig1_All, CL28020.Contig1_All, CL28036.Contig1_All, CL28106.Contig1_All, CL28124.Contig1_All, CL2871.Contig1_All, CL2871.Contig2_All, CL2871.Contig3_All, CL2953.Contig2_All, CL2961.Contig1_All, CL2961.Contig2_All, CL2961.Contig3_All, CL3275.Contig1_All, CL3275.Contig2_All, CL3361.Contig1_All,

		CL3697.Contig1_All, CL3697.Contig2_All, CL3708.Contig1_All, CL3708.Contig2_All, CL3775.Contig1_All, CL3775.Contig2_All, CL3790.Contig1_All, CL3790.Contig2_All, CL4005.Contig1_All, CL401.Contig1_All, CL401.Contig2_All, CL401.Contig3_All, CL401.Contig4_All, CL4082.Contig1_All, CL4082.Contig2_All, CL4163.Contig1_All, CL4293.Contig1_All, CL4293.Contig2_All, CL432.Contig2_All, CL4448.Contig1_All, CL4448.Contig2_All, CL4547.Contig1_All, CL4547.Contig2_All, CL4584.Contig1_All, CL4584.Contig2_All, CL4584.Contig3_All, CL4677.Contig1_All, CL4774.Contig1_All, CL4774.Contig2_All, CL4819.Contig1_All, CL4819.Contig2_All, CL482.Contig5_All, CL4931.Contig2_All, CL4931.Contig3_All, CL4945.Contig1_All, CL5174.Contig1_All, CL5174.Contig2_All, CL5174.Contig3_All, CL5472.Contig1_All, CL5472.Contig2_All, CL5698.Contig1_All, CL5698.Contig2_All, CL58.Contig1_All, CL58.Contig3_All, CL58.Contig7_All, CL5875.Contig1_All, CL5875.Contig2_All, CL5928.Contig1_All, CL5944.Contig1_All, CL6080.Contig1_All, CL6159.Contig1_All, CL6185.Contig1_All, CL6358.Contig1_All, CL6358.Contig2_All, CL6531.Contig1_All, CL6563.Contig1_All, CL6754.Contig1_All, CL6754.Contig2_All, CL6854.Contig1_All, CL6854.Contig2_All, CL6884.Contig1_All, CL6884.Contig2_All, CL6930.Contig1_All, CL7190.Contig1_All, CL7311.Contig1_All, CL7588.Contig1_All, CL77.Contig3_All, CL77.Contig4_All, CL77.Contig6_All, CL7700.Contig1_All, CL7826.Contig1_All, CL7839.Contig1_All, CL7844.Contig1_All, CL8044.Contig1_All, CL8044.Contig2_All, CL8140.Contig1_All, CL8140.Contig2_All, CL8183.Contig1_All, CL8252.Contig1_All, CL8502.Contig1_All, CL851.Contig1_All, CL851.Contig2_All, CL851.Contig3_All, CL8615.Contig2_All, CL8834.Contig1_All, CL8835.Contig1_All, CL8835.Contig2_All, CL903.Contig1_All, CL903.Contig2_All, CL903.Contig3_All, CL903.Contig4_All, CL9045.Contig1_All, CL9045.Contig2_All, CL9113.Contig1_All, CL9381.Contig1_All, CL9381.Contig2_All, CL9402.Contig1_All, CL9402.Contig2_All, CL9595.Contig1_All, CL9595.Contig2_All, CL96.Contig3_All, CL9659.Contig1_All, CL9827.Contig1_All, Unigene1236_All, Unigene1237_All, Unigene1784_All, Unigene1798_All, Unigene191_All, Unigene2040_All, Unigene2293_All, Unigene2300_All, Unigene2301_All, Unigene2381_All, Unigene3048_All, Unigene3059_All, Unigene3078_All, Unigene319_All, Unigene372_All, Unigene4443_All, Unigene4620_All, Unigene4684_All, Unigene4856_All, Unigene5061_All, Unigene5367_All, Unigene5414_All, Unigene5477_All, Unigene5512_All, Unigene5515_All, Unigene568_All, Unigene692_All, Unigene693_All
10		

	Glycerophospholipid	CL1.Contig120_All, CL1.Contig123_All, CL1.Contig130_All, CL1.Contig23_All, CL1.Contig45_All, CL1.Contig46_All, CL1.Contig57_All, CL1.Contig59_All, CL1.Contig63_All, CL1.Contig91_All, CL1.Contig94_All, CL10406.Contig1_All, CL10416.Contig1_All, CL10416.Contig2_All, CL10416.Contig3_All, CL10417.Contig1_All, CL10483.Contig1_All, CL10515.Contig1_All, CL10535.Contig1_All, CL10577.Contig1_All, CL10621.Contig1_All, CL10624.Contig1_All, CL10629.Contig1_All, CL10675.Contig1_All, CL10678.Contig1_All, CL10686.Contig1_All, CL1069.Contig1_All, CL10716.Contig1_All, CL1074.Contig1_All, CL1074.Contig2_All, CL10932.Contig1_All, CL11041.Contig1_All, CL11094.Contig1_All, CL11145.Contig1_All, CL11160.Contig1_All, CL11236.Contig1_All, CL11261.Contig1_All, CL11423.Contig1_All, CL11477.Contig1_All, CL11484.Contig1_All, CL11520.Contig1_All, CL11580.Contig1_All, CL11616.Contig1_All, CL11631.Contig1_All, CL11672.Contig1_All, CL1173.Contig1_All, CL1175.Contig1_All, CL1196.Contig4_All, CL11980.Contig1_All, CL1207.Contig1_All, CL1207.Contig2_All, CL1207.Contig3_All, CL1207.Contig4_All, CL12095.Contig1_All, CL12197.Contig1_All, CL12210.Contig1_All, CL1231.Contig3_All, CL12715.Contig1_All, CL13022.Contig1_All, CL13169.Contig1_All, CL13194.Contig1_All, CL1328.Contig1_All, CL13297.Contig2_All, CL13357.Contig1_All, CL13410.Contig1_All, CL13666.Contig1_All, CL13717.Contig1_All, CL13717.Contig2_All, CL1378.Contig1_All, CL1378.Contig2_All, CL13818.Contig1_All, CL13877.Contig1_All, CL13975.Contig1_All, CL13975.Contig2_All, CL14003.Contig1_All, CL14008.Contig1_All, CL1406.Contig2_All, CL1406.Contig3_All, CL14112.Contig1_All, CL14310.Contig1_All, CL14313.Contig1_All, CL14326.Contig1_All, CL14365.Contig1_All, CL14392.Contig1_All, CL14416.Contig1_All, CL14422.Contig1_All, CL14487.Contig1_All, CL14558.Contig1_All, CL14732.Contig1_All, CL14738.Contig1_All, CL14752.Contig1_All, CL14764.Contig1_All, CL14877.Contig1_All, CL1496.Contig1_All, CL14966.Contig1_All, CL151.Contig2_All, CL151.Contig4_All, CL15160.Contig1_All, CL15163.Contig1_All, CL15207.Contig1_All, CL15251.Contig1_All, CL15301.Contig1_All, CL15341.Contig1_All, CL15591.Contig1_All, CL15643.Contig1_All, CL15700.Contig1_All, CL15700.Contig2_All, CL15822.Contig1_All, CL15849.Contig1_All, CL16059.Contig1_All, CL1609.Contig1_All, CL1609.Contig4_All, CL16126.Contig1_All,

	metabolism	CL16330.Contig1_All, CL16345.Contig1_All, CL16706.Contig1_All, CL1675.Contig1_All, CL1679.Contig1_All, CL171.Contig6_All, CL1711.Contig2_All, CL17263.Contig1_All, CL17278.Contig1_All, CL17278.Contig2_All, CL17326.Contig1_All, CL1734.Contig1_All, CL17382.Contig1_All, CL17413.Contig1_All, CL17426.Contig1_All, CL17732.Contig1_All, CL1791.Contig1_All, CL18074.Contig1_All, CL18194.Contig1_All, CL18212.Contig1_All, CL18214.Contig1_All, CL1830.Contig1_All, CL18308.Contig1_All, CL18351.Contig1_All, CL18369.Contig1_All, CL18429.Contig1_All, CL1858.Contig1_All, CL18751.Contig1_All, CL18800.Contig1_All, CL18816.Contig1_All, CL19050.Contig1_All, CL1917.Contig1_All, CL19337.Contig1_All, CL19427.Contig1_All, CL19434.Contig1_All, CL19499.Contig1_All, CL19523.Contig1_All, CL19670.Contig1_All, CL19701.Contig1_All, CL1978.Contig1_All, CL19848.Contig1_All, CL1999.Contig1_All, CL1999.Contig2_All, CL20376.Contig1_All, CL20397.Contig1_All, CL20474.Contig1_All, CL20504.Contig1_All, CL20578.Contig1_All, CL20606.Contig1_All, CL20608.Contig1_All, CL20624.Contig1_All, CL20680.Contig1_All, CL20703.Contig1_All, CL20826.Contig1_All, CL209.Contig4_All, CL21089.Contig1_All, CL21093.Contig1_All, CL21148.Contig1_All, CL2116.Contig1_All, CL21173.Contig1_All, CL21227.Contig1_All, CL21321.Contig1_All, CL21335.Contig1_All, CL21393.Contig1_All, CL21451.Contig1_All, CL21453.Contig1_All, CL21562.Contig1_All, CL21576.Contig1_All, CL2160.Contig1_All, CL21715.Contig1_All, CL2181.Contig1_All, CL2181.Contig2_All, CL2181.Contig3_All, CL21819.Contig1_All, CL2192.Contig1_All, CL2192.Contig3_All, CL21955.Contig1_All, CL22085.Contig1_All, CL22136.Contig1_All, CL22152.Contig1_All, CL22314.Contig1_All, CL22377.Contig1_All, CL22380.Contig1_All, CL22461.Contig1_All, CL22836.Contig1_All, CL22977.Contig1_All, CL23055.Contig1_All, CL2314.Contig1_All, CL23284.Contig1_All, CL23358.Contig1_All, CL2353.Contig1_All, CL2353.Contig4_All, CL2355.Contig1_All, CL23588.Contig1_All, CL2387.Contig1_All, CL2391.Contig1_All, CL23965.Contig1_All, CL24251.Contig1_All, CL24281.Contig1_All, CL24479.Contig1_All, CL24524.Contig1_All, CL24831.Contig1_All, CL25143.Contig1_All, CL2515.Contig1_All, CL25220.Contig1_All, CL25277.Contig1_All, CL25363.Contig1_All, CL25376.Contig1_All, CL25479.Contig1_All,

			CL25710.Contig1_All, CL2579.Contig1_All, CL26163.Contig1_All, CL2630.Contig1_All, CL2630.Contig2_All, CL26309.Contig1_All, CL26483.Contig1_All, CL26501.Contig1_All, CL26610.Contig1_All, CL26645.Contig1_All, CL26978.Contig1_All, CL27010.Contig1_All, CL27661.Contig1_All, CL27703.Contig1_All, CL27802.Contig1_All, CL2860.Contig3_All, CL288.Contig3_All, CL288.Contig8_All, CL293.Contig1_All, CL293.Contig3_All, CL293.Contig4_All, CL293.Contig5_All, CL293.Contig6_All, CL293.Contig7_All, CL2961.Contig4_All, CL3103.Contig1_All, CL3186.Contig1_All, CL3260.Contig1_All, CL3264.Contig1_All, CL3264.Contig2_All, CL3285.Contig1_All, CL3285.Contig2_All, CL3308.Contig2_All, CL331.Contig2_All, CL331.Contig4_All, CL35.Contig11_All, CL35.Contig16_All, CL35.Contig1_All, CL35.Contig22_All, CL35.Contig4_All, CL35.Contig6_All, CL35.Contig8_All, CL351.Contig5_All, CL3570.Contig1_All, CL3646.Contig1_All, CL3646.Contig2_All, CL3695.Contig1_All, CL374.Contig5_All, CL3840.Contig1_All, CL385.Contig1_All, CL385.Contig2_All, CL385.Contig3_All, CL388.Contig1_All, CL388.Contig2_All, CL388.Contig3_All, CL388.Contig4_All, CL388.Contig5_All, CL3884.Contig1_All, CL3932.Contig1_All, CL3938.Contig1_All, CL3938.Contig2_All, CL3938.Contig3_All, CL3943.Contig1_All, CL3957.Contig1_All, CL3957.Contig2_All, CL4.Contig32_All, CL4.Contig35_All, CL4.Contig4_All, CL4.Contig5_All, CL4062.Contig1_All, CL4062.Contig2_All, CL4077.Contig1_All, CL4190.Contig1_All, CL4190.Contig2_All, CL4295.Contig1_All, CL4295.Contig2_All, CL4305.Contig1_All, CL4339.Contig1_All, CL4342.Contig1_All, CL4362.Contig1_All, CL4630.Contig1_All, CL468.Contig1_All, CL468.Contig2_All, CL468.Contig3_All, CL4685.Contig1_All, CL4723.Contig2_All, CL4892.Contig1_All, CL4892.Contig2_All, CL4946.Contig2_All, CL4946.Contig3_All, CL5036.Contig1_All, CL5068.Contig1_All, CL5122.Contig1_All, CL518.Contig1_All, CL518.Contig2_All, CL518.Contig3_All, CL518.Contig4_All, CL518.Contig5_All, CL5210.Contig1_All, CL5277.Contig1_All, CL5317.Contig1_All, CL5321.Contig1_All, CL535.Contig1_All, CL5359.Contig1_All, CL537.Contig2_All, CL537.Contig5_All, CL5393.Contig1_All, CL5401.Contig1_All, CL5475.Contig1_All, CL55.Contig2_All, CL55.Contig4_All, CL5870.Contig1_All, CL589.Contig1_All, CL589.Contig2_All, CL589.Contig3_All, CL5950.Contig1_All, CL6.Contig1_All, CL6.Contig25_All, CL6002.Contig1_All, CL6002.Contig2_All, CL6009.Contig1_All, CL6009.Contig2_All, CL606.Contig1_All, CL6085.Contig1_All, CL6096.Contig1_All, CL6155.Contig1_All, CL625.Contig1_All, CL6263.Contig1_All, CL6263.Contig2_All, CL6377.Contig1_All, CL6388.Contig1_All, CL6442.Contig1_All, CL6506.Contig1_All, CL6538.Contig1_All,

CL6548.Contig1_All, CL6581.Contig1_All,
CL6617.Contig1_All, CL6617.Contig2_All,
CL6696.Contig1_All, CL674.Contig1_All, CL674.Contig2_All,
CL674.Contig3_All, CL674.Contig4_All, CL674.Contig5_All,
CL6913.Contig1_All, CL7.Contig28_All, CL70.Contig8_All,
CL7030.Contig1_All, CL7181.Contig1_All,
CL7240.Contig1_All, CL7240.Contig2_All,
CL7255.Contig1_All, CL7276.Contig1_All,
CL7276.Contig2_All, CL7298.Contig1_All,
CL7329.Contig1_All, CL748.Contig1_All, CL748.Contig2_All,
CL748.Contig3_All, CL748.Contig4_All, CL7484.Contig2_All,
CL7557.Contig1_All, CL7597.Contig2_All, CL761.Contig1_All,
CL761.Contig2_All, CL761.Contig3_All, CL761.Contig4_All,
CL7615.Contig2_All, CL7941.Contig1_All,
CL7959.Contig1_All, CL7961.Contig1_All,
CL8001.Contig1_All, CL8008.Contig1_All,
CL8159.Contig1_All, CL820.Contig1_All, CL820.Contig2_All,
CL8225.Contig1_All, CL8239.Contig1_All,
CL8299.Contig1_All, CL8339.Contig1_All,
CL8375.Contig1_All, CL8439.Contig1_All,
CL8468.Contig1_All, CL8468.Contig2_All,
CL8562.Contig1_All, CL8608.Contig1_All,
CL8608.Contig2_All, CL864.Contig1_All, CL864.Contig3_All,
CL8643.Contig1_All, CL8643.Contig2_All, CL878.Contig1_All,
CL8814.Contig1_All, CL8918.Contig1_All,
CL8932.Contig1_All, CL9190.Contig1_All,
CL9408.Contig1_All, CL946.Contig1_All, CL946.Contig2_All,
CL946.Contig3_All, CL946.Contig4_All, CL9508.Contig1_All,
CL9568.Contig1_All, CL9673.Contig1_All,
CL9724.Contig1_All, CL9741.Contig1_All,
CL9767.Contig1_All, Unigene1043_All, Unigene1053_All,
Unigene1153_All, Unigene1207_All, Unigene1262_All,
Unigene1274_All, Unigene1294_All, Unigene1327_All,
Unigene1329_All, Unigene1683_All, Unigene1782_All,
Unigene1783_All, Unigene1819_All, Unigene1835_All,
Unigene185_All, Unigene1907_All, Unigene1931_All,
Unigene2218_All, Unigene2250_All, Unigene2282_All,
Unigene2292_All, Unigene2340_All, Unigene248_All,
Unigene2510_All, Unigene255_All, Unigene2560_All,
Unigene2613_All, Unigene2681_All, Unigene2709_All,
Unigene2741_All, Unigene3023_All, Unigene3036_All,
Unigene3077_All, Unigene3079_All, Unigene3101_All,
Unigene3757_All, Unigene3774_All, Unigene4316_All,
Unigene4371_All, Unigene4372_All, Unigene4403_All,
Unigene4406_All, Unigene4427_All, Unigene4439_All,
Unigene4464_All, Unigene4503_All, Unigene4628_All,
Unigene4669_All, Unigene4679_All, Unigene4680_All,
Unigene4685_All, Unigene4687_All, Unigene4688_All,
Unigene4741_All, Unigene4805_All, Unigene4810_All,
Unigene484_All, Unigene4908_All, Unigene4953_All,
Unigene5219_All, Unigene5291_All, Unigene5339_All,
Unigene5362_All, Unigene5377_All, Unigene5410_All,
Unigene5413_All, Unigene5437_All, Unigene560_All,
Unigene576_All, Unigene607_All, Unigene882_All,

		Unigene939_All
11		CL1.Contig127_All, CL1.Contig77_All, CL1.Contig92_All, CL1.Contig98_All, CL1004.Contig1_All, CL1004.Contig2_All, CL1004.Contig3_All, CL1004.Contig4_All, CL10405.Contig1_All, CL10445.Contig1_All, CL1058.Contig1_All, CL1058.Contig2_All, CL1058.Contig3_All, CL10602.Contig1_All, CL10833.Contig1_All, CL10903.Contig1_All, CL11.Contig14_All, CL11.Contig20_All, CL11.Contig4_All, CL11.Contig8_All, CL11074.Contig1_All, CL11291.Contig1_All, CL11346.Contig1_All, CL11359.Contig1_All, CL11478.Contig1_All, CL11573.Contig1_All, CL1182.Contig1_All, CL1182.Contig2_All, CL11830.Contig1_All, CL11860.Contig1_All, CL11885.Contig1_All, CL11980.Contig1_All, CL12040.Contig1_All, CL12040.Contig2_All, CL1208.Contig1_All, CL12140.Contig1_All, CL1229.Contig1_All, CL1229.Contig2_All, CL12316.Contig1_All, CL12451.Contig1_All, CL125.Contig3_All, CL125.Contig4_All, CL125.Contig5_All, CL125.Contig6_All, CL125.Contig7_All, CL12650.Contig1_All, CL12726.Contig1_All, CL12911.Contig1_All, CL12952.Contig1_All, CL13016.Contig1_All, CL13105.Contig1_All, CL13218.Contig1_All, CL13248.Contig2_All, CL13296.Contig1_All, CL13420.Contig1_All, CL13570.Contig1_All, CL13607.Contig1_All, CL13767.Contig1_All, CL13785.Contig1_All, CL13802.Contig1_All, CL13839.Contig1_All, CL139.Contig2_All, CL139.Contig6_All, CL13950.Contig1_All, CL13961.Contig1_All, CL14331.Contig1_All, CL14348.Contig1_All, CL14419.Contig1_All, CL14428.Contig1_All, CL14428.Contig2_All, CL14430.Contig1_All, CL14456.Contig1_All, CL14525.Contig1_All, CL14810.Contig1_All, CL14845.Contig1_All, CL14911.Contig1_All, CL1501.Contig1_All, CL1501.Contig2_All, CL15081.Contig1_All, CL15126.Contig1_All, CL15154.Contig1_All, CL15307.Contig1_All, CL15381.Contig1_All, CL15423.Contig1_All, CL15458.Contig1_All, CL15548.Contig1_All, CL15610.Contig1_All, CL15763.Contig1_All, CL15829.Contig1_All, CL15911.Contig1_All, CL15985.Contig1_All, CL1609.Contig1_All, CL1609.Contig2_All, CL1609.Contig3_All, CL1613.Contig1_All, CL1613.Contig2_All, CL1613.Contig4_All, CL1617.Contig1_All, CL16272.Contig1_All, CL16311.Contig1_All, CL16337.Contig1_All, CL1637.Contig1_All, CL16409.Contig1_All, CL16429.Contig1_All, CL16469.Contig1_All, CL16567.Contig1_All, CL16668.Contig1_All, CL16913.Contig1_All, CL16952.Contig1_All, CL16986.Contig1_All, CL16999.Contig1_All, CL16999.Contig2_All,

	[Ribosome biogenesis in eukaryotes]()	CL17142.Contig1_All, CL172.Contig1_All, CL172.Contig2_All, CL172.Contig3_All, CL172.Contig5_All, CL172.Contig6_All, CL172.Contig7_All, CL172.Contig8_All, CL172.Contig9_All, CL1720.Contig1_All, CL17246.Contig1_All, CL17360.Contig1_All, CL17491.Contig1_All, CL17658.Contig1_All, CL1786.Contig1_All, CL1786.Contig2_All, CL17934.Contig1_All, CL17946.Contig1_All, CL17968.Contig1_All, CL18055.Contig1_All, CL18165.Contig1_All, CL18207.Contig1_All, CL1823.Contig1_All, CL18239.Contig1_All, CL18270.Contig1_All, CL18308.Contig1_All, CL18343.Contig1_All, CL18391.Contig1_All, CL18475.Contig1_All, CL18478.Contig1_All, CL18533.Contig1_All, CL18545.Contig1_All, CL186.Contig2_All, CL186.Contig3_All, CL186.Contig4_All, CL186.Contig6_All, CL18603.Contig1_All, CL18735.Contig1_All, CL18833.Contig1_All, CL18835.Contig1_All, CL18846.Contig1_All, CL19082.Contig1_All, CL19118.Contig1_All, CL19190.Contig1_All, CL19204.Contig1_All, CL19301.Contig1_All, CL19317.Contig1_All, CL19333.Contig1_All, CL19335.Contig1_All, CL1941.Contig1_All, CL19411.Contig1_All, CL19427.Contig1_All, CL19464.Contig1_All, CL195.Contig1_All, CL195.Contig2_All, CL195.Contig3_All, CL195.Contig4_All, CL195.Contig5_All, CL1954.Contig1_All, CL19731.Contig1_All, CL19934.Contig1_All, CL19962.Contig1_All, CL20004.Contig1_All, CL20055.Contig1_All, CL20190.Contig1_All, CL2022.Contig1_All, CL20420.Contig1_All, CL20499.Contig1_All, CL20524.Contig1_All, CL20533.Contig1_All, CL20610.Contig1_All, CL20658.Contig1_All, CL21.Contig10_All, CL21.Contig11_All, CL21.Contig1_All, CL21.Contig2_All, CL21.Contig3_All, CL21.Contig5_All, CL21.Contig6_All, CL21.Contig7_All, CL21.Contig8_All, CL21.Contig9_All, CL21055.Contig1_All, CL21069.Contig1_All, CL21159.Contig1_All, CL21229.Contig1_All, CL21546.Contig1_All, CL2168.Contig1_All, CL21752.Contig1_All, CL21756.Contig1_All, CL21814.Contig1_All, CL21845.Contig1_All, CL22067.Contig1_All, CL2243.Contig1_All, CL22459.Contig1_All, CL22467.Contig1_All, CL2251.Contig1_All, CL22642.Contig1_All, CL22647.Contig1_All, CL22723.Contig1_All, CL22749.Contig1_All, CL22802.Contig1_All, CL22826.Contig1_All, CL22949.Contig1_All, CL22991.Contig1_All, CL23030.Contig1_All, CL23175.Contig1_All, CL23183.Contig1_All, CL2320.Contig1_All, CL2320.Contig2_All, CL23322.Contig1_All, CL2333.Contig1_All, CL23411.Contig1_All, CL23539.Contig1_All, CL23682.Contig1_All, CL23783.Contig1_All, CL23822.Contig1_All, CL23827.Contig1_All, CL23891.Contig1_All,

			CL23892.Contig1_All, CL2417.Contig1_All, CL24588.Contig1_All, CL246.Contig1_All, CL246.Contig2_All, CL246.Contig3_All, CL246.Contig4_All, CL25063.Contig1_All, CL25077.Contig1_All, CL25080.Contig1_All, CL25251.Contig1_All, CL25320.Contig1_All, CL25366.Contig1_All, CL25785.Contig1_All, CL25854.Contig1_All, CL25883.Contig1_All, CL25900.Contig1_All, CL25925.Contig1_All, CL25932.Contig1_All, CL26.Contig4_All, CL26.Contig6_All, CL26122.Contig1_All, CL26149.Contig1_All, CL26190.Contig1_All, CL26282.Contig1_All, CL26325.Contig1_All, CL26457.Contig1_All, CL26570.Contig1_All, CL26615.Contig1_All, CL26652.Contig1_All, CL26684.Contig1_All, CL26695.Contig1_All, CL26708.Contig1_All, CL2675.Contig1_All, CL26861.Contig1_All, CL27086.Contig1_All, CL27104.Contig1_All, CL27151.Contig1_All, CL272.Contig1_All, CL27286.Contig1_All, CL27451.Contig1_All, CL275.Contig2_All, CL2784.Contig1_All, CL2785.Contig1_All, CL2812.Contig1_All, CL290.Contig6_All, CL314.Contig1_All, CL314.Contig2_All, CL314.Contig3_All, CL314.Contig4_All, CL314.Contig5_All, CL3216.Contig1_All, CL3335.Contig1_All, CL3389.Contig1_All, CL3422.Contig1_All, CL3422.Contig2_All, CL3422.Contig3_All, CL3467.Contig1_All, CL3490.Contig1_All, CL3544.Contig1_All, CL3544.Contig2_All, CL3703.Contig1_All, CL3723.Contig1_All, CL3723.Contig2_All, CL3755.Contig1_All, CL3814.Contig1_All, CL3823.Contig1_All, CL3832.Contig1_All, CL3832.Contig2_All, CL4050.Contig1_All, CL4234.Contig1_All, CL4249.Contig1_All, CL432.Contig4_All, CL4360.Contig1_All, CL4360.Contig2_All, CL450.Contig2_All, CL450.Contig3_All, CL450.Contig4_All, CL450.Contig5_All, CL4539.Contig1_All, CL455.Contig1_All, CL455.Contig2_All, CL455.Contig3_All, CL455.Contig4_All, CL455.Contig5_All, CL455.Contig6_All, CL4581.Contig1_All, CL4641.Contig1_All, CL4641.Contig2_All, CL4644.Contig1_All, CL5.Contig25_All, CL5.Contig29_All, CL5.Contig4_All, CL5027.Contig1_All, CL5040.Contig1_All, CL5099.Contig1_All, CL5117.Contig1_All, CL5118.Contig1_All, CL5301.Contig1_All, CL5355.Contig1_All, CL5390.Contig1_All, CL5442.Contig1_All, CL5547.Contig1_All, CL557.Contig1_All, CL557.Contig2_All, CL557.Contig3_All, CL557.Contig4_All, CL5573.Contig1_All, CL5688.Contig1_All, CL5713.Contig1_All, CL5713.Contig2_All, CL5732.Contig1_All, CL5790.Contig1_All, CL5790.Contig2_All, CL5790.Contig3_All, CL5790.Contig4_All, CL59.Contig5_All, CL59.Contig7_All, CL59.Contig8_All, CL6015.Contig1_All, CL6149.Contig1_All, CL6238.Contig1_All, CL6270.Contig1_All, CL6306.Contig1_All, CL6334.Contig1_All, CL6354.Contig1_All,

			CL6361.Contig1_All, CL6415.Contig1_All, CL6559.Contig1_All, CL6619.Contig1_All, CL663.Contig3_All, CL6695.Contig1_All, CL6701.Contig2_All, CL6742.Contig1_All, CL6768.Contig1_All, CL6768.Contig2_All, CL6768.Contig3_All, CL6779.Contig1_All, CL6886.Contig1_All, CL6934.Contig1_All, CL6939.Contig1_All, CL7.Contig5_All, CL7004.Contig1_All, CL7004.Contig2_All, CL719.Contig2_All, CL719.Contig4_All, CL719.Contig7_All, CL719.Contig8_All, CL7206.Contig1_All, CL7236.Contig1_All, CL747.Contig1_All, CL75.Contig11_All, CL75.Contig12_All, CL75.Contig13_All, CL75.Contig14_All, CL75.Contig1_All, CL75.Contig2_All, CL75.Contig4_All, CL75.Contig5_All, CL75.Contig8_All, CL7504.Contig1_All, CL7626.Contig1_All, CL7663.Contig1_All, CL7671.Contig1_All, CL7673.Contig1_All, CL7865.Contig1_All, CL7962.Contig1_All, CL7962.Contig2_All, CL7991.Contig1_All, CL80.Contig8_All, CL80.Contig9_All, CL8081.Contig1_All, CL8387.Contig1_All, CL8411.Contig1_All, CL8444.Contig1_All, CL8553.Contig1_All, CL8591.Contig1_All, CL8623.Contig1_All, CL8661.Contig1_All, CL8711.Contig1_All, CL8711.Contig2_All, CL8726.Contig1_All, CL8832.Contig1_All, CL8895.Contig1_All, CL8895.Contig2_All, CL9001.Contig1_All, CL9212.Contig1_All, CL9370.Contig1_All, CL956.Contig1_All, CL968.Contig3_All, CL9680.Contig1_All, CL9732.Contig1_All, CL9802.Contig2_All, CL9816.Contig1_All, CL9934.Contig1_All, CL9986.Contig1_All, Unigene1059_All, Unigene1092_All, Unigene1094_All, Unigene1272_All, Unigene1273_All, Unigene1364_All, Unigene1408_All, Unigene1528_All, Unigene1549_All, Unigene1735_All, Unigene1768_All, Unigene1944_All, Unigene201_All, Unigene214_All, Unigene2270_All, Unigene2379_All, Unigene2436_All, Unigene243_All, Unigene2521_All, Unigene2522_All, Unigene2553_All, Unigene2582_All, Unigene2805_All, Unigene2995_All, Unigene3036_All, Unigene3111_All, Unigene3119_All, Unigene3177_All, Unigene3255_All, Unigene3258_All, Unigene3264_All, Unigene3270_All, Unigene3301_All, Unigene3353_All, Unigene3365_All, Unigene3366_All, Unigene3480_All, Unigene3787_All, Unigene398_All, Unigene3990_All, Unigene4030_All, Unigene4094_All, Unigene4107_All, Unigene4428_All, Unigene446_All, Unigene4488_All, Unigene4789_All, Unigene5307_All, Unigene5475_All, Unigene685_All, Unigene74_All, Unigene948_All
12			CL10155.Contig1_All, CL10198.Contig2_All, CL10235.Contig1_All, CL10247.Contig1_All, CL10347.Contig1_All, CL10394.Contig1_All, CL10414.Contig1_All, CL10495.Contig1_All, CL10496.Contig1_All, CL10557.Contig1_All, CL10565.Contig1_All, CL10565.Contig2_All, CL10587.Contig1_All, CL10691.Contig1_All,

	Starch and sucrose	CL10775.Contig1_All, CL10864.Contig1_All, CL10871.Contig1_All, CL10943.Contig1_All, CL10951.Contig1_All, CL11022.Contig1_All, CL1111.Contig1_All, CL1111.Contig2_All, CL11162.Contig1_All, CL11198.Contig1_All, CL11296.Contig1_All, CL11324.Contig1_All, CL1145.Contig1_All, CL1145.Contig2_All, CL1145.Contig3_All, CL1150.Contig1_All, CL11524.Contig1_All, CL11617.Contig1_All, CL11699.Contig1_All, CL11719.Contig1_All, CL1180.Contig1_All, CL1180.Contig2_All, CL1180.Contig3_All, CL11843.Contig1_All, CL11993.Contig1_All, CL12029.Contig1_All, CL12077.Contig1_All, CL12102.Contig1_All, CL12123.Contig1_All, CL12159.Contig1_All, CL1216.Contig1_All, CL12202.Contig1_All, CL12208.Contig1_All, CL12239.Contig1_All, CL12385.Contig1_All, CL12443.Contig1_All, CL12497.Contig1_All, CL12533.Contig1_All, CL12644.Contig1_All, CL1266.Contig1_All, CL12687.Contig1_All, CL12810.Contig1_All, CL12821.Contig1_All, CL12842.Contig1_All, CL12951.Contig1_All, CL13175.Contig1_All, CL13779.Contig1_All, CL13814.Contig1_All, CL14024.Contig1_All, CL1409.Contig1_All, CL1409.Contig2_All, CL1409.Contig3_All, CL1409.Contig4_All, CL1409.Contig5_All, CL1410.Contig1_All, CL14352.Contig1_All, CL14404.Contig1_All, CL14461.Contig1_All, CL14470.Contig1_All, CL14498.Contig1_All, CL14522.Contig1_All, CL15319.Contig1_All, CL15329.Contig1_All, CL15361.Contig1_All, CL15424.Contig1_All, CL15428.Contig1_All, CL15606.Contig1_All, CL15670.Contig1_All, CL15730.Contig1_All, CL15730.Contig2_All, CL160.Contig3_All, CL16037.Contig1_All, CL16080.Contig1_All, CL16130.Contig1_All, CL16308.Contig1_All, CL16313.Contig1_All, CL1645.Contig1_All, CL16462.Contig1_All, CL16510.Contig1_All, CL16555.Contig1_All, CL16589.Contig1_All, CL1671.Contig1_All, CL16810.Contig1_All, CL17006.Contig1_All, CL17111.Contig1_All, CL17200.Contig1_All, CL17291.Contig1_All, CL17291.Contig2_All, CL17355.Contig1_All, CL17410.Contig1_All, CL17498.Contig1_All, CL17542.Contig1_All, CL17552.Contig1_All, CL17552.Contig2_All, CL178.Contig1_All, CL178.Contig2_All, CL178.Contig3_All, CL178.Contig4_All, CL1802.Contig1_All, CL18183.Contig1_All, CL18626.Contig1_All, CL1868.Contig1_All, CL18792.Contig1_All, CL18822.Contig1_All, CL18832.Contig1_All, CL18898.Contig1_All, CL1904.Contig1_All, CL19110.Contig1_All, CL19267.Contig1_All,

	metabolism	CL1928.Contig2_All, CL19381.Contig1_All, CL19408.Contig1_All, CL1942.Contig1_All, CL19602.Contig1_All, CL19619.Contig1_All, CL19792.Contig1_All, CL198.Contig1_All, CL198.Contig2_All, CL198.Contig3_All, CL198.Contig4_All, CL198.Contig5_All, CL198.Contig6_All, CL198.Contig7_All, CL198.Contig8_All, CL198.Contig9_All, CL19855.Contig1_All, CL1987.Contig1_All, CL19882.Contig1_All, CL20001.Contig1_All, CL20036.Contig1_All, CL2008.Contig1_All, CL20202.Contig1_All, CL20358.Contig1_All, CL20562.Contig1_All, CL20752.Contig1_All, CL20969.Contig1_All, CL21351.Contig1_All, CL21623.Contig1_All, CL21780.Contig1_All, CL2180.Contig1_All, CL21861.Contig1_All, CL21862.Contig1_All, CL22098.Contig1_All, CL22294.Contig1_All, CL22338.Contig1_All, CL2245.Contig1_All, CL2245.Contig2_All, CL22561.Contig1_All, CL2262.Contig1_All, CL2262.Contig2_All, CL2262.Contig3_All, CL2262.Contig4_All, CL22639.Contig1_All, CL22926.Contig1_All, CL23006.Contig1_All, CL2306.Contig1_All, CL23237.Contig1_All, CL23331.Contig1_All, CL23456.Contig1_All, CL23524.Contig1_All, CL23777.Contig1_All, CL24071.Contig1_All, CL24640.Contig1_All, CL24820.Contig1_All, CL2486.Contig1_All, CL24878.Contig1_All, CL24913.Contig1_All, CL25.Contig11_All, CL25.Contig12_All, CL25.Contig14_All, CL25.Contig15_All, CL25.Contig2_All, CL25.Contig9_All, CL250.Contig1_All, CL250.Contig2_All, CL250.Contig3_All, CL250.Contig4_All, CL250.Contig5_All, CL250.Contig6_All, CL2516.Contig1_All, CL25208.Contig1_All, CL2528.Contig1_All, CL2528.Contig2_All, CL25467.Contig1_All, CL25490.Contig1_All, CL25682.Contig1_All, CL257.Contig1_All, CL257.Contig2_All, CL25835.Contig1_All, CL26054.Contig1_All, CL26059.Contig1_All, CL2610.Contig2_All, CL26226.Contig1_All, CL26366.Contig1_All, CL26426.Contig1_All, CL26455.Contig1_All, CL26673.Contig1_All, CL26793.Contig1_All, CL27068.Contig1_All, CL27221.Contig1_All, CL27605.Contig1_All, CL27648.Contig1_All, CL27778.Contig1_All, CL27796.Contig1_All, CL27998.Contig1_All, CL2942.Contig2_All, CL2942.Contig3_All, CL2970.Contig1_All, CL2970.Contig2_All, CL2970.Contig3_All, CL2985.Contig1_All, CL2985.Contig2_All, CL2991.Contig1_All, CL2991.Contig2_All, CL2991.Contig3_All, CL2995.Contig1_All, CL2995.Contig2_All, CL3022.Contig1_All, CL3064.Contig1_All, CL3128.Contig1_All, CL315.Contig1_All, CL315.Contig2_All, CL315.Contig3_All, CL315.Contig4_All, CL315.Contig5_All, CL315.Contig6_All, CL315.Contig7_All, CL315.Contig8_All,

			CL3191.Contig1_All, CL3200.Contig1_All, CL331.Contig1_All, CL331.Contig3_All, CL3310.Contig1_All, CL3334.Contig1_All, CL3456.Contig1_All, CL3466.Contig1_All, CL3466.Contig2_All, CL3542.Contig1_All, CL3770.Contig1_All, CL384.Contig3_All, CL384.Contig8_All, CL3879.Contig1_All, CL3882.Contig1_All, CL3932.Contig2_All, CL412.Contig1_All, CL412.Contig2_All, CL412.Contig3_All, CL4121.Contig1_All, CL4127.Contig1_All, CL4127.Contig2_All, CL4127.Contig3_All, CL424.Contig1_All, CL424.Contig2_All, CL424.Contig3_All, CL424.Contig4_All, CL4490.Contig1_All, CL4520.Contig1_All, CL4717.Contig1_All, CL4731.Contig1_All, CL4758.Contig1_All, CL4832.Contig1_All, CL505.Contig1_All, CL505.Contig2_All, CL505.Contig3_All, CL505.Contig4_All, CL5198.Contig1_All, CL5225.Contig1_All, CL5261.Contig1_All, CL5261.Contig2_All, CL5276.Contig1_All, CL5333.Contig1_All, CL5363.Contig1_All, CL5436.Contig1_All, CL5546.Contig1_All, CL5651.Contig1_All, CL5685.Contig1_All, CL5690.Contig1_All, CL5916.Contig1_All, CL5917.Contig2_All, CL5917.Contig3_All, CL5985.Contig1_All, CL5985.Contig2_All, CL6045.Contig1_All, CL619.Contig1_All, CL6242.Contig1_All, CL6330.Contig1_All, CL651.Contig1_All, CL6558.Contig1_All, CL6589.Contig1_All, CL6700.Contig1_All, CL6782.Contig1_All, CL6791.Contig1_All, CL6799.Contig1_All, CL6799.Contig2_All, CL6838.Contig1_All, CL6970.Contig1_All, CL707.Contig1_All, CL7085.Contig1_All, CL71.Contig7_All, CL7101.Contig1_All, CL7142.Contig1_All, CL7155.Contig1_All, CL7158.Contig1_All, CL7158.Contig2_All, CL717.Contig1_All, CL717.Contig2_All, CL717.Contig3_All, CL717.Contig4_All, CL7381.Contig1_All, CL7387.Contig1_All, CL7448.Contig1_All, CL7463.Contig1_All, CL7487.Contig1_All, CL7528.Contig1_All, CL7528.Contig2_All, CL7555.Contig1_All, CL7562.Contig1_All, CL7580.Contig1_All, CL7581.Contig1_All, CL766.Contig1_All, CL766.Contig2_All, CL766.Contig3_All, CL766.Contig4_All, CL766.Contig5_All, CL7664.Contig1_All, CL7676.Contig1_All, CL7707.Contig1_All, CL7725.Contig1_All, CL7772.Contig1_All, CL7823.Contig1_All, CL7840.Contig1_All, CL8003.Contig1_All, CL8104.Contig1_All, CL8161.Contig1_All, CL8165.Contig1_All, CL829.Contig1_All, CL829.Contig2_All, CL829.Contig3_All, CL8293.Contig1_All, CL8320.Contig1_All, CL8338.Contig1_All, CL8338.Contig2_All, CL8346.Contig1_All, CL8452.Contig1_All, CL8630.Contig1_All, CL8748.Contig1_All, CL8773.Contig1_All, CL8909.Contig1_All, CL8909.Contig2_All, CL9085.Contig1_All, CL9598.Contig1_All, CL9739.Contig1_All, CL9777.Contig1_All, CL996.Contig1_All, CL996.Contig2_All, CL996.Contig3_All, CL996.Contig4_All, CL996.Contig5_All,

			Unigene1055_All, Unigene1232_All, Unigene1235_All, Unigene1441_All, Unigene145_All, Unigene1481_All, Unigene1686_All, Unigene1738_All, Unigene173_All, Unigene1788_All, Unigene1909_All, Unigene2001_All, Unigene2207_All, Unigene2253_All, Unigene2334_All, Unigene2335_All, Unigene2504_All, Unigene2590_All, Unigene2688_All, Unigene2697_All, Unigene2706_All, Unigene278_All, Unigene2865_All, Unigene2880_All, Unigene2921_All, Unigene3094_All, Unigene3156_All, Unigene316_All, Unigene3185_All, Unigene3202_All, Unigene3468_All, Unigene3648_All, Unigene4000_All, Unigene4176_All, Unigene4180_All, Unigene4401_All, Unigene4671_All, Unigene4775_All, Unigene477_All, Unigene4843_All, Unigene4862_All, Unigene4917_All, Unigene5188_All, Unigene518_All, Unigene5279_All, Unigene5303_All, Unigene5322_All, Unigene5357_All, Unigene579_All, Unigene622_All, Unigene760_All
13			CL1.Contig102_All, CL1.Contig12_All, CL1.Contig57_All, CL10048.Contig1_All, CL10058.Contig1_All, CL10073.Contig1_All, CL10132.Contig1_All, CL1015.Contig2_All, CL10214.Contig1_All, CL10353.Contig1_All, CL10384.Contig1_All, CL10406.Contig1_All, CL10477.Contig1_All, CL1048.Contig1_All, CL10497.Contig1_All, CL10524.Contig1_All, CL10592.Contig1_All, CL106.Contig1_All, CL106.Contig2_All, CL106.Contig3_All, CL106.Contig4_All, CL106.Contig5_All, CL106.Contig6_All, CL10600.Contig1_All, CL10652.Contig1_All, CL10685.Contig1_All, CL10729.Contig1_All, CL10746.Contig1_All, CL1084.Contig1_All, CL1084.Contig2_All, CL1084.Contig3_All, CL1084.Contig4_All, CL1084.Contig5_All, CL10964.Contig1_All, CL11137.Contig1_All, CL11279.Contig1_All, CL11320.Contig1_All, CL11464.Contig1_All, CL11525.Contig1_All, CL11562.Contig1_All, CL11570.Contig1_All, CL1165.Contig1_All, CL1165.Contig2_All, CL1165.Contig3_All, CL1165.Contig4_All, CL11954.Contig1_All, CL12020.Contig1_All, CL1227.Contig1_All, CL12430.Contig1_All, CL12487.Contig1_All, CL12514.Contig1_All, CL12611.Contig1_All, CL12668.Contig1_All, CL12810.Contig1_All, CL13025.Contig1_All, CL13080.Contig1_All, CL13270.Contig1_All, CL13277.Contig1_All, CL1342.Contig1_All, CL1342.Contig2_All, CL1342.Contig3_All, CL1342.Contig4_All, CL1346.Contig1_All, CL1346.Contig2_All, CL1346.Contig3_All, CL1346.Contig4_All, CL13523.Contig1_All, CL13574.Contig1_All, CL13714.Contig1_All, CL13836.Contig1_All, CL1384.Contig1_All, CL13928.Contig1_All, CL13973.Contig1_All, CL13980.Contig1_All, CL14122.Contig1_All, CL14122.Contig2_All, CL14140.Contig1_All,

	Purine metabolism	CL1426.Contig1_All, CL14394.Contig1_All, CL14423.Contig1_All, CL14503.Contig1_All, CL14510.Contig1_All, CL14510.Contig2_All, CL1484.Contig1_All, CL1484.Contig2_All, CL1484.Contig3_All, CL14954.Contig1_All, CL14981.Contig1_All, CL15039.Contig1_All, CL15100.Contig1_All, CL1527.Contig1_All, CL1527.Contig2_All, CL1527.Contig3_All, CL15275.Contig1_All, CL15323.Contig1_All, CL1548.Contig2_All, CL15566.Contig1_All, CL1559.Contig1_All, CL1559.Contig2_All, CL15638.Contig1_All, CL15697.Contig1_All, CL15799.Contig1_All, CL15961.Contig1_All, CL16128.Contig1_All, CL16425.Contig1_All, CL16515.Contig1_All, CL16668.Contig1_All, CL1672.Contig1_All, CL16771.Contig1_All, CL16817.Contig1_All, CL17057.Contig1_All, CL17066.Contig1_All, CL17291.Contig1_All, CL17291.Contig2_All, CL17370.Contig1_All, CL17405.Contig1_All, CL17512.Contig1_All, CL17778.Contig1_All, CL17852.Contig1_All, CL18207.Contig1_All, CL18239.Contig1_All, CL18317.Contig1_All, CL18325.Contig1_All, CL18360.Contig1_All, CL18391.Contig1_All, CL18441.Contig1_All, CL18491.Contig1_All, CL18538.Contig1_All, CL1863.Contig2_All, CL1863.Contig3_All, CL19082.Contig1_All, CL19200.Contig1_All, CL19398.Contig1_All, CL19445.Contig1_All, CL19651.Contig1_All, CL19667.Contig1_All, CL19812.Contig1_All, CL20405.Contig1_All, CL20484.Contig1_All, CL20773.Contig1_All, CL20934.Contig1_All, CL21003.Contig1_All, CL2108.Contig1_All, CL2108.Contig2_All, CL21240.Contig1_All, CL2129.Contig2_All, CL213.Contig1_All, CL21356.Contig1_All, CL21432.Contig1_All, CL21473.Contig1_All, CL21662.Contig1_All, CL2179.Contig1_All, CL2181.Contig1_All, CL21845.Contig1_All, CL21945.Contig1_All, CL22044.Contig1_All, CL22352.Contig1_All, CL22823.Contig1_All, CL2289.Contig3_All, CL22913.Contig1_All, CL23087.Contig1_All, CL23155.Contig1_All, CL23227.Contig1_All, CL23312.Contig1_All, CL23363.Contig1_All, CL23445.Contig1_All, CL23570.Contig1_All, CL23629.Contig1_All, CL23834.Contig1_All, CL23911.Contig1_All, CL23928.Contig1_All, CL24063.Contig1_All, CL24100.Contig1_All, CL24469.Contig1_All, CL24517.Contig1_All, CL24536.Contig1_All, CL24615.Contig1_All, CL24617.Contig1_All, CL24635.Contig1_All, CL24709.Contig1_All, CL24714.Contig1_All, CL24786.Contig1_All, CL24839.Contig1_All, CL2498.Contig1_All, CL25009.Contig1_All,

			CL25044.Contig1_All, CL25107.Contig1_All, CL25340.Contig1_All, CL2546.Contig1_All, CL25515.Contig1_All, CL25712.Contig1_All, CL25785.Contig1_All, CL25836.Contig1_All, CL26190.Contig1_All, CL26303.Contig1_All, CL26380.Contig1_All, CL264.Contig8_All, CL26534.Contig1_All, CL2679.Contig1_All, CL26933.Contig1_All, CL27025.Contig1_All, CL27026.Contig1_All, CL27071.Contig1_All, CL27120.Contig1_All, CL27261.Contig1_All, CL27307.Contig1_All, CL275.Contig1_All, CL275.Contig2_All, CL2761.Contig1_All, CL2775.Contig1_All, CL27833.Contig1_All, CL27834.Contig1_All, CL27884.Contig1_All, CL27887.Contig1_All, CL27901.Contig1_All, CL27926.Contig1_All, CL2839.Contig1_All, CL2874.Contig1_All, CL2874.Contig2_All, CL2942.Contig2_All, CL2942.Contig3_All, CL3027.Contig1_All, CL3134.Contig1_All, CL3152.Contig1_All, CL3243.Contig1_All, CL3243.Contig2_All, CL3367.Contig1_All, CL3367.Contig2_All, CL3376.Contig1_All, CL3401.Contig1_All, CL3434.Contig1_All, CL3452.Contig1_All, CL3452.Contig2_All, CL351.Contig1_All, CL351.Contig5_All, CL36.Contig13_All, CL36.Contig15_All, CL36.Contig16_All, CL36.Contig17_All, CL36.Contig3_All, CL36.Contig9_All, CL3645.Contig1_All, CL3740.Contig1_All, CL3740.Contig2_All, CL4030.Contig1_All, CL4161.Contig1_All, CL4205.Contig1_All, CL4359.Contig1_All, CL436.Contig1_All, CL4363.Contig1_All, CL4363.Contig3_All, CL4415.Contig1_All, CL4453.Contig1_All, CL4477.Contig1_All, CL4477.Contig2_All, CL4593.Contig1_All, CL4616.Contig1_All, CL4724.Contig1_All, CL4793.Contig1_All, CL4845.Contig1_All, CL4845.Contig2_All, CL4873.Contig1_All, CL488.Contig1_All, CL488.Contig2_All, CL488.Contig3_All, CL488.Contig4_All, CL4900.Contig1_All, CL4903.Contig1_All, CL4951.Contig1_All, CL4975.Contig1_All, CL498.Contig1_All, CL5243.Contig1_All, CL5333.Contig1_All, CL5395.Contig1_All, CL545.Contig1_All, CL545.Contig2_All, CL545.Contig3_All, CL5500.Contig1_All, CL5500.Contig2_All, CL5569.Contig1_All, CL5607.Contig2_All, CL5618.Contig1_All, CL5618.Contig2_All, CL577.Contig1_All, CL577.Contig2_All, CL577.Contig3_All, CL5854.Contig1_All, CL6042.Contig1_All, CL6053.Contig1_All, CL612.Contig1_All, CL6172.Contig1_All, CL6299.Contig1_All, CL6388.Contig1_All, CL6424.Contig1_All, CL6475.Contig1_All, CL6515.Contig1_All, CL6557.Contig1_All, CL6557.Contig2_All, CL656.Contig1_All, CL656.Contig2_All, CL656.Contig3_All, CL656.Contig4_All, CL6652.Contig1_All, CL6722.Contig1_All, CL6763.Contig1_All, CL6805.Contig1_All, CL6861.Contig1_All, CL6917.Contig1_All, CL697.Contig1_All,

			CL699.Contig1_All, CL6991.Contig1_All, CL7090.Contig1_All, CL7112.Contig1_All, CL7167.Contig1_All, CL7279.Contig1_All, CL7364.Contig1_All, CL7412.Contig2_All, CL7593.Contig1_All, CL77.Contig10_All, CL77.Contig1_All, CL77.Contig2_All, CL77.Contig5_All, CL77.Contig7_All, CL77.Contig8_All, CL77.Contig9_All, CL771.Contig1_All, CL7747.Contig1_All, CL7880.Contig1_All, CL7904.Contig1_All, CL7923.Contig1_All, CL7923.Contig2_All, CL7965.Contig1_All, CL8030.Contig1_All, CL8081.Contig1_All, CL8159.Contig1_All, CL8213.Contig1_All, CL8358.Contig1_All, CL8437.Contig1_All, CL8450.Contig1_All, CL8487.Contig1_All, CL8511.Contig1_All, CL8528.Contig1_All, CL8545.Contig1_All, CL8564.Contig1_All, CL8588.Contig1_All, CL8639.Contig1_All, CL8639.Contig2_All, CL8644.Contig1_All, CL8668.Contig1_All, CL8738.Contig1_All, CL9093.Contig1_All, CL9266.Contig1_All, CL928.Contig1_All, CL928.Contig2_All, CL928.Contig3_All, CL9303.Contig1_All, CL9570.Contig1_All, CL9680.Contig1_All, CL9797.Contig1_All, CL9799.Contig1_All, CL9987.Contig1_All, Unigene1109_All, Unigene1335_All, Unigene1386_All, Unigene1586_All, Unigene1597_All, Unigene1707_All, Unigene1792_All, Unigene1935_All, Unigene2133_All, Unigene214_All, Unigene2195_All, Unigene229_All, Unigene234_All, Unigene2360_All, Unigene2408_All, Unigene2436_All, Unigene2509_All, Unigene2511_All, Unigene2550_All, Unigene2570_All, Unigene2613_All, Unigene2637_All, Unigene2717_All, Unigene2839_All, Unigene2876_All, Unigene2881_All, Unigene2974_All, Unigene3151_All, Unigene3236_All, Unigene3255_All, Unigene3258_All, Unigene3275_All, Unigene3522_All, Unigene3654_All, Unigene3657_All, Unigene3744_All, Unigene3789_All, Unigene4017_All, Unigene4038_All, Unigene4051_All, Unigene4058_All, Unigene4065_All, Unigene408_All, Unigene4211_All, Unigene4277_All, Unigene4293_All, Unigene4444_All, Unigene4613_All, Unigene4808_All, Unigene4864_All, Unigene5261_All, Unigene5332_All, Unigene5511_All, Unigene606_All, Unigene652_All, Unigene734_All, Unigene74_All, Unigene75_All, Unigene854_All, Unigene87_All
14			CL10066.Contig1_All, CL10080.Contig1_All, CL10103.Contig1_All, CL10229.Contig1_All, CL10249.Contig1_All, CL10285.Contig1_All, CL104.Contig1_All, CL104.Contig2_All, CL104.Contig3_All, CL104.Contig4_All, CL104.Contig5_All, CL104.Contig6_All, CL104.Contig7_All, CL10549.Contig1_All, CL1058.Contig1_All, CL1058.Contig2_All, CL1058.Contig3_All, CL1068.Contig1_All, CL10778.Contig1_All, CL10916.Contig1_All, CL10954.Contig1_All, CL10976.Contig1_All, CL1098.Contig1_All, CL1098.Contig2_All,

	mRNA surveillance pathway	CL1098.Contig3_All, CL1098.Contig4_All, CL11.Contig12_All, CL11055.Contig1_All, CL11055.Contig2_All, CL11240.Contig1_All, CL1147.Contig2_All, CL11495.Contig1_All, CL115.Contig2_All, CL11544.Contig1_All, CL1157.Contig1_All, CL11636.Contig1_All, CL11644.Contig1_All, CL1178.Contig1_All, CL1178.Contig2_All, CL1178.Contig3_All, CL11825.Contig1_All, CL11905.Contig1_All, CL11987.Contig1_All, CL1199.Contig1_All, CL1199.Contig2_All, CL11998.Contig1_All, CL1204.Contig1_All, CL1204.Contig2_All, CL1204.Contig3_All, CL12118.Contig1_All, CL12371.Contig1_All, CL12425.Contig1_All, CL12595.Contig1_All, CL12630.Contig1_All, CL12786.Contig1_All, CL12807.Contig1_All, CL1295.Contig1_All, CL13131.Contig1_All, CL13159.Contig1_All, CL13171.Contig1_All, CL13248.Contig2_All, CL13361.Contig1_All, CL13624.Contig1_All, CL1364.Contig1_All, CL1364.Contig2_All, CL13697.Contig1_All, CL14088.Contig1_All, CL14100.Contig1_All, CL14292.Contig1_All, CL144.Contig4_All, CL144.Contig6_All, CL144.Contig7_All, CL144.Contig8_All, CL14472.Contig1_All, CL14771.Contig1_All, CL14830.Contig1_All, CL14870.Contig1_All, CL14948.Contig1_All, CL15042.Contig1_All, CL15201.Contig1_All, CL15346.Contig1_All, CL15371.Contig1_All, CL15388.Contig1_All, CL15454.Contig1_All, CL1572.Contig1_All, CL1572.Contig2_All, CL15744.Contig1_All, CL15775.Contig1_All, CL15786.Contig1_All, CL15824.Contig1_All, CL15997.Contig1_All, CL1613.Contig3_All, CL16229.Contig1_All, CL1652.Contig1_All, CL16533.Contig1_All, CL16548.Contig1_All, CL16551.Contig1_All, CL16639.Contig1_All, CL1678.Contig3_All, CL16887.Contig1_All, CL16920.Contig1_All, CL1700.Contig1_All, CL17067.Contig1_All, CL17343.Contig1_All, CL17422.Contig1_All, CL17430.Contig1_All, CL17452.Contig1_All, CL1767.Contig1_All, CL1767.Contig2_All, CL17792.Contig1_All, CL1819.Contig1_All, CL1828.Contig1_All, CL18308.Contig1_All, CL18388.Contig1_All, CL1846.Contig1_All, CL1846.Contig3_All, CL1846.Contig4_All, CL1846.Contig5_All, CL1846.Contig6_All, CL18514.Contig1_All, CL18531.Contig1_All, CL1870.Contig2_All, CL18772.Contig1_All, CL19094.Contig1_All, CL19427.Contig1_All, CL19478.Contig1_All, CL19665.Contig1_All, CL19684.Contig1_All, CL19714.Contig1_All, CL19715.Contig1_All, CL19748.Contig1_All, CL1996.Contig1_All, CL1996.Contig2_All, CL1996.Contig3_All,

			CL1996.Contig4_All, CL2.Contig39_All, CL2002.Contig1_All, CL2010.Contig1_All, CL202.Contig1_All, CL202.Contig2_All, CL202.Contig3_All, CL202.Contig4_All, CL202.Contig5_All, CL20382.Contig1_All, CL20451.Contig1_All, CL20493.Contig1_All, CL20539.Contig1_All, CL20647.Contig1_All, CL20649.Contig1_All, CL20662.Contig1_All, CL20676.Contig1_All, CL20687.Contig1_All, CL20708.Contig1_All, CL20814.Contig1_All, CL20971.Contig1_All, CL2103.Contig1_All, CL2103.Contig2_All, CL2103.Contig3_All, CL21333.Contig1_All, CL21418.Contig1_All, CL21549.Contig1_All, CL21703.Contig1_All, CL21752.Contig1_All, CL21890.Contig1_All, CL22123.Contig1_All, CL22270.Contig1_All, CL2268.Contig1_All, CL2268.Contig2_All, CL22736.Contig1_All, CL2291.Contig1_All, CL23090.Contig1_All, CL2311.Contig1_All, CL2311.Contig2_All, CL23158.Contig1_All, CL23242.Contig1_All, CL23250.Contig1_All, CL23359.Contig1_All, CL23662.Contig1_All, CL23747.Contig1_All, CL24047.Contig1_All, CL2406.Contig1_All, CL2412.Contig1_All, CL2412.Contig2_All, CL24281.Contig1_All, CL243.Contig2_All, CL243.Contig3_All, CL243.Contig4_All, CL243.Contig5_All, CL2440.Contig1_All, CL24497.Contig1_All, CL24879.Contig1_All, CL24898.Contig1_All, CL24924.Contig1_All, CL2521.Contig1_All, CL26000.Contig1_All, CL2601.Contig1_All, CL2606.Contig1_All, CL26405.Contig1_All, CL26408.Contig1_All, CL26468.Contig1_All, CL2647.Contig1_All, CL26539.Contig1_All, CL26578.Contig1_All, CL26587.Contig1_All, CL26710.Contig1_All, CL26798.Contig1_All, CL26810.Contig1_All, CL26964.Contig1_All, CL27178.Contig1_All, CL27345.Contig1_All, CL27443.Contig1_All, CL27517.Contig1_All, CL27639.Contig1_All, CL2780.Contig1_All, CL27802.Contig1_All, CL27913.Contig1_All, CL28114.Contig1_All, CL2877.Contig1_All, CL2901.Contig1_All, CL2951.Contig1_All, CL2951.Contig2_All, CL2960.Contig1_All, CL298.Contig1_All, CL298.Contig2_All, CL2984.Contig2_All, CL3038.Contig1_All, CL3038.Contig2_All, CL306.Contig1_All, CL306.Contig2_All, CL306.Contig3_All, CL3082.Contig1_All, CL3119.Contig1_All, CL3253.Contig1_All, CL3253.Contig2_All, CL3389.Contig1_All, CL3403.Contig1_All, CL351.Contig2_All, CL351.Contig7_All, CL3551.Contig1_All, CL3552.Contig1_All, CL3578.Contig1_All, CL3578.Contig2_All, CL3578.Contig3_All, CL3693.Contig1_All, CL3693.Contig2_All, CL3817.Contig1_All, CL3817.Contig2_All, CL3817.Contig3_All, CL3917.Contig1_All, CL396.Contig1_All, CL396.Contig2_All, CL396.Contig3_All, CL396.Contig4_All, CL4142.Contig1_All,

		CL4147.Contig1_All, CL4228.Contig1_All, CL4228.Contig2_All, CL4228.Contig3_All, CL4233.Contig1_All, CL4287.Contig1_All, CL4290.Contig1_All, CL4297.Contig1_All, CL4601.Contig1_All, CL4715.Contig1_All, CL4738.Contig1_All, CL4889.Contig1_All, CL496.Contig2_All, CL5035.Contig1_All, CL513.Contig1_All, CL5131.Contig1_All, CL5242.Contig1_All, CL5299.Contig1_All, CL5305.Contig1_All, CL5313.Contig1_All, CL5438.Contig1_All, CL5451.Contig1_All, CL5454.Contig1_All, CL5530.Contig1_All, CL5530.Contig2_All, CL5613.Contig1_All, CL570.Contig1_All, CL5740.Contig1_All, CL5887.Contig1_All, CL5944.Contig1_All, CL6070.Contig1_All, CL6255.Contig1_All, CL6319.Contig1_All, CL6397.Contig1_All, CL6505.Contig1_All, CL6517.Contig1_All, CL654.Contig2_All, CL654.Contig3_All, CL654.Contig4_All, CL664.Contig1_All, CL665.Contig1_All, CL665.Contig2_All, CL665.Contig3_All, CL667.Contig1_All, CL667.Contig2_All, CL667.Contig3_All, CL667.Contig4_All, CL667.Contig5_All, CL6736.Contig1_All, CL6829.Contig1_All, CL7067.Contig1_All, CL7081.Contig1_All, CL721.Contig1_All, CL721.Contig2_All, CL721.Contig3_All, CL721.Contig4_All, CL721.Contig5_All, CL7226.Contig1_All, CL7237.Contig1_All, CL7264.Contig1_All, CL7455.Contig1_All, CL7505.Contig1_All, CL7525.Contig1_All, CL7666.Contig1_All, CL7690.Contig1_All, CL7748.Contig1_All, CL7871.Contig1_All, CL791.Contig3_All, CL791.Contig4_All, CL7949.Contig1_All, CL7972.Contig1_All, CL7986.Contig1_All, CL8147.Contig1_All, CL8160.Contig1_All, CL824.Contig1_All, CL824.Contig2_All, CL8313.Contig1_All, CL8329.Contig1_All, CL8337.Contig1_All, CL835.Contig1_All, CL835.Contig2_All, CL8399.Contig1_All, CL8428.Contig1_All, CL8537.Contig1_All, CL883.Contig1_All, CL8844.Contig1_All, CL901.Contig1_All, CL901.Contig2_All, CL901.Contig3_All, CL901.Contig4_All, CL9313.Contig1_All, CL9621.Contig1_All, CL9801.Contig1_All, CL9808.Contig1_All, CL9808.Contig2_All, CL9895.Contig1_All, Unigene1058_All, Unigene1068_All, Unigene1092_All, Unigene1094_All, Unigene1100_All, Unigene1150_All, Unigene1208_All, Unigene1279_All, Unigene1407_All, Unigene1426_All, Unigene1597_All, Unigene159_All, Unigene1607_All, Unigene1609_All, Unigene1727_All, Unigene1733_All, Unigene1824_All, Unigene1825_All, Unigene192_All, Unigene1997_All, Unigene202_All, Unigene2147_All, Unigene2404_All, Unigene2417_All, Unigene2658_All, Unigene2968_All, Unigene3036_All, Unigene3072_All, Unigene3073_All, Unigene3249_All, Unigene3307_All, Unigene3362_All, Unigene3664_All, Unigene3842_All, Unigene4244_All, Unigene4434_All, Unigene4441_All, Unigene4688_All, Unigene5067_All, Unigene5102_All, Unigene528_All, Unigene600_All, Unigene668_All, Unigene865_All
15		

	Ubiquitin mediated	CL10070.Contig1_All, CL10119.Contig1_All, CL10177.Contig1_All, CL10219.Contig1_All, CL10276.Contig1_All, CL10301.Contig1_All, CL10404.Contig1_All, CL10422.Contig1_All, CL1083.Contig1_All, CL1083.Contig2_All, CL10907.Contig1_All, CL1107.Contig1_All, CL11175.Contig1_All, CL11258.Contig1_All, CL11289.Contig1_All, CL11297.Contig1_All, CL11307.Contig1_All, CL11347.Contig1_All, CL11436.Contig1_All, CL115.Contig3_All, CL11680.Contig1_All, CL1169.Contig2_All, CL11723.Contig1_All, CL12009.Contig1_All, CL1226.Contig1_All, CL12291.Contig1_All, CL1230.Contig1_All, CL12311.Contig1_All, CL12318.Contig1_All, CL12360.Contig1_All, CL12431.Contig1_All, CL1245.Contig1_All, CL12488.Contig1_All, CL1251.Contig1_All, CL1251.Contig2_All, CL1251.Contig3_All, CL1259.Contig1_All, CL12641.Contig1_All, CL12936.Contig1_All, CL1296.Contig1_All, CL1296.Contig2_All, CL1296.Contig3_All, CL1296.Contig4_All, CL1296.Contig5_All, CL13093.Contig1_All, CL13173.Contig1_All, CL1321.Contig1_All, CL1321.Contig2_All, CL1321.Contig3_All, CL1321.Contig4_All, CL13287.Contig1_All, CL13306.Contig1_All, CL13340.Contig1_All, CL13425.Contig1_All, CL1373.Contig1_All, CL13791.Contig1_All, CL1411.Contig1_All, CL14120.Contig1_All, CL14160.Contig1_All, CL14196.Contig1_All, CL1438.Contig1_All, CL1439.Contig1_All, CL1459.Contig1_All, CL14767.Contig1_All, CL1502.Contig2_All, CL15034.Contig1_All, CL15037.Contig1_All, CL15049.Contig2_All, CL15086.Contig1_All, CL15482.Contig1_All, CL15549.Contig1_All, CL15630.Contig1_All, CL15794.Contig1_All, CL15797.Contig1_All, CL15838.Contig1_All, CL16223.Contig1_All, CL16223.Contig2_All, CL16393.Contig1_All, CL16429.Contig1_All, CL16467.Contig1_All, CL1653.Contig1_All, CL1653.Contig2_All, CL1653.Contig3_All, CL1653.Contig4_All, CL16574.Contig1_All, CL16796.Contig1_All, CL168.Contig1_All, CL16807.Contig1_All, CL16965.Contig1_All, CL17.Contig15_All, CL17.Contig16_All, CL17.Contig30_All, CL17.Contig40_All, CL17.Contig41_All, CL17.Contig45_All, CL17082.Contig1_All, CL17252.Contig1_All, CL1726.Contig1_All, CL17287.Contig1_All, CL1737.Contig1_All, CL1737.Contig2_All, CL1737.Contig3_All, CL17443.Contig1_All, CL18222.Contig1_All, CL1842.Contig1_All, CL18460.Contig1_All, CL18488.Contig1_All, CL18929.Contig1_All, CL1898.Contig1_All, CL19416.Contig1_All,

	[proteolysis]()	CL19497.Contig1_All, CL1960.Contig1_All, CL19603.Contig1_All, CL19797.Contig1_All, CL19825.Contig1_All, CL19851.Contig1_All, CL19887.Contig1_All, CL19920.Contig1_All, CL19938.Contig1_All, CL19979.Contig1_All, CL2.Contig45_All, CL20127.Contig1_All, CL2016.Contig1_All, CL2029.Contig1_All, CL20330.Contig1_All, CL20625.Contig1_All, CL20768.Contig1_All, CL20891.Contig1_All, CL20910.Contig1_All, CL20995.Contig1_All, CL21206.Contig1_All, CL21348.Contig1_All, CL21483.Contig1_All, CL21607.Contig1_All, CL21840.Contig1_All, CL21894.Contig1_All, CL22110.Contig1_All, CL22202.Contig1_All, CL2229.Contig1_All, CL2229.Contig2_All, CL2229.Contig3_All, CL22332.Contig1_All, CL22456.Contig1_All, CL22704.Contig1_All, CL2271.Contig1_All, CL2271.Contig2_All, CL2271.Contig3_All, CL22764.Contig1_All, CL22838.Contig1_All, CL22847.Contig1_All, CL2295.Contig1_All, CL2309.Contig1_All, CL2309.Contig2_All, CL23163.Contig1_All, CL23696.Contig1_All, CL23757.Contig1_All, CL23792.Contig1_All, CL2405.Contig1_All, CL2409.Contig1_All, CL2432.Contig1_All, CL2466.Contig1_All, CL24914.Contig1_All, CL25106.Contig1_All, CL25204.Contig1_All, CL25614.Contig1_All, CL2584.Contig1_All, CL25897.Contig1_All, CL2608.Contig1_All, CL2608.Contig2_All, CL2608.Contig3_All, CL26153.Contig1_All, CL26158.Contig1_All, CL2628.Contig1_All, CL26415.Contig1_All, CL26846.Contig1_All, CL27007.Contig1_All, CL2707.Contig1_All, CL27202.Contig1_All, CL27615.Contig1_All, CL27777.Contig1_All, CL2788.Contig1_All, CL2809.Contig1_All, CL28109.Contig1_All, CL2813.Contig1_All, CL282.Contig2_All, CL2833.Contig1_All, CL2886.Contig1_All, CL297.Contig1_All, CL3075.Contig1_All, CL3075.Contig3_All, CL313.Contig1_All, CL313.Contig2_All, CL313.Contig3_All, CL316.Contig1_All, CL316.Contig2_All, CL316.Contig3_All, CL316.Contig4_All, CL3190.Contig1_All, CL3313.Contig1_All, CL3360.Contig1_All, CL3360.Contig2_All, CL3360.Contig3_All, CL3407.Contig1_All, CL3414.Contig1_All, CL344.Contig1_All, CL344.Contig2_All, CL344.Contig3_All, CL344.Contig4_All, CL344.Contig5_All, CL344.Contig6_All, CL344.Contig7_All, CL344.Contig8_All, CL344.Contig9_All, CL3447.Contig1_All, CL3493.Contig1_All, CL350.Contig1_All, CL3585.Contig1_All, CL3585.Contig2_All, CL3599.Contig1_All, CL3599.Contig2_All, CL3624.Contig1_All, CL3700.Contig1_All, CL3760.Contig1_All, CL3796.Contig1_All, CL3928.Contig1_All, CL3951.Contig1_All, CL3951.Contig2_All, CL4053.Contig1_All, CL4070.Contig1_All,

		CL4169.Contig1_All, CL417.Contig1_All, CL417.Contig3_All, CL417.Contig4_All, CL417.Contig6_All, CL417.Contig7_All, CL4176.Contig1_All, CL4208.Contig1_All, CL4349.Contig1_All, CL4375.Contig1_All, CL4375.Contig2_All, CL4396.Contig1_All, CL4439.Contig1_All, CL4470.Contig2_All, CL4474.Contig1_All, CL4612.Contig1_All, CL4639.Contig2_All, CL4660.Contig1_All, CL4707.Contig2_All, CL4714.Contig1_All, CL4714.Contig2_All, CL4757.Contig1_All, CL48.Contig10_All, CL48.Contig4_All, CL48.Contig5_All, CL48.Contig8_All, CL48.Contig9_All, CL485.Contig1_All, CL5001.Contig1_All, CL5087.Contig1_All, CL5164.Contig1_All, CL5164.Contig2_All, CL5212.Contig1_All, CL5322.Contig1_All, CL5322.Contig2_All, CL5331.Contig1_All, CL5349.Contig1_All, CL549.Contig1_All, CL549.Contig2_All, CL549.Contig3_All, CL549.Contig4_All, CL5532.Contig1_All, CL5667.Contig1_All, CL5667.Contig2_All, CL5667.Contig3_All, CL568.Contig1_All, CL573.Contig1_All, CL573.Contig2_All, CL573.Contig3_All, CL5911.Contig1_All, CL6100.Contig1_All, CL616.Contig2_All, CL616.Contig3_All, CL616.Contig4_All, CL6163.Contig1_All, CL6163.Contig2_All, CL632.Contig1_All, CL632.Contig2_All, CL632.Contig3_All, CL6610.Contig1_All, CL6658.Contig1_All, CL668.Contig1_All, CL693.Contig2_All, CL6986.Contig1_All, CL7074.Contig1_All, CL7082.Contig1_All, CL7256.Contig1_All, CL7319.Contig1_All, CL7471.Contig1_All, CL7552.Contig1_All, CL7558.Contig1_All, CL7629.Contig1_All, CL7756.Contig1_All, CL780.Contig1_All, CL780.Contig2_All, CL780.Contig3_All, CL7883.Contig1_All, CL7968.Contig1_All, CL8051.Contig1_All, CL8055.Contig1_All, CL8315.Contig1_All, CL8315.Contig2_All, CL8344.Contig1_All, CL8412.Contig1_All, CL844.Contig1_All, CL844.Contig2_All, CL844.Contig3_All, CL861.Contig5_All, CL867.Contig1_All, CL874.Contig1_All, CL874.Contig2_All, CL875.Contig1_All, CL8751.Contig1_All, CL8766.Contig1_All, CL8768.Contig1_All, CL8793.Contig1_All, CL9181.Contig1_All, CL9209.Contig1_All, CL931.Contig1_All, CL94.Contig4_All, CL94.Contig7_All, CL9475.Contig1_All, CL956.Contig1_All, CL963.Contig1_All, CL9709.Contig1_All, CL972.Contig1_All, CL9897.Contig1_All, Unigene1011_All, Unigene1048_All, Unigene107_All, Unigene1117_All, Unigene1154_All, Unigene1249_All, Unigene1284_All, Unigene1637_All, Unigene1847_All, Unigene1897_All, Unigene2254_All, Unigene2294_All, Unigene2295_All, Unigene2448_All, Unigene2488_All, Unigene24_All, Unigene2586_All, Unigene2659_All, Unigene2770_All, Unigene2969_All, Unigene3148_All, Unigene348_All, Unigene3650_All, Unigene3673_All, Unigene3783_All, Unigene3900_All, Unigene3906_All, Unigene4099_All, Unigene4100_All, Unigene4101_All, Unigene4597_All, Unigene4610_All, Unigene4630_All, Unigene4636_All, Unigene4682_All, Unigene4683_All, Unigene4739_All,

			Unigene4898_All, Unigene5069_All, Unigene5201_All, Unigene529_All, Unigene5407_All, Unigene994_All
16			CL1.Contig120_All, CL1.Contig123_All, CL1.Contig130_All, CL1.Contig23_All, CL1.Contig45_All, CL1.Contig46_All, CL1.Contig57_All, CL1.Contig59_All, CL1.Contig63_All, CL1.Contig91_All, CL1.Contig94_All, CL10406.Contig1_All, CL10416.Contig1_All, CL10416.Contig2_All, CL10416.Contig3_All, CL10417.Contig1_All, CL10483.Contig1_All, CL10535.Contig1_All, CL10577.Contig1_All, CL10621.Contig1_All, CL10624.Contig1_All, CL10629.Contig1_All, CL10678.Contig1_All, CL10686.Contig1_All, CL1069.Contig1_All, CL10716.Contig1_All, CL1074.Contig1_All, CL1074.Contig2_All, CL10932.Contig1_All, CL11041.Contig1_All, CL11094.Contig1_All, CL11145.Contig1_All, CL11160.Contig1_All, CL11236.Contig1_All, CL11261.Contig1_All, CL11423.Contig1_All, CL11477.Contig1_All, CL11520.Contig1_All, CL11616.Contig1_All, CL11672.Contig1_All, CL1173.Contig1_All, CL1175.Contig1_All, CL11980.Contig1_All, CL1207.Contig1_All, CL1207.Contig2_All, CL1207.Contig3_All, CL1207.Contig4_All, CL12095.Contig1_All, CL12715.Contig1_All, CL13169.Contig1_All, CL1328.Contig1_All, CL13297.Contig2_All, CL13357.Contig1_All, CL13666.Contig1_All, CL13717.Contig1_All, CL13717.Contig2_All, CL1378.Contig1_All, CL1378.Contig2_All, CL13818.Contig1_All, CL13877.Contig1_All, CL13975.Contig1_All, CL13975.Contig2_All, CL14003.Contig1_All, CL1406.Contig2_All, CL1406.Contig3_All, CL14112.Contig1_All, CL14310.Contig1_All, CL14313.Contig1_All, CL14326.Contig1_All, CL14365.Contig1_All, CL14392.Contig1_All, CL14416.Contig1_All, CL14487.Contig1_All, CL14558.Contig1_All, CL14732.Contig1_All, CL14738.Contig1_All, CL14752.Contig1_All, CL14764.Contig1_All, CL14877.Contig1_All, CL14966.Contig1_All, CL151.Contig2_All, CL151.Contig4_All, CL15160.Contig1_All, CL15163.Contig1_All, CL15207.Contig1_All, CL15251.Contig1_All, CL15301.Contig1_All, CL15341.Contig1_All, CL15591.Contig1_All, CL15643.Contig1_All, CL15700.Contig1_All, CL15700.Contig2_All, CL15822.Contig1_All, CL15849.Contig1_All, CL16059.Contig1_All, CL1609.Contig1_All, CL1609.Contig4_All, CL16330.Contig1_All, CL16345.Contig1_All, CL16706.Contig1_All, CL1675.Contig1_All, CL1679.Contig1_All, CL171.Contig6_All, CL1711.Contig2_All, CL17263.Contig1_All, CL17278.Contig1_All, CL17278.Contig2_All, CL17326.Contig1_All, CL17382.Contig1_All, CL17426.Contig1_All,

	Ether lipid metabolism	CL18194.Contig1_All, CL1830.Contig1_All, CL18369.Contig1_All, CL18429.Contig1_All, CL1858.Contig1_All, CL18751.Contig1_All, CL18800.Contig1_All, CL18816.Contig1_All, CL19337.Contig1_All, CL19434.Contig1_All, CL19670.Contig1_All, CL19701.Contig1_All, CL19848.Contig1_All, CL1999.Contig1_All, CL1999.Contig2_All, CL20376.Contig1_All, CL20397.Contig1_All, CL20578.Contig1_All, CL20608.Contig1_All, CL20826.Contig1_All, CL209.Contig4_All, CL21089.Contig1_All, CL21093.Contig1_All, CL21148.Contig1_All, CL21227.Contig1_All, CL21321.Contig1_All, CL21335.Contig1_All, CL21393.Contig1_All, CL21451.Contig1_All, CL21562.Contig1_All, CL2160.Contig1_All, CL2181.Contig1_All, CL2181.Contig2_All, CL2181.Contig3_All, CL2192.Contig1_All, CL2192.Contig3_All, CL21955.Contig1_All, CL22085.Contig1_All, CL22136.Contig1_All, CL22152.Contig1_All, CL22314.Contig1_All, CL22377.Contig1_All, CL22461.Contig1_All, CL22977.Contig1_All, CL23358.Contig1_All, CL2353.Contig1_All, CL2353.Contig4_All, CL2355.Contig1_All, CL23588.Contig1_All, CL2387.Contig1_All, CL23965.Contig1_All, CL24251.Contig1_All, CL24281.Contig1_All, CL24479.Contig1_All, CL24831.Contig1_All, CL25143.Contig1_All, CL2515.Contig1_All, CL25220.Contig1_All, CL25363.Contig1_All, CL25376.Contig1_All, CL25710.Contig1_All, CL2579.Contig1_All, CL26309.Contig1_All, CL26483.Contig1_All, CL26501.Contig1_All, CL26645.Contig1_All, CL26978.Contig1_All, CL27010.Contig1_All, CL27661.Contig1_All, CL27703.Contig1_All, CL2860.Contig3_All, CL288.Contig3_All, CL288.Contig8_All, CL2961.Contig4_All, CL3103.Contig1_All, CL3186.Contig1_All, CL3285.Contig1_All, CL3285.Contig2_All, CL3308.Contig2_All, CL331.Contig2_All, CL331.Contig4_All, CL35.Contig11_All, CL35.Contig16_All, CL35.Contig1_All, CL35.Contig22_All, CL35.Contig4_All, CL35.Contig6_All, CL35.Contig8_All, CL351.Contig5_All, CL3646.Contig1_All, CL3646.Contig2_All, CL374.Contig5_All, CL3840.Contig1_All, CL385.Contig1_All, CL385.Contig2_All, CL385.Contig3_All, CL388.Contig1_All, CL388.Contig2_All, CL388.Contig3_All, CL388.Contig4_All, CL388.Contig5_All, CL3932.Contig1_All, CL3938.Contig1_All, CL3938.Contig2_All, CL3938.Contig3_All, CL3957.Contig1_All, CL3957.Contig2_All, CL4062.Contig1_All, CL4062.Contig2_All, CL4077.Contig1_All, CL4190.Contig1_All, CL4190.Contig2_All, CL4295.Contig1_All, CL4295.Contig2_All, CL4362.Contig1_All, CL4685.Contig1_All, CL4723.Contig2_All,

			CL4892.Contig1_All, CL4892.Contig2_All, CL4946.Contig2_All, CL4946.Contig3_All, CL5036.Contig1_All, CL5068.Contig1_All, CL518.Contig1_All, CL518.Contig2_All, CL518.Contig3_All, CL518.Contig4_All, CL518.Contig5_All, CL5317.Contig1_All, CL5321.Contig1_All, CL535.Contig1_All, CL5359.Contig1_All, CL537.Contig2_All, CL537.Contig5_All, CL5401.Contig1_All, CL5475.Contig1_All, CL55.Contig2_All, CL55.Contig4_All, CL5870.Contig1_All, CL589.Contig1_All, CL589.Contig2_All, CL589.Contig3_All, CL6.Contig1_All, CL6.Contig25_All, CL6002.Contig1_All, CL6002.Contig2_All, CL6009.Contig1_All, CL6009.Contig2_All, CL606.Contig1_All, CL6096.Contig1_All, CL6155.Contig1_All, CL625.Contig1_All, CL6263.Contig1_All, CL6263.Contig2_All, CL6388.Contig1_All, CL6506.Contig1_All, CL6538.Contig1_All, CL6581.Contig1_All, CL6617.Contig1_All, CL6617.Contig2_All, CL6696.Contig1_All, CL6913.Contig1_All, CL7.Contig28_All, CL70.Contig8_All, CL7030.Contig1_All, CL7181.Contig1_All, CL7240.Contig1_All, CL7240.Contig2_All, CL7276.Contig1_All, CL7276.Contig2_All, CL7597.Contig2_All, CL7615.Contig2_All, CL7941.Contig1_All, CL7959.Contig1_All, CL7961.Contig1_All, CL8159.Contig1_All, CL820.Contig1_All, CL820.Contig2_All, CL8225.Contig1_All, CL8299.Contig1_All, CL8375.Contig1_All, CL8439.Contig1_All, CL8562.Contig1_All, CL8608.Contig1_All, CL8608.Contig2_All, CL864.Contig1_All, CL864.Contig3_All, CL8643.Contig1_All, CL8643.Contig2_All, CL878.Contig1_All, CL8814.Contig1_All, CL8918.Contig1_All, CL9190.Contig1_All, CL9408.Contig1_All, CL946.Contig1_All, CL946.Contig2_All, CL946.Contig3_All, CL946.Contig4_All, CL9508.Contig1_All, CL9568.Contig1_All, CL9724.Contig1_All, Unigene1043_All, Unigene1053_All, Unigene1153_All, Unigene1207_All, Unigene1262_All, Unigene1274_All, Unigene1294_All, Unigene1329_All, Unigene1683_All, Unigene1782_All, Unigene1783_All, Unigene1819_All, Unigene185_All, Unigene1907_All, Unigene1931_All, Unigene2282_All, Unigene2292_All, Unigene2340_All, Unigene255_All, Unigene2560_All, Unigene2613_All, Unigene2681_All, Unigene2709_All, Unigene2741_All, Unigene3023_All, Unigene3077_All, Unigene3079_All, Unigene3101_All, Unigene3774_All, Unigene4316_All, Unigene4371_All, Unigene4372_All, Unigene4406_All, Unigene4439_All, Unigene4464_All, Unigene4503_All, Unigene4628_All, Unigene4669_All, Unigene4679_All, Unigene4680_All, Unigene4685_All, Unigene4687_All, Unigene4688_All, Unigene4741_All, Unigene4805_All, Unigene4810_All, Unigene4953_All, Unigene5339_All, Unigene5362_All, Unigene5413_All, Unigene5437_All, Unigene560_All, Unigene607_All, Unigene939_All
17			CL1.Contig132_All, CL1.Contig89_All, CL10.Contig11_All, CL10.Contig18_All, CL10.Contig9_All, CL10066.Contig1_All,

	RNA degradation	CL10249.Contig1_All, CL10250.Contig1_All, CL10263.Contig1_All, CL10285.Contig1_All, CL10396.Contig1_All, CL1053.Contig1_All, CL10549.Contig1_All, CL10582.Contig1_All, CL10618.Contig1_All, CL10645.Contig1_All, CL10705.Contig1_All, CL10846.Contig1_All, CL10846.Contig2_All, CL10858.Contig1_All, CL10976.Contig1_All, CL11071.Contig1_All, CL11102.Contig1_All, CL11301.Contig1_All, CL11393.Contig1_All, CL11415.Contig1_All, CL11644.Contig1_All, CL1176.Contig1_All, CL12014.Contig1_All, CL12040.Contig1_All, CL12040.Contig2_All, CL12191.Contig1_All, CL12253.Contig1_All, CL12256.Contig1_All, CL12269.Contig1_All, CL12407.Contig1_All, CL12425.Contig1_All, CL12696.Contig1_All, CL13218.Contig1_All, CL13248.Contig2_All, CL13309.Contig1_All, CL1341.Contig1_All, CL13747.Contig1_All, CL14178.Contig1_All, CL14286.Contig1_All, CL14296.Contig1_All, CL14326.Contig1_All, CL144.Contig4_All, CL144.Contig6_All, CL144.Contig7_All, CL144.Contig8_All, CL14845.Contig1_All, CL1520.Contig1_All, CL15299.Contig1_All, CL15371.Contig1_All, CL1555.Contig1_All, CL1555.Contig2_All, CL15556.Contig1_All, CL15576.Contig1_All, CL15583.Contig2_All, CL15618.Contig1_All, CL15823.Contig1_All, CL160.Contig2_All, CL160.Contig5_All, CL16000.Contig1_All, CL16033.Contig1_All, CL1617.Contig1_All, CL16211.Contig1_All, CL16364.Contig1_All, CL16576.Contig1_All, CL16603.Contig1_All, CL16668.Contig1_All, CL16699.Contig1_All, CL16774.Contig1_All, CL16826.Contig1_All, CL16842.Contig1_All, CL16896.Contig1_All, CL17036.Contig1_All, CL17143.Contig1_All, CL1722.Contig1_All, CL17234.Contig1_All, CL17239.Contig1_All, CL17360.Contig1_All, CL17490.Contig1_All, CL17573.Contig1_All, CL17597.Contig1_All, CL17597.Contig2_All, CL17663.Contig1_All, CL1782.Contig1_All, CL18036.Contig1_All, CL18135.Contig1_All, CL1817.Contig1_All, CL18207.Contig1_All, CL18239.Contig1_All, CL18308.Contig1_All, CL18314.Contig1_All, CL18388.Contig1_All, CL18391.Contig1_All, CL18475.Contig1_All, CL18816.Contig1_All, CL18827.Contig1_All, CL18833.Contig1_All, CL18931.Contig1_All, CL19082.Contig1_All, CL19159.Contig1_All, CL19213.Contig1_All, CL19333.Contig1_All, CL19427.Contig1_All, CL1970.Contig1_All, CL19745.Contig1_All, CL19856.Contig1_All, CL20049.Contig1_All, CL2012.Contig1_All, CL20218.Contig1_All, CL20236.Contig1_All, CL20533.Contig1_All, CL20577.Contig1_All,

			CL20659.Contig1_All, CL20795.Contig1_All, CL20844.Contig1_All, CL209.Contig1_All, CL209.Contig2_All, CL209.Contig3_All, CL209.Contig4_All, CL209.Contig5_All, CL21066.Contig1_All, CL2126.Contig1_All, CL2126.Contig2_All, CL21402.Contig1_All, CL2163.Contig1_All, CL21683.Contig1_All, CL21724.Contig1_All, CL21752.Contig1_All, CL21753.Contig1_All, CL21845.Contig1_All, CL21973.Contig1_All, CL22132.Contig1_All, CL22826.Contig1_All, CL2284.Contig1_All, CL2291.Contig1_All, CL22921.Contig1_All, CL23077.Contig1_All, CL23117.Contig1_All, CL2318.Contig1_All, CL23263.Contig1_All, CL23411.Contig1_All, CL23859.Contig1_All, CL23965.Contig1_All, CL24024.Contig1_All, CL24281.Contig1_All, CL243.Contig2_All, CL243.Contig3_All, CL243.Contig4_All, CL243.Contig5_All, CL2440.Contig1_All, CL24898.Contig1_All, CL25020.Contig1_All, CL25027.Contig1_All, CL2514.Contig1_All, CL25333.Contig1_All, CL25785.Contig1_All, CL25900.Contig1_All, CL2600.Contig1_All, CL26190.Contig1_All, CL26227.Contig1_All, CL26249.Contig1_All, CL26400.Contig1_All, CL26431.Contig1_All, CL26578.Contig1_All, CL27086.Contig1_All, CL27142.Contig1_All, CL275.Contig2_All, CL27554.Contig1_All, CL27693.Contig1_All, CL27757.Contig1_All, CL27902.Contig1_All, CL28135.Contig1_All, CL2951.Contig1_All, CL2951.Contig2_All, CL3040.Contig1_All, CL3040.Contig2_All, CL306.Contig1_All, CL306.Contig2_All, CL306.Contig3_All, CL3090.Contig1_All, CL3174.Contig1_All, CL3204.Contig1_All, CL3262.Contig1_All, CL329.Contig1_All, CL329.Contig2_All, CL329.Contig3_All, CL329.Contig4_All, CL33.Contig12_All, CL33.Contig13_All, CL3389.Contig1_All, CL3422.Contig1_All, CL3422.Contig2_All, CL3422.Contig3_All, CL3490.Contig1_All, CL3494.Contig1_All, CL3517.Contig1_All, CL359.Contig1_All, CL359.Contig2_All, CL359.Contig3_All, CL359.Contig4_All, CL3646.Contig2_All, CL3655.Contig1_All, CL3802.Contig1_All, CL3839.Contig1_All, CL3865.Contig1_All, CL3942.Contig1_All, CL3953.Contig1_All, CL4080.Contig1_All, CL41.Contig10_All, CL4297.Contig1_All, CL4318.Contig1_All, CL436.Contig1_All, CL4449.Contig1_All, CL4632.Contig1_All, CL4632.Contig2_All, CL4641.Contig1_All, CL4641.Contig2_All, CL4760.Contig1_All, CL4851.Contig1_All, CL4851.Contig2_All, CL4862.Contig1_All, CL4889.Contig1_All, CL5008.Contig1_All, CL5008.Contig2_All, CL527.Contig1_All, CL531.Contig1_All, CL531.Contig2_All, CL5402.Contig1_All, CL5416.Contig1_All, CL5442.Contig1_All, CL55.Contig2_All, CL5530.Contig1_All, CL5530.Contig2_All, CL5558.Contig1_All, CL5567.Contig1_All, CL5573.Contig1_All,

			CL5757.Contig2_All, CL5822.Contig1_All, CL5845.Contig1_All, CL5845.Contig2_All, CL5946.Contig1_All, CL6001.Contig1_All, CL6070.Contig1_All, CL6073.Contig1_All, CL611.Contig1_All, CL617.Contig1_All, CL625.Contig2_All, CL625.Contig4_All, CL625.Contig5_All, CL625.Contig6_All, CL6292.Contig1_All, CL64.Contig1_All, CL64.Contig2_All, CL64.Contig3_All, CL64.Contig4_All, CL64.Contig5_All, CL64.Contig6_All, CL64.Contig7_All, CL6443.Contig1_All, CL6742.Contig1_All, CL687.Contig1_All, CL687.Contig2_All, CL687.Contig3_All, CL687.Contig4_All, CL687.Contig5_All, CL687.Contig6_All, CL6886.Contig1_All, CL689.Contig1_All, CL6899.Contig1_All, CL6916.Contig1_All, CL6980.Contig1_All, CL7067.Contig1_All, CL7231.Contig1_All, CL7237.Contig1_All, CL7350.Contig1_All, CL7353.Contig1_All, CL7360.Contig1_All, CL7408.Contig1_All, CL7455.Contig1_All, CL7727.Contig1_All, CL7755.Contig1_All, CL78.Contig3_All, CL78.Contig7_All, CL7886.Contig1_All, CL791.Contig3_All, CL791.Contig4_All, CL8013.Contig1_All, CL806.Contig1_All, CL8081.Contig1_All, CL8149.Contig1_All, CL8155.Contig1_All, CL8257.Contig1_All, CL826.Contig1_All, CL8303.Contig1_All, CL831.Contig1_All, CL831.Contig2_All, CL8332.Contig1_All, CL835.Contig1_All, CL835.Contig2_All, CL8470.Contig1_All, CL8532.Contig1_All, CL8621.Contig1_All, CL8661.Contig1_All, CL8714.Contig1_All, CL8719.Contig1_All, CL8772.Contig1_All, CL8806.Contig1_All, CL8817.Contig1_All, CL8953.Contig1_All, CL910.Contig1_All, CL910.Contig2_All, CL910.Contig3_All, CL910.Contig4_All, CL9124.Contig1_All, CL913.Contig1_All, CL913.Contig2_All, CL913.Contig3_All, CL913.Contig4_All, CL9409.Contig1_All, CL9470.Contig1_All, CL9680.Contig1_All, CL9802.Contig2_All, CL9803.Contig1_All, CL9814.Contig1_All, Unigene1068_All, Unigene1092_All, Unigene1094_All, Unigene1100_All, Unigene1107_All, Unigene1257_All, Unigene1408_All, Unigene1602_All, Unigene1733_All, Unigene214_All, Unigene2244_All, Unigene2436_All, Unigene2611_All, Unigene2658_All, Unigene2805_All, Unigene2812_All, Unigene2868_All, Unigene3036_All, Unigene3255_All, Unigene3258_All, Unigene3778_All, Unigene398_All, Unigene4078_All, Unigene4169_All, Unigene4441_All, Unigene446_All, Unigene5079_All, Unigene5513_All, Unigene724_All, Unigene74_All, Unigene798_All
18			CL10055.Contig1_All, CL10142.Contig1_All, CL10556.Contig1_All, CL10585.Contig1_All, CL1077.Contig1_All, CL10775.Contig1_All, CL10877.Contig1_All, CL10951.Contig1_All, CL11032.Contig1_All, CL11047.Contig1_All, CL11064.Contig1_All, CL11093.Contig1_All, CL11163.Contig1_All, CL11351.Contig1_All, CL11524.Contig1_All, CL1196.Contig2_All, CL12218.Contig1_All, CL12239.Contig1_All,

	Phenylpropanoid	CL12370.Contig1_All, CL12443.Contig1_All, CL12470.Contig1_All, CL1266.Contig1_All, CL12702.Contig1_All, CL12820.Contig1_All, CL12842.Contig1_All, CL12917.Contig1_All, CL13068.Contig1_All, CL13172.Contig1_All, CL13244.Contig1_All, CL13594.Contig1_All, CL13635.Contig1_All, CL1409.Contig1_All, CL1409.Contig2_All, CL1409.Contig3_All, CL1409.Contig4_All, CL1409.Contig5_All, CL14311.Contig1_All, CL14522.Contig1_All, CL14726.Contig1_All, CL14758.Contig1_All, CL15602.Contig1_All, CL15606.Contig1_All, CL15670.Contig1_All, CL15730.Contig1_All, CL15730.Contig2_All, CL15813.Contig1_All, CL16130.Contig1_All, CL16203.Contig1_All, CL16277.Contig1_All, CL16277.Contig2_All, CL1645.Contig1_All, CL16643.Contig1_All, CL16663.Contig1_All, CL16726.Contig1_All, CL16810.Contig1_All, CL16918.Contig1_All, CL17363.Contig1_All, CL17498.Contig1_All, CL17539.Contig1_All, CL17542.Contig1_All, CL17719.Contig1_All, CL17858.Contig1_All, CL17992.Contig1_All, CL18183.Contig1_All, CL18330.Contig1_All, CL18418.Contig1_All, CL18510.Contig1_All, CL1855.Contig3_All, CL18550.Contig1_All, CL1881.Contig1_All, CL1881.Contig2_All, CL1881.Contig3_All, CL18841.Contig1_All, CL18869.Contig1_All, CL1919.Contig1_All, CL19381.Contig1_All, CL19619.Contig1_All, CL19792.Contig1_All, CL19817.Contig1_All, CL20001.Contig1_All, CL20154.Contig1_All, CL20335.Contig1_All, CL20344.Contig1_All, CL20987.Contig1_All, CL21389.Contig1_All, CL21611.Contig1_All, CL22.Contig10_All, CL22.Contig11_All, CL22.Contig12_All, CL22.Contig13_All, CL22.Contig14_All, CL22.Contig15_All, CL22.Contig16_All, CL22.Contig17_All, CL22.Contig18_All, CL22.Contig19_All, CL22.Contig1_All, CL22.Contig20_All, CL22.Contig21_All, CL22.Contig22_All, CL22.Contig2_All, CL22.Contig3_All, CL22.Contig4_All, CL22.Contig5_All, CL22.Contig6_All, CL22.Contig7_All, CL22.Contig8_All, CL22.Contig9_All, CL22035.Contig1_All, CL22098.Contig1_All, CL22102.Contig1_All, CL22250.Contig1_All, CL22294.Contig1_All, CL2245.Contig1_All, CL2245.Contig2_All, CL22464.Contig1_All, CL22561.Contig1_All, CL2262.Contig1_All, CL2262.Contig2_All, CL2262.Contig3_All, CL2262.Contig4_All, CL22771.Contig1_All, CL22926.Contig1_All, CL2306.Contig1_All, CL23369.Contig1_All, CL2385.Contig1_All, CL23945.Contig1_All, CL2414.Contig1_All, CL24216.Contig1_All, CL2461.Contig1_All, CL2461.Contig2_All, CL2461.Contig3_All, CL2461.Contig4_All,

	biosynthesis	CL24764.Contig1_All, CL24900.Contig1_All, CL24951.Contig1_All, CL2516.Contig1_All, CL25208.Contig1_All, CL25414.Contig1_All, CL25490.Contig1_All, CL25612.Contig1_All, CL25689.Contig1_All, CL257.Contig1_All, CL257.Contig2_All, CL25749.Contig1_All, CL25996.Contig1_All, CL26226.Contig1_All, CL26257.Contig1_All, CL26274.Contig1_All, CL26484.Contig1_All, CL26736.Contig1_All, CL26885.Contig1_All, CL26967.Contig1_All, CL27033.Contig1_All, CL27064.Contig1_All, CL27308.Contig1_All, CL281.Contig1_All, CL281.Contig2_All, CL281.Contig3_All, CL281.Contig4_All, CL281.Contig5_All, CL281.Contig6_All, CL281.Contig7_All, CL2879.Contig1_All, CL2991.Contig1_All, CL2991.Contig2_All, CL2991.Contig3_All, CL2995.Contig1_All, CL2995.Contig2_All, CL3053.Contig1_All, CL3076.Contig1_All, CL3191.Contig1_All, CL3211.Contig1_All, CL3225.Contig1_All, CL3225.Contig3_All, CL3310.Contig1_All, CL3321.Contig1_All, CL3321.Contig2_All, CL3321.Contig3_All, CL3413.Contig1_All, CL3413.Contig2_All, CL3476.Contig1_All, CL3476.Contig2_All, CL3788.Contig1_All, CL40.Contig11_All, CL40.Contig3_All, CL4024.Contig1_All, CL4095.Contig1_All, CL412.Contig1_All, CL412.Contig2_All, CL412.Contig3_All, CL4120.Contig1_All, CL4121.Contig1_All, CL4129.Contig1_All, CL4280.Contig1_All, CL4312.Contig1_All, CL4312.Contig2_All, CL4471.Contig1_All, CL4591.Contig2_All, CL4591.Contig3_All, CL4731.Contig1_All, CL4743.Contig1_All, CL4745.Contig1_All, CL4846.Contig1_All, CL4876.Contig1_All, CL4961.Contig1_All, CL4963.Contig1_All, CL50.Contig16_All, CL50.Contig9_All, CL5092.Contig1_All, CL5103.Contig1_All, CL511.Contig4_All, CL511.Contig5_All, CL5215.Contig1_All, CL5231.Contig1_All, CL5266.Contig1_All, CL5325.Contig1_All, CL5426.Contig1_All, CL5463.Contig1_All, CL5477.Contig1_All, CL5523.Contig1_All, CL5639.Contig1_All, CL5843.Contig1_All, CL6089.Contig1_All, CL6146.Contig1_All, CL619.Contig1_All, CL6287.Contig1_All, CL6410.Contig2_All, CL6466.Contig2_All, CL6626.Contig1_All, CL6792.Contig1_All, CL6792.Contig2_All, CL6799.Contig1_All, CL6799.Contig2_All, CL6877.Contig2_All, CL702.Contig1_All, CL702.Contig2_All, CL702.Contig3_All, CL702.Contig4_All, CL7584.Contig1_All, CL766.Contig1_All, CL766.Contig2_All, CL766.Contig3_All, CL766.Contig4_All, CL766.Contig5_All, CL7707.Contig1_All, CL7758.Contig1_All, CL7840.Contig1_All, CL801.Contig3_All, CL832.Contig1_All, CL832.Contig2_All, CL8324.Contig1_All, CL8381.Contig1_All, CL8384.Contig1_All, CL8414.Contig1_All, CL8485.Contig1_All, CL8630.Contig1_All, CL8749.Contig1_All,

		CL8773.Contig1_All, CL8823.Contig1_All, CL8905.Contig1_All, CL8962.Contig1_All, CL9178.Contig1_All, CL9407.Contig1_All, CL9539.Contig1_All, CL9688.Contig1_All, CL9723.Contig1_All, Unigene1055_All, Unigene1232_All, Unigene1543_All, Unigene1694_All, Unigene1788_All, Unigene1909_All, Unigene2232_All, Unigene2334_All, Unigene2335_All, Unigene2338_All, Unigene2429_All, Unigene2449_All, Unigene2590_All, Unigene2697_All, Unigene2865_All, Unigene2961_All, Unigene2962_All, Unigene2963_All, Unigene2964_All, Unigene3163_All, Unigene316_All, Unigene342_All, Unigene3648_All, Unigene3670_All, Unigene3751_All, Unigene4180_All, Unigene4302_All, Unigene4417_All, Unigene462_All, Unigene4655_All, Unigene4656_All, Unigene4671_All, Unigene4973_All, Unigene4983_All, Unigene5117_All, Unigene5236_All, Unigene5238_All, Unigene5262_All, Unigene5329_All, Unigene5426_All, Unigene5446_All, Unigene5518_All, Unigene5525_All, Unigene557_All, Unigene708_All, Unigene719_All, Unigene720_All, Unigene793_All, Unigene800_All, Unigene825_All, Unigene895_All, Unigene938_All
19		CL1.Contig102_All, CL1.Contig12_All, CL1.Contig57_All, CL10020.Contig1_All, CL10058.Contig1_All, CL10115.Contig1_All, CL10165.Contig1_All, CL10406.Contig1_All, CL10477.Contig1_All, CL10497.Contig1_All, CL106.Contig1_All, CL106.Contig2_All, CL106.Contig3_All, CL106.Contig4_All, CL106.Contig5_All, CL106.Contig6_All, CL10652.Contig1_All, CL10746.Contig1_All, CL1084.Contig1_All, CL1084.Contig2_All, CL1084.Contig3_All, CL1084.Contig4_All, CL1084.Contig5_All, CL11110.Contig1_All, CL11137.Contig1_All, CL11279.Contig1_All, CL11464.Contig1_All, CL1177.Contig1_All, CL118.Contig10_All, CL118.Contig12_All, CL118.Contig13_All, CL118.Contig14_All, CL118.Contig4_All, CL118.Contig5_All, CL12668.Contig1_All, CL12938.Contig1_All, CL13080.Contig1_All, CL1342.Contig1_All, CL1342.Contig2_All, CL1342.Contig3_All, CL1342.Contig4_All, CL13523.Contig1_All, CL13714.Contig1_All, CL13729.Contig1_All, CL13776.Contig1_All, CL14122.Contig1_All, CL14122.Contig2_All, CL14140.Contig1_All, CL14164.Contig1_All, CL1424.Contig1_All, CL1426.Contig1_All, CL14423.Contig1_All, CL14503.Contig1_All, CL14510.Contig1_All, CL14510.Contig2_All, CL1484.Contig1_All, CL1484.Contig2_All, CL1484.Contig3_All, CL14954.Contig1_All, CL14981.Contig1_All, CL15039.Contig1_All, CL15100.Contig1_All, CL15260.Contig1_All, CL15275.Contig1_All, CL15378.Contig1_All, CL15387.Contig1_All, CL1548.Contig2_All, CL15697.Contig1_All,

	Pyrimidine metabolism	CL15878.Contig1_All, CL16128.Contig1_All, CL16204.Contig1_All, CL16425.Contig1_All, CL16515.Contig1_All, CL1672.Contig1_All, CL16771.Contig1_All, CL17057.Contig1_All, CL17158.Contig1_All, CL17405.Contig1_All, CL17512.Contig1_All, CL18317.Contig1_All, CL18325.Contig1_All, CL18441.Contig1_All, CL18538.Contig1_All, CL1863.Contig2_All, CL1863.Contig3_All, CL18907.Contig1_All, CL1893.Contig1_All, CL19148.Contig1_All, CL19445.Contig1_All, CL19651.Contig1_All, CL19667.Contig1_All, CL1977.Contig1_All, CL19812.Contig1_All, CL19831.Contig1_All, CL20405.Contig1_All, CL2062.Contig1_All, CL20934.Contig1_All, CL21003.Contig1_All, CL2108.Contig1_All, CL2108.Contig2_All, CL2129.Contig2_All, CL21309.Contig1_All, CL2179.Contig1_All, CL2181.Contig1_All, CL22012.Contig1_All, CL22044.Contig1_All, CL22823.Contig1_All, CL22913.Contig1_All, CL23087.Contig1_All, CL23155.Contig1_All, CL23227.Contig1_All, CL23312.Contig1_All, CL23363.Contig1_All, CL23570.Contig1_All, CL23629.Contig1_All, CL23834.Contig1_All, CL23911.Contig1_All, CL24063.Contig1_All, CL24517.Contig1_All, CL24536.Contig1_All, CL24615.Contig1_All, CL24622.Contig1_All, CL24635.Contig1_All, CL24709.Contig1_All, CL24714.Contig1_All, CL24786.Contig1_All, CL24825.Contig1_All, CL24839.Contig1_All, CL25009.Contig1_All, CL25044.Contig1_All, CL25051.Contig1_All, CL25107.Contig1_All, CL25192.Contig1_All, CL25340.Contig1_All, CL25515.Contig1_All, CL25833.Contig1_All, CL25836.Contig1_All, CL26303.Contig1_All, CL264.Contig8_All, CL26534.Contig1_All, CL26598.Contig1_All, CL26623.Contig1_All, CL2679.Contig1_All, CL26933.Contig1_All, CL26953.Contig1_All, CL27180.Contig1_All, CL2724.Contig1_All, CL2775.Contig1_All, CL27834.Contig1_All, CL27901.Contig1_All, CL2796.Contig1_All, CL2796.Contig2_All, CL2796.Contig3_All, CL2796.Contig4_All, CL2839.Contig1_All, CL3027.Contig1_All, CL3134.Contig1_All, CL318.Contig2_All, CL318.Contig4_All, CL3367.Contig1_All, CL3367.Contig2_All, CL3376.Contig1_All, CL3434.Contig1_All, CL351.Contig1_All, CL351.Contig5_All, CL3564.Contig1_All, CL3750.Contig1_All, CL3905.Contig1_All, CL3996.Contig1_All, CL4030.Contig1_All, CL4205.Contig1_All, CL4359.Contig1_All, CL436.Contig1_All, CL4363.Contig1_All, CL4363.Contig3_All, CL4453.Contig1_All, CL4468.Contig1_All, CL4494.Contig1_All, CL4593.Contig1_All, CL4616.Contig1_All,

			CL4636.Contig1_All, CL4724.Contig1_All, CL475.Contig1_All, CL475.Contig2_All, CL475.Contig3_All, CL475.Contig4_All, CL479.Contig2_All, CL479.Contig3_All, CL479.Contig5_All, CL4793.Contig1_All, CL488.Contig1_All, CL488.Contig2_All, CL488.Contig3_All, CL488.Contig4_All, CL4903.Contig1_All, CL498.Contig1_All, CL5173.Contig1_All, CL5243.Contig1_All, CL5282.Contig1_All, CL5282.Contig2_All, CL5569.Contig1_All, CL5607.Contig2_All, CL5618.Contig1_All, CL5618.Contig2_All, CL577.Contig1_All, CL577.Contig2_All, CL577.Contig3_All, CL5854.Contig1_All, CL6042.Contig1_All, CL6053.Contig1_All, CL6299.Contig1_All, CL6388.Contig1_All, CL6424.Contig1_All, CL6515.Contig1_All, CL6763.Contig1_All, CL6917.Contig1_All, CL6928.Contig1_All, CL699.Contig1_All, CL6991.Contig1_All, CL7040.Contig1_All, CL7090.Contig1_All, CL7112.Contig1_All, CL7167.Contig1_All, CL7279.Contig1_All, CL7379.Contig1_All, CL7379.Contig2_All, CL7412.Contig2_All, CL7561.Contig1_All, CL77.Contig10_All, CL77.Contig1_All, CL77.Contig2_All, CL77.Contig5_All, CL77.Contig7_All, CL77.Contig8_All, CL77.Contig9_All, CL771.Contig1_All, CL7796.Contig1_All, CL7923.Contig1_All, CL7923.Contig2_All, CL8159.Contig1_All, CL8213.Contig1_All, CL8219.Contig2_All, CL8358.Contig1_All, CL8437.Contig1_All, CL8487.Contig1_All, CL8511.Contig1_All, CL8528.Contig1_All, CL8564.Contig1_All, CL8639.Contig1_All, CL8639.Contig2_All, CL8644.Contig1_All, CL8662.Contig1_All, CL8668.Contig1_All, CL8724.Contig1_All, CL8859.Contig1_All, CL9093.Contig1_All, CL9266.Contig1_All, CL928.Contig3_All, CL9303.Contig1_All, CL9385.Contig1_All, CL9570.Contig1_All, CL9797.Contig1_All, CL9799.Contig1_All, CL9987.Contig1_All, Unigene1335_All, Unigene1386_All, Unigene1597_All, Unigene1792_All, Unigene1935_All, Unigene2102_All, Unigene2133_All, Unigene2195_All, Unigene229_All, Unigene234_All, Unigene2408_All, Unigene2509_All, Unigene2550_All, Unigene2613_All, Unigene2687_All, Unigene2719_All, Unigene2876_All, Unigene2881_All, Unigene3151_All, Unigene3236_All, Unigene3275_All, Unigene3522_All, Unigene3657_All, Unigene3789_All, Unigene4007_All, Unigene4017_All, Unigene4038_All, Unigene4051_All, Unigene4211_All, Unigene4444_All, Unigene606_All, Unigene734_All, Unigene87_All
20			CL10476.Contig1_All, CL10491.Contig1_All, CL10505.Contig1_All, CL10583.Contig1_All, CL10638.Contig1_All, CL10651.Contig1_All, CL10766.Contig1_All, CL10875.Contig1_All, CL10972.Contig1_All, CL11003.Contig1_All, CL11023.Contig1_All, CL11033.Contig1_All, CL11067.Contig1_All, CL11212.Contig1_All,

	[Oxidative phosphorylation]()	CL11213.Contig1_All, CL11416.Contig1_All, CL11433.Contig1_All, CL11576.Contig1_All, CL11734.Contig1_All, CL11738.Contig1_All, CL11838.Contig1_All, CL11912.Contig1_All, CL12039.Contig1_All, CL12039.Contig2_All, CL12064.Contig1_All, CL12075.Contig1_All, CL12104.Contig1_All, CL12174.Contig1_All, CL12211.Contig1_All, CL12424.Contig1_All, CL12530.Contig1_All, CL12765.Contig1_All, CL12857.Contig1_All, CL12944.Contig1_All, CL12980.Contig1_All, CL13046.Contig1_All, CL13060.Contig1_All, CL13272.Contig1_All, CL13341.Contig1_All, CL13356.Contig1_All, CL13381.Contig1_All, CL13604.Contig1_All, CL13630.Contig1_All, CL13817.Contig1_All, CL14038.Contig1_All, CL14060.Contig1_All, CL14074.Contig1_All, CL14119.Contig1_All, CL14141.Contig1_All, CL14169.Contig1_All, CL14225.Contig1_All, CL14273.Contig1_All, CL14283.Contig1_All, CL14293.Contig1_All, CL14315.Contig1_All, CL14375.Contig1_All, CL14385.Contig1_All, CL14388.Contig1_All, CL14400.Contig1_All, CL14505.Contig1_All, CL14573.Contig1_All, CL14574.Contig1_All, CL14590.Contig1_All, CL14592.Contig1_All, CL14605.Contig1_All, CL14728.Contig1_All, CL14751.Contig1_All, CL1477.Contig1_All, CL1477.Contig2_All, CL1477.Contig3_All, CL14795.Contig1_All, CL14883.Contig1_All, CL14892.Contig1_All, CL14897.Contig1_All, CL15015.Contig1_All, CL15079.Contig1_All, CL15119.Contig1_All, CL15143.Contig1_All, CL15161.Contig1_All, CL15253.Contig1_All, CL15284.Contig1_All, CL15430.Contig1_All, CL15518.Contig1_All, CL15530.Contig1_All, CL15565.Contig1_All, CL16187.Contig1_All, CL16385.Contig1_All, CL16703.Contig1_All, CL16824.Contig1_All, CL17124.Contig1_All, CL17785.Contig1_All, CL1792.Contig1_All, CL1792.Contig2_All, CL181.Contig1_All, CL181.Contig2_All, CL181.Contig3_All, CL181.Contig5_All, CL18104.Contig1_All, CL18211.Contig1_All, CL1831.Contig1_All, CL18440.Contig1_All, CL18727.Contig1_All, CL18904.Contig1_All, CL19644.Contig1_All, CL1983.Contig1_All, CL2015.Contig1_All, CL20366.Contig1_All, CL2046.Contig1_All, CL20496.Contig1_All, CL20638.Contig1_All, CL20664.Contig1_All, CL20667.Contig1_All, CL20672.Contig1_All, CL20879.Contig1_All, CL20897.Contig1_All, CL21079.Contig1_All, CL21568.Contig1_All, CL21734.Contig1_All, CL2214.Contig1_All, CL22206.Contig1_All, CL22382.Contig1_All, CL22531.Contig1_All, CL22788.Contig1_All, CL22974.Contig1_All,

			CL23133.Contig1_All, CL23220.Contig1_All, CL23223.Contig1_All, CL23560.Contig1_All, CL23632.Contig1_All, CL23735.Contig1_All, CL23768.Contig1_All, CL23791.Contig1_All, CL23845.Contig1_All, CL24066.Contig1_All, CL24319.Contig1_All, CL24664.Contig1_All, CL24705.Contig1_All, CL2475.Contig1_All, CL2505.Contig1_All, CL2505.Contig2_All, CL25387.Contig1_All, CL25472.Contig1_All, CL25555.Contig1_All, CL25667.Contig1_All, CL2568.Contig1_All, CL2568.Contig2_All, CL2568.Contig3_All, CL2568.Contig4_All, CL25705.Contig1_All, CL25761.Contig1_All, CL25791.Contig1_All, CL25813.Contig1_All, CL25852.Contig1_All, CL25939.Contig1_All, CL25955.Contig1_All, CL26026.Contig1_All, CL26145.Contig1_All, CL26467.Contig1_All, CL26518.Contig1_All, CL26593.Contig1_All, CL26639.Contig1_All, CL26751.Contig1_All, CL26756.Contig1_All, CL26769.Contig1_All, CL26827.Contig1_All, CL26904.Contig1_All, CL27281.Contig1_All, CL273.Contig1_All, CL27603.Contig1_All, CL27720.Contig1_All, CL27816.Contig1_All, CL27957.Contig1_All, CL27965.Contig1_All, CL28009.Contig1_All, CL28088.Contig1_All, CL3317.Contig1_All, CL4182.Contig1_All, CL4182.Contig2_All, CL4231.Contig1_All, CL4231.Contig2_All, CL4268.Contig1_All, CL4536.Contig1_All, CL4776.Contig1_All, CL5288.Contig1_All, CL5288.Contig2_All, CL5288.Contig3_All, CL54.Contig18_All, CL5633.Contig1_All, CL5633.Contig2_All, CL57.Contig10_All, CL57.Contig11_All, CL57.Contig1_All, CL57.Contig2_All, CL57.Contig3_All, CL57.Contig4_All, CL57.Contig5_All, CL57.Contig6_All, CL57.Contig7_All, CL57.Contig8_All, CL57.Contig9_All, CL5922.Contig1_All, CL6153.Contig1_All, CL6153.Contig2_All, CL6224.Contig1_All, CL63.Contig5_All, CL63.Contig7_All, CL6612.Contig1_All, CL6612.Contig2_All, CL6720.Contig1_All, CL7296.Contig1_All, CL7453.Contig1_All, CL7526.Contig1_All, CL7526.Contig2_All, CL7700.Contig1_All, CL774.Contig1_All, CL774.Contig2_All, CL774.Contig3_All, CL774.Contig4_All, CL7799.Contig1_All, CL8132.Contig1_All, CL8132.Contig2_All, CL8494.Contig1_All, CL8500.Contig1_All, CL8500.Contig2_All, CL876.Contig3_All, CL8949.Contig1_All, CL9028.Contig1_All, CL9095.Contig1_All, CL9109.Contig1_All, CL9122.Contig1_All, CL9172.Contig1_All, CL9222.Contig1_All, CL9270.Contig1_All, CL9274.Contig1_All, CL9339.Contig1_All, CL973.Contig2_All, CL9810.Contig1_All, CL9916.Contig1_All, CL9935.Contig1_All, CL9938.Contig1_All, CL998.Contig2_All, CL998.Contig3_All, CL998.Contig4_All, CL9990.Contig1_All, Unigene102_All, Unigene1037_All, Unigene1108_All,

			Unigene1150_All, Unigene1155_All, Unigene1222_All, Unigene1266_All, Unigene144_All, Unigene1483_All, Unigene1539_All, Unigene177_All, Unigene1889_All, Unigene2290_All, Unigene235_All, Unigene239_All, Unigene252_All, Unigene3203_All, Unigene324_All, Unigene335_All, Unigene350_All, Unigene3548_All, Unigene366_All, Unigene3680_All, Unigene3772_All, Unigene4354_All, Unigene4397_All, Unigene4567_All, Unigene4633_All, Unigene5010_All, Unigene5224_All, Unigene5476_All, Unigene5491_All, Unigene5499_All, Unigene915_All
21			CL10155.Contig1_All, CL10198.Contig2_All, CL10347.Contig1_All, CL10445.Contig1_All, CL10448.Contig1_All, CL10691.Contig1_All, CL10800.Contig1_All, CL10864.Contig1_All, CL11022.Contig1_All, CL1111.Contig1_All, CL1111.Contig2_All, CL1113.Contig1_All, CL1113.Contig2_All, CL1113.Contig3_All, CL1113.Contig4_All, CL1113.Contig5_All, CL1113.Contig6_All, CL11162.Contig1_All, CL114.Contig10_All, CL114.Contig11_All, CL114.Contig12_All, CL114.Contig1_All, CL114.Contig2_All, CL114.Contig3_All, CL114.Contig4_All, CL114.Contig5_All, CL114.Contig6_All, CL114.Contig7_All, CL114.Contig8_All, CL114.Contig9_All, CL1145.Contig1_All, CL1145.Contig2_All, CL1145.Contig3_All, CL11477.Contig1_All, CL11617.Contig1_All, CL11719.Contig1_All, CL11843.Contig1_All, CL11866.Contig1_All, CL11930.Contig1_All, CL12077.Contig1_All, CL12112.Contig1_All, CL12123.Contig1_All, CL12159.Contig1_All, CL12201.Contig1_All, CL12202.Contig1_All, CL12497.Contig1_All, CL12533.Contig1_All, CL12597.Contig1_All, CL12616.Contig1_All, CL12810.Contig1_All, CL12869.Contig1_All, CL12967.Contig1_All, CL13036.Contig1_All, CL13175.Contig1_All, CL13195.Contig1_All, CL13245.Contig1_All, CL13346.Contig1_All, CL13394.Contig1_All, CL13475.Contig1_All, CL13483.Contig1_All, CL1410.Contig1_All, CL14334.Contig1_All, CL14461.Contig1_All, CL14470.Contig1_All, CL14672.Contig1_All, CL14874.Contig1_All, CL15082.Contig1_All, CL151.Contig7_All, CL15189.Contig1_All, CL15189.Contig2_All, CL15361.Contig1_All, CL15401.Contig1_All, CL15947.Contig1_All, CL160.Contig3_All, CL16201.Contig1_All, CL16302.Contig1_All, CL16308.Contig1_All, CL16462.Contig1_All, CL16516.Contig1_All, CL16555.Contig1_All, CL16589.Contig1_All, CL1671.Contig1_All, CL17006.Contig1_All, CL17200.Contig1_All, CL17291.Contig1_All, CL17291.Contig2_All, CL17309.Contig1_All, CL17410.Contig1_All, CL17548.Contig1_All, CL17552.Contig1_All,

	Amino sugar and nucleotide sugar metabolism	CL17552.Contig2_All, CL1802.Contig1_All, CL18355.Contig1_All, CL1868.Contig1_All, CL18822.Contig1_All, CL19062.Contig1_All, CL19110.Contig1_All, CL1928.Contig2_All, CL19642.Contig1_All, CL198.Contig1_All, CL198.Contig2_All, CL198.Contig3_All, CL198.Contig4_All, CL198.Contig5_All, CL198.Contig6_All, CL198.Contig7_All, CL198.Contig8_All, CL198.Contig9_All, CL20170.Contig1_All, CL20562.Contig1_All, CL21274.Contig1_All, CL21298.Contig1_All, CL21429.Contig1_All, CL21780.Contig1_All, CL22227.Contig1_All, CL22446.Contig1_All, CL22514.Contig1_All, CL22739.Contig1_All, CL23006.Contig1_All, CL2301.Contig1_All, CL23202.Contig1_All, CL23524.Contig1_All, CL23777.Contig1_All, CL24.Contig11_All, CL24.Contig18_All, CL24.Contig21_All, CL24536.Contig1_All, CL24820.Contig1_All, CL25.Contig11_All, CL25.Contig12_All, CL25.Contig14_All, CL25.Contig15_All, CL25.Contig2_All, CL25.Contig9_All, CL250.Contig1_All, CL250.Contig2_All, CL250.Contig3_All, CL250.Contig4_All, CL250.Contig5_All, CL250.Contig6_All, CL2571.Contig1_All, CL25835.Contig1_All, CL25845.Contig1_All, CL2610.Contig2_All, CL26455.Contig1_All, CL26673.Contig1_All, CL2746.Contig1_All, CL2887.Contig1_All, CL2887.Contig2_All, CL2942.Contig2_All, CL2942.Contig3_All, CL2993.Contig1_All, CL3011.Contig1_All, CL3128.Contig1_All, CL3334.Contig1_All, CL3356.Contig1_All, CL3508.Contig1_All, CL3590.Contig1_All, CL38.Contig10_All, CL38.Contig8_All, CL4094.Contig1_All, CL4450.Contig1_All, CL4490.Contig1_All, CL4821.Contig1_All, CL5198.Contig1_All, CL5225.Contig1_All, CL5300.Contig1_All, CL5333.Contig1_All, CL5363.Contig1_All, CL5436.Contig1_All, CL5615.Contig1_All, CL5621.Contig1_All, CL5685.Contig1_All, CL5917.Contig2_All, CL5917.Contig3_All, CL5953.Contig1_All, CL6017.Contig1_All, CL6045.Contig1_All, CL6267.Contig1_All, CL6293.Contig1_All, CL6381.Contig1_All, CL6381.Contig2_All, CL6481.Contig1_All, CL6481.Contig2_All, CL6558.Contig1_All, CL6662.Contig1_All, CL6700.Contig1_All, CL6746.Contig1_All, CL6746.Contig2_All, CL6817.Contig1_All, CL6838.Contig1_All, CL7142.Contig1_All, CL717.Contig1_All, CL717.Contig2_All, CL717.Contig3_All, CL717.Contig4_All, CL7195.Contig2_All, CL728.Contig1_All, CL728.Contig2_All, CL728.Contig3_All, CL7381.Contig1_All, CL7388.Contig1_All, CL7438.Contig1_All, CL7487.Contig1_All, CL7528.Contig1_All, CL7528.Contig2_All, CL7664.Contig1_All, CL7676.Contig1_All, CL7680.Contig1_All, CL7725.Contig1_All, CL7772.Contig1_All, CL7909.Contig1_All,

			CL7909.Contig2_All, CL8293.Contig1_All, CL8300.Contig1_All, CL851.Contig4_All, CL8663.Contig1_All, CL8695.Contig1_All, CL8846.Contig1_All, CL8846.Contig2_All, CL8909.Contig1_All, CL8909.Contig2_All, CL9164.Contig1_All, CL9184.Contig1_All, CL9192.Contig1_All, CL921.Contig1_All, CL921.Contig2_All, CL921.Contig3_All, CL921.Contig4_All, CL921.Contig5_All, CL921.Contig6_All, CL921.Contig7_All, CL921.Contig8_All, CL9611.Contig1_All, CL9777.Contig1_All, CL996.Contig1_All, CL996.Contig2_All, CL996.Contig3_All, CL996.Contig4_All, CL996.Contig5_All, Unigene1017_All, Unigene1176_All, Unigene132_All, Unigene1441_All, Unigene173_All, Unigene1878_All, Unigene2552_All, Unigene2873_All, Unigene2921_All, Unigene3156_All, Unigene334_All, Unigene3423_All, Unigene380_All, Unigene4773_All, Unigene477_All, Unigene4839_All, Unigene523_All, Unigene641_All, Unigene831_All
22			CL10064.Contig1_All, CL10172.Contig1_All, CL1019.Contig1_All, CL1019.Contig2_All, CL1019.Contig3_All, CL1019.Contig4_All, CL10350.Contig1_All, CL10505.Contig1_All, CL11.Contig10_All, CL11.Contig1_All, CL11.Contig9_All, CL11023.Contig1_All, CL11067.Contig1_All, CL11138.Contig1_All, CL11210.Contig1_All, CL11212.Contig1_All, CL11548.Contig1_All, CL11706.Contig1_All, CL11753.Contig1_All, CL12075.Contig1_All, CL1214.Contig1_All, CL12147.Contig1_All, CL12211.Contig1_All, CL12522.Contig1_All, CL12643.Contig1_All, CL12645.Contig1_All, CL12880.Contig1_All, CL13046.Contig1_All, CL13060.Contig1_All, CL13061.Contig1_All, CL132.Contig10_All, CL132.Contig11_All, CL132.Contig1_All, CL132.Contig2_All, CL132.Contig3_All, CL132.Contig4_All, CL132.Contig5_All, CL132.Contig6_All, CL132.Contig7_All, CL132.Contig8_All, CL132.Contig9_All, CL13356.Contig1_All, CL13381.Contig1_All, CL13437.Contig1_All, CL13460.Contig1_All, CL13588.Contig1_All, CL13606.Contig1_All, CL13732.Contig1_All, CL13841.Contig1_All, CL13864.Contig1_All, CL14270.Contig1_All, CL14375.Contig1_All, CL1477.Contig1_All, CL1477.Contig2_All, CL1477.Contig3_All, CL14795.Contig1_All, CL14871.Contig1_All, CL14883.Contig1_All, CL14965.Contig1_All, CL15015.Contig1_All, CL15119.Contig1_All, CL15130.Contig1_All, CL15197.Contig1_All, CL15284.Contig1_All, CL153.Contig1_All, CL153.Contig2_All, CL153.Contig3_All, CL153.Contig4_All, CL153.Contig5_All, CL153.Contig6_All, CL153.Contig7_All, CL16187.Contig1_All, CL16257.Contig1_All, CL16342.Contig1_All, CL16373.Contig1_All, CL16384.Contig1_All, CL16478.Contig1_All, CL16824.Contig1_All, CL17178.Contig1_All, CL17220.Contig1_All,

	Phagosome	CL17267.Contig1_All, CL17768.Contig1_All, CL17906.Contig1_All, CL18264.Contig1_All, CL18806.Contig1_All, CL18976.Contig1_All, CL19219.Contig1_All, CL19319.Contig1_All, CL19680.Contig1_All, CL19758.Contig1_All, CL2015.Contig1_All, CL20313.Contig1_All, CL2046.Contig1_All, CL20460.Contig1_All, CL20796.Contig1_All, CL20897.Contig1_All, CL20917.Contig1_All, CL2096.Contig1_All, CL21566.Contig1_All, CL21788.Contig1_All, CL21971.Contig1_All, CL22153.Contig1_All, CL22382.Contig1_All, CL22537.Contig1_All, CL22974.Contig1_All, CL23220.Contig1_All, CL23238.Contig1_All, CL2341.Contig1_All, CL23631.Contig1_All, CL23663.Contig1_All, CL23765.Contig1_All, CL24400.Contig1_All, CL24638.Contig1_All, CL24705.Contig1_All, CL25014.Contig1_All, CL2518.Contig1_All, CL2537.Contig1_All, CL25667.Contig1_All, CL25791.Contig1_All, CL25800.Contig1_All, CL259.Contig2_All, CL259.Contig3_All, CL259.Contig5_All, CL259.Contig6_All, CL26225.Contig1_All, CL26427.Contig1_All, CL26467.Contig1_All, CL26639.Contig1_All, CL26707.Contig1_All, CL26756.Contig1_All, CL26872.Contig1_All, CL27054.Contig1_All, CL27153.Contig1_All, CL27281.Contig1_All, CL27497.Contig1_All, CL27720.Contig1_All, CL2790.Contig1_All, CL2790.Contig2_All, CL27938.Contig1_All, CL2795.Contig1_All, CL2795.Contig2_All, CL2795.Contig3_All, CL27953.Contig1_All, CL28088.Contig1_All, CL300.Contig1_All, CL300.Contig2_All, CL300.Contig3_All, CL300.Contig4_All, CL300.Contig5_All, CL300.Contig6_All, CL300.Contig7_All, CL305.Contig3_All, CL305.Contig4_All, CL305.Contig5_All, CL305.Contig6_All, CL305.Contig7_All, CL305.Contig8_All, CL305.Contig9_All, CL32.Contig4_All, CL346.Contig10_All, CL346.Contig11_All, CL346.Contig12_All, CL346.Contig13_All, CL346.Contig1_All, CL346.Contig2_All, CL346.Contig3_All, CL346.Contig4_All, CL346.Contig5_All, CL346.Contig6_All, CL346.Contig7_All, CL346.Contig8_All, CL346.Contig9_All, CL3550.Contig1_All, CL3550.Contig2_All, CL4123.Contig1_All, CL4182.Contig1_All, CL4182.Contig2_All, CL4231.Contig1_All, CL4231.Contig2_All, CL4536.Contig1_All, CL4570.Contig1_All, CL493.Contig1_All, CL493.Contig6_All, CL5253.Contig1_All, CL5288.Contig1_All, CL5288.Contig2_All, CL5288.Contig3_All, CL5310.Contig1_All, CL5635.Contig1_All, CL5848.Contig1_All, CL6224.Contig1_All, CL6237.Contig1_All, CL6237.Contig2_All, CL6237.Contig3_All, CL6570.Contig1_All, CL6612.Contig1_All, CL6612.Contig2_All, CL6673.Contig1_All, CL6673.Contig2_All, CL7.Contig25_All, CL7152.Contig1_All, CL7292.Contig1_All,

		CL7468.Contig1_All, CL764.Contig1_All, CL774.Contig1_All, CL774.Contig2_All, CL774.Contig3_All, CL774.Contig4_All, CL7782.Contig1_All, CL8107.Contig1_All, CL8133.Contig1_All, CL8355.Contig1_All, CL8367.Contig1_All, CL8371.Contig1_All, CL8494.Contig1_All, CL8495.Contig1_All, CL876.Contig3_All, CL9003.Contig1_All, CL9015.Contig1_All, CL9122.Contig1_All, CL926.Contig2_All, CL926.Contig3_All, CL9268.Contig1_All, CL9357.Contig1_All, CL9852.Contig1_All, CL998.Contig2_All, CL998.Contig3_All, CL998.Contig4_All, Unigene1037_All, Unigene1108_All, Unigene1259_All, Unigene1346_All, Unigene1483_All, Unigene1518_All, Unigene1987_All, Unigene239_All, Unigene3042_All, Unigene3099_All, Unigene324_All, Unigene3772_All, Unigene3845_All, Unigene4365_All, Unigene4397_All, Unigene4557_All, Unigene4761_All, Unigene4900_All, Unigene5290_All, Unigene5490_All, Unigene5514_All, Unigene915_All, Unigene953_All, Unigene966_All
23		CL1.Contig134_All, CL1008.Contig1_All, CL1008.Contig2_All, CL1008.Contig3_All, CL10180.Contig1_All, CL10180.Contig2_All, CL10328.Contig1_All, CL10399.Contig1_All, CL10405.Contig1_All, CL10643.Contig1_All, CL1067.Contig1_All, CL1067.Contig2_All, CL1067.Contig3_All, CL1067.Contig4_All, CL10734.Contig1_All, CL10844.Contig1_All, CL11.Contig14_All, CL11.Contig20_All, CL11.Contig4_All, CL11.Contig8_All, CL11013.Contig1_All, CL11063.Contig1_All, CL11100.Contig1_All, CL11274.Contig1_All, CL11344.Contig1_All, CL1182.Contig1_All, CL1182.Contig2_All, CL1182.Contig3_All, CL11827.Contig1_All, CL11856.Contig1_All, CL11913.Contig1_All, CL11975.Contig1_All, CL12051.Contig1_All, CL123.Contig2_All, CL123.Contig3_All, CL123.Contig4_All, CL123.Contig5_All, CL12428.Contig1_All, CL12451.Contig1_All, CL1269.Contig1_All, CL1269.Contig3_All, CL1269.Contig4_All, CL1269.Contig5_All, CL12914.Contig1_All, CL13160.Contig1_All, CL13210.Contig1_All, CL13286.Contig1_All, CL13420.Contig1_All, CL13524.Contig1_All, CL1365.Contig1_All, CL1365.Contig2_All, CL1365.Contig3_All, CL13855.Contig1_All, CL14348.Contig1_All, CL1448.Contig1_All, CL15068.Contig1_All, CL15355.Contig1_All, CL15380.Contig1_All, CL15423.Contig1_All, CL15610.Contig1_All, CL1577.Contig1_All, CL1577.Contig2_All, CL16.Contig10_All, CL16.Contig13_All, CL16.Contig21_All, CL16.Contig2_All, CL16.Contig3_All, CL16.Contig6_All, CL16054.Contig1_All, CL16114.Contig1_All, CL16452.Contig1_All, CL16476.Contig1_All, CL16809.Contig1_All, CL17152.Contig1_All, CL1727.Contig1_All, CL17379.Contig1_All, CL1806.Contig1_All,

	Circadian rhythm - plant	CL1808.Contig1_All, CL18165.Contig1_All, CL18241.Contig1_All, CL18994.Contig1_All, CL19097.Contig1_All, CL193.Contig5_All, CL20311.Contig1_All, CL2034.Contig1_All, CL20524.Contig1_All, CL20856.Contig1_All, CL21069.Contig1_All, CL21587.Contig1_All, CL21587.Contig2_All, CL21814.Contig1_All, CL2197.Contig1_All, CL22121.Contig1_All, CL22240.Contig1_All, CL2236.Contig1_All, CL2236.Contig2_All, CL22523.Contig1_All, CL22529.Contig1_All, CL226.Contig2_All, CL226.Contig4_All, CL22652.Contig1_All, CL2269.Contig1_All, CL2274.Contig1_All, CL2274.Contig2_All, CL2307.Contig1_All, CL23129.Contig1_All, CL23175.Contig1_All, CL23182.Contig1_All, CL2320.Contig1_All, CL2320.Contig2_All, CL24006.Contig1_All, CL24320.Contig1_All, CL2435.Contig1_All, CL24651.Contig1_All, CL24778.Contig1_All, CL25556.Contig1_All, CL26282.Contig1_All, CL2633.Contig1_All, CL2662.Contig1_All, CL26695.Contig1_All, CL26809.Contig1_All, CL27.Contig11_All, CL27.Contig1_All, CL2713.Contig1_All, CL27293.Contig1_All, CL27493.Contig1_All, CL2772.Contig1_All, CL27958.Contig1_All, CL2844.Contig1_All, CL2902.Contig1_All, CL3318.Contig1_All, CL3318.Contig2_All, CL3682.Contig1_All, CL3699.Contig3_All, CL3699.Contig4_All, CL3710.Contig1_All, CL3722.Contig1_All, CL3722.Contig2_All, CL3723.Contig1_All, CL3723.Contig2_All, CL4017.Contig1_All, CL4046.Contig1_All, CL4179.Contig1_All, CL4192.Contig1_All, CL4192.Contig2_All, CL451.Contig1_All, CL455.Contig1_All, CL455.Contig2_All, CL455.Contig3_All, CL455.Contig4_All, CL455.Contig5_All, CL455.Contig6_All, CL4556.Contig1_All, CL4581.Contig1_All, CL4625.Contig1_All, CL4625.Contig2_All, CL4728.Contig1_All, CL480.Contig1_All, CL480.Contig4_All, CL480.Contig5_All, CL4888.Contig1_All, CL5028.Contig1_All, CL5067.Contig1_All, CL5100.Contig1_All, CL5151.Contig1_All, CL5172.Contig1_All, CL52.Contig14_All, CL52.Contig16_All, CL52.Contig18_All, CL52.Contig2_All, CL52.Contig3_All, CL52.Contig5_All, CL52.Contig6_All, CL52.Contig9_All, CL5301.Contig1_All, CL5362.Contig1_All, CL5362.Contig2_All, CL5409.Contig1_All, CL5556.Contig1_All, CL557.Contig1_All, CL557.Contig2_All, CL557.Contig3_All, CL557.Contig4_All, CL5732.Contig1_All, CL5786.Contig1_All, CL6211.Contig1_All, CL6228.Contig1_All, CL6228.Contig2_All, CL6228.Contig3_All, CL6228.Contig4_All, CL6228.Contig5_All, CL6266.Contig1_All, CL6266.Contig2_All, CL6283.Contig1_All, CL6438.Contig1_All, CL6464.Contig1_All, CL6543.Contig1_All, CL6639.Contig1_All,

		CL6660.Contig1_All, CL6660.Contig3_All, CL7004.Contig1_All, CL7004.Contig2_All, CL7039.Contig1_All, CL7039.Contig2_All, CL7206.Contig1_All, CL7208.Contig1_All, CL7437.Contig1_All, CL7702.Contig1_All, CL8089.Contig1_All, CL809.Contig2_All, CL8152.Contig1_All, CL8455.Contig1_All, CL8455.Contig2_All, CL8575.Contig1_All, CL861.Contig5_All, CL9031.Contig1_All, CL915.Contig1_All, CL9324.Contig1_All, CL9683.Contig1_All, CL989.Contig1_All, CL989.Contig2_All, CL989.Contig3_All, Unigene1059_All, Unigene1221_All, Unigene1506_All, Unigene1805_All, Unigene200_All, Unigene2314_All, Unigene2577_All, Unigene2705_All, Unigene3069_All, Unigene3070_All, Unigene3353_All, Unigene3409_All, Unigene4435_All
24		CL10055.Contig1_All, CL10240.Contig1_All, CL10449.Contig1_All, CL10569.Contig1_All, CL1077.Contig1_All, CL10877.Contig1_All, CL11070.Contig1_All, CL11203.Contig1_All, CL11229.Contig1_All, CL11351.Contig1_All, CL11759.Contig1_All, CL12218.Contig1_All, CL12307.Contig1_All, CL12370.Contig1_All, CL12389.Contig1_All, CL12702.Contig1_All, CL12777.Contig1_All, CL12898.Contig1_All, CL13068.Contig1_All, CL13104.Contig1_All, CL13253.Contig1_All, CL13256.Contig1_All, CL13385.Contig1_All, CL13444.Contig1_All, CL13490.Contig1_All, CL1408.Contig1_All, CL1408.Contig2_All, CL1408.Contig3_All, CL1408.Contig4_All, CL14853.Contig1_All, CL15303.Contig1_All, CL1539.Contig1_All, CL1539.Contig2_All, CL15754.Contig1_All, CL16038.Contig1_All, CL16430.Contig1_All, CL16556.Contig1_All, CL16643.Contig1_All, CL16833.Contig1_All, CL17719.Contig1_All, CL17743.Contig1_All, CL17858.Contig1_All, CL17992.Contig1_All, CL18061.Contig1_All, CL18144.Contig1_All, CL18342.Contig1_All, CL18841.Contig1_All, CL18869.Contig1_All, CL18999.Contig1_All, CL19564.Contig1_All, CL20052.Contig1_All, CL20081.Contig1_All, CL20154.Contig1_All, CL20187.Contig1_All, CL20321.Contig1_All, CL20345.Contig1_All, CL20875.Contig1_All, CL20987.Contig1_All, CL2102.Contig1_All, CL21051.Contig1_All, CL21053.Contig1_All, CL21241.Contig1_All, CL22002.Contig1_All, CL22652.Contig1_All, CL22771.Contig1_All, CL23161.Contig1_All, CL23347.Contig1_All, CL23799.Contig1_All, CL2385.Contig1_All, CL23940.Contig1_All, CL2439.Contig1_All, CL2439.Contig2_All, CL24605.Contig1_All, CL25641.Contig1_All, CL25658.Contig1_All, CL25792.Contig1_All, CL25793.Contig1_All, CL25841.Contig1_All,

	Stilbenoid, diarylheptanoid and gingerol biosynthesis	CL26121.Contig1_All, CL26299.Contig1_All, CL266.Contig1_All, CL266.Contig2_All, CL266.Contig3_All, CL266.Contig4_All, CL266.Contig5_All, CL266.Contig6_All, CL26701.Contig1_All, CL26736.Contig1_All, CL26882.Contig1_All, CL26967.Contig1_All, CL27033.Contig1_All, CL27064.Contig1_All, CL27352.Contig1_All, CL27706.Contig1_All, CL2794.Contig1_All, CL2879.Contig1_All, CL3076.Contig1_All, CL3225.Contig1_All, CL3225.Contig3_All, CL3321.Contig1_All, CL3321.Contig2_All, CL3321.Contig3_All, CL3413.Contig1_All, CL3413.Contig2_All, CL3476.Contig1_All, CL3476.Contig2_All, CL3494.Contig1_All, CL3984.Contig1_All, CL4.Contig17_All, CL4.Contig25_All, CL4.Contig5_All, CL40.Contig10_All, CL40.Contig11_All, CL40.Contig1_All, CL40.Contig2_All, CL40.Contig3_All, CL40.Contig4_All, CL40.Contig5_All, CL40.Contig6_All, CL40.Contig7_All, CL40.Contig8_All, CL40.Contig9_All, CL4101.Contig1_All, CL4101.Contig2_All, CL4120.Contig1_All, CL4280.Contig1_All, CL4608.Contig1_All, CL476.Contig1_All, CL476.Contig2_All, CL476.Contig3_All, CL476.Contig4_All, CL476.Contig5_All, CL4842.Contig1_All, CL4963.Contig1_All, CL50.Contig10_All, CL50.Contig11_All, CL50.Contig12_All, CL50.Contig13_All, CL50.Contig14_All, CL50.Contig15_All, CL50.Contig16_All, CL50.Contig1_All, CL50.Contig2_All, CL50.Contig3_All, CL50.Contig4_All, CL50.Contig5_All, CL50.Contig6_All, CL50.Contig7_All, CL50.Contig8_All, CL50.Contig9_All, CL5092.Contig1_All, CL5183.Contig1_All, CL5183.Contig2_All, CL5183.Contig3_All, CL5215.Contig1_All, CL5230.Contig1_All, CL5264.Contig1_All, CL5264.Contig2_All, CL5402.Contig1_All, CL5463.Contig1_All, CL5755.Contig1_All, CL5829.Contig1_All, CL5843.Contig1_All, CL6013.Contig1_All, CL6089.Contig1_All, CL6141.Contig1_All, CL6141.Contig2_All, CL6466.Contig2_All, CL6479.Contig1_All, CL66.Contig4_All, CL702.Contig1_All, CL702.Contig2_All, CL702.Contig3_All, CL702.Contig4_All, CL7052.Contig1_All, CL7063.Contig1_All, CL7063.Contig2_All, CL7125.Contig1_All, CL7174.Contig1_All, CL7230.Contig1_All, CL7758.Contig1_All, CL832.Contig1_All, CL832.Contig2_All, CL8398.Contig1_All, CL8485.Contig1_All, CL8749.Contig1_All, CL8905.Contig1_All, CL8962.Contig1_All, CL9002.Contig1_All, CL9220.Contig1_All, CL9275.Contig1_All, CL9517.Contig1_All, CL9539.Contig1_All, CL9550.Contig1_All, Unigene1269_All, Unigene1543_All, Unigene1634_All, Unigene1694_All, Unigene2429_All, Unigene2466_All, Unigene2472_All, Unigene2646_All, Unigene2836_All, Unigene2961_All, Unigene2962_All, Unigene2963_All, Unigene2964_All, Unigene325_All, Unigene346_All, Unigene3504_All, Unigene3719_All,

		Unigene3792_All, Unigene462_All, Unigene4640_All, Unigene4676_All, Unigene4818_All, Unigene4987_All, Unigene5048_All, Unigene5117_All, Unigene5184_All, Unigene5207_All, Unigene5242_All, Unigene5316_All, Unigene5329_All, Unigene5438_All, Unigene644_All, Unigene71_All
25		CL10026.Contig1_All, CL10048.Contig1_All, CL10068.Contig1_All, CL10073.Contig1_All, CL10128.Contig1_All, CL10151.Contig1_All, CL10162.Contig1_All, CL10202.Contig1_All, CL10216.Contig1_All, CL10453.Contig1_All, CL10453.Contig2_All, CL10472.Contig1_All, CL10582.Contig1_All, CL10600.Contig1_All, CL10655.Contig1_All, CL10671.Contig1_All, CL10740.Contig1_All, CL10834.Contig1_All, CL10840.Contig1_All, CL10841.Contig1_All, CL10846.Contig1_All, CL10846.Contig2_All, CL10870.Contig1_All, CL10924.Contig1_All, CL11132.Contig1_All, CL11562.Contig1_All, CL11570.Contig1_All, CL11710.Contig1_All, CL11754.Contig1_All, CL11926.Contig1_All, CL12183.Contig1_All, CL12184.Contig1_All, CL12202.Contig1_All, CL1234.Contig1_All, CL1234.Contig2_All, CL12487.Contig1_All, CL12498.Contig1_All, CL12514.Contig1_All, CL12753.Contig1_All, CL12810.Contig1_All, CL12865.Contig1_All, CL12942.Contig1_All, CL13175.Contig1_All, CL13179.Contig1_All, CL13222.Contig1_All, CL13277.Contig1_All, CL13321.Contig1_All, CL1339.Contig1_All, CL1339.Contig2_All, CL1339.Contig3_All, CL1339.Contig4_All, CL13408.Contig1_All, CL13492.Contig1_All, CL13540.Contig1_All, CL13548.Contig1_All, CL13593.Contig1_All, CL13600.Contig1_All, CL13835.Contig1_All, CL139.Contig6_All, CL1412.Contig1_All, CL1412.Contig2_All, CL14669.Contig1_All, CL1502.Contig1_All, CL15447.Contig1_All, CL16239.Contig1_All, CL16268.Contig1_All, CL16543.Contig1_All, CL16555.Contig1_All, CL16675.Contig1_All, CL16719.Contig1_All, CL16871.Contig1_All, CL16913.Contig1_All, CL17189.Contig1_All, CL17291.Contig1_All, CL17291.Contig2_All, CL17597.Contig1_All, CL17597.Contig2_All, CL17689.Contig1_All, CL180.Contig2_All, CL180.Contig4_All, CL180.Contig7_All, CL18117.Contig1_All, CL1865.Contig1_All, CL19037.Contig1_All, CL19121.Contig1_All, CL19286.Contig1_All, CL19779.Contig1_All, CL2.Contig25_All, CL2005.Contig1_All, CL2041.Contig1_All, CL2041.Contig2_All, CL20832.Contig1_All, CL20874.Contig1_All, CL21049.Contig1_All, CL21511.Contig1_All, CL21565.Contig1_All, CL21692.Contig1_All, CL21741.Contig1_All, CL21780.Contig1_All,

	Glycolysis / Gluconeogenesis	CL21853.Contig1_All, CL21987.Contig1_All, CL22545.Contig1_All, CL22751.Contig1_All, CL22966.Contig1_All, CL23117.Contig1_All, CL23375.Contig1_All, CL23615.Contig1_All, CL23687.Contig1_All, CL24211.Contig1_All, CL250.Contig1_All, CL250.Contig2_All, CL250.Contig3_All, CL250.Contig4_All, CL250.Contig5_All, CL250.Contig6_All, CL25407.Contig1_All, CL25514.Contig1_All, CL26575.Contig1_All, CL26742.Contig1_All, CL27973.Contig1_All, CL28135.Contig1_All, CL2880.Contig1_All, CL2929.Contig1_All, CL2942.Contig2_All, CL2942.Contig3_All, CL3152.Contig1_All, CL3246.Contig1_All, CL3246.Contig2_All, CL3246.Contig3_All, CL3246.Contig4_All, CL3457.Contig1_All, CL3481.Contig1_All, CL3481.Contig2_All, CL3481.Contig3_All, CL353.Contig1_All, CL353.Contig2_All, CL353.Contig3_All, CL353.Contig4_All, CL353.Contig5_All, CL3532.Contig1_All, CL3532.Contig2_All, CL3532.Contig3_All, CL3803.Contig1_All, CL3915.Contig1_All, CL3942.Contig1_All, CL437.Contig1_All, CL437.Contig2_All, CL437.Contig3_All, CL437.Contig4_All, CL437.Contig5_All, CL437.Contig6_All, CL4477.Contig1_All, CL4477.Contig2_All, CL4549.Contig1_All, CL4568.Contig1_All, CL4632.Contig1_All, CL4632.Contig2_All, CL4932.Contig1_All, CL5224.Contig1_All, CL5333.Contig1_All, CL5478.Contig1_All, CL5516.Contig1_All, CL5549.Contig1_All, CL5549.Contig2_All, CL5583.Contig1_All, CL5793.Contig1_All, CL5864.Contig1_All, CL5864.Contig2_All, CL5968.Contig1_All, CL609.Contig1_All, CL609.Contig2_All, CL609.Contig3_All, CL609.Contig4_All, CL6371.Contig1_All, CL641.Contig1_All, CL641.Contig2_All, CL641.Contig3_All, CL6559.Contig1_All, CL6613.Contig1_All, CL6613.Contig2_All, CL6637.Contig1_All, CL6700.Contig1_All, CL6722.Contig1_All, CL7095.Contig1_All, CL7130.Contig1_All, CL7244.Contig1_All, CL7244.Contig2_All, CL7280.Contig1_All, CL729.Contig1_All, CL729.Contig2_All, CL729.Contig3_All, CL729.Contig4_All, CL7381.Contig1_All, CL7772.Contig1_All, CL8393.Contig1_All, CL8747.Contig1_All, CL9106.Contig1_All, CL928.Contig1_All, CL928.Contig2_All, Unigene1285_All, Unigene1434_All, Unigene1781_All, Unigene2140_All, Unigene2263_All, Unigene337_All, Unigene3654_All, Unigene3918_All, Unigene3983_All, Unigene4094_All, Unigene4184_All, Unigene4277_All, Unigene4340_All, Unigene4417_All, Unigene4438_All, Unigene4507_All, Unigene4688_All, Unigene4732_All, Unigene515_All, Unigene5261_All, Unigene5340_All, Unigene5473_All, Unigene5509_All, Unigene666_All, Unigene865_All
		CL10301.Contig1_All, CL10598.Contig1_All, CL106.Contig1_All, CL106.Contig2_All, CL106.Contig3_All,

26	Nucleotide excision repair	CL106.Contig4_All, CL106.Contig5_All, CL106.Contig6_All, CL10715.Contig1_All, CL10900.Contig1_All, CL11311.Contig1_All, CL11886.Contig1_All, CL12002.Contig1_All, CL12181.Contig1_All, CL12312.Contig1_All, CL12318.Contig1_All, CL12641.Contig1_All, CL12987.Contig1_All, CL13080.Contig1_All, CL13093.Contig1_All, CL13287.Contig1_All, CL13312.Contig1_All, CL1342.Contig1_All, CL1342.Contig2_All, CL1342.Contig3_All, CL1342.Contig4_All, CL14161.Contig1_All, CL14493.Contig1_All, CL1462.Contig1_All, CL14981.Contig1_All, CL15039.Contig1_All, CL15182.Contig1_All, CL15256.Contig1_All, CL15298.Contig1_All, CL15341.Contig1_All, CL15703.Contig1_All, CL16222.Contig1_All, CL16404.Contig1_All, CL16613.Contig1_All, CL16648.Contig1_All, CL16686.Contig1_All, CL17037.Contig1_All, CL17287.Contig1_All, CL17390.Contig1_All, CL17834.Contig1_All, CL18004.Contig1_All, CL18259.Contig1_All, CL18260.Contig1_All, CL1870.Contig2_All, CL18753.Contig1_All, CL18947.Contig1_All, CL18958.Contig1_All, CL19568.Contig1_All, CL19640.Contig1_All, CL19828.Contig1_All, CL19845.Contig1_All, CL19920.Contig1_All, CL20029.Contig1_All, CL20315.Contig1_All, CL20418.Contig1_All, CL20575.Contig1_All, CL20693.Contig1_All, CL20891.Contig1_All, CL20894.Contig1_All, CL20934.Contig1_All, CL2129.Contig2_All, CL21298.Contig1_All, CL21348.Contig1_All, CL21483.Contig1_All, CL21607.Contig1_All, CL21917.Contig1_All, CL22419.Contig1_All, CL2255.Contig1_All, CL22631.Contig1_All, CL22699.Contig1_All, CL2271.Contig1_All, CL2271.Contig2_All, CL2271.Contig3_All, CL22764.Contig1_All, CL23007.Contig1_All, CL23087.Contig1_All, CL2317.Contig1_All, CL23229.Contig1_All, CL23363.Contig1_All, CL23570.Contig1_All, CL23696.Contig1_All, CL23708.Contig1_All, CL23740.Contig1_All, CL23834.Contig1_All, CL24063.Contig1_All, CL24352.Contig1_All, CL24382.Contig1_All, CL24555.Contig1_All, CL24615.Contig1_All, CL24635.Contig1_All, CL2466.Contig1_All, CL24714.Contig1_All, CL24839.Contig1_All, CL25044.Contig1_All, CL2512.Contig1_All, CL25205.Contig1_All, CL25417.Contig1_All, CL25727.Contig1_All, CL25951.Contig1_All, CL26122.Contig1_All, CL26153.Contig1_All, CL26185.Contig1_All, CL26303.Contig1_All, CL2646.Contig1_All, CL26501.Contig1_All, CL26591.Contig1_All, CL2707.Contig1_All, CL27138.Contig1_All, CL27160.Contig1_All,

			CL27209.Contig1_All, CL27226.Contig1_All, CL27405.Contig1_All, CL27747.Contig1_All, CL27792.Contig1_All, CL2788.Contig1_All, CL27899.Contig1_All, CL27901.Contig1_All, CL2833.Contig1_All, CL316.Contig1_All, CL316.Contig2_All, CL316.Contig3_All, CL316.Contig4_All, CL3162.Contig1_All, CL3164.Contig1_All, CL3365.Contig1_All, CL3367.Contig1_All, CL3367.Contig2_All, CL3375.Contig1_All, CL3414.Contig1_All, CL3514.Contig2_All, CL4205.Contig1_All, CL4617.Contig1_All, CL5087.Contig1_All, CL5389.Contig1_All, CL564.Contig1_All, CL584.Contig1_All, CL6169.Contig1_All, CL6483.Contig1_All, CL6508.Contig1_All, CL6739.Contig1_All, CL6739.Contig2_All, CL6763.Contig1_All, CL6917.Contig1_All, CL6918.Contig1_All, CL7.Contig5_All, CL7319.Contig1_All, CL7412.Contig2_All, CL7433.Contig1_All, CL7610.Contig1_All, CL7610.Contig2_All, CL780.Contig1_All, CL780.Contig2_All, CL780.Contig3_All, CL7968.Contig1_All, CL8055.Contig1_All, CL8179.Contig1_All, CL8259.Contig1_All, CL8317.Contig1_All, CL8358.Contig1_All, CL8412.Contig1_All, CL8424.Contig1_All, CL8528.Contig1_All, CL8531.Contig1_All, CL8644.Contig1_All, CL8701.Contig1_All, CL8701.Contig2_All, CL8857.Contig2_All, CL8901.Contig2_All, CL9093.Contig1_All, CL9257.Contig1_All, CL9317.Contig1_All, CL94.Contig4_All, CL94.Contig7_All, CL9587.Contig1_All, CL9755.Contig1_All, CL9924.Contig1_All, CL9987.Contig1_All, Unigene1011_All, Unigene107_All, Unigene1386_All, Unigene1558_All, Unigene2075_All, Unigene2133_All, Unigene234_All, Unigene2408_All, Unigene2536_All, Unigene2585_All, Unigene2712_All, Unigene2864_All, Unigene2876_All, Unigene3204_All, Unigene3267_All, Unigene3275_All, Unigene3616_All, Unigene3789_All, Unigene4017_All, Unigene4038_All, Unigene4217_All, Unigene4286_All, Unigene4682_All, Unigene675_All, Unigene853_All
27			CL10240.Contig1_All, CL10449.Contig1_All, CL10569.Contig1_All, CL10727.Contig1_All, CL10740.Contig1_All, CL11070.Contig1_All, CL11203.Contig1_All, CL11229.Contig1_All, CL11759.Contig1_All, CL12067.Contig1_All, CL12136.Contig1_All, CL12307.Contig1_All, CL12389.Contig1_All, CL12498.Contig1_All, CL12777.Contig1_All, CL12898.Contig1_All, CL13023.Contig1_All, CL13104.Contig1_All, CL13253.Contig1_All, CL13256.Contig1_All, CL13385.Contig1_All, CL13444.Contig1_All, CL13490.Contig1_All, CL1408.Contig1_All, CL1408.Contig2_All, CL1408.Contig3_All, CL1408.Contig4_All, CL1412.Contig1_All, CL1412.Contig2_All, CL1418.Contig1_All, CL14294.Contig1_All, CL14364.Contig1_All,

	Limonene and pinene degradation	CL14853.Contig1_All, CL15303.Contig1_All, CL1539.Contig1_All, CL1539.Contig2_All, CL15754.Contig1_All, CL16038.Contig1_All, CL16239.Contig1_All, CL16430.Contig1_All, CL16556.Contig1_All, CL16833.Contig1_All, CL16871.Contig1_All, CL1749.Contig1_All, CL17743.Contig1_All, CL17858.Contig1_All, CL18061.Contig1_All, CL18144.Contig1_All, CL18342.Contig1_All, CL18999.Contig1_All, CL19564.Contig1_All, CL20052.Contig1_All, CL20081.Contig1_All, CL20187.Contig1_All, CL20321.Contig1_All, CL20345.Contig1_All, CL20875.Contig1_All, CL2102.Contig1_All, CL21051.Contig1_All, CL21053.Contig1_All, CL21241.Contig1_All, CL22002.Contig1_All, CL22771.Contig1_All, CL23161.Contig1_All, CL23185.Contig1_All, CL23347.Contig1_All, CL23687.Contig1_All, CL23799.Contig1_All, CL23940.Contig1_All, CL2439.Contig1_All, CL2439.Contig2_All, CL24605.Contig1_All, CL25641.Contig1_All, CL25658.Contig1_All, CL25792.Contig1_All, CL25793.Contig1_All, CL25841.Contig1_All, CL26121.Contig1_All, CL26299.Contig1_All, CL266.Contig1_All, CL266.Contig2_All, CL266.Contig3_All, CL266.Contig4_All, CL266.Contig5_All, CL266.Contig6_All, CL26701.Contig1_All, CL26882.Contig1_All, CL26967.Contig1_All, CL27352.Contig1_All, CL2755.Contig1_All, CL2755.Contig2_All, CL2755.Contig3_All, CL27706.Contig1_All, CL2794.Contig1_All, CL2929.Contig1_All, CL3457.Contig1_All, CL3494.Contig1_All, CL3545.Contig1_All, CL3984.Contig1_All, CL4.Contig17_All, CL4.Contig25_All, CL4.Contig5_All, CL40.Contig10_All, CL40.Contig11_All, CL40.Contig1_All, CL40.Contig2_All, CL40.Contig3_All, CL40.Contig4_All, CL40.Contig5_All, CL40.Contig6_All, CL40.Contig7_All, CL40.Contig8_All, CL40.Contig9_All, CL4101.Contig1_All, CL4101.Contig2_All, CL4608.Contig1_All, CL476.Contig1_All, CL476.Contig2_All, CL476.Contig3_All, CL476.Contig4_All, CL476.Contig5_All, CL4842.Contig1_All, CL50.Contig10_All, CL50.Contig11_All, CL50.Contig12_All, CL50.Contig13_All, CL50.Contig14_All, CL50.Contig15_All, CL50.Contig16_All, CL50.Contig1_All, CL50.Contig2_All, CL50.Contig3_All, CL50.Contig4_All, CL50.Contig5_All, CL50.Contig6_All, CL50.Contig7_All, CL50.Contig8_All, CL50.Contig9_All, CL5183.Contig1_All, CL5183.Contig2_All, CL5183.Contig3_All, CL5230.Contig1_All, CL5264.Contig1_All, CL5264.Contig2_All, CL5402.Contig1_All, CL5549.Contig1_All, CL5549.Contig2_All, CL5755.Contig1_All, CL5829.Contig1_All, CL5843.Contig1_All, CL5884.Contig1_All, CL6013.Contig1_All, CL6064.Contig1_All, CL6141.Contig1_All, CL6141.Contig2_All,

			CL6371.Contig1_All, CL6479.Contig1_All, CL66.Contig4_All, CL7052.Contig1_All, CL7063.Contig1_All, CL7063.Contig2_All, CL7125.Contig1_All, CL7174.Contig1_All, CL7230.Contig1_All, CL8398.Contig1_All, CL9002.Contig1_All, CL9106.Contig1_All, CL9220.Contig1_All, CL9275.Contig1_All, CL9429.Contig1_All, CL9517.Contig1_All, CL9550.Contig1_All, CL9868.Contig1_All, Unigene1269_All, Unigene1634_All, Unigene1694_All, Unigene2466_All, Unigene2472_All, Unigene2646_All, Unigene2836_All, Unigene2872_All, Unigene325_All, Unigene346_All, Unigene3503_All, Unigene3504_All, Unigene3719_All, Unigene3792_All, Unigene4417_All, Unigene462_All, Unigene4640_All, Unigene4676_All, Unigene4818_All, Unigene4987_All, Unigene5048_All, Unigene5085_All, Unigene5184_All, Unigene5207_All, Unigene5242_All, Unigene5316_All, Unigene5438_All, Unigene644_All, Unigene71_All, Unigene920_All
28			CL10057.Contig1_All, CL10088.Contig1_All, CL10167.Contig1_All, CL10305.Contig1_All, CL10494.Contig1_All, CL10530.Contig1_All, CL10541.Contig1_All, CL10547.Contig1_All, CL10687.Contig1_All, CL10814.Contig1_All, CL11024.Contig1_All, CL11234.Contig1_All, CL11328.Contig1_All, CL11330.Contig1_All, CL11434.Contig1_All, CL11869.Contig1_All, CL12067.Contig1_All, CL12173.Contig1_All, CL12238.Contig1_All, CL12277.Contig1_All, CL12555.Contig1_All, CL12674.Contig1_All, CL1282.Contig1_All, CL12822.Contig1_All, CL12868.Contig1_All, CL12948.Contig1_All, CL12981.Contig1_All, CL13034.Contig1_All, CL13073.Contig1_All, CL13574.Contig1_All, CL13862.Contig1_All, CL13923.Contig1_All, CL14042.Contig1_All, CL14042.Contig2_All, CL14284.Contig1_All, CL14294.Contig1_All, CL14330.Contig1_All, CL14490.Contig1_All, CL14659.Contig1_All, CL14677.Contig1_All, CL14754.Contig1_All, CL14931.Contig1_All, CL15127.Contig1_All, CL15301.Contig1_All, CL15451.Contig1_All, CL15468.Contig1_All, CL1558.Contig1_All, CL1558.Contig2_All, CL1559.Contig1_All, CL1559.Contig2_All, CL15629.Contig1_All, CL15676.Contig1_All, CL16640.Contig1_All, CL16795.Contig1_All, CL17109.Contig1_All, CL1728.Contig1_All, CL1728.Contig2_All, CL17699.Contig1_All, CL18360.Contig1_All, CL19200.Contig1_All, CL19567.Contig1_All, CL19689.Contig1_All, CL2083.Contig1_All, CL21134.Contig1_All, CL2115.Contig1_All, CL21372.Contig1_All, CL2146.Contig1_All, CL2146.Contig2_All, CL2146.Contig3_All, CL2225.Contig1_All,

	Peroxisome	CL2225.Contig2_All, CL22401.Contig1_All, CL22684.Contig1_All, CL23271.Contig1_All, CL23290.Contig1_All, CL233.Contig2_All, CL233.Contig3_All, CL233.Contig5_All, CL23420.Contig1_All, CL23423.Contig1_All, CL2381.Contig1_All, CL24030.Contig1_All, CL24508.Contig1_All, CL24617.Contig1_All, CL2462.Contig1_All, CL24912.Contig1_All, CL25435.Contig1_All, CL25486.Contig1_All, CL25537.Contig1_All, CL25778.Contig1_All, CL25786.Contig1_All, CL26096.Contig1_All, CL2670.Contig1_All, CL2697.Contig1_All, CL2697.Contig2_All, CL2745.Contig1_All, CL2745.Contig2_All, CL27926.Contig1_All, CL2932.Contig1_All, CL2936.Contig1_All, CL296.Contig3_All, CL3039.Contig1_All, CL3070.Contig2_All, CL3070.Contig3_All, CL3143.Contig1_All, CL3143.Contig2_All, CL3182.Contig1_All, CL3182.Contig2_All, CL32.Contig12_All, CL340.Contig1_All, CL3410.Contig1_All, CL3489.Contig1_All, CL3525.Contig1_All, CL3634.Contig1_All, CL3955.Contig1_All, CL4014.Contig1_All, CL402.Contig1_All, CL402.Contig3_All, CL4053.Contig1_All, CL4390.Contig1_All, CL4394.Contig1_All, CL4511.Contig1_All, CL4661.Contig1_All, CL4714.Contig1_All, CL4714.Contig2_All, CL486.Contig1_All, CL486.Contig2_All, CL486.Contig3_All, CL486.Contig4_All, CL5081.Contig1_All, CL53.Contig10_All, CL53.Contig11_All, CL53.Contig1_All, CL53.Contig3_All, CL53.Contig6_All, CL53.Contig7_All, CL53.Contig8_All, CL53.Contig9_All, CL5691.Contig1_All, CL5691.Contig2_All, CL5744.Contig1_All, CL5819.Contig1_All, CL5884.Contig1_All, CL5971.Contig1_All, CL6091.Contig1_All, CL656.Contig1_All, CL656.Contig2_All, CL656.Contig3_All, CL656.Contig4_All, CL7398.Contig1_All, CL7401.Contig1_All, CL7476.Contig1_All, CL7670.Contig1_All, CL7978.Contig1_All, CL8052.Contig1_All, CL8438.Contig1_All, CL8496.Contig1_All, CL8651.Contig1_All, CL8935.Contig1_All, CL9174.Contig1_All, CL9214.Contig1_All, CL9396.Contig1_All, CL9396.Contig2_All, CL984.Contig1_All, Unigene1219_All, Unigene1245_All, Unigene1434_All, Unigene1504_All, Unigene1717_All, Unigene1718_All, Unigene2308_All, Unigene2602_All, Unigene2718_All, Unigene2953_All, Unigene2954_All, Unigene2955_All, Unigene2956_All, Unigene2970_All, Unigene3102_All, Unigene3208_All, Unigene327_All, Unigene363_All, Unigene4058_All, Unigene4109_All, Unigene4114_All, Unigene4409_All, Unigene4528_All, Unigene4745_All, Unigene4931_All, Unigene5089_All, Unigene5243_All, Unigene964_All
		CL10008.Contig1_All, CL10038.Contig1_All, CL10055.Contig1_All, CL10753.Contig1_All, CL1077.Contig1_All, CL10825.Contig1_All, CL10877.Contig1_All, CL11013.Contig1_All,

29	Flavonoid biosynthesis	CL11247.Contig1_All, CL11351.Contig1_All, CL11827.Contig1_All, CL12207.Contig1_All, CL12218.Contig1_All, CL12370.Contig1_All, CL1246.Contig1_All, CL12470.Contig1_All, CL1269.Contig1_All, CL1269.Contig3_All, CL1269.Contig4_All, CL1269.Contig5_All, CL12702.Contig1_All, CL1277.Contig2_All, CL13068.Contig1_All, CL13286.Contig1_All, CL13444.Contig1_All, CL13524.Contig1_All, CL14802.Contig1_All, CL15754.Contig1_All, CL1591.Contig1_All, CL1591.Contig2_All, CL1591.Contig3_All, CL1591.Contig4_All, CL1591.Contig5_All, CL16000.Contig1_All, CL16054.Contig1_All, CL16274.Contig1_All, CL16430.Contig1_All, CL16603.Contig1_All, CL16605.Contig1_All, CL16643.Contig1_All, CL17261.Contig1_All, CL17379.Contig1_All, CL17419.Contig1_All, CL17441.Contig1_All, CL17466.Contig1_All, CL17667.Contig1_All, CL17684.Contig1_All, CL17693.Contig1_All, CL17719.Contig1_All, CL17858.Contig1_All, CL17992.Contig1_All, CL18342.Contig1_All, CL18841.Contig1_All, CL18869.Contig1_All, CL19553.Contig1_All, CL19564.Contig1_All, CL19616.Contig1_All, CL20016.Contig1_All, CL20154.Contig1_All, CL20859.Contig1_All, CL20987.Contig1_All, CL21022.Contig1_All, CL21921.Contig1_All, CL2205.Contig1_All, CL22603.Contig1_All, CL22652.Contig1_All, CL22771.Contig1_All, CL23127.Contig1_All, CL23275.Contig1_All, CL23475.Contig1_All, CL2385.Contig1_All, CL2490.Contig1_All, CL25456.Contig1_All, CL25556.Contig1_All, CL25567.Contig1_All, CL25658.Contig1_All, CL25793.Contig1_All, CL26496.Contig1_All, CL26736.Contig1_All, CL26967.Contig1_All, CL27033.Contig1_All, CL27064.Contig1_All, CL27352.Contig1_All, CL2879.Contig1_All, CL2902.Contig1_All, CL3076.Contig1_All, CL3225.Contig1_All, CL3225.Contig3_All, CL3307.Contig1_All, CL3321.Contig1_All, CL3321.Contig2_All, CL3321.Contig3_All, CL3413.Contig1_All, CL3413.Contig2_All, CL3473.Contig1_All, CL3476.Contig1_All, CL3476.Contig2_All, CL3590.Contig1_All, CL3838.Contig1_All, CL3838.Contig2_All, CL40.Contig10_All, CL40.Contig11_All, CL40.Contig2_All, CL40.Contig4_All, CL40.Contig5_All, CL40.Contig6_All, CL40.Contig7_All, CL40.Contig8_All, CL4120.Contig1_All, CL4224.Contig1_All, CL4280.Contig1_All, CL4445.Contig1_All, CL447.Contig1_All, CL447.Contig3_All, CL447.Contig4_All, CL447.Contig5_All, CL447.Contig6_All, CL4842.Contig1_All, CL4963.Contig1_All, CL4974.Contig1_All, CL50.Contig10_All, CL50.Contig12_All, CL50.Contig13_All, CL50.Contig14_All, CL50.Contig15_All,

		CL50.Contig16_All, CL50.Contig2_All, CL50.Contig3_All, CL50.Contig4_All, CL50.Contig5_All, CL50.Contig8_All, CL50.Contig9_All, CL5020.Contig1_All, CL5020.Contig2_All, CL5092.Contig1_All, CL5100.Contig1_All, CL5215.Contig1_All, CL5337.Contig1_All, CL5463.Contig1_All, CL5637.Contig1_All, CL5637.Contig2_All, CL6025.Contig1_All, CL6089.Contig1_All, CL6466.Contig2_All, CL6914.Contig1_All, CL6929.Contig1_All, CL700.Contig1_All, CL700.Contig2_All, CL700.Contig3_All, CL700.Contig4_All, CL700.Contig5_All, CL702.Contig1_All, CL702.Contig2_All, CL702.Contig3_All, CL702.Contig4_All, CL7257.Contig1_All, CL7621.Contig1_All, CL7758.Contig1_All, CL832.Contig1_All, CL832.Contig2_All, CL8485.Contig1_All, CL8546.Contig1_All, CL8624.Contig1_All, CL8689.Contig1_All, CL8749.Contig1_All, CL8873.Contig1_All, CL8905.Contig1_All, CL8962.Contig1_All, CL9435.Contig1_All, CL9539.Contig1_All, Unigene1419_All, Unigene1543_All, Unigene1564_All, Unigene1634_All, Unigene1694_All, Unigene2429_All, Unigene2961_All, Unigene2962_All, Unigene2963_All, Unigene2964_All, Unigene3707_All, Unigene4219_All, Unigene4370_All, Unigene4876_All, Unigene5117_All, Unigene5292_All, Unigene5329_All, Unigene828_All, Unigene895_All
30		CL1.Contig111_All, CL1.Contig144_All, CL1.Contig24_All, CL1.Contig31_All, CL1.Contig42_All, CL1.Contig48_All, CL1.Contig66_All, CL1.Contig77_All, CL1.Contig82_All, CL1.Contig96_All, CL1.Contig98_All, CL10338.Contig1_All, CL10774.Contig1_All, CL11311.Contig1_All, CL1136.Contig1_All, CL11616.Contig1_All, CL11862.Contig1_All, CL11886.Contig1_All, CL12002.Contig1_All, CL1225.Contig1_All, CL1225.Contig3_All, CL12794.Contig1_All, CL12829.Contig1_All, CL13297.Contig2_All, CL13495.Contig1_All, CL13495.Contig2_All, CL13513.Contig1_All, CL14415.Contig1_All, CL14569.Contig1_All, CL1462.Contig1_All, CL14739.Contig1_All, CL14810.Contig1_All, CL15.Contig15_All, CL15.Contig7_All, CL15.Contig9_All, CL151.Contig6_All, CL15138.Contig1_All, CL15182.Contig1_All, CL15298.Contig1_All, CL15449.Contig1_All, CL15583.Contig1_All, CL15736.Contig1_All, CL1636.Contig1_All, CL1661.Contig1_All, CL16613.Contig1_All, CL16738.Contig1_All, CL169.Contig1_All, CL169.Contig4_All, CL169.Contig5_All, CL169.Contig8_All, CL16930.Contig1_All, CL17290.Contig1_All, CL17390.Contig1_All, CL1786.Contig2_All, CL17971.Contig1_All, CL1813.Contig1_All, CL18260.Contig1_All, CL18309.Contig1_All, CL18562.Contig1_All, CL18605.Contig1_All, CL18947.Contig1_All, CL19094.Contig1_All, CL1910.Contig2_All, CL19109.Contig1_All, CL19118.Contig1_All, CL19568.Contig1_All, CL19640.Contig1_All,

	Basal transcription factors	CL20.Contig1_All, CL20.Contig9_All, CL20259.Contig1_All, CL20315.Contig1_All, CL20575.Contig1_All, CL21002.Contig1_All, CL2105.Contig1_All, CL21083.Contig1_All, CL21156.Contig1_All, CL21282.Contig1_All, CL21560.Contig1_All, CL22462.Contig1_All, CL22467.Contig1_All, CL22631.Contig1_All, CL22699.Contig1_All, CL233.Contig1_All, CL23508.Contig1_All, CL2360.Contig1_All, CL24382.Contig1_All, CL24555.Contig1_All, CL24637.Contig1_All, CL2512.Contig1_All, CL25205.Contig1_All, CL25512.Contig1_All, CL26674.Contig1_All, CL27.Contig2_All, CL27.Contig5_All, CL27.Contig6_All, CL270.Contig1_All, CL27104.Contig1_All, CL27226.Contig1_All, CL27660.Contig1_All, CL27747.Contig1_All, CL27792.Contig1_All, CL2782.Contig1_All, CL27899.Contig1_All, CL2855.Contig1_All, CL2855.Contig2_All, CL2855.Contig3_All, CL2860.Contig3_All, CL295.Contig1_All, CL3365.Contig1_All, CL348.Contig1_All, CL3705.Contig1_All, CL3705.Contig2_All, CL38.Contig11_All, CL38.Contig12_All, CL38.Contig1_All, CL38.Contig3_All, CL38.Contig5_All, CL38.Contig7_All, CL38.Contig9_All, CL3929.Contig1_All, CL4136.Contig1_All, CL4136.Contig2_All, CL414.Contig2_All, CL4267.Contig2_All, CL4274.Contig1_All, CL4513.Contig1_All, CL4524.Contig1_All, CL4740.Contig2_All, CL5013.Contig2_All, CL5273.Contig1_All, CL5354.Contig1_All, CL5389.Contig1_All, CL5437.Contig1_All, CL5601.Contig1_All, CL5601.Contig2_All, CL5601.Contig3_All, CL5607.Contig2_All, CL5790.Contig1_All, CL5790.Contig2_All, CL5790.Contig3_All, CL5790.Contig4_All, CL611.Contig2_All, CL6187.Contig1_All, CL6307.Contig1_All, CL6372.Contig1_All, CL6437.Contig1_All, CL6442.Contig1_All, CL6512.Contig1_All, CL6739.Contig1_All, CL6739.Contig2_All, CL675.Contig1_All, CL675.Contig3_All, CL7734.Contig1_All, CL786.Contig1_All, CL7860.Contig1_All, CL8091.Contig1_All, CL8424.Contig1_All, CL8547.Contig2_All, CL8635.Contig1_All, CL8701.Contig1_All, CL8701.Contig2_All, CL89.Contig1_All, CL89.Contig5_All, CL89.Contig6_All, CL8901.Contig2_All, CL8918.Contig1_All, CL9007.Contig1_All, CL9305.Contig1_All, CL9519.Contig1_All, CL9781.Contig1_All, CL9860.Contig1_All, Unigene1558_All, Unigene1739_All, Unigene2536_All, Unigene2652_All, Unigene2656_All, Unigene2712_All, Unigene3291_All, Unigene359_All, Unigene4103_All, Unigene4369_All, Unigene4623_All, Unigene4920_All, Unigene5361_All, Unigene643_All
		CL10136.Contig1_All, CL10163.Contig1_All, CL10230.Contig1_All, CL10238.Contig1_All, CL10469.Contig1_All, CL10490.Contig1_All, CL10507.Contig1_All, CL10568.Contig1_All,

31	[Cysteine and methionine metabolism]	CL10623.Contig1_All, CL10672.Contig1_All, CL10753.Contig1_All, CL10841.Contig1_All, CL10886.Contig1_All, CL10994.Contig1_All, CL11111.Contig1_All, CL11262.Contig1_All, CL11468.Contig1_All, CL11527.Contig1_All, CL11669.Contig1_All, CL11811.Contig1_All, CL11909.Contig1_All, CL12050.Contig1_All, CL12133.Contig1_All, CL12185.Contig1_All, CL12293.Contig1_All, CL12720.Contig1_All, CL12750.Contig1_All, CL12945.Contig1_All, CL12945.Contig2_All, CL13083.Contig1_All, CL13232.Contig1_All, CL13451.Contig1_All, CL13506.Contig1_All, CL13560.Contig1_All, CL13634.Contig1_All, CL13721.Contig1_All, CL13884.Contig1_All, CL14045.Contig1_All, CL14098.Contig1_All, CL14269.Contig1_All, CL14753.Contig1_All, CL14922.Contig1_All, CL15029.Contig1_All, CL15147.Contig1_All, CL15169.Contig1_All, CL15356.Contig1_All, CL15517.Contig1_All, CL15635.Contig1_All, CL16043.Contig1_All, CL16051.Contig1_All, CL16291.Contig1_All, CL16751.Contig1_All, CL16787.Contig1_All, CL16932.Contig1_All, CL17356.Contig1_All, CL17419.Contig1_All, CL17459.Contig1_All, CL17459.Contig2_All, CL17574.Contig1_All, CL17581.Contig1_All, CL17693.Contig1_All, CL18109.Contig1_All, CL18159.Contig1_All, CL18289.Contig1_All, CL18529.Contig1_All, CL18780.Contig1_All, CL1882.Contig1_All, CL1882.Contig2_All, CL1882.Contig3_All, CL1882.Contig4_All, CL1882.Contig5_All, CL1882.Contig6_All, CL1958.Contig2_All, CL1995.Contig1_All, CL1995.Contig2_All, CL1995.Contig3_All, CL2118.Contig2_All, CL21259.Contig1_All, CL21280.Contig1_All, CL21372.Contig1_All, CL2143.Contig1_All, CL2164.Contig1_All, CL21674.Contig1_All, CL2221.Contig1_All, CL2221.Contig2_All, CL22366.Contig1_All, CL22411.Contig1_All, CL22601.Contig1_All, CL23294.Contig1_All, CL23320.Contig1_All, CL23435.Contig1_All, CL23614.Contig1_All, CL2367.Contig1_All, CL2367.Contig2_All, CL2367.Contig3_All, CL23781.Contig1_All, CL24023.Contig1_All, CL24088.Contig1_All, CL24579.Contig1_All, CL2554.Contig1_All, CL25675.Contig1_All, CL25992.Contig1_All, CL26021.Contig1_All, CL2621.Contig1_All, CL2621.Contig2_All, CL26496.Contig1_All, CL26948.Contig1_All, CL26958.Contig1_All, CL27264.Contig1_All, CL27895.Contig1_All, CL2876.Contig1_All, CL3041.Contig1_All, CL3916.Contig1_All, CL3916.Contig2_All, CL4055.Contig1_All, CL4099.Contig1_All,

		CL4347.Contig1_All, CL4347.Contig2_All, CL4347.Contig3_All, CL4347.Contig4_All, CL4432.Contig1_All, CL4445.Contig1_All, CL4450.Contig1_All, CL447.Contig1_All, CL447.Contig2_All, CL4675.Contig1_All, CL5017.Contig1_All, CL504.Contig1_All, CL504.Contig2_All, CL504.Contig3_All, CL504.Contig4_All, CL504.Contig5_All, CL5179.Contig1_All, CL5179.Contig2_All, CL5337.Contig1_All, CL5662.Contig1_All, CL5680.Contig1_All, CL5787.Contig1_All, CL587.Contig1_All, CL587.Contig3_All, CL587.Contig4_All, CL587.Contig5_All, CL587.Contig6_All, CL6395.Contig1_All, CL6407.Contig1_All, CL6579.Contig1_All, CL6640.Contig1_All, CL6865.Contig1_All, CL6871.Contig1_All, CL6889.Contig1_All, CL7800.Contig1_All, CL8200.Contig1_All, CL8297.Contig1_All, CL8386.Contig1_All, CL856.Contig1_All, CL8651.Contig1_All, CL8804.Contig1_All, CL8894.Contig1_All, CL8964.Contig1_All, CL9578.Contig1_All, CL9657.Contig1_All, Unigene1045_All, Unigene1083_All, Unigene1142_All, Unigene2291_All, Unigene2486_All, Unigene2600_All, Unigene2884_All, Unigene3071_All, Unigene3120_All, Unigene3426_All, Unigene4348_All, Unigene4352_All, Unigene4469_All, Unigene4646_All, Unigene4755_All, Unigene4811_All, Unigene4943_All, Unigene5120_All, Unigene5364_All
32		CL1.Contig128_All, CL10064.Contig1_All, CL10071.Contig1_All, CL10684.Contig1_All, CL10743.Contig1_All, CL10881.Contig1_All, CL11092.Contig1_All, CL11408.Contig1_All, CL11490.Contig1_All, CL11655.Contig1_All, CL1214.Contig1_All, CL12645.Contig1_All, CL1312.Contig1_All, CL13220.Contig1_All, CL13588.Contig1_All, CL13917.Contig1_All, CL13947.Contig1_All, CL14054.Contig1_All, CL14136.Contig1_All, CL14471.Contig1_All, CL14777.Contig1_All, CL14855.Contig1_All, CL1487.Contig1_All, CL15140.Contig1_All, CL1531.Contig1_All, CL15918.Contig1_All, CL16182.Contig1_All, CL16492.Contig1_All, CL17401.Contig1_All, CL17705.Contig1_All, CL17732.Contig1_All, CL18351.Contig1_All, CL1871.Contig1_All, CL18732.Contig1_All, CL19075.Contig1_All, CL19319.Contig1_All, CL19523.Contig1_All, CL1976.Contig1_All, CL1978.Contig1_All, CL19884.Contig1_All, CL20024.Contig1_All, CL20167.Contig1_All, CL20313.Contig1_All, CL20514.Contig1_All, CL20669.Contig1_All, CL2079.Contig1_All, CL20796.Contig1_All, CL2096.Contig1_All, CL2116.Contig1_All, CL21476.Contig1_All, CL21575.Contig1_All, CL21718.Contig1_All, CL21788.Contig1_All, CL22116.Contig1_All, CL22276.Contig1_All, CL22822.Contig1_All, CL2320.Contig1_All, CL2320.Contig2_All,

	[Phosphatidylinositol signaling system]()	CL2341.Contig1_All, CL23631.Contig1_All, CL24400.Contig1_All, CL2456.Contig1_All, CL2460.Contig1_All, CL2460.Contig2_All, CL25168.Contig1_All, CL25277.Contig1_All, CL2537.Contig1_All, CL25581.Contig1_All, CL2582.Contig1_All, CL26225.Contig1_All, CL26584.Contig1_All, CL26876.Contig1_All, CL26888.Contig1_All, CL27054.Contig1_All, CL27069.Contig1_All, CL27290.Contig1_All, CL27482.Contig1_All, CL27520.Contig1_All, CL27592.Contig1_All, CL27744.Contig1_All, CL27788.Contig1_All, CL28116.Contig1_All, CL3014.Contig1_All, CL3165.Contig1_All, CL3165.Contig2_All, CL3165.Contig3_All, CL3264.Contig1_All, CL3264.Contig2_All, CL3572.Contig1_All, CL3572.Contig2_All, CL3723.Contig1_All, CL3723.Contig2_All, CL3884.Contig1_All, CL3922.Contig1_All, CL3943.Contig1_All, CL4098.Contig1_All, CL4098.Contig2_All, CL4123.Contig1_All, CL4125.Contig1_All, CL4181.Contig1_All, CL4181.Contig2_All, CL4305.Contig1_All, CL4342.Contig1_All, CL4509.Contig1_All, CL456.Contig1_All, CL456.Contig2_All, CL456.Contig3_All, CL456.Contig4_All, CL456.Contig5_All, CL456.Contig6_All, CL4570.Contig1_All, CL4578.Contig1_All, CL4674.Contig1_All, CL4858.Contig1_All, CL4968.Contig1_All, CL5236.Contig1_All, CL5253.Contig1_All, CL5393.Contig1_All, CL5450.Contig1_All, CL5494.Contig1_All, CL557.Contig1_All, CL557.Contig2_All, CL557.Contig3_All, CL557.Contig4_All, CL5703.Contig1_All, CL5745.Contig1_All, CL5937.Contig1_All, CL6377.Contig1_All, CL6522.Contig1_All, CL674.Contig1_All, CL674.Contig2_All, CL674.Contig3_All, CL674.Contig4_All, CL674.Contig5_All, CL6842.Contig1_All, CL7.Contig25_All, CL7072.Contig1_All, CL7383.Contig1_All, CL7518.Contig1_All, CL7627.Contig1_All, CL764.Contig1_All, CL7740.Contig1_All, CL7850.Contig1_All, CL8133.Contig1_All, CL8600.Contig1_All, CL8739.Contig1_All, CL9054.Contig1_All, CL9165.Contig1_All, CL917.Contig1_All, CL917.Contig2_All, CL917.Contig3_All, CL9204.Contig1_All, CL9981.Contig1_All, Unigene1020_All, Unigene1104_All, Unigene1135_All, Unigene1697_All, Unigene1900_All, Unigene1987_All, Unigene1990_All, Unigene2094_All, Unigene2218_All, Unigene2628_All, Unigene3138_All, Unigene3563_All, Unigene388_All, Unigene3980_All, Unigene4548_All, Unigene4558_All, Unigene5302_All, Unigene5373_All, Unigene5505_All, Unigene930_All, Unigene966_All, Unigene975_All
		CL10048.Contig1_All, CL10073.Contig1_All, CL10162.Contig1_All, CL10202.Contig1_All, CL10266.Contig1_All, CL10363.Contig1_All, CL10551.Contig1_All, CL10600.Contig1_All,

33	Pyruvate metabolism	CL10655.Contig1_All, CL10740.Contig1_All, CL10841.Contig1_All, CL11421.Contig1_All, CL11480.Contig1_All, CL11562.Contig1_All, CL11570.Contig1_All, CL11754.Contig1_All, CL11878.Contig1_All, CL11966.Contig1_All, CL12091.Contig1_All, CL12126.Contig1_All, CL12226.Contig1_All, CL12301.Contig1_All, CL1234.Contig1_All, CL1234.Contig2_All, CL12429.Contig1_All, CL12466.Contig1_All, CL12487.Contig1_All, CL12498.Contig1_All, CL12514.Contig1_All, CL12523.Contig1_All, CL12753.Contig1_All, CL12852.Contig1_All, CL12865.Contig1_All, CL12909.Contig1_All, CL12957.Contig1_All, CL13019.Contig1_All, CL13277.Contig1_All, CL1339.Contig1_All, CL1339.Contig2_All, CL1339.Contig3_All, CL1339.Contig4_All, CL13408.Contig1_All, CL13926.Contig1_All, CL1412.Contig1_All, CL1412.Contig2_All, CL14254.Contig1_All, CL14282.Contig1_All, CL14439.Contig1_All, CL14586.Contig1_All, CL147.Contig1_All, CL150.Contig7_All, CL1502.Contig1_All, CL15128.Contig1_All, CL15128.Contig2_All, CL15778.Contig1_All, CL1614.Contig1_All, CL1614.Contig2_All, CL1615.Contig1_All, CL16239.Contig1_All, CL16543.Contig1_All, CL16719.Contig1_All, CL16871.Contig1_All, CL1689.Contig1_All, CL16982.Contig1_All, CL17189.Contig1_All, CL17689.Contig1_All, CL17949.Contig1_All, CL18005.Contig1_All, CL18116.Contig1_All, CL1865.Contig1_All, CL19205.Contig1_All, CL2041.Contig1_All, CL2041.Contig2_All, CL2138.Contig1_All, CL23.Contig11_All, CL23.Contig12_All, CL23.Contig15_All, CL23.Contig16_All, CL23.Contig6_All, CL23272.Contig1_All, CL23375.Contig1_All, CL23687.Contig1_All, CL25053.Contig1_All, CL25269.Contig1_All, CL26742.Contig1_All, CL2762.Contig1_All, CL288.Contig1_All, CL288.Contig2_All, CL288.Contig6_All, CL288.Contig7_All, CL2929.Contig1_All, CL3152.Contig1_All, CL3342.Contig1_All, CL3346.Contig1_All, CL3346.Contig2_All, CL3448.Contig1_All, CL3448.Contig2_All, CL3457.Contig1_All, CL395.Contig2_All, CL395.Contig3_All, CL395.Contig5_All, CL4156.Contig1_All, CL4156.Contig2_All, CL4477.Contig1_All, CL4477.Contig2_All, CL4560.Contig1_All, CL4666.Contig1_All, CL5224.Contig1_All, CL5251.Contig1_All, CL5478.Contig1_All, CL5549.Contig1_All, CL5549.Contig2_All, CL5769.Contig1_All, CL5793.Contig1_All, CL5968.Contig1_All, CL6305.Contig1_All, CL6371.Contig1_All, CL641.Contig1_All, CL641.Contig2_All, CL641.Contig3_All, CL6565.Contig1_All, CL6722.Contig1_All, CL729.Contig1_All, CL729.Contig2_All, CL729.Contig3_All, CL729.Contig4_All, CL7443.Contig1_All,

		CL7443.Contig2_All, CL7537.Contig1_All, CL7537.Contig2_All, CL7537.Contig3_All, CL7891.Contig1_All, CL7927.Contig1_All, CL8138.Contig1_All, CL8366.Contig1_All, CL8718.Contig1_All, CL8747.Contig1_All, CL8831.Contig1_All, CL8891.Contig1_All, CL8959.Contig1_All, CL9106.Contig1_All, CL928.Contig1_All, CL928.Contig2_All, CL9846.Contig1_All, CL9950.Contig1_All, Unigene1242_All, Unigene1285_All, Unigene205_All, Unigene2140_All, Unigene3654_All, Unigene3666_All, Unigene3997_All, Unigene4277_All, Unigene4417_All, Unigene4507_All, Unigene4527_All, Unigene5261_All, Unigene5389_All, Unigene5473_All, Unigene5496_All, Unigene5509_All, Unigene666_All
34	Homologous recombination	CL10045.Contig1_All, CL105.Contig10_All, CL106.Contig1_All, CL106.Contig2_All, CL106.Contig3_All, CL106.Contig4_All, CL106.Contig5_All, CL106.Contig6_All, CL10900.Contig1_All, CL12181.Contig1_All, CL1236.Contig3_All, CL12987.Contig1_All, CL13029.Contig1_All, CL13236.Contig1_All, CL1334.Contig1_All, CL1334.Contig2_All, CL14493.Contig1_All, CL15256.Contig1_All, CL1538.Contig1_All, CL15603.Contig1_All, CL16039.Contig1_All, CL16241.Contig1_All, CL16566.Contig1_All, CL16686.Contig1_All, CL1685.Contig1_All, CL16850.Contig1_All, CL16893.Contig1_All, CL17342.Contig1_All, CL17545.Contig1_All, CL1777.Contig1_All, CL17909.Contig1_All, CL18091.Contig1_All, CL18259.Contig1_All, CL1870.Contig2_All, CL18819.Contig1_All, CL18829.Contig1_All, CL19123.Contig1_All, CL19194.Contig1_All, CL19388.Contig1_All, CL19695.Contig1_All, CL19713.Contig1_All, CL19799.Contig1_All, CL19828.Contig1_All, CL19963.Contig1_All, CL20045.Contig1_All, CL20073.Contig1_All, CL20326.Contig1_All, CL20507.Contig1_All, CL20530.Contig1_All, CL20899.Contig1_All, CL20934.Contig1_All, CL21122.Contig1_All, CL2120.Contig1_All, CL2315.Contig1_All, CL2315.Contig2_All, CL2315.Contig3_All, CL23708.Contig1_All, CL23795.Contig1_All, CL23843.Contig1_All, CL23903.Contig1_All, CL23983.Contig1_All, CL24441.Contig1_All, CL24615.Contig1_All, CL24695.Contig1_All, CL25044.Contig1_All, CL25291.Contig1_All, CL25374.Contig1_All, CL25417.Contig1_All, CL25493.Contig1_All, CL25886.Contig1_All, CL25907.Contig1_All, CL26258.Contig1_All, CL26319.Contig1_All, CL26348.Contig1_All, CL26537.Contig1_All, CL26594.Contig1_All, CL26819.Contig1_All, CL26877.Contig1_All, CL27145.Contig1_All, CL27160.Contig1_All, CL2735.Contig1_All, CL27375.Contig1_All,

		CL27463.Contig1_All, CL27983.Contig1_All, CL2814.Contig2_All, CL3023.Contig1_All, CL3511.Contig1_All, CL3895.Contig1_All, CL3929.Contig1_All, CL4114.Contig1_All, CL4114.Contig2_All, CL4205.Contig1_All, CL4465.Contig1_All, CL449.Contig3_All, CL4540.Contig1_All, CL5037.Contig1_All, CL5054.Contig1_All, CL5344.Contig1_All, CL5445.Contig1_All, CL5488.Contig1_All, CL5818.Contig1_All, CL588.Contig1_All, CL588.Contig3_All, CL588.Contig4_All, CL5912.Contig1_All, CL6169.Contig1_All, CL642.Contig1_All, CL6508.Contig1_All, CL6544.Contig1_All, CL6633.Contig1_All, CL6880.Contig1_All, CL6886.Contig1_All, CL6917.Contig1_All, CL6945.Contig1_All, CL7028.Contig1_All, CL716.Contig1_All, CL7433.Contig1_All, CL7510.Contig1_All, CL7610.Contig1_All, CL7610.Contig2_All, CL792.Contig1_All, CL792.Contig2_All, CL792.Contig3_All, CL792.Contig4_All, CL792.Contig5_All, CL8057.Contig1_All, CL8179.Contig1_All, CL8251.Contig1_All, CL8275.Contig1_All, CL8425.Contig1_All, CL8644.Contig1_All, CL8778.Contig1_All, CL9247.Contig1_All, CL93.Contig2_All, CL9332.Contig1_All, CL9332.Contig2_All, CL9755.Contig1_All, CL98.Contig1_All, CL98.Contig3_All, CL98.Contig4_All, CL98.Contig5_All, CL98.Contig6_All, CL9987.Contig1_All, Unigene1386_All, Unigene1899_All, Unigene1983_All, Unigene2186_All, Unigene2408_All, Unigene2579_All, Unigene2622_All, Unigene2732_All, Unigene2814_All, Unigene2876_All, Unigene3275_All, Unigene3542_All, Unigene3942_All, Unigene4286_All, Unigene537_All, Unigene675_All, Unigene931_All
35		CL10342.Contig1_All, CL10360.Contig1_All, CL10410.Contig1_All, CL10532.Contig1_All, CL1055.Contig1_All, CL1055.Contig2_All, CL1055.Contig3_All, CL1055.Contig4_All, CL10669.Contig1_All, CL10739.Contig1_All, CL10963.Contig1_All, CL11129.Contig1_All, CL11224.Contig1_All, CL11259.Contig1_All, CL11330.Contig1_All, CL11370.Contig1_All, CL11504.Contig1_All, CL11782.Contig1_All, CL11812.Contig1_All, CL11829.Contig1_All, CL11914.Contig1_All, CL11928.Contig1_All, CL11946.Contig1_All, CL11958.Contig1_All, CL12362.Contig1_All, CL12402.Contig1_All, CL12617.Contig1_All, CL12813.Contig1_All, CL12963.Contig1_All, CL13250.Contig1_All, CL13261.Contig1_All, CL13662.Contig1_All, CL13923.Contig1_All, CL13948.Contig1_All, CL14016.Contig1_All, CL14039.Contig1_All, CL14357.Contig1_All, CL14370.Contig1_All, CL14703.Contig1_All, CL15019.Contig1_All, CL15024.Contig1_All, CL15114.Contig1_All, CL15196.Contig1_All, CL15363.Contig1_All, CL1548.Contig2_All, CL15558.Contig1_All,

	Glutathione metabolism	CL15651.Contig1_All, CL1568.Contig1_All, CL1568.Contig2_All, CL1568.Contig3_All, CL1568.Contig4_All, CL15909.Contig1_All, CL16372.Contig1_All, CL17201.Contig1_All, CL17243.Contig1_All, CL17412.Contig1_All, CL18280.Contig1_All, CL185.Contig3_All, CL185.Contig7_All, CL185.Contig8_All, CL1882.Contig1_All, CL1882.Contig2_All, CL1882.Contig3_All, CL1882.Contig4_All, CL1882.Contig5_All, CL1882.Contig6_All, CL19812.Contig1_All, CL20516.Contig1_All, CL2108.Contig1_All, CL2108.Contig2_All, CL21848.Contig1_All, CL22106.Contig1_All, CL22379.Contig1_All, CL226.Contig3_All, CL226.Contig5_All, CL226.Contig7_All, CL22659.Contig1_All, CL22672.Contig1_All, CL22673.Contig1_All, CL233.Contig2_All, CL233.Contig3_All, CL233.Contig5_All, CL24022.Contig1_All, CL2429.Contig1_All, CL24608.Contig1_All, CL26000.Contig1_All, CL26096.Contig1_All, CL2621.Contig1_All, CL2621.Contig2_All, CL2655.Contig1_All, CL27289.Contig1_All, CL27328.Contig1_All, CL2745.Contig1_All, CL2745.Contig2_All, CL27880.Contig1_All, CL3111.Contig1_All, CL3111.Contig2_All, CL3111.Contig3_All, CL373.Contig1_All, CL4313.Contig1_All, CL4943.Contig1_All, CL4953.Contig1_All, CL4953.Contig2_All, CL4954.Contig1_All, CL4954.Contig2_All, CL51.Contig13_All, CL61.Contig10_All, CL61.Contig1_All, CL61.Contig2_All, CL61.Contig3_All, CL61.Contig4_All, CL61.Contig5_All, CL61.Contig6_All, CL61.Contig7_All, CL61.Contig8_All, CL61.Contig9_All, CL6190.Contig1_All, CL6529.Contig1_All, CL6529.Contig2_All, CL6579.Contig1_All, CL68.Contig1_All, CL68.Contig3_All, CL68.Contig4_All, CL68.Contig6_All, CL68.Contig8_All, CL7184.Contig2_All, CL7184.Contig3_All, CL7435.Contig1_All, CL7458.Contig1_All, CL7458.Contig2_All, CL7733.Contig1_All, CL8213.Contig1_All, CL8340.Contig1_All, CL8639.Contig1_All, CL8639.Contig2_All, CL8911.Contig1_All, CL893.Contig1_All, CL9358.Contig1_All, CL937.Contig1_All, CL9660.Contig1_All, Unigene1233_All, Unigene2530_All, Unigene2686_All, Unigene2970_All, Unigene338_All, Unigene339_All, Unigene3426_All, Unigene3868_All, Unigene4109_All, Unigene4336_All, Unigene4497_All, Unigene4542_All, Unigene4733_All, Unigene5280_All, Unigene5468_All, Unigene5474_All, Unigene5522_All, Unigene657_All, Unigene907_All
		CL1.Contig128_All, CL10064.Contig1_All, CL10577.Contig1_All, CL10629.Contig1_All, CL10684.Contig1_All, CL10743.Contig1_All, CL10881.Contig1_All, CL10924.Contig1_All, CL11092.Contig1_All, CL11408.Contig1_All, CL11490.Contig1_All, CL11655.Contig1_All, CL1214.Contig1_All, CL12645.Contig1_All, CL1312.Contig1_All, CL13220.Contig1_All,

36	Inositol phosphate metabolism	CL13571.Contig1_All, CL13588.Contig1_All, CL13593.Contig1_All, CL14136.Contig1_All, CL14532.Contig1_All, CL14732.Contig1_All, CL14855.Contig1_All, CL15140.Contig1_All, CL1531.Contig1_All, CL15918.Contig1_All, CL16492.Contig1_All, CL17401.Contig1_All, CL17705.Contig1_All, CL1871.Contig1_All, CL18732.Contig1_All, CL19075.Contig1_All, CL19319.Contig1_All, CL19523.Contig1_All, CL1976.Contig1_All, CL19884.Contig1_All, CL1989.Contig2_All, CL20024.Contig1_All, CL20167.Contig1_All, CL20313.Contig1_All, CL2079.Contig1_All, CL20796.Contig1_All, CL2096.Contig1_All, CL2111.Contig1_All, CL21183.Contig1_All, CL21476.Contig1_All, CL216.Contig1_All, CL216.Contig2_All, CL216.Contig3_All, CL216.Contig4_All, CL216.Contig5_All, CL21718.Contig1_All, CL21788.Contig1_All, CL22116.Contig1_All, CL22276.Contig1_All, CL22822.Contig1_All, CL2320.Contig1_All, CL2320.Contig2_All, CL2341.Contig1_All, CL23631.Contig1_All, CL24400.Contig1_All, CL2460.Contig1_All, CL2460.Contig2_All, CL25277.Contig1_All, CL2537.Contig1_All, CL2579.Contig1_All, CL2582.Contig1_All, CL26225.Contig1_All, CL26584.Contig1_All, CL26876.Contig1_All, CL26888.Contig1_All, CL27054.Contig1_All, CL27069.Contig1_All, CL27290.Contig1_All, CL27482.Contig1_All, CL27592.Contig1_All, CL27744.Contig1_All, CL27788.Contig1_All, CL3014.Contig1_All, CL3083.Contig1_All, CL3103.Contig1_All, CL3572.Contig1_All, CL3572.Contig2_All, CL3723.Contig1_All, CL3723.Contig2_All, CL4098.Contig1_All, CL4098.Contig2_All, CL4123.Contig1_All, CL4125.Contig1_All, CL4509.Contig1_All, CL456.Contig1_All, CL456.Contig2_All, CL456.Contig3_All, CL456.Contig4_All, CL456.Contig5_All, CL456.Contig6_All, CL4570.Contig1_All, CL4578.Contig1_All, CL4629.Contig3_All, CL4858.Contig1_All, CL4968.Contig1_All, CL5236.Contig1_All, CL5253.Contig1_All, CL5393.Contig1_All, CL5431.Contig1_All, CL5494.Contig1_All, CL557.Contig1_All, CL557.Contig2_All, CL557.Contig3_All, CL557.Contig4_All, CL5703.Contig1_All, CL5745.Contig1_All, CL5937.Contig1_All, CL6522.Contig1_All, CL6842.Contig1_All, CL7.Contig25_All, CL7072.Contig1_All, CL7383.Contig1_All, CL7518.Contig1_All, CL7627.Contig1_All, CL764.Contig1_All, CL7740.Contig1_All, CL8133.Contig1_All, CL8600.Contig1_All, CL8739.Contig1_All, CL9054.Contig1_All, CL9165.Contig1_All, CL917.Contig1_All, CL917.Contig2_All, CL917.Contig3_All, CL9204.Contig1_All, CL9981.Contig1_All, Unigene1020_All, Unigene1104_All, Unigene1135_All, Unigene1697_All, Unigene1987_All,

		Unigene1990_All, Unigene2094_All, Unigene2628_All, Unigene3138_All, Unigene3563_All, Unigene3980_All, Unigene4548_All, Unigene4558_All, Unigene4953_All, Unigene5302_All, Unigene5362_All, Unigene5373_All, Unigene5437_All, Unigene930_All, Unigene966_All, Unigene975_All
37	Cyanoamino acid metabolism	CL1.Contig101_All, CL1.Contig110_All, CL1.Contig39_All, CL1.Contig81_All, CL10161.Contig1_All, CL10306.Contig1_All, CL1051.Contig1_All, CL1051.Contig2_All, CL10594.Contig1_All, CL10668.Contig1_All, CL10775.Contig1_All, CL10951.Contig1_All, CL11392.Contig1_All, CL11524.Contig1_All, CL11812.Contig1_All, CL11819.Contig1_All, CL11835.Contig1_All, CL11900.Contig1_All, CL12239.Contig1_All, CL12317.Contig1_All, CL12443.Contig1_All, CL12640.Contig1_All, CL1266.Contig1_All, CL12714.Contig1_All, CL12842.Contig1_All, CL12945.Contig1_All, CL12945.Contig2_All, CL13050.Contig1_All, CL13232.Contig1_All, CL13445.Contig1_All, CL13770.Contig1_All, CL1409.Contig1_All, CL1409.Contig2_All, CL1409.Contig3_All, CL1409.Contig4_All, CL1409.Contig5_All, CL14098.Contig1_All, CL14522.Contig1_All, CL14941.Contig1_All, CL15335.Contig1_All, CL1545.Contig1_All, CL1545.Contig2_All, CL15606.Contig1_All, CL15623.Contig1_All, CL15670.Contig1_All, CL15730.Contig1_All, CL15730.Contig2_All, CL16130.Contig1_All, CL1645.Contig1_All, CL16810.Contig1_All, CL17004.Contig1_All, CL17498.Contig1_All, CL17542.Contig1_All, CL17962.Contig1_All, CL18183.Contig1_All, CL19381.Contig1_All, CL19619.Contig1_All, CL19792.Contig1_All, CL20001.Contig1_All, CL22098.Contig1_All, CL2227.Contig1_All, CL22294.Contig1_All, CL2245.Contig1_All, CL2245.Contig2_All, CL22561.Contig1_All, CL2262.Contig1_All, CL2262.Contig2_All, CL2262.Contig3_All, CL2262.Contig4_All, CL22926.Contig1_All, CL2306.Contig1_All, CL23525.Contig1_All, CL23909.Contig1_All, CL24023.Contig1_All, CL24040.Contig1_All, CL24070.Contig1_All, CL2429.Contig1_All, CL2516.Contig1_All, CL25208.Contig1_All, CL25490.Contig1_All, CL257.Contig1_All, CL257.Contig2_All, CL26226.Contig1_All, CL26274.Contig1_All, CL26460.Contig1_All, CL26770.Contig1_All, CL27267.Contig1_All, CL27432.Contig1_All, CL2991.Contig1_All, CL2991.Contig2_All, CL2991.Contig3_All, CL2995.Contig1_All, CL2995.Contig2_All, CL3191.Contig1_All, CL3310.Contig1_All, CL412.Contig1_All, CL412.Contig2_All, CL412.Contig3_All, CL4121.Contig1_All, CL4455.Contig1_All,

		CL4731.Contig1_All, CL4944.Contig1_All, CL50.Contig1_All, CL511.Contig1_All, CL511.Contig2_All, CL511.Contig3_All, CL511.Contig4_All, CL511.Contig5_All, CL5664.Contig1_All, CL619.Contig1_All, CL6493.Contig1_All, CL6799.Contig1_All, CL6799.Contig2_All, CL6877.Contig1_All, CL6877.Contig2_All, CL7521.Contig1_All, CL766.Contig1_All, CL766.Contig2_All, CL766.Contig3_All, CL766.Contig4_All, CL766.Contig5_All, CL7707.Contig1_All, CL7840.Contig1_All, CL8215.Contig1_All, CL8499.Contig1_All, CL8630.Contig1_All, CL8773.Contig1_All, CL9821.Contig1_All, Unigene1055_All, Unigene1232_All, Unigene1267_All, Unigene1788_All, Unigene1909_All, Unigene2125_All, Unigene2167_All, Unigene2334_All, Unigene2335_All, Unigene2590_All, Unigene2697_All, Unigene2865_All, Unigene316_All, Unigene3638_All, Unigene3648_All, Unigene3710_All, Unigene3711_All, Unigene3764_All, Unigene4180_All, Unigene4292_All, Unigene4420_All, Unigene4671_All, Unigene4727_All, Unigene5280_All, Unigene761_All
38	Aminoacyl-tRNA biosynthesis	CL10032.Contig1_All, CL1007.Contig1_All, CL10109.Contig1_All, CL10578.Contig1_All, CL1099.Contig1_All, CL11200.Contig1_All, CL11663.Contig1_All, CL11898.Contig1_All, CL12068.Contig1_All, CL12447.Contig1_All, CL12467.Contig1_All, CL12546.Contig1_All, CL12604.Contig1_All, CL1265.Contig1_All, CL12692.Contig1_All, CL12781.Contig1_All, CL12790.Contig1_All, CL12882.Contig1_All, CL13063.Contig1_All, CL13084.Contig1_All, CL13489.Contig1_All, CL14011.Contig1_All, CL14025.Contig1_All, CL14360.Contig1_All, CL1445.Contig3_All, CL1445.Contig6_All, CL14818.Contig1_All, CL15407.Contig1_All, CL1650.Contig1_All, CL17.Contig10_All, CL17.Contig11_All, CL17.Contig12_All, CL17.Contig13_All, CL17.Contig14_All, CL17.Contig15_All, CL17.Contig16_All, CL17.Contig17_All, CL17.Contig18_All, CL17.Contig19_All, CL17.Contig1_All, CL17.Contig20_All, CL17.Contig21_All, CL17.Contig22_All, CL17.Contig23_All, CL17.Contig24_All, CL17.Contig25_All, CL17.Contig28_All, CL17.Contig29_All, CL17.Contig2_All, CL17.Contig30_All, CL17.Contig31_All, CL17.Contig34_All, CL17.Contig35_All, CL17.Contig36_All, CL17.Contig37_All, CL17.Contig38_All, CL17.Contig3_All, CL17.Contig41_All, CL17.Contig43_All, CL17.Contig44_All, CL17.Contig45_All, CL17.Contig4_All, CL17.Contig5_All, CL17.Contig6_All, CL17.Contig7_All, CL17.Contig8_All, CL17.Contig9_All, CL19441.Contig1_All, CL20115.Contig1_All, CL20612.Contig1_All, CL20823.Contig1_All, CL2090.Contig1_All, CL20931.Contig1_All, CL21275.Contig1_All, CL22103.Contig1_All, CL23045.Contig1_All, CL2312.Contig1_All, CL23396.Contig1_All, CL2351.Contig1_All, CL23833.Contig1_All, CL2413.Contig1_All, CL24213.Contig1_All, CL24665.Contig1_All,

		CL2752.Contig1_All, CL2965.Contig1_All, CL3236.Contig1_All, CL3270.Contig1_All, CL3270.Contig2_All, CL3270.Contig3_All, CL3270.Contig4_All, CL3412.Contig1_All, CL352.Contig1_All, CL352.Contig2_All, CL352.Contig3_All, CL352.Contig4_All, CL352.Contig5_All, CL352.Contig6_All, CL3555.Contig1_All, CL3736.Contig1_All, CL3866.Contig1_All, CL3880.Contig1_All, CL3974.Contig1_All, CL3974.Contig2_All, CL4011.Contig1_All, CL4431.Contig1_All, CL4574.Contig1_All, CL4574.Contig2_All, CL4576.Contig1_All, CL4899.Contig1_All, CL506.Contig1_All, CL520.Contig1_All, CL520.Contig2_All, CL520.Contig3_All, CL520.Contig4_All, CL5312.Contig1_All, CL5440.Contig1_All, CL5440.Contig2_All, CL5487.Contig1_All, CL5865.Contig1_All, CL6083.Contig1_All, CL6375.Contig1_All, CL6467.Contig1_All, CL655.Contig1_All, CL655.Contig2_All, CL655.Contig3_All, CL6765.Contig1_All, CL677.Contig1_All, CL677.Contig2_All, CL677.Contig3_All, CL677.Contig4_All, CL677.Contig5_All, CL677.Contig7_All, CL7362.Contig1_All, CL763.Contig1_All, CL763.Contig2_All, CL763.Contig3_All, CL763.Contig4_All, CL763.Contig5_All, CL8156.Contig1_All, CL8671.Contig1_All, CL887.Contig1_All, CL887.Contig2_All, CL887.Contig3_All, CL999.Contig1_All, Unigene3082_All, Unigene3376_All, Unigene3479_All, Unigene3554_All, Unigene4267_All, Unigene4383_All, Unigene524_All
39		CL10095.Contig1_All, CL10308.Contig1_All, CL10323.Contig1_All, CL1044.Contig2_All, CL10597.Contig1_All, CL1118.Contig1_All, CL1118.Contig2_All, CL11220.Contig1_All, CL11220.Contig2_All, CL12309.Contig1_All, CL12383.Contig1_All, CL12839.Contig1_All, CL1404.Contig1_All, CL1404.Contig2_All, CL1404.Contig3_All, CL1404.Contig4_All, CL15032.Contig1_All, CL15228.Contig1_All, CL15673.Contig1_All, CL15707.Contig1_All, CL15939.Contig1_All, CL1687.Contig1_All, CL1687.Contig2_All, CL1687.Contig3_All, CL17225.Contig1_All, CL17304.Contig1_All, CL17507.Contig1_All, CL17836.Contig1_All, CL17910.Contig1_All, CL18432.Contig1_All, CL1853.Contig1_All, CL1853.Contig2_All, CL18811.Contig1_All, CL18844.Contig1_All, CL18886.Contig1_All, CL1890.Contig1_All, CL19131.Contig1_All, CL19529.Contig1_All, CL19671.Contig1_All, CL19740.Contig1_All, CL19908.Contig1_All, CL1992.Contig1_All, CL20091.Contig1_All, CL20954.Contig1_All, CL21457.Contig1_All, CL21485.Contig1_All, CL2156.Contig1_All, CL21832.Contig1_All, CL2230.Contig1_All, CL2230.Contig2_All, CL2247.Contig1_All, CL22481.Contig1_All, CL22565.Contig1_All, CL22646.Contig1_All,

	ABC transporters	CL22743.Contig1_All, CL23022.Contig1_All, CL23112.Contig1_All, CL23259.Contig1_All, CL23283.Contig1_All, CL23319.Contig1_All, CL23705.Contig1_All, CL23755.Contig1_All, CL23801.Contig1_All, CL23856.Contig1_All, CL23988.Contig1_All, CL24844.Contig1_All, CL24912.Contig1_All, CL25437.Contig1_All, CL2605.Contig1_All, CL26704.Contig1_All, CL2695.Contig1_All, CL2695.Contig2_All, CL27129.Contig1_All, CL27179.Contig1_All, CL2765.Contig1_All, CL2765.Contig2_All, CL2850.Contig2_All, CL2895.Contig1_All, CL2956.Contig1_All, CL31.Contig11_All, CL31.Contig17_All, CL31.Contig18_All, CL31.Contig5_All, CL3102.Contig1_All, CL321.Contig1_All, CL3215.Contig1_All, CL3537.Contig1_All, CL3786.Contig1_All, CL3946.Contig1_All, CL402.Contig1_All, CL402.Contig3_All, CL4166.Contig1_All, CL4232.Contig1_All, CL4302.Contig1_All, CL4626.Contig1_All, CL473.Contig1_All, CL4993.Contig1_All, CL501.Contig1_All, CL5120.Contig1_All, CL5287.Contig1_All, CL584.Contig1_All, CL5885.Contig1_All, CL5915.Contig1_All, CL594.Contig1_All, CL6137.Contig1_All, CL6138.Contig1_All, CL6359.Contig1_All, CL6730.Contig2_All, CL6826.Contig1_All, CL7281.Contig1_All, CL7385.Contig1_All, CL7577.Contig1_All, CL7637.Contig1_All, CL8268.Contig1_All, CL8325.Contig1_All, CL8325.Contig2_All, CL8684.Contig1_All, CL879.Contig1_All, CL8929.Contig1_All, CL934.Contig2_All, CL934.Contig3_All, CL9380.Contig1_All, CL9380.Contig2_All, CL9401.Contig1_All, CL953.Contig1_All, CL953.Contig2_All, CL9692.Contig1_All, CL9734.Contig1_All, CL984.Contig1_All, Unigene1014_All, Unigene1196_All, Unigene1199_All, Unigene1385_All, Unigene1519_All, Unigene1628_All, Unigene2242_All, Unigene231_All, Unigene3212_All, Unigene3779_All, Unigene390_All, Unigene3961_All, Unigene4326_All, Unigene5231_All, Unigene5489_All, Unigene628_All, Unigene718_All, Unigene94_All, Unigene997_All
		CL10224.Contig1_All, CL10235.Contig1_All, CL1035.Contig1_All, CL1035.Contig2_All, CL10456.Contig1_All, CL10495.Contig1_All, CL10496.Contig1_All, CL10557.Contig1_All, CL10587.Contig1_All, CL10740.Contig1_All, CL11237.Contig1_All, CL11296.Contig1_All, CL11375.Contig1_All, CL1150.Contig1_All, CL11699.Contig1_All, CL11843.Contig1_All, CL12102.Contig1_All, CL12208.Contig1_All, CL12498.Contig1_All, CL12644.Contig1_All, CL12687.Contig1_All, CL12937.Contig1_All, CL12951.Contig1_All, CL13326.Contig1_All, CL13779.Contig1_All, CL13814.Contig1_All, CL14024.Contig1_All, CL1412.Contig1_All, CL1412.Contig2_All, CL14252.Contig1_All, CL14352.Contig1_All, CL15424.Contig1_All,

40	Pentose and glucuronate interconversions	CL16037.Contig1_All, CL16080.Contig1_All, CL16239.Contig1_All, CL16353.Contig1_All, CL16871.Contig1_All, CL17355.Contig1_All, CL178.Contig1_All, CL178.Contig2_All, CL178.Contig3_All, CL178.Contig4_All, CL18472.Contig1_All, CL18626.Contig1_All, CL18792.Contig1_All, CL18822.Contig1_All, CL198.Contig1_All, CL198.Contig2_All, CL198.Contig3_All, CL198.Contig4_All, CL198.Contig5_All, CL198.Contig6_All, CL198.Contig7_All, CL198.Contig8_All, CL198.Contig9_All, CL19809.Contig1_All, CL20593.Contig1_All, CL20657.Contig1_All, CL20752.Contig1_All, CL21180.Contig1_All, CL21862.Contig1_All, CL23213.Contig1_All, CL23302.Contig1_All, CL23456.Contig1_All, CL23687.Contig1_All, CL24071.Contig1_All, CL24640.Contig1_All, CL25312.Contig1_All, CL25467.Contig1_All, CL25922.Contig1_All, CL26426.Contig1_All, CL26793.Contig1_All, CL26980.Contig1_All, CL27648.Contig1_All, CL27778.Contig1_All, CL28111.Contig1_All, CL2929.Contig1_All, CL3003.Contig1_All, CL3022.Contig1_All, CL3200.Contig1_All, CL331.Contig1_All, CL331.Contig3_All, CL3456.Contig1_All, CL3457.Contig1_All, CL3549.Contig1_All, CL3770.Contig1_All, CL3879.Contig1_All, CL3932.Contig2_All, CL4497.Contig1_All, CL4771.Contig1_All, CL5225.Contig1_All, CL5546.Contig1_All, CL5549.Contig1_All, CL5549.Contig2_All, CL5985.Contig1_All, CL5985.Contig2_All, CL6330.Contig1_All, CL6371.Contig1_All, CL6389.Contig1_All, CL6471.Contig1_All, CL7085.Contig1_All, CL7101.Contig1_All, CL7155.Contig1_All, CL7387.Contig1_All, CL7463.Contig1_All, CL7555.Contig1_All, CL7562.Contig1_All, CL7580.Contig1_All, CL760.Contig3_All, CL7823.Contig1_All, CL8104.Contig1_All, CL8165.Contig1_All, CL829.Contig1_All, CL829.Contig2_All, CL829.Contig3_All, CL8320.Contig1_All, CL8338.Contig1_All, CL8338.Contig2_All, CL8346.Contig1_All, CL8452.Contig1_All, CL8514.Contig1_All, CL860.Contig1_All, CL860.Contig2_All, CL860.Contig3_All, CL860.Contig4_All, CL8748.Contig1_All, CL9106.Contig1_All, CL9598.Contig1_All, CL9958.Contig1_All, Unigene1738_All, Unigene2485_All, Unigene2688_All, Unigene3094_All, Unigene3202_All, Unigene3468_All, Unigene4417_All, Unigene4843_All, Unigene507_All, Unigene5188_All, Unigene579_All, Unigene622_All, Unigene640_All
		CL10136.Contig1_All, CL10238.Contig1_All, CL10460.Contig1_All, CL10511.Contig1_All, CL10568.Contig1_All, CL10740.Contig1_All, CL10816.Contig1_All, CL10994.Contig1_All, CL11017.Contig1_All, CL11117.Contig1_All, CL11262.Contig1_All, CL11575.Contig1_All, CL11695.Contig1_All, CL11741.Contig1_All,

41	Arginine and proline metabolism	CL11909.Contig1_All, CL12133.Contig1_All, CL12194.Contig1_All, CL12297.Contig1_All, CL12498.Contig1_All, CL12629.Contig1_All, CL12762.Contig1_All, CL13384.Contig1_All, CL13384.Contig2_All, CL13527.Contig1_All, CL13789.Contig1_All, CL1412.Contig1_All, CL1412.Contig2_All, CL14304.Contig1_All, CL1469.Contig3_All, CL1552.Contig1_All, CL1578.Contig1_All, CL1578.Contig2_All, CL1578.Contig3_All, CL1578.Contig4_All, CL1599.Contig1_All, CL16043.Contig1_All, CL16157.Contig1_All, CL16239.Contig1_All, CL16388.Contig1_All, CL16543.Contig1_All, CL16871.Contig1_All, CL17852.Contig1_All, CL18159.Contig1_All, CL1882.Contig1_All, CL1882.Contig2_All, CL1882.Contig3_All, CL1882.Contig4_All, CL1882.Contig5_All, CL1882.Contig6_All, CL19904.Contig1_All, CL21259.Contig1_All, CL21764.Contig1_All, CL22010.Contig1_All, CL22065.Contig1_All, CL2207.Contig1_All, CL22411.Contig1_All, CL23024.Contig1_All, CL23375.Contig1_All, CL23582.Contig1_All, CL23687.Contig1_All, CL24077.Contig1_All, CL2483.Contig1_All, CL25987.Contig1_All, CL2621.Contig1_All, CL2621.Contig2_All, CL26806.Contig1_All, CL27486.Contig1_All, CL27867.Contig1_All, CL2929.Contig1_All, CL3057.Contig1_All, CL323.Contig1_All, CL323.Contig2_All, CL323.Contig3_All, CL323.Contig4_All, CL3457.Contig1_All, CL3618.Contig1_All, CL3831.Contig1_All, CL3841.Contig1_All, CL4171.Contig1_All, CL4171.Contig2_All, CL4199.Contig1_All, CL4299.Contig1_All, CL4299.Contig2_All, CL4339.Contig1_All, CL4430.Contig1_All, CL51.Contig10_All, CL51.Contig11_All, CL51.Contig12_All, CL51.Contig14_All, CL51.Contig15_All, CL51.Contig1_All, CL51.Contig2_All, CL51.Contig3_All, CL51.Contig4_All, CL51.Contig5_All, CL51.Contig6_All, CL51.Contig8_All, CL51.Contig9_All, CL5549.Contig1_All, CL5549.Contig2_All, CL56.Contig10_All, CL56.Contig12_All, CL56.Contig9_All, CL5649.Contig1_All, CL612.Contig1_All, CL63.Contig3_All, CL6371.Contig1_All, CL6396.Contig1_All, CL6579.Contig1_All, CL6715.Contig1_All, CL6870.Contig1_All, CL7049.Contig1_All, CL7409.Contig1_All, CL7800.Contig1_All, CL7816.Contig1_All, CL812.Contig1_All, CL8217.Contig1_All, CL837.Contig1_All, CL837.Contig2_All, CL837.Contig3_All, CL837.Contig4_All, CL8386.Contig1_All, CL8390.Contig1_All, CL8602.Contig1_All, CL8687.Contig1_All, CL8691.Contig1_All, CL8723.Contig1_All, CL8762.Contig1_All, CL9106.Contig1_All, CL9450.Contig1_All, CL9805.Contig1_All, CL9960.Contig1_All, Unigene1712_All, Unigene1774_All, Unigene2486_All, Unigene259_All, Unigene2600_All,

		Unigene3426_All, Unigene4020_All, Unigene4417_All
42	Carotenoid biosynthesis	CL10242.Contig1_All, CL10278.Contig1_All, CL10637.Contig1_All, CL10934.Contig1_All, CL11116.Contig1_All, CL11595.Contig1_All, CL11944.Contig1_All, CL1197.Contig1_All, CL1197.Contig2_All, CL11985.Contig1_All, CL12059.Contig1_All, CL12250.Contig1_All, CL1256.Contig1_All, CL1256.Contig2_All, CL12888.Contig1_All, CL12925.Contig1_All, CL12985.Contig1_All, CL13535.Contig1_All, CL13538.Contig1_All, CL13575.Contig1_All, CL143.Contig1_All, CL143.Contig4_All, CL14782.Contig1_All, CL14824.Contig1_All, CL1529.Contig1_All, CL1529.Contig2_All, CL1529.Contig3_All, CL1529.Contig4_All, CL1529.Contig5_All, CL15867.Contig1_All, CL16173.Contig1_All, CL1625.Contig1_All, CL16661.Contig1_All, CL16813.Contig1_All, CL17086.Contig1_All, CL17086.Contig2_All, CL17409.Contig1_All, CL17554.Contig1_All, CL18532.Contig1_All, CL18669.Contig1_All, CL19044.Contig1_All, CL2003.Contig1_All, CL2003.Contig2_All, CL2003.Contig3_All, CL2003.Contig4_All, CL2030.Contig1_All, CL20473.Contig1_All, CL2049.Contig2_All, CL2049.Contig4_All, CL2053.Contig1_All, CL2053.Contig2_All, CL20630.Contig1_All, CL20798.Contig1_All, CL21613.Contig1_All, CL2253.Contig1_All, CL2253.Contig2_All, CL2253.Contig3_All, CL22584.Contig1_All, CL23047.Contig1_All, CL23526.Contig1_All, CL23611.Contig1_All, CL25282.Contig1_All, CL25842.Contig1_All, CL26560.Contig1_All, CL2729.Contig1_All, CL27439.Contig1_All, CL2778.Contig1_All, CL3314.Contig2_All, CL3444.Contig1_All, CL3528.Contig1_All, CL3610.Contig1_All, CL3673.Contig1_All, CL3777.Contig1_All, CL3804.Contig1_All, CL3804.Contig2_All, CL3952.Contig1_All, CL3955.Contig1_All, CL3988.Contig1_All, CL3992.Contig1_All, CL4141.Contig1_All, CL4174.Contig1_All, CL4256.Contig1_All, CL4306.Contig1_All, CL4306.Contig2_All, CL4405.Contig1_All, CL4462.Contig1_All, CL4869.Contig1_All, CL5526.Contig1_All, CL5622.Contig1_All, CL5737.Contig1_All, CL649.Contig1_All, CL649.Contig2_All, CL6981.Contig1_All, CL7032.Contig1_All, CL7289.Contig1_All, CL7460.Contig1_All, CL7478.Contig1_All, CL752.Contig1_All, CL752.Contig2_All, CL752.Contig3_All, CL752.Contig4_All, CL752.Contig5_All, CL8059.Contig1_All, CL8423.Contig1_All, CL849.Contig1_All, CL849.Contig2_All, CL8707.Contig1_All, CL898.Contig1_All, CL898.Contig2_All, CL9055.Contig1_All, CL9195.Contig1_All, CL932.Contig1_All, CL932.Contig2_All, CL932.Contig3_All,

		CL932.Contig4_All, CL9342.Contig1_All, CL9876.Contig1_All, CL9876.Contig2_All, CL9984.Contig1_All, Unigene1401_All, Unigene1914_All, Unigene2178_All, Unigene2302_All, Unigene2549_All, Unigene2869_All, Unigene2888_All, Unigene3055_All, Unigene3769_All, Unigene3793_All, Unigene3870_All, Unigene4432_All, Unigene4433_All, Unigene4999_All, Unigene5015_All, Unigene5519_All, Unigene735_All
43	RNA polymerase	CL1.Contig102_All, CL1.Contig12_All, CL1.Contig57_All, CL10058.Contig1_All, CL10406.Contig1_All, CL10497.Contig1_All, CL10652.Contig1_All, CL10746.Contig1_All, CL1084.Contig1_All, CL1084.Contig2_All, CL1084.Contig3_All, CL1084.Contig4_All, CL1084.Contig5_All, CL11137.Contig1_All, CL11279.Contig1_All, CL11464.Contig1_All, CL12668.Contig1_All, CL13523.Contig1_All, CL13714.Contig1_All, CL14122.Contig1_All, CL14122.Contig2_All, CL1426.Contig1_All, CL14423.Contig1_All, CL14503.Contig1_All, CL14510.Contig1_All, CL14510.Contig2_All, CL1484.Contig1_All, CL1484.Contig2_All, CL1484.Contig3_All, CL14954.Contig1_All, CL15100.Contig1_All, CL15697.Contig1_All, CL16128.Contig1_All, CL16515.Contig1_All, CL16771.Contig1_All, CL17057.Contig1_All, CL17405.Contig1_All, CL18317.Contig1_All, CL18325.Contig1_All, CL18538.Contig1_All, CL1863.Contig2_All, CL1863.Contig3_All, CL19445.Contig1_All, CL19651.Contig1_All, CL19667.Contig1_All, CL20405.Contig1_All, CL21003.Contig1_All, CL2179.Contig1_All, CL2181.Contig1_All, CL22044.Contig1_All, CL22913.Contig1_All, CL23155.Contig1_All, CL23227.Contig1_All, CL23312.Contig1_All, CL23629.Contig1_All, CL24517.Contig1_All, CL24536.Contig1_All, CL24786.Contig1_All, CL25009.Contig1_All, CL25107.Contig1_All, CL25340.Contig1_All, CL25515.Contig1_All, CL25836.Contig1_All, CL264.Contig8_All, CL2679.Contig1_All, CL26933.Contig1_All, CL2775.Contig1_All, CL27834.Contig1_All, CL2839.Contig1_All, CL3027.Contig1_All, CL3134.Contig1_All, CL3376.Contig1_All, CL3434.Contig1_All, CL351.Contig1_All, CL351.Contig5_All, CL4359.Contig1_All, CL4363.Contig1_All, CL4363.Contig3_All, CL4453.Contig1_All, CL4593.Contig1_All, CL4616.Contig1_All, CL4724.Contig1_All, CL4793.Contig1_All, CL4903.Contig1_All, CL498.Contig1_All, CL5243.Contig1_All, CL5607.Contig2_All, CL5618.Contig1_All, CL5618.Contig2_All, CL5854.Contig1_All, CL6299.Contig1_All, CL6388.Contig1_All, CL699.Contig1_All, CL7090.Contig1_All, CL7112.Contig1_All, CL7167.Contig1_All, CL7279.Contig1_All, CL77.Contig10_All,

		CL77.Contig1_All, CL77.Contig2_All, CL77.Contig5_All, CL77.Contig7_All, CL77.Contig8_All, CL77.Contig9_All, CL771.Contig1_All, CL7923.Contig1_All, CL7923.Contig2_All, CL8159.Contig1_All, CL8437.Contig1_All, CL8487.Contig1_All, CL8511.Contig1_All, CL8564.Contig1_All, CL8668.Contig1_All, CL9266.Contig1_All, CL928.Contig3_All, CL9303.Contig1_All, CL9570.Contig1_All, CL9797.Contig1_All, CL9799.Contig1_All, Unigene1335_All, Unigene1597_All, Unigene1792_All, Unigene1935_All, Unigene2195_All, Unigene229_All, Unigene2509_All, Unigene2550_All, Unigene2613_All, Unigene3236_All, Unigene3522_All, Unigene3657_All, Unigene4051_All, Unigene4444_All, Unigene734_All, Unigene87_All
44	DNA replication	CL106.Contig1_All, CL106.Contig2_All, CL106.Contig3_All, CL106.Contig4_All, CL106.Contig5_All, CL106.Contig6_All, CL10900.Contig1_All, CL11511.Contig1_All, CL11927.Contig1_All, CL12181.Contig1_All, CL12987.Contig1_All, CL13080.Contig1_All, CL1342.Contig1_All, CL1342.Contig2_All, CL1342.Contig3_All, CL1342.Contig4_All, CL14493.Contig1_All, CL14981.Contig1_All, CL15039.Contig1_All, CL15256.Contig1_All, CL16014.Contig1_All, CL16648.Contig1_All, CL16686.Contig1_All, CL17834.Contig1_All, CL17879.Contig1_All, CL18218.Contig1_All, CL18441.Contig1_All, CL1870.Contig2_All, CL18701.Contig1_All, CL188.Contig1_All, CL188.Contig4_All, CL19828.Contig1_All, CL19845.Contig1_All, CL19942.Contig1_All, CL20521.Contig1_All, CL20837.Contig1_All, CL20934.Contig1_All, CL20993.Contig1_All, CL2129.Contig2_All, CL21471.Contig1_All, CL21475.Contig1_All, CL22100.Contig1_All, CL22419.Contig1_All, CL22549.Contig1_All, CL22629.Contig1_All, CL22823.Contig1_All, CL22971.Contig1_All, CL23001.Contig1_All, CL23087.Contig1_All, CL2317.Contig1_All, CL23363.Contig1_All, CL23570.Contig1_All, CL23708.Contig1_All, CL23834.Contig1_All, CL24063.Contig1_All, CL24095.Contig1_All, CL24277.Contig1_All, CL24615.Contig1_All, CL24635.Contig1_All, CL24709.Contig1_All, CL24714.Contig1_All, CL24728.Contig1_All, CL24839.Contig1_All, CL25044.Contig1_All, CL25417.Contig1_All, CL25951.Contig1_All, CL26122.Contig1_All, CL26303.Contig1_All, CL26337.Contig1_All, CL2646.Contig1_All, CL26534.Contig1_All, CL26689.Contig1_All, CL27138.Contig1_All, CL27160.Contig1_All, CL27375.Contig1_All, CL27423.Contig1_All, CL27901.Contig1_All, CL3164.Contig1_All, CL3367.Contig1_All, CL3367.Contig2_All, CL3514.Contig2_All, CL4205.Contig1_All, CL4550.Contig1_All,

		CL4617.Contig1_All, CL5569.Contig1_All, CL564.Contig1_All, CL5743.Contig1_All, CL5912.Contig1_All, CL6077.Contig1_All, CL6169.Contig1_All, CL6288.Contig1_All, CL6483.Contig1_All, CL6508.Contig1_All, CL6763.Contig1_All, CL6917.Contig1_All, CL7412.Contig2_All, CL7433.Contig1_All, CL7610.Contig1_All, CL7610.Contig2_All, CL8259.Contig1_All, CL8317.Contig1_All, CL8358.Contig1_All, CL8528.Contig1_All, CL8644.Contig1_All, CL8857.Contig2_All, CL9093.Contig1_All, CL9317.Contig1_All, CL9755.Contig1_All, CL9782.Contig1_All, CL9924.Contig1_All, CL9987.Contig1_All, Unigene109_All, Unigene1386_All, Unigene2133_All, Unigene234_All, Unigene2408_All, Unigene2585_All, Unigene2846_All, Unigene2876_All, Unigene2881_All, Unigene3151_All, Unigene3274_All, Unigene3275_All, Unigene3456_All, Unigene3789_All, Unigene4017_All, Unigene4038_All, Unigene4211_All, Unigene4286_All, Unigene434_All, Unigene606_All, Unigene675_All
45	Zeatin biosynthesis	CL10236.Contig1_All, CL10748.Contig1_All, CL10814.Contig1_All, CL11752.Contig1_All, CL12160.Contig1_All, CL1244.Contig1_All, CL1250.Contig1_All, CL1250.Contig4_All, CL12981.Contig1_All, CL13018.Contig1_All, CL13316.Contig1_All, CL13763.Contig1_All, CL14860.Contig1_All, CL14941.Contig1_All, CL15304.Contig1_All, CL15629.Contig1_All, CL15759.Contig1_All, CL16006.Contig1_All, CL16018.Contig1_All, CL16034.Contig1_All, CL16640.Contig1_All, CL17069.Contig1_All, CL17425.Contig1_All, CL1753.Contig1_All, CL17547.Contig1_All, CL17699.Contig1_All, CL181.Contig4_All, CL18450.Contig1_All, CL18550.Contig1_All, CL18601.Contig1_All, CL19398.Contig1_All, CL19628.Contig1_All, CL20175.Contig1_All, CL20242.Contig1_All, CL20301.Contig1_All, CL20484.Contig1_All, CL20705.Contig1_All, CL2115.Contig1_All, CL21945.Contig1_All, CL2278.Contig1_All, CL23027.Contig1_All, CL24469.Contig1_All, CL2455.Contig1_All, CL24829.Contig1_All, CL24981.Contig1_All, CL25160.Contig1_All, CL25270.Contig1_All, CL2546.Contig1_All, CL26083.Contig1_All, CL26113.Contig1_All, CL26274.Contig1_All, CL264.Contig1_All, CL264.Contig2_All, CL264.Contig3_All, CL264.Contig4_All, CL264.Contig5_All, CL264.Contig6_All, CL264.Contig7_All, CL2664.Contig1_All, CL2672.Contig1_All, CL27025.Contig1_All, CL275.Contig2_All, CL2750.Contig2_All, CL27887.Contig1_All, CL3053.Contig1_All, CL3716.Contig1_All, CL3716.Contig2_All, CL4156.Contig1_All, CL4156.Contig2_All,

		CL4243.Contig1_All, CL4243.Contig2_All, CL4813.Contig1_All, CL4876.Contig1_All, CL5081.Contig1_All, CL511.Contig1_All, CL511.Contig3_All, CL511.Contig4_All, CL511.Contig5_All, CL5266.Contig1_All, CL53.Contig10_All, CL53.Contig11_All, CL53.Contig1_All, CL53.Contig3_All, CL53.Contig6_All, CL53.Contig7_All, CL53.Contig8_All, CL53.Contig9_All, CL5691.Contig1_All, CL5691.Contig2_All, CL6124.Contig1_All, CL6243.Contig1_All, CL6475.Contig1_All, CL6877.Contig2_All, CL697.Contig1_All, CL7364.Contig1_All, CL7497.Contig1_All, CL7593.Contig1_All, CL8215.Contig1_All, CL8276.Contig1_All, CL8496.Contig1_All, CL8612.Contig1_All, CL8915.Contig1_All, Unigene1109_All, Unigene1287_All, Unigene1470_All, Unigene1586_All, Unigene1707_All, Unigene2148_All, Unigene2308_All, Unigene2759_All, Unigene2761_All, Unigene3131_All, Unigene327_All, Unigene3708_All, Unigene3744_All, Unigene3751_All, Unigene3756_All, Unigene3764_All, Unigene3838_All, Unigene3862_All, Unigene4065_All, Unigene4325_All, Unigene4565_All, Unigene5380_All, Unigene5446_All, Unigene5483_All, Unigene793_All
46	Tryptophan metabolism	CL1.Contig101_All, CL1.Contig110_All, CL1.Contig39_All, CL1.Contig81_All, CL10161.Contig1_All, CL10215.Contig1_All, CL10551.Contig1_All, CL10668.Contig1_All, CL10740.Contig1_All, CL10856.Contig1_All, CL11310.Contig1_All, CL11392.Contig1_All, CL11524.Contig1_All, CL11759.Contig1_All, CL11900.Contig1_All, CL12067.Contig1_All, CL12115.Contig1_All, CL12204.Contig1_All, CL12239.Contig1_All, CL12345.Contig1_All, CL12498.Contig1_All, CL12550.Contig1_All, CL12555.Contig1_All, CL12720.Contig1_All, CL12838.Contig1_All, CL12842.Contig1_All, CL13023.Contig1_All, CL13054.Contig1_All, CL13253.Contig1_All, CL13530.Contig1_All, CL13661.Contig1_All, CL1412.Contig1_All, CL1412.Contig2_All, CL14294.Contig1_All, CL14695.Contig1_All, CL15166.Contig1_All, CL15335.Contig1_All, CL1545.Contig1_All, CL1545.Contig2_All, CL15451.Contig1_All, CL15676.Contig1_All, CL15730.Contig1_All, CL15867.Contig1_All, CL16239.Contig1_All, CL16288.Contig1_All, CL16298.Contig1_All, CL16543.Contig1_All, CL16871.Contig1_All, CL17086.Contig1_All, CL17086.Contig2_All, CL18160.Contig1_All, CL18183.Contig1_All, CL18669.Contig1_All, CL18942.Contig1_All, CL19111.Contig1_All, CL19283.Contig1_All, CL19381.Contig1_All, CL19619.Contig1_All, CL19754.Contig1_All, CL20630.Contig1_All, CL21454.Contig1_All, CL21613.Contig1_All, CL21901.Contig1_All, CL22180.Contig1_All, CL23271.Contig1_All,

		CL23294.Contig1_All, CL23375.Contig1_All, CL23611.Contig1_All, CL23687.Contig1_All, CL25282.Contig1_All, CL25842.Contig1_All, CL25992.Contig1_All, CL26958.Contig1_All, CL27439.Contig1_All, CL2929.Contig1_All, CL2950.Contig1_All, CL3182.Contig1_All, CL3182.Contig2_All, CL3448.Contig1_All, CL3448.Contig2_All, CL3457.Contig1_All, CL3545.Contig1_All, CL40.Contig1_All, CL4141.Contig1_All, CL4463.Contig1_All, CL4944.Contig1_All, CL5483.Contig1_All, CL5504.Contig1_All, CL5549.Contig1_All, CL5549.Contig2_All, CL6141.Contig2_All, CL6371.Contig1_All, CL6765.Contig1_All, CL7442.Contig1_All, CL7476.Contig1_All, CL7478.Contig1_All, CL8297.Contig1_All, CL8307.Contig1_All, CL849.Contig1_All, CL849.Contig2_All, CL8499.Contig1_All, CL8555.Contig1_All, CL8671.Contig1_All, CL9106.Contig1_All, CL9429.Contig1_All, CL9821.Contig1_All, Unigene1054_All, Unigene1055_All, Unigene1796_All, Unigene1909_All, Unigene2178_All, Unigene2549_All, Unigene2696_All, Unigene2697_All, Unigene2872_All, Unigene316_All, Unigene3769_All, Unigene4292_All, Unigene4409_All, Unigene4417_All, Unigene4528_All, Unigene4627_All, Unigene4671_All, Unigene826_All, Unigene920_All
47	Regulation of autophagy	CL10077.Contig1_All, CL1016.Contig3_All, CL10635.Contig1_All, CL10752.Contig1_All, CL10913.Contig1_All, CL12205.Contig1_All, CL12355.Contig1_All, CL12407.Contig1_All, CL12645.Contig1_All, CL1278.Contig1_All, CL1296.Contig1_All, CL1296.Contig4_All, CL1296.Contig5_All, CL13446.Contig1_All, CL13715.Contig1_All, CL13868.Contig1_All, CL13981.Contig1_All, CL14030.Contig1_All, CL1411.Contig1_All, CL146.Contig1_All, CL146.Contig2_All, CL146.Contig3_All, CL146.Contig4_All, CL146.Contig5_All, CL146.Contig6_All, CL14633.Contig1_All, CL14665.Contig1_All, CL15050.Contig1_All, CL1540.Contig1_All, CL1540.Contig2_All, CL1540.Contig3_All, CL1592.Contig1_All, CL16046.Contig1_All, CL16178.Contig1_All, CL16216.Contig1_All, CL16321.Contig1_All, CL16321.Contig2_All, CL16400.Contig1_All, CL16732.Contig1_All, CL17039.Contig1_All, CL1768.Contig1_All, CL1800.Contig1_All, CL18439.Contig1_All, CL18469.Contig1_All, CL19914.Contig1_All, CL2009.Contig1_All, CL20138.Contig1_All, CL2029.Contig1_All, CL2040.Contig1_All, CL22503.Contig1_All, CL2335.Contig1_All, CL2341.Contig1_All, CL23437.Contig1_All, CL242.Contig1_All, CL242.Contig2_All, CL242.Contig3_All, CL242.Contig4_All, CL242.Contig5_All, CL2449.Contig1_All, CL2451.Contig1_All, CL268.Contig1_All, CL268.Contig2_All, CL268.Contig3_All, CL26837.Contig1_All,

		CL27543.Contig1_All, CL27602.Contig1_All, CL2807.Contig1_All, CL2886.Contig1_All, CL3247.Contig1_All, CL3605.Contig1_All, CL3657.Contig1_All, CL3657.Contig2_All, CL3686.Contig1_All, CL3812.Contig1_All, CL3977.Contig1_All, CL404.Contig2_All, CL404.Contig3_All, CL404.Contig4_All, CL404.Contig5_All, CL404.Contig6_All, CL404.Contig7_All, CL4190.Contig2_All, CL4218.Contig1_All, CL4273.Contig1_All, CL4488.Contig1_All, CL4488.Contig2_All, CL5200.Contig1_All, CL5200.Contig2_All, CL523.Contig1_All, CL5253.Contig1_All, CL5532.Contig1_All, CL5772.Contig1_All, CL633.Contig1_All, CL647.Contig1_All, CL647.Contig2_All, CL647.Contig3_All, CL647.Contig4_All, CL647.Contig5_All, CL647.Contig6_All, CL647.Contig7_All, CL6798.Contig1_All, CL7.Contig21_All, CL7041.Contig1_All, CL7070.Contig1_All, CL709.Contig1_All, CL709.Contig2_All, CL7162.Contig1_All, CL7339.Contig1_All, CL7770.Contig1_All, CL7890.Contig1_All, CL7896.Contig1_All, CL8206.Contig1_All, CL8212.Contig1_All, CL83.Contig1_All, CL83.Contig3_All, CL83.Contig5_All, CL9152.Contig1_All, CL963.Contig1_All, Unigene2631_All, Unigene2693_All, Unigene3292_All, Unigene3781_All, Unigene4473_All, Unigene5383_All
48	Porphyrin and chlorophyll metabolism	CL10.Contig14_All, CL10.Contig30_All, CL10004.Contig1_All, CL10072.Contig2_All, CL10104.Contig1_All, CL10108.Contig1_All, CL10109.Contig1_All, CL10171.Contig1_All, CL10334.Contig1_All, CL10334.Contig2_All, CL10366.Contig1_All, CL10571.Contig1_All, CL10785.Contig1_All, CL10883.Contig1_All, CL11256.Contig1_All, CL11286.Contig1_All, CL11542.Contig1_All, CL11785.Contig1_All, CL11816.Contig1_All, CL11983.Contig1_All, CL12232.Contig1_All, CL12261.Contig1_All, CL12294.Contig1_All, CL12334.Contig1_All, CL12447.Contig1_All, CL12496.Contig1_All, CL12532.Contig1_All, CL12575.Contig1_All, CL12692.Contig1_All, CL12743.Contig1_All, CL12751.Contig1_All, CL12780.Contig1_All, CL12993.Contig1_All, CL13269.Contig1_All, CL13347.Contig1_All, CL13440.Contig1_All, CL14213.Contig1_All, CL14476.Contig1_All, CL14476.Contig2_All, CL14988.Contig1_All, CL15481.Contig1_All, CL15902.Contig1_All, CL16279.Contig1_All, CL1642.Contig1_All, CL17059.Contig1_All, CL1729.Contig1_All, CL1729.Contig2_All, CL1729.Contig3_All, CL1729.Contig4_All, CL17313.Contig1_All, CL17486.Contig1_All, CL17914.Contig1_All, CL17967.Contig1_All, CL18836.Contig1_All, CL18986.Contig1_All, CL19021.Contig1_All, CL19070.Contig1_All, CL20571.Contig1_All, CL20943.Contig1_All, CL21070.Contig1_All, CL21133.Contig1_All, CL21272.Contig1_All, CL21406.Contig1_All,

		CL21740.Contig1_All, CL21929.Contig1_All, CL23297.Contig1_All, CL24604.Contig1_All, CL2536.Contig1_All, CL2536.Contig2_All, CL25555.Contig1_All, CL25788.Contig1_All, CL2640.Contig1_All, CL26718.Contig1_All, CL26929.Contig1_All, CL27126.Contig1_All, CL2729.Contig1_All, CL27370.Contig1_All, CL27714.Contig1_All, CL27806.Contig1_All, CL27930.Contig1_All, CL3176.Contig1_All, CL3408.Contig1_All, CL3408.Contig2_All, CL3408.Contig3_All, CL3587.Contig1_All, CL3991.Contig1_All, CL3991.Contig2_All, CL4682.Contig1_All, CL4716.Contig1_All, CL4716.Contig2_All, CL4917.Contig1_All, CL5201.Contig1_All, CL5585.Contig1_All, CL576.Contig7_All, CL5877.Contig1_All, CL63.Contig5_All, CL63.Contig7_All, CL6744.Contig1_All, CL6840.Contig1_All, CL6867.Contig1_All, CL6946.Contig1_All, CL759.Contig3_All, CL759.Contig4_All, CL759.Contig5_All, CL759.Contig6_All, CL7786.Contig1_All, CL7799.Contig1_All, CL847.Contig1_All, CL847.Contig2_All, CL847.Contig3_All, CL847.Contig4_All, CL856.Contig2_All, CL8720.Contig1_All, CL881.Contig1_All, CL881.Contig2_All, CL881.Contig3_All, CL881.Contig4_All, CL9291.Contig1_All, CL9291.Contig2_All, Unigene1603_All, Unigene2129_All, Unigene4164_All, Unigene4178_All
49	Proteasome	CL10001.Contig1_All, CL10190.Contig1_All, CL10190.Contig2_All, CL10502.Contig1_All, CL10502.Contig2_All, CL10544.Contig1_All, CL10544.Contig2_All, CL10947.Contig1_All, CL11025.Contig1_All, CL11470.Contig1_All, CL11479.Contig1_All, CL11496.Contig1_All, CL11621.Contig1_All, CL11701.Contig1_All, CL11709.Contig1_All, CL11786.Contig1_All, CL11861.Contig1_All, CL11949.Contig1_All, CL11996.Contig1_All, CL12007.Contig1_All, CL12015.Contig1_All, CL12148.Contig1_All, CL12276.Contig1_All, CL12335.Contig1_All, CL12489.Contig1_All, CL12962.Contig1_All, CL13308.Contig1_All, CL13461.Contig1_All, CL13649.Contig1_All, CL13929.Contig1_All, CL14214.Contig1_All, CL14284.Contig1_All, CL14785.Contig1_All, CL15243.Contig1_All, CL15362.Contig1_All, CL15412.Contig1_All, CL15523.Contig1_All, CL15570.Contig1_All, CL15603.Contig1_All, CL16123.Contig1_All, CL16441.Contig1_All, CL17028.Contig1_All, CL17308.Contig1_All, CL1826.Contig1_All, CL1826.Contig2_All, CL1826.Contig3_All, CL1826.Contig4_All, CL18281.Contig1_All, CL19221.Contig1_All, CL19362.Contig1_All, CL19467.Contig1_All, CL20715.Contig1_All, CL20719.Contig1_All, CL20966.Contig1_All, CL21072.Contig1_All, CL21126.Contig1_All, CL2235.Contig1_All, CL2235.Contig2_All,

		CL2235.Contig3_All, CL2235.Contig4_All, CL23.Contig17_All, CL23.Contig18_All, CL23.Contig5_All, CL23.Contig7_All, CL23.Contig8_All, CL23.Contig9_All, CL2334.Contig1_All, CL2334.Contig2_All, CL24052.Contig1_All, CL2434.Contig1_All, CL2434.Contig2_All, CL2434.Contig3_All, CL25584.Contig1_All, CL26348.Contig1_All, CL26594.Contig1_All, CL27942.Contig1_All, CL3068.Contig1_All, CL3068.Contig2_All, CL31.Contig12_All, CL31.Contig13_All, CL31.Contig15_All, CL31.Contig16_All, CL31.Contig1_All, CL31.Contig2_All, CL31.Contig3_All, CL31.Contig6_All, CL3223.Contig1_All, CL3291.Contig1_All, CL3348.Contig1_All, CL3348.Contig2_All, CL3348.Contig3_All, CL4516.Contig1_All, CL4956.Contig1_All, CL4995.Contig1_All, CL5746.Contig1_All, CL6342.Contig1_All, CL6412.Contig1_All, CL658.Contig2_All, CL683.Contig1_All, CL718.Contig1_All, CL718.Contig2_All, CL718.Contig3_All, CL718.Contig4_All, CL72.Contig10_All, CL72.Contig14_All, CL72.Contig1_All, CL72.Contig4_All, CL72.Contig6_All, CL8027.Contig1_All, CL8493.Contig1_All, CL8794.Contig1_All, CL8942.Contig1_All, CL9272.Contig1_All, CL9498.Contig1_All, CL9498.Contig2_All, Unigene1132_All, Unigene1801_All, Unigene1924_All, Unigene2786_All, Unigene4090_All, Unigene5494_All, Unigene855_All
50	Carbon fixation in photosynthetic organisms	CL10026.Contig1_All, CL10048.Contig1_All, CL10073.Contig1_All, CL10145.Contig1_All, CL10202.Contig1_All, CL10216.Contig1_All, CL10224.Contig1_All, CL10238.Contig1_All, CL10568.Contig1_All, CL10600.Contig1_All, CL10924.Contig1_All, CL10994.Contig1_All, CL11480.Contig1_All, CL11562.Contig1_All, CL11570.Contig1_All, CL11926.Contig1_All, CL12074.Contig1_All, CL12091.Contig1_All, CL12126.Contig1_All, CL12133.Contig1_All, CL12183.Contig1_All, CL12184.Contig1_All, CL12226.Contig1_All, CL12283.Contig1_All, CL12301.Contig1_All, CL12342.Contig1_All, CL12429.Contig1_All, CL12487.Contig1_All, CL12514.Contig1_All, CL1272.Contig1_All, CL1272.Contig2_All, CL1272.Contig3_All, CL12850.Contig1_All, CL12909.Contig1_All, CL12942.Contig1_All, CL12957.Contig1_All, CL13019.Contig1_All, CL13179.Contig1_All, CL13246.Contig1_All, CL13277.Contig1_All, CL13326.Contig1_All, CL13408.Contig1_All, CL13492.Contig1_All, CL13593.Contig1_All, CL13835.Contig1_All, CL14669.Contig1_All, CL15447.Contig1_All, CL15475.Contig1_All, CL15538.Contig1_All, CL15778.Contig1_All, CL1615.Contig1_All, CL16428.Contig1_All, CL17949.Contig1_All, CL180.Contig2_All, CL180.Contig4_All, CL180.Contig7_All, CL18117.Contig1_All,

		CL18472.Contig1_All, CL18609.Contig1_All, CL19121.Contig1_All, CL2138.Contig1_All, CL22411.Contig1_All, CL22484.Contig1_All, CL22751.Contig1_All, CL23272.Contig1_All, CL25269.Contig1_All, CL2762.Contig1_All, CL288.Contig1_All, CL288.Contig2_All, CL288.Contig6_All, CL288.Contig7_All, CL3152.Contig1_All, CL3346.Contig1_All, CL3346.Contig2_All, CL3773.Contig1_All, CL395.Contig2_All, CL395.Contig3_All, CL395.Contig5_All, CL395.Contig6_All, CL4448.Contig1_All, CL4477.Contig1_All, CL4477.Contig2_All, CL4666.Contig1_All, CL4806.Contig1_All, CL5251.Contig1_All, CL5516.Contig1_All, CL593.Contig1_All, CL593.Contig2_All, CL593.Contig3_All, CL593.Contig4_All, CL593.Contig5_All, CL6305.Contig1_All, CL6607.Contig1_All, CL6722.Contig1_All, CL7069.Contig1_All, CL7280.Contig1_All, CL7537.Contig1_All, CL7537.Contig2_All, CL7537.Contig3_All, CL8138.Contig1_All, CL8222.Contig1_All, CL8386.Contig1_All, CL8831.Contig1_All, CL928.Contig1_All, CL928.Contig2_All, CL9846.Contig1_All, Unigene1242_All, Unigene2140_All, Unigene3432_All, Unigene3447_All, Unigene3654_All, Unigene3666_All, Unigene400_All, Unigene4277_All, Unigene441_All, Unigene4527_All, Unigene4732_All, Unigene4968_All, Unigene5134_All, Unigene5261_All, Unigene5389_All, Unigene5496_All
51	Galactose metabolism	CL10151.Contig1_All, CL10394.Contig1_All, CL108.Contig2_All, CL10870.Contig1_All, CL11324.Contig1_All, CL11462.Contig1_All, CL11843.Contig1_All, CL12385.Contig1_All, CL1277.Contig1_All, CL12810.Contig1_All, CL1288.Contig1_All, CL1288.Contig2_All, CL1288.Contig3_All, CL1288.Contig4_All, CL1304.Contig1_All, CL1304.Contig2_All, CL1304.Contig3_All, CL13284.Contig1_All, CL13540.Contig1_All, CL1428.Contig1_All, CL1428.Contig2_All, CL1428.Contig3_All, CL1428.Contig4_All, CL1428.Contig5_All, CL1428.Contig6_All, CL1428.Contig7_All, CL14498.Contig1_All, CL14672.Contig1_All, CL16302.Contig1_All, CL16368.Contig1_All, CL16555.Contig1_All, CL16919.Contig1_All, CL17196.Contig1_All, CL17291.Contig1_All, CL17291.Contig2_All, CL17309.Contig1_All, CL17482.Contig1_All, CL1750.Contig1_All, CL18355.Contig1_All, CL19037.Contig1_All, CL19062.Contig1_All, CL19267.Contig1_All, CL19408.Contig1_All, CL19717.Contig1_All, CL20816.Contig1_All, CL21780.Contig1_All, CL21853.Contig1_All, CL23651.Contig1_All, CL24913.Contig1_All, CL250.Contig1_All, CL250.Contig2_All, CL250.Contig3_All, CL250.Contig4_All, CL250.Contig5_All, CL250.Contig6_All, CL2571.Contig1_All, CL25863.Contig1_All, CL2942.Contig2_All,

		CL2942.Contig3_All, CL2952.Contig1_All, CL2993.Contig1_All, CL3064.Contig1_All, CL353.Contig1_All, CL353.Contig2_All, CL353.Contig3_All, CL353.Contig4_All, CL353.Contig5_All, CL3576.Contig1_All, CL3576.Contig2_All, CL3654.Contig1_All, CL3915.Contig1_All, CL399.Contig1_All, CL399.Contig2_All, CL399.Contig3_All, CL399.Contig4_All, CL399.Contig5_All, CL399.Contig6_All, CL4568.Contig1_All, CL5333.Contig1_All, CL560.Contig1_All, CL5621.Contig1_All, CL5638.Contig1_All, CL6.Contig23_All, CL6017.Contig1_All, CL621.Contig1_All, CL621.Contig2_All, CL6242.Contig1_All, CL6700.Contig1_All, CL6830.Contig1_All, CL7381.Contig1_All, CL741.Contig1_All, CL7581.Contig1_All, CL7772.Contig1_All, CL7909.Contig1_All, CL7909.Contig2_All, CL9164.Contig1_All, CL9611.Contig1_All, CL9739.Contig1_All, Unigene1686_All, Unigene1781_All, Unigene2001_All, Unigene2207_All, Unigene2263_All, Unigene2287_All, Unigene2545_All, Unigene278_All, Unigene3777_All, Unigene4184_All, Unigene4401_All, Unigene4438_All, Unigene4688_All, Unigene4790_All, Unigene4932_All, Unigene4933_All, Unigene515_All, Unigene5279_All, Unigene5322_All, Unigene664_All, Unigene705_All, Unigene865_All
52	Base excision repair	CL10368.Contig1_All, CL106.Contig1_All, CL106.Contig2_All, CL106.Contig3_All, CL106.Contig4_All, CL106.Contig5_All, CL106.Contig6_All, CL11511.Contig1_All, CL13080.Contig1_All, CL13180.Contig1_All, CL13252.Contig1_All, CL1342.Contig1_All, CL1342.Contig2_All, CL1342.Contig3_All, CL1342.Contig4_All, CL13565.Contig1_All, CL14276.Contig1_All, CL14332.Contig1_All, CL14507.Contig1_All, CL14580.Contig1_All, CL14689.Contig1_All, CL14981.Contig1_All, CL15039.Contig1_All, CL15080.Contig1_All, CL16014.Contig1_All, CL16830.Contig1_All, CL16893.Contig1_All, CL17120.Contig1_All, CL1717.Contig1_All, CL17819.Contig1_All, CL17834.Contig1_All, CL18146.Contig1_All, CL18672.Contig1_All, CL18719.Contig1_All, CL20009.Contig1_All, CL206.Contig3_All, CL206.Contig4_All, CL206.Contig6_All, CL206.Contig7_All, CL20923.Contig1_All, CL20934.Contig1_All, CL2129.Contig2_All, CL22420.Contig1_All, CL23087.Contig1_All, CL23151.Contig1_All, CL23279.Contig1_All, CL23363.Contig1_All, CL23528.Contig1_All, CL23570.Contig1_All, CL23834.Contig1_All, CL23873.Contig1_All, CL23924.Contig1_All, CL24063.Contig1_All, CL24519.Contig1_All, CL24615.Contig1_All, CL24635.Contig1_All, CL24714.Contig1_All, CL24839.Contig1_All, CL25044.Contig1_All, CL26264.Contig1_All, CL26303.Contig1_All, CL26387.Contig1_All, CL27901.Contig1_All, CL28030.Contig1_All, CL2941.Contig1_All, CL3164.Contig1_All, CL3305.Contig1_All, CL3367.Contig1_All,

		CL3367.Contig2_All, CL3471.Contig1_All, CL3746.Contig1_All, CL4131.Contig1_All, CL4131.Contig3_All, CL4205.Contig1_All, CL4461.Contig1_All, CL4617.Contig1_All, CL4773.Contig1_All, CL5226.Contig1_All, CL5960.Contig1_All, CL6483.Contig1_All, CL67.Contig5_All, CL6763.Contig1_All, CL6917.Contig1_All, CL6936.Contig1_All, CL7273.Contig1_All, CL7407.Contig1_All, CL7412.Contig2_All, CL8358.Contig1_All, CL8528.Contig1_All, CL8644.Contig1_All, CL8705.Contig1_All, CL8857.Contig2_All, CL9093.Contig1_All, CL9608.Contig1_All, CL9608.Contig2_All, CL983.Contig1_All, CL9924.Contig1_All, CL9987.Contig1_All, Unigene1386_All, Unigene2133_All, Unigene2200_All, Unigene234_All, Unigene2408_All, Unigene2585_All, Unigene266_All, Unigene2876_All, Unigene2_All, Unigene3275_All, Unigene3789_All, Unigene3978_All, Unigene4017_All, Unigene4038_All, Unigene434_All, Unigene4887_All, Unigene4904_All, Unigene5055_All, Unigene517_All, Unigene62_All, Unigene729_All, Unigene85_All
53	Citrate cycle (TCA cycle)	CL10162.Contig1_All, CL10202.Contig1_All, CL10215.Contig1_All, CL10458.Contig1_All, CL10655.Contig1_All, CL11330.Contig1_All, CL11738.Contig1_All, CL11754.Contig1_All, CL12301.Contig1_All, CL1234.Contig1_All, CL1234.Contig2_All, CL12429.Contig1_All, CL12722.Contig1_All, CL12753.Contig1_All, CL12865.Contig1_All, CL12909.Contig1_All, CL12957.Contig1_All, CL13019.Contig1_All, CL1339.Contig1_All, CL1339.Contig2_All, CL1339.Contig3_All, CL1339.Contig4_All, CL13408.Contig1_All, CL13569.Contig1_All, CL13661.Contig1_All, CL13923.Contig1_All, CL13956.Contig1_All, CL14038.Contig1_All, CL1460.Contig2_All, CL14933.Contig1_All, CL1502.Contig1_All, CL15253.Contig1_All, CL1562.Contig1_All, CL1562.Contig2_All, CL1562.Contig3_All, CL1562.Contig4_All, CL15778.Contig1_All, CL16214.Contig1_All, CL16457.Contig1_All, CL16676.Contig1_All, CL16719.Contig1_All, CL17189.Contig1_All, CL17689.Contig1_All, CL18226.Contig1_All, CL18244.Contig1_All, CL1865.Contig1_All, CL1874.Contig1_All, CL1874.Contig2_All, CL1874.Contig3_All, CL1874.Contig4_All, CL21826.Contig1_All, CL21909.Contig1_All, CL22305.Contig1_All, CL233.Contig2_All, CL233.Contig3_All, CL233.Contig5_All, CL26096.Contig1_All, CL262.Contig1_All, CL262.Contig2_All, CL262.Contig3_All, CL262.Contig4_All, CL262.Contig5_All, CL262.Contig6_All, CL262.Contig7_All, CL26742.Contig1_All, CL2745.Contig1_All, CL2745.Contig2_All, CL2762.Contig1_All, CL27816.Contig1_All, CL2950.Contig1_All,

		CL3505.Contig1_All, CL452.Contig1_All, CL452.Contig2_All, CL452.Contig3_All, CL452.Contig4_All, CL452.Contig5_All, CL4666.Contig1_All, CL4980.Contig1_All, CL5224.Contig1_All, CL5309.Contig1_All, CL5793.Contig1_All, CL5936.Contig1_All, CL5936.Contig2_All, CL641.Contig1_All, CL641.Contig2_All, CL641.Contig3_All, CL69.Contig1_All, CL69.Contig2_All, CL69.Contig3_All, CL69.Contig4_All, CL69.Contig5_All, CL69.Contig6_All, CL69.Contig7_All, CL69.Contig8_All, CL729.Contig1_All, CL729.Contig2_All, CL729.Contig3_All, CL729.Contig4_All, CL7700.Contig1_All, CL8138.Contig1_All, CL8223.Contig1_All, CL8307.Contig1_All, CL8747.Contig1_All, Unigene1120_All, Unigene1285_All, Unigene1773_All, Unigene198_All, Unigene2140_All, Unigene2970_All, Unigene3203_All, Unigene4109_All, Unigene4118_All, Unigene4153_All, Unigene4502_All, Unigene4507_All, Unigene4527_All, Unigene5473_All, Unigene5496_All, Unigene5509_All
54	alpha-Linolenic acid metabolism	CL10189.Contig1_All, CL10199.Contig1_All, CL10382.Contig1_All, CL10556.Contig1_All, CL10814.Contig1_All, CL1089.Contig1_All, CL1172.Contig1_All, CL1172.Contig2_All, CL12798.Contig1_All, CL12822.Contig1_All, CL12927.Contig1_All, CL12927.Contig2_All, CL12981.Contig1_All, CL13172.Contig1_All, CL13964.Contig1_All, CL14008.Contig1_All, CL1437.Contig1_All, CL1437.Contig2_All, CL1437.Contig3_All, CL1437.Contig4_All, CL1437.Contig5_All, CL14992.Contig1_All, CL15629.Contig1_All, CL16061.Contig1_All, CL16640.Contig1_All, CL1679.Contig1_All, CL17148.Contig1_All, CL17699.Contig1_All, CL1776.Contig1_All, CL1776.Contig2_All, CL1776.Contig3_All, CL1776.Contig4_All, CL1776.Contig5_All, CL1776.Contig6_All, CL17938.Contig1_All, CL18509.Contig1_All, CL18800.Contig1_All, CL19072.Contig1_All, CL19423.Contig1_All, CL20682.Contig1_All, CL20941.Contig1_All, CL211.Contig10_All, CL211.Contig11_All, CL211.Contig1_All, CL211.Contig2_All, CL211.Contig3_All, CL211.Contig4_All, CL211.Contig5_All, CL211.Contig6_All, CL211.Contig7_All, CL211.Contig8_All, CL211.Contig9_All, CL21134.Contig1_All, CL2115.Contig1_All, CL21576.Contig1_All, CL21677.Contig1_All, CL22623.Contig1_All, CL23069.Contig1_All, CL23575.Contig1_All, CL23945.Contig1_All, CL24045.Contig1_All, CL24050.Contig1_All, CL25435.Contig1_All, CL25532.Contig1_All, CL2630.Contig1_All, CL2630.Contig2_All, CL27480.Contig1_All, CL27907.Contig1_All, CL3031.Contig1_All, CL3039.Contig1_All, CL3116.Contig1_All, CL3509.Contig1_All, CL468.Contig1_All, CL468.Contig2_All, CL468.Contig3_All, CL5081.Contig1_All, CL5176.Contig1_All,

		CL5176.Contig2_All, CL53.Contig10_All, CL53.Contig11_All, CL53.Contig1_All, CL53.Contig3_All, CL53.Contig6_All, CL53.Contig7_All, CL53.Contig8_All, CL53.Contig9_All, CL5691.Contig1_All, CL5691.Contig2_All, CL5819.Contig1_All, CL6085.Contig1_All, CL6146.Contig1_All, CL627.Contig1_All, CL6421.Contig1_All, CL7005.Contig1_All, CL7124.Contig1_All, CL7150.Contig1_All, CL7255.Contig1_All, CL7978.Contig1_All, CL8008.Contig1_All, CL8094.Contig1_All, CL84.Contig12_All, CL84.Contig14_All, CL84.Contig1_All, CL84.Contig2_All, CL84.Contig7_All, CL84.Contig9_All, CL8496.Contig1_All, CL8935.Contig1_All, CL9688.Contig1_All, CL9842.Contig1_All, Unigene1230_All, Unigene1421_All, Unigene1432_All, Unigene2308_All, Unigene2602_All, Unigene327_All, Unigene3346_All, Unigene4745_All
55	Phenylalanine metabolism	CL10055.Contig1_All, CL10136.Contig1_All, CL10238.Contig1_All, CL10556.Contig1_All, CL10568.Contig1_All, CL10585.Contig1_All, CL1077.Contig1_All, CL10877.Contig1_All, CL10994.Contig1_All, CL11163.Contig1_All, CL12133.Contig1_All, CL12720.Contig1_All, CL12820.Contig1_All, CL12917.Contig1_All, CL13068.Contig1_All, CL13172.Contig1_All, CL13244.Contig1_All, CL13594.Contig1_All, CL13635.Contig1_All, CL13794.Contig1_All, CL13936.Contig1_All, CL14726.Contig1_All, CL14758.Contig1_All, CL14990.Contig1_All, CL15602.Contig1_All, CL15813.Contig1_All, CL16643.Contig1_All, CL17459.Contig1_All, CL17459.Contig2_All, CL17539.Contig1_All, CL17858.Contig1_All, CL18250.Contig1_All, CL18330.Contig1_All, CL1881.Contig1_All, CL1881.Contig2_All, CL1881.Contig3_All, CL2043.Contig1_All, CL21082.Contig1_All, CL22102.Contig1_All, CL22411.Contig1_All, CL23294.Contig1_All, CL23369.Contig1_All, CL23945.Contig1_All, CL24764.Contig1_All, CL25689.Contig1_All, CL25992.Contig1_All, CL26257.Contig1_All, CL26484.Contig1_All, CL26736.Contig1_All, CL26958.Contig1_All, CL26967.Contig1_All, CL27064.Contig1_All, CL27308.Contig1_All, CL281.Contig1_All, CL281.Contig2_All, CL281.Contig3_All, CL281.Contig4_All, CL281.Contig5_All, CL281.Contig6_All, CL281.Contig7_All, CL2876.Contig1_All, CL3321.Contig1_All, CL3321.Contig2_All, CL3321.Contig3_All, CL3788.Contig1_All, CL4024.Contig1_All, CL4055.Contig1_All, CL4129.Contig1_All, CL4743.Contig1_All, CL4846.Contig1_All, CL504.Contig1_All, CL504.Contig2_All, CL504.Contig3_All, CL504.Contig4_All, CL504.Contig5_All, CL5231.Contig1_All, CL5237.Contig1_All, CL5325.Contig1_All, CL5787.Contig1_All, CL6089.Contig1_All, CL6146.Contig1_All,

		CL6401.Contig1_All, CL6792.Contig1_All, CL6792.Contig2_All, CL7584.Contig1_All, CL8277.Contig1_All, CL8297.Contig1_All, CL8324.Contig1_All, CL8381.Contig1_All, CL8386.Contig1_All, CL8697.Contig1_All, CL8749.Contig1_All, CL908.Contig1_All, CL908.Contig2_All, CL908.Contig3_All, CL908.Contig4_All, CL9407.Contig1_All, CL9688.Contig1_All, CL9764.Contig1_All, Unigene1045_All, Unigene1694_All, Unigene2338_All, Unigene2961_All, Unigene2962_All, Unigene2963_All, Unigene2964_All, Unigene3071_All, Unigene3611_All, Unigene4302_All, Unigene4342_All, Unigene4973_All, Unigene4983_All, Unigene5236_All, Unigene5238_All, Unigene5262_All, Unigene5329_All, Unigene5525_All, Unigene938_All
56	Glycerolipid metabolism	CL10280.Contig1_All, CL10740.Contig1_All, CL10840.Contig1_All, CL11041.Contig1_All, CL11423.Contig1_All, CL11474.Contig1_All, CL11484.Contig1_All, CL12210.Contig1_All, CL12498.Contig1_All, CL13284.Contig1_All, CL13394.Contig1_All, CL1412.Contig1_All, CL1412.Contig2_All, CL1428.Contig1_All, CL1428.Contig2_All, CL1428.Contig3_All, CL1428.Contig4_All, CL1428.Contig5_All, CL1428.Contig6_All, CL1428.Contig7_All, CL16225.Contig1_All, CL16225.Contig2_All, CL16225.Contig3_All, CL16239.Contig1_All, CL16268.Contig1_All, CL16330.Contig1_All, CL16543.Contig1_All, CL16675.Contig1_All, CL1679.Contig1_All, CL16871.Contig1_All, CL18254.Contig1_All, CL18351.Contig1_All, CL20606.Contig1_All, CL20624.Contig1_All, CL20680.Contig1_All, CL20703.Contig1_All, CL20832.Contig1_All, CL20870.Contig1_All, CL20900.Contig1_All, CL21011.Contig1_All, CL21819.Contig1_All, CL23055.Contig1_All, CL23375.Contig1_All, CL23377.Contig1_All, CL23687.Contig1_All, CL24524.Contig1_All, CL26163.Contig1_All, CL26610.Contig1_All, CL2929.Contig1_All, CL3260.Contig1_All, CL3457.Contig1_All, CL3481.Contig1_All, CL3481.Contig2_All, CL3481.Contig3_All, CL3532.Contig1_All, CL3532.Contig2_All, CL3532.Contig3_All, CL3549.Contig1_All, CL3884.Contig1_All, CL3943.Contig1_All, CL4042.Contig1_All, CL4305.Contig1_All, CL4932.Contig1_All, CL5468.Contig1_All, CL5549.Contig1_All, CL5549.Contig2_All, CL5638.Contig1_All, CL5938.Contig1_All, CL5950.Contig1_All, CL6079.Contig1_All, CL6079.Contig2_All, CL6371.Contig1_All, CL6441.Contig1_All, CL6559.Contig1_All, CL674.Contig1_All, CL674.Contig2_All, CL674.Contig3_All, CL674.Contig4_All, CL674.Contig5_All, CL6830.Contig1_All, CL6973.Contig1_All, CL7244.Contig1_All, CL7244.Contig2_All,

		CL7557.Contig1_All, CL761.Contig1_All, CL761.Contig2_All, CL761.Contig3_All, CL761.Contig4_All, CL7724.Contig1_All, CL7994.Contig1_All, CL7997.Contig1_All, CL8001.Contig1_All, CL8239.Contig1_All, CL8339.Contig1_All, CL8393.Contig1_All, CL8932.Contig1_All, CL8988.Contig1_All, CL9106.Contig1_All, CL922.Contig3_All, CL946.Contig1_All, CL946.Contig2_All, CL946.Contig3_All, CL946.Contig4_All, CL9603.Contig1_All, CL9673.Contig1_All, Unigene2545_All, Unigene3777_All, Unigene4340_All, Unigene4417_All, Unigene4499_All, Unigene484_All, Unigene4908_All, Unigene4932_All, Unigene4933_All, Unigene5410_All, Unigene576_All
57	Glycine, serine and threonine metabolism	CL10162.Contig1_All, CL1051.Contig1_All, CL1051.Contig2_All, CL10672.Contig1_All, CL10992.Contig1_All, CL11.Contig17_All, CL11072.Contig1_All, CL11091.Contig1_All, CL11144.Contig1_All, CL11235.Contig1_All, CL11284.Contig1_All, CL11754.Contig1_All, CL11819.Contig1_All, CL11835.Contig1_All, CL11994.Contig1_All, CL12097.Contig1_All, CL1234.Contig1_All, CL1234.Contig2_All, CL12364.Contig1_All, CL12520.Contig1_All, CL12607.Contig1_All, CL12640.Contig1_All, CL12852.Contig1_All, CL12868.Contig1_All, CL12946.Contig1_All, CL13445.Contig1_All, CL13770.Contig1_All, CL13895.Contig1_All, CL13905.Contig1_All, CL13936.Contig1_All, CL14117.Contig1_All, CL14237.Contig1_All, CL14774.Contig1_All, CL15291.Contig1_All, CL15368.Contig1_All, CL15392.Contig1_All, CL15660.Contig1_All, CL16291.Contig1_All, CL16543.Contig1_All, CL16561.Contig1_All, CL16566.Contig1_All, CL16982.Contig1_All, CL17962.Contig1_All, CL18250.Contig1_All, CL1834.Contig3_All, CL19.Contig21_All, CL2043.Contig1_All, CL20666.Contig1_All, CL21082.Contig1_All, CL21372.Contig1_All, CL2143.Contig1_All, CL2227.Contig1_All, CL22366.Contig1_All, CL23375.Contig1_All, CL23525.Contig1_All, CL23818.Contig1_All, CL25053.Contig1_All, CL2554.Contig1_All, CL26700.Contig1_All, CL3041.Contig1_All, CL3805.Contig1_All, CL4140.Contig1_All, CL4168.Contig1_All, CL4675.Contig1_All, CL4850.Contig1_All, CL4850.Contig2_All, CL4850.Contig3_All, CL4987.Contig1_All, CL5445.Contig1_All, CL5661.Contig1_All, CL5709.Contig1_All, CL6401.Contig1_All, CL6753.Contig1_All, CL6753.Contig2_All, CL729.Contig1_All, CL729.Contig2_All, CL729.Contig3_All, CL729.Contig4_All, CL7521.Contig1_All, CL805.Contig1_All, CL805.Contig2_All, CL805.Contig3_All, CL805.Contig4_All, CL805.Contig5_All, CL8200.Contig1_All, CL8505.Contig1_All, CL8651.Contig1_All,

		CL8697.Contig1_All, CL8762.Contig1_All, CL8804.Contig1_All, CL8830.Contig1_All, CL8972.Contig1_All, CL908.Contig1_All, CL908.Contig2_All, CL908.Contig3_All, CL908.Contig4_All, CL94.Contig2_All, CL94.Contig3_All, CL94.Contig5_All, CL94.Contig6_All, CL94.Contig8_All, CL94.Contig9_All, CL9652.Contig1_All, CL9848.Contig1_All, Unigene1453_All, Unigene2167_All, Unigene2406_All, Unigene3611_All, Unigene4417_All, Unigene4420_All, Unigene4727_All, Unigene5473_All, Unigene5509_All
58	Pentose phosphate pathway	CL10026.Contig1_All, CL10151.Contig1_All, CL10216.Contig1_All, CL10224.Contig1_All, CL10347.Contig1_All, CL10870.Contig1_All, CL11129.Contig1_All, CL11224.Contig1_All, CL11370.Contig1_All, CL11396.Contig1_All, CL12074.Contig1_All, CL12183.Contig1_All, CL12184.Contig1_All, CL12202.Contig1_All, CL12810.Contig1_All, CL12942.Contig1_All, CL13175.Contig1_All, CL13179.Contig1_All, CL13246.Contig1_All, CL13326.Contig1_All, CL13540.Contig1_All, CL14182.Contig1_All, CL14588.Contig1_All, CL15447.Contig1_All, CL15538.Contig1_All, CL1568.Contig1_All, CL1568.Contig2_All, CL1568.Contig3_All, CL1568.Contig4_All, CL16372.Contig1_All, CL17291.Contig1_All, CL17291.Contig2_All, CL17793.Contig1_All, CL180.Contig2_All, CL180.Contig4_All, CL180.Contig7_All, CL18472.Contig1_All, CL19037.Contig1_All, CL19121.Contig1_All, CL21853.Contig1_All, CL22352.Contig1_All, CL22484.Contig1_All, CL22751.Contig1_All, CL23445.Contig1_All, CL24022.Contig1_All, CL2498.Contig1_All, CL2655.Contig1_All, CL27120.Contig1_All, CL27289.Contig1_All, CL2942.Contig2_All, CL2942.Contig3_All, CL353.Contig1_All, CL353.Contig2_All, CL353.Contig3_All, CL353.Contig4_All, CL353.Contig5_All, CL3915.Contig1_All, CL4026.Contig1_All, CL4183.Contig1_All, CL4568.Contig1_All, CL4954.Contig1_All, CL4954.Contig2_All, CL5333.Contig1_All, CL5516.Contig1_All, CL6.Contig26_All, CL6051.Contig1_All, CL61.Contig10_All, CL61.Contig1_All, CL61.Contig2_All, CL61.Contig3_All, CL61.Contig4_All, CL61.Contig5_All, CL61.Contig6_All, CL61.Contig7_All, CL61.Contig8_All, CL61.Contig9_All, CL6172.Contig1_All, CL6233.Contig1_All, CL68.Contig1_All, CL68.Contig3_All, CL68.Contig4_All, CL68.Contig6_All, CL68.Contig8_All, CL6861.Contig1_All, CL7069.Contig1_All, CL7260.Contig1_All, CL7280.Contig1_All, CL7348.Contig1_All, CL7732.Contig1_All, CL7733.Contig1_All, CL7974.Contig1_All, CL8222.Contig1_All, CL8545.Contig1_All, CL9383.Contig1_All, CL9383.Contig2_All, Unigene1599_All, Unigene1781_All, Unigene2263_All, Unigene2530_All, Unigene2637_All, Unigene3447_All, Unigene4184_All,

		Unigene4438_All, Unigene4688_All, Unigene4732_All, Unigene4968_All, Unigene5134_All, Unigene515_All, Unigene5522_All, Unigene657_All, Unigene865_All
59	Ascorbate and aldarate metabolism	CL10667.Contig1_All, CL10690.Contig1_All, CL10740.Contig1_All, CL11504.Contig1_All, CL11655.Contig1_All, CL11914.Contig1_All, CL11928.Contig1_All, CL12316.Contig1_All, CL12340.Contig1_All, CL12498.Contig1_All, CL12556.Contig1_All, CL12848.Contig1_All, CL12911.Contig1_All, CL12963.Contig1_All, CL13016.Contig1_All, CL13525.Contig1_All, CL1412.Contig1_All, CL1412.Contig2_All, CL14532.Contig1_All, CL1479.Contig1_All, CL1479.Contig2_All, CL1479.Contig3_All, CL1479.Contig4_All, CL1479.Contig5_All, CL15140.Contig1_All, CL16239.Contig1_All, CL16540.Contig1_All, CL16543.Contig1_All, CL1667.Contig1_All, CL16871.Contig1_All, CL17393.Contig1_All, CL17412.Contig1_All, CL17880.Contig1_All, CL18233.Contig1_All, CL185.Contig3_All, CL185.Contig7_All, CL185.Contig8_All, CL18785.Contig1_All, CL18822.Contig1_All, CL198.Contig1_All, CL198.Contig2_All, CL198.Contig3_All, CL198.Contig4_All, CL198.Contig5_All, CL198.Contig6_All, CL198.Contig7_All, CL198.Contig8_All, CL198.Contig9_All, CL19934.Contig1_All, CL2033.Contig1_All, CL21183.Contig1_All, CL21299.Contig1_All, CL21323.Contig1_All, CL21851.Contig1_All, CL22027.Contig1_All, CL22106.Contig1_All, CL22539.Contig1_All, CL226.Contig3_All, CL226.Contig5_All, CL226.Contig7_All, CL23375.Contig1_All, CL23687.Contig1_All, CL24608.Contig1_All, CL25005.Contig1_All, CL25898.Contig1_All, CL27060.Contig1_All, CL27328.Contig1_All, CL2903.Contig1_All, CL2929.Contig1_All, CL2980.Contig1_All, CL3328.Contig1_All, CL3398.Contig1_All, CL3457.Contig1_All, CL3532.Contig1_All, CL3532.Contig3_All, CL3734.Contig1_All, CL3826.Contig1_All, CL4775.Contig1_All, CL4932.Contig1_All, CL5225.Contig1_All, CL5271.Contig1_All, CL5549.Contig1_All, CL5549.Contig2_All, CL6058.Contig1_All, CL6058.Contig2_All, CL6145.Contig1_All, CL6371.Contig1_All, CL7244.Contig2_All, CL757.Contig1_All, CL757.Contig2_All, CL757.Contig3_All, CL757.Contig4_All, CL8393.Contig1_All, CL8690.Contig1_All, CL8845.Contig1_All, CL9106.Contig1_All, CL9202.Contig1_All, CL9660.Contig1_All, Unigene3868_All, Unigene4336_All, Unigene4417_All, Unigene4523_All, Unigene4773_All, Unigene5126_All, Unigene5143_All, Unigene5214_All, Unigene544_All
		CL1.Contig101_All, CL1.Contig110_All, CL1.Contig39_All, CL1.Contig81_All, CL10107.Contig1_All,

60	Nitrogen metabolism	CL10161.Contig1_All, CL10375.Contig1_All, CL10611.Contig1_All, CL10697.Contig1_All, CL10788.Contig1_All, CL10816.Contig1_All, CL1086.Contig1_All, CL1086.Contig2_All, CL11015.Contig1_All, CL11392.Contig1_All, CL11461.Contig1_All, CL11513.Contig1_All, CL11519.Contig1_All, CL11575.Contig1_All, CL11664.Contig1_All, CL11664.Contig2_All, CL11728.Contig1_All, CL12607.Contig1_All, CL12906.Contig1_All, CL13278.Contig1_All, CL13384.Contig1_All, CL13384.Contig2_All, CL13648.Contig1_All, CL13789.Contig1_All, CL13790.Contig1_All, CL14382.Contig1_All, CL14547.Contig1_All, CL15005.Contig1_All, CL15335.Contig1_All, CL1552.Contig1_All, CL15537.Contig1_All, CL1578.Contig1_All, CL1578.Contig2_All, CL1578.Contig3_All, CL1578.Contig4_All, CL16056.Contig1_All, CL16172.Contig1_All, CL16534.Contig1_All, CL1834.Contig3_All, CL18544.Contig1_All, CL1881.Contig1_All, CL1881.Contig2_All, CL1881.Contig3_All, CL19052.Contig1_All, CL20185.Contig1_All, CL21157.Contig1_All, CL22010.Contig1_All, CL22065.Contig1_All, CL22077.Contig1_All, CL22954.Contig1_All, CL22992.Contig1_All, CL23024.Contig1_All, CL2502.Contig1_All, CL25719.Contig1_All, CL2594.Contig1_All, CL2849.Contig1_All, CL2849.Contig2_All, CL411.Contig1_All, CL4177.Contig1_All, CL4647.Contig1_All, CL4647.Contig3_All, CL4944.Contig1_All, CL51.Contig10_All, CL51.Contig11_All, CL51.Contig12_All, CL51.Contig14_All, CL51.Contig15_All, CL51.Contig1_All, CL51.Contig2_All, CL51.Contig3_All, CL51.Contig4_All, CL51.Contig5_All, CL51.Contig6_All, CL51.Contig8_All, CL51.Contig9_All, CL561.Contig1_All, CL561.Contig2_All, CL561.Contig3_All, CL561.Contig4_All, CL561.Contig5_All, CL561.Contig6_All, CL561.Contig7_All, CL561.Contig8_All, CL6395.Contig1_All, CL6862.Contig1_All, CL8499.Contig1_All, CL9501.Contig1_All, Unigene1712_All, Unigene3782_All, Unigene4156_All, Unigene4158_All, Unigene4159_All, Unigene4161_All, Unigene4162_All, Unigene4973_All, Unigene4983_All, Unigene5236_All, Unigene5317_All, Unigene5498_All
		CL10134.Contig1_All, CL10403.Contig1_All, CL1042.Contig1_All, CL10452.Contig1_All, CL10481.Contig1_All, CL1154.Contig1_All, CL11594.Contig1_All, CL11964.Contig1_All, CL11970.Contig1_All, CL12419.Contig1_All, CL12526.Contig1_All, CL13756.Contig1_All, CL13925.Contig1_All, CL14379.Contig1_All, CL14555.Contig1_All, CL14772.Contig1_All, CL14927.Contig1_All, CL15237.Contig1_All, CL15809.Contig1_All, CL1618.Contig1_All, CL1618.Contig2_All, CL16449.Contig1_All,

61	N-Glycan biosynthesis	CL16790.Contig1_All, CL1779.Contig3_All, CL18789.Contig1_All, CL19341.Contig1_All, CL19368.Contig1_All, CL19512.Contig1_All, CL19570.Contig1_All, CL20564.Contig1_All, CL20614.Contig1_All, CL20640.Contig1_All, CL21015.Contig1_All, CL2158.Contig2_All, CL2203.Contig1_All, CL22129.Contig1_All, CL22682.Contig1_All, CL23243.Contig1_All, CL23718.Contig1_All, CL24532.Contig1_All, CL25603.Contig1_All, CL2618.Contig1_All, CL2618.Contig2_All, CL274.Contig2_All, CL2831.Contig1_All, CL2831.Contig2_All, CL3064.Contig1_All, CL3201.Contig1_All, CL334.Contig1_All, CL334.Contig2_All, CL334.Contig3_All, CL3437.Contig1_All, CL3465.Contig1_All, CL3530.Contig1_All, CL3725.Contig1_All, CL39.Contig10_All, CL39.Contig12_All, CL39.Contig14_All, CL39.Contig2_All, CL39.Contig3_All, CL39.Contig4_All, CL39.Contig5_All, CL39.Contig6_All, CL39.Contig8_All, CL39.Contig9_All, CL4410.Contig1_All, CL5098.Contig1_All, CL5220.Contig1_All, CL5286.Contig1_All, CL5684.Contig1_All, CL5935.Contig1_All, CL5957.Contig1_All, CL626.Contig1_All, CL644.Contig1_All, CL644.Contig2_All, CL644.Contig3_All, CL644.Contig4_All, CL644.Contig5_All, CL644.Contig6_All, CL644.Contig7_All, CL644.Contig8_All, CL6485.Contig1_All, CL6509.Contig1_All, CL7104.Contig1_All, CL7375.Contig1_All, CL7382.Contig1_All, CL7874.Contig1_All, CL7917.Contig1_All, CL7983.Contig1_All, CL8491.Contig1_All, CL8491.Contig2_All, CL8872.Contig1_All, CL922.Contig3_All, Unigene1416_All, Unigene1762_All, Unigene1978_All, Unigene2341_All, Unigene2826_All, Unigene3293_All, Unigene343_All, Unigene4401_All, Unigene5365_All
		CL10211.Contig1_All, CL10526.Contig1_All, CL1054.Contig1_All, CL1054.Contig2_All, CL1054.Contig3_All, CL10736.Contig1_All, CL10749.Contig1_All, CL10780.Contig1_All, CL10829.Contig1_All, CL11099.Contig1_All, CL11416.Contig1_All, CL11555.Contig1_All, CL11629.Contig1_All, CL11653.Contig1_All, CL11729.Contig1_All, CL11737.Contig1_All, CL11883.Contig1_All, CL11973.Contig1_All, CL12028.Contig1_All, CL12124.Contig1_All, CL12354.Contig1_All, CL12369.Contig1_All, CL12529.Contig1_All, CL12996.Contig1_All, CL12996.Contig2_All, CL13272.Contig1_All, CL13436.Contig1_All, CL13712.Contig1_All, CL13739.Contig1_All, CL13739.Contig2_All, CL13819.Contig1_All, CL13995.Contig1_All, CL14083.Contig1_All, CL14141.Contig1_All, CL14261.Contig1_All, CL14267.Contig1_All, CL14293.Contig1_All, CL14367.Contig1_All, CL14385.Contig1_All, CL14468.Contig1_All, CL14792.Contig1_All, CL14794.Contig1_All,

62	Photosynthesis	CL14903.Contig1_All, CL14903.Contig2_All, CL1494.Contig1_All, CL1494.Contig2_All, CL1494.Contig3_All, CL1494.Contig4_All, CL14997.Contig1_All, CL15030.Contig1_All, CL15035.Contig1_All, CL15077.Contig1_All, CL15111.Contig1_All, CL15191.Contig1_All, CL15300.Contig1_All, CL15403.Contig1_All, CL15436.Contig1_All, CL15518.Contig1_All, CL15686.Contig1_All, CL17263.Contig1_All, CL18211.Contig1_All, CL18868.Contig1_All, CL18904.Contig1_All, CL197.Contig1_All, CL197.Contig2_All, CL1983.Contig1_All, CL20766.Contig1_All, CL20893.Contig1_All, CL21399.Contig1_All, CL22325.Contig1_All, CL23768.Contig1_All, CL2475.Contig1_All, CL2682.Contig1_All, CL3427.Contig1_All, CL3538.Contig1_All, CL3622.Contig1_All, CL3799.Contig1_All, CL3799.Contig2_All, CL4268.Contig1_All, CL4275.Contig1_All, CL4874.Contig1_All, CL5072.Contig1_All, CL52.Contig7_All, CL6178.Contig1_All, CL6178.Contig2_All, CL6507.Contig1_All, CL6521.Contig1_All, CL6879.Contig1_All, CL6879.Contig2_All, CL6976.Contig1_All, CL8646.Contig1_All, CL9026.Contig1_All, CL9236.Contig1_All, CL928.Contig3_All, CL9921.Contig1_All, Unigene1786_All, Unigene2829_All, Unigene3075_All, Unigene3669_All
63	Alanine, aspartate and glutamate metabolism	CL10115.Contig1_All, CL10136.Contig1_All, CL10212.Contig1_All, CL10238.Contig1_All, CL10568.Contig1_All, CL10788.Contig1_All, CL10816.Contig1_All, CL1086.Contig1_All, CL1086.Contig2_All, CL10994.Contig1_All, CL11461.Contig1_All, CL11513.Contig1_All, CL11575.Contig1_All, CL12133.Contig1_All, CL12156.Contig1_All, CL12346.Contig1_All, CL12520.Contig1_All, CL12611.Contig1_All, CL12868.Contig1_All, CL12906.Contig1_All, CL13384.Contig1_All, CL13384.Contig2_All, CL13466.Contig1_All, CL13789.Contig1_All, CL14164.Contig1_All, CL1455.Contig1_All, CL1455.Contig2_All, CL1455.Contig3_All, CL15401.Contig1_All, CL1552.Contig1_All, CL1578.Contig1_All, CL1578.Contig2_All, CL1578.Contig3_All, CL1578.Contig4_All, CL16056.Contig1_All, CL16172.Contig1_All, CL16428.Contig1_All, CL16534.Contig1_All, CL17548.Contig1_All, CL18491.Contig1_All, CL18544.Contig1_All, CL1893.Contig1_All, CL22010.Contig1_All, CL22065.Contig1_All, CL2207.Contig1_All, CL22077.Contig1_All, CL22411.Contig1_All, CL23024.Contig1_All, CL24622.Contig1_All, CL2483.Contig1_All, CL25845.Contig1_All, CL2594.Contig1_All, CL2724.Contig1_All, CL27261.Contig1_All,

		CL2796.Contig1_All, CL2796.Contig2_All, CL2796.Contig3_All, CL2796.Contig4_All, CL3618.Contig1_All, CL4161.Contig1_All, CL4177.Contig1_All, CL4647.Contig1_All, CL4647.Contig3_All, CL4806.Contig1_All, CL4951.Contig1_All, CL51.Contig10_All, CL51.Contig11_All, CL51.Contig12_All, CL51.Contig14_All, CL51.Contig15_All, CL51.Contig17_All, CL51.Contig1_All, CL51.Contig2_All, CL51.Contig3_All, CL51.Contig4_All, CL51.Contig5_All, CL51.Contig6_All, CL51.Contig8_All, CL51.Contig9_All, CL5138.Contig1_All, CL593.Contig1_All, CL593.Contig2_All, CL593.Contig3_All, CL593.Contig4_All, CL593.Contig5_All, CL6371.Contig1_All, CL6753.Contig1_All, CL6753.Contig2_All, CL6805.Contig1_All, CL7561.Contig1_All, CL8386.Contig1_All, CL8662.Contig1_All, CL8762.Contig1_All, CL8830.Contig1_All, CL8972.Contig1_All, Unigene1712_All, Unigene2687_All, Unigene3432_All, Unigene831_All
64	Protein export	CL10412.Contig1_All, CL10427.Contig1_All, CL10581.Contig1_All, CL11545.Contig1_All, CL11548.Contig1_All, CL11876.Contig1_All, CL12581.Contig1_All, CL13061.Contig1_All, CL1332.Contig1_All, CL1332.Contig2_All, CL13323.Contig1_All, CL13606.Contig1_All, CL13730.Contig1_All, CL13841.Contig1_All, CL14343.Contig1_All, CL1450.Contig1_All, CL14822.Contig1_All, CL15017.Contig1_All, CL15017.Contig2_All, CL15151.Contig1_All, CL15197.Contig1_All, CL1563.Contig1_All, CL1563.Contig2_All, CL1563.Contig3_All, CL16067.Contig1_All, CL16099.Contig1_All, CL16099.Contig2_All, CL16334.Contig1_All, CL16522.Contig1_All, CL17339.Contig1_All, CL17339.Contig2_All, CL17718.Contig1_All, CL17854.Contig1_All, CL18905.Contig1_All, CL18984.Contig1_All, CL19219.Contig1_All, CL20351.Contig1_All, CL20812.Contig1_All, CL21006.Contig1_All, CL21790.Contig1_All, CL22107.Contig1_All, CL23663.Contig1_All, CL23761.Contig1_All, CL2403.Contig1_All, CL24934.Contig1_All, CL2518.Contig1_All, CL2548.Contig2_All, CL2548.Contig3_All, CL2626.Contig1_All, CL26804.Contig1_All, CL27153.Contig1_All, CL27601.Contig1_All, CL2795.Contig1_All, CL2795.Contig2_All, CL2795.Contig3_All, CL27953.Contig1_All, CL2856.Contig1_All, CL3358.Contig1_All, CL3423.Contig1_All, CL4271.Contig1_All, CL54.Contig10_All, CL54.Contig11_All, CL54.Contig12_All, CL54.Contig13_All, CL54.Contig14_All, CL54.Contig15_All, CL54.Contig5_All, CL54.Contig6_All, CL54.Contig8_All, CL54.Contig9_All, CL5508.Contig1_All, CL5898.Contig1_All, CL6390.Contig1_All, CL6618.Contig1_All, CL7340.Contig1_All, CL7511.Contig1_All,

		CL7933.Contig1_All, CL8355.Contig1_All, CL8571.Contig1_All, CL8637.Contig1_All, CL8861.Contig1_All, CL9376.Contig1_All, CL9376.Contig2_All, CL9441.Contig1_All, CL9441.Contig2_All, CL954.Contig1_All, CL954.Contig2_All, Unigene1252_All, Unigene1259_All, Unigene1369_All, Unigene1747_All, Unigene3049_All, Unigene3501_All, Unigene3754_All, Unigene4492_All, Unigene5237_All, Unigene5490_All, Unigene5493_All
65	Fatty acid metabolism	CL10057.Contig1_All, CL10068.Contig1_All, CL10541.Contig1_All, CL10551.Contig1_All, CL10687.Contig1_All, CL10740.Contig1_All, CL10814.Contig1_All, CL12067.Contig1_All, CL12498.Contig1_All, CL12674.Contig1_All, CL1282.Contig1_All, CL12822.Contig1_All, CL12981.Contig1_All, CL13023.Contig1_All, CL13073.Contig1_All, CL13321.Contig1_All, CL13548.Contig1_All, CL1412.Contig1_All, CL1412.Contig2_All, CL14294.Contig1_All, CL15629.Contig1_All, CL16239.Contig1_All, CL16543.Contig1_All, CL16640.Contig1_All, CL16795.Contig1_All, CL16871.Contig1_All, CL1728.Contig1_All, CL1728.Contig2_All, CL17699.Contig1_All, CL19567.Contig1_All, CL2.Contig25_All, CL2005.Contig1_All, CL21134.Contig1_All, CL2115.Contig1_All, CL21565.Contig1_All, CL23375.Contig1_All, CL23423.Contig1_All, CL23687.Contig1_All, CL24211.Contig1_All, CL24508.Contig1_All, CL25407.Contig1_All, CL25435.Contig1_All, CL2670.Contig1_All, CL2929.Contig1_All, CL3039.Contig1_All, CL3049.Contig1_All, CL3448.Contig1_All, CL3448.Contig2_All, CL3457.Contig1_All, CL3545.Contig1_All, CL4511.Contig1_All, CL4549.Contig1_All, CL486.Contig1_All, CL486.Contig2_All, CL486.Contig3_All, CL486.Contig4_All, CL5081.Contig1_All, CL53.Contig10_All, CL53.Contig11_All, CL53.Contig1_All, CL53.Contig3_All, CL53.Contig6_All, CL53.Contig7_All, CL53.Contig8_All, CL53.Contig9_All, CL5549.Contig1_All, CL5549.Contig2_All, CL5691.Contig1_All, CL5691.Contig2_All, CL5819.Contig1_All, CL609.Contig1_All, CL609.Contig2_All, CL609.Contig3_All, CL609.Contig4_All, CL6091.Contig1_All, CL6371.Contig1_All, CL6637.Contig1_All, CL7401.Contig1_All, CL7670.Contig1_All, CL7978.Contig1_All, CL8052.Contig1_All, CL8496.Contig1_All, CL8935.Contig1_All, CL9106.Contig1_All, CL9174.Contig1_All, CL9429.Contig1_All, Unigene2308_All, Unigene2602_All, Unigene2872_All, Unigene3102_All, Unigene327_All, Unigene363_All, Unigene3918_All, Unigene3983_All, Unigene4114_All, Unigene4417_All, Unigene4745_All, Unigene920_All
		CL13478.Contig1_All, CL14114.Contig1_All, CL16136.Contig1_All, CL16342.Contig1_All,

66	Natural killer cell mediated cytotoxicity	CL16373.Contig1_All, CL1639.Contig1_All, CL164.Contig1_All, CL164.Contig2_All, CL164.Contig3_All, CL164.Contig4_All, CL164.Contig5_All, CL16416.Contig1_All, CL16680.Contig1_All, CL1669.Contig1_All, CL1669.Contig2_All, CL17270.Contig1_All, CL1783.Contig1_All, CL1783.Contig2_All, CL1783.Contig3_All, CL17906.Contig1_All, CL18006.Contig1_All, CL18619.Contig1_All, CL18777.Contig1_All, CL1885.Contig1_All, CL1885.Contig2_All, CL19165.Contig1_All, CL20917.Contig1_All, CL20978.Contig1_All, CL212.Contig4_All, CL21254.Contig1_All, CL220.Contig3_All, CL22347.Contig1_All, CL22742.Contig1_All, CL2421.Contig1_All, CL2421.Contig2_All, CL25235.Contig1_All, CL2622.Contig1_All, CL2622.Contig2_All, CL27238.Contig1_All, CL27827.Contig1_All, CL2790.Contig1_All, CL2790.Contig2_All, CL3254.Contig1_All, CL3282.Contig1_All, CL3282.Contig2_All, CL33.Contig10_All, CL33.Contig11_All, CL33.Contig13_All, CL33.Contig14_All, CL33.Contig1_All, CL33.Contig2_All, CL33.Contig3_All, CL33.Contig4_All, CL33.Contig5_All, CL33.Contig6_All, CL33.Contig7_All, CL33.Contig9_All, CL3550.Contig1_All, CL3550.Contig2_All, CL380.Contig1_All, CL380.Contig2_All, CL380.Contig3_All, CL380.Contig4_All, CL380.Contig5_All, CL4.Contig10_All, CL4.Contig11_All, CL4.Contig13_All, CL4.Contig21_All, CL4.Contig24_All, CL4467.Contig1_All, CL4517.Contig1_All, CL4746.Contig1_All, CL5088.Contig1_All, CL5088.Contig2_All, CL5848.Contig1_All, CL6297.Contig1_All, CL6604.Contig1_All, CL7152.Contig1_All, CL7292.Contig1_All, CL7450.Contig1_All, CL8139.Contig1_All, CL8208.Contig1_All, CL8209.Contig1_All, CL8560.Contig1_All, CL8648.Contig1_All, CL9003.Contig1_All, CL9331.Contig1_All, CL9331.Contig2_All, CL9439.Contig1_All, CL9710.Contig1_All, CL9756.Contig1_All, Unigene2027_All, Unigene2116_All, Unigene3845_All, Unigene4761_All
		CL10026.Contig1_All, CL10072.Contig2_All, CL10151.Contig1_All, CL10155.Contig1_All, CL10198.Contig2_All, CL10216.Contig1_All, CL10347.Contig1_All, CL10445.Contig1_All, CL10870.Contig1_All, CL10924.Contig1_All, CL11022.Contig1_All, CL11866.Contig1_All, CL12134.Contig1_All, CL12183.Contig1_All, CL12184.Contig1_All, CL12937.Contig1_All, CL12942.Contig1_All, CL13179.Contig1_All, CL13475.Contig1_All, CL13540.Contig1_All, CL13590.Contig1_All, CL13593.Contig1_All, CL15447.Contig1_All, CL16555.Contig1_All, CL1671.Contig1_All, CL180.Contig2_All, CL180.Contig4_All, CL180.Contig7_All, CL19037.Contig1_All, CL19121.Contig1_All, CL21780.Contig1_All,

67	Fructose and mannose metabolism	CL21853.Contig1_All, CL22514.Contig1_All, CL22751.Contig1_All, CL2301.Contig1_All, CL23202.Contig1_All, CL250.Contig1_All, CL250.Contig2_All, CL250.Contig3_All, CL250.Contig4_All, CL250.Contig5_All, CL250.Contig6_All, CL2746.Contig1_All, CL353.Contig1_All, CL353.Contig2_All, CL353.Contig3_All, CL353.Contig4_All, CL353.Contig5_All, CL3582.Contig1_All, CL3915.Contig1_All, CL3991.Contig1_All, CL4568.Contig1_All, CL4716.Contig1_All, CL4716.Contig2_All, CL4821.Contig1_All, CL5363.Contig1_All, CL5516.Contig1_All, CL6018.Contig1_All, CL6662.Contig1_All, CL6700.Contig1_All, CL6744.Contig1_All, CL7142.Contig1_All, CL7195.Contig2_All, CL7280.Contig1_All, CL7325.Contig1_All, CL7381.Contig1_All, CL7438.Contig1_All, CL759.Contig1_All, CL759.Contig2_All, CL759.Contig3_All, CL759.Contig4_All, CL759.Contig5_All, CL759.Contig6_All, CL7772.Contig1_All, CL828.Contig1_All, CL828.Contig2_All, CL828.Contig3_All, CL828.Contig4_All, CL828.Contig5_All, CL8300.Contig1_All, CL851.Contig4_All, CL8663.Contig1_All, CL8695.Contig1_All, CL8720.Contig1_All, Unigene1017_All, Unigene1781_All, Unigene1878_All, Unigene2263_All, Unigene2873_All, Unigene4184_All, Unigene4438_All, Unigene4688_All, Unigene4732_All, Unigene515_All, Unigene865_All
68	Mismatch repair	CL1.Contig132_All, CL1.Contig78_All, CL10900.Contig1_All, CL12119.Contig1_All, CL12181.Contig1_All, CL12987.Contig1_All, CL13177.Contig1_All, CL14493.Contig1_All, CL14634.Contig1_All, CL15256.Contig1_All, CL15541.Contig1_All, CL16648.Contig1_All, CL16686.Contig1_All, CL17254.Contig1_All, CL17462.Contig1_All, CL17567.Contig1_All, CL17736.Contig1_All, CL17834.Contig1_All, CL1828.Contig1_All, CL18296.Contig1_All, CL1870.Contig2_All, CL18753.Contig1_All, CL18791.Contig1_All, CL18879.Contig1_All, CL19254.Contig1_All, CL19828.Contig1_All, CL19845.Contig1_All, CL20418.Contig1_All, CL20934.Contig1_All, CL21615.Contig1_All, CL22308.Contig1_All, CL22419.Contig1_All, CL2255.Contig1_All, CL22734.Contig1_All, CL2317.Contig1_All, CL23671.Contig1_All, CL23708.Contig1_All, CL24042.Contig1_All, CL24615.Contig1_All, CL24644.Contig1_All, CL25044.Contig1_All, CL25294.Contig1_All, CL25417.Contig1_All, CL25619.Contig1_All, CL2562.Contig2_All, CL25951.Contig1_All, CL26122.Contig1_All, CL2646.Contig1_All, CL27080.Contig1_All, CL27138.Contig1_All, CL27160.Contig1_All, CL27375.Contig1_All, CL2773.Contig1_All, CL27845.Contig1_All, CL28103.Contig1_All, CL3164.Contig1_All, CL3514.Contig2_All, CL4617.Contig1_All, CL5507.Contig1_All, CL564.Contig1_All,

		CL5912.Contig1_All, CL6076.Contig1_All, CL6143.Contig1_All, CL6169.Contig1_All, CL6483.Contig1_All, CL6508.Contig1_All, CL7168.Contig1_All, CL7433.Contig1_All, CL7610.Contig1_All, CL7610.Contig2_All, CL8259.Contig1_All, CL8317.Contig1_All, CL8644.Contig1_All, CL8857.Contig2_All, CL9317.Contig1_All, CL9430.Contig1_All, CL9564.Contig1_All, CL9727.Contig1_All, CL9755.Contig1_All, CL9924.Contig1_All, CL9987.Contig1_All, Unigene1386_All, Unigene1585_All, Unigene2408_All, Unigene2585_All, Unigene2876_All, Unigene3267_All, Unigene3275_All, Unigene4286_All, Unigene466_All, Unigene643_All, Unigene675_All
69	Biosynthesis of unsaturated fatty acids	CL10318.Contig1_All, CL10424.Contig1_All, CL10424.Contig2_All, CL10687.Contig1_All, CL10814.Contig1_All, CL11437.Contig1_All, CL11756.Contig1_All, CL11952.Contig1_All, CL12136.Contig1_All, CL12285.Contig1_All, CL12501.Contig1_All, CL12674.Contig1_All, CL12784.Contig1_All, CL12784.Contig2_All, CL12785.Contig1_All, CL12822.Contig1_All, CL12981.Contig1_All, CL13176.Contig1_All, CL13725.Contig1_All, CL14144.Contig1_All, CL14252.Contig1_All, CL14364.Contig1_All, CL15213.Contig1_All, CL15336.Contig1_All, CL15629.Contig1_All, CL16640.Contig1_All, CL1728.Contig1_All, CL1728.Contig2_All, CL17699.Contig1_All, CL18506.Contig1_All, CL1905.Contig1_All, CL1905.Contig2_All, CL21134.Contig1_All, CL2115.Contig1_All, CL21352.Contig1_All, CL21644.Contig1_All, CL22862.Contig1_All, CL23185.Contig1_All, CL2381.Contig1_All, CL24473.Contig1_All, CL24508.Contig1_All, CL25435.Contig1_All, CL25757.Contig1_All, CL2755.Contig1_All, CL2755.Contig2_All, CL2755.Contig3_All, CL3039.Contig1_All, CL3070.Contig1_All, CL3070.Contig3_All, CL5081.Contig1_All, CL5296.Contig1_All, CL53.Contig10_All, CL53.Contig11_All, CL53.Contig1_All, CL53.Contig3_All, CL53.Contig6_All, CL53.Contig7_All, CL53.Contig8_All, CL53.Contig9_All, CL5369.Contig1_All, CL5476.Contig1_All, CL5691.Contig1_All, CL5691.Contig2_All, CL5819.Contig1_All, CL7347.Contig1_All, CL7529.Contig1_All, CL7978.Contig1_All, CL822.Contig1_All, CL822.Contig2_All, CL822.Contig3_All, CL8496.Contig1_All, CL8578.Contig1_All, CL8819.Contig1_All, CL8935.Contig1_All, CL9174.Contig1_All, CL9612.Contig1_All, CL9976.Contig1_All, Unigene1166_All, Unigene2308_All, Unigene2602_All, Unigene2771_All, Unigene327_All, Unigene363_All, Unigene4745_All, Unigene5065_All, Unigene5085_All, Unigene785_All, Unigene802_All, Unigene973_All

70	Phenylalanine, tyrosine and tryptophan biosynthesis	CL10136.Contig1_All, CL10183.Contig1_All, CL102.Contig10_All, CL102.Contig1_All, CL102.Contig2_All, CL102.Contig3_All, CL102.Contig4_All, CL102.Contig5_All, CL102.Contig6_All, CL102.Contig7_All, CL102.Contig8_All, CL102.Contig9_All, CL10238.Contig1_All, CL10444.Contig1_All, CL10568.Contig1_All, CL10992.Contig1_All, CL10994.Contig1_All, CL11401.Contig1_All, CL1166.Contig1_All, CL1166.Contig2_All, CL1166.Contig3_All, CL11762.Contig1_All, CL11940.Contig1_All, CL11994.Contig1_All, CL12133.Contig1_All, CL12364.Contig1_All, CL12720.Contig1_All, CL12818.Contig1_All, CL12928.Contig1_All, CL14842.Contig1_All, CL16561.Contig1_All, CL16840.Contig1_All, CL17233.Contig1_All, CL17459.Contig1_All, CL17459.Contig2_All, CL17921.Contig1_All, CL18979.Contig1_All, CL19059.Contig1_All, CL1968.Contig1_All, CL22411.Contig1_All, CL22879.Contig1_All, CL23294.Contig1_All, CL234.Contig5_All, CL234.Contig6_All, CL23703.Contig1_All, CL2398.Contig1_All, CL25992.Contig1_All, CL26700.Contig1_All, CL26958.Contig1_All, CL27798.Contig1_All, CL2876.Contig1_All, CL3351.Contig1_All, CL3629.Contig1_All, CL3805.Contig1_All, CL4055.Contig1_All, CL4140.Contig1_All, CL4316.Contig1_All, CL4331.Contig1_All, CL4392.Contig1_All, CL4529.Contig1_All, CL4850.Contig1_All, CL4850.Contig2_All, CL4850.Contig3_All, CL504.Contig1_All, CL504.Contig2_All, CL504.Contig3_All, CL504.Contig4_All, CL504.Contig5_All, CL5237.Contig1_All, CL5408.Contig1_All, CL5787.Contig1_All, CL6511.Contig1_All, CL6587.Contig1_All, CL7271.Contig1_All, CL7335.Contig1_All, CL8297.Contig1_All, CL8386.Contig1_All, CL8559.Contig1_All, CL9652.Contig1_All, CL9882.Contig1_All, CL9882.Contig2_All, Unigene1045_All, Unigene1062_All, Unigene2126_All, Unigene3071_All, Unigene4342_All
		CL10286.Contig2_All, CL10409.Contig1_All, CL10579.Contig1_All, CL10968.Contig1_All, CL11028.Contig1_All, CL11177.Contig1_All, CL11364.Contig1_All, CL11706.Contig1_All, CL11731.Contig1_All, CL11979.Contig1_All, CL13010.Contig1_All, CL132.Contig10_All, CL132.Contig11_All, CL132.Contig1_All, CL132.Contig2_All, CL132.Contig3_All, CL132.Contig4_All, CL132.Contig5_All, CL132.Contig6_All, CL132.Contig7_All, CL132.Contig8_All, CL132.Contig9_All, CL13774.Contig1_All, CL13914.Contig1_All, CL14274.Contig1_All, CL14287.Contig1_All, CL14387.Contig1_All, CL15096.Contig1_All, CL15130.Contig1_All, CL15221.Contig1_All, CL15391.Contig1_All, CL15519.Contig1_All, CL15596.Contig1_All,

71	SNARE interactions in vesicular transport	CL16453.Contig1_All, CL16568.Contig1_All, CL17421.Contig1_All, CL18030.Contig1_All, CL18128.Contig1_All, CL18976.Contig1_All, CL19065.Contig1_All, CL19065.Contig2_All, CL19328.Contig1_All, CL19810.Contig1_All, CL20203.Contig1_All, CL20637.Contig1_All, CL21155.Contig1_All, CL22072.Contig1_All, CL23062.Contig1_All, CL23308.Contig1_All, CL23642.Contig1_All, CL23765.Contig1_All, CL2489.Contig1_All, CL2489.Contig2_All, CL2489.Contig3_All, CL27388.Contig1_All, CL2882.Contig1_All, CL2882.Contig2_All, CL3135.Contig1_All, CL3631.Contig1_All, CL4622.Contig1_All, CL4794.Contig1_All, CL4794.Contig2_All, CL4798.Contig1_All, CL5310.Contig1_All, CL537.Contig1_All, CL537.Contig3_All, CL537.Contig4_All, CL537.Contig6_All, CL5404.Contig1_All, CL5686.Contig1_All, CL5889.Contig1_All, CL5889.Contig2_All, CL6932.Contig1_All, CL7530.Contig1_All, CL7855.Contig1_All, CL8079.Contig1_All, CL8367.Contig1_All, CL8483.Contig1_All, CL8987.Contig1_All, CL9015.Contig1_All, Unigene1787_All, Unigene2127_All, Unigene2684_All, Unigene4253_All, Unigene751_All
72	Valine, leucine and isoleucine degradation	CL10162.Contig1_All, CL10551.Contig1_All, CL10687.Contig1_All, CL10727.Contig1_All, CL10740.Contig1_All, CL11271.Contig1_All, CL11342.Contig1_All, CL1162.Contig1_All, CL11754.Contig1_All, CL12067.Contig1_All, CL12238.Contig1_All, CL12277.Contig1_All, CL1228.Contig1_All, CL1234.Contig1_All, CL1234.Contig2_All, CL12498.Contig1_All, CL12674.Contig1_All, CL13023.Contig1_All, CL13092.Contig1_All, CL13571.Contig1_All, CL1412.Contig1_All, CL1412.Contig2_All, CL14294.Contig1_All, CL15340.Contig1_All, CL16239.Contig1_All, CL16543.Contig1_All, CL16871.Contig1_All, CL1728.Contig1_All, CL1728.Contig2_All, CL18933.Contig1_All, CL201.Contig5_All, CL20673.Contig1_All, CL21178.Contig1_All, CL2130.Contig1_All, CL23231.Contig1_All, CL23285.Contig1_All, CL23375.Contig1_All, CL23687.Contig1_All, CL24033.Contig1_All, CL24508.Contig1_All, CL25324.Contig1_All, CL27255.Contig1_All, CL2929.Contig1_All, CL3049.Contig1_All, CL3170.Contig1_All, CL32.Contig12_All, CL3448.Contig1_All, CL3448.Contig2_All, CL3457.Contig1_All, CL3545.Contig1_All, CL3665.Contig1_All, CL4353.Contig1_All, CL4403.Contig1_All, CL444.Contig3_All, CL4678.Contig1_All, CL5024.Contig1_All, CL5400.Contig1_All, CL5549.Contig1_All, CL5549.Contig2_All, CL5560.Contig1_All, CL5678.Contig1_All, CL5837.Contig1_All,

		CL6064.Contig1_All, CL6371.Contig1_All, CL6596.Contig1_All, CL6607.Contig1_All, CL729.Contig1_All, CL729.Contig2_All, CL729.Contig3_All, CL729.Contig4_All, CL8172.Contig1_All, CL9106.Contig1_All, CL9174.Contig1_All, CL9429.Contig1_All, CL9947.Contig1_All, Unigene1434_All, Unigene2872_All, Unigene3368_All, Unigene363_All, Unigene4417_All, Unigene5473_All, Unigene5509_All, Unigene920_All
73	Sphingolipid metabolism	CL108.Contig2_All, CL10902.Contig1_All, CL11066.Contig1_All, CL1288.Contig1_All, CL1288.Contig2_All, CL1288.Contig3_All, CL1288.Contig4_All, CL1304.Contig1_All, CL1304.Contig2_All, CL1304.Contig3_All, CL13284.Contig1_All, CL1428.Contig1_All, CL1428.Contig2_All, CL1428.Contig3_All, CL1428.Contig4_All, CL1428.Contig5_All, CL1428.Contig6_All, CL1428.Contig7_All, CL16094.Contig1_All, CL17196.Contig1_All, CL1738.Contig1_All, CL17482.Contig1_All, CL1750.Contig1_All, CL18263.Contig1_All, CL19717.Contig1_All, CL20816.Contig1_All, CL21109.Contig1_All, CL22290.Contig1_All, CL22556.Contig1_All, CL23651.Contig1_All, CL23669.Contig1_All, CL24467.Contig1_All, CL25863.Contig1_All, CL2668.Contig1_All, CL27552.Contig1_All, CL2783.Contig1_All, CL3070.Contig1_All, CL3070.Contig2_All, CL3070.Contig3_All, CL3576.Contig1_All, CL3576.Contig2_All, CL3654.Contig1_All, CL399.Contig1_All, CL399.Contig2_All, CL399.Contig3_All, CL399.Contig4_All, CL399.Contig5_All, CL399.Contig6_All, CL4.Contig34_All, CL4170.Contig1_All, CL4628.Contig1_All, CL4628.Contig2_All, CL4811.Contig1_All, CL5116.Contig1_All, CL5150.Contig1_All, CL5425.Contig1_All, CL560.Contig1_All, CL5638.Contig1_All, CL5856.Contig1_All, CL5925.Contig1_All, CL613.Contig1_All, CL613.Contig2_All, CL613.Contig3_All, CL613.Contig4_All, CL621.Contig1_All, CL621.Contig2_All, CL6369.Contig1_All, CL657.Contig1_All, CL657.Contig3_All, CL657.Contig4_All, CL657.Contig5_All, CL657.Contig6_All, CL6830.Contig1_All, CL7566.Contig1_All, Unigene2287_All, Unigene2545_All, Unigene3777_All, Unigene4790_All, Unigene4932_All, Unigene4933_All, Unigene664_All, Unigene705_All
		CL10355.Contig1_All, CL10397.Contig1_All, CL11080.Contig1_All, CL11135.Contig1_All, CL11140.Contig1_All, CL11971.Contig1_All, CL12055.Contig1_All, CL12270.Contig1_All, CL12281.Contig1_All, CL12396.Contig1_All, CL1246.Contig1_All, CL12653.Contig1_All, CL12667.Contig1_All, CL13139.Contig1_All, CL13549.Contig1_All, CL1369.Contig1_All, CL1369.Contig2_All, CL1369.Contig3_All, CL15652.Contig1_All, CL15733.Contig1_All, CL15857.Contig1_All, CL16358.Contig1_All,

74	Steroid biosynthesis	CL16414.Contig1_All, CL16464.Contig1_All, CL16502.Contig1_All, CL16785.Contig1_All, CL1679.Contig1_All, CL16998.Contig1_All, CL18215.Contig1_All, CL18450.Contig1_All, CL19577.Contig1_All, CL19710.Contig1_All, CL20094.Contig1_All, CL20454.Contig1_All, CL22071.Contig1_All, CL22607.Contig1_All, CL22885.Contig1_All, CL22895.Contig1_All, CL23330.Contig1_All, CL24032.Contig1_All, CL25142.Contig1_All, CL2531.Contig1_All, CL2531.Contig4_All, CL2531.Contig5_All, CL2531.Contig6_All, CL2531.Contig7_All, CL26522.Contig1_All, CL26896.Contig1_All, CL271.Contig1_All, CL271.Contig7_All, CL271.Contig8_All, CL273.Contig6_All, CL2958.Contig1_All, CL2958.Contig2_All, CL3230.Contig1_All, CL3455.Contig1_All, CL349.Contig1_All, CL349.Contig2_All, CL349.Contig3_All, CL349.Contig5_All, CL3590.Contig1_All, CL3846.Contig1_All, CL4811.Contig1_All, CL4837.Contig1_All, CL5385.Contig1_All, CL5385.Contig2_All, CL5984.Contig1_All, CL6199.Contig1_All, CL6199.Contig2_All, CL6709.Contig1_All, CL6817.Contig1_All, CL7902.Contig1_All, CL8943.Contig1_All, CL8945.Contig1_All, CL9226.Contig1_All, Unigene1655_All, Unigene2574_All, Unigene4096_All, Unigene4538_All
75	Tyrosine metabolism	CL10068.Contig1_All, CL1009.Contig1_All, CL10136.Contig1_All, CL10238.Contig1_All, CL10532.Contig1_All, CL10568.Contig1_All, CL10994.Contig1_All, CL11004.Contig1_All, CL12133.Contig1_All, CL12720.Contig1_All, CL1275.Contig1_All, CL13321.Contig1_All, CL13548.Contig1_All, CL13794.Contig1_All, CL13936.Contig1_All, CL14027.Contig1_All, CL14937.Contig1_All, CL14990.Contig1_All, CL16786.Contig1_All, CL16878.Contig1_All, CL17459.Contig1_All, CL17459.Contig2_All, CL18250.Contig1_All, CL2.Contig25_All, CL2005.Contig1_All, CL2043.Contig1_All, CL21082.Contig1_All, CL21286.Contig1_All, CL21565.Contig1_All, CL22411.Contig1_All, CL23294.Contig1_All, CL24211.Contig1_All, CL25407.Contig1_All, CL25992.Contig1_All, CL26958.Contig1_All, CL27162.Contig1_All, CL2876.Contig1_All, CL3113.Contig1_All, CL4055.Contig1_All, CL4137.Contig1_All, CL4549.Contig1_All, CL504.Contig1_All, CL504.Contig2_All, CL504.Contig3_All, CL504.Contig4_All, CL504.Contig5_All, CL51.Contig17_All, CL5187.Contig1_All, CL5237.Contig1_All, CL5787.Contig1_All, CL609.Contig1_All, CL609.Contig2_All, CL609.Contig3_All, CL609.Contig4_All, CL6321.Contig1_All, CL6337.Contig1_All, CL6371.Contig1_All, CL6401.Contig1_All, CL6637.Contig1_All, CL6998.Contig1_All, CL8277.Contig1_All, CL8297.Contig1_All,

			CL8386.Contig1_All, CL8697.Contig1_All, CL8972.Contig1_All, CL9067.Contig1_All, CL908.Contig1_All, CL908.Contig2_All, CL908.Contig3_All, CL908.Contig4_All, CL9764.Contig1_All, Unigene1045_All, Unigene2503_All, Unigene3071_All, Unigene3611_All, Unigene3918_All, Unigene3983_All, Unigene4170_All, Unigene4497_All
76	Ubiquinone and other terpenoid-quinone biosynthesis		CL10556.Contig1_All, CL11130.Contig1_All, CL11277.Contig1_All, CL12720.Contig1_All, CL12820.Contig1_All, CL12917.Contig1_All, CL13001.Contig1_All, CL13152.Contig1_All, CL13172.Contig1_All, CL1407.Contig1_All, CL1407.Contig2_All, CL1407.Contig3_All, CL1469.Contig3_All, CL16124.Contig1_All, CL16186.Contig1_All, CL17459.Contig1_All, CL17459.Contig2_All, CL18330.Contig1_All, CL1847.Contig1_All, CL1916.Contig1_All, CL20484.Contig1_All, CL21671.Contig1_All, CL2226.Contig1_All, CL23294.Contig1_All, CL23945.Contig1_All, CL24036.Contig1_All, CL24379.Contig1_All, CL25126.Contig1_All, CL25992.Contig1_All, CL26958.Contig1_All, CL27567.Contig1_All, CL2876.Contig1_All, CL3062.Contig2_All, CL311.Contig1_All, CL3226.Contig1_All, CL3314.Contig1_All, CL3846.Contig1_All, CL4024.Contig1_All, CL4055.Contig1_All, CL4129.Contig1_All, CL4543.Contig1_All, CL4743.Contig1_All, CL4751.Contig1_All, CL4837.Contig1_All, CL504.Contig1_All, CL504.Contig2_All, CL504.Contig3_All, CL504.Contig4_All, CL504.Contig5_All, CL5325.Contig1_All, CL5787.Contig1_All, CL5906.Contig1_All, CL6146.Contig1_All, CL7192.Contig1_All, CL7192.Contig2_All, CL7326.Contig1_All, CL7354.Contig1_All, CL7545.Contig1_All, CL7545.Contig2_All, CL7829.Contig1_All, CL793.Contig1_All, CL8197.Contig1_All, CL8277.Contig1_All, CL8297.Contig1_All, CL8465.Contig1_All, CL9407.Contig1_All, CL9677.Contig1_All, CL9688.Contig1_All, Unigene1045_All, Unigene1064_All, Unigene1109_All, Unigene1586_All, Unigene2338_All, Unigene2543_All, Unigene3071_All, Unigene3397_All, Unigene440_All, Unigene938_All
			CL10053.Contig1_All, CL10266.Contig1_All, CL10551.Contig1_All, CL10740.Contig1_All, CL10841.Contig1_All, CL11342.Contig1_All, CL12067.Contig1_All, CL12466.Contig1_All, CL12498.Contig1_All, CL12523.Contig1_All, CL12698.Contig1_All, CL12722.Contig1_All, CL13023.Contig1_All, CL13092.Contig1_All, CL13571.Contig1_All, CL1412.Contig1_All, CL1412.Contig2_All, CL14294.Contig1_All, CL14677.Contig1_All, CL147.Contig1_All, CL150.Contig7_All, CL15340.Contig1_All, CL16239.Contig1_All, CL16274.Contig1_All, CL16543.Contig1_All, CL16871.Contig1_All, CL17667.Contig1_All,

77	Propanoate metabolism	CL1874.Contig1_All, CL1874.Contig2_All, CL1874.Contig3_All, CL1874.Contig4_All, CL19616.Contig1_All, CL2041.Contig1_All, CL2041.Contig2_All, CL2225.Contig1_All, CL2225.Contig2_All, CL23375.Contig1_All, CL23687.Contig1_All, CL2490.Contig1_All, CL25324.Contig1_All, CL2929.Contig1_All, CL3049.Contig1_All, CL3307.Contig1_All, CL3342.Contig1_All, CL3448.Contig1_All, CL3448.Contig2_All, CL3457.Contig1_All, CL3545.Contig1_All, CL4403.Contig1_All, CL5024.Contig1_All, CL5478.Contig1_All, CL5549.Contig1_All, CL5549.Contig2_All, CL5968.Contig1_All, CL6371.Contig1_All, CL6565.Contig1_All, CL6596.Contig1_All, CL6607.Contig1_All, CL700.Contig1_All, CL700.Contig2_All, CL700.Contig3_All, CL700.Contig4_All, CL700.Contig5_All, CL8366.Contig1_All, CL8624.Contig1_All, CL8718.Contig1_All, CL8873.Contig1_All, CL9106.Contig1_All, CL9429.Contig1_All, Unigene1773_All, Unigene205_All, Unigene2872_All, Unigene3997_All, Unigene4417_All, Unigene5089_All, Unigene666_All, Unigene920_All
78	Terpenoid backbone biosynthesis	CL10217.Contig1_All, CL10426.Contig1_All, CL10433.Contig1_All, CL10551.Contig1_All, CL10632.Contig1_All, CL10650.Contig1_All, CL11021.Contig1_All, CL11090.Contig1_All, CL11178.Contig1_All, CL11348.Contig1_All, CL11535.Contig1_All, CL12037.Contig1_All, CL12247.Contig1_All, CL12322.Contig1_All, CL12373.Contig1_All, CL12410.Contig1_All, CL12496.Contig1_All, CL13024.Contig1_All, CL13071.Contig1_All, CL13486.Contig1_All, CL13999.Contig1_All, CL13999.Contig2_All, CL16109.Contig1_All, CL16347.Contig1_All, CL18054.Contig1_All, CL19263.Contig1_All, CL20240.Contig1_All, CL20332.Contig1_All, CL20484.Contig1_All, CL21201.Contig1_All, CL21945.Contig1_All, CL22573.Contig1_All, CL22641.Contig1_All, CL23042.Contig1_All, CL24287.Contig1_All, CL2468.Contig1_All, CL25097.Contig1_All, CL2546.Contig1_All, CL2674.Contig1_All, CL27025.Contig1_All, CL27097.Contig1_All, CL275.Contig1_All, CL275.Contig2_All, CL278.Contig1_All, CL278.Contig2_All, CL278.Contig6_All, CL278.Contig7_All, CL27887.Contig1_All, CL3143.Contig1_All, CL3143.Contig2_All, CL3448.Contig1_All, CL3448.Contig2_All, CL5066.Contig1_All, CL5930.Contig1_All, CL6475.Contig1_All, CL6534.Contig1_All, CL7213.Contig1_All, CL7213.Contig2_All, CL7364.Contig1_All, CL7593.Contig1_All, CL7879.Contig1_All, CL8172.Contig1_All, CL8535.Contig1_All, CL8878.Contig1_All,

		CL9150.Contig1_All, CL9302.Contig1_All, Unigene1348_All, Unigene2109_All, Unigene2360_All, Unigene2511_All, Unigene3744_All, Unigene4065_All, Unigene4539_All, Unigene619_All, Unigene733_All, Unigene979_All
79	Valine, leucine and isoleucine biosynthesis	CL1007.Contig1_All, CL10655.Contig1_All, CL11.Contig17_All, CL11200.Contig1_All, CL1162.Contig1_All, CL11632.Contig1_All, CL11779.Contig1_All, CL11840.Contig1_All, CL11877.Contig1_All, CL11878.Contig1_All, CL11966.Contig1_All, CL1265.Contig1_All, CL12753.Contig1_All, CL12865.Contig1_All, CL13338.Contig1_All, CL1339.Contig1_All, CL1339.Contig2_All, CL1339.Contig3_All, CL1339.Contig4_All, CL13926.Contig1_All, CL14011.Contig1_All, CL14439.Contig1_All, CL1502.Contig1_All, CL15125.Contig1_All, CL15367.Contig1_All, CL15407.Contig1_All, CL16566.Contig1_All, CL17689.Contig1_All, CL201.Contig5_All, CL20673.Contig1_All, CL20774.Contig1_All, CL21307.Contig1_All, CL2185.Contig1_All, CL2185.Contig2_All, CL2212.Contig1_All, CL2212.Contig2_All, CL22599.Contig1_All, CL2312.Contig1_All, CL23231.Contig1_All, CL24033.Contig1_All, CL24665.Contig1_All, CL26742.Contig1_All, CL27255.Contig1_All, CL2752.Contig1_All, CL2965.Contig1_All, CL3170.Contig1_All, CL3445.Contig1_All, CL3445.Contig2_All, CL3445.Contig3_All, CL3914.Contig1_All, CL444.Contig3_All, CL506.Contig1_All, CL520.Contig1_All, CL520.Contig2_All, CL520.Contig3_All, CL520.Contig4_All, CL5410.Contig1_All, CL5445.Contig1_All, CL5678.Contig1_All, CL5793.Contig1_All, CL71.Contig2_All, CL71.Contig3_All, CL7202.Contig1_All, CL7261.Contig1_All, CL7261.Contig2_All, CL8261.Contig1_All, CL887.Contig1_All, CL887.Contig2_All, CL887.Contig3_All, CL8959.Contig1_All, CL9950.Contig1_All, Unigene4507_All
80	beta-Alanine metabolism	CL10740.Contig1_All, CL11.Contig18_All, CL11.Contig19_All, CL11.Contig2_All, CL11342.Contig1_All, CL12067.Contig1_All, CL12156.Contig1_All, CL12346.Contig1_All, CL12498.Contig1_All, CL13023.Contig1_All, CL13092.Contig1_All, CL13936.Contig1_All, CL1412.Contig1_All, CL1412.Contig2_All, CL14294.Contig1_All, CL14677.Contig1_All, CL15175.Contig1_All, CL15340.Contig1_All, CL15387.Contig1_All, CL16239.Contig1_All, CL16543.Contig1_All, CL16871.Contig1_All, CL18250.Contig1_All, CL1882.Contig1_All, CL1882.Contig2_All, CL1882.Contig3_All, CL1882.Contig4_All, CL1882.Contig5_All, CL1882.Contig6_All, CL19968.Contig1_All, CL20054.Contig1_All, CL2043.Contig1_All, CL21082.Contig1_All, CL2225.Contig1_All, CL2225.Contig2_All,

		CL23375.Contig1_All, CL23687.Contig1_All, CL25324.Contig1_All, CL2621.Contig1_All, CL2621.Contig2_All, CL2929.Contig1_All, CL3049.Contig1_All, CL3457.Contig1_All, CL3545.Contig1_All, CL4301.Contig1_All, CL4403.Contig1_All, CL5024.Contig1_All, CL5138.Contig1_All, CL5173.Contig1_All, CL5549.Contig1_All, CL5549.Contig2_All, CL6371.Contig1_All, CL6401.Contig1_All, CL6579.Contig1_All, CL6596.Contig1_All, CL7040.Contig1_All, CL7796.Contig1_All, CL8638.Contig1_All, CL8697.Contig1_All, CL908.Contig1_All, CL908.Contig2_All, CL908.Contig3_All, CL908.Contig4_All, CL9106.Contig1_All, CL9385.Contig1_All, CL9429.Contig1_All, Unigene2872_All, Unigene3426_All, Unigene3611_All, Unigene4417_All, Unigene5089_All, Unigene920_All
81	Glyoxylate and dicarboxylate metabolism	CL10551.Contig1_All, CL12222.Contig1_All, CL12301.Contig1_All, CL12429.Contig1_All, CL1272.Contig1_All, CL1272.Contig2_All, CL1272.Contig3_All, CL12852.Contig1_All, CL12909.Contig1_All, CL12957.Contig1_All, CL13019.Contig1_All, CL13034.Contig1_All, CL14659.Contig1_All, CL14933.Contig1_All, CL15475.Contig1_All, CL1558.Contig1_All, CL1558.Contig2_All, CL15778.Contig1_All, CL15935.Contig1_All, CL16214.Contig1_All, CL16802.Contig1_All, CL16982.Contig1_All, CL17109.Contig1_All, CL18226.Contig1_All, CL18244.Contig1_All, CL20185.Contig1_All, CL25053.Contig1_All, CL2762.Contig1_All, CL2932.Contig1_All, CL3448.Contig1_All, CL3448.Contig2_All, CL3505.Contig1_All, CL3773.Contig1_All, CL395.Contig6_All, CL4448.Contig1_All, CL4666.Contig1_All, CL4980.Contig1_All, CL561.Contig1_All, CL561.Contig2_All, CL561.Contig3_All, CL561.Contig4_All, CL561.Contig5_All, CL561.Contig6_All, CL561.Contig7_All, CL561.Contig8_All, CL5769.Contig1_All, CL5936.Contig1_All, CL5936.Contig2_All, CL595.Contig2_All, CL6607.Contig1_All, CL69.Contig1_All, CL69.Contig2_All, CL69.Contig3_All, CL69.Contig4_All, CL69.Contig5_All, CL69.Contig6_All, CL69.Contig7_All, CL69.Contig8_All, CL8138.Contig1_All, CL8606.Contig1_All, Unigene1120_All, Unigene1245_All, Unigene400_All, Unigene4118_All, Unigene4156_All, Unigene441_All, Unigene4527_All, Unigene5496_All, Unigene5498_All
		CL10215.Contig1_All, CL10542.Contig1_All, CL10551.Contig1_All, CL10740.Contig1_All, CL1168.Contig1_All, CL1191.Contig1_All, CL1191.Contig2_All, CL1191.Contig3_All, CL12067.Contig1_All, CL12498.Contig1_All, CL13023.Contig1_All, CL13271.Contig1_All, CL13462.Contig1_All, CL13661.Contig1_All, CL1412.Contig1_All, CL1412.Contig2_All,

82	Lysine degradation	CL14294.Contig1_All, CL1562.Contig1_All, CL1562.Contig2_All, CL1562.Contig3_All, CL1562.Contig4_All, CL1587.Contig1_All, CL16239.Contig1_All, CL16346.Contig1_All, CL16543.Contig1_All, CL16871.Contig1_All, CL17359.Contig1_All, CL17451.Contig1_All, CL18296.Contig1_All, CL19197.Contig1_All, CL19305.Contig1_All, CL20915.Contig1_All, CL22305.Contig1_All, CL23375.Contig1_All, CL23687.Contig1_All, CL24101.Contig1_All, CL2912.Contig1_All, CL2929.Contig1_All, CL2950.Contig1_All, CL3229.Contig1_All, CL332.Contig1_All, CL3448.Contig1_All, CL3448.Contig2_All, CL3457.Contig1_All, CL3545.Contig1_All, CL3962.Contig1_All, CL3962.Contig2_All, CL5309.Contig1_All, CL5549.Contig1_All, CL5549.Contig2_All, CL565.Contig1_All, CL6371.Contig1_All, CL6778.Contig1_All, CL7214.Contig1_All, CL7655.Contig1_All, CL7655.Contig2_All, CL8307.Contig1_All, CL905.Contig1_All, CL9106.Contig1_All, CL9229.Contig1_All, CL9334.Contig1_All, CL9429.Contig1_All, CL9496.Contig1_All, CL9753.Contig1_All, Unigene2872_All, Unigene3164_All, Unigene4417_All, Unigene5187_All, Unigene920_All
83	Sulfur metabolism	CL10490.Contig1_All, CL10623.Contig1_All, CL10685.Contig1_All, CL10886.Contig1_All, CL10964.Contig1_All, CL11468.Contig1_All, CL11525.Contig1_All, CL11606.Contig1_All, CL12945.Contig1_All, CL12945.Contig2_All, CL13025.Contig1_All, CL13054.Contig1_All, CL13220.Contig1_All, CL13232.Contig1_All, CL13506.Contig1_All, CL14098.Contig1_All, CL14922.Contig1_All, CL15166.Contig1_All, CL15323.Contig1_All, CL15961.Contig1_All, CL16051.Contig1_All, CL17370.Contig1_All, CL17574.Contig1_All, CL17581.Contig1_All, CL18109.Contig1_All, CL18289.Contig1_All, CL18529.Contig1_All, CL18732.Contig1_All, CL19111.Contig1_All, CL1995.Contig1_All, CL1995.Contig2_All, CL1995.Contig3_All, CL23614.Contig1_All, CL2367.Contig1_All, CL2367.Contig2_All, CL2367.Contig3_All, CL23781.Contig1_All, CL24023.Contig1_All, CL27895.Contig1_All, CL3401.Contig1_All, CL4099.Contig1_All, CL4347.Contig1_All, CL4347.Contig2_All, CL4347.Contig3_All, CL4347.Contig4_All, CL4509.Contig1_All, CL4873.Contig1_All, CL5500.Contig1_All, CL5500.Contig2_All, CL5504.Contig1_All, CL5680.Contig1_All, CL587.Contig1_All, CL587.Contig3_All, CL587.Contig4_All, CL587.Contig5_All, CL587.Contig6_All, CL6395.Contig1_All, CL6871.Contig1_All, CL7442.Contig1_All, CL7518.Contig1_All, CL7740.Contig1_All, CL856.Contig1_All, CL8894.Contig1_All,

		CL9970.Contig1_All, CL9981.Contig1_All, Unigene3980_All, Unigene4469_All, Unigene4548_All, Unigene4864_All
84	Glycosylphosphatidylinositol (GPI)-anchor biosynthesis	CL10251.Contig1_All, CL10408.Contig1_All, CL10534.Contig1_All, CL1059.Contig1_All, CL1059.Contig2_All, CL10865.Contig1_All, CL11964.Contig1_All, CL12418.Contig1_All, CL12748.Contig1_All, CL12816.Contig1_All, CL13531.Contig1_All, CL13889.Contig1_All, CL15446.Contig1_All, CL15504.Contig1_All, CL15626.Contig1_All, CL15672.Contig1_All, CL16723.Contig1_All, CL17630.Contig1_All, CL18111.Contig1_All, CL18474.Contig1_All, CL19257.Contig1_All, CL19635.Contig1_All, CL1967.Contig1_All, CL1967.Contig2_All, CL1967.Contig3_All, CL19784.Contig1_All, CL2026.Contig1_All, CL22335.Contig1_All, CL22547.Contig1_All, CL22666.Contig1_All, CL23167.Contig1_All, CL23240.Contig1_All, CL23314.Contig1_All, CL23328.Contig1_All, CL23898.Contig1_All, CL24569.Contig1_All, CL25040.Contig1_All, CL25352.Contig1_All, CL2591.Contig1_All, CL26687.Contig1_All, CL26902.Contig1_All, CL27321.Contig1_All, CL27470.Contig1_All, CL27904.Contig1_All, CL27982.Contig1_All, CL6212.Contig1_All, CL7303.Contig1_All, CL7303.Contig2_All, CL8236.Contig1_All, CL8587.Contig1_All, CL8781.Contig1_All, CL8781.Contig2_All, CL8803.Contig1_All, CL8869.Contig1_All, CL9104.Contig1_All, CL9104.Contig2_All, CL9129.Contig1_All, CL9682.Contig1_All, CL9745.Contig1_All, CL9790.Contig1_All, Unigene127_All, Unigene1734_All, Unigene2341_All, Unigene2797_All, Unigene2994_All, Unigene3293_All, Unigene4281_All, Unigene832_All
85	Tropane, piperidine and pyridine alkaloid biosynthesis	CL10238.Contig1_All, CL10318.Contig1_All, CL10568.Contig1_All, CL10994.Contig1_All, CL11635.Contig1_All, CL12133.Contig1_All, CL12720.Contig1_All, CL12875.Contig1_All, CL13936.Contig1_All, CL156.Contig1_All, CL156.Contig2_All, CL156.Contig3_All, CL156.Contig4_All, CL156.Contig5_All, CL156.Contig6_All, CL156.Contig7_All, CL156.Contig8_All, CL156.Contig9_All, CL17459.Contig1_All, CL17459.Contig2_All, CL18250.Contig1_All, CL18381.Contig1_All, CL20025.Contig1_All, CL2043.Contig1_All, CL20518.Contig1_All, CL21082.Contig1_All, CL21700.Contig1_All, CL22411.Contig1_All, CL22703.Contig1_All, CL23294.Contig1_All, CL24115.Contig1_All, CL25210.Contig1_All, CL25992.Contig1_All, CL26958.Contig1_All, CL2876.Contig1_All, CL4055.Contig1_All, CL504.Contig1_All, CL504.Contig2_All, CL504.Contig3_All, CL504.Contig4_All, CL504.Contig5_All, CL5237.Contig1_All, CL5787.Contig1_All,

		CL5884.Contig1_All, CL6401.Contig1_All, CL8297.Contig1_All, CL8386.Contig1_All, CL8697.Contig1_All, CL908.Contig1_All, CL908.Contig2_All, CL908.Contig3_All, CL908.Contig4_All, CL986.Contig1_All, CL986.Contig2_All, CL986.Contig3_All, CL986.Contig4_All, CL986.Contig5_All, Unigene1045_All, Unigene3071_All, Unigene3611_All
86	Fatty acid biosynthesis	CL10266.Contig1_All, CL10318.Contig1_All, CL10504.Contig1_All, CL10630.Contig1_All, CL11952.Contig1_All, CL12260.Contig1_All, CL12466.Contig1_All, CL12501.Contig1_All, CL12523.Contig1_All, CL1294.Contig1_All, CL1294.Contig2_All, CL1294.Contig3_All, CL1294.Contig4_All, CL13176.Contig1_All, CL13291.Contig1_All, CL14252.Contig1_All, CL1427.Contig1_All, CL1427.Contig3_All, CL1427.Contig4_All, CL1427.Contig5_All, CL147.Contig1_All, CL150.Contig7_All, CL15213.Contig1_All, CL18453.Contig1_All, CL18506.Contig1_All, CL21352.Contig1_All, CL22417.Contig1_All, CL23767.Contig1_All, CL2381.Contig1_All, CL3061.Contig1_All, CL3070.Contig1_All, CL3070.Contig3_All, CL3342.Contig1_All, CL370.Contig2_All, CL370.Contig3_All, CL370.Contig8_All, CL3744.Contig1_All, CL4534.Contig1_All, CL4571.Contig1_All, CL4571.Contig2_All, CL5296.Contig1_All, CL5952.Contig1_All, CL6565.Contig1_All, CL7347.Contig1_All, CL7449.Contig1_All, CL7529.Contig1_All, CL830.Contig2_All, CL830.Contig3_All, CL830.Contig4_All, CL830.Contig5_All, CL8366.Contig1_All, CL8718.Contig1_All, CL8819.Contig1_All, Unigene205_All, Unigene2771_All, Unigene3997_All
87	Butanoate metabolism	CL10072.Contig2_All, CL10551.Contig1_All, CL10655.Contig1_All, CL11840.Contig1_All, CL11877.Contig1_All, CL12067.Contig1_All, CL12156.Contig1_All, CL12238.Contig1_All, CL12277.Contig1_All, CL12346.Contig1_All, CL12753.Contig1_All, CL12865.Contig1_All, CL13023.Contig1_All, CL1339.Contig1_All, CL1339.Contig2_All, CL1339.Contig3_All, CL1339.Contig4_All, CL14294.Contig1_All, CL1502.Contig1_All, CL17689.Contig1_All, CL18773.Contig1_All, CL22054.Contig1_All, CL22990.Contig1_All, CL23.Contig10_All, CL23.Contig14_All, CL23.Contig1_All, CL23.Contig3_All, CL26742.Contig1_All, CL32.Contig12_All, CL3448.Contig1_All, CL3448.Contig2_All, CL3545.Contig1_All, CL3991.Contig1_All, CL4716.Contig1_All, CL4716.Contig2_All, CL51.Contig17_All, CL5138.Contig1_All, CL5793.Contig1_All, CL6371.Contig1_All, CL6744.Contig1_All, CL7202.Contig1_All, CL759.Contig1_All, CL759.Contig2_All, CL759.Contig3_All, CL759.Contig4_All, CL759.Contig5_All, CL759.Contig6_All, CL8172.Contig1_All, CL8720.Contig1_All, CL8972.Contig1_All, CL9429.Contig1_All, Unigene1434_All,

		Unigene2872_All, Unigene2966_All, Unigene4507_All, Unigene920_All
88	Flavone and flavonol biosynthesis	CL12470.Contig1_All, CL13444.Contig1_All, CL15754.Contig1_All, CL16430.Contig1_All, CL18342.Contig1_All, CL1855.Contig1_All, CL1855.Contig2_All, CL1855.Contig3_All, CL1855.Contig4_All, CL19564.Contig1_All, CL22250.Contig1_All, CL22464.Contig1_All, CL22771.Contig1_All, CL23475.Contig1_All, CL24918.Contig1_All, CL25612.Contig1_All, CL25658.Contig1_All, CL25793.Contig1_All, CL25996.Contig1_All, CL27352.Contig1_All, CL40.Contig10_All, CL40.Contig11_All, CL40.Contig2_All, CL40.Contig4_All, CL40.Contig5_All, CL40.Contig6_All, CL40.Contig7_All, CL40.Contig8_All, CL4095.Contig1_All, CL4471.Contig1_All, CL4842.Contig1_All, CL50.Contig10_All, CL50.Contig12_All, CL50.Contig13_All, CL50.Contig14_All, CL50.Contig15_All, CL50.Contig16_All, CL50.Contig2_All, CL50.Contig3_All, CL50.Contig4_All, CL50.Contig5_All, CL50.Contig8_All, CL50.Contig9_All, CL5495.Contig1_All, CL6287.Contig1_All, CL9299.Contig1_All, CL9723.Contig1_All, Unigene1419_All, Unigene1482_All, Unigene1564_All, Unigene1634_All, Unigene2449_All, Unigene3670_All, Unigene3708_All, Unigene828_All
89	Other glycan degradation (no map in kegg database)	CL108.Contig2_All, CL12766.Contig1_All, CL1288.Contig1_All, CL1288.Contig2_All, CL1288.Contig3_All, CL1288.Contig4_All, CL1304.Contig1_All, CL1304.Contig2_All, CL1304.Contig3_All, CL14001.Contig1_All, CL14874.Contig1_All, CL17196.Contig1_All, CL17482.Contig1_All, CL1750.Contig1_All, CL17703.Contig1_All, CL19717.Contig1_All, CL20816.Contig1_All, CL22796.Contig1_All, CL23651.Contig1_All, CL25863.Contig1_All, CL26183.Contig1_All, CL26516.Contig1_All, CL3011.Contig1_All, CL3576.Contig1_All, CL3576.Contig2_All, CL3654.Contig1_All, CL3926.Contig1_All, CL399.Contig1_All, CL399.Contig2_All, CL399.Contig3_All, CL399.Contig4_All, CL399.Contig5_All, CL399.Contig6_All, CL4094.Contig1_All, CL4162.Contig1_All, CL4162.Contig2_All, CL560.Contig1_All, CL5615.Contig1_All, CL621.Contig1_All, CL621.Contig2_All, CL6875.Contig1_All, CL733.Contig1_All, CL8484.Contig1_All, CL9116.Contig1_All, CL9192.Contig1_All, CL939.Contig1_All, Unigene2287_All, Unigene3628_All, Unigene3672_All, Unigene4790_All, Unigene664_All, Unigene705_All
89		CL10668.Contig1_All, CL10856.Contig1_All, CL1162.Contig1_All, CL11759.Contig1_All, CL11878.Contig1_All, CL11900.Contig1_All, CL11966.Contig1_All, CL12345.Contig1_All, CL12550.Contig1_All, CL12720.Contig1_All, CL13054.Contig1_All, CL13253.Contig1_All, CL13530.Contig1_All, CL13926.Contig1_All, CL14439.Contig1_All, CL14695.Contig1_All,

90	Glucosinolate biosynthesis (no map in kegg database)	CL15166.Contig1_All, CL1545.Contig1_All, CL1545.Contig2_All, CL16298.Contig1_All, CL19111.Contig1_All, CL19283.Contig1_All, CL19754.Contig1_All, CL201.Contig5_All, CL20673.Contig1_All, CL21454.Contig1_All, CL21901.Contig1_All, CL23231.Contig1_All, CL23294.Contig1_All, CL24033.Contig1_All, CL24981.Contig1_All, CL25992.Contig1_All, CL26958.Contig1_All, CL27255.Contig1_All, CL3170.Contig1_All, CL444.Contig3_All, CL5504.Contig1_All, CL5678.Contig1_All, CL7442.Contig1_All, CL8297.Contig1_All, CL8555.Contig1_All, CL8959.Contig1_All, CL9821.Contig1_All, CL9950.Contig1_All, Unigene1796_All, Unigene4292_All, Unigene4627_All
91	Glycosaminoglycan degradation	CL1288.Contig1_All, CL1288.Contig2_All, CL1288.Contig3_All, CL1288.Contig4_All, CL1304.Contig1_All, CL1304.Contig2_All, CL1304.Contig3_All, CL14874.Contig1_All, CL16586.Contig1_All, CL17196.Contig1_All, CL17482.Contig1_All, CL1750.Contig1_All, CL19717.Contig1_All, CL20816.Contig1_All, CL21897.Contig1_All, CL23544.Contig1_All, CL23651.Contig1_All, CL2549.Contig1_All, CL25504.Contig1_All, CL25863.Contig1_All, CL2858.Contig1_All, CL2858.Contig2_All, CL3011.Contig1_All, CL3576.Contig1_All, CL3576.Contig2_All, CL3654.Contig1_All, CL399.Contig1_All, CL399.Contig2_All, CL399.Contig3_All, CL399.Contig4_All, CL399.Contig5_All, CL399.Contig6_All, CL403.Contig1_All, CL4094.Contig1_All, CL54.Contig17_All, CL54.Contig18_All, CL54.Contig19_All, CL54.Contig3_All, CL5615.Contig1_All, CL6101.Contig1_All, CL621.Contig1_All, CL621.Contig2_All, CL9192.Contig1_All, Unigene2287_All, Unigene4790_All, Unigene705_All
92	Lysine biosynthesis	CL10146.Contig1_All, CL10672.Contig1_All, CL11718.Contig1_All, CL12376.Contig1_All, CL12612.Contig1_All, CL12912.Contig1_All, CL14609.Contig1_All, CL1587.Contig1_All, CL16291.Contig1_All, CL16543.Contig1_All, CL17637.Contig1_All, CL17764.Contig1_All, CL1997.Contig1_All, CL1997.Contig2_All, CL1997.Contig3_All, CL20125.Contig1_All, CL20717.Contig1_All, CL21372.Contig1_All, CL2143.Contig1_All, CL22366.Contig1_All, CL22776.Contig1_All, CL23059.Contig1_All, CL23375.Contig1_All, CL24249.Contig1_All, CL2554.Contig1_All, CL25890.Contig1_All, CL27416.Contig1_All, CL3041.Contig1_All, CL4430.Contig1_All, CL4675.Contig1_All, CL6396.Contig1_All, CL7655.Contig1_All, CL7655.Contig2_All, CL8200.Contig1_All, CL8651.Contig1_All, CL8804.Contig1_All, CL8979.Contig1_All, CL927.Contig1_All, CL927.Contig2_All,

		CL927.Contig3_All, CL927.Contig4_All, CL927.Contig5_All, CL927.Contig6_All, CL927.Contig7_All, CL927.Contig8_All, Unigene1760_All
93	Histidine metabolism	CL1029.Contig2_All, CL10740.Contig1_All, CL11004.Contig1_All, CL11625.Contig1_All, CL12498.Contig1_All, CL12705.Contig1_All, CL1275.Contig1_All, CL1412.Contig1_All, CL1412.Contig2_All, CL1544.Contig1_All, CL16239.Contig1_All, CL16473.Contig1_All, CL16543.Contig1_All, CL16786.Contig1_All, CL16871.Contig1_All, CL16878.Contig1_All, CL20788.Contig1_All, CL23375.Contig1_All, CL23687.Contig1_All, CL26676.Contig1_All, CL27162.Contig1_All, CL2929.Contig1_All, CL3113.Contig1_All, CL3457.Contig1_All, CL3581.Contig1_All, CL3581.Contig2_All, CL3581.Contig3_All, CL3581.Contig4_All, CL4137.Contig1_All, CL5187.Contig1_All, CL5237.Contig1_All, CL5549.Contig1_All, CL5549.Contig2_All, CL6321.Contig1_All, CL6337.Contig1_All, CL6371.Contig1_All, CL6998.Contig1_All, CL7314.Contig1_All, CL7833.Contig1_All, CL8407.Contig1_All, CL9067.Contig1_All, CL9106.Contig1_All, Unigene2503_All, Unigene4157_All, Unigene4417_All
94	Pantothenate and CoA biosynthesis	CL11523.Contig1_All, CL1162.Contig1_All, CL11632.Contig1_All, CL11840.Contig1_All, CL11877.Contig1_All, CL12413.Contig1_All, CL13772.Contig1_All, CL14671.Contig1_All, CL14671.Contig2_All, CL14866.Contig1_All, CL15175.Contig1_All, CL15387.Contig1_All, CL17034.Contig1_All, CL17474.Contig1_All, CL201.Contig5_All, CL20673.Contig1_All, CL21155.Contig1_All, CL23231.Contig1_All, CL24033.Contig1_All, CL24405.Contig1_All, CL26462.Contig1_All, CL27255.Contig1_All, CL2996.Contig1_All, CL3118.Contig1_All, CL3170.Contig1_All, CL3914.Contig1_All, CL444.Contig3_All, CL4972.Contig1_All, CL5173.Contig1_All, CL525.Contig4_All, CL525.Contig5_All, CL5538.Contig1_All, CL5596.Contig1_All, CL5678.Contig1_All, CL7040.Contig1_All, CL7202.Contig1_All, CL7796.Contig1_All, CL8288.Contig1_All, CL8638.Contig1_All, CL9151.Contig1_All, CL9385.Contig1_All, Unigene3041_All, Unigene3282_All, Unigene3443_All
		CL10654.Contig1_All, CL10728.Contig1_All, CL10971.Contig1_All, CL11550.Contig1_All, CL11828.Contig1_All, CL12367.Contig1_All, CL15382.Contig1_All, CL15790.Contig1_All, CL16638.Contig1_All, CL17226.Contig1_All, CL20595.Contig1_All, CL21279.Contig1_All, CL2445.Contig1_All, CL24599.Contig1_All, CL26598.Contig1_All, CL285.Contig2_All, CL345.Contig5_All, CL3995.Contig1_All, CL4031.Contig1_All,

95	Riboflavin metabolism	CL4525.Contig1_All, CL4535.Contig1_All, CL522.Contig1_All, CL6319.Contig1_All, CL6319.Contig2_All, CL634.Contig1_All, CL634.Contig3_All, CL634.Contig4_All, CL6705.Contig1_All, CL6984.Contig1_All, CL7057.Contig1_All, CL7057.Contig2_All, CL710.Contig1_All, CL710.Contig2_All, CL710.Contig3_All, CL7315.Contig1_All, CL7617.Contig1_All, CL8337.Contig1_All, CL8859.Contig1_All, CL8896.Contig1_All, CL894.Contig4_All, Unigene2636_All, Unigene4377_All, Unigene5155_All, Unigene5206_All
96	Non-homologous end-joining	CL10045.Contig1_All, CL105.Contig10_All, CL11511.Contig1_All, CL12513.Contig1_All, CL13424.Contig1_All, CL1506.Contig1_All, CL16014.Contig1_All, CL16039.Contig1_All, CL16850.Contig1_All, CL17819.Contig1_All, CL19194.Contig1_All, CL19688.Contig1_All, CL20456.Contig1_All, CL20899.Contig1_All, CL21122.Contig1_All, CL23844.Contig1_All, CL23893.Contig1_All, CL23965.Contig1_All, CL25493.Contig1_All, CL26925.Contig1_All, CL2814.Contig2_All, CL3023.Contig1_All, CL3929.Contig1_All, CL4114.Contig1_All, CL4114.Contig2_All, CL449.Contig3_All, CL5037.Contig1_All, CL5344.Contig1_All, CL5699.Contig1_All, CL5818.Contig1_All, CL642.Contig1_All, CL6567.Contig1_All, CL6633.Contig1_All, CL6936.Contig1_All, CL6997.Contig1_All, CL8251.Contig1_All, CL8425.Contig1_All, CL9247.Contig1_All, CL93.Contig2_All, Unigene434_All, Unigene537_All
97	Benzoxazinoid biosynthesis	CL10035.Contig1_All, CL10594.Contig1_All, CL12207.Contig1_All, CL12714.Contig1_All, CL14941.Contig1_All, CL1591.Contig1_All, CL1591.Contig2_All, CL1591.Contig3_All, CL1591.Contig4_All, CL1591.Contig5_All, CL17004.Contig1_All, CL17693.Contig1_All, CL181.Contig4_All, CL26021.Contig1_All, CL26083.Contig1_All, CL26274.Contig1_All, CL2664.Contig1_All, CL2672.Contig1_All, CL4224.Contig1_All, CL4471.Contig1_All, CL4876.Contig1_All, CL5020.Contig1_All, CL5020.Contig2_All, CL511.Contig1_All, CL511.Contig3_All, CL511.Contig5_All, CL6929.Contig1_All, CL7621.Contig1_All, CL8215.Contig1_All, CL9435.Contig1_All, Unigene3708_All, Unigene3751_All, Unigene3756_All, Unigene3764_All, Unigene3838_All, Unigene4370_All, Unigene793_All
98	Indole alkaloid biosynthesis	CL10334.Contig1_All, CL10334.Contig2_All, CL10366.Contig1_All, CL13520.Contig1_All, CL1729.Contig1_All, CL1729.Contig2_All, CL1729.Contig3_All, CL1729.Contig4_All, CL17967.Contig1_All, CL18268.Contig1_All, CL19070.Contig1_All, CL20571.Contig1_All, CL21086.Contig1_All, CL21133.Contig1_All, CL21272.Contig1_All, CL2323.Contig1_All, CL2323.Contig2_All, CL23297.Contig1_All, CL23997.Contig1_All, CL2485.Contig1_All,

		CL2536.Contig1_All, CL2536.Contig2_All, CL2643.Contig1_All, CL2643.Contig2_All, CL2643.Contig3_All, CL27714.Contig1_All, CL5201.Contig1_All, CL5877.Contig1_All, CL6402.Contig1_All, CL6501.Contig1_All, CL6867.Contig1_All, CL6946.Contig1_All, CL8700.Contig1_All, CL9749.Contig1_All, Unigene1484_All, Unigene4275_All, Unigene990_All
99	Circadian rhythm - mammal	CL10.Contig16_All, CL10.Contig19_All, CL10.Contig20_All, CL10.Contig21_All, CL10.Contig22_All, CL10.Contig23_All, CL10.Contig24_All, CL10.Contig25_All, CL10.Contig26_All, CL10.Contig27_All, CL10.Contig28_All, CL10.Contig29_All, CL10.Contig2_All, CL10.Contig4_All, CL10.Contig5_All, CL10.Contig8_All, CL10177.Contig1_All, CL11297.Contig1_All, CL13508.Contig1_All, CL1454.Contig1_All, CL15482.Contig1_All, CL15797.Contig1_All, CL2271.Contig1_All, CL2271.Contig2_All, CL2271.Contig3_All, CL616.Contig2_All, CL616.Contig3_All, CL616.Contig4_All, CL7106.Contig1_All, CL9475.Contig1_All, CL9709.Contig1_All, Unigene2221_All, Unigene260_All, Unigene3020_All, Unigene639_All
100	Isoquinoline alkaloid biosynthesis	CL10238.Contig1_All, CL10568.Contig1_All, CL10994.Contig1_All, CL12133.Contig1_All, CL12720.Contig1_All, CL13936.Contig1_All, CL17459.Contig1_All, CL17459.Contig2_All, CL18250.Contig1_All, CL2043.Contig1_All, CL21082.Contig1_All, CL21286.Contig1_All, CL22411.Contig1_All, CL23294.Contig1_All, CL25992.Contig1_All, CL26958.Contig1_All, CL2876.Contig1_All, CL4055.Contig1_All, CL504.Contig1_All, CL504.Contig2_All, CL504.Contig3_All, CL504.Contig4_All, CL504.Contig5_All, CL5787.Contig1_All, CL6401.Contig1_All, CL8297.Contig1_All, CL8386.Contig1_All, CL8697.Contig1_All, CL908.Contig1_All, CL908.Contig2_All, CL908.Contig3_All, CL908.Contig4_All, Unigene1045_All, Unigene3071_All, Unigene3611_All
101	Photosynthesis - antenna proteins	CL10319.Contig1_All, CL10694.Contig1_All, CL10701.Contig1_All, CL1076.Contig1_All, CL1076.Contig2_All, CL1076.Contig3_All, CL1076.Contig4_All, CL11036.Contig1_All, CL11076.Contig1_All, CL11385.Contig1_All, CL11540.Contig1_All, CL1203.Contig1_All, CL1203.Contig2_All, CL1203.Contig3_All, CL1203.Contig4_All, CL12279.Contig1_All, CL13162.Contig1_All, CL14543.Contig1_All, CL14560.Contig1_All, CL15072.Contig1_All, CL15286.Contig1_All, CL15546.Contig1_All, CL16125.Contig1_All, CL16275.Contig1_All, CL16293.Contig1_All, CL17274.Contig1_All, CL26108.Contig1_All, CL26750.Contig1_All, CL27620.Contig1_All, CL4849.Contig1_All, CL5042.Contig1_All, Unigene1264_All, Unigene2288_All, Unigene5506_All
		CL1051.Contig1_All, CL1051.Contig2_All,

102	One carbon pool by folate	CL11819.Contig1_All, CL11835.Contig1_All, CL12107.Contig1_All, CL12607.Contig1_All, CL12640.Contig1_All, CL13445.Contig1_All, CL13770.Contig1_All, CL1384.Contig1_All, CL1424.Contig1_All, CL15935.Contig1_All, CL16728.Contig1_All, CL16728.Contig2_All, CL17962.Contig1_All, CL1834.Contig3_All, CL2090.Contig1_All, CL22012.Contig1_All, CL2227.Contig1_All, CL23525.Contig1_All, CL2874.Contig1_All, CL2874.Contig2_All, CL3433.Contig1_All, CL3433.Contig2_All, CL4484.Contig1_All, CL4484.Contig2_All, CL4615.Contig1_All, CL7521.Contig1_All, CL8014.Contig1_All, CL8606.Contig1_All, Unigene2102_All, Unigene2167_All, Unigene4420_All, Unigene4727_All
103	Glycosphingolipid biosynthesis - ganglio series	CL1288.Contig1_All, CL1288.Contig2_All, CL1288.Contig3_All, CL1288.Contig4_All, CL1304.Contig1_All, CL1304.Contig2_All, CL1304.Contig3_All, CL14874.Contig1_All, CL17196.Contig1_All, CL17482.Contig1_All, CL1750.Contig1_All, CL19717.Contig1_All, CL20816.Contig1_All, CL23651.Contig1_All, CL25863.Contig1_All, CL3011.Contig1_All, CL3576.Contig1_All, CL3576.Contig2_All, CL3654.Contig1_All, CL399.Contig1_All, CL399.Contig2_All, CL399.Contig3_All, CL399.Contig4_All, CL399.Contig5_All, CL399.Contig6_All, CL4094.Contig1_All, CL5615.Contig1_All, CL621.Contig1_All, CL621.Contig2_All, CL9192.Contig1_All, Unigene2287_All, Unigene4790_All, Unigene705_All
104	Folate biosynthesis	CL1115.Contig1_All, CL1115.Contig2_All, CL11287.Contig1_All, CL11613.Contig1_All, CL12145.Contig1_All, CL12955.Contig1_All, CL13192.Contig1_All, CL13859.Contig1_All, CL1390.Contig1_All, CL1424.Contig1_All, CL15330.Contig1_All, CL16725.Contig1_All, CL20670.Contig1_All, CL22012.Contig1_All, CL23052.Contig1_All, CL23585.Contig1_All, CL24890.Contig1_All, CL27079.Contig1_All, CL28012.Contig1_All, CL2854.Contig1_All, CL3034.Contig1_All, CL6039.Contig1_All, CL6039.Contig2_All, CL6752.Contig1_All, CL6772.Contig1_All, CL7678.Contig1_All, Unigene1165_All, Unigene2102_All, Unigene2470_All, Unigene2714_All, Unigene3395_All, Unigene3917_All, Unigene4899_All
105	Diterpenoid biosynthesis	CL10035.Contig1_All, CL10217.Contig1_All, CL12059.Contig1_All, CL12949.Contig1_All, CL13586.Contig1_All, CL1529.Contig1_All, CL1529.Contig2_All, CL1529.Contig3_All, CL1529.Contig4_All, CL1529.Contig5_All, CL20574.Contig1_All, CL21539.Contig1_All, CL21674.Contig1_All, CL2205.Contig1_All, CL22167.Contig1_All, CL2359.Contig1_All, CL25606.Contig1_All, CL26273.Contig1_All, CL27979.Contig1_All, CL4445.Contig1_All,

		CL4836.Contig1_All, CL530.Contig1_All, CL530.Contig2_All, CL530.Contig3_All, CL6914.Contig1_All, CL7697.Contig1_All, CL932.Contig1_All, CL932.Contig2_All, CL932.Contig3_All, CL932.Contig4_All, Unigene3637_All, Unigene3826_All, Unigene5264_All
106	Nicotinate and nicotinamide metabolism	CL12173.Contig1_All, CL131.Contig1_All, CL131.Contig2_All, CL131.Contig3_All, CL131.Contig4_All, CL131.Contig5_All, CL131.Contig6_All, CL131.Contig7_All, CL1326.Contig1_All, CL1326.Contig2_All, CL14053.Contig1_All, CL1455.Contig1_All, CL1455.Contig2_All, CL1455.Contig3_All, CL18261.Contig1_All, CL21365.Contig1_All, CL24148.Contig1_All, CL2686.Contig1_All, CL4030.Contig1_All, CL4048.Contig1_All, CL5175.Contig1_All, CL582.Contig1_All, CL6042.Contig1_All, CL6454.Contig1_All, CL6454.Contig2_All, CL6557.Contig1_All, CL6557.Contig2_All, CL8262.Contig1_All, CL8438.Contig1_All, CL8588.Contig1_All, Unigene2660_All
107	Selenocompound metabolism	CL10623.Contig1_All, CL10685.Contig1_All, CL10964.Contig1_All, CL11669.Contig1_All, CL12750.Contig1_All, CL12938.Contig1_All, CL13025.Contig1_All, CL13063.Contig1_All, CL13451.Contig1_All, CL13634.Contig1_All, CL13721.Contig1_All, CL15323.Contig1_All, CL16751.Contig1_All, CL16932.Contig1_All, CL17356.Contig1_All, CL23320.Contig1_All, CL24088.Contig1_All, CL3412.Contig1_All, CL3901.Contig1_All, CL4011.Contig1_All, CL4507.Contig1_All, CL475.Contig1_All, CL475.Contig2_All, CL475.Contig3_All, CL475.Contig4_All, CL5017.Contig1_All, CL5500.Contig1_All, CL5500.Contig2_All, CL6395.Contig1_All, Unigene4864_All
108	Vitamin B6 metabolism	CL10887.Contig1_All, CL11260.Contig1_All, CL11284.Contig1_All, CL11453.Contig1_All, CL11881.Contig1_All, CL13895.Contig1_All, CL17648.Contig1_All, CL18778.Contig1_All, CL188.Contig1_All, CL188.Contig2_All, CL188.Contig3_All, CL4775.Contig1_All, CL5598.Contig1_All, CL6259.Contig1_All, CL6559.Contig1_All, CL757.Contig1_All, CL757.Contig3_All, CL757.Contig4_All, CL805.Contig1_All, CL805.Contig2_All, CL805.Contig3_All, CL805.Contig4_All, CL805.Contig5_All, CL8758.Contig1_All, CL9044.Contig1_All, CL9848.Contig1_All, Unigene1453_All, Unigene1600_All, Unigene4011_All, Unigene4510_All
109	Glycosphingolipid biosynthesis - globo series	CL13284.Contig1_All, CL1428.Contig1_All, CL1428.Contig2_All, CL1428.Contig3_All, CL1428.Contig4_All, CL1428.Contig5_All, CL1428.Contig6_All, CL1428.Contig7_All, CL14874.Contig1_All, CL19694.Contig1_All, CL26011.Contig1_All, CL3011.Contig1_All, CL4094.Contig1_All, CL5615.Contig1_All, CL5638.Contig1_All, CL6830.Contig1_All, CL9192.Contig1_All, CL9200.Contig1_All, Unigene2545_All, Unigene3777_All, Unigene4889_All, Unigene4932_All,

		Unigene4933_All, Unigene5049_All, Unigene5436_All
110	Linoleic acid metabolism	CL10072.Contig2_All, CL10382.Contig1_All, CL1172.Contig1_All, CL1172.Contig2_All, CL13964.Contig1_All, CL1679.Contig1_All, CL18800.Contig1_All, CL21677.Contig1_All, CL24045.Contig1_All, CL3031.Contig1_All, CL3509.Contig1_All, CL3991.Contig1_All, CL4716.Contig1_All, CL4716.Contig2_All, CL627.Contig1_All, CL6744.Contig1_All, CL759.Contig1_All, CL759.Contig2_All, CL759.Contig3_All, CL759.Contig4_All, CL759.Contig5_All, CL759.Contig6_All, CL8720.Contig1_All, Unigene3346_All
111	Other types of O-glycan biosynthesis (no map in kegg database)	CL1116.Contig1_All, CL1116.Contig2_All, CL1541.Contig1_All, CL1985.Contig1_All, CL19918.Contig1_All, CL20153.Contig1_All, CL2149.Contig1_All, CL2169.Contig1_All, CL25994.Contig1_All, CL27241.Contig1_All, CL3369.Contig1_All, CL3369.Contig2_All, CL377.Contig1_All, CL4279.Contig2_All, CL4936.Contig1_All, CL507.Contig2_All, CL538.Contig1_All, CL538.Contig2_All, CL8733.Contig1_All, CL8956.Contig1_All, CL936.Contig4_All
112	Brassinosteroid biosynthesis	CL12250.Contig1_All, CL13586.Contig1_All, CL1523.Contig1_All, CL15336.Contig1_All, CL18320.Contig1_All, CL18450.Contig1_All, CL2.Contig1_All, CL2.Contig22_All, CL2.Contig35_All, CL22454.Contig1_All, CL23002.Contig1_All, CL23391.Contig1_All, CL24081.Contig1_All, CL24683.Contig1_All, CL26066.Contig1_All, CL3131.Contig2_All, CL5443.Contig1_All, CL6214.Contig1_All, Unigene961_All
113	Arachidonic acid metabolism	CL11812.Contig1_All, CL12617.Contig1_All, CL13250.Contig1_All, CL13662.Contig1_All, CL14039.Contig1_All, CL14370.Contig1_All, CL1679.Contig1_All, CL18280.Contig1_All, CL18800.Contig1_All, CL22379.Contig1_All, CL2242.Contig1_All, CL2429.Contig1_All, CL4391.Contig1_All, CL7435.Contig1_All, CL8911.Contig1_All, CL937.Contig1_All, Unigene4542_All, Unigene5280_All
114	Sulfur relay system	CL11164.Contig1_All, CL13155.Contig1_All, CL13884.Contig1_All, CL14116.Contig1_All, CL14638.Contig1_All, CL14753.Contig1_All, CL15330.Contig1_All, CL1884.Contig1_All, CL2854.Contig1_All, CL2857.Contig1_All, CL2857.Contig2_All, CL3126.Contig1_All, CL6039.Contig1_All, CL6039.Contig2_All, CL6605.Contig1_All, CL6772.Contig1_All, CL9098.Contig1_All, CL9657.Contig1_All
115	Fatty acid elongation	CL10901.Contig1_All, CL11465.Contig1_All, CL16319.Contig1_All, CL2.Contig25_All, CL21695.Contig1_All, CL21729.Contig1_All, CL24005.Contig1_All, CL24371.Contig1_All, CL25688.Contig1_All, CL27710.Contig1_All, CL569.Contig2_All, CL569.Contig5_All, CL569.Contig6_All, CL7574.Contig1_All, Unigene3064_All, Unigene790_All,

		Unigene920_All
116	Taurine and hypotaurine metabolism	CL10292.Contig1_All, CL11812.Contig1_All, CL12156.Contig1_All, CL12346.Contig1_All, CL2429.Contig1_All, CL5138.Contig1_All, CL768.Contig1_All, CL768.Contig2_All, CL768.Contig3_All, CL768.Contig4_All, CL768.Contig5_All, CL768.Contig6_All, CL968.Contig1_All, Unigene193_All, Unigene5280_All, Unigene874_All
117	Thiamine metabolism	CL13155.Contig1_All, CL13275.Contig1_All, CL1526.Contig1_All, CL1526.Contig2_All, CL2647.Contig1_All, CL3983.Contig1_All, CL3983.Contig2_All, CL4507.Contig1_All, CL4537.Contig1_All, CL7254.Contig1_All, Unigene2683_All, Unigene3029_All, Unigene3427_All
118	C5-Branched dibasic acid metabolism	CL11779.Contig1_All, CL11840.Contig1_All, CL11877.Contig1_All, CL13338.Contig1_All, CL15125.Contig1_All, CL15367.Contig1_All, CL21307.Contig1_All, CL71.Contig2_All, CL71.Contig3_All, CL7202.Contig1_All, CL7261.Contig1_All, CL7261.Contig2_All, CL8261.Contig1_All
119	Caffeine metabolism	CL13574.Contig1_All, CL1559.Contig1_All, CL1559.Contig2_All, CL18360.Contig1_All, CL19200.Contig1_All, CL24617.Contig1_All, CL27926.Contig1_All, CL4975.Contig1_All, CL656.Contig1_All, CL656.Contig2_All, CL656.Contig3_All, CL656.Contig4_All, Unigene4058_All
120	Monoterpenoid biosynthesis	CL10433.Contig1_All, CL12410.Contig1_All, CL16109.Contig1_All, CL19263.Contig1_All, CL23042.Contig1_All, CL4174.Contig1_All, CL4865.Contig1_All, CL4865.Contig2_All, CL5942.Contig1_All, CL8054.Contig1_All
121	Anthocyanin biosynthesis	CL12265.Contig1_All, CL14229.Contig1_All, CL22440.Contig1_All, CL5238.Contig1_All, CL5495.Contig1_All, CL6877.Contig1_All, CL7918.Contig2_All, Unigene1470_All, Unigene1482_All
122	Synthesis and degradation of ketone bodies	CL10551.Contig1_All, CL12238.Contig1_All, CL12277.Contig1_All, CL32.Contig12_All, CL3448.Contig1_All, CL3448.Contig2_All, CL8172.Contig1_All, Unigene1434_All
123	Biotin metabolism	CL11845.Contig1_All, CL12463.Contig1_All, CL16853.Contig1_All, CL18849.Contig1_All, CL3999.Contig1_All, Unigene4152_All, Unigene683_All
124	Lipoic acid metabolism	CL10200.Contig1_All, CL19193.Contig1_All, CL19659.Contig1_All, CL25441.Contig1_All, CL6290.Contig1_All
125	Glycosphingolipid biosynthesis - lacto and neolacto series	CL19178.Contig1_All
126	Sesquiterpenoid biosynthesis	CL10217.Contig1_All
