# Supplementary material for: Transcriptome Analysis of Barbarea vulgaris Infested with Diamondback Moth (Plutella xylostella) Larvae
Source: PLoS One. 2013 May 16;8(5):e64481. doi: 10.1371/journal.pone.0064481 (PMC3655962; doi:10.1371/journal.pone.0064481)
Supplement: Text S1 — Alignment of cDNA cloning and Illumina sequencing of β-AS gene. (PDF) [file pone.0064481.s010.pdf]

Alignment of cDNA cloning and Illumina sequencing of β-AS gene

```
bAS-cDNA               -------------------------------------------ATTGGTGATAAAGGCAA
CL2531.Contig7_All     GTTACCTCTATATAAGCGTACGTACATGAAACTCTTCCAAAGTATTGGTGATAAAGGCAA
                                                                  *****************

bAS-cDNA               ACATCGACCCAGCACAAATCATCATTATGTGGAGGTTGAAGTTAGGAGAGGGAAATGGAG
CL2531.Contig7_All     ACATCGACCCAGCACAAATCATCATTATGTGGAGGTTGAAGTTAGGAGAGGGAAATGGAG
                       ************************************************************

bAS-cDNA               ACGACCCTTACTTGTTCAGCAGCAACAATTTCGTCGGACGTCAAACATGGGAATTTGATC
CL2531.Contig7_All     ACGACCCTTACTTGTTCAGCAGCAACAATTTCGTCGGACGTCAAACATGGGAATTTGATC
                       ************************************************************

bAS-cDNA               CCAAAGCCGGCACACTGGAGGAACGAGCCGCCGTTGAAGAAGCTCGCCGGAGTTTCTTGG
CL2531.Contig7_All     CCAAAGCCGGCACACTGGAGGAACGAGCCGCCGTTGAAGAAGCTCGCCGGAGTTTCTTGG
                       ************************************************************

bAS-cDNA               TTAACCGTTCTCGTGTTAAAGCTTGCAGTGATCTCTTGTGGCGAATGCAATTTTTGAAAG
CL2531.Contig7_All     TTAACCGTTCTCGTGTTAAAGCTTGCAGTGATCTCTTGTGGCGAATGCAATTTTTGAAAG
                       ************************************************************

bAS-cDNA               AGGCAAAATTCGAACAAGTGATCCCGCCGGTGAAGATTGAAGACGCCAAAGACATAACTT
CL2531.Contig7_All     AGGCAAAATTCGAACAAGTGATCCCGCCGGTGAAGATTGAAGACGCCAAAGACATAACTT
                       ************************************************************

bAS-cDNA               ATGAAAATGCGACGGATTCATTACGGAGAGGAGTTTCATTCTTCTCAGCTTTGCAAGCTT
CL2531.Contig7_All     ATGAAAATGCGACGGATTCATTACGGAGAGGAGTTTCATTCTTCTCAGCTTTGCAAGCTT
                       ************************************************************

bAS-cDNA               CCGATGGCCACTGGCCAGGAGAAATCGCCGGACCTCTCTTCTTCCTTCCTCCATTGGTAT
CL2531.Contig7_All     CCGATGGCCACTGGCCAGGAGAAATCGCCGGACCTCTCTTCTTCCTTCCTCCATTGGTAT
                       ************************************************************

bAS-cDNA               TTTGTTTATATATCACAGGACACCTCGAAGAGATATTCGATGAAGAACATCGCAAAGAGA
CL2531.Contig7_All     TTTGTTTATATATCACAGGACACCTCGAAGAGATATTCGATGAAGAACATCGCAAAGAGA
                       ************************************************************

bAS-cDNA               TGCTTCGACATGTCTATTGTCATCAGAACGAAGATGGTGGATGGGGATTACACGTTGAGA
CL2531.Contig7_All     TGCTTCGACATGTCTATTGTCATCAGAACGAAGATGGTGGATGGGGATTACACGTTGAGA
                       ************************************************************

bAS-cDNA               GCAAGAGCATTATGTTTTGCACCGTGTTGAATTACATATGCTTACGTATGCTCGGTGAAG
CL2531.Contig7_All     GCAAGAGCATTATGTTTTGCACCGTGTTGAATTACATATGCTTACGTATGCTCGGTGAAG
                       ************************************************************

bAS-cDNA               GTCCTAATGGAGGACGAGATAACGCATGTAAACGAGCTAGACAATGGATTCTTGATCGTG
CL2531.Contig7_All     GTCCTAATGGAGGACGAGATAACGCATGTAAACGAGCTAGACAATGGATTCTTGATCGTG
                       ************************************************************

bAS-cDNA               GTGGTGTTACCTATATTCCTTCATGGGGAAAAATTTGGTTATCGATACTTGGAATCTATG
CL2531.Contig7_All     GTGGTGTTACCTATATTCCTTCATGGGGAAAAATTTGGTTATCGATACTTGGAATCTATG
                       ************************************************************

bAS-cDNA               ATTGGTCCGGAACCAATCCAATGCCTCCTGAGATTTGGTTACTACCTTCTTTTGTTCCTA
CL2531.Contig7_All     ATTGGTCCGGAACCAATCCAATGCCTCCTGAGATTTGGTTACTACCTTCTTTTGTTCCTA
                       ************************************************************

bAS-cDNA               TACACTTAGCGAAAACTTTGTGCTATTGCCGAATGGTGTACATGCCAATGTCATATCTAT
CL2531.Contig7_All     TACACTTAGCGAAAACTTTGTGCTATTGCCGAATGGTGTACATGCCAATGTCATATCTAT
                       ************************************************************

bAS-cDNA               ATGGGAAGAGATTTGTTGGTCCAATAACACCTCTTATTCTACAACTTCGTGAAGAACTTC
CL2531.Contig7_All     ATGGGAAGAGATTTGTTGGTCCAATAACACCTCTTATTCTACAACTTCGTGAAGAACTTC
                       ************************************************************

bAS-cDNA               ATTTACAACCTTATGAAGCAATCAACTGGAATAAAACGCGGCGTTTATATGCTAAGGAGG
CL2531.Contig7_All     ATTTACAACCTTATGAAGCAATCAACTGGAATAAAACGCGGCGTTTATATGCTAAGGAGG
                       ************************************************************

bAS-cDNA               ATATGTATTTTCCTCATCCTTTGGTTCAAGATTTGATATGGGACACTCTTCACATCTTTG
CL2531.Contig7_All     ATATGTATTTTCCTCATCCTTTGGTTCAAGATTTGATATGGGACACTCTTCACATCTTTG
                       ************************************************************

bAS-cDNA               TGGAGCCGTTACTTACTCATTGGCCGTTAAACAAGCTTGTGAGGGAAAAAGCTCTTCGGC
CL2531.Contig7_All     TGGAGCCGTTACTTACTCATTGGCCGTTAAACAAGCTTGTGAGGGAAAAAGCTCTTCGGC
                       ************************************************************

bAS-cDNA               TAGCAATGAAACACATACATTATGAAGATGAAAATAGCCATTATATTACCATTGGATGTG
CL2531.Contig7_All     TAGCAATGAAACACATACATTATGAAGATGAAAATAGCCATTATATTACCATTGGATGTG
                       ************************************************************

bAS-cDNA               TTGAGAAGGTATTGTGCATGCTTGCTTGTTGGATTGATGACCCTAATGGGGATTACTTCA
CL2531.Contig7_All     TTGAGAAGGTATTGTGCATGCTTGCTTGTTGGATTGATGACCCTAATGGGGATTACTTCA
                       ************************************************************
```

```
bAS-cDNA              AAAAGCATCTTGCTAGAATTCCAGATTACATGTGGGTAGCCGAAGATGGAATGAAAATGC
CL2531.Contig7_All    AAAAGCATCTTGCTAGAATTCCAGATTACATGTGGGTAGCCGAAGATGGAATGAAAATGC
                      ************************************************************

bAS-cDNA              AGAGCTTTGGAAGTCAACAATGGGATACAGGGTTTGCGGTTCAAGCTATAATTGCTAGTG
CL2531.Contig7_All    AGAGCTTTGGAAGTCAACAATGGGATACAGGGTTTGCGGTTCAAGCTATAATTGCTAGTG
                      ************************************************************

bAS-cDNA              ATCTCTCTAGTGAAACCGGCGATGTACTTAAGAGAGGACATGATTACATTAAGAAATCTC
CL2531.Contig7_All    ATCTCTCTAGTGAAACCGGCGATGTACTTAAGAGAGGACATGATTACATTAAGAAATCTC
                      ************************************************************

bAS-cDNA              AGATTAGAGAGAATCCTTCTGGTGACTTTAAGAGTATGTACCGTCACATATCCAAAGGAG
CL2531.Contig7_All    AGATTAGAGAGAATCCTTCTGGTGACTTTAAGAGTATGTACCGTCACATATCCAAAGGAG
                      ************************************************************

bAS-cDNA              CATGGACTTTATCTGATCGAGATCATGGATGGCAAGTTTCAGACTGTACAGCTGAAGCTT
CL2531.Contig7_All    CATGGACTTTATCTGATCGAGATCATGGATGGCAAGTTTCAGACTGTACAGCTGAAGCTT
                      ************************************************************

bAS-cDNA              TGAAGTGTTGCCTGCTGCTTTCTATGATGCCAGCTGAAGTTGTTGGCCATAAAATGGATC
CL2531.Contig7_All    TGAAGTGTTGCCTGCTGCTTTCTATGATGCCAGCTGAAGTTGTTGGCCATAAAATGGATC
                      ************************************************************

bAS-cDNA              CCGAACAGCTCTATGATTCTGTTAATCTCTTGCTATCTTTACAGAGTGCAAATGGAGGTG
CL2531.Contig7_All    CCGAACAGCTCTATGATTCTGTTAATCTCTTGCTATCTTTACAGAGTGCAAATGGAGGTG
                      ************************************************************

bAS-cDNA              TGACTGCATGGGAGCCTGTCCGCGCATATGCATGGACTGAATTGCTTAATCCGACAGAAT
CL2531.Contig7_All    TGACTGCATGGGAGCCTGTCCGCGCATATGCATGGACTGAATTGCTTAATCCGACAGAAT
                      ************************************************************

bAS-cDNA              TTCTGGCTAATCTTGTGGCTGAGCGTGAGTACGTGGAATGTACCTCATCTGTTGTACAAG
CL2531.Contig7_All    TTCTGGCTAATCTTGTGGCTGAGCGTGAGTACGTGGAATGTACCTCATCTGTTGTACAAG
                      ************************************************************

bAS-cDNA              CTTTGGTTCTATTCCAGCAACTCTATCCAGATCATAAGACAAAAAAGATCAGCAGGGCCA
CL2531.Contig7_All    CTTTGGTTCTATTCCAGCAACTCTATCCAGATCATAAGACAAAAAAGATCAGCAGGGCCA
                      ************************************************************

bAS-cDNA              TCGAGAAAGCGGTGCAATTCTTAGAAAACGAACAAAAGCCAGATGGTTCATGGTATGGAA
CL2531.Contig7_All    TCGAGAAAGCGGTGCAATTCTTAGAAAACGAACAAAAGCCAGATGGTTCATGGTATGGAA
                      ************************************************************

bAS-cDNA              ATTGGGGTGTTTGTTTCATATATGCGACATGGTTTGCTCTTGGTGGCCTAGCAGCTGCTG
CL2531.Contig7_All    ATTGGGGTGTTTGTTTCATATATGCGACATGGTTTGCTCTTGGTGGCCTAGCAGCTGCTG
                      ************************************************************

bAS-cDNA              GTAAAACATATAAAACTAGCCAGGCGATGCGTAAAGGTGTTGAGTTTTTACTCACGACAC
CL2531.Contig7_All    GTAAAACATATAAAACTAGCCAGGCGATGCGTAAAGGTGTTGAGTTTTTACTCACGACAC
                      ************************************************************

bAS-cDNA              AGAAAGATGATGGAGGTTGGGGTGAAAGCTATCTGTCATGCCCTGAACAGAGATACATAC
CL2531.Contig7_All    AGAAAGATGATGGAGGTTGGGGTGAAAGCTATCTGTCATGCCCTGAACAGAGATACATAC
                      ************************************************************

bAS-cDNA              CATTAGAAGGGAATAGATCCAACCTGGTGCAAACCGCTTGGGCAATAATGGGGTTGATTC
CL2531.Contig7_All    CATTAGAAGGGAATAGATCCAACCTGGTGCAAACCGCTTGGGCAATAATGGGGTTGATTC
                      ************************************************************

bAS-cDNA              ACGCCGGACAGGCCGAGAGAGATCCAATACCTCTTCACCGTGCTGCGAAACTTATCATCA
CL2531.Contig7_All    ACGCCGGACAGGCCGAGAGAGATCCAATACCTCTTCACCGTGCTGCGAAACTTATCATCA
                      ************************************************************

bAS-cDNA              ATTCGCAAATGGAAAATGGAGATTTCCCTCAACAGGAAATAGTAGGAGTGTTCATGAGGA
CL2531.Contig7_All    ATTCGCAAATGGAAAATGGAGATTTCCCTCAACAGGAAATAGTAGGAGTGTTCATGAGGA
                      ************************************************************

bAS-cDNA              ATTGCTTGTTACACTATGCAACCTTCAGAAACACTTTCCCATTATGGGCACTTGCCGAGT
CL2531.Contig7_All    ATTGCTTGTTACACTATGCAACCTTCAGAAACACTTTCCCATTATGGGCACTTGCCGAGT
                      ************************************************************

bAS-cDNA              ACCGAAAAGCTGCGTTCGTCACTCATAAACATTAATTAGTGTATCTGGGAGGAATAACAC
CL2531.Contig7_All    ACCGAAAAGCTGCGTTCGTCACTCATAAACATTAATTAGTGTATCTGGGAGGAATAACAC
                      ************************************************************

bAS-cDNA              ATACATTATCAAGACGAAATTAGCCATTAGGCAAAACATTTAAGGACCCTTTAATGTAAA
CL2531.Contig7_All    ATACATTATCAAGACGAAATTAGCCATTAGGCAAAACATTTAAGGACCCTTTAATGTAAA
                      ************************************************************

bAS-cDNA              CATATATAAAAAGATGTTTATATTCTCTTAAGAGTAATGTACATATTCAAGAAGCATCTA
CL2531.Contig7_All    CATATATAAAAAGATGTTTATATTCTCTTAAGAGTAATGTACATATTCAAGAAGCATCTA
                      ************************************************************

bAS-cDNA              GCTAGAATTCAGAAGATGGAATGAAAATGTAGGAAGTAATTGTTTGTTGCAGTGGACTTC
CL2531.Contig7_All    GCTAGAATTCAGAAGATGGAATGAAAATGTAGGAAGTAATTGTTTGTTGCAGTGGACTTC
                      ************************************************************
```

```
bAS-cDNA                AAACATAGAAAAAAAAAAAAAAAAAAAAAAAA
CL2531.Contig7_All      --------------------------------
```

Alignment of cDNA cloning and Illumina sequencing of UGT gene

```
UGT74-cDNA              ------------------------------------------------------------
CL12550.Contig1_All     CTTTTAAATCTTTCCTTCTCTGTCGTCTTCTTCAATTGAACCAAAACACTCTTATCAAAA

UGT74-cDNA              -------------------------ATGGCGGAAACAACTAACAAACTCAAACGCCACGT
CL12550.Contig1_All     GCTCTGCTTCTTTGAGCTCACTACAATGGCGGAAACAACTAACAAACTCAAACGCCACGT
                                                 ***********************************

UGT74-cDNA              CCTAGTCTTACCATACCCAGTTCAAGGCCACATTAACCCAATGCTTCAATTCGCTAAACG
CL12550.Contig1_All     CCTAGTCTTACCATACCCAGTTCAAGGCCACATTAACCCAATGCTTCAATTCGCTAAACG
                        ************************************************************

UGT74-cDNA              TCTAGTCTCCAAAAACGTCAAAGTCACAATCGCCACCACAACCTACACAGTCTCCTCAAT
CL12550.Contig1_All     TCTAGTCTCCAAAAACGTCAAAGTCACAATCGCCACCACAACCTACACAGTCTCCTCAAT
                        ************************************************************

UGT74-cDNA              CACAACTCCATCAGTCTCCGTCGAACCAATCTCCGACGGATTCGATTCAATCCCCTTAGC
CL12550.Contig1_All     CACAACTCCATCAGTCTCCGTCGAACCAATCTCCGACGGATTCGATTCAATCCCCTTAGC
                        ************************************************************

UGT74-cDNA              TATCCCCGGTTTCAGCGTCGATGTCTTCTCGGAATCCTTCAAACTCAACGGATCCGAAAC
CL12550.Contig1_All     TATCCCCGGTTTCAGCGTCGATGTCTTCTCGGAATCCTTCAAACTCAACGGATCCGAAAC
                        ************************************************************

UGT74-cDNA              CCTAACTCGCCTAATCGAGAAATTCAAATCCACCGATTCACCAATCGATTGCTTAGTCTA
CL12550.Contig1_All     CCTAACTCGCCTAATCGAGAAATTCAAATCCACCGATTCACCAATCGATTGCTTAGTCTA
                        ************************************************************

UGT74-cDNA              CGATTCGTTTCTTCCTTGGGGACTCGAAGTCGCTAGATCTATGGAGATCTCAGGTGCTTC
CL12550.Contig1_All     CGATTCGTTTCTTCCTTGGGGACTCGAAGTCGCTAGATCTATGGAGATCTCAGGTGCTTC
                        ************************************************************

UGT74-cDNA              GTTCTTCACTAACAATCTCTCTGTTTGTTCTGTGCTTCGTAAATTCGCTAACGGAACTTT
CL12550.Contig1_All     GTTCTTCACTAACAATCTCTCTGTTTGTTCTGTGCTTCGTAAATTCGCTAACGGAACTTT
                        ************************************************************

UGT74-cDNA              TCCTCTTCCCGCCGATCCTGATTCGGCGCCGTTTTTGACACGTGGCTTGCCGTCGTTGAG
CL12550.Contig1_All     TCCTCTTCCCGCCGATCCTGATTCGGCGCCGTTTTTGACACGTGGCTTGCCGTCGTTGAG
                        ************************************************************

UGT74-cDNA              TTACGATGAGTTACCTTCGTTTGTTGGACGTCATTGGTTGACTCATCCTGGGCATGGGAG
CL12550.Contig1_All     TTACGATGAGTTACCTTCGTTTGTTGGACGTCATTGGTTGACTCATCCTGGGCATGGGAG
                        ************************************************************

UGT74-cDNA              AGTGCTTCTGGATCAGTTTCCTAATCATGAAAATGCTGATTGGTTATTCGTTAATGGCTT
CL12550.Contig1_All     AGTGCTTCTGGATCAGTTTCCTAATCATGAAAATGCTGATTGGTTATTCGTTAATGGCTT
                        ************************************************************

UGT74-cDNA              TGAAGGCTTAGAAACACAAGATTGTGAAACTGGTGAATCAGAGGCAATGAGGGCTACGTT
CL12550.Contig1_All     TGAAGGCTTAGAAACACAAGATTGTGAAACTGGTGAATCAGAGGCAATGAGGGCTACGTT
                        ************************************************************

UGT74-cDNA              GATAGGACCGATGATTCCATCTGCATATCTTGATGGTCGTATAAAAGATGATAAAGACTA
CL12550.Contig1_All     GATAGGACCGATGATTCCATCTGCATATCTTGATGGTCGTATAAAAGATGATAAAGACTA
                        ************************************************************

UGT74-cDNA              TGGTGCGAGTCTCTTGAAACCGGTTTCAGAGGAATGTATGAAGTGGCTTGGGACTAAGCC
CL12550.Contig1_All     TGGTGCGAGTCTCTTGAAACCGGTTTCAGAGGAATGTATGAAGTGGCTTGGGACTAAGCC
                        ************************************************************

UGT74-cDNA              GACTCAGTCTGTAGTGTTTGTCTCGTTTGGTTCCTTTGGTGTTCTCTTTGAGAAACAACT
CL12550.Contig1_All     GACTCAGTCTGTAGTGTTTGTCTCGTTTGGTTCCTTTGGTGTTCTCTTTGAGAAACAACT
                        ************************************************************

UGT74-cDNA              CGTAGAGATAGCAGTCGCTTTACAAGAATCGGACTTGAACTTCTTGTGGGTGATTAAAGA
CL12550.Contig1_All     CGTAGAGATAGCAGTCGCTTTACAAGAATCGGACTTGAACTTCTTGTGGGTGATTAAAGA
                        ************************************************************

UGT74-cDNA              AGCTCATGTAGCGAAATTGCCAGAAGGGTTTGTGGAATCGACTAAAGATAGAGCGTTGTT
CL12550.Contig1_All     AGCTCATGTAGCGAAATTGCCAGAAGGGTTTGTGGAATCGACTAAAGATAGAGCGTTGTT
                        ************************************************************

UGT74-cDNA              GGTTTCTTGGGTTAACCAGCTTGAGGTTTTAGCTCATGAATCGATCGGTTGCTTTTTGAC
CL12550.Contig1_All     GGTTTCTTGGGTTAACCAGCTTGAGGTTTTAGCTCATGAATCGATCGGTTGCTTTTTGAC
                        ************************************************************
```

```
UGT74-cDNA              TCATTGTGGTTGGAACTCTACATTGGAAGGTTTGAGTTTGGGTGTGCCAATGGTGGGTGT
CL12550.Contig1_All     TCATTGTGGTTGGAACTCTACATTGGAAGGTTTGAGTTTGGGTGTGCCAATGGTGGGTGT
                        ************************************************************

UGT74-cDNA              GCCTCAGTGGAGTGATCAGATGAATGATGCTAAGTTTGTGGAGGAAGTTTGGAAAGTTGG
CL12550.Contig1_All     GCCTCAGTGGAGTGATCAGATGAATGATGCTAAGTTTGTGGAGGAAGTTTGGAAAGTTGG
                        ************************************************************

UGT74-cDNA              GTATAGAGCGAAAGAGGAAGGTGGTGAAGCTATTGTGAAGAGTAAAGAGGTGGTGAGGTG
CL12550.Contig1_All     GTATAGAGCGAAAGAGGAAGGTGGTGAAGCTATTGTGAAGAGTAAAGAGGTGGTGAGGTG
                        ************************************************************

UGT74-cDNA              TTTGAAAGGAGTGATGAAAGGAGAAAGTAGTGTGAAGATTAGAGAGAGTTCGAAGAAGTG
CL12550.Contig1_All     TTTGAAAGGAGTGATGAAAGGAGAAAGTAGTGTGAAGATTAGAGAGAGTTCGAAGAAGTG
                        ************************************************************

UGT74-cDNA              GAGAGATTTGGCTGCGAAGGCAATGAGTGAAGGAGGAAGCTCTCATCGGAGTATTAACGA
CL12550.Contig1_All     GAGAGATTTGGCTGTGAAGGCAATGAGTGAAGGAGGAAGCTCTGATCGGAGTATTAACGA
                        ************** **************************** ****************

UGT74-cDNA              GTTTATAGAGAGTTTAGGGAAGAAACATTGA-----------------------------
CL12550.Contig1_All     GTTTATAGAGAGTTTAGGGAAGAAACATTGATTCAAACTTTGTAAATCACGGAATGTATG
                        *******************************

UGT74-cDNA              ------------------------------------------------------------
CL12550.Contig1_All     TTGTTGGAGGTCCACTGTTGTTGTATAAGAGCATTGTACCAAGATGATTATGATTCAATA

UGT74-cDNA              --------------------------------------------------
CL12550.Contig1_All     AACCAATTGTAATTGTGAACAAGTACAATATGTCAAAGAACTGATTAACT
```

Alignment of cDNA cloning and Illumina sequencing of *BvUGT1* gene

```
gi|393887627|gb|JQ291611.1|      ATGGTTTCCGAAATCACCCATAAATCTTATCCTCTTCACTTTGTTCTCTT
CL3716.Contig1_All               ATGGTTTCCGAAATCACCCATAAATCTTATCCTCTTCACTTTGTTCTCTT
                                 **************************************************

gi|393887627|gb|JQ291611.1|      CCCTTTCATGGCTCAAGGCCACATGATTCCCATGGTTGATATTGCAAGGC
CL3716.Contig1_All               CCCTTTCATGGCTCAAGGCCACATGATTCCCATGGTTGATATTGCAAGGC
                                 **************************************************

gi|393887627|gb|JQ291611.1|      TCTTGGCTCAGCGCGGTGTGAAAATAACAATTGTCACAACGCCGCACAAT
CL3716.Contig1_All               TCTTGGCTCAGCGCGGTGTGAAAATAACAATTGTCACAACGCCGCACAAT
                                 **************************************************

gi|393887627|gb|JQ291611.1|      GCAGCGAGGTTCGAGAATGTCCTAAGCCGTGCCATTGAGTCTGGCTTGCC
CL3716.Contig1_All               GCAGCGAGGTTCGAGAATGTCCTAAGCCGTGCCATTGAGTCTGGCTTGCC
                                 **************************************************

gi|393887627|gb|JQ291611.1|      CATCAGCATAGTGCAAGTCAAGCTTCCATCTCAAGAAGCTGGCTTACCAG
CL3716.Contig1_All               CATCAGCATAGTGCAAGTCAAGCTTCCATCTCAAGAAGCTGGCTTACCAG
                                 **************************************************

gi|393887627|gb|JQ291611.1|      AAGGAAATGAGACTTTCGATTCACTTGTCTCGATGGAGTTGCTGGTACCT
CL3716.Contig1_All               AAGGAAATGAGACTTTCGATTCACTTGTCTCGATGGAGTTGCTGGTACCT
                                 **************************************************

gi|393887627|gb|JQ291611.1|      TTCTTTAAAGCGGTTAACATGCTTGAAGAACCGGTCCAGAAGCTCTTTGA
CL3716.Contig1_All               TTCTTTAAAGCGGTTAACATGCTTGAAGAACCGGTCCAGAAGCTCTTTGA
                                 **************************************************

gi|393887627|gb|JQ291611.1|      AGAGATGAGCCCTCAACCAAGCTGTATAATTTCTGATTTTTGTTTGCCTT
CL3716.Contig1_All               AGAGATGAGCCCTCAACCAAGCTGTATAATTTCTGATTTTTGTTTGCCTT
                                 **************************************************

gi|393887627|gb|JQ291611.1|      ATACAAGCAAAATAGCCAAGAAGTTCAATATCCCAAAGATCCTCTTCCAT
CL3716.Contig1_All               ATACAAGCAAAATCGCCAAGAAGTTCAATATCCCAAAGATCCTCTTCCAT
                                 *************.************************************

gi|393887627|gb|JQ291611.1|      GGCATGTGTTGCTTTTGTCTTCTGTGTATGCATGTTTTACGCAAAAACCG
CL3716.Contig1_All               GGCATGTGTTGCTTTTGTCTTCTGTGTATGCATGTTTTACGCAAAAACCG
                                 **************************************************

gi|393887627|gb|JQ291611.1|      TGAGATCTTGGAAAACTTAAAGTCTGACAAGGAGCATTTCGTTGTTCCTT
CL3716.Contig1_All               TGAGATCTTGGAAAACTTAAAGTCTGACAAGGAGCATTTCGTTGTTCCTT
                                 **************************************************

gi|393887627|gb|JQ291611.1|      ATTTTCCTGATCGAGTTGAATTCACAAGACCTCAAGTTCCAATGGCAACA
CL3716.Contig1_All               ATTTTCCTGATCGAGTTGAATTCACAAGACCTCAAGTTCCAATGGCAACA
                                 **************************************************

gi|393887627|gb|JQ291611.1|      TATGTTCCTGGAGAGTGGCACGAGATCAAGGAGGATATAGTAGAAGCGGA
CL3716.Contig1_All               TATGTTCCTGGAGAGTGGCACGAGATCAAGGAGGATATAGTAGAAGCGGA
                                 **************************************************

gi|393887627|gb|JQ291611.1|      TAAGACTTCCTATGGTGTGATAGTCAACACATATCAAGAGCTCGAGCCTG
```

```
CL3716.Contig1_All              TAAGACTTCCTATGGTGTGATAGTCAACACATATCAAGAGCTCGAGCCTG
                                **************************************************

gi|393887627|gb|JQ291611.1|     CTTATGCCAACGACTACAAGGAGGCAAGGTCTGGTAAAGCATGGACCATT
CL3716.Contig1_All              CTTATGCCAACGACTACAAGGAGGCAAGGTCTGGTAAAGCATGGACCATT
                                **************************************************

gi|393887627|gb|JQ291611.1|     GGACCTGTTTCCTTGTGCAACAAGGTGGGAGCCGACAAAGCAGAGAGGGG
CL3716.Contig1_All              GGACCTGTTTCCTTGTGCAACAAGGTGGGAGCCGACAAAGCAGAGAGGGG
                                **************************************************

gi|393887627|gb|JQ291611.1|     AAACAAAGCAGACATTGATCAAGATGAGTGTCTTAAATGGCTTGATTCTA
CL3716.Contig1_All              AAACAAAGCAGACATTGATCAAGATGAGTGTCTTAAATGGCTTGATTCTA
                                **************************************************

gi|393887627|gb|JQ291611.1|     AAGAAGAAGGTTCGGTTCTATATGTTTGCCTTGGAAGTATCTGCAGTCTT
CL3716.Contig1_All              AAGAAGAAGGTTCGGTTCTATATGTTTGCCTTGGAAGTATCTGCAGTCTT
                                **************************************************

gi|393887627|gb|JQ291611.1|     CCTCTGTCTCAGCTCAAAGAGCTGGGGCTAGGCCTTGAGGAATCCCAAAG
CL3716.Contig1_All              CCTCTGTCTCAGCTCAAAGAGCTGGGGCTAGGCCTTGAGGAATCCCAAAG
                                **************************************************

gi|393887627|gb|JQ291611.1|     ACCTTTCATTTGGGTCGTAAGAGGTTGGGAGAAGAACAAAGAGTTACTTG
CL3716.Contig1_All              ACCTTTCATTTGGGTCGTAAGAGGTTGGGAGAAGAACAAAGAGTTACTTG
                                **************************************************

gi|393887627|gb|JQ291611.1|     AGTGGTTCTCGGATAGCGGATTTGAAGAAAGAGTAAAAGACAGAGGGCTT
CL3716.Contig1_All              AGTGGTTCTCGGAGAGCGGATTTGAAGAAAGAGTAAAAGACAGAGGGCTT
                                ************* ************************************

gi|393887627|gb|JQ291611.1|     CTCATCAAAGGATGGTCACCTCAAATGCTTATCCTTGCACATCATTCCGT
CL3716.Contig1_All              CTCATCAAAGGATGGTCACCTCAAATGCTTATCCTTGCACATCATTCCGT
                                **************************************************

gi|393887627|gb|JQ291611.1|     TGGAGGGTTCTTAACACACTGTGGATGGAACTCGACCCTCGAAGGAATCA
CL3716.Contig1_All              TGGAGGGTTCTTAACACACTGTGGATGGAACTCGACCCTCGAAGGAATCA
                                **************************************************

gi|393887627|gb|JQ291611.1|     CTTCAGGCATTCCATTGCTCACTTGGCCACTGTTTGGAGACCAATTCTGC
CL3716.Contig1_All              CTTCAGGCATTCCATTGCTCACTTGGCCACTGTTTGGAGACCAATTCTGC
                                **************************************************

gi|393887627|gb|JQ291611.1|     AACCAAAAACTTGTCGTGCAGGTGCTAAAAGTGGGTGTAAGTGCCGGGGT
CL3716.Contig1_All              AACCAAAAACTTGTCGTGCAGGTGCTAAAAGTGGGTGTAAGTGCCGGGGT
                                **************************************************

gi|393887627|gb|JQ291611.1|     TGAAGAGGTTACGAATTGGGGAGAAGAGGAGAAAATAGGAGTATTAGTGG
CL3716.Contig1_All              TGAAGAGGTTACGAATTGGGGAGAAGAGGAGAAAATAGGAGTATTAGTGG
                                **************************************************

gi|393887627|gb|JQ291611.1|     ATAAAGAGGGAGTGAAGAAGGCAGTTGAAGAATTAATGGGTGAGAGTGAT
CL3716.Contig1_All              ATAAAGAGGGAGTGAAGAAGGCAGTTGAAGAATTAATGGGTGAGAGTGAT
                                **************************************************

gi|393887627|gb|JQ291611.1|     GATGCTAAAGAAAGAAGAAAAAGAGTCAAAGAGCTTGGACAATTAGCTCA
CL3716.Contig1_All              GATGCTAAAGAAAGAAGAAAAAGAGTCAAAGAGCTTGGACAATTAGCTCA
                                **************************************************

gi|393887627|gb|JQ291611.1|     AAAGGCTGTGGAGGAAGGAGGCTCATCTCATTCTAATATCACATCCCTTC
CL3716.Contig1_All              AAAGGCTGTGGAGGAAGGAGGCTCATCTCATTCTAATATCACATCCCTTC
                                **************************************************

gi|393887627|gb|JQ291611.1|     TAGAAGACATAATGCAACTAGCACAATCTAATAATTGATTTTGTACGTCA
CL3716.Contig1_All              TAGAAGACATAATGCAACTAGCACAATCTAATAAT---------------
                                ***********************************

gi|393887627|gb|JQ291611.1|     TTATTAAAAAATTTCTTTAATGGATGAAGTTAAATCGGGGCCTTAGTCAA
CL3716.Contig1_All              --------------------------------------------------

gi|393887627|gb|JQ291611.1|     AAAAAAAAAAAAAAAAAA
CL3716.Contig1_All              ------------------
```

Alignment of cDNA cloning and Illumina sequencing *UGT73C11* of gene

```
gi|393887641|gb|JQ291614.1|     GAGTCTTATTAGAGCTGCTTCGTCATGGTTTCCGAAATCACCCATAAATC
CL3716.Contig2_All              ------------------------ATGGTTTCCGAAATCACCCATAAATC
                                                        **************************

gi|393887641|gb|JQ291614.1|     TTATCCTCTTCACTTTGTTCTCTTCCCTTTCATGGCTCAAGGCCACATGA
CL3716.Contig2_All              TTATCCTCTTCACTTTGTTCTCTTCCCTTTCATGGCTCAAGGCCACATGA
```

```
                                **************************************************

gi|393887641|gb|JQ291614.1|      TTCCCATGGTTGATATTGCAAGGCTCTTGGCTCAGCGCGGTGTGAAAATA
CL3716.Contig2_All               TTCCCATGGTTGATATTGCAAGGCTCTTGGCTCAGCGCGGTGTGAAAATA
                                 **************************************************

gi|393887641|gb|JQ291614.1|      ACAATTGTCACAACGCCGCACAATGCAGCGAGGTTCGAGAATGTCCTAAG
CL3716.Contig2_All               ACAATTGTCACAACGCCGCACAATGCAGCGAGGTTCGAGAATGTCCTAAG
                                 **************************************************

gi|393887641|gb|JQ291614.1|      CCGTGCCATTGAGTCTGGCTTGCCCATCAGCATAGTGCAAGTCAAGCTTC
CL3716.Contig2_All               CCGTGCCATTGAGTCTGGCTTGCCCATCAGCATAGTGCAAGTCAAGCTTC
                                 **************************************************

gi|393887641|gb|JQ291614.1|      CATCTCAAGAAGCTGGCTTACCAGAAGGAAATGAGACTTTCGATTCACTT
CL3716.Contig2_All               CATCTCAAGAAGCTGGCTTACCAGAAGGAAATGAGACTTTCGATTCACTT
                                 **************************************************

gi|393887641|gb|JQ291614.1|      GTCTCGATGGAGTTGCTGGTACCTTTCTTTAAAGCGGTTAACATGCTTGA
CL3716.Contig2_All               GTCTCGATGGAGTTGCTGGTACCTTTCTTTAAAGCGGTTAACATGCTTGA
                                 **************************************************

gi|393887641|gb|JQ291614.1|      AGAACCGGTCCAGAAGCTCTTTGAAGAGATGAGCCCTCAACCAAGCTGTA
CL3716.Contig2_All               AGAACCGGTCCAGAAGCTCTTTGAAGAGATGAGCCCTCAACCAAGCTGTA
                                 **************************************************

gi|393887641|gb|JQ291614.1|      TAATTTCTGATTTTTGTTTGCCTTATACAAGCAAAATAGCCAAGAAGTTC
CL3716.Contig2_All               TAATTTCTGATTTTTGTTTGCCTTATACAAGCAAAATCGCCAAGAAGTTC
                                 *************************************.************

gi|393887641|gb|JQ291614.1|      AATATCCCAAAGATCCTCTTCCATGGCATGTGTTGCTTTTGTCTTCTGTG
CL3716.Contig2_All               AATATCCCAAAGATCCTCTTCCATGGCATGTGTTGCTTTTGTCTTCTGTG
                                 **************************************************

gi|393887641|gb|JQ291614.1|      TATGCATGTTTTACGCAAAAACCGTGAGATCTTGGAAAACTTAAAGTCTG
CL3716.Contig2_All               TATGCATGTTTTACGCAAAAACCGTGAGATCTTGGAAAACTTAAAGTCTG
                                 **************************************************

gi|393887641|gb|JQ291614.1|      ACAAGGAGCATTTCGTTGTTCCTTATTTTCCTGATCGAGTTGAATTCACA
CL3716.Contig2_All               ACAAGGAGCATTTCGTTGTTCCTTATTTTCCTGATCGAGTTGAATTCACA
                                 **************************************************

gi|393887641|gb|JQ291614.1|      AGACCTCAAGTTCCAATGGCAACATATGTTCCTGGAGAGTGGCACGAGAT
CL3716.Contig2_All               AGACCTCAAGTTCCAATGGCAACATATGTTCCTGGAGAGTGGCACGAGAT
                                 **************************************************

gi|393887641|gb|JQ291614.1|      CAAGGAGGATATAGTAGAAGCGGATAAGACTTCCTATGGTGTGATAGTCA
CL3716.Contig2_All               CAAGGAGGATATAGTAGAAGCGGATAAGACTTCCTATGGTGTGATAGTCA
                                 **************************************************

gi|393887641|gb|JQ291614.1|      ACACATATCAAGAGCTCGAGCCTGCTTATGCCAACGACTACAAGGAGGCA
CL3716.Contig2_All               ACACATATCAAGAGCTCGAGCCTGCTTATGCCAACGACTACAAGGAGGCA
                                 **************************************************

gi|393887641|gb|JQ291614.1|      AGGTCTGGTAAAGCATGGACCATTGGACCTGTTTCCTTGTGCAACAAGGT
CL3716.Contig2_All               AGGTCTGGTAAAGCATGGACCATTGGACCTGTTTCCTTGTGCAACAAGGT
                                 **************************************************

gi|393887641|gb|JQ291614.1|      GGGAGCCGACAAAGCAGAGAGGGGAAACAAAGCAGACATTGATCAAGATG
CL3716.Contig2_All               GGGAGCCGACAAAGCAGAGAGGGGAAACAAAGCAGACATTGATCAAGATG
                                 **************************************************

gi|393887641|gb|JQ291614.1|      AGTGTCTTAAATGGCTTGATTCTAAAGAAGAAGGTTCGGTTCTATATGTT
CL3716.Contig2_All               AGTGTCTTAAATGGCTTGATTCTAAAGAAGAAGGTTCGGTTCTATATGTT
                                 **************************************************

gi|393887641|gb|JQ291614.1|      TGCCTTGGAAGTATCTGCAGTCTTCCTCTGTCTCAGCTCAAAGAGCTGGG
CL3716.Contig2_All               TGCCTTGGAAGTATCTGCAGTCTTCCTCTGTCTCAGCTCAAAGAGCTGGG
                                 **************************************************

gi|393887641|gb|JQ291614.1|      GCTAGGCCTTGAGGAATCCCAAAGACCTTTCATTTGGGTCGTAAGAGGTT
CL3716.Contig2_All               GCTAGGCCTTGAGGAATCCCAAAGACCTTTCATTTGGGTCGTAAGAGGTT
                                 **************************************************

gi|393887641|gb|JQ291614.1|      GGGAGAAGAACAAAGAGTTACTTGAGTGGTTCTCGGAGAGCGGATTTGAA
CL3716.Contig2_All               GGGAGAAGAACAAAGAGTTACTTGAGTGGTTCTCGGAGAGCGGATTTGAA
                                 **************************************************

gi|393887641|gb|JQ291614.1|      GAAAGAGTAAAAGACAGAGGGCTTCTCATCAAAGGATGGTCACCTCAAAT
CL3716.Contig2_All               GAAAGAGTAAAAGACAGAGGGCTTCTCATCAAAGGATGGTCACCTCAAAT
                                 **************************************************

gi|393887641|gb|JQ291614.1|      GCTTATCCTTGCACATCATTCCGTTGGAGGGTTCTTAACACACTGTGGAT
CL3716.Contig2_All               GCTTATCCTTGCACATCATTCCGTTGGAGGGTTCTTAACACACTGTGGAT
                                 **************************************************

gi|393887641|gb|JQ291614.1|      GGAACTCGACCCTCGAAGGAATCACTTCAGGCATTCCATTGCTCACTTGG
```

```
CL3716.Contig2_All               GGAACTCGACCCTCGAAGGAATCACTTCAGGCATTCCATTGCTCACTTGG
                                 **************************************************

gi|393887641|gb|JQ291614.1|      CCACTGTTTGGAGACCAATTCTGCAACCAAAAACTTGTCGTGCAGGTGCT
CL3716.Contig2_All               CCACTGTTTGGAGACCAATTCTGCAACCAAAAACTTGTCGTGCAGGTGCT
                                 **************************************************

gi|393887641|gb|JQ291614.1|      AAAAGTGGGTGTAAGTGCCGGGGTTGAAGAGGTTACGAATTGGGGAGAAG
CL3716.Contig2_All               AAAAGTGGGTGTAAGTGCCGGGGTTGAAGAGGTTACGAATTGGGGAGAAG
                                 **************************************************

gi|393887641|gb|JQ291614.1|      AGGAGAAAATAGGAGTATTAGTGGATAAAGAGGGAGTGAAGAAGGCAGTT
CL3716.Contig2_All               AGGAGAAAATAGGAGTATTAGTGGATAAAGAGGGAGTGAAGAAGGCAGTT
                                 **************************************************

gi|393887641|gb|JQ291614.1|      GAAGAATTAATGGGTGAGAGTGATGATGCTAAAGAAAGAAGAAAAAGAGT
CL3716.Contig2_All               GAAGAATTAATGGGTGAGAGTGATGATGCTAAAGAAAGAAGAAAAAGAGT
                                 **************************************************

gi|393887641|gb|JQ291614.1|      CAAAGAGCTTGGACAATTAGCTCAAAAGGCTGTGGAGGAAGGAGGCTCAT
CL3716.Contig2_All               CAAAGAGCTTGGACAATTAGCTCAAAAGGCTGTGGAGGAAGGAGGCTCAT
                                 **************************************************

gi|393887641|gb|JQ291614.1|      CTCATTCTAATATCACATCCTTGCTAGAAGACATAATGCAACTAGCACAA
CL3716.Contig2_All               CTCATTCTAATATCACATCCCTTCTAGAAGACATAATGCAACTAGCACAA
                                 ******************** * ***************************

gi|393887641|gb|JQ291614.1|      TCTAATAATTGATTTTGTACGTCATTATTAAAAAATTTCTTTAATGGATG
CL3716.Contig2_All               TCTAATAAT-----------------------------------------
                                 *********

gi|393887641|gb|JQ291614.1|      AAGTTAAATCGGGGCCT
CL3716.Contig2_All               -----------------
```
